# Microglia Are Mediators of *Borrelia burgdorferi*–Induced Apoptosis in SH-SY5Y Neuronal Cells

**DOI:** 10.1371/journal.ppat.1000659

**Published:** 2009-11-13

**Authors:** Tereance A. Myers, Deepak Kaushal, Mario T. Philipp

**Affiliations:** Division of Bacteriology & Parasitology, Tulane National Primate Research Center, Tulane University Health Sciences Center, Louisiana, United States of America; Medical College of Wisconsin, United States of America

## Abstract

Inflammation has long been implicated as a contributor to pathogenesis in many CNS illnesses, including Lyme neuroborreliosis. *Borrelia burgdorferi* is the spirochete that causes Lyme disease and it is known to potently induce the production of inflammatory mediators in a variety of cells. In experiments where *B. burgdorferi* was co-cultured *in vitro* with primary microglia, we observed robust expression and release of IL-6 and IL-8, CCL2 (MCP-1), CCL3 (MIP-1α), CCL4 (MIP-1β) and CCL5 (RANTES), but we detected no induction of microglial apoptosis. In contrast, SH-SY5Y (SY) neuroblastoma cells co-cultured with *B. burgdorferi* expressed negligible amounts of inflammatory mediators and also remained resistant to apoptosis. When SY cells were co-cultured with microglia and *B. burgdorferi*, significant neuronal apoptosis consistently occurred. Confocal microscopy imaging of these cell cultures stained for apoptosis and with cell type-specific markers confirmed that it was predominantly the SY cells that were dying. Microarray analysis demonstrated an intense microglia-mediated inflammatory response to *B. burgdorferi* including up-regulation in gene transcripts for TLR-2 and NFκβ. Surprisingly, a pathway that exhibited profound changes in regard to inflammatory signaling was triggering receptor expressed on myeloid cells-1 (TREM1). Significant transcript alterations in essential p53 pathway genes also occurred in SY cells cultured in the presence of microglia and *B. burgdorferi*, which indicated a shift from cell survival to preparation for apoptosis when compared to SY cells cultured in the presence of *B. burgdorferi* alone. Taken together, these findings indicate that *B. burgdorferi* is not directly toxic to SY cells; rather, these cells become distressed and die in the inflammatory surroundings generated by microglia through a bystander effect. If, as we hypothesized, neuronal apoptosis is the key pathogenic event in Lyme neuroborreliosis, then targeting microglial responses may be a significant therapeutic approach for the treatment of this form of Lyme disease.

## Author Summary

Lyme disease, which is transmitted to humans through the bite of a tick, is currently the most frequently reported vector-borne illness in the northern hemisphere. *Borrelia burgdorferi* is the bacterium that causes Lyme disease and it is known to readily induce inflammation within a variety of infected tissues. Many of the neurological signs and symptoms that may affect patients with Lyme disease have been associated with *B. burgdorferi*-induced inflammation in the central nervous system (CNS). In this report we investigated which of the primary cell types residing in the CNS might be functioning to create the inflammatory environment that, in addition to helping clear the pathogen, could simultaneously be harming nearby neurons. We report findings that implicate microglia, a macrophage cell type in the CNS, as the key responders to infection with *B. burgdorferi.* We also present evidence indicating that this organism is not directly toxic to neurons; rather, a bystander effect is generated whereby the inflammatory surroundings created by microglia in response to *B. burgdorferi* may themselves be toxic to neuronal cells.

## Introduction

Lyme borreliosis is the most prevalent vector-borne illness in the northern hemisphere [1],[2]. Transmission from the animal reservoir to the human host occurs via an Ixodes tick bite into the skin, where *B. burgdorferi*, the spirochete that causes Lyme disease, can then disseminate hematogenously to various organs, including the heart, joints and both the peripheral and central nervous systems [2]–[4]. A prominent, recurring and yet only partially understood feature of Lyme disease is the presence of inflammatory infiltrates within infected tissues [1]–[5]. Evidence of neurological involvement occurs to varying degrees in both the central and peripheral nervous systems of Lyme disease patients, and inflammation is often associated with the neurological manifestations that define neuroborreliosis [2], [5]–[8],[9],[10]. Upon gaining access to the central nervous system (CNS), *B. burgdorferi* may induce cerebrospinal fluid pleocytosis, meningoradiculitis and cranial neuritis as well as encephalopathies with neurocognitive abnormalities [3],[5],[6],[11]. This complex process of *B. burgdorferi-*induced pathology in the CNS has many aspects as yet to be clarified, with reactive inflammation potentially being one of the principal contributors to neuronal dysfunction.

Although *B. burgdorferi* lacks lipopolysaccharide (LPS), the organism does produce spirochetal lipoproteins that can induce inflammation [11]–[17]. By signaling through CD14, binding Toll-like receptors (TLR) 2 and 1 and subsequent activation of NFκβ, lipoproteins have been shown to generate inflammatory mediators in a variety of cell types [14], [18]–[21]. Recent studies with TLR2 and/or MyD88-deficient mice in which *B. burgdorferi-*induced inflammatory infiltrates were greater than that of wild type controls, indicated however, that there are alternative pathways regulating the inflammatory response to infection with *B. burgdorferi* [11],[22],[23]. As neuronal dysfunction is further analyzed in the light of these findings, it becomes necessary to consider which cells of the CNS milieu can function most aggressively/efficiently in creating an inflammatory environment that will contribute to clearance of the pathogen, but may in the process, harm nearby neurons.

Using *in vitro* and *ex vivo* experiments, investigators have shown that *B. burgdorferi* can induce potent production of inflammatory cytokines and chemokines in microglia, the resident macrophage cells of the CNS [2],[6],[12],[24]. The secretion of these mediators is likely a vital step in the development of inflammatory reactions, as chemokines, depending on their CC/CXC family sequence, may recruit distinct immune-effector cells, including monocytes, lymphocytes or neutrophils to sites of inflammation. Additionally, many cytokines and chemokines become involved in apoptosis, cell cycle regulation and angiogenesis [3], [6], [23], [25]–[28]. While a similar response is observed in astrocytes, the repertoire range and scale of concentration is typically much lower than that of activated microglia [2],[12],[29]. Importantly, although astrocytes are considered to be the primary CNS support cells, activated microglia can also secrete a host of soluble agents, such as glia-derived neurotrophic factor, that are potentially neuroprotective [30]–[32]. As the majority of molecules produced by activated microglia are however considered to be pro-inflammatory and neurotoxic [2],[30],[31],[33], the microglial response to CNS infection with *B. burgdorferi* could tip the scales toward neuronal damage and death rather than survival in Lyme neuroborreliosis.

We have argued that the associated neurocognitive symptoms of Lyme neuroborreliosis may be the result of neuronal dysfunction resulting from inflammatory mediators released in response to infection with *B. burgdorferi*. Serving as an *in vivo* proof of concept to this hypothesis, our laboratory has recently shown the production of IL-6 by astrocytes, as well as the induction of oligodendrocyte and neuronal apoptosis in brain tissues taken from rhesus macaques that received intraparenchymal stereotaxic inoculations of live *B. burgdorferi* [6]. With the goal of beginning to establish cause and effect relationships between glial cell responses to *B. burgdorferi* and neuronal apoptosis, we quantified the production/secretion of inflammatory cytokines and chemokines in purified rhesus brain cortex microglia and astrocytes, in human neuronal cells from the SH-SY5Y (SY) neuroblastoma cell line, and in combinations of the above cells co-cultured with live *B. burgdorferi* or recombinant purified lipidated outer-surface protein A (L-OspA). SY cells were cultured in three dimensions as opposed to monolayer culture, as we had shown that that mode of cultivation significantly narrows the phenotypic gap between neuronal cell lines and primary neurons [34]. We also determined the extent of *B. burgdorferi-*induced apoptosis in each of the above cellular combinations. Using microarray analysis, we further examined the principal inflammation and apoptosis pathways affected by *B. burgdorferi* in these cells. Our findings suggest a bystander effect in which the neurotoxic surroundings generated by microglia may contribute to neuronal cell damage.

## Materials and Methods

### Ethics statement

Brain tissues used in this study were collected from rhesus macaques (*Macaca mulatta*). These animals were not experimentally infected with *B. burgdorferi* and were culled from the breeding colony because of chronic diarrhea or injury. The procedure used for euthanasia was consistent with the recommendations of the American Veterinary Medical Association's Panel on Euthanasia and was approved by Tulane University's Institutional Animal Care and Use Committee.

### Media and reagents

Dulbecco's modified Eagle's medium (D-MEM)-F-12 with _L_-glutamine and 15 mM HEPES buffer, D-MEM high glucose with _L_-glutamine, F-12 (Ham) with glutamax, penicillin (100 units/ml), streptomycin (100 units/ml), amphotericin B (0.25 µg/ml), non essential amino acids (NEAA) (100 units/ml), sodium pyruvate (100 units/ml), sodium bicarbonate (7.5% solution), trypsin (0.25%)/EDTA (0.38 g/ml), trypan blue™, normal goat serum (NGS), Alexa-562 (red)-conjugated secondary antibody, and the ToPro (blue) nuclear stain were each from Invitrogen. Primocin was from Invivogen. Fetal bovine serum was from Hyclone/Thermo Scientific and Cytodex-3™ micro-carrier beads were from Amersham Biosciences. Granulocyte-macrophage colony-stimulating factor (GM-CSF), L-leucine methyl ester (LME), Barbour-Stoenner-Kelly-H medium with rabbit serum, rifampicin, amphotericin, fish skin gelatin (FSG), propidium iodide (PI) (red) and anti-GFAP antibody were from Sigma-Aldrich. Lipidated (L-OspA) and unlipidated (U-OspA) outer surface protein A were a kind gift from GlaxoSmithKline. Paraformaldehyde (PFA, 2%) was from USB Corporation, and Triton X-100 from ICN Biochemicals. HuD primary antibody was from Santa Cruz Biotechnology. IBA-1 antibody was from WAKO.

### Cell isolation and culture

#### Primary glia

Brain tissues used in this study were collected from rhesus macaques (*Macaca mulatta*). These animals were not experimentally infected with *B. burgdorferi* and were culled from the breeding colony because of chronic diarrhea or injury. Tissue was removed from the cortical region of the brain and processed as follows. After removal of the leptomeninges and leptomeningeal blood vessels, the cortex tissue was first dissociated mechanically by mincing the tissue with a sterile scalpel. Enzymatic dissociation followed by resuspending the minced tissue in D-MEM-F-12 medium with 0.25% trypsin-EDTA, 0.1% DNase and primocin (100 µg/ml), at 37°C for approximately 30 minutes, with occasional manual shaking. The solution was then centrifuged (425 x g, 10 minutes), the upper layer of cells removed, passed through a 20 µm Nitex filter and resuspended in D-MEM-F-12 with 10% FBS, 0.5 ng/ml GM-CSF and primocin antibiotic/antimycotic. After an incubation of approximately 10 days to 4 weeks at 37° in a 5% CO2, humid atmosphere incubator, microglia could be dislodged and recovered by vigorously tapping the culture flasks. The astrocyte cells were purified of any remaining microglia by removal of the GM-CSF component from the medium and subsequent treatment with 25 mM L-leucine methyl ester (LME) for 60 minutes at room temperature. Purity of both the astrocyte and microglial cell cultures was assessed to be approximately 98 percent and was confirmed by staining with GFAP and IBA-1. The culture was considered an aggregate of astrocytes and microglia before being purified for either specific cell type.

#### Neuroblastoma cells

Human SY neuroblastoma cells [American Type Culture Collection (ATCC) CRL-2266] were seeded into T75 flasks with medium renewal every 3–7 days. Cell propagation was performed as per the ATCC product sheet. Three-dimensional (3-D) cultures were then prepared as previously described [34]. For any SY cell co-culture with glia or *B. burgdorferi*, SY cell culture complete growth medium was used. This medium was composed of a 1∶1 mixture of D-MEM high glucose with L-glutamine and F-12 medium. Penicillin, streptomycin and amphotericin B were added to the medium only when spirochetes were not present. Trypsin/EDTA was used to dislodge the cells in both neuronal and glia cultures, and trypan blue stain was used to assess cell viability.

#### Bacterial culture

*B. burgdorferi* was cultured as previously described [12] with limited modifications. When combined in any co-culture with glia and/or SY cells, the described appropriate mammalian cell culture medium was used, but without the addition of antibiotics and antimycotic. While *B. burgdorferi* did not divide in any of the mammalian culture media used, spirochetal viability was determined to be above 85% in all assays. The LIVE/DEAD BacLight bacterial viability kit (Invitrogen) was used as per the manufacturer's instructions, to determine viability at both 24 hours and 5 days during co-culture with glia and/or SY cells. *B. burgdorferi* was additionally cultured in the mammalian cell medium alone to confirm proliferation rates and viability.

### Transwell culture

Three-micrometer pore diameter polyester transwell culture inserts (Becton Dickinson, Falcon) were incorporated into our co-culture models to physically separate SY cells from microglia. The SY cells were seeded directly into 24 well dishes (Costar) with the microglia then seeded onto culture inserts that had been placed into the same wells as the SY cells. The cell density in these experiments was 1×10^5^ cells per ml of culture medium with an initial microglia to neuronal cell ratio of 4∶1. Live *B. burgdorferi* was added to the culture medium in both chambers for a 5-day stimulation, at an MOI of 10∶1, in relation to the entire cell population. In this way, it was possible to maintain a generally homogeneous exposure of each cell type to the culture medium while still allowing for bidirectional transfer of secreted molecules between the cell types.

### Quantification of cytokines

Supernatants collected from primary glia and/or SY cells co-cultured with *B. burgdorferi* (MOI 10∶1) or in medium alone for either 24 hours or 5 days were used for quantification of secreted cytokines and chemokines. All of the mammalian cells were seeded at a density of 5×10^4^ cells per 500 µl of culture medium with a glial cell to SY cell ratio of 4∶1. The 27-cytokine bioplex assays (Bio-Rad) were performed according to manufacturer's directions as were the individual antigen-capture ELISAs for IL-6, IL-8, TNF, CCL3, CCL4, and MCP-1 (CCL2). Sandwich ELISA capture and detection antibody pairs for human IL-6, IL-8 and TNF, along with recombinant human IL-6, IL-8, TNF and horseradish peroxidase (HRP) were from BD Biosciences. We obtained CCL3 and CCL4 ELISA DuoSet kits and the MCP-1 (CCL2) kit from R&D Systems.

### Determination of nitric oxide production

Supernatants collected from primary glia and/or SY cells co-cultured with *B. burgdorferi* (MOI 10∶1) or in medium alone for either 24 hours or 5 days were used for quantitative determination of total secreted nitric oxide. The mammalian cells were seeded at a density of 5×10^4^ cells per 500 µl of culture medium with an initial glial cell to SY cell ratio of 4∶1. The Total Nitric Oxide Assay Kit (catalogue #917–020) from Assay Designs was used, and all analyses were performed according to directions from the manufacturer.

### Apoptosis assays, confocal microscopy

Microglia, astrocytes, SY cells or combinations thereof were cultured in the presence of *B. burgdorferi* (MOI 10∶1) or medium alone for either 24 hours or 5 days. After removal of the supernatant, the cells were harvested using trypsin, washed in phosphate buffered saline (PBS), and fixed for 5–10 minutes in 2% PFA. The fixed cells were permeabilized in PBS/FSG/Triton and blocked with 10% NGS. Apoptosis was evaluated using the Apoptag TUNEL assay kit (Chemicon/Millipore) as per manufacturer's instructions and the results were analyzed using a Leica TCS SP2 confocal microscope equipped with 3 lasers. Briefly, 6–18 0.2-µm optical slices per image were collected at 512×512 pixel resolution. In order to distinguish SY cells in the co-cultures containing glia, the cells were additionally stained with primary anti-HuD antibody for 1 hour, washed 3 times in PBS and then stained with Alexa 562-labeled secondary antibody for 45 minutes. The To-Pro nuclear stain was combined with the secondary antibody at a concentration equal to 0.05 µg/ml. The identities of microglia and astrocytes were additionally confirmed using the above protocol, substituting anti-IBA-1 and anti-GFAP respectively, for the anti-HuD. Cell morphology consistent with apoptosis including cell shrinkage, nuclear condensation and membrane blebbing was assessed along with the fluorescein staining for TUNEL. The number of apoptotic cells counted was divided by the total (500 minimum) number of cells counted. When the assays included co-culture of SY cells with glia, the number of cells that were double- stained for apoptosis and neuron specificity were divided by the total number of cells displaying the neuronal marker stain. Statistical significance was evaluated by One Way analysis of variance (ANOVA) followed by Bonferroni, Tukey and Levine's tests.

### Microarray analysis

RNA was isolated from approximately 5×10^6^ isolated SY cells or microglia using the RNeasy kit (Qiagen) plus DNA-*free* (Ambion) to eliminate DNA contamination. Five hundred nanogram of total RNA was amplified and used to synthesize Cy-labeled cDNA with the Low RNA Input Linear Amplification Kit (LRILAK, Agilent Technologies). Cy3 (control) and Cy5 (experimental) labeled cDNA were mixed in equimolar quantities and hybridized overnight at 55°C, to Agilent whole-genome microarrays (4×44 k format). While the samples derived from macaque microglia were hybridized to rhesus macaque arrays (Agilent # G2519F) with over 44,000 rhesus macaque probes, representing approximately 18,000 individual annotated genes, the samples derived from the neuronal cells were hybridized to human genome arrays (Agilent # G4112F) with over 41,000 oligonucleotides, representing approximately 22,000 unique human genes. The slides were scanned on a GenePix 4000B scanner, and data were extracted from the resulting 16-bit TIFF images using GenePix Pro 6.1 software. Data were analyzed using Spotfire DecisionSite for Microarray Analysis. Values were log2 transformed and normalized using a Locally Weighted ScatterPlot Smoothing (LOWESS) script within S+ ArrayAnalyzer.

### QRT-PCR

RNA was isolated from approximately 5×10^6^ isolated microglia or neuronal cells using the RNeasy kit (Qiagen) plus DNA-*free* (Ambion) to eliminate DNA contamination. The RNA was reverse-transcribed into DNA using a OneStep RT-PCR kit (Qiagen) and the QuantiFast™ SYBR® Green PCR kit (Qiagen) was then used for the quantitative real-time (QRT)-PCR. All assays were performed according to directions from the manufacturer and using Qiagen Quantitect® primer pairs in a 96-well block Applied Biosystems 7900 HT fast RT PCR System. PCR efficiencies, average fold change and statistical significance were evaluated using REST© software.

## Results

### *B. burgdorferi* upregulates the expression of inflammatory cytokines/chemokines in primary rhesus glia but not in SY neurons

**Figure 1:**
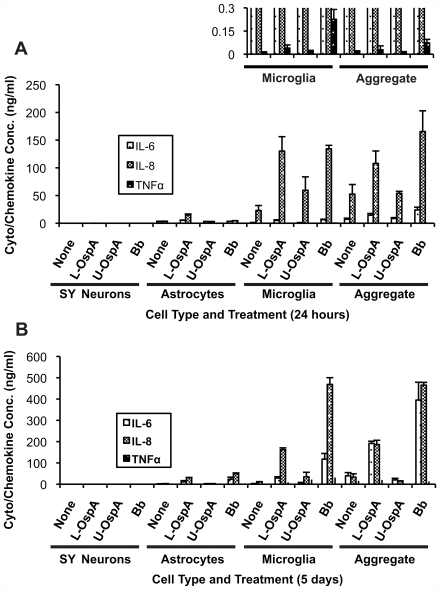
*B. burgdorferi* induces the expression of inflammatory mediators in primary rhesus CNS glial cells but not in SY cells. (A) Sandwich ELISA shows prominent increases in IL-6 and IL-8 expression/secretion by primary cultures of rhesus cortex glia in the presence of lipidated outer-surface protein A (L-OspA): 0.25 µg/ml, or *B. burgdorferi* (*Bb*; MOI of 10∶1), for 24 hours, and (B) for 5 days. Unlipidated outer-surface protein A (U-OspA): 0.25 µg/ml, was used as the control for L-OspA. While a slight U-OspA-induced increase in IL-8 expression was observed in the microglia samples at 24 hours, this increase was not statistically different from control values. TNF expression was only observed in cultures containing microglia, where it peaked at 24 hours and was not observed at 5 days. Neuron responses were absent or negligible in comparison to that of the glial cells at both time points. In order to better visualize TNF expression, the Y axis in Figure 1A was expanded (insert). Values on each graph are from a single representative animal where depending on the stimulus and time of incubation, each experiment was repeated in 3 or more rhesus macaques. Error bars represent standard deviation of replicates on the plate where n = 3.

In experiments where *B. burgdorferi* or recombinant L-OspA was co-cultured with isolated rhesus cortex glia, we observed robust expression and release of IL-6 and IL-8 (Figure 1A). We also observed the production of TNF, although at a much lower level, and only by microglial cells and cells in the aggregate cultures, which themselves contained microglia (Figure 1A insert). SY cell production of IL-6 and IL-8 in response to *B. burgdorferi* and L-OspA were below the limit of detection for the assay used. Cytokine/chemokine expression levels in response to the same stimuli often varied significantly in glial cells obtained from different animals. The patterns of expression, however, were reproducible in experiments where the glial cells were isolated from tissue originating from a single individual animal. As such, the highest response was always that of microglia, followed by that of aggregate cultures and then astrocytes, regardless of the animal from whom the cells had been obtained. With the exception of TNF, whose expression peaked at 24 hours and then declined, levels of cytokine/chemokine expression also increased with the time of stimulation (Figure 1B).

As the inherent difficulties in culturing primary neurons [35],[36] often render their use in experiments impractical, the neuroblastoma cell line SH-SY5Y was employed to assess neuronal responses. We used a three-dimensional (3D) rather than traditional monolayer (2D) culture for the SY cells, as this culture method has been shown to promote a more normal, untransformed phenotype as compared to that of transformed cells grown in 2D [34], [35], [37]–[45].

### Neuronal apoptosis occurs in the inflammatory environment created by microglia in response to *B. burgdorferi*

**Figure 2:**
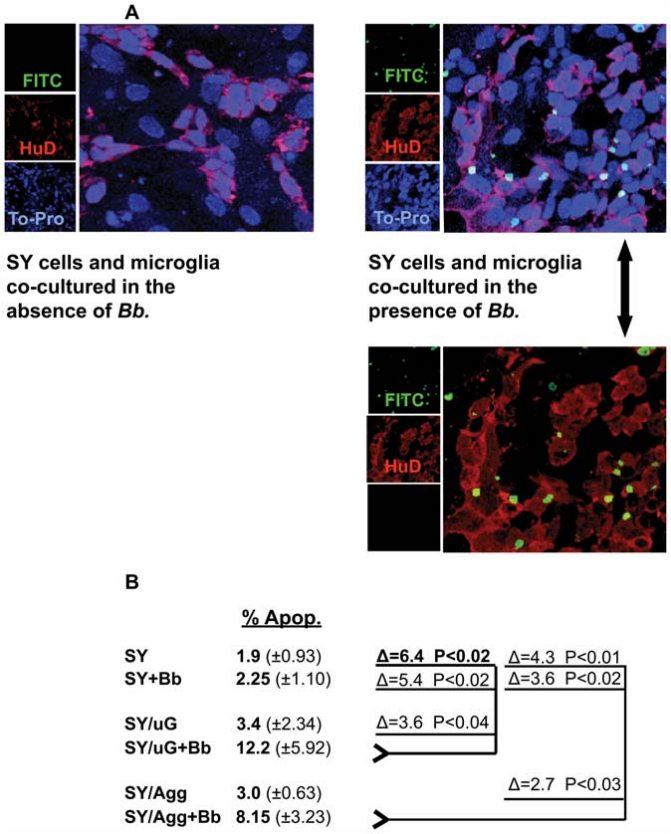
Neuronal apoptosis occurs in the inflammatory environment created by microglia in response to *B. burgdorferi*. (A) Cell specific markers and a separate nuclear stain were combined with the TUNEL assay. TUNEL: FITC (green), neuron specific marker: HuD (red), nucleus specific marker: To-Pro (blue). Confocal microscopy was used to determine that the majority of cells dying by apoptosis in response to *B. burgdorferi* were neurons. An accurate total cell number per field was established by counting all of the individual nuclei (blue). An on/off toggle of the laser channel for the nuclear stain (blue), would allow easy detection of overlap between the cell specific (red) and TUNEL (green) markers (lower panel). As the SY neuronal cells tend to grow in clusters, multiple panels were counted in order to acquire a proper percentage of each cell type. Total cell counts typically ranged between 500 and 1000. (B) In experiments focused on SY cells co-cultured with combinations of both microglia (µG) and *B. burgdorferi* (*Bb*), significant increases in SY, but not glial apoptosis consistently occurred. Cell culture in these experiments was performed as above, but the microglia and aggregate (Agg) cultures were obtained from 4 individual animals. Data represent the mean (n = 4) ± S.D. Δ  =  change. P values were obtained using One Way analysis of variance (ANOVA) followed by Bonferroni, Tukey and Levine's tests.

In order to assess endpoint damage to the glia and SY cells responding to co-culture with *B. burgdorferi* or L-OspA, we employed the terminal deoxynuclease dUTP nick end labeled (TUNEL) assay as a tool for visualization of apoptosis. When microglia isolated either alone or in aggregate with astrocyte cells, were co-cultured with both *B. burgdorferi* and SY for at least 5 days, increases in cellular apoptosis consistently occurred. Apoptosis in glial cells was minimal as compared to the un-stimulated controls and there were no remarkable changes in apoptotic levels with regard to individual animals. SY cells cultured for 5 days with *B. burgdorferi* alone, or in combination with astrocytes and *B. burgdorferi*, showed only baseline levels of apoptosis (Table 1). No significant increase in astrocyte apoptosis was observed whether these cells were incubated with other cell types, L-OspA or *B. burgdorferi* (Table 1). Confocal microscopy images of mixed cultures stained for TUNEL and with cell-specific markers indicated that the majority of cells dying in response to *B. burgdorferi* were SY cells (Figure 2A). In consideration of these findings, we focused our next experiments on SY cells cultured in the presence of microglial cells and *B. burgdorferi* for 5 days. Significant increases in SY cell apoptosis occurred consistently in cell cultures from each of the 4 animals sampled when both microglia and *B. burgdorferi* were included in the culture conditions (Figure 2B).

**Table 1:** *Borrelia burgdorferi-*induced apoptosis in SH-SY5Y (SY) neurons and primary rhesus glia.

	%Apop in Single Cell Culture	%Apop in Glia+SY Culture (Glia)	%Apop in Glia+SY Culture (SY)
**SY**	1.6		
**SY+L-OspA**	2.1		
**SY+U-OspA**	1.9		
**SY+** ***Bb***	1.6		
**A**	0.2	1.5	1.6
**A+L-OspA**	0.4	0.7	1.6
**A+U-OspA**	0.3	3.4	3.8
**A+** ***Bb***	0.6	1.5	2.0
**μG**	0.9	3.7	5.4
**μG+L-OspA**	0.5	0.5	2.3
**μG+U-OspA**	1.9	0.8	1.0
**μG+** ***Bb***	1.1	3.6	**14.8**
**Agg**	0.7	0.8	2.4
**Agg+L-OspA**	0.2	1.4	1.5
**Agg+U-OspA**	0.2	0.9	1.9
**Agg+** ***Bb***	0.6	3.4	**12.0**

The TUNEL assay was used to measure apoptosis in combinations of neurons and glia that were cultured for 5 days in medium alone, or in the presence of lipidated outer-surface protein A (L-OspA) (0.25 µg/ml), Un-lipidated outer surface protein A (U-OspA) (0.25 µg/ml) or *Borrelia burgdorferi (Bb)* at a multiplicity of infection (MOI) 10∶1. Induction of apoptosis above baseline occurred only in the neurons and importantly, only in those that were co-cultured in the presence of both *Bb* and microglia. Values shown are from a single representative animal. SY: SHSY5Y “n” type neuroblastoma cells; A: astrocytes; μG: microglia; Agg: astrocytes and microglia in proportion of original primary culture.

In order to determine whether stimulation of the culture medium with IL-6, IL-8, TNF or combinations thereof would be sufficient to induce apoptosis in the SY cells, we conducted experiments in which concentrations of each cytokine (recombinant-human) were added to the neuronal medium that were comparable to the highest expression values obtained during our *in vitro* assays with microglia and SY cells. After a 5 day stimulation, we did not find increases in neuronal apoptosis comparable to those observed in the co-culture of SY cells with microglia and *B. burgdorferi*, or significantly above the baseline levels of SY cells cultured in medium alone (data not shown).

### Potent and sustained expression of the chemokines CCL2, CCL3, CCL4 and CCL5, in addition to that of IL-6 and IL-8, contributes to maintaining the inflammatory environment created by microglia in response to *B. burgdorferi*

In view of the evidence indicating that microglia were the more robust responders to the inflammatory stimuli of *B. burgdorferi* (Figure 1), and that molecules other than, or in addition to IL-6, IL-8 and TNF were required to elicit SY cell apoptosis, we further explored the diversity of mediators produced by microglia in response to *B. burgdorferi*. As several investigators had reported that the activated glia seen in many CNS pathologies were able to kill neurons by the release of nitric oxide (NO) into their surrounding environments [46]–[48], and knowing that *B. burgdorferi* could elicit the release of NO from exposed macrophages [49], we explored this possibility in our models. Using an adaptation of the Greiss reaction, we were not able to detect any significant release of NO into the supernatant of microglia, astrocytes, SY cells or any combinations of the three cell types co-cultured with *B. burgdorferi* for either 24 hours or for 5 days (data not shown).

Using a 27 cytokine Bio-Plex assay to expand on our previous data, we found along with IL-6 and IL-8, significant *B. burgdorferi*-induced upregulation in expression of the pro-inflammatory chemokines CCL2, CCL3, CCL4 and CCL5 by microglial cells (Table 2). L-OspA (0.25 µg/ml) was observed to elicit a comparable upregulation of cytokines in both microglia and astrocytes, indicating that *B. burgdorferi* lipoproteins may elicit the lion's share of the spirochetal stimulus. However, when microglia were present in the cultures, alone or with astrocytes (aggregate cultures), it appeared that spirochetes provided, overall, the stronger stimulus. SY cell responses were absent or minimal in comparison to that of the microglia, except for the production of vascular endothelial growth factor (VEGF) (Table 2), which has been increasingly implicated as a contributing factor in neuronal protection and survival [50]–[53].

**Table 2:** Reactive cytokine/chemokine expression in SH-SY5Y (SY) neurons and glia co-cultured for 5 days in the presence of *Bb* or L-OspA.

	IL-6	IL-8	CCL2 MCP-1	*CCL3 MIP-1α	CCL4 MIP-1β	CCL5 RANTES	VEGF
**SY**	**0** (±0)	**0** (±0)	**0.21** (±0.02)	**0** (±0)	**0** (±0)	**0** (±0)	**1.1** (±0.94)
**SY+L-OspA**	**0** (±0)	**0.11** (±0.06)	**1.05** (±0.18)	**0** (±0)	**0** (±0)	**0.14** (±0.17)	**1.6** (±0.56)
**SY+** ***Bb***	**0** (±0)	**0.34** (±0.24)	**0.95** (±0.01)	**0** (±0)	**0** (±0)	**0.24** (±0.05)	**3.4** (±0.65)
**A**	**10.4** (±1.02)	**2.06** (±1.0)	**10.6** (±0.2)	**1.68** (±0.64)	**0.01** (±0)	**0** (±0)	**2.99** (±0.01)
**A+L-OspA**	**19.0** (±4.7)	**12.45** (±1.62)	**47.5** (±11.0)	**3.17** (±1.58)	**0.03** (±0.01)	**0.01** (±0)	**3.4** (±0.6)
**A+** ***Bb***	**15.36** (±1.72)	**5.89** (±1.05)	**18.6** (±6.4)	**8.33** (±1.79)	**0.05** (±0.01)	**0** (±0)	**2.72** (±0.29)
**A/SY**	**3.62** (±0.23)	**0.96** (±0.14)	**6.95** (±1.2)	**2.37** (±0.88)	**0.08** (±0.06)	**0** (±0)	**2.89** (±0.06)
**A/SY+** ***Bb***	**3.36** (±0.39)	**1.29** (±0.24)	**34.4** (±8.8)	**8.07** (±1.32)	**0.03** (±0)	**0** (±0)	**3.22** (±0.4)
**μG**	**6.42** (±2.0)	**5.33** (±0.21)	**56.9** (±15.4)	**7.68** (±1.5)	**4.99** (±0.87)	**0.02** (±0.01)	**1.16** (±0)
**μG+L-OspA**	**19.6** (±0.65)	**29.87** (±3.32)	**257.0** (±28.0)	**32.5** (±8.98)	**14.7** (±1.23)	**0.23** (±0.01)	**1.65** (±0.1)
**μG+** ***Bb***	**22.8** (±0.66)	**51.3** (±0.53)	**272.0** (±30.0)	**48.55** (±21.32)	**18.58** (±0)	**1.73** (±0.02)	**2.04** (±0.09)
**μG/SY**	**1.53** (±0.19)	**5.6** (±2.6)	**49.7** (±11.4)	**7.10** (±1.55)	**4.93** (±0.89)	**0.03** (±0.03)	**0.87** (±0.03)
**μG/SY+** ***Bb***	**9.8** (±0.02)	**41.7** (±0.82)	**158.67** (±4.0)	**25.15** (±4.25)	**8.96** (±0.19)	**0.37** (±	**2.5** (±0.34)
**Agg**	**7.6** (±1.16)	**3.63** (±0.33)	**37.7** (±2.04)	**6.55** (±1.68)	**3.08** (±0.79)	**0.02** (0.01)	**1.74** (±0.26)
**Agg+L-OspA**	**55.17** (±4.01)	**31.5** (±0.63)	**177.0** (±32.1)	**12.15** (±2.79)		**0.12** (±0.03)	**1.84** (±1.67)
**Agg+** ***Bb***	**36.55** (±1.93)	**60.0** (±0.25)	**201.0** (±46.6)	**20.35** (±1.48)	**12.17** (±0)	**1.23** (±0.14)	**2.22** (±0.09)
**Agg/SY**	**2.06** (±0.03)	**2.81** (±0.46)	**65.8** (±1.1)	**2.87** (±0)	**0.08** (±0.06)	**0.02** (±0)	**1.61** (±0.04)
**Agg/SY+** ***Bb***	**15.23** (±0)	**60.2** (±0)	**134.9** (±16.2)	**15.79** (±2.36)		**0.84** (±0)	**1.98** (±0)

Microglia (μG) and aggregate cells (Agg), which contain astrocytes and microglia, produce higher concentrations of the inflammatory mediators assessed in response to *Borrelia burgdorferi (Bb)* as compared to astrocytes (A) or SY neuronal cells. Lipidated-outer surface protein A (L-OspA) was an important stimulant in this reaction. The growth factor VEGF, was expressed in neurons equally or more abundantly than glia in response to *Bb*. Aliquots of glial cell supernatant were all obtained from a single representative animal during an individual experiment. Values listed are in ng/ml except for *CCL3 which is in pg/ml, followed by a standard error of the mean where n = 2.

*B. burgdorferi* induced significant cytokine/chemokine expression in astrocytes, but expression levels were generally orders of magnitude lower than those of microglia that had been derived from the same tissue (Table 2). These results, combined with our previous cytokine and apoptosis assays, prompted us to focus on interactions of microglia with SY cells in the presence of *B. burgdorferi*, and to broaden our exploration of pathways involved in microglial activation and neuronal apoptosis.

### Multiple inflammatory signaling pathways are activated in microglia exposed to *B. burgdorferi*

**Figure 3:**
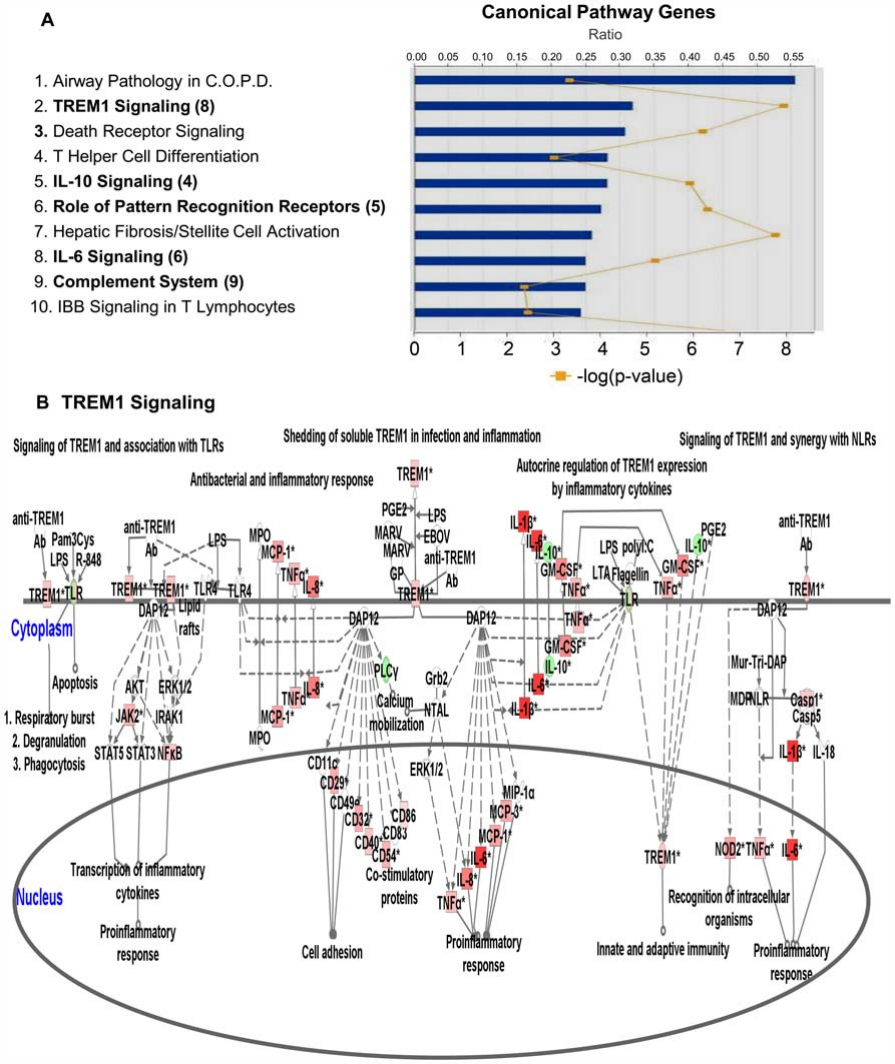
Microarray analysis of gene expression in primary rhesus microglial cells cultured for 5 days in the presence of *B. burgdorferi*, versus microglia cultured in medium alone. (A) Five of the ten canonical pathways most affected during co-culture of microglia with *B. burgdorferi* involved inflammatory signaling (in bold) and occurred in cells isolated from each of two rhesus macaques sampled. The number in parenthesis indicates the pathway order of significance in the 2nd animal. Bar graph represents the ratio of gene expression in microglia co-cultured with *B. burgdorferi* as compared to control microglia cultured in medium alone. Line graph represents significance as –log (p-value) where p<0.05. (B) Microarray analysis of the triggering receptor expressed in myeloid cells (TREM1) pathway indicates, through transcript up-regulation of numerous pro-inflammatory genes including MCP-1 (CCL2), IL-6 and lL-8, the tendency for a sustained microglia-generated inflammatory response to *B. burgdorferi.* Values were obtained using Ingenuity Pathways Analysis (IPA) software, version 6. Minimum fold change was ≥2.0 with significance of p<0.05. All genes in this pathway were represented on the array chips. Red: increased gene expression. Green: decreased gene expression. The results displayed in sections (A) and (B) represent findings from animal number 1. Individual transcript changes for animals 1 and 2 can be accessed through the online supplemental data (Tables S1 and S2).

We used microarray analysis to further investigate how exposure to *B. burgdorferi* might affect global gene expression in microglial cells. As changes in the expression and activity of multiple genes often work in concert to affect responses to many cellular pathogens, including *B. burgdorferi* [10], [54]–[56], we used Ingenuity Pathways Analysis software to compare transcript levels in 18,000 annotated rhesus genes. When microglia were cultured in the presence of *B. burgdorferi*, as compared to medium alone, five of the ten most altered canonical pathways, as well as the chemokine signaling pathway (number 19 on the list of 232 affected pathways), showed significant upregulation in inflammatory signaling (Figure 3A).

Many of the transcript changes that occurred within the triggering receptor expressed on myeloid cells-1 (TREM1), pattern recognition receptors (PRR), IL-10, IL-6 and chemokine signaling (Chem. Sig.) pathways were proinflammatory and they were repeated in several pathways that included both innate and adaptive immune responses (Table 3). Interestingly, the pathway that exhibited the most profound changes in regard to inflammatory signaling was TREM1 (Figure 3B, Table 4).

**Table 3:** Gene transcripts significantly affected by *Borrelia burgdorferi (Bb)* in microglia.

Symbol	Entrez Gene Name	Exp Val Fold Δ	TREM	PRR	IL-6	IL-10	Chem. Sig.
**CASP1**	caspase1 (IL1β convertase)	**↑1.335**	**	**			
**CCL2 (MCP-1)**	chemokine (C-C motif) ligand 2	**↑2.355**	**				**
**CCL4 (Mip-1β)**	chemokine (C-C motif) ligand 4	**↑1.176**					**
**CCL5 (RANTES)**	chemokine (C-C motif) ligand 5	**↑2.766**		**			**
**CCL7 (MCP-3)**	chemokine (C-C motif) ligand 7	**↑2.178**	**				**
**CXCL12**	chemokine (C-C motif) ligand 12 (stromal cell derived factor 1)	**↑1.640**					**
**CXCR4**	chemokine (CxC motif) receptor 4	**↑2.036**					**
**IL-6**	interleukin 6	**↑5.053**	**	**	**	**	
**IL-8**	interleukin 8	**↑3.353**	**		**		
**IL-10**	interleukin 10	**↓1.531**	**	**		**	
**IL-12β**	interleukin 12 beta	**↑5.299**		**			
**IL-1α**	interleukin 1, alpha	**↑5.585**			**	**	
**IL-1β**	interleukin 1, beta	**↑5.640**	**	**	**	**	
**IL-1R2**	interleukin 1 receptor, type II	**↑1.598**			**	**	
**NFκβ1**	nuclear factor of kappa light polypeptide gene enhancer in B-cells 1	**↑1.514**	**	**	**	**	
**NFκβ2**	nuclear factor of kappa light polypeptide gene enhancer in B-cells 2	**↑1.295**	**	**	**	**	
**NOD2**	nucleotide-binding oligomerization domain containing 2	**↑2.258**	**	**			
**TLR2**	toll-like receptor 2	**↑1.641**	**	**			
**TNF**	tumor necrosis factor (member 2)	**↑1.711**	**	**	**	**	

Values were obtained using Ingenuity Pathways Analysis software, version 6. Minimum fold change was set at ≥2 and is shown as ratio, log 2, with p<0.05. All of the listed pathway genes were represented on the array chips. Pathway abbreviations: TREM: Triggering Receptor Expressed on Myeloid Cells; PRR: Role of Pattern Recognition Receptors; Chem. Sig.: Chemokine Signaling.

**Table 4:** Molecules associated with TREM1 signaling in microglia co-cultured in the presence of *B. burgdorferi* for 5 days.

Gene Symbol	Entrez Gene Name	GenBank/RefSeq	Exp Val/Fold Change
**CASP1**	Caspase 1	**NM_001223**	↑1.335
**CCL2**	chemokine ligand 2	**NM_002982**	↑2.335
**CCL7**	chemokine ligand 7	**NM_006273**	↑2.178
**CD40**	CD40 molecule	**NM_001250**	↑1.453
**CD86**	CD86 molecule	**NM_006889**	↑1.000
**CSF2**	colony stimulating factor	**NM_000758**	↑3.064
**FCGR28**	Fc fragment of IgG	**NM_004001**	↑2.200
**ICAM1**	intercellular adhesion molecule 1	**NM_000201**	↑1.964
**IL-6**	interleukin 6	**NM_000600**	↑5.053
**IL-8**	interleukin 8	**NM_000584**	↑3.353
**IL-10**	interleukin 10	**NM_000572**	↓1.531
**IL-18**	interleukin 1 beta	**NM_000576**	↑5.640
**ITGB1**	integrin beta 1	**NM_033667**	↑2.078
**JAk2**	Janus kinase 2	**NM_004972**	↑1.907
**NFκβ1**	nuclear factor of kappa light polypeptide gene enhancer in B-cells 1	**NM_003998**	↑1.514
**NFκβ2**	nuclear factor of kappa light polypeptide gene enhancer in B-cells 2	**NM_002502**	↑1.295
**NOD2**	nucleotide-binding oligomerization domain containing 2	**NM_022162**	↑2.258
**PLCG2**	phospholipase C	**NM_002661**	↓1.500
**TLR2**	toll-like receptor 2	**NM_003264**	↑1.641
**TLR8**	toll-like receptor 8	**NM_138636**	↓1.756
**TNF**	tumor necrosis factor	**NM_000594**	↑1.711
**TREM1**	triggering receptor expressed on myeloid cells	**NM_018643**	↑1.462

TREM1 cell surface receptors associate with the adaptor molecule DAP12 for signaling and function and have been classified as immune/inflammatory response amplifiers. Although the receptor has thus far not been found to be expressed on microglial cells [55], [57]–[61], our array data indicate that it is expressed in rhesus microglia, or a subpopulation thereof, and is also upregulated during infection with *B. burgdorferi*. A low level of TREM1 expression in rhesus microglia was confirmed by RT-PCR (data not shown). RT-PCR was further used to confirm significant transcript up-regulation in the following three selected molecules: CCL2 (TREM1 and Chem. Sig. pathways), IL-6 (TREM1, PRR, IL-10 and IL-6 pathways) and CCL5 (PRR and Chem. Sig. pathways), (Table 5). Individual transcript changes for both animals sampled can be accessed online through the supplemental data portion of this report (Tables S1 and S2).

**Table 5:** Confirmation of rhesus array results using QRT-PCR.

Gene	μG/Bb (fold change)	P-value
CCL2	+4.17	0.001
IL-6	+6.62	0.001
CCL5	+11.07	0.001

Transcript RNA collected from microglia cultured in the presence of *B. burgdorferi (Bb)* was compared to that of RNA collected from microglia (μG) cultured in medium alone. RT-PCR reactions were run in triplicate with GAPDH gene expression used as the reference. PCR efficiencies, average fold change and statistics were evaluated using REST© software.

### *B. burgdorferi*-activated microglia may influence p53 Signaling in SY cells

**Figure 4:**
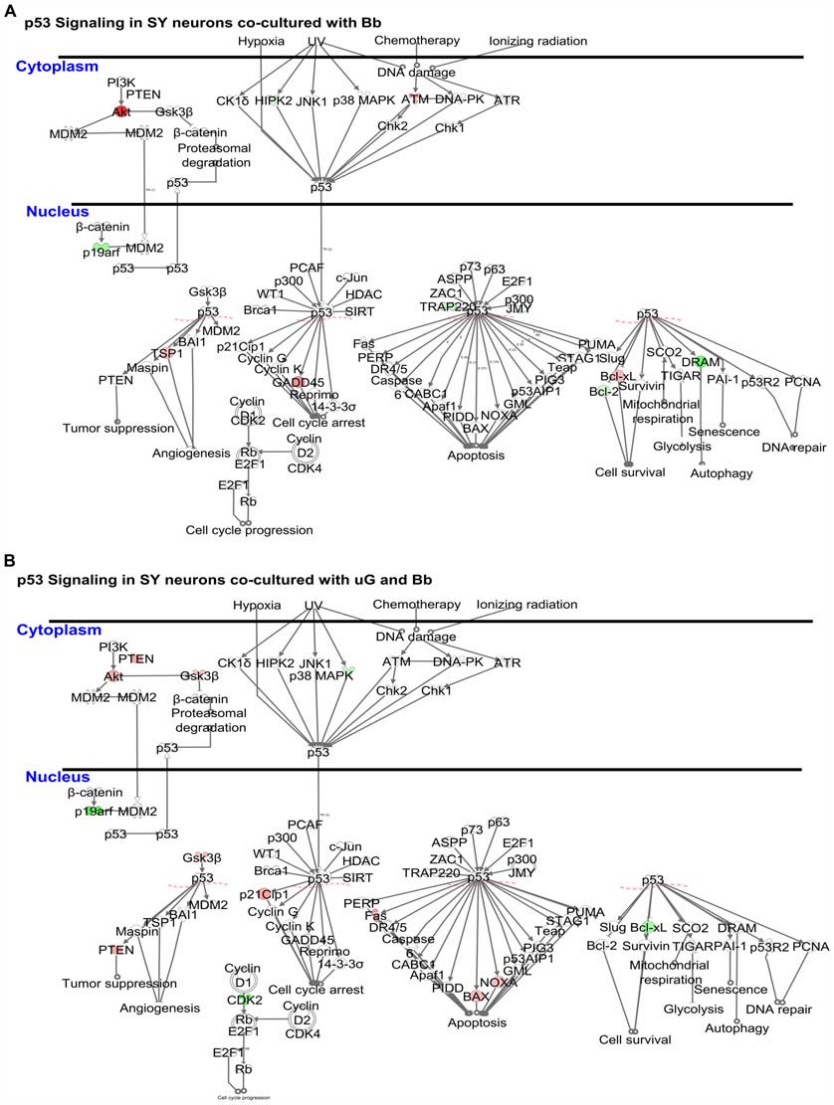
The p53 Signaling Pathway indicates a microglial cell-induced shift in the gene expression of SH-SY5Y (SY) cells co-cultured with *B. burgdorferi* from one of cell survival to one of preparation for apoptosis. (A) Gene expression for p53 signaling in SY cells co-cultured with *B. burgdorferi* exhibits through up-regulation of transcript for protein kinase B (AKT), thrombospondin (TSP)-1 and Bcl2-like (Bcl-X_L_) combined with decreased transcript for damage-regulated autophagy modulator (DRAM) and p16, a mode of cell survival and proliferation. (B) The inclusion of microglial cells in the co-culture of SY cells with *B. burgdorferi* causes a shift in SY gene expression more in line with preparation for apoptosis than cell survival. There is increased transcript expression for pro-apoptotic phosphatase and tensin homolog (PTEN), Bcl-2-associated X protein (BAX) and phorbol-12-myristate-13-acetate-induced protein (NOXA)-1 genes combined with a reversal to decreased transcript for the anti-apoptotic molecule Bcl-xL. There is also increased expression of the cyclin-dependent kinase inhibitor, p21 and down-regulation in cyclin dependent kinase CDK2, both indicating a potential preparation for cell cycle arrest at G1/S. See also, Table 5. Values were obtained using Ingenuity Pathways Analysis (IPA) software version 6. Fold change was ≥1.5 with p≤0.05. The human array chips contained approximately 22,000 unique annotated genes and all genes in these pathways were represented on the chips. Red: increased gene expression. Green: decreased gene expression. Microglia were physically separated from the SY cells by 3.0-micrometer pore diameter transwell culture inserts. Individual transcript can be accessed through the online supplemental data (Tables S3 and Tables S4).

In addition to affecting the environment surrounding neurons exposed to *B. burgdorferi*, activated microglia may also affect gene expression of neurons themselves. Microarray analysis for the p53 Signaling pathway revealed a microglia-induced shift in the gene expression of SY cells co-cultured with *B. burgdorferi* from one of cell survival and proliferation to one more in line with cell cycle arrest and apoptosis (Figure 4A and 4B).

In cultures containing only SY cells and *B. burgdorferi*, there were prominent increases in transcript for the cell survival and anti-apoptotic molecules protein kinase B (AKT1) and BCL2-like (Bcl-X_L_) genes, combined with a decrease in transcript for pro-apoptotic damage-regulated autophagy modulator (DRAM) (Table 6-left, Figure 4A).

**Table 6:** p53 Signaling in SH-SY5Y (SY) Cells.

SY cells co-cultured with *Bb*			SY cells co-cultured with microglia and *Bb*		
Symbol	Entrez Gene Name	Exp Val Fold Δ	Symbol	Entrez Gene Name	Exp Val Fold Δ
**AKT1**	v-akt murine thymoma viral oncogene homolog 1	**↑2.550**	**AKT1**	v-akt murine thymoma viral oncogene homolog 1	**↑2.132**
**ATM**	ataxia telangiectasia mutated	**↑0.908**	**AKT3**	v-akt murine thymoma viral oncogene homolog 3	**↑0.868**
**BCL2**	B-cell CLL/lymphoma 2	**↓0.649**	*BAX	BCL2-associated X protein	**↑0.826**
**BCL2L1 (Bcl-X_L_)**	BCL2-like 1	**↑0.703**	*BCL2L1 (Bcl-X_L_)	BCL2-like 1	**↓1.267**
**CDKN2A (p16)**	cyclin-dependent kinase inhibitor 2A	**↓1.554**	**CDKN2A (p16)**	cyclin-dependent kinase inhibitor 2A	**↓2.397**
**DRAM**	damage-regulated autophagy modulator	**↓2.340**	**CDK2	cyclin-dependent kinase 2	**↓2.091**
**GADD45B**	growth arrest and DNA-damage-inducible, beta	**↑1.089**	**CDKN1A (p21)	cyclin-dependent kinase inhibitor 1A	**↑1.072**
**HIPK2**	Homeodomain interacting protein kinase 2	**↓0.707**	**FASN**	fatty acid synthase	**↑1.189**
**MED1**	mediator complex subunit 1	**↓0.848**	**GSK3B	glycogen synthase kinase 3 beta	**↑0.717**
**THBS1**	thrombospondin 1	**↑0.624**	**MAPK14**	mitogen activated protein kinase 14	**↓0.788**
			*PMAIP1 (NOXA-1)	phorbol-12-myristate-13-acetate-induced protein 1	**↑0.750**
			*PTEN	phosphatase and tensin homolog	**↑1.027**

Changes in p53 Signaling gene expression indicate a microglial cell-induced shift in the state of SY cells co-cultured with *B. burgdorferi (Bb)* from one of cell survival and proliferation, toward one of apoptosis and cell cycle arrest. Changes noted with a single asterisk represent a pro-apoptotic shift while those shown with a double asterisk suggest preparation for cell cycle arrest at G1/S.

This scenario, however, changed dramatically in experiments when microglia were included in the co-culture of SY cells with *B. burgdorferi* (Figure 4B, Table 6, right). In order to study microglia-induced gene expression changes in the SY cells, transwell inserts were incorporated into the culture system providing physical separation between the SY cells and the microglial cells, yet allowing for bidirectional transfer of secreted molecules between the cell types. In this model, gene expression for phosphatase and tensin homolog (PTEN), which functions to modulate cell survival and proliferation primarily through its downstream effects on AKT1, was shown to be significantly increased (Table 6, right). SY cell anti-apoptotic Bcl-X_L_ expression was observed to completely swing from up to down-regulation in the presence of microglia, while pro-apoptotic BCL2-associated X protein (Bax) and phorbol-12-myristate-13-acetate-induced protein 1 (NOXA-1) transcripts were increased. Additionally, the transcript for cyclin-dependent kinase 2 (CDK2), which typically drives the cell cycle, was down-regulated, and the G1/S cell cycle checkpoint inhibitors, cyclin-dependent kinase inhibitor 1A (p21) and glycogen synthase kinase 3 beta (GSK3β), were both increased (Figure 4, Table 6 right). The observed changes for individual gene transcript levels may be accessed online in the supplemental data portion of this report (Tables S3 and S4). RT-PCR was used to confirm transcript changes in selected molecules from co-cultures of the SY neurons with *B. burgdorferi* both in the presence and absence of microglia (Table 7).

**Table 7:** Confirmation of SY array results using QRT-PCR.

Gene	SY/*Bb*(fold change)	P-value
ATM	+2.02	0.001
DRAM	−1.56	0.001
	**SY/μG/** ***Bb*** **(fold change)**	
PTEN	+1.81	0.05
BAX	+1.40	0.001
Bcl-X_L_	−2.83	0.001

RT-PCR reactions were run in triplicate with GAPDH gene expression used as the reference. PCR efficiencies, average fold change and statistics were evaluated using REST© software. SY: SHSY5Y “n” type neuroblastoma cells; *Bb: B. burgdorferi*; μG: microglia.

### The induction of neuronal apoptosis during co-culture with *B. burgdorferi* and microglia might require cell-cell contact between the SY cells and microglia

Several investigators have shown that when in the presence of certain stimuli, not only can macrophages/microglia induce apoptosis in neighboring cells through a bystander effect, but that there might be an additional requirement for cell-cell contact between the macrophages/microglia and the affected nearby cells [62]–[64].

**Figure 5:**
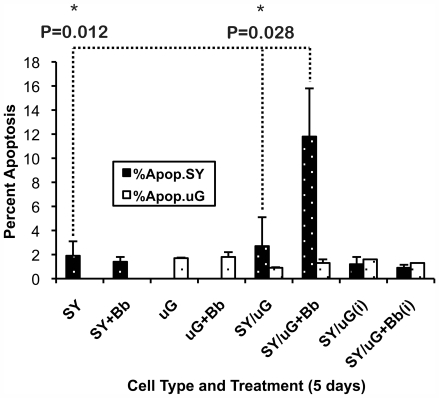
Induction of neuronal apoptosis during co-culture with *B. burgdorferi* and microglia may be influenced by or even require cell-cell contact between neurons and microglia. The TUNEL assay was used to evaluate apoptosis in SH-SY5Y (SY) neurons and microglia (μG) co-cultured with *B. burgdorferi (Bb)* for 5 days. An average 7.7 fold-increase in neuronal apoptosis was observed when SY cells were co-cultured with primary microglia and *B. burgdorferi* as compared to the SY cells cultured in medium alone. An increase in neuronal cell apoptosis was also observed during the co-culture of SY cells with primary microglia when *B. burgdorferi* was present. No statistical changes in apoptosis were noted between the SY cells cultured in medium alone and those co-cultured with either *B. burgdorferi* alone, with microglia or co-cultured with microglia and *B. burgdorferi* using 3.0-micrometer filter transwell inserts to physically separate the cells (i). Values represent the mean (n = 3) ± S.D. P values were obtained using One Way analysis of variance (ANOVA) followed by Bonferroni, Tukey and Levine's tests. Microglial cells in each experiment were all obtained from the same animal, but different animals were used as a source of cells for each of the three experiments.

To address the question of whether the microglia-secreted mediators in our system were enough to stimulate SY cell apoptosis on their own, we again incorporated transwell inserts into our co-culture models to physically separate the microglia from the SY cells while at the same time, maintaining a generally homogeneous exposure of each cell type to the culture medium. Parallel assays for *B. burgdorferi*-induced apoptosis were set up where one model followed the same experimental design that was described in Figure 2, and the second model included the addition of transwell inserts into the co-culture system for the duration of the experiment. In each case, primary microglia or aggregate cell isolates from three separate non-human primates were combined in co-culture for 5 days with SY neurons and *B. burgdorferi*. Using the original co-culture format, we found an average 7.7-fold increase in neuronal apoptosis when SY cells were co-cultured with microglia and *B. burgdorferi* as compared to the SY cells cultured in medium alone. In contrast, no significant changes in apoptosis levels were observed when the SY cells were co-cultured with *B. burgdorferi*, with microglia, or with microglia and *B. burgdorferi* using the transwell inserts (Figure 5).

## Discussion

Neuroinflammation is thought to be a contributing factor in a number of neurodegenerative disorders including Alzheimer's disease, Parkinson's disease and multiple sclerosis, as well as in Lyme neuroborreliosis [6],[7],[31],[65]. Inflammatory mediator levels are often elevated in these disorders suggesting that they are actively involved in the disease process [2],[6],[31],[66]. Because neurological symptoms do occur in many patients with Lyme disease, and cognitive impairment is often a part of this scenario, it was important to discover which mediators likely caused the effects of *B. burgdorferi-*induced damage in neurons. We hypothesized that the inflammatory environment generated during a possible *in vivo* exposure of glial cells to *B. burgdorferi* could harm neurons through a bystander effect. To address this hypothesis, we used *in vitro* experiments to demonstrate that with regard to microglia, astrocytes, and neurons, the fundamental triumvirate of cells in the CNS, it was the microglia that most aggressively responded to interaction with *B. burgdorferi*. In addition to inducing a pronounced and sustained production of cytokines and chemokines in microglial cells, *B. burgdorferi* also activated important inflammatory signaling pathways in these cells. Together, these responses potentially contributed to creating a reactive environment that was toxic to the SY cells. Interestingly, we also found that in addition to inducing SY cell apoptosis through a bystander effect, there might be a requirement for direct cell-cell contact between microglia and neurons for end-stage damage to occur.

In early experiments, we determined that when SY cells were co-cultured in the presence of microglia and *B. burgdorferi* for at least 5 days (MOI 10∶1), significant increases in SY, but not glial cell apoptosis occurred. Our experiments also ruled out any *B. burgdorferi* related upregulation in the production of nitric oxide. While previous reports had indicated that L-OspA would induce significant levels of apoptosis in primary rhesus astrocytes [67], the L-OspA concentration of 1 µg/ml used in those experiments was 4 times greater than the one used in the current experiments. Using a formula developed by Norgard, et al. [68] we approximated the total amount of outer surface lipoproteins correlating to the number of spirochetes used in our MOI. An L-OspA concentration of 0.25 µg/ml provided a quantitatively more realistic representation of lipoproteins present in *B. burgdorferi* at the MOI used in our studies. We further discovered that although *B. burgdorferi* did induce a potent expression and secretion of the inflammatory cytokines/chemokines IL-6, IL-8 and TNF in microglia, additional mediators were required to trigger neuronal cell apoptosis. These results prompted us to broaden our study of potential inflammatory mediators, and at the same time, to explore *B. burgdorferi*-activated pathways in microglia and SY cells that might be further contributing to the neurotoxicity demonstrated in the apoptosis assays.

By expanding our search for the expression of cytokines and chemokines that were potentially relevant to *B. burgdorferi-*induced neurotoxicity we found that in addition to IL-6, IL-8 and TNF, the four well known proinflammatory chemokines CCL2, CCL3, CCL4 and CCL5 were significantly upregulated in microglial cells co-cultured with *B. burgdorferi*. Upregulation of CC chemokines has been described both in murine models of neurocysticerosis and multiple sclerosis (experimental autoimmune encephalomyelitis, EAE) [69],[70]. CCL2 in particular has been shown to play a role in the pathogenesis of EAE [71]. Perhaps most interesting were the microarray results showing that each of these molecules, with the exception of CCL3, was represented in more than one of the microglial signaling pathways that were most affected by *B. burgdorferi*. Considering the high number of inflammatory genes involved in these pathways and their potential for cross-talk and downstream regulation, we believe that the TREM1 pathway, together with those involving PRR, IL-10, IL-6 and Chem. Sig., contribute to *B. burgdorferi*-induced inflammation in the CNS.

Even though some of the remaining affected pathways may have provided checks and balances to our findings, many of the transcript changes that occurred in the pathways that we focused on were proinflammatory and included both innate and adaptive immune response molecules. As such, there seems to be a consistent pattern of inflammatory signaling in microglial cells that is directly associated with the presence of *B. burgdorferi*, and that might adversely affect nearby neuronal cells.

When glial cells were co-cultured with *B. burgdorferi* in the presence of SY cells the concentration of inflammatory mediators was often reduced as compared to that elicited in the absence of SY cells (Table 2). Since we saw no evidence of glial cell apoptosis, this decrease is most likely due to the reduced number of glial cells, the main producers of pro-inflammatory mediators that were included in these cultures. There are reports that apoptotic neurons co-cultured with microglia may down-regulate microglial synthesis of pro-inflammatory molecules [32]. Such a mechanism also may have contributed to the observed reduction, at least in the case of microglia/SY cell co-cultures.

Using microarray analysis, we also showed that *B. burgdorferi-*activated microglia could invoke changes in their cellular environment that affected gene expression in proximal SY cells. When SY cells were co-cultured with *B. burgdorferi* alone, SY gene expression for molecules in the p53 signaling pathway indicated a mode of cell survival and proliferation. This conclusion was founded on the observed upregulation in transcripts for AKT1 and Bcl-X_L_, paired with decreased gene expression for DRAM. AKT1, which is activated through the phosphatidylinositol 3-kinase (PI3K) pathway, is known to stimulate cell cycle progression (via p21 phosphorylation and release from CDK2) and to play an important role in promoting cell survival through the suppression of apoptosis [72]-[74]. Bcl-X_L_ is likewise a strong promoter of cell survival, but as one of the major anti-apoptotic members of the conserved Bcl-2 family of proteins, it functions to inhibit programmed cell death through the control of mitochondrial membrane permeabilization [75]–[78]. DRAM on the other hand, is a lysosomal protein critical for the ability of p53 to induce either autophagy or programmed cell death [79]–[81].

In parallel experiments where microglia were included in the co-culture of SY cells with *B. burgdorferi*, but physically separated from the SY cells by transwell inserts, we found a striking change of circumstance. One primary difference observed with the inclusion of microglial cells was increased SY cell gene expression of the tumor suppressor PTEN. This molecule acts as a dual protein and lipid phosphatase that can modulate cell survival, proliferation and apoptosis through its downstream effects on phosphatidylinositol 3-kinase (PI3K) and the AKT1 gene [72],[82],[83]. Complementing the PTEN regulation of AKT1 was a decrease in transcript for CDK2 combined with transcript increases for the CDK inhibitors p21 and GSK3β. Within the cell cycle machinery, cyclin-dependent kinases (CDKs) associate with their respective cyclins to drive a cell through the G1/S checkpoint. CDK2 associates with cyclin E and during events of cell stress or DNA damage, p21 can bind the CDK2/cyclinE complex and induce cell cycle arrest at G1/S. Furthermore, association of the CDK4 with cyclin D1 can also be disrupted by the action of GSK3β, which phosphorylates cyclin D1, triggering its nuclear export and degradation [84]–[86]. Both of these events, if realized, would indicate a drift toward cell cycle arrest, which was not apparent in the transcript changes for the culture of SY cells with *B. burgdorferi*, but lacking microglia.

Other significant changes in gene expression were found in members of the apoptotic Bcl-2 family. Transcript of the anti-apoptotic Bcl-X_L_ molecule, whose function was described previously, changed from being up-regulated when microglia were not present to being markedly down-regulated in the neuron/microglia/*B. burgdorferi* co-cultures. At the same time, expression of the pro-apoptotic Bax and NOXA-1 transcripts was found to be increased. Under normal conditions, Bax is constitutively associated with ly-X_L_. During cellular stress, Bax can be activated by a BH3-only protein such as Bid, Bim or NOXA-1. It may also be displaced by Bad or Bik molecules which possess Bad-like BH3 domains, but act directly on Bcl-2 or Bcl-X_L_ rather than Bax itself, freeing it from Bcl-X_L_ to translocate from the cytosol and directly bind to the mitochondrial membrane, initiating an apoptotic cascade [75], [76], [87]–[89]. AKT1 kinase is responsible for the phosphorylation of Bad, keeping it tied to its support molecule 14-3-3. In an inactivated state, such as that induced by the up-regulation of PTEN, Bad may become unphosphorylated, disassociate from 14-3-3 and move to displace Bax [75],[88]. While the results of *in vitro* experiments may not always mirror *in vivo* responses, this compilation of neuronal transcript changes in the p53 signaling pathway appears to be predicated on the presence of microglia, and provides a compelling example of the neurotoxic effects that activated microglia may have on neurons in their surroundings.

Finally, although we have presented evidence of the ability of microglia to harm SY cells through a bystander effect, our findings indicate that microglia-SY cell proximity, while necessary, may not be sufficient for end-stage SY cell damage/apoptosis to occur. When transwell filter inserts were incorporated into our apoptosis experiments, no significant increases in SY cell death were observed regardless of whether microglia were included in the co-cultures of SY cells with *B. burgdorferi* or not. This differed significantly from a near 8-fold increase in SY cell apoptosis that occurred when microglia (from the same animal) and SY cells were in direct physical contact during their co-culture with *B. burgdorferi*, indicating that neuronal-microglia contact was required for apoptosis to occur. Several investigators have reported a similar cell-cell contact requirement for apoptosis induced by activated macrophages and other non-neuronal cells [62]–[64],[90]. Whether their contact with mediators secreted by *B. burgdorferi-*activated microglia is itself enough to critically damage SY cells, or it simply acts to prime the cells for eventual apoptosis, remains in question. Due to viability constraints of the cell culture medium, we were not able to extend our apoptosis assays beyond 5 days. We therefore cannot exclude the possibility that longer exposure to microglial mediators might have induced apoptosis in SY cells. If this were the case, the cell-cell contact hypothesis would shift from one of “requirement” to one of “influence”. Further, even though steps were taken to more normalize the SY cell-line phenotype, these cells may respond differently than would non-transformed neurons. While experiments with primary neurons would be optimal, they are often not practical. Similar experiments with other cell lines and even primary neurons may be critical to understanding how *B. burgdorferi* affects neurons of the CNS during neuroborreliosis.

## Supporting Information

Table S1 Microglia + *Borrelia burgdorferi* vs. Microglia alone, animal 1
(0.45 MB PDF)

Table S2 Microglia + *Borrelia burgdorferi* vs. Microglia alone, animal 2
(0.23 MB PDF)

Table S3 SY3D + *B. burgdorferi* vs. SY3D alone
(0.28 MB PDF)

Table S4 SY3D + *B. burgdorferi* + Microglia vs. SY3D alone
(0.83 MB PDF)
