# Supplementary material for: Microglia Are Mediators of Borrelia burgdorferi–Induced Apoptosis in SH-SY5Y Neuronal Cells
Source: PLoS Pathog. 2009 Nov 13;5(11):e1000659. doi: 10.1371/journal.ppat.1000659 (PMC2771360; doi:10.1371/journal.ppat.1000659)
Supplement: Table S1 — Microglia + Borrelia burgdorferi vs. Microglia alone, animal 1 (0.45 MB PDF) [file ppat.1000659.s001.pdf]

Table S1: Microglia + Borrelia burgdorferi vs. Microglia alone, animal 1

GeneName	Description	Average Log2 Normalized Fold-change	Standard Deviation
NM_001432	epiregulin (EREG),	6.2189643	2.47985833
NM_007115	tumor necrosis factor, alpha-induced protein 6 (TNFAIP6),	6.07264961	0.67473041
NM_181755	hydroxysteroid (11-beta) dehydrogenase 1 (HSD11B1), transcript variant 2,	5.82753125	1.47753007
NM_002425	matrix metalloproteinase 10 (stromelysin 2) (MMP10),	5.79039799	0.38923012
NM_007115	tumor necrosis factor, alpha-induced protein 6 (TNFAIP6),	5.77659882	0.09169986
NM_002164	indoleamine-pyrrole 2,3 dioxygenase (INDO),	5.6997377	0.44010202
NM_002164	indoleamine-pyrrole 2,3 dioxygenase (INDO),	5.69485005	0.3355518
NM_000576	interleukin 1, beta (IL1B),	5.63971485	0.84618105
NM_000576	interleukin 1, beta (IL1B),	5.58587122	0.64490653
NM_000575	interleukin 1, alpha (IL1A),	5.58509802	1.13338751
NM_002425	matrix metalloproteinase 10 (stromelysin 2) (MMP10),	5.3386994	0.06390854
NM_002187	interleukin 12B (natural killer cell stimulatory factor 2, cytotoxic lymphocyte maturation factor 2, p40) (IL12B),	5.29860926	1.89597667
NM_002422	matrix metalloproteinase 3 (stromelysin 1, progelatinase) (MMP3),	5.26377226	0.5871791
NM_181755	hydroxysteroid (11-beta) dehydrogenase 1 (HSD11B1), transcript variant 2,	5.25981835	0.89409023
NM_002782	pregnancy specific beta-1-glycoprotein 6 (PSG6),	5.23114278	0.54337606
NM_000596	insulin-like growth factor binding protein 1 (IGFBP1),	5.22788771	0.20071633
NM_006290	tumor necrosis factor, alpha-induced protein 3 (TNFAIP3),	5.21856212	0.31351826
NM_000596	insulin-like growth factor binding protein 1 (IGFBP1),	5.20511864	0.18976711
NM_002422	matrix metalloproteinase 3 (stromelysin 1, progelatinase) (MMP3),	5.18929799	0.59422618
NM_005204	mitogen-activated protein kinase kinase kinase 8 (MAP3K8),	5.171801	4.27981558
NM_000575	interleukin 1, alpha (IL1A),	5.15244396	0.75958992
CK232222	TFPI2	5.15122032	0.02772375
NM_052934	solute carrier family 26, member 9 (SLC26A9), transcript variant 1,	5.11192043	3.34456058
NM_002421	matrix metalloproteinase 1 (interstitial collagenase) (MMP1),	5.08797847	0.21452771
NM_000600	interleukin 6 (interferon, beta 2) (IL6),	5.05339937	0.30027065
CK232222	TFPI2	5.01921936	0.05749874
CK232222	TFPI2	5.00219703	0.25223092
CK232222	TFPI2	4.98624553	0.01511038
NM_002421	matrix metalloproteinase 1 (interstitial collagenase) (MMP1),	4.98039976	0.50000621
NM_001305	claudin 4 (CLDN4),	4.96771829	2.53505931
NM_006290	tumor necrosis factor, alpha-induced protein 3 (TNFAIP3),	4.94535429	0.00957012
NM_000600	interleukin 6 (interferon, beta 2) (IL6),	4.9328358	0.24534095
NM_182597	hypothetical protein FLJ39575 (FLJ39575),	4.85384021	1.76848159
CO644910	NMES1	4.74555644	0.6908729
NM_002993	chemokine (C-X-C motif) ligand 6 (granulocyte chemotactic protein 2) (CXCL6),	4.72478425	0.2615077
CO644910	NMES1	4.59731688	0.59250971
NM_001511	chemokine (C-X-C motif) ligand 1 (melanoma growth stimulating activity, alpha) (CXCL1),	4.56701447	0.38052105
NM_001511	chemokine (C-X-C motif) ligand 1 (melanoma growth stimulating activity, alpha) (CXCL1),	4.53621412	0.13199985
NM_003855	interleukin 18 receptor 1 (IL18R1),	4.53464531	0.35443881
NM_016584	interleukin 23, alpha subunit p19 (IL23A),	4.51973148	0.66728356
NM_005651	tryptophan 2,3-dioxygenase (TDO2),	4.41968514	0.6741336

CK231513	PSG5	4.40304214	0.08958886
NM_000623	bradykinin receptor B2 (BDKRB2),	4.40086206	3.30909899
NM_021101	claudin 1 (CLDN1),	4.38801326	0.45205438
XR_013663	Macaca mulatta Putative lymphocyte G0 [XR_013663]	4.37162217	0.43942071
XR_013663	Macaca mulatta Putative lymphocyte G0 [XR_013663]	4.36591254	0.65404827
NM_000636	superoxide dismutase 2, mitochondrial (SOD2), nuclear gene encoding mitochondrial protein, transcript variant 1,	4.35563444	0.52590158
NM_001165	baculoviral IAP repeat-containing 3 (BIRC3), transcript variant 1,	4.34707058	0.07830487
NM_018370	hypothetical protein FLJ11259 (FLJ11259),	4.30983776	0.22821466
NM_003856	interleukin 1 receptor-like 1 (IL1RL1), transcript variant 2,	4.3035947	0.0789442
NM_022154	solute carrier family 39 (zinc transporter), member 8 (SLC39A8),	4.29157857	0.14070887
NM_001009181	statherin (STATH), transcript variant 2,	4.27133051	0.53517189
NM_002993	chemokine (C-X-C motif) ligand 6 (granulocyte chemotactic protein 2) (CXCL6),	4.2530016	0.0698527
NM_002782	pregnancy specific beta-1-glycoprotein 6 (PSG6),	4.25210734	0.2918965
NM_000963	prostaglandin-endoperoxide synthase 2 (prostaglandin G/H synthase and cyclooxygenase) (PTGS2),	4.23767021	0.24454826
NM_001165	baculoviral IAP repeat-containing 3 (BIRC3), transcript variant 1,	4.22358973	0.39652808
NM_002090	chemokine (C-X-C motif) ligand 3 (CXCL3),	4.22316888	0.1809484
NM_014382	ATPase, Ca++ transporting, type 2C, member 1 (ATP2C1), transcript variant 1,	4.22158086	1.13021428
NM_018370	hypothetical protein FLJ11259 (FLJ11259),	4.21525362	0.26514734
NM_022154	solute carrier family 39 (zinc transporter), member 8 (SLC39A8),	4.18562484	0.0003563
NM_152327	adenylate kinase 7 (AK7),	4.18071991	0.78665562
NM_003701	tumor necrosis factor (ligand) superfamily, member 11 (TNFSF11), transcript variant 1,	4.15817207	3.78852906
NM_002090	chemokine (C-X-C motif) ligand 3 (CXCL3),	4.15729441	0.06418838
NM_021016	pregnancy specific beta-1-glycoprotein 3 (PSG3),	4.12720574	1.08536205
NM_002620	platelet factor 4 variant 1 (PF4V1),	4.12437862	0.38521515
NM_021101	claudin 1 (CLDN1),	4.09530259	0.28628069
NM_002620	platelet factor 4 variant 1 (PF4V1),	4.09165784	0.55819311
NM_005949	metallothionein 1F (functional) (MT1F),	4.04581868	0.57228093
NM_006211	proenkephalin (PENK),	4.03674304	3.66070475
NM_002785	pregnancy specific beta-1-glycoprotein 11 (PSG11), transcript variant 1,	4.00105737	0.15417399
NM_153361	hypothetical protein MGC42105 (MGC42105),	3.99710145	3.44069733
NM_001037332	cytoplasmic FMR1 interacting protein 2 (CYFIP2), transcript variant 2,	3.96730558	3.8228976
NM_003155	stanniocalcin 1 (STC1),	3.96245583	0.20136327
NM_001432	epiregulin (EREG),	3.91354673	0.22276687
NM_003155	stanniocalcin 1 (STC1),	3.89398223	0.5787974
NM_002781	pregnancy specific beta-1-glycoprotein 5 (PSG5),	3.88642155	0.4765103
NM_005949	metallothionein 1F (functional) (MT1F),	3.87922169	0.14567132
NM_006398	ubiquitin D (UBD),	3.85279097	0.56669894
CK231513	PSG5	3.84788306	0.25000514
NM_000096	ceruloplasmin (ferroxidase) (CP),	3.84595915	1.02180192
CK231427	ILLUMIGEN_MCQ_2215 Katze_MMLG Macaca mulatta cDNA 5',	3.82452956	0.23430325
NM_006528	tissue factor pathway inhibitor 2 (TFPI2),	3.81885812	0.76468172
NM_021016	pregnancy specific beta-1-glycoprotein 3 (PSG3),	3.80675119	0.78152429
NM_016584	interleukin 23, alpha subunit p19 (IL23A),	3.80218493	0.13167581
NM_003855	interleukin 18 receptor 1 (IL18R1),	3.7999326	0.09368316
NM_002785	pregnancy specific beta-1-glycoprotein 11 (PSG11), transcript variant 1,	3.79706601	0.2107762

NM_000963	prostaglandin-endoperoxide synthase 2 (prostaglandin G/H synthase and cyclooxygenase) (PTGS2),	3.79490658	0.03448729
CN802479	NEK7	3.76287917	1.36889903
CK231427	ILLUMIGEN_MCQ_2215 Katze_MMLG Macaca mulatta cDNA 5',	3.75794129	0.01615497
XM_292225	Ribonuclease pancreatic precursor (RNase 1) (RNase A) (RL1) (LOC338879),	3.74840544	0.98035451
CN801994	MT1X	3.7447372	0.50970125
NM_002704	pro-platelet basic protein (chemokine (C-X-C motif) ligand 7) (PPBP),	3.74129148	0.36644584
NM_000636	superoxide dismutase 2, mitochondrial (SOD2), nuclear gene encoding mitochondrial protein, transcript variant 1,	3.72591109	0.60883452
CO647386	CXCL2	3.72188723	0.19459571
NM_021165	family with sequence similarity 5, member B (FAM5B),	3.71490984	0.61522852
NM_001218	carbonic anhydrase XII (CA12), transcript variant 1,	3.70668899	0.51919524
NM_016568	relaxin 3 receptor 1 (RLN3R1),	3.6970644	3.10247008
XM_375224	cervical cancer suppressor-1 (LOC400410),	3.67982833	0.08448694
CO647386	CXCL2	3.67680809	0.17878897
NM_004591	chemokine (C-C motif) ligand 20 (CCL20),	3.66184148	0.13486559
AY635466	Macaca mulatta cytochrome P450 CYP3A66 (CYP3A66)	3.66181046	0.37077326
XM_375224	cervical cancer suppressor-1 (LOC400410),	3.65141533	0.49339052
NM_000331	serum amyloid A1 (SAA1), transcript variant 1,	3.63576132	0.49113607
NM_006033	lipase, endothelial (LIPG),	3.62294801	0.84027887
NM_002704	pro-platelet basic protein (chemokine (C-X-C motif) ligand 7) (PPBP),	3.60840025	0.83781567
NM_001218	carbonic anhydrase XII (CA12), transcript variant 1,	3.57816016	0.25014908
XM_292225	Ribonuclease pancreatic precursor (RNase 1) (RNase A) (RL1) (LOC338879),	3.5631495	0.19496865
CO580929	SAA2	3.55771986	0.9218401
NM_207335	kelch domain containing 6 (KLHDC6),	3.52758087	1.2322644
NM_080757	chromosome 20 open reading frame 127 (C20orf127),	3.52343489	0.34214123
CK230409	GLRX	3.50552951	0.20438952
CN801994	MT1X	3.47882652	0.58510102
CO580929	SAA2	3.47771101	0.38264924
CK230409	GLRX	3.46932854	0.21458711
NM_005947	metallothionein 1B (functional) (MT1B),	3.46909855	0.05841252
NM_080757	chromosome 20 open reading frame 127 (C20orf127),	3.45804323	0.35587098
NM_000161	GTP cyclohydrolase 1 (dopa-responsive dystonia) (GCH1),	3.4508291	0.31269012
NM_000331	serum amyloid A1 (SAA1), transcript variant 1,	3.44758704	0.31768711
NM_002784	pregnancy specific beta-1-glycoprotein 9 (PSG9),	3.44274665	0.48246211
AY635466	Macaca mulatta cytochrome P450 CYP3A66 (CYP3A66)	3.43730235	0.18519763
NM_002053	guanylate binding protein 1, interferon-inducible, 67kDa (GBP1),	3.42821183	0.079564
NM_006038	spermatogenesis associated 2 (SPATA2),	3.40692245	2.27523472
NM_001657	amphiregulin (schwannoma-derived growth factor) (AREG),	3.3919879	1.14419415
NM_006528	tissue factor pathway inhibitor 2 (TFPI2),	3.37382904	0.54482637
NM_175622	metallothionein 1J (MT1J),	3.37174705	0.13771458
NM_000584	interleukin 8 (IL8),	3.35263935	0.33553969
NM_178859	organic solute transporter beta (OSTbeta),	3.34549531	0.93206359
NM_005950	metallothionein 1G (MT1G),	3.32463982	0.35551512
NM_019609	carboxypeptidase X (M14 family) (CPXM),	3.31907496	0.40915993
NM_000096	ceruloplasmin (ferroxidase) (CP),	3.30640557	0.18627264
NM_006905	pregnancy specific beta-1-glycoprotein 1 (PSG1),	3.29120654	0.30335938

NM_004000	chitinase 3-like 2 (CHI3L2),	3.27863992	0.10914008
NM_003392	wingless-type MMTV integration site family, member 5A (WNT5A),	3.27653289	0.05749562
NM_175622	metallothionein 1J (MT1J),	3.27091354	0.38499492
NM_003392	wingless-type MMTV integration site family, member 5A (WNT5A),	3.26526891	0.10842513
NM_032865	C-terminal tensin-like (CTEN),	3.26450491	0.21803341
NM_001902	cystathionase (cystathionine gamma-lyase) (CTH), transcript variant 1,	3.26279475	2.02999794
NM_032935	metallothionein IV (MT4),	3.25519525	0.66845026
NM_006905	pregnancy specific beta-1-glycoprotein 1 (PSG1),	3.25467994	0.29114412
NM_000641	interleukin 11 (IL11),	3.2543507	0.25274153
NM_006216	serine (or cysteine) proteinase inhibitor, clade E (nexin, plasminogen activator inhibitor type 1), member 2 (SERPINE2),	3.24725283	0.0438452
NM_002583	PRKC, apoptosis, WT1, regulator (PAWR),	3.24415015	2.17783247
NM_000351	steroid sulfatase (microsomal), arylsulfatase C, isozyme S (STS),	3.23897538	0.24452724
NM_006186	nuclear receptor subfamily 4, group A, member 2 (NR4A2), transcript variant 1,	3.22812219	0.11960396
NM_000266	Norrie disease (pseudoglioma) (NDP),	3.21814817	0.59684967
DR771828	SLC7A2	3.20498287	0.25348774
NM_004942	defensin, beta 4 (DEFB4),	3.19294207	0.43638707
NM_006186	nuclear receptor subfamily 4, group A, member 2 (NR4A2), transcript variant 1,	3.19142479	0.39729696
NM_001124	adrenomedullin (ADM),	3.16944878	0.05894013
NM_175617	metallothionein 1E (functional) (MT1E),	3.15650494	1.03443097
NM_000641	interleukin 11 (IL11),	3.15517547	0.37607773
NM_021016	pregnancy specific beta-1-glycoprotein 3 (PSG3),	3.14979959	0.70074952
NM_003937	kynureninase (L-kynurenine hydrolase) (KYNU),	3.14190619	0.07845711
NM_000577	interleukin 1 receptor antagonist (IL1RN), transcript variant 3,	3.13949437	0.15533843
AF303085	Macaca mulatta epididymal-specific lipocalin LCN6	3.1247637	1.94531257
NM_003937	kynureninase (L-kynurenine hydrolase) (KYNU),	3.12101408	0.19778154
NM_002575	serine (or cysteine) proteinase inhibitor, clade B (ovalbumin), member 2 (SERPINB2),	3.11650853	2.57683243
NM_207517	ADAMTS-like 3 (ADAMTSL3),	3.11290668	0.29792573
NM_004613	transglutaminase 2 (C polypeptide, protein-glutamine-gamma-glutamyltransferase) (TGM2), transcript variant 1,	3.11216434	0.11951994
NM_000617	solute carrier family 11 (proton-coupled divalent metal ion transporters), member 2 (SLC11A2),	3.11214694	0.04291848
NM_006398	ubiquitin D (UBD),	3.1115055	0.67178912
NM_002852	pentaxin-related gene, rapidly induced by IL-1 beta (PTX3),	3.11107404	0.38443033
NM_001124	adrenomedullin (ADM),	3.10774658	0.04264521
NM_002781	pregnancy specific beta-1-glycoprotein 5 (PSG5),	3.1042755	0.65847205
NM_021165	family with sequence similarity 5, member B (FAM5B),	3.1014918	1.77488572
NM_000161	GTP cyclohydrolase 1 (dopa-responsive dystonia) (GCH1),	3.09096733	0.06476507
NM_006216	serine (or cysteine) proteinase inhibitor, clade E (nexin, plasminogen activator inhibitor type 1), member 2 (SERPINE2),	3.08790542	0.0498159
NM_001353	aldo-keto reductase family 1, member C1 (dihydrodiol dehydrogenase 1; 20-alpha (3-alpha)-hydroxysteroid dehydrogenase) (AKR1C1),	3.08709937	0.37529552
XM_093895	KIAA0882 protein (KIAA0882),	3.08296995	0.14930191
XM_093895	KIAA0882 protein (KIAA0882),	3.08171565	0.2524952
DR771828	SLC7A2	3.08143872	0.09322807
NM_002053	guanylate binding protein 1, interferon-inducible, 67kDa (GBP1),	3.07912607	0.35754562
NM_002426	matrix metalloproteinase 12 (macrophage elastase) (MMP12),	3.07352651	0.06412574
NM_198797	prostaglandin E synthase (PTGES), transcript variant 2,	3.07165415	0.25551386

NM_000758	colony stimulating factor 2 (granulocyte-macrophage) (CSF2),	3.06449758	0.75884011
NM_019859	5-hydroxytryptamine (serotonin) receptor 7 (adenylate cyclase-coupled) (HTR7), transcript variant d,	3.05846962	0.09757132
NM_152327	adenylate kinase 7 (AK7),	3.05828626	0.6007725
NM_198797	prostaglandin E synthase (PTGES), transcript variant 2,	3.05464783	0.0965507
XR_012825	Macaca mulatta early B-cell factor 3 (LOC713536),	3.04581987	1.47045027
NM_004613	transglutaminase 2 (C polypeptide, protein-glutamine-gamma-glutamyltransferase) (TGM2), transcript variant 1,	3.03575959	0.03490078
NM_005947	metallothionein 1B (functional) (MT1B),	3.0330617	1.25296537
NM_000577	interleukin 1 receptor antagonist (IL1RN), transcript variant 3,	3.02784587	5.65E-05
NM_000758	colony stimulating factor 2 (granulocyte-macrophage) (CSF2),	3.02004017	0.11620119
NM_031419	molecule possessing ankyrin repeats induced by lipopolysaccharide (MAIL), homolog of mouse (MAIL),	3.01890116	0.08159443
NM_021181	SLAM family member 7 (SLAMF7),	3.01498439	0.94726475
NM_032935	metallothionein IV (MT4),	3.0145272	0.6162295
NM_002638	protease inhibitor 3, skin-derived (SKALP) (PI3),	3.00959573	0.36600958
NM_015009	PDZ domain containing RING finger 3 (PDZRN3),	3.00778724	0.00867827
NM_019859	5-hydroxytryptamine (serotonin) receptor 7 (adenylate cyclase-coupled) (HTR7), transcript variant d,	3.00771376	0.53214124
NM_004458	acyl-CoA synthetase long-chain family member 4 (ACSL4), transcript variant 1,	3.00690259	0.04508171
NM_005950	metallothionein 1G (MT1G),	3.00656018	0.42047587
NM_170735	brain-derived neurotrophic factor (BDNF), transcript variant 1,	3.00234165	1.37144695
NM_019609	carboxypeptidase X (M14 family) (CPXM),	3.00196472	0.42164819
NM_001353	aldo-keto reductase family 1, member C1 (dihydrodiol dehydrogenase 1; 20-alpha (3-alpha)-hydroxysteroid dehydrogenase) (AKR1C1),	2.99968128	0.90093362
NM_00100381[illegible]	tripartite motif-containing 6 and tripartite motif-containing 34 (TRIM6-TRIM34),	2.99520957	0.33372168
NM_005949	metallothionein 1F (functional) (MT1F),	2.98967615	0.49101815
NM_017515	solute carrier family 35, member F2 (SLC35F2),	2.9869946	0.40405525
NM_172220	colony stimulating factor 3 (granulocyte) (CSF3), transcript variant 3,	2.97970138	0.2847045
NM_022842	CUB domain containing protein 1 (CDCP1), transcript variant 1,	2.96997174	0.62410715
NM_005949	metallothionein 1F (functional) (MT1F),	2.95273635	0.37524076
NM_175617	metallothionein 1E (functional) (MT1E),	2.95027695	0.49542801
NM_006274	chemokine (C-C motif) ligand 19 (CCL19),	2.94375852	0.30049328
NM_198148	carboxypeptidase X (M14 family), member 2 (CPXM2),	2.94194894	0.20000301
NM_032239	La ribonucleoprotein domain family, member 2 (LARP2), transcript variant 3,	2.93229987	2.33435862
XM_050625	secreted frizzled-related protein 2 (SFRP2),	2.93113795	0.07947681
NM_004458	acyl-CoA synthetase long-chain family member 4 (ACSL4), transcript variant 1,	2.92783361	0.02415266
NM_019859	5-hydroxytryptamine (serotonin) receptor 7 (adenylate cyclase-coupled) (HTR7), transcript variant d,	2.92683213	0.36493114
NM_058237	KIAA1622 (KIAA1622), transcript variant 1,	2.92141576	1.51902515
NM_002638	protease inhibitor 3, skin-derived (SKALP) (PI3),	2.91820665	0.3593909
NM_000640	interleukin 13 receptor, alpha 2 (IL13RA2),	2.91026661	0.32154638
NM_175617	metallothionein 1E (functional) (MT1E),	2.89830855	0.82102051
NM_152858	Wilms tumor 1 associated protein (WTAP), transcript variant 3,	2.89706392	0.06717446
NM_022842	CUB domain containing protein 1 (CDCP1), transcript variant 1,	2.89619188	0.48932619
NM_172220	colony stimulating factor 3 (granulocyte) (CSF3), transcript variant 3,	2.8860263	0.324443
NM_000617	solute carrier family 11 (proton-coupled divalent metal ion transporters), member 2 (SLC11A2),	2.87375475	0.04116848
NM_031419	molecule possessing ankyrin repeats induced by lipopolysaccharide (MAIL), homolog of mouse (MAIL),	2.86843455	0.03663657
NM_007011	abhydrolase domain containing 2 (ABHD2), transcript variant 1,	2.86686341	2.43736005
NM_015009	PDZ domain containing RING finger 3 (PDZRN3),	2.8636235	0.23994592
NM_006512	serum amyloid A4, constitutive (SAA4),	2.86037871	0.64852333

NM_178859	organic solute transporter beta (OSTbeta),	2.85869208	0.66937714
NM_002426	matrix metalloproteinase 12 (macrophage elastase) (MMP12),	2.85851477	0.22538595
NM_004864	growth differentiation factor 15 (GDF15),	2.85755135	0.36263238
NM_002309	leukemia inhibitory factor (cholinergic differentiation factor) (LIF),	2.85122297	0.30960053
NM_002232	potassium voltage-gated channel, shaker-related subfamily, member 3 (KCNA3),	2.85120405	0.29913105
NM_198148	carboxypeptidase X (M14 family), member 2 (CPXM2),	2.84242696	0.03120481
NM_005923	mitogen-activated protein kinase kinase kinase 5 (MAP3K5),	2.83443234	0.30182773
NM_002185	interleukin 7 receptor (IL7R),	2.81615016	0.53725233
NM_006274	chemokine (C-C motif) ligand 19 (CCL19),	2.81207246	0.68834573
NM_020130	chromosome 8 open reading frame 4 (C8orf4),	2.80698637	0.29001766
NM_172220	colony stimulating factor 3 (granulocyte) (CSF3), transcript variant 3,	2.79783125	0.5237584
NM_178033	cytochrome P450, family 4, subfamily X, polypeptide 1 (CYP4X1),	2.7949427	0.39960461
NM_002784	pregnancy specific beta-1-glycoprotein 9 (PSG9),	2.79378622	0.74107912
NM_004915	ATP-binding cassette, sub-family G (WHITE), member 1 (ABCG1), transcript variant 4,	2.7929564	0.03914462
NM_002185	interleukin 7 receptor (IL7R),	2.78258599	0.13886204
NM_001906	chymotrypsinogen B1 (CTRB1),	2.78030551	2.15436796
NM_052934	solute carrier family 26, member 9 (SLC26A9), transcript variant 1,	2.77238186	0.27367157
NM_003914	cyclin A1 (CCNA1),	2.77142686	1.07547141
NM_017515	solute carrier family 35, member F2 (SLC35F2),	2.77067498	0.21612827
NM_001794	cadherin 4, type 1, R-cadherin (retinal) (CDH4),	2.76678173	0.25557933
NM_003856	interleukin 1 receptor-like 1 (IL1RL1), transcript variant 2,	2.76652996	0.49571901
NM_002985	chemokine (C-C motif) ligand 5 (CCL5),	2.76581585	0.20038108
NM_006512	serum amyloid A4, constitutive (SAA4),	2.76392233	0.34684118
NM_001354	aldo-keto reductase family 1, member C2 (dihydrodiol dehydrogenase 2; bile acid binding protein; 3-alpha hydroxysteroid dehydrogenase, type III) (AKR1C2), transcript variant 1,	2.76354507	0.21895909
NM_172220	colony stimulating factor 3 (granulocyte) (CSF3), transcript variant 3,	2.75769276	0.46638321
XM_050625	secreted frizzled-related protein 2 (SFRP2),	2.75067864	0.05230012
NM_006033	lipase, endothelial (LIPG),	2.74946857	0.11363909
NM_002924	regulator of G-protein signalling 7 (RGS7),	2.74115529	0.08390273
NM_002309	leukemia inhibitory factor (cholinergic differentiation factor) (LIF),	2.73936823	0.11233489
NM_003833	matrilin 4 (MATN4), transcript variant 1,	2.73652033	2.61174287
NM_006350	follistatin (FST), transcript variant FST317,	2.72957063	0.35707117
NM_002245	potassium channel, subfamily K, member 1 (KCNK1),	2.72784675	0.85575617
XM_375284	interleukin 9 receptor (LOC400481),	2.72732732	1.52681996
NM_006350	follistatin (FST), transcript variant FST317,	2.71565584	0.06848689
NM_198504	progestin and adipoQ receptor family member IX (PAQR9),	2.70680773	0.5604813
NM_054110	UDP-N-acetyl-alpha-D-galactosamine:polypeptide N-acetylgalactosaminyltransferase-like 2 (GALNTL2),	2.70287356	0.10971344
XR_013330	Macaca mulatta deiodinase, iodothyronine, type III (DIO3),	2.69854686	0.07510218
NM_148960	claudin 19 (CLDN19),	2.69289117	2.61563118
CN641580	LDLR	2.68808013	0.11137545
NM_00100856!	nucleoporin like 1 (NUPL1), transcript variant 3,	2.68413276	1.93318518
NM_003701	tumor necrosis factor (ligand) superfamily, member 11 (TNFSF11), transcript variant 1,	2.67996043	0.63623901
NM_139072	delta-notch-like EGF repeat-containing transmembrane (DNER),	2.67651617	0.03299833
NM_006350	follistatin (FST), transcript variant FST317,	2.67402882	0.11617466
NM_005752	C-type (calcium dependent, carbohydrate-recognition domain) lectin, superfamily member 1 (cartilage-derived) (CLECSF1),	2.67082945	1.47209712

NM_002986	chemokine (C-C motif) ligand 11 (CCL11),	2.67030005	0.98638635
NM_002985	chemokine (C-C motif) ligand 5 (CCL5),	2.66791619	0.27895272
NM_176891	interferon epsilon 1 (IFNE1),	2.66342862	1.39734521
NM_005923	mitogen-activated protein kinase kinase kinase 5 (MAP3K5),	2.66340674	0.03883243
BE257326	601108540F1 NIH_MGC_16 cDNA clone IMAGE:3344889 5',	2.66152495	0.09738723
NM_052972	leucine-rich alpha-2-glycoprotein 1 (LRG1),	2.65111541	0.86253291
NM_002924	regulator of G-protein signalling 7 (RGS7),	2.65061159	0.3471613
NM_00103733	cytoplasmic FMR1 interacting protein 2 (CYFIP2), transcript variant 2,	2.64446795	1.1127793
NM_175617	metallothionein 1E (functional) (MT1E),	2.64236396	0.28063647
NM_021100	NFS1 nitrogen fixation 1 (S. cerevisiae) (NFS1), nuclear gene encoding mitochondrial protein, transcript variant 1,	2.63919073	1.9102657
NM_006350	follistatin (FST), transcript variant FST317,	2.63887686	0.46545388
NM_173515	CNKSR family member 3 (CNKSR3),	2.63322555	0.74640032
NM_004054	complement component 3a receptor 1 (C3AR1),	2.6306708	0.06263054
NM_019859	5-hydroxytryptamine (serotonin) receptor 7 (adenylate cyclase-coupled) (HTR7), transcript variant d,	2.61216359	0.05859707
NM_212479	zinc finger, MYND domain containing 11 (ZMYND11), transcript variant 2,	2.60605814	1.82092384
NM_175895	hypothetical protein FLJ25590 (FLJ25590),	2.59690724	0.4193446
NM_002006	fibroblast growth factor 2 (basic) (FGF2),	2.59115628	0.0195946
NM_004942	defensin, beta 4 (DEFB4),	2.59013744	0.02802573
NM_054110	UDP-N-acetyl-alpha-D-galactosamine:polypeptide N-acetylgalactosaminyltransferase-like 2 (GALNTL2),	2.5844209	0.20726392
NM_012242	dickkopf homolog 1 (Xenopus laevis) (DKK1),	2.58435174	0.1515862
NM_139072	delta-notch-like EGF repeat-containing transmembrane (DNER),	2.58145232	0.04687625
NM_031415	melanoma-derived leucine zipper, extra-nuclear factor (MLZE),	2.57866061	0.03997776
NM_012242	dickkopf homolog 1 (Xenopus laevis) (DKK1),	2.57853668	0.10352648
NM_032849	hypothetical protein FLJ14834 (FLJ14834),	2.57274478	0.06830963
NM_152858	Wilms tumor 1 associated protein (WTAP), transcript variant 3,	2.57090468	0.10962912
NM_003638	integrin, alpha 8 (ITGA8),	2.56658951	0.01943155
NM_001354	aldo-keto reductase family 1, member C2 (dihydrodiol dehydrogenase 2; bile acid binding protein; 3-alpha hydroxysteroid dehydrogenase, type III) (AKR1C2), transcript variant 1,	2.55961965	0.23440174
NM_022837	hypothetical protein FLJ22833 (FLJ22833),	2.54672735	2.4572323
NM_015900	phospholipase A1 member A (PLA1A),	2.54312019	0.3346419
NM_001497	UDP-Gal:betaGlcNAc beta 1,4- galactosyltransferase, polypeptide 1 (B4GALT1),	2.54077317	1.63411005
CN642474	RCL1	2.54073885	0.23305754
NM_170735	brain-derived neurotrophic factor (BDNF), transcript variant 1,	2.53649375	0.75990481
NM_022136	SAM domain, SH3 domain and nuclear localisation signals, 1 (SAMSN1),	2.53137036	0.09037588
NM_012466	tetraspanin TM4-B (TM4-B),	2.52687104	1.26030138
NM_147175	heparan sulfate 6-O-sulfotransferase 2 (HS6ST2), transcript variant S,	2.5251268	0.280296
NM_015022	PDZ domain containing 3 (PDZK3), transcript variant 2,	2.52323809	0.37447922
NM_002006	fibroblast growth factor 2 (basic) (FGF2),	2.52142831	0.02728827
NM_004054	complement component 3a receptor 1 (C3AR1),	2.52057329	0.07086429
NM_00100526	chromosome 15 open reading frame 21 (C15orf21), transcript variant 1,	2.5196588	1.70404428
NM_000270	nucleoside phosphorylase (NP),	2.51423941	0.23225544
NM_052934	solute carrier family 26, member 9 (SLC26A9), transcript variant 1,	2.51272462	0.40338907
NM_032849	hypothetical protein FLJ14834 (FLJ14834),	2.51205949	0.16290321
NM_000270	nucleoside phosphorylase (NP),	2.51022737	0.26185492
NM_152565	ATPase, H+ transporting, lysosomal 38kDa, V0 subunit d isoform 2 (ATP6V0D2),	2.50835668	1.27646035
XR_011678	Macaca mulatta transmembrane protein 63C (LOC707093),	2.50378382	1.71877142

NM_032793	hypothetical protein FLJ14490 (FLJ14490),	2.50226443	0.84875075
NM_178033	cytochrome P450, family 4, subfamily X, polypeptide 1 (CYP4X1),	2.4942744	0.26435353
NM_144710	septin 10 (SEPT10), transcript variant 1,	2.4874968	0.20190613
NM_020130	chromosome 8 open reading frame 4 (C8orf4),	2.47319313	0.14999919
NM_025079	zinc finger CCCH-type containing 12A (ZC3H12A),	2.46716241	0.11425348
NM_004170	solute carrier family 1 (neuronal/epithelial high affinity glutamate transporter, system Xag), member 1 (SLC1A1),	2.46515399	0.25763693
NM_145649	glucosaminyl (N-acetyl) transferase 2, I-branching enzyme (GCNT2), transcript variant 1,	2.46451083	1.63613265
NM_014143	CD274 antigen (CD274),	2.45800672	1.31959993
NM_001570	interleukin-1 receptor-associated kinase 2 (IRAK2),	2.4576567	0.04075442
BU933087	AGENCOURT_10473089 NIH_MGC_127 cDNA clone IMAGE:6673792 5',	2.45601133	0.1642421
XR_014707	Macaca mulatta Complement C1r subcomponent precursor (Complement component 1, r subcomponent) (LOC722131),	2.45430965	0.15378368
NM_015359	solute carrier family 39 (zinc transporter), member 14 (SLC39A14),	2.45203276	0.54686354
XM_294540	N-acylsphingosine amidohydrolase 3-like (ASAH3L),	2.45199357	0.28468185
NM_139266	signal transducer and activator of transcription 1, 91kDa (STAT1), transcript variant beta,	2.45106425	0.15073043
NM_002009	fibroblast growth factor 7 (keratinocyte growth factor) (FGF7),	2.45096563	0.03872187
NM_173198	nuclear receptor subfamily 4, group A, member 3 (NR4A3), transcript variant 2,	2.44798139	0.87642275
NM_025079	zinc finger CCCH-type containing 12A (ZC3H12A),	2.44737407	0.14790928
NM_203349	rai-like protein (RaLP),	2.44215607	0.55876988
NM_020529	nuclear factor of kappa light polypeptide gene enhancer in B-cells inhibitor, alpha (NFKBIA),	2.44092681	0.00572479
NM_001305	claudin 4 (CLDN4),	2.43825506	0.60186577
NM_012449	six transmembrane epithelial antigen of the prostate (STEAP),	2.43734132	0.97374806
NM_004170	solute carrier family 1 (neuronal/epithelial high affinity glutamate transporter, system Xag), member 1 (SLC1A1),	2.43676479	0.20912015
NM_020529	nuclear factor of kappa light polypeptide gene enhancer in B-cells inhibitor, alpha (NFKBIA),	2.43563796	0.08426402
NM_001734	complement component 1, s subcomponent (C1S), transcript variant 1,	2.4345284	0.20725166
NM_006806	BTG family, member 3 (BTG3),	2.43114526	0.15965526
NM_003638	integrin, alpha 8 (ITGA8),	2.42803008	0.15413819
NM_015900	phospholipase A1 member A (PLA1A),	2.42510652	0.10848235
NM_021020	leucine zipper, putative tumor suppressor 1 (LZTS1),	2.42320623	0.90914455
NM_005118	tumor necrosis factor (ligand) superfamily, member 15 (TNFSF15),	2.42217207	0.08815226
NM_003330	thioredoxin reductase 1 (TXNRD1), transcript variant 1,	2.41996636	0.04704254
XR_014707	Macaca mulatta Complement C1r subcomponent precursor (Complement component 1, r subcomponent) (LOC722131),	2.41987607	0.10985584
NM_005168	ras homolog gene family, member E (ARHE),	2.41278163	0.03718204
NM_001122	adipose differentiation-related protein (ADFP),	2.41253683	0.2096107
NM_000584	interleukin 8 (IL8),	2.41041471	0.22902712
NM_001777	CD47 antigen (Rh-related antigen, integrin-associated signal transducer) (CD47), transcript variant 1,	2.40891955	0.22989524
CN642474	RCL1	2.39450006	0.01934965
NM_002009	fibroblast growth factor 7 (keratinocyte growth factor) (FGF7),	2.39280583	0.41604707
NM_003082	small nuclear RNA activating complex, polypeptide 1, 43kDa (SNAPC1),	2.38786861	0.0977698
NM_004675	DIRAS family, GTP-binding RAS-like 3 (DIRAS3),	2.38465712	1.34480132
NM_001963	epidermal growth factor (beta-urogastrone) (EGF),	2.37692311	2.21476817
NM_001122	adipose differentiation-related protein (ADFP),	2.37665932	0.20461466
NM_002243	potassium inwardly-rectifying channel, subfamily J, member 15 (KCNJ15), transcript variant 2,	2.37409782	1.18470427
NM_00100523[illegible]	olfactory receptor, family 51, subfamily G, member 2 (OR51G2),	2.36909199	2.0017129
NM_080839	gamma-glutamyltransferase-like 4 (GGTL4), transcript variant 2,	2.36390659	1.53988591

NM_058237	KIAA1622 (KIAA1622), transcript variant 1,	2.36155861	0.68970479
NM_014317	trans-prenyltransferase (TPRT),	2.35706185	0.0200283
NM_000417	interleukin 2 receptor, alpha (IL2RA),	2.3559392	0.05491408
NM_002982	chemokine (C-C motif) ligand 2 (CCL2),	2.35460998	0.09080157
NM_00100143	chemokine (C-C motif) ligand 3-like, centromeric (MGC12815),	2.35299638	0.04454935
NM_181054	hypoxia-inducible factor 1, alpha subunit (basic helix-loop-helix transcription factor) (HIF1A), transcript variant 2,	2.35290678	0.11037155
NM_016733	LIM domain kinase 2 (LIMK2), transcript variant 2b,	2.35063915	0.04209584
NM_018948	mitogen-inducible gene 6 (MIG-6),	2.34756794	0.15008494
XM_294540	N-acylsphingosine amidohydrolase 3-like (ASAH3L),	2.34669809	0.00798308
NM_006041	heparan sulfate (glucosamine) 3-O-sulfotransferase 3B1 (HS3ST3B1),	2.34552843	1.58698779
NM_013343	loss of heterozygosity, 3, chromosomal region 2, gene A (LOH3CR2A),	2.34416154	1.05813698
NM_000574	decay accelerating factor for complement (CD55, Cromer blood group system) (DAF),	2.34414088	0.39319765
NM_147686	chromosome 6 open reading frame 4 (C6orf4), transcript variant 2,	2.34007998	0.11071785
NM_009587	lectin, galactoside-binding, soluble, 9 (galectin 9) (LGALS9), transcript variant long,	2.33720318	0.04097628
NM_032793	hypothetical protein FLJ14490 (FLJ14490),	2.3370472	0.19947276
NM_003330	thioredoxin reductase 1 (TXNRD1), transcript variant 1,	2.32573153	0.17815152
NM_198570	PSST739 (UNQ739),	2.32466406	1.67557881
NM_014317	trans-prenyltransferase (TPRT),	2.32312618	0.19529061
NM_173527	hypothetical protein FLJ38964 (FLJ38964),	2.32301864	0.86913501
NM_178450	membrane-associated ring finger (C3HC4) 3 (MARCH3),	2.32152256	0.5534074
NM_021181	SLAM family member 7 (SLAMF7),	2.31983985	1.10598179
NM_018948	mitogen-inducible gene 6 (MIG-6),	2.31787064	0.11217153
NM_145307	pleckstrin homology domain containing, family K member 1 (PLEKHK1),	2.31569439	2.28061456
NM_005168	ras homolog gene family, member E (ARHE),	2.3151981	0.06344668
NM_001216	carbonic anhydrase IX (CA9),	2.31379315	0.84307747
NM_00100856	nucleoporin like 1 (NUPL1), transcript variant 3,	2.31375048	0.46809012
NM_170735	brain-derived neurotrophic factor (BDNF), transcript variant 1,	2.30719436	0.03746677
NM_139266	signal transducer and activator of transcription 1, 91kDa (STAT1), transcript variant beta,	2.30027094	0.06322335
NM_016733	LIM domain kinase 2 (LIMK2), transcript variant 2b,	2.29657081	0.11164922
NM_000064	complement component 3 (C3),	2.29201889	0.48046656
NM_016170	T-cell leukemia, homeobox 2 (TLX2), transcript variant 1,	2.29081631	1.07231878
NM_017414	ubiquitin specific protease 18 (USP18),	2.29049491	0.22054018
NM_002198	interferon regulatory factor 1 (IRF1),	2.28554346	1.26029745
NM_006403	neural precursor cell expressed, developmentally down-regulated 9 (NEDD9),	2.28371906	0.01695965
NM_00100143	chemokine (C-C motif) ligand 3-like, centromeric (MGC12815),	2.27439673	0.03558012
XR_011068	Macaca mulatta hypothetical protein LOC702799 (LOC702799),	2.27410672	0.51085505
XR_012566	Macaca mulatta RNA binding motif protein 24 (LOC712107),	2.27155863	0.02332459
NM_00100840	B-cell receptor-associated protein 29 (BCAP29), transcript variant 1,	2.26972116	0.02121566
CN648055	GZMB	2.2611104	1.06315349
NM_006806	BTG family, member 3 (BTG3),	2.26079254	0.06706403
XR_013588	Macaca mulatta interleukin 33 (LOC717301),	2.25946854	0.10523677
NM_000064	complement component 3 (C3),	2.25944628	0.64439736
NM_022162	caspase recruitment domain family, member 15 (CARD15),	2.25818505	1.01758099
NM_009587	lectin, galactoside-binding, soluble, 9 (galectin 9) (LGALS9), transcript variant long,	2.25804693	0.17013729
NM_00100715	neurotrophic tyrosine kinase, receptor, type 3 (NTRK3), transcript variant 3,	2.25783862	0.96630438
NM_017565	family with sequence similarity 20, member A (FAM20A),	2.25403412	0.44840133

NM_001628	aldo-keto reductase family 1, member B1 (aldose reductase) (AKR1B1),	2.25207147	0.08565822
CB550080	MMPL0011_H01 MMPL Macaca mulatta cDNA,	2.25202496	2.17208331
NM_203371	RIKEN cDNA 1110018M03 (LOC387758),	2.25159557	0.17940285
NM_032119	monogenic, audiogenic seizure susceptibility 1 homolog (mouse) (MASS1),	2.2480147	0.00841355
NM_181054	hypoxia-inducible factor 1, alpha subunit (basic helix-loop-helix transcription factor) (HIF1A), transcript variant 2,	2.24715754	0.08299089
NM_173567	abhydrolase domain containing 7 (ABHD7),	2.24548822	0.60533043
NM_004915	ATP-binding cassette, sub-family G (WHITE), member 1 (ABCG1), transcript variant 4,	2.2439105	0.24188655
NM_004906	Wilms tumor 1 associated protein (WTAP), transcript variant 1,	2.23777381	0.18422603
NM_001778	CD48 antigen (B-cell membrane protein) (CD48),	2.23727375	0.05062455
NM_002982	chemokine (C-C motif) ligand 2 (CCL2),	2.23706479	0.04609645
NM_014382	ATPase, Ca++ transporting, type 2C, member 1 (ATP2C1), transcript variant 1,	2.23662161	1.53296892
NM_032940	polymerase (RNA) II (DNA directed) polypeptide C, 33kDa (POLR2C), transcript variant gamma,	2.23555053	2.13201617
NM_00100434(	methylenetetrahydrofolate dehydrogenase (NADP+ dependent) 2-like (MTHFD2L),	2.23471529	0.27900977
XR_012593	Macaca mulatta bradykinin receptor B1 (LOC712251),	2.23388303	0.61506328
NM_003758	eukaryotic translation initiation factor 3, subunit 1 alpha, 35kDa (EIF3S1),	2.23254381	0.11864245
CN641580	LDLR	2.23082248	0.85563187
NM_000599	insulin-like growth factor binding protein 5 (IGFBP5),	2.22323744	0.41476015
CO581591	LBP	2.21797489	1.76201798
NM_005170	achaete-scute complex-like 2 (Drosophila) (ASCL2),	2.21742975	0.32139328
NM_006203	phosphodiesterase 4D, cAMP-specific (phosphodiesterase E3 dunce homolog, Drosophila) (PDE4D),	2.2151392	0.17690231
NM_022377	intercellular adhesion molecule 4, Landsteiner-Wiener blood group (ICAM4), transcript variant 2,	2.21410763	0.19398594
NM_005121	thyroid hormone receptor associated protein 1 (THRAP1),	2.21372365	1.1518105
NM_003645	solute carrier family 27 (fatty acid transporter), member 2 (SLC27A2),	2.21273137	0.06432989
NM_002009	fibroblast growth factor 7 (keratinocyte growth factor) (FGF7),	2.20797942	0.06254672
NM_002187	interleukin 12B (natural killer cell stimulatory factor 2, cytotoxic lymphocyte maturation factor 2, p40) (IL12B),	2.20534654	0.31661711
NM_017565	family with sequence similarity 20, member A (FAM20A),	2.20491553	0.31992894
NM_203371	RIKEN cDNA 1110018M03 (LOC387758),	2.20046887	0.11496701
NM_004001	Fc fragment of IgG, low affinity IIb, receptor for (CD32) (FCGR2B),	2.20020564	0.07547087
NM_002069	guanine nucleotide binding protein (G protein), alpha inhibiting activity polypeptide 1 (GNAI1),	2.19974073	0.06253644
CN641959	PRKAR2B	2.19506481	0.06689175
NM_203391	glycerol kinase (GK), transcript variant 1,	2.19384278	1.36509343
NM_172200	interleukin 15 receptor, alpha (IL15RA), transcript variant 2,	2.19349882	0.6399103
NM_174936	proprotein convertase subtilisin/kexin type 9 (PCSK9),	2.19007541	0.09015059
NM_025246	transmembrane protein 22 (TMEM22),	2.18681502	0.02192197
NM_020297	ATP-binding cassette, sub-family C (CFTR/MRP), member 9 (ABCC9), transcript variant SUR2B,	2.18591819	1.89293837
NM_002698	POU domain, class 2, transcription factor 2 (POU2F2),	2.18448829	1.58060553
NM_005118	tumor necrosis factor (ligand) superfamily, member 15 (TNFSF15),	2.18326186	0.08117762
NM_004906	Wilms tumor 1 associated protein (WTAP), transcript variant 1,	2.17973501	0.03197339
NM_006273	chemokine (C-C motif) ligand 7 (CCL7),	2.17754535	0.81124746
XR_011068	Macaca mulatta hypothetical protein LOC702799 (LOC702799),	2.17409591	0.29977928
NM_018700	tripartite motif-containing 36 (TRIM36), transcript variant 1,	2.17114234	0.32478267
NM_025239	programmed cell death 1 ligand 2 (PDCD1LG2),	2.17077518	0.21503049
NM_080739	chromosome 20 open reading frame 141 (C20orf141),	2.16949633	1.30225581
NM_173198	nuclear receptor subfamily 4, group A, member 3 (NR4A3), transcript variant 2,	2.16743334	0.59444095
NM_018689	KIAA1199 (KIAA1199),	2.16555176	0.01178029
NM_014322	opsin 3 (encephalopsin, panopsin) (OPN3),	2.16443196	1.48584662

NM_001276	chitinase 3-like 1 (cartilage glycoprotein-39) (CHI3L1),	2.16361938	0.40285572
NM_000574	decay accelerating factor for complement (CD55, Cromer blood group system) (DAF),	2.16291687	0.05186429
NM_004864	growth differentiation factor 15 (GDF15),	2.16259864	1.11665494
XR_010861	Macaca mulatta zinc finger protein 406 isoform ZFAT-1 (LOC698512),	2.15998101	0.08975169
NM_015393	DKFZP564O0823 protein (DKFZP564O0823),	2.15988711	0.43641081
NM_001838	chemokine (C-C motif) receptor 7 (CCR7),	2.15693956	0.30229508
XM_044334	RIM binding protein 2 (KIAA0318),	2.1561676	0.11655909
NM_004001	Fc fragment of IgG, low affinity IIb, receptor for (CD32) (FCGR2B),	2.15518792	0.38947403
NM_005658	TNF receptor-associated factor 1 (TRAF1),	2.15437283	0.66465126
NM_022136	SAM domain, SH3 domain and nuclear localisation signals, 1 (SAMSN1),	2.15393745	0.01527453
NM_174936	proprotein convertase subtilisin/kexin type 9 (PCSK9),	2.15242378	0.1901937
NM_005429	vascular endothelial growth factor C (VEGFC),	2.15197694	0.03939617
NM_015022	PDZ domain containing 3 (PDZK3), transcript variant 2,	2.1483616	0.06894183
NM_001902	cystathionase (cystathionine gamma-lyase) (CTH), transcript variant 1,	2.1466162	0.27167749
XM_371039	hypothetical protein MGC19764 (MGC19764),	2.14545917	1.88199125
BU933087	AGENCOURT_10473089 NIH_MGC_127 cDNA clone IMAGE:6673792 5',	2.14370931	0.00826025
NM_005752	C-type (calcium dependent, carbohydrate-recognition domain) lectin, superfamily member 1 (cartilage-derived) (CLECSF1),	2.14263208	0.31110523
NM_000728	calcitonin-related polypeptide, beta (CALCB),	2.14218024	0.04268766
NM_015359	solute carrier family 39 (zinc transporter), member 14 (SLC39A14),	2.14135352	0.03162511
NM_003786	ATP-binding cassette, sub-family C (CFTR/MRP), member 3 (ABCC3), transcript variant MRP3,	2.13996454	0.31120892
NM_002029	formyl peptide receptor 1 (FPR1),	2.139288	0.47021188
NM_004433	E74-like factor 3 (ets domain transcription factor, epithelial-specific) (ELF3),	2.13846028	0.33752285
NM_024717	hypothetical protein FLJ22344 (FLJ22344),	2.13770519	0.06976965
NM_005143	haptoglobin (HP),	2.13390883	0.98011174
NM_009587	lectin, galactoside-binding, soluble, 9 (galectin 9) (LGALS9), transcript variant long,	2.13287309	0.06602498
NM_003465	chitinase 1 (chitotriosidase) (CHIT1),	2.13233214	0.31223511
NM_003358	UDP-glucose ceramide glucosyltransferase (UGCG),	2.12413045	0.08749113
NM_003821	receptor-interacting serine-threonine kinase 2 (RIPK2),	2.12387142	0.0024655
NM_000621	5-hydroxytryptamine (serotonin) receptor 2A (HTR2A),	2.12035737	0.09609649
NM_001216	carbonic anhydrase IX (CA9),	2.1187785	0.04584683
NM_178450	membrane-associated ring finger (C3HC4) 3 (MARCH3),	2.1177617	0.38276079
NM_147686	chromosome 6 open reading frame 4 (C6orf4), transcript variant 2,	2.11773309	0.09406487
NM_013261	peroxisome proliferative activated receptor, gamma, coactivator 1, alpha (PPARGC1A),	2.11301189	0.1663025
NM_001628	aldo-keto reductase family 1, member B1 (aldose reductase) (AKR1B1),	2.11232227	0.02472704
NM_025195	tribbles homolog 1 (Drosophila) (TRIB1),	2.11057414	0.08951458
NM_004288	pleckstrin homology, Sec7 and coiled-coil domains, binding protein (PSCDBP),	2.10956131	0.05643763
NM_170735	brain-derived neurotrophic factor (BDNF), transcript variant 1,	2.10522307	0.0594528
NM_013322	sorting nexin 10 (SNX10),	2.09623447	0.20897141
NM_031415	melanoma-derived leucine zipper, extra-nuclear factor (MLZE),	2.09530539	0.15672449
NM_015263	rabconnectin-3 (RC3),	2.090491	0.52003045
NM_006211	proenkephalin (PENK),	2.08741486	0.08714531
NM_032119	monogenic, audiogenic seizure susceptibility 1 homolog (mouse) (MASS1),	2.08666423	0.1563005
NM_212558	RIKEN A930001M12 (LOC401498),	2.08599618	0.68879317
NM_018689	KIAA1199 (KIAA1199),	2.08471242	0.01217524
NM_000189	hexokinase 2 (HK2),	2.07825267	0.21412978

NM_033667	integrin, beta 1 (fibronectin receptor, beta polypeptide, antigen CD29 includes MDF2, MSK12) (ITGB1), transcript variant 1C-1,	2.07766611	1.38753805
NM_006273	chemokine (C-C motif) ligand 7 (CCL7),	2.07678021	0.14438041
BC057815	Ras-related associated with diabetes,	2.07522861	0.0674121
NM_016235	G protein-coupled receptor, family C, group 5, member B (GPRC5B),	2.07382325	0.0730218
NM_000825	gonadotropin-releasing hormone 1 (luteinizing-releasing hormone) (GNRH1),	2.0728672	1.62752679
XR_013330	Macaca mulatta deiodinase, iodothyronine, type III (DIO3),	2.07002148	0.27159435
NM_006203	phosphodiesterase 4D, cAMP-specific (phosphodiesterase E3 dunce homolog, Drosophila) (PDE4D),	2.06907233	0.68129379
NM_001838	chemokine (C-C motif) receptor 7 (CCR7),	2.06275181	0.08449769
NM_000158	glucan (1,4-alpha-), branching enzyme 1 (glycogen branching enzyme, Andersen disease, glycogen storage disease type IV) (GBE1),	2.06201378	0.01271986
NM_001276	chitinase 3-like 1 (cartilage glycoprotein-39) (CHI3L1),	2.06085515	1.29121219
NM_000399	early growth response 2 (Krox-20 homolog, Drosophila) (EGR2),	2.05871574	0.04354727
NM_024574	hypothetical protein FLJ23191 (FLJ23191),	2.05263749	2.01748753
NM_013372	gremlin 1 homolog, cysteine knot superfamily (Xenopus laevis) (GREM1),	2.05039703	0.03188393
NM_198541	insulin growth factor-like family member 1 (IGFL1),	2.04300171	1.2836854
NM_016353	zinc finger, DHHC-type containing 2 (ZDHHC2),	2.04146546	0.93058079
NM_015892	B cell RAG associated protein (GALNAC4S-6ST),	2.03948344	0.04617457
NM_024848	hypothetical protein FLJ13941 (FLJ13941),	2.03917212	1.86366162
NM_002581	pregnancy-associated plasma protein A, pappalysin 1 (PAPPA),	2.03910915	0.19808241
NM_012223	myosin IB (MYO1B),	2.03603074	0.04134168
NM_003467	chemokine (C-X-C motif) receptor 4 (CXCR4),	2.03562253	0.05656568
NM_003467	chemokine (C-X-C motif) receptor 4 (CXCR4),	2.03375812	0.12274073
NM_030569	inter-alpha (globulin) inhibitor H5 (ITIH5), transcript variant 1,	2.0287552	0.68052971
CN641451	Hs.529772	2.02851623	0.78739727
NM_004460	fibroblast activation protein, alpha (FAP),	2.02712857	0.00289604
NM_002069	guanine nucleotide binding protein (G protein), alpha inhibiting activity polypeptide 1 (GNAI1),	2.026897	0.17201596
NM_004460	fibroblast activation protein, alpha (FAP),	2.02311753	0.09476807
NM_003465	chitinase 1 (chitotriosidase) (CHIT1),	2.02222934	0.38341379
NM_001710	B-factor, properdin (BF),	2.02025362	0.46762665
NM_001554	cysteine-rich, angiogenic inducer, 61 (CYR61),	2.01836708	0.11208364
NM_000493	collagen, type X, alpha 1(Schmid metaphyseal chondrodysplasia) (COL10A1),	2.01807837	1.20385006
NM_005564	lipocalin 2 (oncogene 24p3) (LCN2),	2.01555651	0.33569259
NM_000599	insulin-like growth factor binding protein 5 (IGFBP5),	2.01526688	0.12592896
NM_001734	complement component 1, s subcomponent (C1S), transcript variant 1,	2.01487816	0.06156928
NM_016816	2',5'-oligoadenylate synthetase 1, 40/46kDa (OAS1), transcript variant E18,	2.01242521	1.2868535
NM_005502	ATP-binding cassette, sub-family A (ABC1), member 1 (ABCA1),	2.01118184	0.05010859
NM_000158	glucan (1,4-alpha-), branching enzyme 1 (glycogen branching enzyme, Andersen disease, glycogen storage disease type IV) (GBE1),	2.01079788	0.05603138
NM_001710	B-factor, properdin (BF),	2.00857404	0.84108809
NM_005534	interferon gamma receptor 2 (interferon gamma transducer 1) (IFNGR2),	2.00716114	0.04286907
NM_006255	protein kinase C, eta (PRKCH),	2.00679207	0.01935845
NM_003358	UDP-glucose ceramide glucosyltransferase (UGCG),	2.00529795	0.10728851
NM_007203	A kinase (PRKA) anchor protein 2 (AKAP2), transcript variant 1,	2.00523879	0.07670094
NM_015892	B cell RAG associated protein (GALNAC4S-6ST),	2.00443989	0.05031424
XR_012270	Macaca mulatta hypothetical protein LOC710695 (LOC710695),	2.00354496	1.14191161

NM_001777	CD47 antigen (Rh-related antigen, integrin-associated signal transducer) (CD47), transcript variant 1,	2.00153956	0.04248904
NM_002245	potassium channel, subfamily K, member 1 (KCNK1),	2.00148227	0.3584341
NM_00100410!	G protein-coupled receptor kinase 6 (GRK6), transcript variant 3,	2.00113149	0.14797307
NM_002600	phosphodiesterase 4B, cAMP-specific (phosphodiesterase E4 dunce homolog, Drosophila) (PDE4B),	1.99974736	0.12099718
NM_004403	deafness, autosomal dominant 5 (DFNA5),	1.99512898	0.54768595
NM_000189	hexokinase 2 (HK2),	1.9933554	0.00558539
NM_004566	6-phosphofructo-2-kinase/fructose-2,6-biphosphatase 3 (PFKFB3),	1.99311481	0.23597346
NM_002742	protein kinase C, mu (PRKCM),	1.99305494	0.03235332
NM_020370	G protein-coupled receptor 84 (GPR84),	1.99006224	0.19215928
NM_000640	interleukin 13 receptor, alpha 2 (IL13RA2),	1.98985479	0.46337101
NM_173567	abhydrolase domain containing 7 (ABHD7),	1.98954834	0.27466288
NM_014011	suppressor of cytokine signaling 5 (SOCS5), transcript variant 1,	1.98726236	1.17409152
NM_003905	amyloid beta precursor protein binding protein 1, 59kDa (APPBP1),	1.98460032	1.84199667
NM_001778	CD48 antigen (B-cell membrane protein) (CD48),	1.98334534	0.04973001
NM_009587	lectin, galactoside-binding, soluble, 9 (galectin 9) (LGALS9), transcript variant long,	1.98318494	0.1345992
CN641959	PRKAR2B	1.98042705	0.16812779
NM_002232	potassium voltage-gated channel, shaker-related subfamily, member 3 (KCNA3),	1.97439522	0.46949072
NM_006417	interferon-induced protein 44 (IFI44),	1.97348556	0.25821658
NM_080283	ATP-binding cassette, sub-family A (ABC1), member 9 (ABCA9), transcript variant 1,	1.9701946	0.56714678
NM_152465	hypothetical protein MGC39650 (MGC39650),	1.96999717	0.36747838
NM_004288	pleckstrin homology, Sec7 and coiled-coil domains, binding protein (PSCDBP),	1.96565219	0.05749452
NM_001200	bone morphogenetic protein 2 (BMP2),	1.96494982	0.96209802
NM_014467	sushi-repeat-containing protein, X-linked 2 (SRPX2),	1.96461747	0.04341804
NM_000201	intercellular adhesion molecule 1 (CD54), human rhinovirus receptor (ICAM1),	1.96446981	0.02641229
NM_004403	deafness, autosomal dominant 5 (DFNA5),	1.96438139	0.59967023
NM_013363	procollagen C-endopeptidase enhancer 2 (PCOLCE2),	1.96000064	0.01401424
NM_006366	CAP, adenylate cyclase-associated protein, 2 (yeast) (CAP2),	1.95918179	0.76643868
NM_004345	cathelicidin antimicrobial peptide (CAMP),	1.95600171	0.86431444
NM_147175	heparan sulfate 6-O-sulfotransferase 2 (HS6ST2), transcript variant S,	1.95400273	0.36805881
NM_006417	interferon-induced protein 44 (IFI44),	1.95306902	0.08525575
NM_013962	neuregulin 1 (NRG1), transcript variant GGF2,	1.95040755	0.7284155
NM_001541	heat shock 27kDa protein 2 (HSPB2),	1.95032263	1.25659287
NM_012223	myosin IB (MYO1B),	1.9489539	0.15254318
NM_152565	ATPase, H+ transporting, lysosomal 38kDa, V0 subunit d isoform 2 (ATP6V0D2),	1.94882816	0.89913164
NM_016235	G protein-coupled receptor, family C, group 5, member B (GPRC5B),	1.94813934	0.12550017
NM_005502	ATP-binding cassette, sub-family A (ABC1), member 1 (ABCA1),	1.94522118	0.00753786
NM_007374	sine oculis homeobox homolog 6 (Drosophila) (SIX6),	1.94478463	1.02607366
NM_006054	reticulon 3 (RTN3), transcript variant 1,	1.9390884	1.89836748
CR601067	full-length cDNA clone CS0DC005YL10 of Neuroblastoma Cot 25-normalized of (human) [CR601067]	1.9389179	0.0366894
NM_001159	aldehyde oxidase 1 (AOX1),	1.93806512	0.16600053
NM_181506	synleurin (SLRN),	1.93785039	1.00522427
NM_003064	secretory leukocyte protease inhibitor (antileukoproteinase) (SLPI),	1.93736779	0.04677973
NM_001656	tripartite motif-containing 23 (TRIM23), transcript variant alpha,	1.93578365	1.58110427
NM_004274	A kinase (PRKA) anchor protein 6 (AKAP6),	1.93501276	0.00937706
NM_030569	inter-alpha (globulin) inhibitor H5 (ITIH5), transcript variant 1,	1.93499297	0.81688619
NM_017791	chromosome 14 open reading frame 58 (C14orf58),	1.93466397	0.22515819

XR_014091	Macaca mulatta Actin, cytoplasmic 2 (Gamma-actin) (LOC715364),	1.93408647	1.86397931
NM_147175	heparan sulfate 6-O-sulfotransferase 2 (HS6ST2), transcript variant S,	1.93382709	0.10363613
NM_020370	G protein-coupled receptor 84 (GPR84),	1.93293734	0.30140382
NM_018976	solute carrier family 38, member 2 (SLC38A2),	1.92905225	1.83462084
XR_012082	Macaca mulatta protein disulfide isomerase-associated 4 (PDIA4),	1.9286131	1.30813364
NM_001920	decorin (DCN), transcript variant A1,	1.92802979	0.10447967
NM_152309	phosphoinositide-3-kinase adaptor protein 1 (PIK3AP1),	1.92712116	0.22873074
NM_017938	hypothetical protein FLJ20716 (FLJ20716),	1.92691893	1.06989616
NM_198570	PSST739 (UNQ739),	1.92651235	1.14980611
NM_024598	hypothetical protein FLJ13154 (FLJ13154),	1.92532178	1.46339198
XM_166529	glucocorticoid induced transcript 1 (GLCCI1),	1.92442401	1.80833995
NM_014452	tumor necrosis factor receptor superfamily, member 21 (TNFRSF21),	1.92412976	0.00334918
NM_001306	claudin 3 (CLDN3),	1.9234768	0.863947
NM_004944	deoxyribonuclease I-like 3 (DNASE1L3),	1.92193848	1.64500279
NM_080283	ATP-binding cassette, sub-family A (ABC1), member 9 (ABCA9), transcript variant 1,	1.92112911	0.44037566
NM_003786	ATP-binding cassette, sub-family C (CFTR/MRP), member 3 (ABCC3), transcript variant MRP3,	1.92055172	0.22291266
NM_145202	proline-rich acidic protein 1 (PRAP1),	1.91906149	0.88096523
DR774422	Hs.529672	1.91578251	0.49128749
NM_017651	Abelson helper integration site (AHI1),	1.9127248	0.16793134
NM_002581	pregnancy-associated plasma protein A, pappalysin 1 (PAPPA),	1.91256447	0.19565884
NM_003326	tumor necrosis factor (ligand) superfamily, member 4 (tax-transcriptionally activated glycoprotein 1, 34kDa) (TNFSF4),	1.91132462	0.0625487
NM_017933	hypothetical protein FLJ20701 (FLJ20701),	1.91094829	0.05572838
NM_022162	caspase recruitment domain family, member 15 (CARD15),	1.90996827	0.69325405
NM_018360	chromosome X open reading frame 15 (CXorf15),	1.90908909	0.74522973
NM_005194	CCAAT/enhancer binding protein (C/EBP), beta (CEBPB),	1.90801121	0.09697279
NM_004972	Janus kinase 2 (a protein tyrosine kinase) (JAK2),	1.90681967	0.28610872
NM_013363	procollagen C-endopeptidase enhancer 2 (PCOLCE2),	1.90652588	0.02608229
NM_015077	sterile alpha and TIR motif containing 1 (SARM1),	1.90616936	0.48106664
NM_004414	Down syndrome critical region gene 1 (DSCR1), transcript variant 1,	1.90563467	0.17010134
CN644277	TFRC	1.90195455	0.02275397
XR_011249	Macaca mulatta protein tyrosine phosphatase, receptor type, E (PTPRE),	1.90157034	0.43650961
NM_014388	chromosome 1 open reading frame 107 (C1orf107),	1.90079455	1.40147919
DR774422	Hs.529672	1.9001963	0.42812526
NM_015393	DKFZP564O0823 protein (DKFZP564O0823),	1.89689416	0.29250623
NM_016734	paired box gene 5 (B-cell lineage specific activator) (PAX5),	1.89433747	1.30260895
NM_207517	ADAMTS-like 3 (ADAMTSL3),	1.8940825	0.32109726
NM_00100871(	RNA binding protein with multiple splicing (RBPMS), transcript variant 1,	1.89075224	0.44390939
NM_003749	insulin receptor substrate 2 (IRS2),	1.88957642	0.1404389
XR_012593	Macaca mulatta bradykinin receptor B1 (LOC712251),	1.88910065	0.25675829
NM_001554	cysteine-rich, angiogenic inducer, 61 (CYR61),	1.88856137	0.19623926
NM_017933	hypothetical protein FLJ20701 (FLJ20701),	1.88360228	0.1337476
NM_145058	hypothetical protein MGC7036 (MGC7036),	1.87947511	0.11556286
NM_005098	musculin (activated B-cell factor-1) (MSC),	1.87871112	0.20547854
NM_014701	KIAA0256 gene product (KIAA0256),	1.87730654	0.98666212
NM_004414	Down syndrome critical region gene 1 (DSCR1), transcript variant 1,	1.87691243	0.06473499

NM_020808	signal-induced proliferation-associated 1 like 2 (SIPA1L2),	1.87610144	0.01637568
CB550527	MMPL0022_D01 MMPL Macaca mulatta cDNA,	1.87591309	0.19204047
NM_198795	tudor domain containing 1 (TDRD1),	1.87423405	0.70005333
XR_011208	Macaca mulatta protein tyrosine phosphatase, receptor type, G precursor (LOC703937),	1.87388523	0.28623439
XR_010861	Macaca mulatta zinc finger protein 406 isoform ZFAT-1 (LOC698512),	1.87295642	0.20062795
NM_003749	insulin receptor substrate 2 (IRS2),	1.87019631	0.0159494
DV768600	IGFBP3	1.86990297	0.02146488
NM_013955	NADPH oxidase 1 (NOX1), transcript variant NOH-1Lv,	1.86950338	1.36326165
NM_000399	early growth response 2 (Krox-20 homolog, Drosophila) (EGR2),	1.86940186	0.04605634
NM_003064	secretory leukocyte protease inhibitor (antileukoproteinase) (SLPI),	1.86860476	0.06135042
NM_006255	protein kinase C, eta (PRKCH),	1.86835677	0.02894037
NM_018030	oxysterol binding protein-like 1A (OSBPL1A), transcript variant OSBPL1A,	1.86830895	1.61870013
XM_044334	RIM binding protein 2 (KIAA0318),	1.8678594	0.43971176
NM_148979	cathepsin H (CTSH), transcript variant 2,	1.86589769	0.05634866
NM_018465	chromosome 9 open reading frame 46 (C9orf46),	1.8644604	1.59531347
NM_024996	mitochondrial elongation factor G1 (EFG1), nuclear gene encoding mitochondrial protein,	1.86232983	1.02147593
NM_145058	hypothetical protein MGC7036 (MGC7036),	1.86224417	0.14221054
NM_001078	vascular cell adhesion molecule 1 (VCAM1), transcript variant 1,	1.85957963	0.3688484
NM_005349	recombining binding protein suppressor of hairless (Drosophila) (RBPSUH), transcript variant 1,	1.85881307	0.58915442
NM_020808	signal-induced proliferation-associated 1 like 2 (SIPA1L2),	1.85858893	0.12204981
NM_181506	synleurin (SLRN),	1.85301749	1.50576485
NM_017413	apelin, AGTRL1 ligand (APLN),	1.85016289	0.16186019
NM_005564	lipocalin 2 (oncogene 24p3) (LCN2),	1.85007912	0.27420605
NM_014452	tumor necrosis factor receptor superfamily, member 21 (TNFRSF21),	1.84880538	0.07521767
NM_025144	alpha-kinase 1 (ALPK1),	1.84811721	0.36590672
NM_004414	Down syndrome critical region gene 1 (DSCR1), transcript variant 1,	1.84668706	0.11198309
XR_013588	Macaca mulatta interleukin 33 (LOC717301),	1.84500021	0.2229074
NM_013310	chromosome 2 open reading frame 27 (C2orf27),	1.84327141	1.43778708
NM_002183	interleukin 3 receptor, alpha (low affinity) (IL3RA),	1.8429177	0.72094733
NM_003151	signal transducer and activator of transcription 4 (STAT4),	1.84208899	0.68955748
NM_005514	major histocompatibility complex, class I, B (HLA-B),	1.84194481	0.03757852
NM_147175	heparan sulfate 6-O-sulfotransferase 2 (HS6ST2), transcript variant S,	1.83915139	0.0402306
NM_003467	chemokine (C-X-C motif) receptor 4 (CXCR4),	1.83724739	0.11098097
NM_022049	G-protein coupled receptor 88 (GPR88),	1.83581711	0.35768624
NM_206967	MGC17624 protein (MGC17624),	1.83462288	0.30837584
NM_002518	neuronal PAS domain protein 2 (NPAS2),	1.83193837	0.0505436
NM_153218	hypothetical protein FLJ38725 (FLJ38725),	1.83131071	0.94894773
NM_007216	Hermansky-Pudlak syndrome 5 (HPS5), transcript variant 2,	1.83119889	0.49037077
NM_005194	CCAAT/enhancer binding protein (C/EBP), beta (CEBPB),	1.82823383	0.01149017
NM_00100723	immunoglobulin superfamily, member 3 (IGSF3), transcript variant 2,	1.82553293	0.48941218
NM_015488	myofibrillogenesis regulator 1 (MR-1),	1.82427278	0.1664914
NM_003811	tumor necrosis factor (ligand) superfamily, member 9 (TNFSF9),	1.82066831	1.46517205
XR_010897	Macaca mulatta hypothetical protein LOC702966 (LOC702966),	1.82019264	1.48928334
NM_004052	BCL2/adenovirus E1B 19kDa interacting protein 3 (BNIP3), nuclear gene encoding mitochondrial protein,	1.81799825	0.06948181
NM_005195	CCAAT/enhancer binding protein (C/EBP), delta (CEBPD),	1.81587908	0.19606323
NM_005627	serum/glucocorticoid regulated kinase (SGK),	1.8128304	0.17440059

NM_021048	melanoma antigen, family A, 10 (MAGEA10),	1.8114809	1.52778777
NM_005308	G protein-coupled receptor kinase 5 (GRK5),	1.81121722	0.99245657
NM_014376	cytoplasmic FMR1 interacting protein 2 (CYFIP2),	1.81063287	0.01195328
NM_001773	CD34 antigen (CD34),	1.81045807	0.37405356
NM_032206	nucleotide-binding oligomerization domains 27 (NOD27),	1.81010006	0.8881317
NM_000143	fumarate hydratase (FH), nuclear gene encoding mitochondrial protein,	1.80902328	0.00487908
NM_005429	vascular endothelial growth factor C (VEGFC),	1.80840136	0.08294871
NM_002619	platelet factor 4 (chemokine (C-X-C motif) ligand 4) (PF4),	1.80831402	0.22229225
NM_174926	hypothetical protein MGC17839 (MGC17839),	1.80777117	0.53272752
NM_005534	interferon gamma receptor 2 (interferon gamma transducer 1) (IFNGR2),	1.80678855	0.16105418
NM_181501	integrin, alpha 1 (ITGA1),	1.80668181	1.74452632
NM_005627	serum/glucocorticoid regulated kinase (SGK),	1.80521777	0.3835272
NM_152680	hypothetical protein FLJ32028 (FLJ32028),	1.80461314	0.66166682
NM_013372	gremlin 1 homolog, cysteine knot superfamily (Xenopus laevis) (GREM1),	1.80332325	0.06744323
NM_00100988	chromosome 22 open reading frame 9 (C22orf9), transcript variant 2,	1.80234037	0.87540245
NM_006931	solute carrier family 2 (facilitated glucose transporter), member 3 (SLC2A3),	1.80056209	0.11288508
NM_000425	L1 cell adhesion molecule (L1CAM), transcript variant 1,	1.7997122	0.69280906
NM_156036	homeo box B6 (HOXB6), transcript variant 3,	1.79948079	0.61054601
NM_003467	chemokine (C-X-C motif) receptor 4 (CXCR4),	1.79912646	0.08648567
NM_001852	collagen, type IX, alpha 2 (COL9A2),	1.79883897	0.89512968
NM_005514	major histocompatibility complex, class I, B (HLA-B),	1.79875668	0.03329459
XM_372397	olfactory receptor, family 5, subfamily M, member 9 (OR5M9),	1.79777537	1.61843492
NM_001200	bone morphogenetic protein 2 (BMP2),	1.7975685	0.14457094
BM423303	PLATE4_H02 Rhesus Macaca mulatta cDNA,	1.795803	0.25725305
NM_003558	phosphatidylinositol-4-phosphate 5-kinase, type I, beta (PIP5K1B),	1.79544586	0.18817572
NM_00100292	adenylate kinase 3-like 2 (AK3L2),	1.79538109	0.20768537
NM_016353	zinc finger, DHHC-type containing 2 (ZDHHC2),	1.79534001	0.89547044
NM_002619	platelet factor 4 (chemokine (C-X-C motif) ligand 4) (PF4),	1.79363313	0.43829278
NM_000143	fumarate hydratase (FH), nuclear gene encoding mitochondrial protein,	1.79350748	0.17973458
CK230551	PSG5	1.78769059	0.09590123
NM_182983	hepsin (transmembrane protease, serine 1) (HPN), transcript variant 1,	1.78667026	0.5193642
NM_001804	caudal type homeo box transcription factor 1 (CDX1),	1.78614141	0.21835111
CK230551	PSG5	1.78588147	0.09841999
NM_004052	BCL2/adenovirus E1B 19kDa interacting protein 3 (BNIP3), nuclear gene encoding mitochondrial protein,	1.78362385	0.06869123
NM_175898	hypothetical protein LOC283687 (LOC283687),	1.78285984	0.34105096
NM_004414	Down syndrome critical region gene 1 (DSCR1), transcript variant 1,	1.78236863	0.1033132
NM_005195	CCAAT/enhancer binding protein (C/EBP), delta (CEBPD),	1.7815122	0.00844037
XR_012476	Macaca mulatta hypothetical protein LOC711693 (LOC711693),	1.78111378	0.12771478
NM_002842	protein tyrosine phosphatase, receptor type, H (PTPRH),	1.77948098	0.84677
NM_198951	transglutaminase 2 (C polypeptide, protein-glutamine-gamma-glutamyltransferase) (TGM2), transcript variant 2,	1.77906876	0.55433086
NM_002922	regulator of G-protein signalling 1 (RGS1),	1.77905647	0.03658524
NM_003558	phosphatidylinositol-4-phosphate 5-kinase, type I, beta (PIP5K1B),	1.77851323	0.31938071
NM_001306	claudin 3 (CLDN3),	1.77770407	0.18536201
CN644277	TFRC	1.7776927	0.10576813
NM_015424	chordin-like 2 (CHRDL2),	1.77693415	0.16763897
NM_005566	lactate dehydrogenase A (LDHA),	1.77687603	0.14713713

NM_002029	formyl peptide receptor 1 (FPR1),	1.77404713	1.39633183
CN644277	TFRC	1.77189207	0.05705653
NM_148979	cathepsin H (CTSH), transcript variant 2,	1.7697767	0.03749752
NM_001450	four and a half LIM domains 2 (FHL2), transcript variant 1,	1.76975113	0.02496978
NM_194430	ribonuclease, RNase A family, 4 (RNASE4), transcript variant 1,	1.76966352	0.46291986
NM_153355	T-cell lymphoma breakpoint associated target 1 (TCBA1),	1.76965737	1.06003717
CO581591	LBP	1.76962648	1.49889866
NM_024996	mitochondrial elongation factor G1 (EFG1), nuclear gene encoding mitochondrial protein,	1.76931144	1.42643421
NM_000689	aldehyde dehydrogenase 1 family, member A1 (ALDH1A1),	1.76726807	0.18621332
NM_013438	ubiquilin 1 (UBQLN1), transcript variant 1,	1.76659493	1.56028229
NM_015488	myofibrillogenesis regulator 1 (MR-1),	1.76626917	0.04174912
NM_031479	inhibin, beta E (INHBE),	1.76565549	0.13199818
CN644277	TFRC	1.76315956	0.14015585
NM_002518	neuronal PAS domain protein 2 (NPAS2),	1.76253636	0.05134005
NM_004024	activating transcription factor 3 (ATF3),	1.76194571	1.10332701
NM_015238	KIBRA protein (KIBRA),	1.76085764	0.08207792
NM_001078	vascular cell adhesion molecule 1 (VCAM1), transcript variant 1,	1.76062691	0.41221628
NM_006403	neural precursor cell expressed, developmentally down-regulated 9 (NEDD9),	1.75789647	0.25705714
NM_182983	hepsin (transmembrane protease, serine 1) (HPN), transcript variant 1,	1.75640246	0.56958886
NM_153257	gonadotropin inducible transcription repressor 1 (GIOT-1),	1.75602634	0.45491854
NM_148979	cathepsin H (CTSH), transcript variant 2,	1.75587897	0.00581763
NM_005218	defensin, beta 1 (DEFB1),	1.75231419	0.19893825
DV768600	IGFBP3	1.75113478	0.20698934
XR_014510	Macaca mulatta leucine-rich repeat-containing G protein-coupled receptor 6 (LGR6),	1.75062174	1.64542774
CN647263	MT3	1.74952676	0.44413978
NM_018291	hypothetical protein FLJ10986 (FLJ10986),	1.74679387	0.24137086
NM_181723	EF hand domain family, member A2 (EFHA2),	1.74668108	0.46054015
NM_002526	5'-nucleotidase, ecto (CD73) (NT5E),	1.74290115	0.01174656
NM_012464	tolloid-like 1 (TLL1),	1.74233287	1.21890513
CN648055	GZMB	1.74150687	0.21946185
NM_017414	ubiquitin specific protease 18 (USP18),	1.74075618	0.15696086
NM_182901	chromosome 11 open reading frame 17 (C11orf17), transcript variant 1,	1.73898877	0.18682695
NM_000930	plasminogen activator, tissue (PLAT), transcript variant 1,	1.73884453	0.04506213
NM_006622	polo-like kinase 2 (Drosophila) (PLK2),	1.73761949	0.06608595
NM_148979	cathepsin H (CTSH), transcript variant 2,	1.73483021	0.07680556
NM_018590	chondroitin sulfate GalNAcT-2 (GALNACT-2),	1.73458709	0.07445956
NM_000240	monoamine oxidase A (MAOA), nuclear gene encoding mitochondrial protein,	1.7333203	1.14114419
BM423303	PLATE4_H02 Rhesus Macaca mulatta cDNA,	1.73285582	0.06431118
NM_007106	ubiquitin-like 3 (UBL3),	1.73272572	0.90271392
NM_003821	receptor-interacting serine-threonine kinase 2 (RIPK2),	1.73250428	0.15404966
NM_018686	cytidine monophosphate N-acetylneuraminic acid synthetase (CMAS),	1.73072567	0.01382779
NM_015017	ubiquitin specific protease 33 (USP33), transcript variant 1,	1.73049542	0.03663415
NM_130439	MAX interactor 1 (MXI1), transcript variant 2,	1.72941138	1.11716515
NM_004887	chemokine (C-X-C motif) ligand 14 (CXCL14),	1.72922018	0.38879512
CN644277	TFRC	1.72806271	0.07939175
NM_144649	hypothetical protein FLJ33069 (FLJ33069),	1.7273487	0.24438484

NM_024769	adipocyte-specific adhesion molecule (ASAM),	1.72669861	0.16962737
NM_023028	fibroblast growth factor receptor 2 (bacteria-expressed kinase, keratinocyte growth factor receptor, craniofacial dysostosis 1, Crouzon syndrome, Pfeiffer syndrome, Jackson-Weiss syndrome) (FGFR2), transcript variant 10,	1.72633893	0.14259506
NM_014079	Kruppel-like factor 15 (KLF15),	1.72596165	0.38388366
NM_001109	a disintegrin and metalloproteinase domain 8 (ADAM8),	1.72349778	0.22065144
NM_017631	hypothetical protein FLJ20035 (FLJ20035),	1.72340941	0.16154929
NM_003032	ST6 beta-galactosamide alpha-2,6-sialyltranferase 1 (ST6GAL1), transcript variant 2,	1.72225326	1.38111292
NM_004495	neuregulin 1 (NRG1), transcript variant HRG-gamma,	1.72222036	1.03459041
NM_014376	cytoplasmic FMR1 interacting protein 2 (CYFIP2),	1.721381	0.13088826
NM_018700	tripartite motif-containing 36 (TRIM36), transcript variant 1,	1.72023051	0.19120537
NM_006380	amyloid beta precursor protein (cytoplasmic tail) binding protein 2 (APPBP2),	1.71638119	0.57707361
NM_015687	filamin A interacting protein 1 (FILIP1),	1.71543902	0.00951432
NM_030912	tripartite motif-containing 8 (TRIM8),	1.71521151	0.05304724
NM_004345	cathelicidin antimicrobial peptide (CAMP),	1.71336977	1.28247925
NM_153361	hypothetical protein MGC42105 (MGC42105),	1.71096299	0.93021822
NM_000594	tumor necrosis factor (TNF superfamily, member 2) (TNF),	1.710568	0.06294942
NM_152550	SH3 domain containing ring finger 2 (SH3RF2),	1.70879937	1.04186392
NM_018283	nudix (nucleoside diphosphate linked moiety X)-type motif 15 (NUDT15),	1.70535127	0.37733438
NM_181719	hypothetical protein LOC255104 (LOC255104),	1.70389072	0.17723121
NM_019006	protein associated with PRK1 (AWP1),	1.70330939	0.11216421
NM_018240	kin of IRRE like (Drosophila) (KIRREL),	1.70280925	0.08540498
NM_001920	decorin (DCN), transcript variant A1,	1.70077229	0.00292577
CO646433	CD44	1.70077099	0.06408263
NM_000962	prostaglandin-endoperoxide synthase 1 (prostaglandin G/H synthase and cyclooxygenase) (PTGS1), transcript variant 1,	1.70011732	0.55273094
NM_030912	tripartite motif-containing 8 (TRIM8),	1.7000221	0.07657791
NM_003052	solute carrier family 34 (sodium phosphate), member 1 (SLC34A1),	1.69977786	0.07232718
NM_006076	HIV-1 Rev binding protein-like (HRBL),	1.69950247	1.42641934
NM_00100434[illegible]	FLJ16171 protein (FLJ16171),	1.69875627	0.258144
NM_017839	hypothetical protein FLJ20481 (FLJ20481),	1.69819038	0.97892715
NM_133494	NIMA (never in mitosis gene a)-related kinase 7 (NEK7),	1.69799464	0.1688622
NM_005623	chemokine (C-C motif) ligand 8 (CCL8),	1.69714278	0.08236424
NM_006775	quaking homolog, KH domain RNA binding (mouse) (QKI), transcript variant 1,	1.69668496	1.6086424
NM_199350	hypothetical protein LOC375759 (LOC375759),	1.69560861	0.42422775
NM_023028	fibroblast growth factor receptor 2 (bacteria-expressed kinase, keratinocyte growth factor receptor, craniofacial dysostosis 1, Crouzon syndrome, Pfeiffer syndrome, Jackson-Weiss syndrome) (FGFR2), transcript variant 10,	1.69483614	0.07233789
NM_002999	syndecan 4 (amphiglycan, ryudocan) (SDC4),	1.69475358	0.06488906
NM_005204	mitogen-activated protein kinase kinase kinase 8 (MAP3K8),	1.69427386	0.08636149
NM_005338	huntingtin interacting protein 1 (HIP1),	1.69421138	0.73144807
NM_005279	G protein-coupled receptor 1 (GPR1),	1.69408411	0.05934256
NM_016589	chromosome 3 open reading frame 1 (C3orf1),	1.69292084	0.89227372
NM_001159	aldehyde oxidase 1 (AOX1),	1.69282153	0.07235166
NM_002874	RAD23 homolog B (S. cerevisiae) (RAD23B),	1.69218979	1.65000161
NM_031412	GABA(A) receptor-associated protein like 1 (GABARAPL1),	1.69187671	0.03634716
NM_00100840[illegible]	B-cell receptor-associated protein 29 (BCAP29), transcript variant 1,	1.69171656	0.06432091
NM_207381	FLJ41287 protein (FLJ41287),	1.691684	0.11882264

NM_018590	chondroitin sulfate GalNAcT-2 (GALNACT-2),	1.6867354	0.0985917
NM_002742	protein kinase C, mu (PRKCM),	1.68424776	0.08486482
CN802066	Hs.432862	1.68416058	0.18547932
NM_000633	B-cell CLL/lymphoma 2 (BCL2), nuclear gene encoding mitochondrial protein, transcript variant alpha,	1.68376775	0.49389905
NM_003012	secreted frizzled-related protein 1 (SFRP1),	1.68370995	0.07048749
NM_207322	nuclear localized factor 1 (NLF1),	1.68242197	1.20461898
NM_000962	prostaglandin-endoperoxide synthase 1 (prostaglandin G/H synthase and cyclooxygenase) (PTGS1), transcript variant 1,	1.67808713	0.04344651
NM_003706	phospholipase A2, group IVC (cytosolic, calcium-independent) (PLA2G4C),	1.67671704	0.43317783
NM_014055	carnitine deficiency-associated, expressed in ventricle 1 (CDV1),	1.6756802	0.30619634
NM_002986	chemokine (C-C motif) ligand 11 (CCL11),	1.67187307	0.73607969
NM_002526	5'-nucleotidase, ecto (CD73) (NT5E),	1.66900929	0.07834023
NM_015711	glioma tumor suppressor candidate region gene 1 (GLTSCR1),	1.66768626	1.49659795
NM_003608	G protein-coupled receptor 65 (GPR65),	1.66741848	0.18436335
NM_003914	cyclin A1 (CCNA1),	1.66629464	0.68459439
NM_002922	regulator of G-protein signalling 1 (RGS1),	1.66281042	0.01468894
NM_018593	solute carrier family 16 (monocarboxylic acid transporters), member 10 (SLC16A10),	1.66115336	0.10781666
NM_031289	germ cell associated 1 (GSG1),	1.66090609	0.0943259
NM_017742	zinc finger, CCHC domain containing 2 (ZCCHC2),	1.6585111	1.04366561
NM_004972	Janus kinase 2 (a protein tyrosine kinase) (JAK2),	1.65755412	0.00069873
NM_031479	inhibin, beta E (INHBE),	1.65645442	0.22193205
NM_002928	regulator of G-protein signalling 16 (RGS16),	1.65622095	0.09792552
NM_173651	fibrous sheath interacting protein 2 (FSIP2),	1.65573546	0.00968994
NM_018593	solute carrier family 16 (monocarboxylic acid transporters), member 10 (SLC16A10),	1.65351627	0.315977
NM_032199	AT rich interactive domain 5B (MRF1-like) (ARID5B),	1.65318457	0.27793076
NM_00100871(	RNA binding protein with multiple splicing (RBPMS), transcript variant 1,	1.65123039	0.00160952
NM_198594	C1q and tumor necrosis factor related protein 1 (C1QTNF1),	1.65121907	0.13124348
NM_138416	hypothetical protein BC011001 (LOC112937),	1.65046351	1.05406546
BE257326	601108540F1 NIH_MGC_16 cDNA clone IMAGE:3344889 5',	1.65038365	0.13688434
NM_181719	hypothetical protein LOC255104 (LOC255104),	1.64899692	0.32314842
NM_005384	nuclear factor, interleukin 3 regulated (NFIL3),	1.64699955	0.10207772
NM_001656	tripartite motif-containing 23 (TRIM23), transcript variant alpha,	1.64632908	1.46671013
NM_020845	phosphatidylinositol transfer protein, membrane-associated 2 (PITPNM2),	1.64601771	0.2572181
NM_006902	paired related homeobox 1 (PRRX1), transcript variant pmx-1a,	1.64485537	0.02446883
NM_015430	regeneration associated muscle protease (DKFZP586H2123),	1.64311553	0.10584877
NM_152309	phosphoinositide-3-kinase adaptor protein 1 (PIK3AP1),	1.64303881	0.15791552
NM_014890	downregulated in ovarian cancer 1 (DOC1), transcript variant 2,	1.64099179	0.19836459
NM_003264	toll-like receptor 2 (TLR2),	1.64073884	0.10989486
NM_005384	nuclear factor, interleukin 3 regulated (NFIL3),	1.64060742	0.21705348
NM_199168	chemokine (C-X-C motif) ligand 12 (stromal cell-derived factor 1) (CXCL12),	1.64020177	0.07777309
NM_003012	secreted frizzled-related protein 1 (SFRP1),	1.63899257	0.08418097
CB550527	MMPL0022_D01 MMPL Macaca mulatta cDNA,	1.63762664	0.02971768
NM_014391	ankyrin repeat domain 1 (cardiac muscle) (ANKRD1),	1.63693991	0.02520166
NM_019083	hypothetical protein FLJ10287 (FLJ10287),	1.63677551	0.43447768
NM_015430	regeneration associated muscle protease (DKFZP586H2123),	1.63530605	0.11815686
NM_001066	tumor necrosis factor receptor superfamily, member 1B (TNFRSF1B),	1.63397511	0.01213703

NM_003151	signal transducer and activator of transcription 4 (STAT4),	1.63369567	0.28519496
NM_004884	putative neuronal cell adhesion molecule (PUNC),	1.63314681	0.07523794
NM_023028	fibroblast growth factor receptor 2 (bacteria-expressed kinase, keratinocyte growth factor receptor, craniofacial dysostosis 1, Crouzon syndrome, Pfeiffer syndrome, Jackson-Weiss syndrome) (FGFR2), transcript variant 10,	1.63226441	0.1490292
NM_007203	A kinase (PRKA) anchor protein 2 (AKAP2), transcript variant 1,	1.63115589	0.13175013
NM_002999	syndecan 4 (amphiglycan, ryudocan) (SDC4),	1.62909649	0.14267922
NM_031453	chromosome 10 open reading frame 45 (C10orf45),	1.62863148	0.07531806
NM_002192	inhibin, beta A (activin A, activin AB alpha polypeptide) (INHBA),	1.62734019	0.47436645
NM_138492	hypothetical protein MGC21644 (MGC21644), transcript variant 3,	1.62721745	0.16628272
NM_001198	PR domain containing 1, with ZNF domain (PRDM1), transcript variant 1,	1.62709956	0.31912973
NM_012329	monocyte to macrophage differentiation-associated (MMD),	1.62688515	0.12343837
NM_001109	a disintegrin and metalloproteinase domain 8 (ADAM8),	1.62547233	0.07976705
NM_173651	fibrous sheath interacting protein 2 (FSIP2),	1.62402249	0.03433922
NM_006042	heparan sulfate (glucosamine) 3-O-sulfotransferase 3A1 (HS3ST3A1),	1.62325723	0.21629776
NM_000930	plasminogen activator, tissue (PLAT), transcript variant 1,	1.62264648	0.00800734
NM_144649	hypothetical protein FLJ33069 (FLJ33069),	1.62244371	0.26828073
NM_002928	regulator of G-protein signalling 16 (RGS16),	1.6198366	0.13663195
NM_002527	neurotrophin 3 (NTF3),	1.61969115	0.78419434
NM_080860	testis specific A2 homolog (mouse) (TSGA2),	1.61953682	0.39822606
NM_00100178(	BRCC2 (BRCC2),	1.61930588	1.15795542
NM_006931	solute carrier family 2 (facilitated glucose transporter), member 3 (SLC2A3),	1.61559101	0.05443615
NM_005230	ELK3, ETS-domain protein (SRF accessory protein 2) (ELK3),	1.61521346	1.14426347
CN645851	ILLUMIGEN_MCQ_24996 Katze_MMBR Macaca mulatta cDNA clone IBIUW:10667 5' Bases 1 to 902 highly human Unigene Hs.411391,	1.61449286	0.06728069
NM_006622	polo-like kinase 2 (Drosophila) (PLK2),	1.61262136	0.07380316
NM_001450	four and a half LIM domains 2 (FHL2), transcript variant 1,	1.61208305	0.00229615
NM_013261	peroxisome proliferative activated receptor, gamma, coactivator 1, alpha (PPARGC1A),	1.61147507	0.23654308
NM_138779	hypothetical protein BC015148 (LOC93081),	1.6112996	0.42818026
NM_032918	RAS-like, estrogen-regulated, growth inhibitor (RERG),	1.60932819	0.02852831
NM_182901	chromosome 11 open reading frame 17 (C11orf17), transcript variant 1,	1.60768356	0.19502868
CO649104	SYTL3	1.60689542	0.28003107
NM_005438	FOS-like antigen 1 (FOSL1),	1.60501815	0.44480702
NM_014337	peptidylprolyl isomerase (cyclophilin)-like 2 (PPIL2), transcript variant 1,	1.6044887	0.46688218
NM_012347	F-box protein 9 (FBXO9), transcript variant 1,	1.60427793	0.43549292
NM_00100148(	ATPase, Ca++ transporting, type 2C, member 1 (ATP2C1), transcript variant 4,	1.60259672	0.01582404
NM_144503	F11 receptor (F11R), transcript variant 4,	1.59971385	0.15994714
NM_004633	interleukin 1 receptor, type II (IL1R2), transcript variant 1,	1.59819252	0.24160395
NM_198281	hypothetical protein LOC285513 (LOC285513),	1.5962865	1.09088234
AK054652	cDNA FLJ30090 fis, clone BNGH41000015 [AK054652]	1.59567523	0.33780767
NM_00100132:	ATPase, Ca++ transporting, plasma membrane 1 (ATP2B1), transcript variant 1,	1.59446682	0.03302326
NM_017791	chromosome 14 open reading frame 58 (C14orf58),	1.59334423	0.20935712
NM_012081	elongation factor, RNA polymerase II, 2 (ELL2),	1.59305495	0.05816969
NM_003326	tumor necrosis factor (ligand) superfamily, member 4 (tax-transcriptionally activated glycoprotein 1, 34kDa) (TNFSF4),	1.59188752	0.1219883
NM_002009	fibroblast growth factor 7 (keratinocyte growth factor) (FGF7),	1.59150403	0.23737626
CB550080	MMPL0011_H01 MMPL Macaca mulatta cDNA,	1.5904504	0.02081377

NM_004073	polo-like kinase 3 (Drosophila) (PLK3),	1.59041531	0.21746768
NM_000291	phosphoglycerate kinase 1 (PGK1),	1.58789502	1.0639598
NM_018569	hypothetical protein PRO0971 (PRO0971),	1.58620343	0.85505318
CK230400	ILLUMIGEN_MCQ_722 Katze_MMPL2 Macaca mulatta cDNA 5',	1.5860702	0.15526595
NM_005098	musculin (activated B-cell factor-1) (MSC),	1.58579736	0.69975242
NM_002203	integrin, alpha 2 (CD49B, alpha 2 subunit of VLA-2 receptor) (ITGA2),	1.58520277	1.47785192
NM_003052	solute carrier family 34 (sodium phosphate), member 1 (SLC34A1),	1.58294782	0.05875578
NM_002387	mutated in colorectal cancers (MCC),	1.58255588	0.03584144
CB555633	MMSP0026_B03 MMSP Macaca mulatta cDNA,	1.57958209	0.00834692
XR_012271	Macaca mulatta ATPase, Ca++ transporting, cardiac muscle, slow twitch 2 isoform 2 (LOC710702),	1.57927049	0.05135748
NM_007315	signal transducer and activator of transcription 1, 91kDa (STAT1), transcript variant alpha,	1.57881088	0.07295385
NM_000675	adenosine A2a receptor (ADORA2A),	1.5787828	0.15031016
NM_000602	serine (or cysteine) proteinase inhibitor, clade E (nexin, plasminogen activator inhibitor type 1), member 1 (SERPINE1),	1.57860692	0.09058443
NM_005566	lactate dehydrogenase A (LDHA),	1.57697216	0.13309447
NM_000953	prostaglandin D2 receptor (DP) (PTGDR),	1.57664459	0.02195846
XM_498527	LOC440068 (LOC440068),	1.57635601	0.06244438
NM_016516	vacuolar protein sorting 54 (yeast) (VPS54), transcript variant 1,	1.5754623	0.89047953
NM_020958	KIAA1622 (KIAA1622), transcript variant 2,	1.57441312	0.66370847
CK230400	ILLUMIGEN_MCQ_722 Katze_MMPL2 Macaca mulatta cDNA 5',	1.57398351	0.0092797
CO646433	CD44	1.57379299	0.01146501
NM_172200	interleukin 15 receptor, alpha (IL15RA), transcript variant 2,	1.57021439	0.29939964
NM_017651	Abelson helper integration site (AHI1),	1.56992768	0.20739165
NM_016201	angiomotin like 2 (AMOTL2),	1.56504472	0.44025509
NM_144632	hypothetical protein FLJ30294 (FLJ30294),	1.56437218	0.5234513
NM_00100662	podoplanin (PDPN), transcript variant 3,	1.56236736	0.01956818
NM_003496	transformation/transcription domain-associated protein (TRRAP),	1.56233003	1.37445464
XR_013556	Macaca mulatta PCTAIRE protein kinase 2 (LOC717183),	1.56192388	0.01280061
NM_199168	chemokine (C-X-C motif) ligand 12 (stromal cell-derived factor 1) (CXCL12),	1.56183398	0.0181802
NM_172193	kelch domain containing 1 (KLHDC1),	1.56100187	0.13975775
NM_057159	endothelial differentiation, lysophosphatidic acid G-protein-coupled receptor, 2 (EDG2), transcript variant 2,	1.56049607	0.1716895
NM_002183	interleukin 3 receptor, alpha (low affinity) (IL3RA),	1.56004348	0.10173801
NM_001975	enolase 2 (gamma, neuronal) (ENO2),	1.55994411	0.01549248
NM_003706	phospholipase A2, group IVC (cytosolic, calcium-independent) (PLA2G4C),	1.55960797	0.06479417
XR_012476	Macaca mulatta hypothetical protein LOC711693 (LOC711693),	1.55932472	0.21522015
NM_024901	hypothetical protein FLJ22457 (FLJ22457),	1.55864357	0.26220696
CN647263	MT3	1.55801948	0.02934334
NM_013252	C-type lectin domain family 5, member A (CLEC5A),	1.55616626	0.09959993
NM_00100132	ATPase, Ca++ transporting, plasma membrane 1 (ATP2B1), transcript variant 1,	1.55526851	0.00265788
CO649104	SYTL3	1.55420162	0.16497527
NM_00100871	RNA binding protein with multiple splicing (RBPMS), transcript variant 3,	1.55418775	0.1395068
NM_006456	ST6 (alpha-N-acetyl-neuraminyl-2,3-beta-galactosyl-1, 3)-N-acetylgalactosaminide alpha-2,6-sialyltransferase 2 (ST6GALNAC2),	1.55349991	0.80257441
XM_166529	glucocorticoid induced transcript 1 (GLCCI1),	1.55321911	0.29644472
CN802472	FBXO32	1.55308687	0.04086419
NM_032918	RAS-like, estrogen-regulated, growth inhibitor (RERG),	1.55222664	0.43436553

CO725402	TFPI	1.55172371	0.12284505
NM_004686	myotubularin related protein 7 (MTMR7),	1.55133649	0.08621262
NM_004675	DIRAS family, GTP-binding RAS-like 3 (DIRAS3),	1.55004395	0.05526719
NM_004155	serine (or cysteine) proteinase inhibitor, clade B (ovalbumin), member 9 (SERPINB9),	1.54774054	0.08441647
NM_201630	leucine rich repeat neuronal 5 (LRRN5), transcript variant 2,	1.54621023	1.4553855
NM_000602	serine (or cysteine) proteinase inhibitor, clade E (nexin, plasminogen activator inhibitor type 1), member 1 (SERPINE1),	1.5441249	0.02240906
NM_007315	signal transducer and activator of transcription 1, 91kDa (STAT1), transcript variant alpha,	1.54339239	0.01978202
NM_178817	melanocortin 2 receptor accessory protein (MRAP), transcript variant 1,	1.543218	0.10121705
NM_173079	RUN domain containing 1 (RUNDC1),	1.54313897	0.4222219
NM_001145	angiogenin, ribonuclease, RNase A family, 5 (ANG),	1.54300613	0.08089307
NM_014788	tripartite motif-containing 14 (TRIM14), transcript variant 1,	1.54108838	0.27724126
NM_207181	nephronophthisis 1 (juvenile) (NPHP1), transcript variant 2,	1.5409605	1.15828833
CK230400	ILLUMIGEN_MCQ_722 Katze_MMPL2 Macaca mulatta cDNA 5',	1.53922802	0.0868781
NM_153374	hypothetical protein MGC35274 (MGC35274),	1.53913459	0.33019996
NM_015443	hypothetical protein LOC284058 (LOC284058),	1.53803435	0.19600923
NM_013957	neuregulin 1 (NRG1), transcript variant HRG-beta2,	1.5379588	0.27862586
NM_152999	six transmembrane epithelial antigen of the prostate 2 (STEAP2),	1.53620892	0.09740293
NM_005406	Rho-associated, coiled-coil containing protein kinase 1 (ROCK1),	1.53517652	1.29160814
BC057815	Ras-related associated with diabetes,	1.53513003	0.12774868
NM_080474	serine (or cysteine) proteinase inhibitor, clade B (ovalbumin), member 12 (SERPINB12),	1.53505999	0.08856313
NM_001066	tumor necrosis factor receptor superfamily, member 1B (TNFRSF1B),	1.53436441	0.23031958
NM_175895	hypothetical protein FLJ25590 (FLJ25590),	1.53428003	0.30063325
NM_012449	six transmembrane epithelial antigen of the prostate (STEAP),	1.53392057	0.05565654
NM_013962	neuregulin 1 (NRG1), transcript variant GGF2,	1.5338837	0.48465392
CN647493	APOL2	1.53294243	1.13853358
NM_014583	LIM and cysteine-rich domains 1 (LMCD1),	1.53231342	1.13198586
NM_013390	transmembrane protein 2 (TMEM2),	1.531822	0.09860055
NM_025239	programmed cell death 1 ligand 2 (PDCD1LG2),	1.53135641	0.21767906
NM_182528	complement component 1, q subcomponent-like 2 (C1QL2),	1.53126147	1.12580207
NM_016569	T-box 3 (ulnar mammary syndrome) (TBX3), transcript variant 2,	1.52943832	0.16403492
NM_024901	hypothetical protein FLJ22457 (FLJ22457),	1.52774712	0.09627358
NM_004113	fibroblast growth factor 12 (FGF12), transcript variant 2,	1.52705591	0.06732181
NM_00100841(	STEAP family member 3 (STEAP3), transcript variant 3,	1.52673745	0.05065053
NM_005443	3'-phosphoadenosine 5'-phosphosulfate synthase 1 (PAPSS1),	1.52668153	0.05971582
NM_002755	mitogen-activated protein kinase kinase 1 (MAP2K1),	1.526449	0.15157925
NM_004887	chemokine (C-X-C motif) ligand 14 (CXCL14),	1.5243664	0.35411371
NM_003764	syntaxin 11 (STX11),	1.52410356	0.15612524
NM_005623	chemokine (C-C motif) ligand 8 (CCL8),	1.52157443	0.06318911
NM_133494	NIMA (never in mitosis gene a)-related kinase 7 (NEK7),	1.52100507	0.02879603
NM_001801	cysteine dioxygenase, type I (CDO1),	1.52074062	0.20920209
NM_178817	melanocortin 2 receptor accessory protein (MRAP), transcript variant 1,	1.51934496	0.20063466
NM_025196	GrpE-like 1, mitochondrial (E. coli) (GRPEL1),	1.51934387	0.19820137
NM_00100379:	RNA binding motif, single stranded interacting protein (RBMS3), transcript variant 3,	1.51910536	0.02297672
NM_001955	endothelin 1 (EDN1),	1.51808902	0.26092248
NM_004113	fibroblast growth factor 12 (FGF12), transcript variant 2,	1.51702447	0.04914396

NM_198594	C1q and tumor necrosis factor related protein 1 (C1QTNF1),	1.51635393	0.20254289
NM_173664	ADP-ribosylation factor-like 10A (ARL10A),	1.51516125	0.07319542
NM_006509	v-rel reticuloendotheliosis viral oncogene homolog B, nuclear factor of kappa light polypeptide gene enhancer in B-cells 3 (avian) (RELB),	1.51496967	0.2090569
NM_002727	proteoglycan 1, secretory granule (PRG1),	1.51458575	0.03808594
NM_021199	sulfide quinone reductase-like (yeast) (SQRDL),	1.51435137	0.05539233
NM_178493	hypothetical protein LOC147111 (LOC147111),	1.51427808	0.13055632
NM_003998	nuclear factor of kappa light polypeptide gene enhancer in B-cells 1 (p105) (NFKB1),	1.5136796	0.03802367
XM_370863	ATPase, Class I, type 8B, member 4 (ATP8B4),	1.51303534	0.09992576
NM_019000	hypothetical protein FLJ20152 (FLJ20152),	1.51218653	0.16541382
NM_001570	interleukin-1 receptor-associated kinase 2 (IRAK2),	1.51205499	0.30763711
NM_173846	chromosome 14 open reading frame 8 (C14orf8),	1.51105854	1.20462576
NM_015238	KIBRA protein (KIBRA),	1.51091603	0.13491212
CR601067	full-length cDNA clone CS0DC005YL10 of Neuroblastoma Cot 25-normalized of (human) [CR601067]	1.51086911	0.08296551
NM_012482	zinc finger protein 281 (ZNF281),	1.50864174	0.02248876
NM_018295	hypothetical protein FLJ11000 (FLJ11000),	1.50864161	0.06937638
XR_013744	Macaca mulatta four jointed box 1 (LOC717833),	1.50857978	0.17980021
CO725402	TFPI	1.50530141	0.02783648
CB555633	MMSP0026_B03 MMSP Macaca mulatta cDNA,	1.50357709	0.08178631
NM_000201	intercellular adhesion molecule 1 (CD54), human rhinovirus receptor (ICAM1),	1.50346745	0.67590813
NM_003841	tumor necrosis factor receptor superfamily, member 10c, decoy without an intracellular domain (TNFRSF10C),	1.50305656	0.11253434
NM_00100148(	ATPase, Ca++ transporting, type 2C, member 1 (ATP2C1), transcript variant 4,	1.50283655	0.13187392
NM_000361	thrombomodulin (THBD),	1.50221361	0.65674061
XR_012476	Macaca mulatta hypothetical protein LOC711693 (LOC711693),	1.50195874	0.17424918
NM_006902	paired related homeobox 1 (PRRX1), transcript variant pmx-1a,	1.50061869	0.19503103
NM_020809	Rho GTPase activating protein 20 (ARHGAP20),	1.50026979	0.04321639
NM_003764	syntaxin 11 (STX11),	1.49895135	0.33319939
NM_003338	ubiquitin-conjugating enzyme E2D 1 (UBC4/5 homolog, yeast) (UBE2D1),	1.49879359	0.00790246
NM_003615	solute carrier family 4, sodium bicarbonate cotransporter, member 7 (SLC4A7),	1.49828789	1.04290945
NM_031412	GABA(A) receptor-associated protein like 1 (GABARAPL1),	1.49804684	0.07333698
NM_001197	BCL2-interacting killer (apoptosis-inducing) (BIK),	1.49612984	1.32168459
NM_003033	ST3 beta-galactoside alpha-2,3-sialyltransferase 1 (ST3GAL1), transcript variant 1,	1.49542632	0.44113572
NM_000578	solute carrier family 11 (proton-coupled divalent metal ion transporters), member 1 (SLC11A1),	1.49381683	0.01348916
NM_006200	proprotein convertase subtilisin/kexin type 5 (PCSK5),	1.49316886	0.22554235
CN644277	TFRC	1.4926507	0.21908132
NM_019006	protein associated with PRK1 (AWP1),	1.49224291	0.20139395
NM_002217	inter-alpha (globulin) inhibitor H3 (ITIH3),	1.49064603	0.0831488
NM_005891	acetyl-Coenzyme A acetyltransferase 2 (acetoacetyl Coenzyme A thiolase) (ACAT2),	1.48994339	0.13187751
NM_018295	hypothetical protein FLJ11000 (FLJ11000),	1.48988963	0.1438084
NM_023028	fibroblast growth factor receptor 2 (bacteria-expressed kinase, keratinocyte growth factor receptor, craniofacial dysostosis 1, Crouzon syndrome, Pfeiffer syndrome, Jackson-Weiss syndrome) (FGFR2), transcript variant 10,	1.48953342	0.11635567
CK230400	ILLUMIGEN_MCQ_722 Katze_MMPL2 Macaca mulatta cDNA 5',	1.48857875	0.19043362
NM_018590	chondroitin sulfate GalNAcT-2 (GALNACT-2),	1.48854005	0.33443666
NM_000991	ribosomal protein L28 (RPL28),	1.48821381	1.03243077
NM_000594	tumor necrosis factor (TNF superfamily, member 2) (TNF),	1.48819786	0.27040177
NM_003645	solute carrier family 27 (fatty acid transporter), member 2 (SLC27A2),	1.48810528	0.11781939

NM_032105	protein phosphatase 1, regulatory (inhibitor) subunit 12B (PPP1R12B), transcript variant 2,	1.48506126	0.14810989
NM_003608	G protein-coupled receptor 65 (GPR65),	1.48313449	0.2170455
NM_175055	histone 3, H2bb (HIST3H2BB),	1.48284771	0.15567428
NM_016006	abhydrolase domain containing 5 (ABHD5),	1.48243902	0.22462724
NM_004155	serine (or cysteine) proteinase inhibitor, clade B (ovalbumin), member 9 (SERPINB9),	1.48182158	0.03687174
NM_201648	glycine-N-acyltransferase (GLYAT), nuclear gene encoding mitochondrial protein, transcript variant 1,	1.48161156	1.21069939
NM_003998	nuclear factor of kappa light polypeptide gene enhancer in B-cells 1 (p105) (NFKB1),	1.48045892	0.04756876
NM_203349	rai-like protein (RaLP),	1.47925587	0.09032081
NM_012130	claudin 14 (CLDN14), transcript variant 2,	1.47908677	1.45756731
XR_013556	Macaca mulatta PCTAIRE protein kinase 2 (LOC717183),	1.47853966	0.05939125
NM_016315	GULP, engulfment adaptor PTB domain containing 1 (GULP1),	1.47814968	0.77556083
NM_016206	vestigial-like 3 (VGL-3),	1.47802822	0.68586275
NM_198853	tripartite motif-containing 50C (TRIM50C),	1.47779367	1.40099139
NM_020530	oncostatin M (OSM),	1.47743913	0.6790196
NM_021246	lymphocyte antigen 6 complex, locus G6D (LY6G6D),	1.47726231	1.19489548
NM_147150	A kinase (PRKA) anchor protein 2 (AKAP2), transcript variant 2,	1.47684232	0.34166518
NM_00100871	RNA binding protein with multiple splicing (RBPMS), transcript variant 3,	1.47527136	0.02368146
NM_000953	prostaglandin D2 receptor (DP) (PTGDR),	1.47488305	0.04428277
NM_000689	aldehyde dehydrogenase 1 family, member A1 (ALDH1A1),	1.4742881	0.1307952
NM_012329	monocyte to macrophage differentiation-associated (MMD),	1.47353853	0.04345503
NM_005570	lectin, mannose-binding, 1 (LMAN1),	1.47324946	1.10128393
CK232441	PSG3	1.47263176	0.03453976
NM_005513	general transcription factor IIE, polypeptide 1 (alpha subunit, 56kD) (GTF2E1),	1.47237239	0.0107724
NM_006366	CAP, adenylate cyclase-associated protein, 2 (yeast) (CAP2),	1.47213844	0.42490525
NM_014322	opsin 3 (encephalopsin, panopsin) (OPN3),	1.46861865	0.46891247
NM_021238	family with sequence similarity 60, member A (FAM60A),	1.46811865	0.53675687
NM_022074	FLJ22794 protein (FLJ22794), transcript variant 1,	1.46808418	0.01943441
NM_016448	RA-regulated nuclear matrix-associated protein (RAMP),	1.46801299	0.89337172
NM_005349	recombining binding protein suppressor of hairless (Drosophila) (RBPSUH), transcript variant 1,	1.46742562	0.33649546
NM_013281	fibronectin leucine rich transmembrane protein 3 (FLRT3), transcript variant 1,	1.46683099	0.21454905
NM_018173	pleckstrin homology domain containing, family G (with RhoGef domain) member 6 (PLEKHG6),	1.46540402	0.46118729
NM_003872	neuropilin 2 (NRP2), transcript variant 2,	1.46513968	0.00302627
NM_015017	ubiquitin specific protease 33 (USP33), transcript variant 1,	1.46504682	0.09926719
NM_024873	TNFAIP3 interacting protein 3 (TNIP3),	1.46496173	0.15656405
NM_002583	PRKC, apoptosis, WT1, regulator (PAWR),	1.46380166	0.35350081
XR_012476	Macaca mulatta hypothetical protein LOC711693 (LOC711693),	1.46287631	0.24975076
NM_144503	F11 receptor (F11R), transcript variant 4,	1.46215918	0.06668334
NM_014584	ERO1-like (S. cerevisiae) (ERO1L),	1.46119719	0.13913573
NM_016348	chromosome 5 open reading frame 4 (C5orf4),	1.46111937	0.85157488
NM_052954	cysteine and tyrosine-rich 1 (CYYR1),	1.45993045	0.51310276
NM_031289	germ cell associated 1 (GSG1),	1.45962054	0.62704536
NM_156036	homeo box B6 (HOXB6), transcript variant 3,	1.4591625	0.08324829
NM_018686	cytidine monophosphate N-acetylneuraminic acid synthetase (CMAS),	1.4584867	0.01958336
NM_002755	mitogen-activated protein kinase kinase 1 (MAP2K1),	1.45728293	0.18995312
NM_002201	interferon stimulated gene 20kDa (ISG20),	1.45725766	0.18827037
NM_00100841(	STEAP family member 3 (STEAP3), transcript variant 3,	1.45665146	0.14120405

NM_004433	E74-like factor 3 (ets domain transcription factor, epithelial-specific) (ELF3),	1.45557246	0.61188254
NM_001250	CD40 antigen (TNF receptor superfamily member 5) (CD40), transcript variant 1,	1.45306682	0.21994147
NM_013246	cardiotrophin-like cytokine (CLC),	1.45283337	0.39097345
NM_003039	solute carrier family 2 (facilitated glucose/fructose transporter), member 5 (SLC2A5),	1.4522865	0.04100569
NM_018643	triggering receptor expressed on myeloid cells 1 (TREM1),	1.45199404	0.29485189
NM_000623	bradykinin receptor B2 (BDKRB2),	1.4516105	1.06209132
NM_012081	elongation factor, RNA polymerase II, 2 (ELL2),	1.45063371	0.49362988
NM_003238	transforming growth factor, beta 2 (TGFB2),	1.44808226	0.0211631
NM_018354	chromosome 20 open reading frame 46 (C20orf46),	1.44803265	0.31304615
NM_032148	solute carrier family 41, member 2 (SLC41A2),	1.44735055	0.23675716
NM_024829	hypothetical protein FLJ22662 (FLJ22662),	1.4469361	0.01010036
NM_000376	vitamin D (1,25- dihydroxyvitamin D3) receptor (VDR), transcript variant 1,	1.4468205	0.103826
NM_003872	neuropilin 2 (NRP2), transcript variant 2,	1.44586612	0.16089823
NM_018393	hypothetical protein FLJ11336 (FLJ11336),	1.44575428	0.97361633
CN802897	PXK	1.44345093	0.75808891
NM_014584	ERO1-like (S. cerevisiae) (ERO1L),	1.44264632	0.07727133
NM_057159	endothelial differentiation, lysophosphatidic acid G-protein-coupled receptor, 2 (EDG2), transcript variant 2,	1.44261616	0.05381929
NM_015443	hypothetical protein LOC284058 (LOC284058),	1.4422258	0.05424369
XR_011249	Macaca mulatta protein tyrosine phosphatase, receptor type, E (PTPRE),	1.4421687	0.13754842
NM_003286	topoisomerase (DNA) I (TOP1),	1.44199159	0.2001469
NM_014213	homeo box D9 (HOXD9),	1.44181509	1.3419299
NM_005513	general transcription factor IIE, polypeptide 1 (alpha subunit, 56kD) (GTF2E1),	1.44048065	0.12178714
DV769280	LEMD3	1.44033244	1.10711564
NM_001906	chymotrypsinogen B1 (CTRB1),	1.44013384	0.22396254
NM_012482	zinc finger protein 281 (ZNF281),	1.43990531	0.01571649
NM_207322	hypothetical LOC145741 (LOC145741),	1.43896983	0.53140836
NM_022377	intercellular adhesion molecule 4, Landsteiner-Wiener blood group (ICAM4), transcript variant 2,	1.43816682	0.26585604
NM_021199	sulfide quinone reductase-like (yeast) (SQRDL),	1.43674068	0.1158609
NM_024769	adipocyte-specific adhesion molecule (ASAM),	1.43591803	0.20147554
NM_015077	sterile alpha and TIR motif containing 1 (SARM1),	1.43562865	0.12801704
NM_178493	hypothetical protein LOC147111 (LOC147111),	1.43561048	0.57500767
NM_016081	palladin (KIAA0992),	1.43388358	0.11574953
CN648722	SLC2A3	1.43145532	0.08084343
NM_000212	integrin, beta 3 (platelet glycoprotein IIIa, antigen CD61) (ITGB3),	1.43089001	0.33322554
NM_003338	ubiquitin-conjugating enzyme E2D 1 (UBC4/5 homolog, yeast) (UBE2D1),	1.4305662	0.2604375
CK232441	PSG3	1.43018857	0.02145477
NM_181435	C1q and tumor necrosis factor related protein 3 (C1QTNF3),	1.42790387	1.00237391
NM_005063	stearoyl-CoA desaturase (delta-9-desaturase) (SCD),	1.42697911	0.09148813
NM_013281	fibronectin leucine rich transmembrane protein 3 (FLRT3), transcript variant 1,	1.42599911	0.50082716
NM_001172	arginase, type II (ARG2), nuclear gene encoding mitochondrial protein,	1.42480701	0.1565389
NM_002350	v-yes-1 Yamaguchi sarcoma viral related oncogene homolog (LYN),	1.42158376	0.02532292
CN648722	SLC2A3	1.42032619	0.04644059
NM_014373	G protein-coupled receptor 160 (GPR160),	1.41963063	1.10106492
NM_018590	chondroitin sulfate GalNAcT-2 (GALNACT-2),	1.41934062	0.05783926
CB550080	MMPL0011_H01 MMPL Macaca mulatta cDNA,	1.4187736	0.09328332
NM_007366	phospholipase A2 receptor 1, 180kDa (PLA2R1),	1.41868607	0.52096398

NM_031453	chromosome 10 open reading frame 45 (C10orf45),	1.41848568	0.05208471
NM_001766	CD1D antigen, d polypeptide (CD1D),	1.41729267	0.04404763
NM_001993	coagulation factor III (thromboplastin, tissue factor) (F3),	1.41699505	0.06231595
NM_005415	solute carrier family 20 (phosphate transporter), member 1 (SLC20A1),	1.41673529	1.11794906
NM_005891	acetyl-Coenzyme A acetyltransferase 2 (acetoacetyl Coenzyme A thiolase) (ACAT2),	1.41585405	0.09950414
NM_014267	small acidic protein (SMAP),	1.41532454	0.6029788
NM_144778	muscleblind-like 2 (Drosophila) (MBNL2), transcript variant 1,	1.41465782	0.09708337
NM_033306	caspase 4, apoptosis-related cysteine protease (CASP4), transcript variant gamma,	1.41452267	0.02835349
NM_000675	adenosine A2a receptor (ADORA2A),	1.41441084	0.32505327
NM_00101098(	ATPase, Class VI, type 11C (ATP11C), transcript variant 2,	1.41355746	0.3600959
NM_005038	peptidylprolyl isomerase D (cyclophilin D) (PPID),	1.41324107	1.37303058
NM_016315	GULP, engulfment adaptor PTB domain containing 1 (GULP1),	1.41065666	1.20387667
CN644186	Hs.100543	1.41058319	0.430496
NM_022763	FAD104 (FAD104),	1.41012857	0.1344899
NM_015424	chordin-like 2 (CHRDL2),	1.4093343	0.22945281
NM_006200	proprotein convertase subtilisin/kexin type 5 (PCSK5),	1.40904214	0.19072547
NM_002648	pim-1 oncogene (PIM1),	1.4084012	0.16723563
CN802479	NEK7	1.40760809	0.07588816
NM_032199	AT rich interactive domain 5B (MRF1-like) (ARID5B),	1.40750074	0.2199404
NM_145284	hypothetical protein MGC17347 (LOC159090),	1.40716424	0.06673664
NM_002844	protein tyrosine phosphatase, receptor type, K (PTPRK),	1.40693669	0.13692826
NM_152692	C1GALT1-specific chaperone 1 (C1GALT1C1), transcript variant 1,	1.40488243	0.73443121
NM_001497	UDP-Gal:betaGlcNAc beta 1,4- galactosyltransferase, polypeptide 1 (B4GALT1),	1.40484347	0.00394206
NM_002116	major histocompatibility complex, class I, A (HLA-A),	1.40263908	0.05233891
NM_014055	carnitine deficiency-associated, expressed in ventricle 1 (CDV1),	1.40155324	0.19865592
NM_022763	FAD104 (FAD104),	1.40052133	0.10826315
NM_013957	neuregulin 1 (NRG1), transcript variant HRG-beta2,	1.40014559	1.22777874
NM_000578	solute carrier family 11 (proton-coupled divalent metal ion transporters), member 1 (SLC11A1),	1.40010646	0.04485129
CN646564	Hs.478826	1.40002662	0.46917288
NM_014096	solute carrier family 43, member 3 (SLC43A3),	1.39942525	0.05098978
CN802479	NEK7	1.3987074	0.1000087
NM_022049	G-protein coupled receptor 88 (GPR88),	1.39786171	0.46280275
XM_498527	LOC440068 (LOC440068),	1.39768315	0.5014578
CO646894	SLC16A3	1.39710115	0.13011367
NM_014391	ankyrin repeat domain 1 (cardiac muscle) (ANKRD1),	1.39663149	0.06418217
NM_030762	basic helix-loop-helix domain containing, class B, 3 (BHLHB3),	1.39511063	0.32957926
XR_012271	Macaca mulatta ATPase, Ca++ transporting, cardiac muscle, slow twitch 2 isoform 2 (LOC710702),	1.39404075	0.12577128
NM_024829	hypothetical protein FLJ22662 (FLJ22662),	1.39321632	0.04364657
NM_002116	major histocompatibility complex, class I, A (HLA-A),	1.39286074	0.09228898
CB311076	AGENCOURT_11616903 NICHD_Rh_Ov1 Macaca mulatta cDNA clone IMAGE:6915187 5',	1.39262005	0.11726229
NM_00100379:	RNA binding motif, single stranded interacting protein (RBMS3), transcript variant 3,	1.3907958	0.44097048
NM_002224	inositol 1,4,5-triphosphate receptor, type 3 (ITPR3),	1.3880082	0.02297251
NM_004566	6-phosphofructo-2-kinase/fructose-2,6-biphosphatase 3 (PFKFB3),	1.38544836	0.1091573
NM_000247	MHC class I polypeptide-related sequence A (MICA),	1.38516677	0.385259
NM_016205	platelet derived growth factor C (PDGFC),	1.38455518	0.06326524
NM_014788	tripartite motif-containing 14 (TRIM14), transcript variant 1,	1.38348783	0.62376304

NM_016081	palladin (KIAA0992),	1.38336633	0.05528764
NM_005279	G protein-coupled receptor 1 (GPR1),	1.38280398	0.02384509
NM_020684	rhomboid, veinlet-like 7 (Drosophila) (RHBDL7),	1.37803263	0.16842607
NM_014704	glycine-, glutamate-, thienylcyclohexylpiperidine-binding protein (KIAA0562),	1.37788441	1.09090236
NM_024942	chromosome 10 open reading frame 88 (C10orf88),	1.37639729	1.0979654
NM_020215	chromosome 14 open reading frame 132 (C14orf132),	1.37626778	0.7510294
NM_016623	hypothetical protein BM-009 (BM-009),	1.37546883	1.12837053
NM_020300	microsomal glutathione S-transferase 1 (MGST1), transcript variant 1b,	1.37449384	0.12210209
NM_015150	raft-linking protein (RAFTLIN),	1.37436764	1.00192702
XM_376059	SERTA domain containing 2 (SERTAD2),	1.3737658	0.19440811
NM_002835	protein tyrosine phosphatase, non-receptor type 12 (PTPN12),	1.37244583	0.09440229
NM_213654	armadillo repeat containing 8 (ARMC8),	1.37211801	0.84088398
NM_000104	cytochrome P450, family 1, subfamily B, polypeptide 1 (CYP1B1),	1.37172573	0.00424621
NM_002791	proteasome (prosome, macropain) subunit, alpha type, 6 (PSMA6),	1.37154632	0.09431421
NM_007366	phospholipase A2 receptor 1, 180kDa (PLA2R1),	1.37104374	0.18509872
NM_005063	stearoyl-CoA desaturase (delta-9-desaturase) (SCD),	1.37097276	0.15173687
XR_012237	Macaca mulatta inositol 1,4,5-triphosphate receptor, type 1 (ITPR1),	1.3706016	0.22377583
NM_001145	angiogenin, ribonuclease, RNase A family, 5 (ANG),	1.37001673	0.03484562
NM_00100662	podoplanin (PDPN), transcript variant 3,	1.36930939	0.14608032
NM_017652	zinc finger protein 586 (ZNF586),	1.3681819	0.90247941
XR_014425	Macaca mulatta CG4203-PA (LOC720347),	1.36786728	1.20086123
NM_020300	microsomal glutathione S-transferase 1 (MGST1), transcript variant 1b,	1.36769113	0.10033214
NM_006516	solute carrier family 2 (facilitated glucose transporter), member 1 (SLC2A1),	1.36651115	0.03928258
NM_020234	x 009 protein (MDS009),	1.36626648	0.12664766
NM_002197	aconitase 1, soluble (ACO1),	1.36562608	0.09212555
NM_004073	polo-like kinase 3 (Drosophila) (PLK3),	1.36550419	0.11402374
NM_058241	cyclin T2 (CCNT2), transcript variant b,	1.36543994	0.78037931
NM_032199	AT rich interactive domain 5B (MRF1-like) (ARID5B),	1.36500249	0.14890794
NM_032827	atonal homolog 8 (Drosophila) (ATOH8),	1.36461013	0.11688946
NM_024596	microcephaly, primary autosomal recessive 1 (MCPH1),	1.36431505	1.0847466
NM_016354	solute carrier organic anion transporter family, member 4A1 (SLCO4A1),	1.36429362	0.04973068
NM_005800	chromosome 13 open reading frame 22 (C13orf22),	1.36322487	0.27091547
NM_138966	neuropilin (NRP) and tolloid (TLL)-like 1 (NETO1), transcript variant 3,	1.36247313	0.0746443
NM_170725	piggyBac transposable element derived 2 (PGBD2), transcript variant 1,	1.36219134	0.17139055
XR_010365	Macaca mulatta glucocorticoid induced transcript 1 (LOC696004),	1.36184169	0.17903854
NM_002217	inter-alpha (globulin) inhibitor H3 (ITIH3),	1.36172257	0.06795152
NM_000906	natriuretic peptide receptor A/guanylate cyclase A (atrionatriuretic peptide receptor A) (NPR1),	1.36136172	0.00226753
CN802472	FBXO32	1.36111992	0.03588683
XR_010344	Macaca mulatta dpy-19-like 3 (LOC702159),	1.36057203	0.6727019
NM_001637	acyloxyacyl hydrolase (neutrophil) (AOAH),	1.36055354	0.10003897
NM_020156	core 1 UDP-galactose:N-acetylgalactosamine-alpha-R beta 1,3-galactosyltransferase (C1GALT1),	1.36021196	0.10972569
NM_024669	hypothetical protein FLJ11795 (FLJ11795),	1.35974319	0.25141074
NM_025246	transmembrane protein 22 (TMEM22),	1.35797117	0.13306878
NM_002224	inositol 1,4,5-triphosphate receptor, type 3 (ITPR3),	1.35790255	0.02365466
NM_007216	Hermansky-Pudlak syndrome 5 (HPS5), transcript variant 2,	1.35692517	0.61856186
NM_175872	FLJ38451 protein (FLJ38451),	1.35677039	0.82762005

NM_022074	FLJ22794 protein (FLJ22794), transcript variant 1,	1.35596353	0.01379627
NM_003714	stanniocalcin 2 (STC2),	1.35484682	0.18550693
NM_000938	polymerase (RNA) II (DNA directed) polypeptide B, 140kDa (POLR2B),	1.35210701	0.64763838
NM_006517	solute carrier family 16 (monocarboxylic acid transporters), member 2 (SLC16A2),	1.34916418	0.05881578
XR_010851	Macaca mulatta hypothetical protein LOC697674 (LOC697674),	1.34887125	0.39695096
NM_00101098(	ATPase, Class VI, type 11C (ATP11C), transcript variant 2,	1.34769357	0.32572732
NM_020300	microsomal glutathione S-transferase 1 (MGST1), transcript variant 1b,	1.34650426	0.09904974
NM_020239	CDC42 small effector 1 (CDC42SE1),	1.34629615	0.72876766
NM_001993	coagulation factor III (thromboplastin, tissue factor) (F3),	1.34586785	0.05858951
NM_032827	atonal homolog 8 (Drosophila) (ATOH8),	1.34567471	0.22841692
NM_004994	matrix metalloproteinase 9 (gelatinase B, 92kDa gelatinase, 92kDa type IV collagenase) (MMP9),	1.34565284	0.11460618
NM_003486	solute carrier family 7 (cationic amino acid transporter, y+ system), member 5 (SLC7A5),	1.34416936	0.10468803
NM_030915	likely ortholog of mouse limb-bud and heart gene (LBH),	1.34401965	0.52976319
CO646894	SLC16A3	1.34365015	0.40996806
NM_032857	lactamase, beta (LACTB), nuclear gene encoding mitochondrial protein, transcript variant 1,	1.34144754	0.01469121
NM_019000	hypothetical protein FLJ20152 (FLJ20152),	1.34072047	0.14086461
NM_017947	molybdenum cofactor sulfurase (MOCOS),	1.33959183	0.07841038
NM_025147	hypothetical protein FLJ13448 (FLJ13448),	1.33713983	0.14778567
NM_018672	ATP-binding cassette, sub-family A (ABC1), member 5 (ABCA5), transcript variant 1,	1.33703636	0.11978895
NM_033087	asparagine-linked glycosylation 2 homolog (yeast, alpha-1,3-mannosyltransferase) (ALG2), transcript variant 1,	1.33655159	0.18913415
NM_003714	stanniocalcin 2 (STC2),	1.33604186	0.12685443
NM_001223	caspase 1, apoptosis-related cysteine protease (interleukin 1, beta, convertase) (CASP1), transcript variant beta,	1.33470516	0.05444516
NM_148894	family with sequence similarity 44, member A (FAM44A),	1.33402234	0.46362303
NM_016569	T-box 3 (ulnar mammary syndrome) (TBX3), transcript variant 2,	1.33304745	0.08452494
NM_001804	caudal type homeo box transcription factor 1 (CDX1),	1.33300254	0.11315037
NM_016230	NADPH cytochrome B5 oxidoreductase (NCB5OR),	1.33258944	0.08550215
CN803652	CTGF	1.33249913	0.01521908
NM_147150	A kinase (PRKA) anchor protein 2 (AKAP2), transcript variant 2,	1.3322769	0.22789592
NM_198690	keratin associated protein 10-9 (KRTAP10-9),	1.33191405	0.81073128
NM_024587	transmembrane protein 53 (TMEM53),	1.33152248	1.30820488
NM_001844	collagen, type II, alpha 1 (primary osteoarthritis, spondyloepiphyseal dysplasia, congenital) (COL2A1), transcript variant 1,	1.33149505	0.80797222
NM_015247	cylindromatosis (turban tumor syndrome) (CYLD),	1.33083056	0.05110716
NM_003238	transforming growth factor, beta 2 (TGFB2),	1.33026106	0.08831736
NM_022754	sideroflexin 1 (SFXN1),	1.32702166	1.15863284
NM_004454	ets variant gene 5 (ets-related molecule) (ETV5),	1.32690844	0.06039286
NM_175884	hypothetical protein FLJ36031 (FLJ36031),	1.32675035	1.14101108
NM_002956	restin (Reed-Steinberg cell-expressed intermediate filament-associated protein) (RSN), transcript variant 1,	1.32661884	0.23048244
NM_014788	tripartite motif-containing 14 (TRIM14), transcript variant 1,	1.32656065	0.30342102
NM_014735	PHD finger protein 16 (PHF16),	1.32641684	1.18721891
NM_032717	hypothetical protein MGC11324 (MGC11324),	1.32632241	1.05830636
NM_00100291(	hypothetical protein LOC285016 (LOC285016),	1.32593733	0.63296083
XR_010654	Macaca mulatta acyl-CoA synthetase long-chain family member 5 isoform a (LOC696404),	1.32559665	0.42603393
NM_198270	Nance-Horan syndrome (congenital cataracts and dental anomalies) (NHS),	1.325485	1.16715593
NM_152715	hypothetical protein MGC10233 (MGC10233),	1.32481265	0.22787081
NM_012328	DnaJ (Hsp40) homolog, subfamily B, member 9 (DNAJB9),	1.32382303	0.0681126

XR_010116	Macaca mulatta interleukin 19 isoform 1 precursor (LOC694806),	1.32351899	1.11731997
NM_003900	sequestosome 1 (SQSTM1),	1.32254	0.87681714
NM_003486	solute carrier family 7 (cationic amino acid transporter, y+ system), member 5 (SLC7A5),	1.32179209	0.05452153
XR_012394	Macaca mulatta putative small membrane protein NID67 (LOC711300),	1.32148559	0.40726643
XR_012325	Macaca mulatta hypothetical protein LOC710960 (LOC710960),	1.32144531	0.14344469
NM_004465	fibroblast growth factor 10 (FGF10),	1.32041748	0.54139996
NM_016029	dehydrogenase/reductase (SDR family) member 7 (DHRS7),	1.32031778	0.14455869
CN802472	FBXO32	1.32027666	0.18680386
NM_019113	fibroblast growth factor 21 (FGF21),	1.31942329	0.76876472
NM_002727	proteoglycan 1, secretory granule (PRG1),	1.31936213	0.11212101
NM_032199	AT rich interactive domain 5B (MRF1-like) (ARID5B),	1.31806808	0.14927307
NM_005438	FOS-like antigen 1 (FOSL1),	1.3178243	0.2731845
NM_022497	mitochondrial ribosomal protein S25 (MRPS25), nuclear gene encoding mitochondrial protein,	1.31752158	1.01677435
NM_002032	ferritin, heavy polypeptide 1 (FTH1),	1.31675323	0.03856131
CO581942	HLA-DQA1	1.31645665	0.79007049
CB309570	AGENCOURT_11830463 NICHD_Rh_Ov1 Macaca mulatta cDNA clone IMAGE:6916521 5',	1.31627991	0.39366233
XR_012582	Macaca mulatta retinoic acid receptor responder (tazarotene induced) 1 isoform 1 (LOC703781),	1.31590117	0.15234094
NM_001196	BH3 interacting domain death agonist (BID), transcript variant 2,	1.31483541	0.83052648
NM_177551	G protein-coupled receptor 109A (GPR109A),	1.31429335	0.10864719
NM_144503	F11 receptor (F11R), transcript variant 4,	1.31395901	0.0600419
NM_052947	heart alpha-kinase (HAK),	1.31350848	0.17131632
NM_030817	hypothetical protein DKFZp434F0318 (DKFZP434F0318),	1.31339949	0.84012185
NM_006744	retinol binding protein 4, plasma (RBP4),	1.31013567	0.02932726
NM_178349	late cornified envelope 1B (LCE1B),	1.30830938	1.12010455
NM_00100410!	G protein-coupled receptor kinase 6 (GRK6), transcript variant 3,	1.30738327	0.11993755
NM_000361	thrombomodulin (THBD),	1.30665093	0.21278242
NM_018898	protocadherin alpha subfamily C, 1 (PCDHAC1), transcript variant 1,	1.30643348	0.04399908
NM_138460	chemokine-like factor super family 5 (CKLFSF5), transcript variant 1,	1.30619581	0.77659101
NM_002387	mutated in colorectal cancers (MCC),	1.30602669	0.23090754
NM_006517	solute carrier family 16 (monocarboxylic acid transporters), member 2 (SLC16A2),	1.30555479	0.0898105
NM_019895	chromosome 3 open reading frame 4 (C3orf4),	1.3051692	0.18137792
NM_000520	hexosaminidase A (alpha polypeptide) (HEXA),	1.30427299	0.74307762
NM_175055	histone 3, H2bb (HIST3H2BB),	1.30370439	0.78489205
NM_022572	myofibrillogenesis regulator 1 (MR-1),	1.3032847	0.23670809
NM_207435	FLJ40142 protein (FLJ40142),	1.30214153	1.06223056
NM_001505	G protein-coupled receptor 30 (GPR30),	1.30053227	0.17463005
AK127395	cDNA FLJ45486 fis, clone BRTHA2002726 [AK127395]	1.30035872	0.14192608
NM_033306	caspase 4, apoptosis-related cysteine protease (CASP4), transcript variant gamma,	1.2999339	0.12118169
CK231449	LOC283241	1.29962506	0.09314909
CN802472	FBXO32	1.29891953	0.17234621
NM_004905	peroxiredoxin 6 (PRDX6),	1.29857412	0.12858713
NM_006042	heparan sulfate (glucosamine) 3-O-sulfotransferase 3A1 (HS3ST3A1),	1.29832889	0.17671333
NM_020244	choline phosphotransferase 1 (CHPT1),	1.29794385	0.89409963
NM_032021	AD031 protein (AD031),	1.29790605	0.26258132
AK075380	cDNA PSEC0070 fis, clone NT2RP2001508, highly Source of immunodominant MHC-associated peptides [AK075380]	1.29600282	1.01118216
NM_002502	nuclear factor of kappa light polypeptide gene enhancer in B-cells 2 (p49/p100) (NFKB2),	1.29473761	0.6996018

NM_020648	twisted gastrulation homolog 1 (Drosophila) (TWSG1),	1.29452976	1.01163323
NM_013272	solute carrier organic anion transporter family, member 3A1 (SLCO3A1),	1.29447316	0.91756635
NM_016006	abhydrolase domain containing 5 (ABHD5),	1.29436396	0.23932133
NM_199350	hypothetical protein LOC375759 (LOC375759),	1.29429583	0.20894718
NM_003047	solute carrier family 9 (sodium/hydrogen exchanger), isoform 1 (antiporter, Na+/H+, amiloride sensitive) (SLC9A1),	1.29410704	1.21077214
NM_000376	vitamin D (1,25- dihydroxyvitamin D3) receptor (VDR), transcript variant 1,	1.29394679	0.05396487
NM_001637	acyloxyacyl hydrolase (neutrophil) (AOAH),	1.29150736	0.26080467
NM_005443	3'-phosphoadenosine 5'-phosphosulfate synthase 1 (PAPSS1),	1.29084942	0.13199575
NM_000676	adenosine A2b receptor (ADORA2B),	1.29077065	0.07199201
NM_175892	hypothetical protein FLJ37266 (FLJ37266),	1.29063551	0.34018472
NM_014467	sushi-repeat-containing protein, X-linked 2 (SRPX2),	1.28946249	0.19482653
NM_006291	tumor necrosis factor, alpha-induced protein 2 (TNFAIP2),	1.28873942	0.20359866
NM_001912	cathepsin L (CTSL), transcript variant 1,	1.28812063	0.17542147
NM_014631	SH3 and PX domains 2A (SH3PXD2A),	1.28659249	0.11406752
NM_134268	cytoglobin (CYGB),	1.28645853	0.89745238
NM_031442	transmembrane 4 superfamily member 10 (TM4SF10),	1.28584892	0.245614
NM_002857	peroxisomal biogenesis factor 19 (PEX19),	1.28558164	0.06879866
NM_019027	RNA-binding protein (FLJ20273),	1.28501892	0.59458113
NM_003039	solute carrier family 2 (facilitated glucose/fructose transporter), member 5 (SLC2A5),	1.28457073	0.17577327
NM_00100171(	nuclear factor of kappa light polypeptide gene enhancer in B-cells inhibitor, beta (NFKBIB), transcript variant 2,	1.28385578	0.19493303
NM_144503	F11 receptor (F11R), transcript variant 4,	1.28300149	0.01774527
XR_012693	Macaca mulatta acyl-CoA synthetase long-chain family member 6 isoform b (LOC706847),	1.28230603	0.77894286
BF683837	602140129F1 NIH_MGC_46 cDNA clone IMAGE:4301287 5',	1.27973565	0.59352621
NM_021965	phosphoglucomutase 5 (PGM5),	1.27879047	0.43724197
NM_004760	serine/threonine kinase 17a (apoptosis-inducing) (STK17A),	1.2781909	0.38710501
NM_152308	hypothetical protein MGC24665 (MGC24665),	1.27716034	0.87179897
NM_007223	putative G protein coupled receptor (GPR),	1.27673617	0.27842809
NM_000786	cytochrome P450, family 51, subfamily A, polypeptide 1 (CYP51A1),	1.27632107	0.14713495
NM_015687	filamin A interacting protein 1 (FILIP1),	1.27622312	0.11580748
NM_019895	chromosome 3 open reading frame 4 (C3orf4),	1.27471011	0.19325446
NM_003045	solute carrier family 7 (cationic amino acid transporter, y+ system), member 1 (SLC7A1),	1.27466117	0.28884861
XM_376370	FLJ33360 protein (FLJ33360),	1.27349521	0.0081642
NM_018534	neuropilin 2 (NRP2), transcript variant 4,	1.27322736	1.1699191
NM_006207	platelet-derived growth factor receptor-like (PDGFRL),	1.2727027	0.03966686
XM_496419	ets variant gene 3; ETS-domain transcriptional repressor; ETS-domain protein; mitogenic Ets transcriptional suppressor METS (LOC440695),	1.27183814	0.30218712
NM_018291	hypothetical protein FLJ10986 (FLJ10986),	1.27182935	0.07879739
NM_00100718(	bovine IgA regulatory protein (LOC492311),	1.27115792	1.1323432
NM_002129	high-mobility group box 2 (HMGB2),	1.27005165	0.05146467
NM_020809	Rho GTPase activating protein 20 (ARHGAP20),	1.26975725	0.19039027
NM_018413	carbohydrate (chondroitin 4) sulfotransferase 11 (CHST11),	1.26898488	0.03069604
NM_015173	TBC1 (tre-2/USP6, BUB2, cdc16) domain family, member 1 (TBC1D1),	1.26760314	0.05439633
NM_000097	coproporphyrinogen oxidase (CPOX),	1.26710302	0.54071425
NM_024101	melanophilin (MLPH),	1.26686519	0.01939493
NM_021194	solute carrier family 30 (zinc transporter), member 1 (SLC30A1),	1.26683508	0.14125307

NM_005081	zinc finger protein 142 (clone pHZ-49) (ZNF142),	1.26680921	0.58612856
NM_018643	triggering receptor expressed on myeloid cells 1 (TREM1),	1.26639177	0.20811884
NM_015247	cylindromatosis (turban tumor syndrome) (CYLD),	1.26628352	0.31191639
NM_014701	KIAA0256 gene product (KIAA0256),	1.2659176	0.04302144
NM_024420	phospholipase A2, group IVA (cytosolic, calcium-dependent) (PLA2G4A),	1.26580661	0.52827087
NM_004994	matrix metalloproteinase 9 (gelatinase B, 92kDa gelatinase, 92kDa type IV collagenase) (MMP9),	1.26397992	0.12340568
NM_022377	intercellular adhesion molecule 4, Landsteiner-Wiener blood group (ICAM4), transcript variant 2,	1.26350317	0.34209689
NM_002662	phospholipase D1, phophatidylcholine-specific (PLD1),	1.26279455	0.65509852
NM_001773	CD34 antigen (CD34),	1.26279284	0.45965122
NM_175892	hypothetical protein FLJ37266 (FLJ37266),	1.26213763	0.19974087
NM_007194	CHK2 checkpoint homolog (S. pombe) (CHEK2), transcript variant 1,	1.26192335	0.03106042
NM_017817	RAB20, member RAS oncogene family (RAB20),	1.26052112	0.00154995
NM_005550	kinesin family member C3 (KIFC3),	1.26040191	0.0800616
NM_016029	dehydrogenase/reductase (SDR family) member 7 (DHRS7),	1.2603911	0.00485902
NM_000609	chemokine (C-X-C motif) ligand 12 (stromal cell-derived factor 1) (CXCL12),	1.26012941	0.35215683
NM_001975	enolase 2 (gamma, neuronal) (ENO2),	1.25960892	0.12117538
NM_006509	v-rel reticuloendotheliosis viral oncogene homolog B, nuclear factor of kappa light polypeptide gene enhancer in B-cells 3 (avian) (RELB),	1.25925598	0.16879491
CN805444	Hs.162601	1.25916857	1.1219594
NM_213651	solute carrier family 25 (mitochondrial carrier; phosphate carrier), member 24 (SLC25A24), transcript variant 2,	1.25909314	1.0026309
NM_144503	F11 receptor (F11R), transcript variant 4,	1.25869093	0.02680637
NM_00100715(	neurotrophic tyrosine kinase, receptor, type 3 (NTRK3), transcript variant 3,	1.25858302	0.08401517
NM_032312	hypothetical protein MGC11061 (MGC11061),	1.25832994	1.02211089
NM_001250	CD40 antigen (TNF receptor superfamily member 5) (CD40), transcript variant 1,	1.25826211	0.02798371
NM_207485	FLJ41327 protein (FLJ41327),	1.25800553	0.28202602
NM_207337	hypothetical protein LOC196394 (LOC196394),	1.25747401	1.2285233
NM_013402	fatty acid desaturase 1 (FADS1),	1.25596623	0.24952662
NM_013402	fatty acid desaturase 1 (FADS1),	1.25532377	0.1148845
NM_020684	rhomboid, veinlet-like 7 (Drosophila) (RHBDL7),	1.25521348	0.06354818
NM_014683	unc-51-like kinase 2 (C. elegans) (ULK2),	1.25412919	0.00987927
NM_020452	ATPase, Class I, type 8B, member 2 (ATP8B2), transcript variant 1,	1.25390464	1.04493576
NM_022168	interferon induced with helicase C domain 1 (IFIH1),	1.25286605	0.41228727
NM_017523	XIAP associated factor-1 (HSXIAPAF1), transcript variant 1,	1.25286468	0.17508657
NM_003082	small nuclear RNA activating complex, polypeptide 1, 43kDa (SNAPC1),	1.25258077	0.36892503
NM_019555	Rho guanine nucleotide exchange factor (GEF) 3 (ARHGEF3),	1.25167222	0.14484675
NM_005550	kinesin family member C3 (KIFC3),	1.25023045	0.05693201
NM_015683	arrestin domain containing 2 (ARRDC2), transcript variant 1,	1.2496882	0.50616124
XR_013744	Macaca mulatta four jointed box 1 (LOC717833),	1.24939672	0.20487053
NM_000104	cytochrome P450, family 1, subfamily B, polypeptide 1 (CYP1B1),	1.24900029	0.15075484
NM_002350	v-yes-1 Yamaguchi sarcoma viral related oncogene homolog (LYN),	1.24870787	0.13084675
NM_003136	signal recognition particle 54kDa (SRP54),	1.24736309	1.12237418
NM_003597	TGFB inducible early growth response 2 (TIEG2),	1.24669592	0.00334058
NM_012347	F-box protein 9 (FBXO9), transcript variant 1,	1.2464794	0.10261444
NM_032647	chromobox homolog 2 (Pc class homolog, Drosophila) (CBX2), transcript variant 2,	1.24595173	1.0589572
NM_144503	F11 receptor (F11R), transcript variant 4,	1.2454081	0.096142
NM_003264	toll-like receptor 2 (TLR2),	1.24450003	0.03819636

NM_006207	platelet-derived growth factor receptor-like (PDGFRL),	1.24448812	0.08632329
NM_207343	hypothetical protein DKFZp547C195 (DKFZp547C195),	1.24436652	0.24160966
NM_005308	G protein-coupled receptor kinase 5 (GRK5),	1.24391073	0.06344358
NM_030762	basic helix-loop-helix domain containing, class B, 3 (BHLHB3),	1.24216716	0.5196588
CO583502	UAP1	1.24206971	0.01717159
NM_00100291!	hypothetical protein LOC285016 (LOC285016),	1.24174329	0.42003567
NM_015368	pannexin 1 (PANX1),	1.23993322	0.23761765
NM_00100292:	adenylate kinase 3-like 2 (AK3L2),	1.23980976	0.17202484
NM_002791	proteasome (prosome, macropain) subunit, alpha type, 6 (PSMA6),	1.23942127	0.16344569
NM_000150	fucosyltransferase 6 (alpha (1,3) fucosyltransferase) (FUT6),	1.23837016	0.04218952
NM_003242	transforming growth factor, beta receptor II (70/80kDa) (TGFBR2),	1.23835852	0.22239836
NM_002241	potassium inwardly-rectifying channel, subfamily J, member 10 (KCNJ10),	1.23814505	0.6120352
NM_018306	transmembrane protein 40 (TMEM40),	1.23788788	1.18867528
NM_052889	CARD only protein (COPl), transcript variant 2,	1.23765916	0.0614305
NM_000906	natriuretic peptide receptor A/guanylate cyclase A (atrionatriuretic peptide receptor A) (NPR1),	1.23687198	0.15675914
NM_182532	hypothetical protein LOC199964 (LOC199964),	1.23687134	0.56945568
NM_006223	protein (peptidyl-prolyl cis/trans isomerase) NIMA-interacting, 4 (parvulin) (PIN4),	1.2361892	0.79899718
NM_022486	sushi domain containing 1 (SUSD1),	1.23599884	0.87909682
CO583502	UAP1	1.2357718	0.00932516
NM_002427	matrix metalloproteinase 13 (collagenase 3) (MMP13),	1.23526288	0.34606253
NM_018222	parvin, alpha (PARVA),	1.23525087	1.00666402
NM_005618	delta-like 1 (Drosophila) (DLL1),	1.23522733	0.30161191
NM_020234	x 009 protein (MDS009),	1.23490247	0.11827847
NM_018325	chromosome 9 open reading frame 72 (C9orf72), transcript variant 1,	1.23473317	0.33936823
NM_031266	heterogeneous nuclear ribonucleoprotein A/B (HNRPAB), transcript variant 1,	1.23467621	0.67694247
XR_012348	Macaca mulatta SHC transforming protein 1 (SH2 domain protein C1) (Src homology 2 domain-containing transforming protein C1) (LOC711071),	1.23427704	0.63436442
NM_001844	collagen, type II, alpha 1 (primary osteoarthritis, spondyloepiphyseal dysplasia, congenital) (COL2A1), transcript variant 1,	1.23325614	0.28867229
NM_014782	armadillo repeat containing, X-linked 2 (ARMCX2),	1.23191799	0.10384906
NM_002600	phosphodiesterase 4B, cAMP-specific (phosphodiesterase E4 dunce homolog, Drosophila) (PDE4B),	1.23176668	0.05327189
NM_024101	melanophilin (MLPH),	1.23136877	0.00284226
NM_052947	heart alpha-kinase (HAK),	1.22983933	0.12699735
NM_031217	kinesin family member 18A (KIF18A),	1.22948904	0.57317324
NM_006502	polymerase (DNA directed), eta (POLH),	1.22938575	0.68217923
NM_024670	suppressor of variegation 3-9 homolog 2 (Drosophila) (SUV39H2),	1.22911841	0.71548099
NM_004586	ribosomal protein S6 kinase, 90kDa, polypeptide 3 (RPS6KA3),	1.22909102	0.93467859
NM_00100148:	hypothetical protein FLJ11011 (FLJ11011), transcript variant 1,	1.22779764	0.91053288
NM_138779	hypothetical protein BC015148 (LOC93081),	1.22718915	0.08086119
NM_000676	adenosine A2b receptor (ADORA2B),	1.22701375	0.03825729
NM_030793	F-box protein 38 (FBXO38), transcript variant 1,	1.22614484	0.94670892
NM_005347	heat shock 70kDa protein 5 (glucose-regulated protein, 78kDa) (HSPA5),	1.22609057	0.86216134
NM_015247	cylindromatosis (turban tumor syndrome) (CYLD),	1.225626	0.27184736
NM_002380	matrilin 2 (MATN2), transcript variant 1,	1.22512626	0.30981917
NM_000671	alcohol dehydrogenase 5 (class III), chi polypeptide (ADH5),	1.22501107	0.63351018
AK098129	cDNA FLJ40810 fis, clone TRACH2009743 [AK098129]	1.22491239	1.10823959

NM_018064	chromosome 6 open reading frame 166 (C6orf166),	1.22397075	0.46404155
NM_004180	TRAF family member-associated NFKB activator (TANK), transcript variant 1,	1.22313301	0.10607018
NM_004794	RAB33A, member RAS oncogene family (RAB33A),	1.22238938	0.13176557
NM_006194	paired box gene 9 (PAX9),	1.22178152	0.62987911
NM_033449	FCH and double SH3 domains 1 (FCHSD1),	1.22094441	0.30313247
NM_022755	chromosome 9 open reading frame 12 (C9orf12),	1.22054863	0.74071329
NM_014096	solute carrier family 43, member 3 (SLC43A3),	1.22044427	0.24612349
NM_005426	tumor protein p53 binding protein, 2 (TP53BP2),	1.22003992	0.00386334
NM_181894	glutamate receptor, ionotrophic, AMPA 3 (GRIA3), transcript variant 3,	1.21939695	0.21683916
NM_015683	arrestin domain containing 2 (ARRDC2), transcript variant 1,	1.21890359	0.33890319
NM_177551	G protein-coupled receptor 109A (GPR109A),	1.21871236	0.08984706
NM_030964	sprouty homolog 4 (Drosophila) (SPRY4),	1.21858288	0.80126545
NM_198507	HGS_RE408 (UNQ1912),	1.21832764	0.95395226
NM_014220	transmembrane 4 L six family member 1 (TM4SF1),	1.21827899	0.19531578
NM_003371	vav 2 oncogene (VAV2),	1.21798866	0.18500769
CN641451	Hs.529772	1.21760849	0.25787283
NM_006449	CDC42 effector protein (Rho GTPase binding) 3 (CDC42EP3),	1.21698071	0.74349782
NM_004612	transforming growth factor, beta receptor I (activin A receptor type II-like kinase, 53kDa) (TGFBR1),	1.21630683	0.04029998
NM_001223	caspase 1, apoptosis-related cysteine protease (interleukin 1, beta, convertase) (CASP1), transcript variant beta,	1.21508471	0.06081071
NM_032105	protein phosphatase 1, regulatory (inhibitor) subunit 12B (PPP1R12B), transcript variant 2,	1.21394894	0.21966938
NM_006734	human immunodeficiency virus type I enhancer binding protein 2 (HIVEP2),	1.21322482	0.00145241
NM_004612	transforming growth factor, beta receptor I (activin A receptor type II-like kinase, 53kDa) (TGFBR1),	1.21284159	0.66793501
NM_198552	chromosome 1 open reading frame 153 (C1orf153),	1.21176747	0.34772735
NM_020408	chromosome 6 open reading frame 149 (C6orf149),	1.21112755	0.69380105
NM_018348	hypothetical protein FLJ11171 (FLJ11171),	1.21069737	1.1061784
NM_015517	MBD2 (methyl-CpG-binding protein)-interacting zinc finger protein (MIZF), transcript variant 1,	1.21050416	1.18387225
NM_145023	coiled-coil domain containing 7 (CCDC7),	1.21016172	0.55631369
XR_012394	Macaca mulatta putative small membrane protein NID67 (LOC711300),	1.20976387	0.07150236
NM_033664	cadherin 11, type 2, OB-cadherin (osteoblast) (CDH11), transcript variant 2,	1.20967957	0.06837786
NM_002198	interferon regulatory factor 1 (IRF1),	1.20926486	0.28475911
NM_014622	loss of heterozygosity, 11, chromosomal region 2, gene A (LOH11CR2A), transcript variant 1,	1.20831027	0.02678053
NM_016448	RA-regulated nuclear matrix-associated protein (RAMP),	1.20777605	0.48638734
CK231449	LOC283241	1.20720475	0.04436212
NM_000765	cytochrome P450, family 3, subfamily A, polypeptide 7 (CYP3A7),	1.20631193	0.62606944
CK232102	ILLUMIGEN_MCQ_3292 Katze_MMPL2 Macaca mulatta cDNA 5',	1.20620246	0.11870087
NM_006980	mitochondrial transcription termination factor (MTERF), nuclear gene encoding mitochondrial protein,	1.20586192	1.18025263
NM_021813	BTB and CNC homology 1, basic leucine zipper transcription factor 2 (BACH2),	1.20472107	1.06252918
NM_004905	peroxiredoxin 6 (PRDX6),	1.204325	0.01768575
NM_014622	loss of heterozygosity, 11, chromosomal region 2, gene A (LOH11CR2A), transcript variant 1,	1.20415381	0.15514071
NM_016147	protein phosphatase methylesterase-1 (PME-1),	1.20352277	0.35718301
NM_172037	retinol dehydrogenase 10 (all-trans) (RDH10),	1.20282697	0.15008987
NM_003141	Sjogren syndrome antigen A1 (52kDa, ribonucleoprotein autoantigen SS-A/Ro) (SSA1),	1.20280145	1.03896685
NM_032944	serine/threonine kinase 31 (STK31), transcript variant 2,	1.20223073	0.04094956
NM_147150	A kinase (PRKA) anchor protein 2 (AKAP2), transcript variant 2,	1.2018147	0.68756572
NM_080920	gamma-glutamyltransferase-like activity 4 (GGTLA4), transcript variant C,	1.20169787	0.08046591
NM_004946	dedicator of cytokinesis 2 (DOCK2),	1.20138936	1.01922564

NM_006058	TNFAIP3 interacting protein 1 (TNIP1),	1.20125807	0.00590798
NM_177551	G protein-coupled receptor 109A (GPR109A),	1.20086472	0.15424665
NM_001198	PR domain containing 1, with ZNF domain (PRDM1), transcript variant 1,	1.20072805	0.00335272
NM_001553	insulin-like growth factor binding protein 7 (IGFBP7),	1.20072233	0.26382223
NM_00100189(	runt-related transcription factor 1 (acute myeloid leukemia 1; aml1 oncogene) (RUNX1), transcript variant 2,	1.19977838	0.0638977
NM_006072	chemokine (C-C motif) ligand 26 (CCL26),	1.19941337	0.10798992
NM_015368	pannexin 1 (PANX1),	1.19789628	0.6203514
NM_006769	LIM domain only 4 (LMO4),	1.19772276	0.03605848
NM_001051	somatostatin receptor 3 (SSTR3),	1.19705524	1.03832197
NM_024520	hypothetical protein FLJ22555 (FLJ22555),	1.19699443	0.93148777
XR_009741	Macaca mulatta GRIP and coiled-coil domain-containing 2 isoform a (LOC693729),	1.19654617	0.26770503
NM_001615	actin, gamma 2, smooth muscle, enteric (ACTG2),	1.19554628	0.09910706
XR_013120	Macaca mulatta CHMP family, member 7 (LOC710050),	1.19552518	0.66735887
NM_001040	sex hormone-binding globulin (SHBG),	1.19453204	0.05274857
NM_00100723	HTLV-1 related endogenous sequence (HRES1),	1.19447801	0.00178484
NM_018325	chromosome 9 open reading frame 72 (C9orf72), transcript variant 1,	1.193655	0.15212741
NM_003597	TGFB inducible early growth response 2 (TIEG2),	1.19188434	0.11061205
NM_004457	acyl-CoA synthetase long-chain family member 3 (ACSL3), transcript variant 1,	1.19159056	0.14556803
NM_00100152	UDP-glucose pyrophosphorylase 2 (UGP2), transcript variant 2,	1.19157771	0.00148979
CN641451	Hs.529772	1.19092066	0.1446049
NM_003851	cellular repressor of E1A-stimulated genes (CREG),	1.19068379	0.04021089
NM_003486	solute carrier family 7 (cationic amino acid transporter, y+ system), member 5 (SLC7A5),	1.19062792	0.13710562
NM_022566	mesoderm development candidate 1 (MESDC1),	1.18930239	0.3482164
NM_018248	nei endonuclease VIII-like 3 (E. coli) (NEIL3),	1.18910547	0.61300067
NM_002570	proprotein convertase subtilisin/kexin type 6 (PCSK6), transcript variant 1,	1.18811604	0.76989191
NM_004419	dual specificity phosphatase 5 (DUSP5),	1.18807768	0.00196444
NM_006769	LIM domain only 4 (LMO4),	1.18803585	0.01549799
NM_020299	aldo-keto reductase family 1, member B10 (aldose reductase) (AKR1B10),	1.18795535	0.09500812
NM_021194	solute carrier family 30 (zinc transporter), member 1 (SLC30A1),	1.18624301	0.1081526
NM_032021	AD031 protein (AD031),	1.18577645	0.37482567
AK000672	cDNA FLJ20665 fis, clone KAIA713, highly AF151848 CGI-90 protein	1.18495902	0.27334017
NM_005614	Ras homolog enriched in brain (RHEB),	1.18460218	0.10182507
NM_194278	chromosome 14 open reading frame 43 (C14orf43),	1.18302615	0.80377772
NM_014220	transmembrane 4 L six family member 1 (TM4SF1),	1.18288212	0.0144918
XM_375359	brain expressed, associated with Nedd4 (BEAN),	1.182564	0.0376556
NM_152277	dendritic cell-derived ubiquitin-like protein (DC-UbP),	1.18243307	0.62406281
NM_016303	WW domain binding protein 1 (WBP5),	1.18156142	0.16551244
NM_024873	TNFAIP3 interacting protein 3 (TNIP3),	1.17909476	0.29923227
NM_000311	prion protein (p27-30) (Creutzfeld-Jakob disease, Gerstmann-Strausler-Scheinker syndrome, fatal familial insomnia) (PRNP), transcript variant 1,	1.17866718	0.04336483
NM_001099	acid phosphatase, prostate (ACPP),	1.17833749	1.09660131
NM_006291	tumor necrosis factor, alpha-induced protein 2 (TNFAIP2),	1.17787287	0.03156921
XR_012351	Macaca mulatta riboflavin kinase (LOC704540),	1.17766602	0.20309752
NM_016230	NADPH cytochrome B5 oxidoreductase (NCB5OR),	1.17757759	0.07849799
NM_004265	fatty acid desaturase 2 (FADS2),	1.17755838	0.115407
NM_000628	interleukin 10 receptor, beta (IL10RB),	1.1773642	0.08969708

NM_002827	protein tyrosine phosphatase, non-receptor type 1 (PTPN1),	1.17724323	0.19015316
NM_032427	mastermind-like 2 (Drosophila) (MAML2),	1.17696807	0.41230603
NM_002984	chemokine (C-C motif) ligand 4 (CCL4),	1.1759401	0.19801708
NM_145649	glucosaminyl (N-acetyl) transferase 2, I-branching enzyme (GCNT2), transcript variant 1,	1.1757994	0.18591974
NM_030627	cytoplasmic polyadenylation element binding protein 4 (CPEB4),	1.17573066	0.22269644
NM_007194	CHK2 checkpoint homolog (S. pombe) (CHEK2), transcript variant 1,	1.17550625	0.04005881
NM_153207	AE binding protein 2 (AEBP2),	1.17505347	0.39294769
AK098129	cDNA FLJ40810 fis, clone TRACH2009743 [AK098129]	1.17368799	1.04582207
NM_020429	SMAD specific E3 ubiquitin protein ligase 1 (SMURF1), transcript variant 1,	1.17319931	0.50855321
NM_006948	stress 70 protein chaperone, microsome-associated, 60kDa (STCH),	1.17277525	0.20367254
NM_015173	TBC1 (tre-2/USP6, BUB2, cdc16) domain family, member 1 (TBC1D1),	1.17245315	0.00441237
NM_198941	tumor differentially expressed 1 (TDE1), transcript variant 2,	1.17227317	0.62174461
NM_002527	neurotrophin 3 (NTF3),	1.17192401	0.24896507
NM_015288	PHD finger protein 15 (PHF15),	1.17175242	0.05431436
NM_032857	lactamase, beta (LACTB), nuclear gene encoding mitochondrial protein, transcript variant 1,	1.17157788	0.10653657
NM_138341	hypothetical protein BC000282 (LOC89894),	1.17066522	0.04685004
NM_004180	TRAF family member-associated NFKB activator (TANK), transcript variant 1,	1.17045701	0.01124307
NM_017631	hypothetical protein FLJ20035 (FLJ20035),	1.16960821	0.30237325
NM_013345	G protein-coupled receptor 132 (GPR132),	1.16947054	0.35896001
NM_000965	retinoic acid receptor, beta (RARB), transcript variant 1,	1.16918782	0.06937807
NM_145284	hypothetical protein MGC17347 (LOC159090),	1.16916744	0.1261703
NM_014801	pecanex-like 2 (Drosophila) (PCNXL2), transcript variant 1,	1.16828848	0.1300075
NM_004079	cathepsin S (CTSS),	1.16797858	0.0115887
NM_003383	very low density lipoprotein receptor (VLDLR),	1.16757472	0.19048943
NM_144587	chromosome 10 open reading frame 87 (C10orf87),	1.16736034	0.29752638
XR_012436	Macaca mulatta mannosidase, alpha, class 2A, member 1 (LOC705480),	1.16676017	0.01816495
NM_024420	phospholipase A2, group IVA (cytosolic, calcium-dependent) (PLA2G4A),	1.16483094	0.17263062
NM_013310	chromosome 2 open reading frame 27 (C2orf27),	1.16422452	0.08775761
NM_001186	BTB and CNC homology 1, basic leucine zipper transcription factor 1 (BACH1), transcript variant 2,	1.16394703	0.18865511
NM_025019	tubulin, alpha 4 (TUBA4),	1.16342152	0.86270765
NM_198552	chromosome 1 open reading frame 153 (C1orf153),	1.16255021	0.5498641
NM_003241	transglutaminase 4 (prostate) (TGM4),	1.16243356	0.56924082
NM_017413	apelin, AGTRL1 ligand (APLN),	1.16232542	0.09624871
BE257326	601108540F1 NIH_MGC_16 cDNA clone IMAGE:3344889 5',	1.1616462	0.25693118
CN646100	Hs.529393	1.16117161	0.57449953
NM_052941	guanylate binding protein 4 (GBP4),	1.16106449	0.09228131
NM_002380	matrilin 2 (MATN2), transcript variant 1,	1.16075909	0.44983587
NM_002984	chemokine (C-C motif) ligand 4 (CCL4),	1.15958982	0.16905448
CK231449	LOC283241	1.15950009	0.09252869
NM_031442	transmembrane 4 superfamily member 10 (TM4SF10),	1.15943706	0.00896041
NM_014701	KIAA0256 gene product (KIAA0256),	1.15920235	0.07301539
NM_003528	histone 2, H2be (HIST2H2BE),	1.15896395	0.01716568
XM_044461	KIAA1102 protein (KIAA1102),	1.15869852	0.06131583
NM_020474	UDP-N-acetyl-alpha-D-galactosamine:polypeptide N-acetylgalactosaminyltransferase 1 (GalNAc-T1) (GALNT1),	1.15789512	0.04480386
NM_001615	actin, gamma 2, smooth muscle, enteric (ACTG2),	1.15783094	0.09656978

NM_006378	sema domain, immunoglobulin domain (Ig), transmembrane domain (TM) and short cytoplasmic domain, (semaphorin) 4D (SEMA4D),	1.15780928	0.04962109
NM_025147	hypothetical protein FLJ13448 (FLJ13448),	1.15743485	0.05518299
NM_003851	cellular repressor of E1A-stimulated genes (CREG),	1.15594617	0.25430029
NM_016206	vestigial-like 3 (VGL-3),	1.15514509	0.13164778
NM_002835	protein tyrosine phosphatase, non-receptor type 12 (PTPN12),	1.15448491	0.01351964
NM_033664	cadherin 11, type 2, OB-cadherin (osteoblast) (CDH11), transcript variant 2,	1.15439521	0.13074385
NM_173457	phosphodiesterase 8A (PDE8A), transcript variant 5,	1.1539262	0.0971147
NM_000176	nuclear receptor subfamily 3, group C, member 1 (glucocorticoid receptor) (NR3C1),	1.15309592	0.12696737
NM_000633	B-cell CLL/lymphoma 2 (BCL2), nuclear gene encoding mitochondrial protein, transcript variant alpha,	1.15163723	0.48790336
NM_019080	Nedd4 family interacting protein 2 (NDFIP2),	1.15083835	0.02328139
NM_005426	tumor protein p53 binding protein, 2 (TP53BP2),	1.15083109	0.00680277
NM_002800	proteasome (prosome, macropain) subunit, beta type, 9 (large multifunctional protease 2) (PSMB9), transcript variant 1,	1.15046977	0.06988392
NM_156036	homeo box B6 (HOXB6), transcript variant 3,	1.14985742	0.78055884
CN641451	Hs.529772	1.1496836	0.0469387
NM_00100475[illegible]	olfactory receptor, family 51, subfamily F, member 2 (OR51F2),	1.1489739	0.09937266
NM_003242	transforming growth factor, beta receptor II (70/80kDa) (TGFBR2),	1.14829687	0.01421142
NM_206998	secretoglobin family 1D member 4 (SCGB1D4),	1.14800743	0.94476246
NM_013421	gamma-glutamyltransferase 1 (GGT1), transcript variant 2,	1.14773387	0.27156131
NM_003879	CASP8 and FADD-like apoptosis regulator (CFLAR),	1.14767624	0.45697691
NM_016205	platelet derived growth factor C (PDGFC),	1.14754761	0.10570843
NM_182972	interferon regulatory factor 2 binding protein 2 (IRF2BP2),	1.14685103	0.51363878
NM_144600	hypothetical protein FLJ31153 (FLJ31153),	1.14618179	0.00301308
NM_016445	pleckstrin 2 (PLEK2),	1.14612577	0.04615748
NM_006729	diaphanous homolog 2 (Drosophila) (DIAPH2), transcript variant 156,	1.14573646	0.05239829
CN644550	TGIF	1.14530562	0.12493252
NM_020474	UDP-N-acetyl-alpha-D-galactosamine:polypeptide N-acetylgalactosaminyltransferase 1 (GalNAc-T1) (GALNT1),	1.14476723	0.09163861
NM_004098	empty spiracles homolog 2 (Drosophila) (EMX2),	1.14418412	0.41500727
NM_000311	prion protein (p27-30) (Creutzfeld-Jakob disease, Gerstmann-Strausler-Scheinker syndrome, fatal familial insomnia) (PRNP), transcript variant 1,	1.14387715	0.0391588
NM_00100171[illegible]	nuclear factor of kappa light polypeptide gene enhancer in B-cells inhibitor, beta (NFKBIB), transcript variant 2,	1.14291919	0.29806546
CN802384	C14orf147	1.14291424	0.04104472
NM_004794	RAB33A, member RAS oncogene family (RAB33A),	1.14262946	0.39008713
NM_018849	ATP-binding cassette, sub-family B (MDR/TAP), member 4 (ABCB4), transcript variant B,	1.14195583	0.01202842
XR_012825	Macaca mulatta early B-cell factor 3 (LOC713536),	1.14002387	0.00476938
NM_138341	hypothetical protein BC000282 (LOC89894),	1.13962485	0.33020854
XM_044461	KIAA1102 protein (KIAA1102),	1.13920057	0.11043134
NM_002842	protein tyrosine phosphatase, receptor type, H (PTPRH),	1.13912548	0.5349414
NM_002163	interferon regulatory factor 8 (IRF8),	1.13872089	0.20376826
NM_020215	chromosome 14 open reading frame 132 (C14orf132),	1.1375795	0.0702131
XR_010175	Macaca mulatta potassium channel tetramerisation domain containing 9 (LOC696565),	1.13757861	0.1509073
NM_007199	interleukin-1 receptor-associated kinase 3 (IRAK3),	1.1374559	0.60505145
XM_371832	KIAA1411 (KIAA1411),	1.13686473	0.19324387
NM_001391	dystrobrevin, alpha (DTNA), transcript variant 3,	1.13669129	0.12543965
XR_010798	Macaca mulatta endothelial differentiation, sphingolipid G-protein-coupled receptor, 3 (LOC700903),	1.13636893	0.70906817

NM_00100141!	zinc finger protein 429 (ZNF429),	1.13611751	0.82419914
NM_004556	nuclear factor of kappa light polypeptide gene enhancer in B-cells inhibitor, epsilon (NFKBIE),	1.13569944	0.04737781
NM_006948	stress 70 protein chaperone, microsome-associated, 60kDa (STCH),	1.13442159	0.21504767
NM_152716	hypothetical protein FLJ36874 (FLJ36874),	1.13432899	0.86218095
NM_032427	mastermind-like 2 (Drosophila) (MAML2),	1.13345603	0.68992028
NM_004708	programmed cell death 5 (PDCD5),	1.13206904	0.0109806
XR_012325	Macaca mulatta hypothetical protein LOC710960 (LOC710960),	1.13173147	0.04977664
NM_00100839·	E1A-like inhibitor of differentiation 3 (EID3),	1.13168889	0.4933772
NM_152680	hypothetical protein FLJ32028 (FLJ32028),	1.1311791	0.05096019
NM_001034	ribonucleotide reductase M2 polypeptide (RRM2),	1.13103922	0.85622978
NM_024910	hypothetical protein FLJ12700 (FLJ12700),	1.1309851	0.96883568
NM_020299	aldo-keto reductase family 1, member B10 (aldose reductase) (AKR1B10),	1.12960677	0.11416364
NM_198566	FLJ32363 protein (FLJ32363),	1.12844699	0.47774418
NM_031904	FKSG44 gene (FKSG44),	1.12840139	0.40267758
CN641390	Hs.405253	1.12817099	0.07931291
CN643443	DKFZP566B183	1.12733384	0.02672111
NM_173573	hypothetical protein MGC35138 (MGC35138),	1.12663779	0.08616486
NM_013957	neuregulin 1 (NRG1), transcript variant HRG-beta2,	1.12659081	0.91797924
NM_020300	microsomal glutathione S-transferase 1 (MGST1), transcript variant 1b,	1.12634133	0.18014617
NM_004086	coagulation factor C homolog, cochlin (Limulus polyphemus) (COCH),	1.12604213	0.52015315
NM_207322	hypothetical LOC145741 (LOC145741),	1.12577127	0.00676948
NM_033211	hypothetical gene supported by AF038182; BC009203 (LOC90355),	1.12424221	0.29172797
NM_024079	asparagine-linked glycosylation 8 homolog (yeast, alpha-1,3-glucosyltransferase) (ALG8),	1.12341403	0.7252941
NM_005460	synuclein, alpha interacting protein (synphilin) (SNCAIP),	1.12323703	0.66446728
NM_022748	tensin-like SH2 domain containing 1 (TENS1),	1.12194946	0.22978086
CB311570	AGENCOURT_11850920 NICHD_Rh_Ov1 Macaca mulatta cDNA clone IMAGE:6912905 5',	1.12185745	0.07383495
NM_080839	gamma-glutamyltransferase-like 4 (GGTL4), transcript variant 2,	1.12143536	0.10730635
NM_015186	vacuolar protein sorting 13A (yeast) (VPS13A), transcript variant B,	1.12142091	0.11568977
CO725462	VAMP5	1.12113706	0.08814182
NM_004686	myotubularin related protein 7 (MTMR7),	1.12112183	0.39733183
NM_025144	alpha-kinase 1 (ALPK1),	1.12081294	0.2657692
NM_001766	CD1D antigen, d polypeptide (CD1D),	1.12073078	0.06181794
NM_033116	NIMA (never in mitosis gene a)- related kinase 9 (NEK9),	1.12004955	0.25417809
NM_004708	programmed cell death 5 (PDCD5),	1.11995884	0.08196008
NM_017947	molybdenum cofactor sulfurase (MOCOS),	1.11904107	0.46692759
NM_001172	arginase, type II (ARG2), nuclear gene encoding mitochondrial protein,	1.11897293	0.58973248
NM_170721	musashi homolog 2 (Drosophila) (MSI2), transcript variant 2,	1.11784863	0.73785402
NM_001470	gamma-aminobutyric acid (GABA) B receptor, 1 (GABBR1), transcript variant 1,	1.11704744	0.10565585
NM_005565	lymphocyte cytosolic protein 2 (SH2 domain containing leukocyte protein of 76kDa) (LCP2),	1.11695914	0.38158306
NM_156036	homeo box B6 (HOXB6), transcript variant 3,	1.11677364	0.16727527
NM_012319	solute carrier family 39 (zinc transporter), member 6 (SLC39A6),	1.11650889	0.41490877
NM_052969	ribosomal protein L39-like (RPL39L),	1.11563755	0.32318222
NM_005806	oligodendrocyte lineage transcription factor 2 (OLIG2),	1.11548925	0.23769679
NM_001955	endothelin 1 (EDN1),	1.11500838	0.20509299
NM_152448	hypothetical protein MGC33951 (MGC33951),	1.11479278	0.20393208
XR_011459	Macaca mulatta enabled homolog isoform b (LOC700559),	1.11417626	0.52778937

NM_012243	solute carrier family 35 (UDP-N-acetylglucosamine (UDP-GlcNAc) transporter), member A3 (SLC35A3),	1.11412611	0.85008961
NM_006516	solute carrier family 2 (facilitated glucose transporter), member 1 (SLC2A1),	1.11371663	0.16176761
NM_001257	cadherin 13, H-cadherin (heart) (CDH13),	1.11351166	0.0798892
NM_033045	keratin, hair, basic, 4 (KRTHB4),	1.1130369	0.32351065
NM_015404	deafness, autosomal recessive 31 (DFNB31),	1.11225765	0.28580138
NM_014801	pecanex-like 2 (Drosophila) (PCNXL2), transcript variant 1,	1.11218581	0.05749567
NM_004556	nuclear factor of kappa light polypeptide gene enhancer in B-cells inhibitor, epsilon (NFKBIE),	1.110918	0.01790631
NM_006219	phosphoinositide-3-kinase, catalytic, beta polypeptide (PIK3CB),	1.11044526	0.43923762
NM_139004	hemochromatosis (HFE), transcript variant 4,	1.11001382	0.60626494
XM_497748	Olfactory receptor 13G1 (LOC441933),	1.10855459	0.252742
NM_005715	uronyl-2-sulfotransferase (UST),	1.10844302	0.38362444
NM_205853	musculoskeletal, embryonic nuclear protein 1 (MUSTN1),	1.10765607	0.17764305
NM_015047	KIAA0090 protein (KIAA0090),	1.10714625	0.25042994
XR_011208	Macaca mulatta protein tyrosine phosphatase, receptor type, G precursor (LOC703937),	1.10695341	0.00057742
XR_012351	Macaca mulatta riboflavin kinase (LOC704540),	1.10614684	0.03071359
XM_372199	FLJ16518 protein (FLJ16518),	1.10614313	0.27925843
NM_013962	neuregulin 1 (NRG1), transcript variant GGF2,	1.10505137	0.1550788
NM_001470	gamma-aminobutyric acid (GABA) B receptor, 1 (GABBR1), transcript variant 1,	1.10489164	0.16251832
NM_022572	myofibrillogenesis regulator 1 (MR-1),	1.10481644	0.36453216
NM_181706	zinc finger, CSL-type containing 3 (ZCSL3),	1.10477412	0.04305801
NM_002182	interleukin 1 receptor accessory protein (IL1RAP), transcript variant 1,	1.10414676	0.67966109
NM_004731	solute carrier family 16 (monocarboxylic acid transporters), member 7 (SLC16A7),	1.1040854	0.38903646
NM_014782	armadillo repeat containing, X-linked 2 (ARMCX2),	1.10401523	0.25546377
XR_013986	Macaca mulatta hypothetical protein LOC718705 (LOC718705),	1.10382259	0.53103908
NM_006950	synapsin I (SYN1), transcript variant Ia,	1.10381738	0.53885695
NM_015571	SUMO1/sentrin specific protease 6 (SENP6),	1.10363895	1.06748085
NM_004497	forkhead box A3 (FOXA3),	1.10298387	0.48082746
NM_00100753[illegible]	transmembrane protein 46 (TMEM46),	1.10234618	0.00557439
NM_006729	diaphanous homolog 2 (Drosophila) (DIAPH2), transcript variant 156,	1.10229539	0.10269001
NM_001553	insulin-like growth factor binding protein 7 (IGFBP7),	1.10215048	0.25759167
NM_033087	asparagine-linked glycosylation 2 homolog (yeast, alpha-1,3-mannosyltransferase) (ALG2), transcript variant 1,	1.10162938	0.01826259
NM_000210	integrin, alpha 6 (ITGA6),	1.10158327	0.06900058
NM_016354	solute carrier organic anion transporter family, member 4A1 (SLCO4A1),	1.10147698	0.0750876
NM_016301	protein x 0004 (MGC14560),	1.10146363	0.23487427
NM_002032	ferritin, heavy polypeptide 1 (FTH1),	1.10112805	0.49268841
NM_016546	complement component 1, r subcomponent-like (C1RL),	1.10112177	0.22622354
NM_006602	transcription factor-like 5 (basic helix-loop-helix) (TCFL5),	1.10065175	0.6096409
NM_002844	protein tyrosine phosphatase, receptor type, K (PTPRK),	1.10063759	0.02662252
NM_000249	mutL homolog 1, colon cancer, nonpolyposis type 2 (E. coli) (MLH1),	1.09942711	0.95977231
NM_004265	fatty acid desaturase 2 (FADS2),	1.09912763	0.13521141
NM_032549	IMP2 inner mitochondrial membrane protease-like (S. cerevisiae) (IMMP2L),	1.09900612	0.06386773
NM_018267	H2A histone family, member J (H2AFJ), transcript variant 1,	1.0986273	0.23379256
XR_012436	Macaca mulatta mannosidase, alpha, class 2A, member 1 (LOC705480),	1.09832183	0.20779598
NM_001924	growth arrest and DNA-damage-inducible, alpha (GADD45A),	1.09789971	0.20085057
XR_010566	Macaca mulatta GrpE protein homolog 2, mitochondrial precursor (Mt-GrpE#2) (LOC699299),	1.09756691	0.37986491
NM_022377	intercellular adhesion molecule 4, Landsteiner-Wiener blood group (ICAM4), transcript variant 2,	1.09713146	0.24240288

NM_004079	cathepsin S (CTSS),	1.09656697	0.18078111
NM_001197	BCL2-interacting killer (apoptosis-inducing) (BIK),	1.09604877	0.35877211
NM_000408	glycerol-3-phosphate dehydrogenase 2 (mitochondrial) (GPD2),	1.09593646	0.20367321
NM_014923	fibronectin type III domain containing 3 (FNDC3),	1.09439799	0.69499242
NM_207322	nuclear localized factor 1 (NLF1),	1.0939885	0.42959542
NM_013246	cardiotrophin-like cytokine (CLC),	1.09349127	0.28682227
NM_020122	potassium channel modulatory factor 1 (KCMF1),	1.09274751	0.1713267
NM_013421	gamma-glutamyltransferase 1 (GGT1), transcript variant 2,	1.09274116	0.04477755
NM_003056	solute carrier family 19 (folate transporter), member 1 (SLC19A1), transcript variant 1,	1.09273656	0.84788584
NM_017817	RAB20, member RAS oncogene family (RAB20),	1.09235253	0.03827677
NM_00100189(	runt-related transcription factor 1 (acute myeloid leukemia 1; aml1 oncogene) (RUNX1), transcript variant 2,	1.0921139	0.14339251
NM_015885	pre-	1.0907032	0.32271936
NM_177551	G protein-coupled receptor 109A (GPR109A),	1.09067261	0.1580278
NM_145686	mitogen-activated protein kinase kinase kinase kinase 4 (MAP4K4), transcript variant 2,	1.09055914	0.05334065
NM_033505	selenoprotein I (SELI),	1.09014868	0.09790857
NM_000693	aldehyde dehydrogenase 1 family, member A3 (ALDH1A3),	1.08979893	0.0829197
NM_021965	phosphoglucomutase 5 (PGM5),	1.0892368	0.20126966
NM_013961	neuregulin 1 (NRG1), transcript variant GGF,	1.08914605	0.04714989
NM_174907	protein phosphatase 4, regulatory subunit 2 (PPP4R2),	1.0891455	0.35550783
NM_018947	cytochrome c, somatic (CYCS), nuclear gene encoding mitochondrial protein,	1.08912673	0.0016681
NM_018950	major histocompatibility complex, class I, F (HLA-F),	1.08863137	0.10877256
NM_000101	cytochrome b-245, alpha polypeptide (CYBA),	1.08783828	0.18284724
CK231677	FGF1	1.08746972	0.57326677
CN802384	C14orf147	1.08738934	0.03727284
NM_052969	ribosomal protein L39-like (RPL39L),	1.08719025	0.18846236
NM_022843	protocadherin 20 (PCDH20),	1.08708322	0.00673011
NM_013382	protein-O-mannosyltransferase 2 (POMT2),	1.08705582	0.00253535
NM_001924	growth arrest and DNA-damage-inducible, alpha (GADD45A),	1.08623984	0.02928312
NM_006167	NK3 transcription factor related, locus 1 (Drosophila) (NKX3-1),	1.08578349	0.10369878
NM_003020	secretory granule, neuroendocrine protein 1 (7B2 protein) (SGNE1),	1.08577896	0.96372937
NM_002892	AT rich interactive domain 4A (RBP1-like) (ARID4A), transcript variant 1,	1.08547944	0.85277521
XR_010622	Macaca mulatta polycomb group ring finger 5 (LOC697020),	1.08535992	0.43695949
NM_003580	neutral sphingomyelinase (N-SMase) activation associated factor (NSMAF),	1.08524379	0.03914431
NM_001912	cathepsin L (CTSL), transcript variant 1,	1.08519869	0.26360086
XM_290546	KIAA0830 protein (KIAA0830),	1.08466886	0.42426317
NM_021642	Fc fragment of IgG, low affinity IIa, receptor (CD32) (FCGR2A),	1.08403819	0.06223191
NM_025168	leucine rich repeat containing 1 (LRRC1),	1.08271123	0.89988736
NM_001949	E2F transcription factor 3 (E2F3),	1.08269737	0.04954459
CB309570	AGENCOURT_11830463 NICHD_Rh_Ov1 Macaca mulatta cDNA clone IMAGE:6916521 5',	1.08224135	0.3458077
NM_019601	sushi domain containing 2 (SUSD2),	1.08222462	0.24887734
NM_000681	adrenergic, alpha-2A-, receptor (ADRA2A),	1.0821504	0.35135052
NM_012154	eukaryotic translation initiation factor 2C, 2 (EIF2C2),	1.0821395	0.68962631
CN643325	TBCC	1.0806004	0.64208451
NM_032505	T-cell activation kelch repeat protein (TA-KRP),	1.0796097	0.09463448
XR_010654	Macaca mulatta acyl-CoA synthetase long-chain family member 5 isoform a (LOC696404),	1.07952002	0.29348971
NM_016040	CGI-100 protein (CGI-100),	1.07914952	0.53918397

NM_00100821:	optineurin (OPTN), transcript variant 1,	1.07906329	0.10223583
NM_015384	Nipped-B homolog (Drosophila) (NIPBL), transcript variant B,	1.07880798	0.69082148
NM_002197	aconitase 1, soluble (ACO1),	1.07809856	0.07397067
NM_175736	formin-like 3 (FMNL3), transcript variant 1,	1.07659409	0.89882625
XR_012241	Macaca mulatta sulfatase 1 (SULF1),	1.0755603	0.59245745
NM_014683	unc-51-like kinase 2 (C. elegans) (ULK2),	1.07554922	0.06661144
NM_016303	WW domain binding protein 1 (WBP5),	1.07551546	0.16095595
NM_002193	inhibin, beta B (activin AB beta polypeptide) (INHBB),	1.07546428	0.19455886
AW014767	UI-H-BI0-aae-f-12-0-UI.s1 NCI_CGAP_Sub1 cDNA clone IMAGE:2709262 3',	1.07493077	0.22270383
NM_004615	transmembrane 4 superfamily member 2 (TM4SF2),	1.07446126	0.13291063
NM_199511	steroid sensitive gene 1 (URB), transcript variant 1,	1.07427369	0.26602997
NM_017849	hypothetical protein FLJ20507 (FLJ20507),	1.07382935	0.10825203
NM_153355	T-cell lymphoma breakpoint associated target 1 (TCBA1),	1.07365708	0.00466732
NM_003612	sema domain, immunoglobulin domain (Ig), and GPI membrane anchor, (semaphorin) 7A (SEMA7A),	1.07278036	0.2434419
NM_198700	CUG triplet repeat, RNA binding protein 1 (CUGBP1), transcript variant 2,	1.07268749	0.48793565
NM_181713	UBX domain containing 4 (UBXD4),	1.07262461	0.19491745
NM_013252	C-type lectin domain family 5, member A (CLEC5A),	1.07186942	0.47764053
NM_000628	interleukin 10 receptor, beta (IL10RB),	1.07087751	0.01554015
XR_012582	Macaca mulatta retinoic acid receptor responder (tazarotene induced) 1 isoform 1 (LOC703781),	1.07039078	0.09792182
CO725462	VAMP5	1.06945406	0.04411993
NM_012328	DnaJ (Hsp40) homolog, subfamily B, member 9 (DNAJB9),	1.06910895	0.01820854
NM_00100753ε	transmembrane protein 46 (TMEM46),	1.06908202	0.06375913
NM_003131	serum response factor (c-fos serum response element-binding transcription factor) (SRF),	1.0690464	0.06593182
NM_001254	CDC6 cell division cycle 6 homolog (S. cerevisiae) (CDC6),	1.06896562	0.21879073
NM_021038	muscleblind-like (Drosophila) (MBNL1), transcript variant 1,	1.06822354	0.06482701
NM_000786	cytochrome P450, family 51, subfamily A, polypeptide 1 (CYP51A1),	1.06821719	0.25350104
NM_004457	acyl-CoA synthetase long-chain family member 3 (ACSL3), transcript variant 1,	1.06811477	0.14070009
BC018929	pleckstrin homology-like domain, family A, member 1,	1.0669372	0.17203695
AK098129	cDNA FLJ40810 fis, clone TRACH2009743 [AK098129]	1.06669299	0.18562769
NM_006150	LIM domain only 6 (LMO6),	1.06655807	0.61439054
NM_022068	family with sequence similarity 38, member B (FAM38B),	1.06617817	0.05023377
NM_001505	G protein-coupled receptor 30 (GPR30),	1.06555025	0.09841515
NM_00100535:	adenylate kinase 3-like 1 (AK3L1), nuclear gene encoding mitochondrial protein, transcript variant 1,	1.0650927	0.14196135
NM_002669	pleiotropic regulator 1 (PRL1homolog, Arabidopsis) (PLRG1),	1.06408503	0.37438072
NM_020156	core 1 UDP-galactose:N-acetylgalactosamine-alpha-R beta 1,3-galactosyltransferase (C1GALT1),	1.06379198	0.09192704
NM_021238	family with sequence similarity 60, member A (FAM60A),	1.06299956	0.27086732
NM_004441	EPH receptor B1 (EPHB1),	1.06282028	0.02246696
NM_144778	muscleblind-like 2 (Drosophila) (MBNL2), transcript variant 1,	1.06228913	0.18702754
NM_022571	G protein-coupled receptor 135 (GPR135),	1.06219098	0.01382828
NM_002584	paired box gene 7 (PAX7), transcript variant 1,	1.06157613	0.04111447
NM_181723	EF hand domain family, member A2 (EFHA2),	1.06132555	0.10134892
NM_005806	oligodendrocyte lineage transcription factor 2 (OLIG2),	1.06064673	0.38005704
NM_183040	dystrobrevin binding protein 1 (DTNBP1), transcript variant 2,	1.06047852	0.66900311
XR_010784	Macaca mulatta platelet-derived growth factor receptor alpha (PDGFRA),	1.06039181	0.01643094
NM_017813	hypothetical protein FLJ20421 (FLJ20421),	1.06037897	0.06040343
NM_007361	nidogen 2 (osteonidogen) (NID2),	1.05974802	0.0911886

NM_004612	transforming growth factor, beta receptor I (activin A receptor type II-like kinase, 53kDa) (TGFBR1),	1.05970107	0.14146059
NM_198951	transglutaminase 2 (C polypeptide, protein-glutamine-gamma-glutamyltransferase) (TGM2), transcript variant 2,	1.05905595	0.33203811
NM_033213	zinc finger protein 670 (ZNF670),	1.05897479	0.55680703
NM_013254	TANK-binding kinase 1 (TBK1),	1.05865563	0.28970201
NM_173457	phosphodiesterase 8A (PDE8A), transcript variant 5,	1.05759748	0.09494829
NM_152999	six transmembrane epithelial antigen of the prostate 2 (STEAP2),	1.05738835	0.30917599
NM_152400	hypothetical protein FLJ39370 (FLJ39370),	1.0568333	0.91805437
NM_014923	fibronectin type III domain containing 3 (FNDC3),	1.05637918	0.22632453
NM_177424	syntaxin 12 (STX12),	1.05619078	0.1992806
NM_000898	monoamine oxidase B (MAOB), nuclear gene encoding mitochondrial protein,	1.05582143	0.11479802
NM_003778	UDP-Gal:betaGlcNAc beta 1,4- galactosyltransferase, polypeptide 4 (B4GALT4), transcript variant 2,	1.05481706	0.08063237
NM_017938	hypothetical protein FLJ20716 (FLJ20716),	1.05460751	0.05716886
CN644557	PDIR	1.05422241	0.27448941
NM_005327	L-3-hydroxyacyl-Coenzyme A dehydrogenase, short chain (HADHSC),	1.05417382	0.00388756
NM_052889	CARD only protein (COPI), transcript variant 2,	1.05330682	0.01664099
NM_006744	retinol binding protein 4, plasma (RBP4),	1.05230072	0.16747549
NM_198141	glucosidase, alpha; neutral C (GANC),	1.05220395	0.52039131
NM_004454	ets variant gene 5 (ets-related molecule) (ETV5),	1.05193132	0.00246326
NM_022748	tensin-like SH2 domain containing 1 (TENS1),	1.05172662	0.10269746
BC107879	sterol-C4-methyl oxidase-like,	1.05152442	0.077486
NM_213589	Ras association (RalGDS/AF-6) and pleckstrin homology domains 1 (RAPH1), transcript variant 1,	1.04991059	0.66782473
NM_000965	retinoic acid receptor, beta (RARB), transcript variant 1,	1.0495289	0.08914836
NM_005729	peptidylprolyl isomerase F (cyclophilin F) (PPIF), nuclear gene encoding mitochondrial protein,	1.04943381	0.08046322
NM_015213	RAB6 interacting protein 1 (RAB6IP1),	1.04870774	0.15423177
NM_003272	transmembrane 7 superfamily member 1 (upregulated in kidney) (TM7SF1),	1.04819286	0.38726566
NM_000261	myocilin, trabecular meshwork inducible glucocorticoid response (MYOC),	1.04761372	0.08142548
NM_001949	E2F transcription factor 3 (E2F3),	1.04743371	0.05571112
NM_021642	Fc fragment of IgG, low affinity IIa, receptor (CD32) (FCGR2A),	1.0456995	0.06377931
NM_005755	Epstein-Barr virus induced gene 3 (EBI3),	1.04559363	0.12390372
NM_003580	neutral sphingomyelinase (N-SMase) activation associated factor (NSMAF),	1.04433777	0.21636094
NM_020925	von Willebrand factor type A and cache domain containing 1 (VWCD1),	1.04324908	0.03096114
NM_031892	SH3-domain kinase binding protein 1 (SH3KBP1),	1.04323234	0.20803643
NM_025040	zinc finger protein 614 (ZNF614),	1.0427998	0.57374553
NM_152829	testis derived transcript (3 LIM domains) (TES), transcript variant 2,	1.04222504	0.16520817
NM_005565	lymphocyte cytosolic protein 2 (SH2 domain containing leukocyte protein of 76kDa) (LCP2),	1.04206536	0.05226947
NM_006558	KH domain containing, RNA binding, signal transduction associated 3 (KHDRBS3),	1.04162963	0.0869432
NM_012323	v-maf musculoaponeurotic fibrosarcoma oncogene homolog F (avian) (MAFF), transcript variant 1,	1.04126893	0.08400804
NM_002068	guanine nucleotide binding protein (G protein), alpha 15 (Gq class) (GNA15),	1.04076581	0.15190224
NM_032834	asparagine-linked glycosylation 10 homolog (yeast, alpha-1,2-glucosyltransferase) (ALG10),	1.04041547	0.62668342
NM_001186	BTB and CNC homology 1, basic leucine zipper transcription factor 1 (BACH1), transcript variant 2,	1.04008597	0.11915298
NM_022770	hypothetical protein FLJ13912 (FLJ13912),	1.03998973	0.01734826
XM_290704	hypothetical protein FLJ12270 (FLJ12270),	1.03971612	1.00639039
XR_010784	Macaca mulatta platelet-derived growth factor receptor alpha (PDGFRA),	1.03832506	0.19653434
NM_005693	nuclear receptor subfamily 1, group H, member 3 (NR1H3),	1.03764922	0.89693127
XM_376059	SERTA domain containing 2 (SERTAD2),	1.03713883	0.00481241
NM_001419	ELAV (embryonic lethal, abnormal vision, Drosophila)-like 1 (Hu antigen R) (ELAVL1),	1.03712129	0.8437339

NM_017870	heat shock 70kDa protein 5 (glucose-regulated protein, 78kDa) binding protein 1 (HSPA5BP1), transcript variant 1,	1.03666815	0.10057966
NM_033550	TP53 regulating kinase (TP53RK),	1.03658785	0.84702526
CK232291	Hs.417764	1.0352831	0.0331666
NM_022059	chemokine (C-X-C motif) ligand 16 (CXCL16),	1.03526207	0.02347523
NM_032342	chromosome 9 open reading frame 125 (C9orf125),	1.03464177	0.20926307
NM_000248	microphthalmia-associated transcription factor (MITF), transcript variant 4,	1.03446398	0.56201492
NM_006007	zinc finger, A20 domain containing 2 (ZA20D2),	1.03442601	0.01120898
CN806583	IFITM1	1.03428837	0.2258745
NM_178834	layilin (LOC143903),	1.03405501	0.50723978
NM_030891	leucine rich repeat containing 3 (LRRC3),	1.03328755	0.85452672
NM_002610	pyruvate dehydrogenase kinase, isoenzyme 1 (PDK1), nuclear gene encoding mitochondrial protein,	1.03222856	0.17520609
NM_015020	PH domain and leucine rich repeat protein phosphatase-like (PHLPPL),	1.03166484	0.14040113
NM_022059	chemokine (C-X-C motif) ligand 16 (CXCL16),	1.03148268	0.16717326
NM_016232	interleukin 1 receptor-like 1 (IL1RL1), transcript variant 1,	1.03142725	0.65078275
NM_194460	ring finger protein 126 (RNF126), transcript variant 2,	1.03133662	1.009449
NM_003447	zinc finger protein 165 (ZNF165),	1.03129868	0.56394708
BC044226	myosin binding protein H,	1.03086602	0.18356128
NM_024599	rhomboid, veinlet-like 6 (Drosophila) (RHBDL6),	1.03028745	0.03765763
NM_015541	leucine-rich repeats and immunoglobulin-like domains 1 (LRIG1),	1.03026684	0.05530751
NM_002894	retinoblastoma binding protein 8 (RBBP8), transcript variant 1,	1.02946333	0.13474924
NM_032784	thrombospondin, type I, domain containing 2 (THSD2),	1.02870662	0.08785472
XM_170736	solute carrier family 25, member 30 (SLC25A30),	1.0278479	0.91922409
XM_374765	chromosome 10 open reading frame 18 (C10orf18),	1.02749318	0.92545834
NM_016639	tumor necrosis factor receptor superfamily, member 12A (TNFRSF12A),	1.02738797	0.16821613
NM_006965	zinc finger protein 24 (KOX 17) (ZNF24),	1.02732763	0.06018581
NM_133376	integrin, beta 1 (fibronectin receptor, beta polypeptide, antigen CD29 includes MDF2, MSK12) (ITGB1), transcript variant 1E,	1.02711023	0.10085955
NM_015483	kelch repeat and BTB (POZ) domain containing 2 (KBTBD2),	1.02670949	0.10807701
XR_011805	Macaca mulatta mitochondrial tumor suppressor 1 isoform 1 (LOC702990),	1.02654618	0.14858792
NM_004844	SH3-domain binding protein 5 (BTK-associated) (SH3BP5),	1.02632902	0.08109276
NM_001539	DnaJ (Hsp40) homolog, subfamily A, member 1 (DNAJA1),	1.02627546	0.09096899
NM_007185	trinucleotide repeat containing 4 (TNRC4),	1.02552938	0.6763485
NM_024599	rhomboid, veinlet-like 6 (Drosophila) (RHBDL6),	1.02490777	0.08713668
NM_012395	PFTAIRE protein kinase 1 (PFTK1),	1.02473965	0.03222159
NM_016027	lactamase, beta 2 (LACTB2),	1.02427625	0.02710502
CO645321	SEC24A	1.02356557	0.06223098
NM_033280	signal peptidase complex (18kD) (LOC90701),	1.02354275	0.10623154
NM_002908	v-rel reticuloendotheliosis viral oncogene homolog (avian) (REL),	1.02328116	0.54493005
NM_003842	tumor necrosis factor receptor superfamily, member 10b (TNFRSF10B), transcript variant 1,	1.02227489	0.5897337
NM_000176	nuclear receptor subfamily 3, group C, member 1 (glucocorticoid receptor) (NR3C1),	1.02212612	0.10581866
NM_004024	activating transcription factor 3 (ATF3),	1.02055309	0.10174593
DQ480434	Macaca mulatta EPH receptor A2 (EPHA2)	1.01676553	0.13779795
NM_014685	homocysteine-inducible, endoplasmic reticulum stress-inducible, ubiquitin-like domain member 1 (HERPUD1),	1.01602655	0.10531242
NM_080920	gamma-glutamyltransferase-like activity 4 (GGTLA4), transcript variant C,	1.01575748	0.26173311
NM_203391	glycerol kinase (GK), transcript variant 1,	1.0151724	0.34408805
NM_001034	ribonucleotide reductase M2 polypeptide (RRM2),	1.01469804	0.95646083

NM_022068	family with sequence similarity 38, member B (FAM38B),	1.01465365	0.05128383
NM_003364	uridine phosphorylase 1 (UPP1), transcript variant 1,	1.0143616	0.14546211
NM_194430	ribonuclease, RNase A family, 4 (RNASE4), transcript variant 1,	1.01354166	0.06564946
NM_007361	nidogen 2 (osteonidogen) (NID2),	1.01331811	0.05048287
NM_032784	thrombospondin, type I, domain containing 2 (THSD2),	1.0132932	0.01524012
NM_004261	15 kDa selenoprotein (SEP15), transcript variant 1,	1.01248812	0.14563853
NM_018155	solute carrier family 25, member 36 (SLC25A36),	1.01245463	0.3397418
NM_020726	neurolysin (metallopeptidase M3 family) (NLN),	1.01238177	0.61675638
NM_005922	mitogen-activated protein kinase kinase kinase 4 (MAP3K4), transcript variant 1,	1.01218997	0.13767505
CN806583	IFITM1	1.01186644	0.14099917
XR_010002	Macaca mulatta hypothetical protein LOC695376 (LOC695376),	1.01109847	0.66256453
NM_170721	musashi homolog 2 (Drosophila) (MSI2), transcript variant 2,	1.00993723	0.81529679
NM_017870	heat shock 70kDa protein 5 (glucose-regulated protein, 78kDa) binding protein 1 (HSPA5BP1), transcript variant 1,	1.00992981	0.13505445
NM_000764	cytochrome P450, family 2, subfamily A, polypeptide 7 (CYP2A7), transcript variant 1,	1.00983709	0.21218368
NM_018360	chromosome X open reading frame 15 (CXorf15),	1.00926659	0.49685235
NM_152342	chromodomain protein, Y-like 2 (CDYL2),	1.00908513	0.89052304
NM_144499	guanine nucleotide binding protein (G protein), alpha transducing activity polypeptide 1 (GNAT1), transcript variant 1,	1.00905763	0.0062311
XR_014443	Macaca mulatta hydroxysteroid (17-beta) dehydrogenase 7 (LOC720399),	1.00852043	0.04093945
NM_002648	pim-1 oncogene (PIM1),	1.00844485	0.11676154
NM_024580	elongation factor Tu GTP binding domain containing 1 (EFTUD1),	1.008231	0.41534456
NM_018945	phosphodiesterase 7B (PDE7B),	1.00822918	0.38802917
NM_001539	DnaJ (Hsp40) homolog, subfamily A, member 1 (DNAJA1),	1.0081613	0.09451651
XR_011805	Macaca mulatta mitochondrial tumor suppressor 1 isoform 1 (LOC702990),	1.0080396	0.10811137
NM_014631	SH3 and PX domains 2A (SH3PXD2A),	1.00745184	0.19776683
NM_018950	major histocompatibility complex, class I, F (HLA-F),	1.00744152	0.03900827
NM_030925	calcium binding protein 39-like (CAB39L),	1.00719244	0.68412952
NM_016216	debranching enzyme homolog 1 (S. cerevisiae) (DBR1),	1.00702414	0.54749331
NM_033280	signal peptidase complex (18kD) (LOC90701),	1.00628842	0.01645745
NM_016042	exosome component 3 (EXOSC3), transcript variant 1,	1.00620612	0.19233524
BC107879	sterol-C4-methyl oxidase-like,	1.00552963	0.02141218
CN802066	Hs.432862	1.00551756	0.38166999
CN641390	Hs.405253	1.00523155	0.31680711
NM_144600	hypothetical protein FLJ31153 (FLJ31153),	1.00497856	0.27392635
NM_002130	3-hydroxy-3-methylglutaryl-Coenzyme A synthase 1 (soluble) (HMGCS1),	1.00442062	0.07574969
NM_016816	2',5'-oligoadenylate synthetase 1, 40/46kDa (OAS1), transcript variant E18,	1.00337292	0.10424155
XR_011459	Macaca mulatta enabled homolog isoform b (LOC700559),	1.00329481	0.49651695
NM_020154	chromosome 15 open reading frame 24 (C15orf24),	1.00293238	0.04912263
NM_178868	chemokine-like factor super family 8 (CKLFSF8),	1.00202542	0.39661494
NM_024295	Der1-like domain family, member 1 (DERL1),	1.00157551	0.07452334
NM_006889	CD86 antigen (CD28 antigen ligand 2, B7-2 antigen) (CD86), transcript variant 2,	1.0001667	0.12128343
NM_00100723[illegible]	immunoglobulin superfamily, member 3 (IGSF3), transcript variant 2,	1.00013605	0.39953753
NM_032229	SLIT and NTRK-like family, member 6 (SLITRK6),	-4.56229153	1.57074403
NM_144717	fibronectin type III domain containing 6 (FNDC6),	-4.46419842	1.0604469
NM_006183	neurotensin (NTS),	-4.43210792	0.05218555
NM_000587	complement component 7 (C7),	-4.32839615	2.06166636

NM_014057	osteoglycin (osteoinductive factor, mimecan) (OGN), transcript variant 3,	-4.09986633	0.30880483
NM_000677	adenosine A3 receptor (ADORA3), transcript variant 2,	-3.94129888	1.79851039
NM_144966	FRAS1 related extracellular matrix 1 (FREM1),	-3.94057579	0.14996058
NM_030820	collagen, type XXI, alpha 1 (COL21A1),	-3.86660588	0.67237756
NM_006183	neurotensin (NTS),	-3.84312856	0.18001111
NM_014057	osteoglycin (osteoinductive factor, mimecan) (OGN), transcript variant 3,	-3.81733669	0.24407127
XM_371114	formin homology 2 domain containing 3 (FHOD3),	-3.78323463	2.78649241
NM_032133	MYCBP associated protein (MYCBPAP),	-3.75729001	1.97125164
NM_021233	DNase II-like acid DNase (DLAD), transcript variant 1,	-3.73903388	0.1259371
NM_001463	frizzled-related protein (FRZB),	-3.71440229	0.12224897
NM_001463	frizzled-related protein (FRZB),	-3.68462252	0.14525019
NM_005833	Rab9 effector p40 (RAB9P40),	-3.68376736	2.24802878
NM_021233	DNase II-like acid DNase (DLAD), transcript variant 1,	-3.6762502	0.22156618
CN802399	KIAA1946	-3.54530009	0.329749
NM_058173	small breast epithelial mucin (LOC118430),	-3.53478226	2.083793
NM_030623	SPHK1 (sphingosine kinase type 1) interacting protein (SKIP),	-3.51033447	0.20007085
NM_002776	kallikrein 10 (KLK10), transcript variant 1,	-3.50069252	0.17102178
NM_005408	chemokine (C-C motif) ligand 13 (CCL13),	-3.49308052	0.15095751
NM_016948	par-6 partitioning defective 6 homolog alpha (C.elegans) (PARD6A),	-3.42971787	0.38081043
XM_086188	dnaj-like protein (LOC148418),	-3.42933381	0.02803611
NM_001878	cellular retinoic acid binding protein 2 (CRABP2),	-3.4177356	0.10630068
NM_005408	chemokine (C-C motif) ligand 13 (CCL13),	-3.41301033	0.02434333
NM_005045	reelin (RELN), transcript variant 1,	-3.39251814	1.93618514
NM_001819	chromogranin B (secretogranin 1) (CHGB),	-3.37465909	0.43237095
NM_201591	glycoprotein M6A (GPM6A), transcript variant 2,	-3.3522337	1.81832342
NM_014479	ADAM-like, decysin 1 (ADAMDEC1),	-3.35041503	1.60920336
NM_153229	hypothetical protein FLJ33318 (FLJ33318),	-3.33501162	0.15326841
NM_00101171<br>6	Unknown	-3.33051523	2.49648496
NM_153343	ectonucleotide pyrophosphatase/phosphodiesterase 6 (ENPP6),	-3.32869288	0.10384788
CN643667	C5orf13	-3.28855084	0.27096483
NM_001444	fatty acid binding protein 5 (psoriasis-associated) (FABP5),	-3.25953626	0.04031433
NM_005602	claudin 11 (oligodendrocyte transmembrane protein) (CLDN11),	-3.24593191	0.3654523
NM_178565	hypothetical protein MGC35555 (MGC35555),	-3.24298554	0.00956361
CN802399	KIAA1946	-3.23588146	0.18515833
NM_001878	cellular retinoic acid binding protein 2 (CRABP2),	-3.23220199	0.01536622
NM_005602	claudin 11 (oligodendrocyte transmembrane protein) (CLDN11),	-3.23206792	0.07475086
CN802399	KIAA1946	-3.21569719	0.22200196
XR_010680	CD180 antigen (CD180),	-3.19495245	0.85920253
NM_152459	hypothetical protein MGC45438 (MGC45438),	-3.19178038	0.10981592
CN643667	C5orf13	-3.18751737	0.13745818
NM_003862	fibroblast growth factor 18 (FGF18), transcript variant 1,	-3.18692289	0.35296594
NM_032411	esophageal cancer related gene 4 protein (ECRG4),	-3.18574549	0.24707938
CN802399	KIAA1946	-3.17237401	0.19862697
NM_152459	hypothetical protein MGC45438 (MGC45438),	-3.16765994	0.0547976
NM_001444	fatty acid binding protein 5 (psoriasis-associated) (FABP5),	-3.12519362	0.09045645

NM_002776	kallikrein 10 (KLK10), transcript variant 1,	-3.10685269	0.45256943
NM_153343	ectonucleotide pyrophosphatase/phosphodiesterase 6 (ENPP6),	-3.08321946	0.01259052
NM_182530	hypothetical protein FLJ25056 (FLJ25056),	-3.08046073	0.99710727
NM_032495	homeodomain-only protein (HOP), transcript variant 1,	-3.07945603	1.50868669
NM_178565	hypothetical protein MGC35555 (MGC35555),	-3.06014272	0.49127035
NM_000587	complement component 7 (C7),	-3.04252964	0.41374155
NM_002404	microfibrillar-associated protein 4 (MFAP4),	-3.0231182	0.03668718
NM_014945	actin binding LIM protein family, member 3 (ABLIM3),	-3.01643293	0.03063853
NM_002825	pleiotrophin (heparin binding growth factor 8, neurite growth-promoting factor 1) (PTN),	-3.01233963	0.19444062
CN806534	CMKOR1	-3.00092297	0.05128785
NM_006953	uroplakin 3A (UPK3A),	-2.99691414	0.44959723
NM_016613	hypothetical protein DKFZp434L142 (DKFZp434L142),	-2.98019899	0.1130761
NM_013227	aggrecan 1 (chondroitin sulfate proteoglycan 1, large aggregating proteoglycan, antigen identified by monoclonal antibody A0122) (AGC1), transcript variant 2,	-2.97568403	2.4604346
NM_002921	retinal G protein coupled receptor (RGR), transcript variant 1,	-2.97235091	1.87980098
NM_004464	fibroblast growth factor 5 (FGF5), transcript variant 1,	-2.96686495	1.63506717
NM_001941	desmocollin 3 (DSC3), transcript variant Dsc3a,	-2.96100791	1.01420403
NM_176887	taste receptor, type 2, member 46 (TAS2R46),	-2.95939419	2.82662435
NM_002825	pleiotrophin (heparin binding growth factor 8, neurite growth-promoting factor 1) (PTN),	-2.95062114	0.10857851
NM_032510	par-6 partitioning defective 6 homolog gamma (C. elegans) (PARD6G),	-2.94369258	0.59840095
NM_015166	megalencephalic leukoencephalopathy with subcortical cysts 1 (MLC1), transcript variant 1,	-2.94013164	0.02626784
NM_000677	adenosine A3 receptor (ADORA3), transcript variant 2,	-2.88791878	0.50676253
NM_002023	fibromodulin (FMOD),	-2.87130911	0.19580321
NM_001046	solute carrier family 12 (sodium/potassium/chloride transporters), member 2 (SLC12A2),	-2.86656141	0.14428324
NM_020404	CD164 sialomucin-like 1 (CD164L1),	-2.86614243	0.18189205
NM_003014	secreted frizzled-related protein 4 (SFRP4),	-2.84550564	0.6977988
XM_375762	netrin G1 (NTNG1),	-2.84432	1.57249122
NM_022036	G protein-coupled receptor, family C, group 5, member C (GPRC5C), transcript variant 1,	-2.83998434	0.00536277
CN806534	CMKOR1	-2.83864611	0.07163521
NM_030623	SPHK1 (sphingosine kinase type 1) interacting protein (SKIP),	-2.83476514	0.17168475
NM_002287	leukocyte-associated Ig-like receptor 1 (LAIR1), transcript variant a,	-2.82855018	0.19990558
NM_019885	cytochrome P450, family 26, subfamily B, polypeptide 1 (CYP26B1),	-2.81993499	0.20281827
NM_015166	megalencephalic leukoencephalopathy with subcortical cysts 1 (MLC1), transcript variant 1,	-2.81942768	0.01743134
NM_006157	NEL-like 1 (chicken) (NELL1),	-2.81918804	0.53133335
DQ223038	putative serine protease 35 complete cds	-2.80584603	0.4023879
NM_001289	chloride intracellular channel 2 (CLIC2),	-2.80211785	0.76884162
XM_086188	dnaj-like protein (LOC148418),	-2.79842505	0.91283662
NM_005202	collagen, type VIII, alpha 2 (COL8A2),	-2.79373274	0.19124026
NM_005045	reelin (RELN), transcript variant 1,	-2.7889654	0.56234086
NM_004791	integrin, beta-like 1 (with EGF-like repeat domains) (ITGBL1),	-2.7824398	0.11947387
NM_004271	lymphocyte antigen 86 (LY86),	-2.77945515	0.35164396
NM_006157	NEL-like 1 (chicken) (NELL1),	-2.77232166	0.09442631
NM_014945	actin binding LIM protein family, member 3 (ABLIM3),	-2.77002765	0.3378694
NM_019885	cytochrome P450, family 26, subfamily B, polypeptide 1 (CYP26B1),	-2.76728275	0.05796165
NM_144966	FRAS1 related extracellular matrix 1 (FREM1),	-2.75734653	0.44734358
NM_004791	integrin, beta-like 1 (with EGF-like repeat domains) (ITGBL1),	-2.75187279	0.03190776

NM_002432	myeloid cell nuclear differentiation antigen (MNDA),	-2.75143273	0.54602654
NM_020404	CD164 sialomucin-like 1 (CD164L1),	-2.74711038	0.04198456
NM_000088	collagen, type I, alpha 1 (COL1A1),	-2.74478623	0.18214748
A_01_P003289	Unknown	-2.73379557	0.31324505
NM_007245	ataxin 2 related protein (A2LP), transcript variant A,	-2.73114433	1.80452069
NM_006307	sushi-repeat-containing protein, X-linked (SRPX),	-2.72814108	0.12000428
NM_005014	osteomodulin (OMD),	-2.72534307	0.35174608
NM_030820	collagen, type XXI, alpha 1 (COL21A1),	-2.72130709	0.05525268
NM_181481	chromosome 18 open reading frame 1 (C18orf1), transcript variant a1,	-2.71720135	1.34333536
NM_153370	protease inhibitor 16 (PI16),	-2.71370475	0.16330511
NM_002404	microfibrillar-associated protein 4 (MFAP4),	-2.71001455	0.06972399
CK230655	ILLUMIGEN_MCQ_1009 Katze_MMPL2 cDNA 5' human Unigene Hs.500464, sequence	-2.69252577	0.09465083
NM_022138	SPARC related modular calcium binding 2 (SMOC2),	-2.68427466	0.33925351
NM_022046	kallikrein 14 (KLK14),	-2.66359098	0.16650843
NM_016580	protocadherin 12 (PCDH12),	-2.66161052	0.39837353
NM_001046	solute carrier family 12 (sodium/potassium/chloride transporters), member 2 (SLC12A2),	-2.65460553	0.3525328
NM_016580	protocadherin 12 (PCDH12),	-2.65250082	0.39612285
NM_024812	brain and acute leukemia, cytoplasmic (BAALC), transcript variant 1,	-2.64122993	0.76725054
NM_000092	collagen, type IV, alpha 4 (COL4A4),	-2.63586635	2.57729099
XM_033173	protocadherin 19 (PCDH19),	-2.63242072	0.27361717
NM_005014	osteomodulin (OMD),	-2.6289713	0.71994344
XM_033173	protocadherin 19 (PCDH19),	-2.62733392	0.09408913
NM_006228	prepronociceptin (PNOC),	-2.62214293	0.00637995
XR_010210	hypothetical protein LOC696802 (LOC696802),	-2.61699884	0.01071728
NM_030781	collectin sub-family member 12 (COLEC12), transcript variant II,	-2.61588694	0.87561757
A_01_P007245	Unknown	-2.60150556	1.05349176
NM_005876	aortic preferentially expressed protein 1 (APEG1),	-2.58706733	0.35781363
NM_006522	wingless-type MMTV integration site family, member 6 (WNT6),	-2.58268872	0.9081198
NM_006307	sushi-repeat-containing protein, X-linked (SRPX),	-2.57904798	0.26770654
NM_022036	G protein-coupled receptor, family C, group 5, member C (GPRC5C), transcript variant 1,	-2.56947055	0.13787947
NM_002403	microfibrillar-associated protein 2 (MFAP2), transcript variant 2,	-2.56746732	0.02818602
XR_014068	ATP-binding cassette, sub-family A, member 10 (LOC714206),	-2.56565682	0.01899196
NM_006080	sema domain, immunoglobulin domain (Ig), short basic domain, secreted, (semaphorin) 3A (SEMA3A),	-2.56038554	0.36803111
NM_205848	synaptotagmin VI (SYT6),	-2.54440143	1.22327461
NM_018965	triggering receptor expressed on myeloid cells 2 (TREM2),	-2.54074034	0.23333674
DQ223038	putative serine protease 35 complete cds	-2.52826072	0.46010919
NM_00100193<br>6	KIAA1914 (KIAA1914), transcript variant 1,	-2.52150255	0.93632016
NM_000860	hydroxyprostaglandin dehydrogenase 15-(NAD) (HPGD),	-2.516996	0.18301442
NM_006566	CD226 antigen (CD226),	-2.51485924	2.33159254
NM_003283	troponin T1, skeletal, slow (TNNT1),	-2.50237562	0.14202145
NM_006536	chloride channel, calcium activated, family member 2 (CLCA2),	-2.49917272	1.40296285
NM_016613	hypothetical protein DKFZp434L142 (DKFZp434L142),	-2.49678072	0.93261561

NM_00101298<br>6	hypothetical LOC388910 (LOC388910),	-2.47112713	1.16218534
CK230655	ILLUMIGEN_MCQ_1009 Katze_MMPL2 cDNA 5' human Unigene Hs.500464, sequence	-2.46986334	0.05534035
NM_022161	baculoviral IAP repeat-containing 7 (livin) (BIRC7), transcript variant 2,	-2.46435767	1.27275124
NM_018965	triggering receptor expressed on myeloid cells 2 (TREM2),	-2.46087023	0.10226256
NM_032411	esophageal cancer related gene 4 protein (ECRG4),	-2.46081109	0.0762678
NM_006228	prepronociceptin (PNOC),	-2.45935876	0.05084436
NM_033209	Thy-1 co-transcribed (LOC94105),	-2.45416952	0.09238498
NM_144620	hypothetical protein MGC14816 (MGC14816),	-2.45285037	1.40574949
NM_000213	integrin, beta 4 (ITGB4),	-2.44738384	0.07929128
XR_010210	hypothetical protein LOC696802 (LOC696802),	-2.44222094	0.01104815
NM_007268	V-set and immunoglobulin domain containing 4 (VSIG4),	-2.44159637	0.11775024
NM_001175	Rho GDP dissociation inhibitor (GDI) beta (ARHGDIB),	-2.4412124	0.06224915
NM_000698	arachidonate 5-lipoxygenase (ALOX5),	-2.43600801	0.46629145
NM_153370	protease inhibitor 16 (PI16),	-2.43065081	0.08642414
NM_152718	hypothetical protein FLJ32009 (FLJ32009),	-2.42875997	0.00645611
NM_000803	folate receptor 2 (fetal) (FOLR2),	-2.41398851	0.02669921
XM_375720	regulating synaptic membrane exocytosis 3 (RIMS3),	-2.4129329	0.01500397
NM_016257	hippocalcin like 4 (HPCAL4),	-2.40582767	1.97731595
NM_033209	Thy-1 co-transcribed (LOC94105),	-2.40352067	0.09352626
NM_000803	folate receptor 2 (fetal) (FOLR2),	-2.38598202	0.12360793
NM_004949	desmocollin 2 (DSC2), transcript variant Dsc2b,	-2.37682173	0.65465755
CO726190	CD36	-2.37669201	0.02709566
NM_015931	fls485 (LOC51066),	-2.36738215	0.08987748
NM_152718	hypothetical protein FLJ32009 (FLJ32009),	-2.36729836	0.28309544
NM_005876	aortic preferentially expressed protein 1 (APEG1),	-2.36692421	0.1015344
NM_005524	hairy and enhancer of split 1, (Drosophila) (HES1),	-2.36414667	0.07410457
NM_015931	fls485 (LOC51066),	-2.36114456	0.57825624
NM_002988	chemokine (C-C motif) ligand 18 (pulmonary and activation-regulated) (CCL18),	-2.35487388	0.13516741
NM_005410	selenoprotein P, plasma, 1 (SEPP1),	-2.34723897	0.08324488
XR_013754	protease, serine, 12 (LOC713377),	-2.34293108	0.16737731
NM_033209	Thy-1 co-transcribed (LOC94105),	-2.34235327	0.13503471
NM_001175	Rho GDP dissociation inhibitor (GDI) beta (ARHGDIB),	-2.34018762	0.02057114
NM_004271	lymphocyte antigen 86 (LY86),	-2.33307581	0.19762406
NM_017415	kelch-like 3 (Drosophila) (KLHL3),	-2.33187401	0.67860218
NM_000370	tocopherol (alpha) transfer protein (ataxia (Friedreich-like) with vitamin E deficiency) (TTPA),	-2.32986353	1.93623936
NM_022138	SPARC related modular calcium binding 2 (SMOC2),	-2.32534477	0.39300944
NM_015991	complement component 1, q subcomponent, alpha polypeptide (C1QA),	-2.31959072	0.04999588
NM_005410	selenoprotein P, plasma, 1 (SEPP1),	-2.31792712	0.08400062
NM_025268	hole gene (MGC4659),	-2.31737542	0.41459532
NM_002023	fibromodulin (FMOD),	-2.31030554	0.01338967
NM_002988	chemokine (C-C motif) ligand 18 (pulmonary and activation-regulated) (CCL18),	-2.30456269	0.07083036
NM_000860	hydroxyprostaglandin dehydrogenase 15-(NAD) (HPGD),	-2.30075044	0.21587602
NM_005447	peptidylglycine alpha-amidating monooxygenase COOH-terminal interactor (PAMCI),	-2.29994207	0.4984499
NM_002403	microfibrillar-associated protein 2 (MFAP2), transcript variant 2,	-2.2981782	0.058179
NM_032495	homeodomain-only protein (HOP), transcript variant 1,	-2.29615674	0.10220485

NM_000213	integrin, beta 4 (ITGB4),	-2.29541866	0.21944338
NM_014178	syntaxin binding protein 6 (amisyn) (STXBP6),	-2.29213912	0.12798673
NM_005524	hairy and enhancer of split 1, (Drosophila) (HES1),	-2.28480387	0.04042521
NM_024637	galactose-3-O-sulfotransferase 4 (GAL3ST4),	-2.28402338	0.3662065
NM_015991	complement component 1, q subcomponent, alpha polypeptide (C1QA),	-2.27828718	0.02872978
XM_499343	paternally expressed 10 (PEG10),	-2.27485144	0.17652133
NM_022046	kallikrein 14 (KLK14),	-2.26827261	0.38925669
NM_152421	hypothetical protein MGC20262 (MGC20262),	-2.26223318	0.12390627
XR_013754	protease, serine, 12 (LOC713377),	-2.25210827	0.12893878
NM_001104	actinin, alpha 3 (ACTN3),	-2.25170396	0.11222513
NM_145056	thymus expressed gene 3-like (MGC15476),	-2.25115411	0.57161962
NM_001853	collagen, type IX, alpha 3 (COL9A3),	-2.24081534	0.12457014
NM_014178	syntaxin binding protein 6 (amisyn) (STXBP6),	-2.23590298	0.05637706
NM_003014	secreted frizzled-related protein 4 (SFRP4),	-2.22832051	0.37691374
NM_003283	troponin T1, skeletal, slow (TNNT1),	-2.21586404	0.00826208
NM_020683	adenosine A3 receptor (ADORA3), transcript variant 1,	-2.21577142	0.41207078
NM_005202	collagen, type VIII, alpha 2 (COL8A2),	-2.20831571	0.00164693
NM_033209	Thy-1 co-transcribed (LOC94105),	-2.20734981	0.06107155
NM_00100727<br>9	RAS-related on chromosome 22 (RRP22), transcript variant 2,	-2.19525284	0.38681117
NM_002307	lectin, galactoside-binding, soluble, 7 (galectin 7) (LGALS7),	-2.19378904	0.38671622
NM_004369	collagen, type VI, alpha 3 (COL6A3), transcript variant 1,	-2.18516882	0.17923286
CO726190	CD36	-2.18149954	0.16504619
NM_001044	solute carrier family 6 (neurotransmitter transporter, dopamine), member 3 (SLC6A3),	-2.18093395	2.17578234
NM_138444	potassium channel tetramerisation domain containing 12 (KCTD12),	-2.17976271	0.08156943
NM_00100727<br>9	RAS-related on chromosome 22 (RRP22), transcript variant 2,	-2.16434215	0.28999166
NM_004934	cadherin 18, type 2 (CDH18),	-2.16190263	0.04683419
NM_00100193<br>6	KIAA1914 (KIAA1914), transcript variant 1,	-2.15432943	0.93571732
NM_001289	chloride intracellular channel 2 (CLIC2),	-2.154189	0.12647844
NM_007037	a disintegrin-like and metalloprotease (reprolysin type) with thrombospondin type 1 motif, 8 (ADAMTS8),	-2.14545022	0.20133628
NM_138788	transmembrane protein 45B (TMEM45B),	-2.13863732	0.27450286
XR_010931	pancreas-enriched phospholipase C (LOC701758),	-2.13551925	0.2797409
NM_181481	chromosome 18 open reading frame 1 (C18orf1), transcript variant a1,	-2.1337536	0.80724439
CO580643	Hs.515465	-2.13039141	0.20106606
NM_024812	brain and acute leukemia, cytoplasmic (BAALC), transcript variant 1,	-2.12764704	0.12909808
NM_002030	formyl peptide receptor-like 2 (FPRL2),	-2.12457162	1.07297419
NM_016210	g20 protein (LOC51161),	-2.12130364	0.00293598
NM_024637	galactose-3-O-sulfotransferase 4 (GAL3ST4),	-2.10940222	0.53260898
NM_007268	V-set and immunoglobulin domain containing 4 (VSIG4),	-2.10561739	0.26176815
NM_004949	desmocollin 2 (DSC2), transcript variant Dsc2b,	-2.09828036	0.39603136
NM_001321	cysteine and glycine-rich protein 2 (CSRP2),	-2.09682563	0.12764739
XR_012709	lipoprotein lipase (LPL),	-2.09570061	0.27624329
XM_499343	paternally expressed 10 (PEG10),	-2.08804944	0.81843047
NM_005824	leucine rich repeat containing 17 (LRRC17),	-2.0839104	0.19784394

NM_001321	cysteine and glycine-rich protein 2 (CSRP2),	-2.08260717	0.10329929
NM_000220	potassium inwardly-rectifying channel, subfamily J, member 1 (KCNJ1), transcript variant rom-k1,	-2.07692296	0.53439856
XM_496215	GPR158-like 1 receptor (LOC440435),	-2.07276123	1.4806096
NM_021170	bHLH factor Hes4 (Hes4),	-2.06670578	0.58983051
XR_010547	CG6432-PA (LOC697823),	-2.06604152	1.18664839
NM_021708	leukocyte-associated Ig-like receptor 1 (LAIR1), transcript variant c,	-2.06405794	0.06823665
NM_025228	TRAF3-interacting Jun N-terminal kinase (JNK)-activating modulator (T3JAM),	-2.06349698	1.24614227
NM_006198	Purkinje cell protein 4 (PCP4),	-2.06309829	1.21590213
NM_001941	desmocollin 3 (DSC3), transcript variant Dsc3a,	-2.06037	0.15506393
A_01_P014940	Unknown	-2.05881939	0.95045554
NM_016610	toll-like receptor 8 (TLR8), transcript variant 1,	-2.05546311	0.96932457
NM_004464	fibroblast growth factor 5 (FGF5), transcript variant 1,	-2.04959816	1.94396611
NM_020125	SLAM family member 8 (SLAMF8),	-2.0492468	0.51849401
NM_001104	actinin, alpha 3 (ACTN3),	-2.04873869	0.04688935
NM_021708	leukocyte-associated Ig-like receptor 1 (LAIR1), transcript variant c,	-2.04745633	0.05753263
NM_024042	meteorin, glial cell differentiation regulator (METRN),	-2.04649768	0.13788552
NM_015396	armadillo repeat containing 8 (ARMC8),	-2.03900572	0.90691194
NM_005822	Down syndrome critical region gene 1-like 1 (DSCR1L1),	-2.03173941	0.03703613
NM_007281	scrapie responsive protein 1 (SCRG1),	-2.02883614	0.2186707
NM_002507	nerve growth factor receptor (TNFR superfamily, member 16) (NGFR),	-2.0277483	0.17498032
NM_144717	fibronectin type III domain containing 6 (FNDC6),	-2.02617963	0.10343767
NM_001147	angiopoietin 2 (ANGPT2),	-2.02542415	0.48478796
XR_010931	pancreas-enriched phospholipase C (LOC701758),	-2.02495006	0.18746738
NM_014479	ADAM-like, decysin 1 (ADAMDEC1),	-2.02455801	0.10086221
NM_153000	adenomatosis polyposis coli down-regulated 1 (APCDD1),	-2.02269501	0.0950754
NM_004934	cadherin 18, type 2 (CDH18),	-2.02245359	0.23450295
NM_181435	C1q and tumor necrosis factor related protein 3 (C1QTNF3),	-2.01555355	1.73560567
NM_024533	carbohydrate (N-acetylglucosamine 6-O) sulfotransferase 5 (CHST5),	-2.01005274	1.50916696
NM_015526	CLIP-170-related protein (CLIPR-59),	-2.00861003	0.13449579
NM_001611	acid phosphatase 5, tartrate resistant (ACP5),	-2.00802658	0.44553577
CO580643	Hs.515465	-2.00785066	0.06669184
NM_182518	hypothetical protein LOC149469 (LOC149469),	-2.00533788	0.73892262
NM_022161	baculoviral IAP repeat-containing 7 (livin) (BIRC7), transcript variant 2,	-2.00315007	0.22708356
NM_024800	NIMA (never in mitosis gene a)- related kinase 11 (NEK11),	-1.98773463	0.26968147
NM_000916	oxytocin receptor (OXTR),	-1.98260072	0.07043404
AF389338	acyl-CoA-desaturase complete cds	-1.98115609	0.12510676
NM_002663	phospholipase D2 (PLD2),	-1.97784985	0.25365693
NM_005849	immunoglobulin superfamily, member 6 (IGSF6),	-1.97771577	0.28312836
NM_00100727 9	RAS-related on chromosome 22 (RRP22), transcript variant 2,	-1.97706578	0.01940163
DV769814	MGP	-1.97591305	0.03636286
NM_138444	potassium channel tetramerisation domain containing 12 (KCTD12),	-1.97372987	0.1852841
NM_002751	mitogen-activated protein kinase 11 (MAPK11), transcript variant 1,	-1.97034196	0.44070007
NM_00103180 4	v-maf musculoaponeurotic fibrosarcoma oncogene homolog (avian) (MAF), transcript variant 2,	-1.96933238	0.23158284

NM_018404	centaurin, alpha 2 (CENTA2),	-1.9680258	0.36374763
NM_032511	chromosome 6 open reading frame 168 (C6orf168),	-1.96683781	0.74229162
NM_170600	SH2 domain containing 3C (SH2D3C),	-1.96591049	0.79218726
NM_00103180<br>4	v-maf musculoaponeurotic fibrosarcoma oncogene homolog (avian) (MAF), transcript variant 2,	-1.96590943	0.09182715
NM_007037	a disintegrin-like and metalloprotease (reprolysin type) with thrombospondin type 1 motif, 8 (ADAMTS8),	-1.95396332	0.25272222
NM_004369	collagen, type VI, alpha 3 (COL6A3), transcript variant 1,	-1.95300214	0.12251737
NM_005269	glioma-associated oncogene homolog (zinc finger protein) (GLI),	-1.95156607	0.69696294
NM_145056	thymus expressed gene 3-like (MGC15476),	-1.9507988	1.02059474
NM_006615	calpain 9 (CAPN9), transcript variant 1,	-1.94755295	1.80438238
NM_014505	potassium large conductance calcium-activated channel, subfamily M, beta member 4 (KCNMB4),	-1.94583264	1.32829832
NM_000371	transthyretin (prealbumin, amyloidosis type I) (TTR),	-1.94385706	0.1709805
NM_134442	cAMP responsive element binding protein 1 (CREB1), transcript variant B,	-1.94225858	0.31879899
NM_003202	transcription factor 7 (T-cell specific, HMG-box) (TCF7), transcript variant 1,	-1.93892646	0.5134399
NM_152775	KM-HN-1 protein (KM-HN-1),	-1.93851912	0.06711774
NM_000698	arachidonate 5-lipoxygenase (ALOX5),	-1.93850202	0.10010346
XR_010680	CD180 antigen (CD180),	-1.93584918	0.33301084
NM_005822	Down syndrome critical region gene 1-like 1 (DSCR1L1),	-1.9358015	0.20669587
XR_012751	melanoma-associated chondroitin sulfate proteoglycan 4 (LOC713086), partial	-1.93484118	0.79339456
NM_004672	mitogen-activated protein kinase kinase kinase 6 (MAP3K6),	-1.93006959	0.04807167
NM_002405	manic fringe homolog (Drosophila) (MFNG),	-1.92950936	0.0544413
NM_016210	g20 protein (LOC51161),	-1.92694663	0.21452391
BX649128	cDNA DKFZp686K0548 (from clone DKFZp686K0548)	-1.92661529	0.36496672
NM_002397	MADS box transcription enhancer factor 2, polypeptide C (myocyte enhancer factor 2C) (MEF2C),	-1.92651518	0.30135184
NM_005849	immunoglobulin superfamily, member 6 (IGSF6),	-1.92644127	0.17382353
AF389338	acyl-CoA-desaturase complete cds	-1.92592642	0.051511
NM_130759	GTPase, IMAP family member 1 (GIMAP1),	-1.92020443	0.45333961
NM_024042	meteorin, glial cell differentiation regulator (METRN),	-1.91542366	0.00676932
NM_171998	RAB39B, member RAS oncogene family (RAB39B),	-1.90635557	1.28314522
NM_003287	tumor protein D52-like 1 (TPD52L1),	-1.90256368	0.29119576
NM_023915	G protein-coupled receptor 87 (GPR87),	-1.90145175	0.34193144
NM_004672	mitogen-activated protein kinase kinase kinase 6 (MAP3K6),	-1.90107804	0.06699361
NM_006953	uroplakin 3A (UPK3A),	-1.90062179	0.00261574
XM_035371	zinc finger, FYVE domain containing 28 (ZFYVE28),	-1.89612155	0.51546611
XR_012709	lipoprotein lipase (LPL),	-1.89569302	0.27054053
AF389338	acyl-CoA-desaturase complete cds	-1.89439879	0.24573517
NM_002438	mannose receptor, C type 1 (MRC1),	-1.89151999	0.69118495
NM_003287	tumor protein D52-like 1 (TPD52L1),	-1.89049348	0.53514501
NM_032402	protocadherin gamma subfamily C, 3 (PCDHGC3), transcript variant 2,	-1.88804896	1.86136911
NM_152423	melanoma associated antigen (mutated) 1-like 1 (MUM1L1),	-1.88740262	0.44219969
NM_144569	hypothetical protein FLJ25348 (FLJ25348),	-1.88661625	0.18049745
NM_002507	nerve growth factor receptor (TNFR superfamily, member 16) (NGFR),	-1.88608291	0.28706176
NM_130759	GTPase, IMAP family member 1 (GIMAP1),	-1.88327498	0.20298082
NM_000870	5-hydroxytryptamine (serotonin) receptor 4 (HTR4), transcript variant b,	-1.8804479	1.49556476
NM_178837	hypothetical testis protein from macaque (LOC352909),	-1.87470782	1.51190736
NM_020125	SLAM family member 8 (SLAMF8),	-1.87129148	0.26371081

CO048892	Hs.406526	-1.8708294	0.43149724
NM_153000	adenomatosis polyposis coli down-regulated 1 (APCDD1),	-1.86725857	0.01171949
XM_376776	thymus high mobility group box protein TOX (TOX),	-1.86479028	0.33968145
NM_001442	fatty acid binding protein 4, adipocyte (FABP4),	-1.86396509	0.36701249
NM_007168	ATP-binding cassette, sub-family A (ABC1), member 8 (ABCA8),	-1.86047829	0.00685943
CN645399	HLA-DRB3	-1.85956025	1.29240823
NM_000916	oxytocin receptor (OXTR),	-1.85782516	0.02960861
DV769814	MGP	-1.85764875	0.07159929
NM_002578	p21 (CDKN1A)-activated kinase 3 (PAK3),	-1.85754501	0.1013258
NM_005824	leucine rich repeat containing 17 (LRRC17),	-1.85375043	0.04105428
NM_000592	complement component 4B (C4B),	-1.85288358	0.66499165
NM_053276	vitrin (VIT),	-1.85200605	0.13947017
XR_012910	4-aminobutyrate aminotransferase precursor (LOC714017),	-1.85150096	0.22352775
NM_178827	hypothetical protein FLJ35834 (FLJ35834),	-1.84329952	1.43345072
CB230657	AGENCOURT_11469158 NICHD_Rh_Ov1 cDNA clone IMAGE:6883614 5', sequence	-1.84105902	0.00952576
NM_000491	complement component 1, q subcomponent, beta polypeptide (C1QB),	-1.8400879	0.09159127
NM_031455	coiled-coil domain containing 3 (CCDC3),	-1.83915497	0.00341498
NM_183357	adenylate cyclase 5 (ADCY5),	-1.83846241	0.47469144
NM_020812	dedicator of cytokinesis 6 (DOCK6),	-1.8362213	0.13633065
NM_002438	mannose receptor, C type 1 (MRC1),	-1.83078544	0.27769985
NM_033274	a disintegrin and metalloproteinase domain 19 (meltrin beta) (ADAM19), transcript variant 2,	-1.82719999	0.05841696
NM_031455	coiled-coil domain containing 3 (CCDC3),	-1.82692755	0.01030128
NM_001611	acid phosphatase 5, tartrate resistant (ACP5),	-1.82259183	0.00170902
NM_001338	coxsackie virus and adenovirus receptor (CXADR),	-1.82251579	0.01500987
NM_019605	SERTA domain containing 4 (SERTAD4),	-1.81170249	0.30655441
NM_015069	zinc finger protein 423 (ZNF423),	-1.80634237	0.10580971
CN646981	NCALD	-1.80511871	1.17307821
AK095614	cDNA FLJ38295 fis, clone FCBBF3012332	-1.80384094	0.12027139
NM_007168	ATP-binding cassette, sub-family A (ABC1), member 8 (ABCA8),	-1.80231114	0.14920328
NM_004616	tetraspanin 8 (TSPAN8),	-1.80201064	0.06254791
NM_000491	complement component 1, q subcomponent, beta polypeptide (C1QB),	-1.80006306	0.02728925
NM_032181	hypothetical protein FLJ13391 (FLJ13391),	-1.79585279	0.45838333
NM_144505	kallikrein 8 (neuropsin/ovasin) (KLK8), transcript variant 2,	-1.79213822	0.1962181
NM_001848	collagen, type VI, alpha 1 (COL6A1),	-1.79148874	0.07829713
NM_006329	fibulin 5 (FBLN5),	-1.79126646	0.12391633
NM_001011716	Unknown	-1.78980064	0.35125375
NM_006522	wingless-type MMTV integration site family, member 6 (WNT6),	-1.78934327	0.74089571
NM_001004019	fibulin 2 (FBLN2), transcript variant 1,	-1.78722797	0.09517317
NM_002133	heme oxygenase (decycling) 1 (HMOX1),	-1.7865747	0.25560439
XM_166420	phosphatase and actin regulator 1 (PHACTR1),	-1.78620631	1.45393623
NM_198501	FLJ42461 protein (FLJ42461),	-1.78470316	0.35914662
NM_001819	chromogranin B (secretogranin 1) (CHGB),	-1.78423318	0.71381156
NM_020812	dedicator of cytokinesis 6 (DOCK6),	-1.78262582	0.05769708
NM_014714	WD and tetratricopeptide repeats 2 (WDTC2),	-1.78057795	1.17743975

NM_002405	manic fringe homolog (Drosophila) (MFNG),	-1.78052618	0.11800427
NM_015526	CLIP-170-related protein (CLIPR-59),	-1.77976323	0.02367067
NM_006536	chloride channel, calcium activated, family member 2 (CLCA2),	-1.77950277	0.03021668
NM_005052	ras-related C3 botulinum toxin substrate 3 (rho family, small GTP binding protein Rac3) (RAC3),	-1.77833533	0.0356949
NM_00100401<br>9	fibulin 2 (FBLN2), transcript variant 1,	-1.77696068	0.02785358
NM_032133	MYCBP associated protein (MYCBPAP),	-1.77676427	1.23029114
NM_032523	oxysterol binding protein-like 6 (OSBPL6), transcript variant 1,	-1.77640688	0.27716877
XR_009973	RAB26, member RAS oncogene family (LOC695143),	-1.77161263	0.13537003
NM_006152	lymphoid-restricted membrane protein (LRMP),	-1.77070354	0.15321554
NM_206890	chromosome 21 open reading frame 106 (C21orf106), transcript variant 3,	-1.76736497	0.04399805
NM_001338	coxsackie virus and adenovirus receptor (CXADR),	-1.76406985	0.26106973
NM_017415	kelch-like 3 (Drosophila) (KLHL3),	-1.7628678	0.22125212
XR_014094	Notch homolog 3 (NOTCH3),	-1.76224939	0.31485929
NM_003650	cystatin F (leukocystatin) (CST7),	-1.7604631	1.64488686
NM_003202	transcription factor 7 (T-cell specific, HMG-box) (TCF7), transcript variant 1,	-1.75884084	0.08059136
NM_138636	toll-like receptor 8 (TLR8), transcript variant 2,	-1.75589561	1.23579863
NM_004594	solute carrier family 9 (sodium/hydrogen exchanger), isoform 5 (SLC9A5),	-1.75537968	0.58880773
CO582652	ENPEP	-1.75471537	0.30834872
NM_173505	ankyrin repeat domain 29 (ANKRD29),	-1.7537701	0.8560603
AF389338	acyl-CoA-desaturase complete cds	-1.75178767	0.4767909
NM_016533	ninjurin 2 (NINJ2),	-1.74978784	0.07974205
A_01_P006523	Unknown	-1.74721361	0.08793419
NM_006578	guanine nucleotide binding protein (G protein), beta 5 (GNB5), transcript variant 1,	-1.74663734	0.13303592
NM_023002	hyaluronan and proteoglycan link protein 4 (HAPLN4),	-1.744362	0.40009993
NM_152775	KM-HN-1 protein (KM-HN-1),	-1.73783369	0.60583681
NM_021643	tribbles homolog 2 (Drosophila) (TRIB2),	-1.73721802	0.04698955
NM_001823	creatine kinase, brain (CKB),	-1.73688239	0.12163559
NM_178545	transmembrane protein 52 (TMEM52),	-1.73547679	0.20334304
NM_004004	gap junction protein, beta 2, 26kDa (connexin 26) (GJB2),	-1.73148177	0.19540051
NM_024533	carbohydrate (N-acetylglucosamine 6-O) sulfotransferase 5 (CHST5),	-1.72689868	1.18315769
NM_004717	diacylglycerol kinase, iota (DGKI),	-1.72425317	0.98774477
NM_015069	zinc finger protein 423 (ZNF423),	-1.72290878	0.03732939
NM_182798	hypothetical protein FLJ39155 (FLJ39155), transcript variant 2,	-1.71954943	0.36912349
NM_030945	C1q and tumor necrosis factor related protein 3 (C1QTNF3), transcript variant 1,	-1.71643011	0.08464866
NM_019018	hypothetical protein FLJ11127 (FLJ11127),	-1.71403064	0.04800555
NM_003948	cyclin-dependent kinase-like 2 (CDC2-related kinase) (CDKL2),	-1.70929054	0.92235616
XR_010456	keratin 19 (LOC698425),	-1.70665682	0.09537178
NM_012101	tripartite motif-containing 29 (TRIM29), transcript variant 1,	-1.70155757	0.1079691
NM_006697	cisplatin resistance associated (CRA),	-1.70078972	0.00038128
NM_012292	minor histocompatibility antigen HA-1 (HA-1),	-1.70021108	0.18515386
NM_016270	Kruppel-like factor 2 (lung) (KLF2),	-1.69589496	0.01268866
NM_002397	MADS box transcription enhancer factor 2, polypeptide C (myocyte enhancer factor 2C) (MEF2C),	-1.6949779	0.23277902
NM_004734	doublecortin and CaM kinase-like 1 (DCAMKL1),	-1.69237747	0.08695573
NM_030945	C1q and tumor necrosis factor related protein 3 (C1QTNF3), transcript variant 1,	-1.68990391	0.01581679

NM_001974	egf-like module containing, mucin-like, hormone receptor-like 1 (EMR1),	-1.68934083	0.58251665
NM_000170	glycine dehydrogenase (decarboxylating; glycine decarboxylase, glycine cleavage system protein P) (GLDC),	-1.68755865	0.29056417
NM_022047	differentially expressed in FDCP 6 homolog (mouse) (DEF6),	-1.68748989	0.08766231
NM_002663	phospholipase D2 (PLD2),	-1.67445576	0.605014
CO048892	Hs.406526	-1.67137088	0.28760341
NM_031910	C1q and tumor necrosis factor related protein 6 (C1QTNF6), transcript variant 1,	-1.67061022	1.31979382
NM_000355	transcobalamin II; macrocytic anemia (TCN2),	-1.66837893	0.02331972
NM_032256	hypothetical protein DKFZp434K2435 (DKFZp434K2435),	-1.66797369	0.70320243
CB554865	MMSP0022_D04 MMSP cDNA, sequence	-1.6663898	0.08112177
NM_032511	chromosome 6 open reading frame 168 (C6orf168),	-1.6650419	0.00724
NM_00100534<br>0	glycoprotein (transmembrane) nmb (GPNMB), transcript variant 1,	-1.66494758	0.01698705
NM_003088	fascin homolog 1, actin-bundling protein (Strongylocentrotus purpuratus) (FSCN1),	-1.66343591	0.12775
NM_005934	myeloid/lymphoid or mixed-lineage leukemia (trithorax homolog, Drosophila); translocated to, 1 (MLLT1),	-1.65232213	1.35520552
NM_012101	tripartite motif-containing 29 (TRIM29), transcript variant 1,	-1.65099239	0.1122792
NM_138636	toll-like receptor 8 (TLR8), transcript variant 2,	-1.65073759	0.30685621
XR_012719	alpha 2 type V collagen (COL5A2),	-1.65056818	0.10143303
A_01_P003289	Unknown	-1.65004894	0.79659813
NM_178837	hypothetical testis protein from macaque (LOC352909),	-1.64859377	0.42066508
NM_018404	centaurin, alpha 2 (CENTA2),	-1.64824064	0.34127627
NM_032181	hypothetical protein FLJ13391 (FLJ13391),	-1.64443876	0.21622591
NM_016533	ninjurin 2 (NINJ2),	-1.64353824	0.25480882
NM_025045	hypothetical protein FLJ22582 (FLJ22582),	-1.64340805	0.62556579
XR_012616	upregulated in colorectal cancer gene 1 protein precursor (LOC705348),	-1.64082093	0.24348732
NM_006009	tubulin, alpha 3 (TUBA3),	-1.63987177	0.13829706
NM_006578	guanine nucleotide binding protein (G protein), beta 5 (GNB5), transcript variant 1,	-1.63938913	0.39728081
NM_030567	hypothetical protein MGC10772 (MGC10772),	-1.63836594	0.06327108
XR_014247	C3 and PZP-like, alpha-2-macroglobulin domain containing 8 (LOC719660),	-1.63787529	0.01158757
NM_003088	fascin homolog 1, actin-bundling protein (Strongylocentrotus purpuratus) (FSCN1),	-1.63758511	0.05735006
NM_002751	mitogen-activated protein kinase 11 (MAPK11), transcript variant 1,	-1.63546573	0.42765764
NM_017872	interphase cytoplasmic foci protein 45 (ICF45),	-1.63453054	1.26332338
NM_032523	oxysterol binding protein-like 6 (OSBPL6), transcript variant 1,	-1.63404802	0.06773622
NM_001056	sulfotransferase family, cytosolic, 1C, member 1 (SULT1C1), transcript variant 1,	-1.63284502	1.39469678
NM_024792	membrane protein expressed in epithelial-like lung adenocarcinoma (CT120),	-1.63228272	0.50630431
CB554865	MMSP0022_D04 MMSP cDNA, sequence	-1.63194049	0.217346
NM_144490	A kinase (PRKA) anchor protein 11 (AKAP11), transcript variant 2,	-1.63084574	0.30447216
NM_025165	elongation factor RNA polymerase II-like 3 (ELL3),	-1.6306086	0.46141557
NM_000495	collagen, type IV, alpha 5 (Alport syndrome) (COL4A5), transcript variant 1,	-1.62680393	0.09147153
NM_006009	tubulin, alpha 3 (TUBA3),	-1.62668891	0.10441538
NM_003948	cyclin-dependent kinase-like 2 (CDC2-related kinase) (CDKL2),	-1.62236931	0.29299312
XR_012719	alpha 2 type V collagen (COL5A2),	-1.61995575	0.10416236
CO048892	Hs.406526	-1.61852408	0.06670274
NM_005615	ribonuclease, RNase A family, k6 (RNASE6),	-1.61631629	0.09722955
NM_004594	solute carrier family 9 (sodium/hydrogen exchanger), isoform 5 (SLC9A5),	-1.61256233	1.13393994
NM_012445	spondin 2, extracellular matrix protein (SPON2),	-1.61163066	0.02445996

NM_144621	zinc finger and BTB domain containing 8 (ZBTB8),	-1.61089922	0.02824139
NM_004616	tetraspanin 8 (TSPAN8),	-1.60772909	0.09248098
CB230657	AGENCOURT_11469158 NICHD_Rh_Ov1 cDNA clone IMAGE:6883614 5', sequence	-1.60715863	0.12585759
NM_006059	laminin, gamma 3 (LAMC3),	-1.60369248	1.23769943
NM_00100709 7	neurotrophic tyrosine kinase, receptor, type 2 (NTRK2), transcript variant b,	-1.60151849	0.24525718
NM_005928	milk fat globule-EGF factor 8 protein (MFGE8),	-1.60089418	0.07647695
NM_153756	fibronectin type III domain containing 5 (FNDC5),	-1.60064922	0.24275937
NM_174898	hypothetical protein LOC129530 (LOC129530),	-1.59647442	0.50845837
NM_001290	LIM domain binding 2 (LDB2),	-1.59408653	0.18834195
NM_000355	transcobalamin II; macrocytic anemia (TCN2),	-1.58893394	0.18704179
NM_001848	collagen, type VI, alpha 1 (COL6A1),	-1.58569597	0.02172074
NM_000170	glycine dehydrogenase (decarboxylating; glycine decarboxylase, glycine cleavage system protein P) (GLDC),	-1.58499256	0.26917772
NM_000850	glutathione S-transferase M4 (GSTM4), transcript variant 1,	-1.58373198	0.05584529
NM_138393	chromosome 19 open reading frame 32 (C19orf32),	-1.58353005	0.14733547
NM_176798	pyrimidinergic receptor P2Y, G-protein coupled, 6 (P2RY6), transcript variant 2,	-1.58043743	0.16947133
NM_138284	interleukin 17D (IL17D),	-1.57688109	0.17604298
XR_014213	tenascin XB isoform 1 (LOC716998),	-1.57359168	0.07666073
NM_175061	juxtaposed with another zinc finger gene 1 (JAZF1),	-1.57282171	0.10180674
NM_005928	milk fat globule-EGF factor 8 protein (MFGE8),	-1.5660626	0.10119751
NM_031910	C1q and tumor necrosis factor related protein 6 (C1QTNF6), transcript variant 1,	-1.56594388	0.22923004
NM_183357	adenylate cyclase 5 (ADCY5),	-1.5646233	0.11320315
CO582652	ENPEP	-1.55872878	0.39557589
NM_000095	cartilage oligomeric matrix protein (COMP),	-1.55531274	0.08939454
NM_016270	Kruppel-like factor 2 (lung) (KLF2),	-1.55453611	0.07677151
NM_006152	lymphoid-restricted membrane protein (LRMP),	-1.55196279	0.19327128
NM_033274	a disintegrin and metalloproteinase domain 19 (meltrin beta) (ADAM19), transcript variant 2,	-1.55083893	0.25833283
XR_013734	neural stem cell-derived dendrite regulator (LOC717799),	-1.55053648	0.82917355
NM_007281	scrapie responsive protein 1 (SCRG1),	-1.5470592	0.02508185
NM_001147	angiopoietin 2 (ANGPT2),	-1.54677207	0.52704838
NM_052880	HGFL gene (MGC17330),	-1.54644273	0.27753375
NM_030899	zinc finger protein 323 (ZNF323),	-1.5455224	0.28960534
NM_022783	DEP domain containing 6 (DEPDC6),	-1.54145227	0.03851967
NM_006475	periostin, osteoblast specific factor (POSTN),	-1.54045125	0.21387969
NM_016179	transient receptor potential cation channel, subfamily C, member 4 (TRPC4),	-1.54032912	0.77753764
NM_152732	chromosome 6 open reading frame 206 (C6orf206),	-1.53939536	0.07624353
NM_024660	hypothetical protein FLJ22573 (FLJ22573),	-1.53768456	0.09359816
NM_020689	solute carrier family 24 (sodium/potassium/calcium exchanger), member 3 (SLC24A3),	-1.53679478	0.04027823
NM_174941	scavenger receptor cysteine-rich type 1 protein M160 (M160),	-1.53452987	0.57525496
NM_153229	hypothetical protein FLJ33318 (FLJ33318),	-1.53304325	0.29985125
NM_012445	spondin 2, extracellular matrix protein (SPON2),	-1.53145388	0.10744889
NM_000572	interleukin 10 (IL10),	-1.53143009	0.46967492
NM_002578	p21 (CDKN1A)-activated kinase 3 (PAK3),	-1.53113858	0.05080602
NM_001979	epoxide hydrolase 2, cytoplasmic (EPHX2),	-1.53001525	0.41241266
NM_007256	solute carrier organic anion transporter family, member 2B1 (SLCO2B1),	-1.52799625	0.0888538
NM_152365	hypothetical protein FLJ34633 (FLJ34633),	-1.52779311	1.04753321

CN646916	DSCR6	-1.52760549	0.3847249
CN644332	ARHB	-1.52626566	0.15708476
DV768382	NTRK2	-1.52605279	0.24224755
NM_004004	gap junction protein, beta 2, 26kDa (connexin 26) (GJB2),	-1.5243677	0.00041803
NM_020962	likely ortholog of mouse neighbor of Punc E11 (NOPE),	-1.52382576	0.03735416
NM_018346	radical S-adenosyl methionine domain containing 1 (RSAD1),	-1.5234121	0.41696734
XR_010248	CG17065-PA (LOC697051),	-1.52258094	0.09419909
XM_291139	RIKEN cDNA 9330196J05 (LOC340075),	-1.52205647	0.14476405
NM_007256	solute carrier organic anion transporter family, member 2B1 (SLCO2B1),	-1.52140495	0.12138001
NM_014010	astrotactin 2 (ASTN2), transcript variant 1,	-1.51888081	0.26782285
CO048892	Hs.406526	-1.51839123	0.18522741
NM_152486	sterile alpha motif domain containing 11 (SAMD11),	-1.51728668	0.26675427
NM_001446	fatty acid binding protein 7, brain (FABP7),	-1.5154149	0.14934694
NM_005052	ras-related C3 botulinum toxin substrate 3 (rho family, small GTP binding protein Rac3) (RAC3),	-1.51540775	0.0558085
NM_032812	plexin domain containing 2 (PLXDC2),	-1.51252499	0.02543435
NM_080415	peanut-like 2 (Drosophila) (PNUTL2), transcript variant 2,	-1.51247792	0.02219439
NM_004734	doublecortin and CaM kinase-like 1 (DCAMKL1),	-1.51224	0.0321411
NM_001007097	neurotrophic tyrosine kinase, receptor, type 2 (NTRK2), transcript variant b,	-1.51103446	0.07999086
NM_005615	ribonuclease, RNase A family, k6 (RNASE6),	-1.5103317	0.10399264
NM_001823	creatine kinase, brain (CKB),	-1.50924758	0.00895351
NM_002214	integrin, beta 8 (ITGB8),	-1.50900698	1.24733698
NM_015429	ABI gene family, member 3 (NESH) binding protein (ABI3BP),	-1.50348091	0.39619805
NM_002661	phospholipase C, gamma 2 (phosphatidylinositol-specific) (PLCG2),	-1.49958099	0.03092788
XR_014258	mannosidase, alpha, class 1C, member 1 (MAN1C1),	-1.49912856	0.11110945
DV768382	NTRK2	-1.49741691	0.12097677
NM_016610	toll-like receptor 8 (TLR8), transcript variant 1,	-1.49332404	0.79574734
NM_015675	growth arrest and DNA-damage-inducible, beta (GADD45B),	-1.49315244	0.08740284
NM_020416	protein phosphatase 2 (formerly 2A), regulatory subunit B (PR 52), gamma isoform (PPP2R2C), transcript variant 1,	-1.49145131	0.0772261
NM_004750	cytokine receptor-like factor 1 (CRLF1),	-1.49073712	0.17031928
NM_021223	myosin, light polypeptide 7, regulatory (MYL7),	-1.49002458	0.51457384
XR_012616	upregulated in colorectal cancer gene 1 protein precursor (LOC705348),	-1.48933235	0.29467422
NM_000093	collagen, type V, alpha 1 (COL5A1),	-1.4877351	0.02088755
CB230657	AGENCOURT_11469158 NICHD_Rh_Ov1 cDNA clone IMAGE:6883614 5', sequence	-1.4875079	0.00153082
NM_001012973	placenta-specific 9 (PLAC9),	-1.48665172	0.09207722
A_01_P018484	Unknown	-1.48597804	0.8346019
A_01_P006686	Unknown	-1.48595345	0.12715996
NM_145909	zinc finger protein 323 (ZNF323),	-1.48481682	0.0924218
NM_020962	likely ortholog of mouse neighbor of Punc E11 (NOPE),	-1.48479978	0.06830904
NM_003202	transcription factor 7 (T-cell specific, HMG-box) (TCF7), transcript variant 1,	-1.48464691	0.19476423
NM_173507	chromosome 1 open reading frame 127 (C1orf127),	-1.48448858	0.70746615
NM_002334	low density lipoprotein receptor-related protein 4 (LRP4),	-1.48258667	0.02599595
NM_030567	hypothetical protein MGC10772 (MGC10772),	-1.48250428	0.08113582

NM_004058	calcyphosine (CAPS), transcript variant 1,	-1.48100775	0.00434364
XR_010248	CG17065-PA (LOC697051),	-1.47988578	0.13917329
NM_018418	spermatogenesis associated 7 (SPATA7),	-1.4791747	0.08453314
XR_009973	RAB26, member RAS oncogene family (LOC695143),	-1.47874051	0.03755047
NM_000971	ribosomal protein L7 (RPL7),	-1.47692665	0.3183694
NM_018646	transient receptor potential cation channel, subfamily V, member 6 (TRPV6),	-1.47625093	0.46173932
NM_022047	differentially expressed in FDCP 6 homolog (mouse) (DEF6),	-1.4727843	0.03094668
NM_004405	distal-less homeo box 2 (DLX2),	-1.46905388	0.56606756
NM_00100201<br>7	host cell factor C1 regulator 1 (XPO1 dependant) (HCFC1R1), transcript variant 2,	-1.46847251	0.04073132
NM_025202	EF hand domain family, member D1 (EFHD1),	-1.46814189	0.97383431
NM_012292	minor histocompatibility antigen HA-1 (HA-1),	-1.46724198	0.2710309
NM_018326	GTPase, IMAP family member 4 (GIMAP4),	-1.46601986	0.13210475
NM_00100534<br>0	glycoprotein (transmembrane) nmb (GPNMB), transcript variant 1,	-1.45757623	0.19077046
NM_176798	pyrimidinergic receptor P2Y, G-protein coupled, 6 (P2RY6), transcript variant 2,	-1.45737544	0.4746192
NM_00100367<br>8	MGC4707 protein (MGC4707), transcript variant 4,	-1.45724526	1.35629025
CO580174	GPR34	-1.45631559	0.02407652
CO580174	GPR34	-1.45590167	0.13860574
NM_173462	papilin, proteoglycan-like sulfated glycoprotein (PAPLN),	-1.45587553	0.03563173
XR_012751	melanoma-associated chondroitin sulfate proteoglycan 4 (LOC713086), partial	-1.45506932	0.31279713
NM_006343	c-mer proto-oncogene tyrosine kinase (MERTK),	-1.44976372	0.16412803
NM_145716	single stranded DNA binding protein 3 (SSBP3),	-1.44749256	0.3107338
CN641482	NEFL	-1.44699454	0.55424143
NM_003118	secreted protein, acidic, cysteine-rich (osteonectin) (SPARC),	-1.44693186	0.15007881
NM_024711	human immune associated nucleotide 2 (hIAN2),	-1.44670051	0.74538142
CN642894	Hs.425023	-1.446404	0.04503689
NM_021643	tribbles homolog 2 (Drosophila) (TRIB2),	-1.44587308	0.02534298
NM_019605	SERTA domain containing 4 (SERTAD4),	-1.44517766	0.19505809
NM_003568	annexin A9 (ANXA9),	-1.44379023	0.17609278
NM_000495	collagen, type IV, alpha 5 (Alport syndrome) (COL4A5), transcript variant 1,	-1.44288017	0.30469016
NM_152499	hypothetical protein MGC45441 (MGC45441),	-1.44192646	0.6789219
NM_005398	protein phosphatase 1, regulatory (inhibitor) subunit 3C (PPP1R3C),	-1.44073597	0.14945191
NM_153703	podocan (PODN),	-1.43635519	0.1053111
NM_182571	hypothetical protein 284297 (FLJ35258),	-1.4355235	0.1265383
NM_152335	chromosome 15 open reading frame 27 (C15orf27),	-1.43497421	0.34488506
NM_145716	single stranded DNA binding protein 3 (SSBP3),	-1.43370204	0.11715797
NM_004822	netrin 1 (NTN1),	-1.43078138	0.71835995
NM_014485	prostaglandin D2 synthase, hematopoietic (PGDS),	-1.42708133	0.83522476
NM_015557	chromodomain helicase DNA binding protein 5 (CHD5),	-1.42600763	0.0811497
NM_003956	cholesterol 25-hydroxylase (CH25H),	-1.42381032	0.01652162
NM_032293	GTPase activating Rap/RanGAP domain-like 3 (GARNL3),	-1.42194108	0.15182747
NM_003713	phosphatidic acid phosphatase type 2B (PPAP2B), transcript variant 1,	-1.42111481	0.12047851
NM_024660	hypothetical protein FLJ22573 (FLJ22573),	-1.41996654	0.1640104
NM_004750	cytokine receptor-like factor 1 (CRLF1),	-1.41848856	0.03123021

NM_000120	epoxide hydrolase 1, microsomal (xenobiotic) (EPHX1),	-1.41774976	0.02238631
NM_002030	formyl peptide receptor-like 2 (FPRL2),	-1.41682501	0.32714775
XR_012553	IQ motif containing GTPase activating protein 2 (IQGAP2),	-1.41372482	0.12070436
NM_003713	phosphatidic acid phosphatase type 2B (PPAP2B), transcript variant 1,	-1.41310927	0.10396595
NM_032293	GTPase activating Rap/RanGAP domain-like 3 (GARNL3),	-1.4094827	0.00158656
NM_024922	esterase 31 (FLJ21736),	-1.40752941	1.10773183
NM_021572	ectonucleotide pyrophosphatase/phosphodiesterase 5 (putative function) (ENPP5),	-1.40686154	0.73126816
XR_014379	cell division cycle 25 homolog B (LOC717573),	-1.4068317	0.01573874
NM_000383	autoimmune regulator (autoimmune polyendocrinopathy candidiasis ectodermal dystrophy) (AIRE), transcript variant AIRE-1,	-1.40505798	0.75556737
NM_025045	hypothetical protein FLJ22582 (FLJ22582),	-1.40312521	0.02347094
XM_291277	hypothetical protein DKFZp761P0423 (DKFZp761P0423),	-1.40259765	1.10518276
A_01_P003596	Unknown	-1.40227076	1.12619947
A_01_P006686	Unknown	-1.40185882	0.12297848
NM_152658	THAP domain containing 8 (THAP8),	-1.40045362	0.02717169
NM_032637	S-phase kinase-associated protein 2 (p45) (SKP2), transcript variant 2,	-1.40041028	0.12212092
NM_004425	extracellular matrix protein 1 (ECM1), transcript variant 1,	-1.40036373	0.11130725
NM_013314	B-cell linker (BLNK),	-1.39996983	0.30502025
NM_004058	calcyphosine (CAPS), transcript variant 1,	-1.39707151	0.06268155
NM_004355	CD74 antigen (invariant polypeptide of major histocompatibility complex, class II antigen-associated) (CD74), transcript variant 2,	-1.39665448	0.2447721
NM_198278	hypothetical protein LOC255743 (LOC255743),	-1.395368	0.11179125
A_01_P006852	Unknown	-1.39431718	0.19389669
NM_002438	mannose receptor, C type 1 (MRC1),	-1.39386718	0.000461
NM_017573	proprotein convertase subtilisin/kexin type 4 (PCSK4),	-1.39213408	0.04774655
NM_00100591 2	inositol hexaphosphate kinase 2 (IHPK2), transcript variant 5,	-1.3903695	0.01156008
NM_018000	hypothetical protein FLJ10116 (FLJ10116),	-1.38951055	0.66946024
NM_007018	centrosomal protein 1 (CEP1),	-1.38821293	0.20561326
NM_003098	syntrophin, alpha 1 (dystrophin-associated protein A1, 59kDa, acidic component) (SNTA1),	-1.38685414	0.02071733
NM_145738	synaptogyrin 1 (SYNGR1), transcript variant 1c,	-1.38669323	0.12108657
NM_023002	hyaluronan and proteoglycan link protein 4 (HAPLN4),	-1.38633954	0.06873779
NM_000964	retinoic acid receptor, alpha (RARA),	-1.38571571	0.01060439
NM_024090	ELOVL family member 6, elongation of long chain fatty acids (FEN1/Elo2, SUR4/Elo3-like, yeast) (ELOVL6),	-1.38563415	0.21156604
NM_007261	CD300A antigen (CD300A),	-1.38446277	0.84724178
NM_001803	CDW52 antigen (CAMPATH-1 antigen) (CDW52),	-1.38368232	0.86870104
NM_144665	sestrin 3 (SESN3),	-1.38017788	0.0234246
NM_145738	synaptogyrin 1 (SYNGR1), transcript variant 1c,	-1.38017166	0.2447511
CO646712	IAN4L1	-1.37970269	0.23290603
NM_00100201 7	host cell factor C1 regulator 1 (XPO1 dependant) (HCFC1R1), transcript variant 2,	-1.37873551	0.30132707
XR_014258	mannosidase, alpha, class 1C, member 1 (MAN1C1),	-1.37789293	0.11728714
NM_014460	RNA-binding protein pippin (PIPPIN),	-1.37585044	0.42371516

NM_020770	cingulin (CGN),	-1.37437582	0.23102776
NM_020416	protein phosphatase 2 (formerly 2A), regulatory subunit B (PR 52), gamma isoform (PPP2R2C), transcript variant 1,	-1.37419205	0.98408786
XR_012910	4-aminobutyrate aminotransferase precursor (LOC714017),	-1.37397428	0.29555561
XM_046861	KRAB box containing C2H2 type zinc finger bA526D8.4 (BA526D8.4),	-1.37199063	0.75526573
NM_000093	collagen, type V, alpha 1 (COL5A1),	-1.37060049	0.24159179
XR_010456	keratin 19 (LOC698425),	-1.370473	0.19109713
NM_004036	adenylate cyclase 3 (ADCY3),	-1.37046428	0.28182152
NM_145798	oxysterol binding protein-like 7 (OSBPL7), transcript variant 1,	-1.36805279	0.06911177
NM_015429	ABI gene family, member 3 (NESH) binding protein (ABI3BP),	-1.3670007	0.21895458
NM_003256	tissue inhibitor of metalloproteinase 4 (TIMP4),	-1.3652381	0.02713972
NM_006340	BAI1-associated protein 2 (BAIAP2), transcript variant 3,	-1.36498033	0.15367854
NM_198278	hypothetical protein LOC255743 (LOC255743),	-1.36431887	0.05169802
XR_014749	calcium channel, voltage-dependent, alpha 1H subunit (CACNA1H),	-1.36404519	0.01989598
NM_003118	secreted protein, acidic, cysteine-rich (osteonectin) (SPARC),	-1.36400903	0.14471159
NM_144665	sestrin 3 (SESN3),	-1.36348145	0.29111027
NM_016602	G protein-coupled receptor 2 (GPR2),	-1.36329427	0.41766924
NM_004425	extracellular matrix protein 1 (ECM1), transcript variant 1,	-1.36313514	0.158895
NM_002905	retinol dehydrogenase 5 (11-cis and 9-cis) (RDH5),	-1.36140491	0.40970909
NM_181481	chromosome 18 open reading frame 1 (C18orf1), transcript variant a1,	-1.36131435	0.05475698
NM_004185	wingless-type MMTV integration site family, member 2B (WNT2B), transcript variant WNT-2B1,	-1.35665502	0.10704497
NM_145867	leukotriene C4 synthase (LTC4S), transcript variant 1,	-1.35636596	0.10358819
XM_291139	RIKEN cDNA 9330196J05 (LOC340075),	-1.35398604	0.39866612
NM_017918	hypothetical protein FLJ20647 (FLJ20647),	-1.35171529	0.23345662
NM_006928	silver homolog (mouse) (SILV),	-1.35096668	0.15967437
NM_182507	hypothetical protein LOC144501 (LOC144501),	-1.35021304	0.19643439
NM_006866	leukocyte immunoglobulin-like receptor, subfamily A (with TM domain), member 2 (LILRA2),	-1.3489557	0.16863983
NM_145867	leukotriene C4 synthase (LTC4S), transcript variant 1,	-1.34834868	0.22484476
NM_014800	engulfment and cell motility 1 (ced-12 homolog, C. elegans) (ELMO1), transcript variant 1,	-1.34279798	0.83923951
NM_138959	vang-like 1 (van gogh, Drosophila) (VANGL1),	-1.34277326	1.11523236
NM_004036	adenylate cyclase 3 (ADCY3),	-1.34232695	0.13741059
NM_004756	numb homolog (Drosophila)-like (NUMBL),	-1.34206374	0.10173909
NM_138804	hypothetical protein BC014602 (LOC130951),	-1.33757203	0.07261575
NM_003873	neuropilin 1 (NRP1),	-1.33714851	0.06366468
NM_178232	hyaluronan and proteoglycan link protein 3 (HAPLN3),	-1.33703552	0.05222372
NM_014351	sulfotransferase family 4A, member 1 (SULT4A1), transcript variant 1,	-1.33686629	0.64780094
XR_014213	tenascin XB isoform 1 (LOC716998),	-1.33432468	0.09996025
NM_153685	hypothetical protein DKFZp547D2210 (DKFZp547D2210),	-1.33288296	0.01534339
NM_152527	solute carrier family 16 (monocarboxylic acid transporters), member 14 (SLC16A14),	-1.33056148	0.07774072
NM_030926	integral membrane protein 2C (ITM2C),	-1.33034012	0.04739404
NM_016602	G protein-coupled receptor 2 (GPR2),	-1.33026553	0.01033399
NM_003063	sarcolipin (SLN),	-1.33017827	0.13140524
NM_152365	hypothetical protein FLJ34633 (FLJ34633),	-1.33010778	0.54038751
NM_173462	papilin, proteoglycan-like sulfated glycoprotein (PAPLN),	-1.33002678	0.23784609
NM_173587	REST corepressor 2 (RCOR2),	-1.32936555	0.13524836
NM_013276	carbohydrate kinase-like (CARKL),	-1.32888777	0.28691077
NM_003279	troponin C2, fast (TNNC2),	-1.32885878	0.182857

NM_020689	solute carrier family 24 (sodium/potassium/calcium exchanger), member 3 (SLC24A3),	-1.32871909	0.19742416
NM_005879	TRAF interacting protein (TRIP),	-1.3284099	0.25641248
NM_182964	neuron navigator 2 (NAV2), transcript variant 1,	-1.32804756	0.10063049
NM_000218	potassium voltage-gated channel, KQT-like subfamily, member 1 (KCNQ1), transcript variant 1,	-1.32781774	0.04133825
A_01_P013100	Unknown	-1.32606276	0.80838835
NM_006475	periostin, osteoblast specific factor (POSTN),	-1.32602426	0.12761453
NM_001974	egf-like module containing, mucin-like, hormone receptor-like 1 (EMR1),	-1.32566505	1.01595718
NM_031469	SH3 domain binding glutamic acid-rich protein like 2 (SH3BGRL2),	-1.32546824	0.23826632
NM_199077	cyclin M2 (CNNM2), transcript variant 3,	-1.32294557	0.52303068
NM_006697	myotubularin related protein 11 (MTMR11),	-1.3227237	0.18954856
NM_020428	CTL2 protein (CTL2),	-1.32192476	0.07077156
NM_032369	hypothetical protein MGC15619 (MGC15619),	-1.32178773	0.63144257
NM_018346	radical S-adenosyl methionine domain containing 1 (RSAD1),	-1.32136855	0.42528862
NM_017918	hypothetical protein FLJ20647 (FLJ20647),	-1.32064125	0.1121338
NM_014149	HSPC049 protein (HSPC049),	-1.31877704	0.03496943
NM_004657	serum deprivation response (phosphatidylserine binding protein) (SDPR),	-1.31786674	0.44632908
NM_000155	galactose-1-phosphate uridylyltransferase (GALT), transcript variant 1,	-1.31775279	0.06483523
NM_002438	mannose receptor, C type 1 (MRC1),	-1.31736361	0.5383806
NM_018204	cytoskeleton associated protein 2 (CKAP2),	-1.31651825	0.21398145
CK231501	ILLUMIGEN_MCQ_2353 Katze_MMLG cDNA 5' human Unigene Hs.417764, sequence	-1.31585939	0.06150358
NM_001482	glycine amidinotransferase (L-arginine:glycine amidinotransferase) (GATM),	-1.31130606	0.03388669
NM_144620	hypothetical protein MGC14816 (MGC14816),	-1.31007177	0.12482056
NM_013356	solute carrier 16 (monocarboxylic acid transporters), member 8 (SLC16A8),	-1.30715467	0.31161146
NM_005619	reticulon 2 (RTN2), transcript variant 1,	-1.30653638	0.05743317
NM_138393	chromosome 19 open reading frame 32 (C19orf32),	-1.30552739	0.12956066
NM_003956	cholesterol 25-hydroxylase (CH25H),	-1.30508712	0.15665364
XM_375720	regulating synaptic membrane exocytosis 3 (RIMS3),	-1.30437483	0.87065567
NM_182520	chromosome 22 open reading frame 15 (C22orf15),	-1.30400182	0.27286294
NM_007233	TP53 activated protein 1 (TP53AP1),	-1.30389565	0.92022384
NM_014485	prostaglandin D2 synthase, hematopoietic (PGDS),	-1.30174799	0.01915349
XM_035371	zinc finger, FYVE domain containing 28 (ZFYVE28),	-1.30168964	0.05477755
NM_001979	epoxide hydrolase 2, cytoplasmic (EPHX2),	-1.30147825	0.20515561
NM_006080	sema domain, immunoglobulin domain (Ig), short basic domain, secreted, (semaphorin) 3A (SEMA3A),	-1.30143029	0.29547731
NM_015976	sorting nexin 7 (SNX7), transcript variant 1,	-1.30054929	0.03416535
NM_182507	hypothetical protein LOC144501 (LOC144501),	-1.30020139	0.12293656
NM_020356	chromosome 20 open reading frame 32 (C20orf32),	-1.29957511	0.96729106
NM_001532	solute carrier family 29 (nucleoside transporters), member 2 (SLC29A2),	-1.29949395	0.13057234
NM_178423	histone deacetylase 9 (HDAC9), transcript variant 4,	-1.29944219	0.0063743
CK232488	PSG4	-1.29896996	0.0878751
NM_018670	mesoderm posterior 1 (MESP1),	-1.29867999	0.91992296
NM_018326	GTPase, IMAP family member 4 (GIMAP4),	-1.2981445	0.11962671
NM_000405	GM2 ganglioside activator (GM2A),	-1.29749922	0.10152542
NM_004756	numb homolog (Drosophila)-like (NUMBL),	-1.29749346	0.05440582
NM_020415	resistin (RETN),	-1.29664061	0.40344574
NM_152328	adenylosuccinate synthase like 1 (ADSSL1), transcript variant 2,	-1.29484115	0.14912162

NM_003617	regulator of G-protein signalling 5 (RGS5),	-1.29394161	0.01429588
NM_004107	Fc fragment of IgG, receptor, transporter, alpha (FCGRT),	-1.29283719	0.06461062
CN646916	DSCR6	-1.29253086	0.20452386
NM_145867	leukotriene C4 synthase (LTC4S), transcript variant 1,	-1.29221345	0.33416378
NM_00100367<br>6	MGC4707 protein (MGC4707), transcript variant 1,	-1.29131084	0.40085268
NM_000218	potassium voltage-gated channel, KQT-like subfamily, member 1 (KCNQ1), transcript variant 1,	-1.29116818	0.03517283
NM_000850	glutathione S-transferase M4 (GSTM4), transcript variant 1,	-1.28992086	0.02282453
NM_014216	inositol 1,3,4-triphosphate 5/6 kinase (ITPK1),	-1.28820722	0.10874426
NM_032868	hypothetical protein FLJ14981 (FLJ14981),	-1.28804614	0.13993503
NM_153703	podocan (PODN),	-1.28752209	0.02376832
NM_015981	calcium/calmodulin-dependent protein kinase (CaM kinase) II alpha (CAMK2A), transcript variant 1,	-1.28622882	0.05784039
NM_001232	calsequestrin 2 (cardiac muscle) (CASQ2),	-1.28615605	0.65219968
NM_006848	hepatitis delta antigen-interacting protein A (DIPA),	-1.28549455	0.02384536
NM_020650	reticulocalbin 3, EF-hand calcium binding domain (RCN3),	-1.28491204	0.37288432
NM_012276	leukocyte immunoglobulin-like receptor, subfamily A (without TM domain), member 4 (ILT7),	-1.28460457	0.16406729
DQ155428	LILRAb complete cds	-1.281771	0.30187786
NM_005619	reticulon 2 (RTN2), transcript variant 1,	-1.2817304	0.21302793
NM_004355	CD74 antigen (invariant polypeptide of major histocompatibility complex, class II antigen-associated) (CD74), transcript variant 2,	-1.28101588	0.13018804
NM_182574	hypothetical protein FLJ36070 (FLJ36070),	-1.28058065	0.46654429
NM_014010	astrotactin 2 (ASTN2), transcript variant 1,	-1.28000464	0.28982354
NM_018684	KIAA1166 (KIAA1166),	-1.27693685	0.1971678
NM_145728	desmuslin (DMN), transcript variant A,	-1.27666506	0.33720672
NM_145867	leukotriene C4 synthase (LTC4S), transcript variant 1,	-1.27663204	0.05005582
NM_000224	keratin 18 (KRT18), transcript variant 1,	-1.27543884	0.17681795
NM_000592	complement component 4B (C4B),	-1.27441276	0.0098275
CN645572	C6orf62	-1.27351044	0.37435178
NM_020632	ATPase, H+ transporting, lysosomal V0 subunit a isoform 4 (ATP6V0A4), transcript variant 1,	-1.27309576	0.61072222
NM_030926	integral membrane protein 2C (ITM2C),	-1.27300934	0.08679429
NM_173653	solute carrier family 9 (sodium/hydrogen exchanger), isoform 9 (SLC9A9),	-1.27265612	0.03800287
NM_144505	kallikrein 8 (neuropsin/ovasin) (KLK8), transcript variant 2,	-1.27133489	0.11363666
NM_198472	chromosome 10 open reading frame 125 (C10orf125),	-1.27098414	0.23573417
NM_032648	hypothetical protein MGC10820 (MGC10820),	-1.27000682	0.46643629
CB311278	AGENCOURT_11616400 NICHD_Rh_Ov1 cDNA clone IMAGE:6915394 5', sequence	-1.2699921	0.13931647
NM_016593	cytochrome P450, family 39, subfamily A, polypeptide 1 (CYP39A1),	-1.26955674	0.00318972
NM_002517	neuronal PAS domain protein 1 (NPAS1),	-1.26871545	1.15733951
NM_015675	growth arrest and DNA-damage-inducible, beta (GADD45B),	-1.26569409	0.11314485
NM_030568	chromosome 6 open reading frame 148 (C6orf148),	-1.26479337	0.39361823
CO580739	C1QG	-1.26293868	0.30772413
NM_014571	hairy/enhancer-of-split related with YRPW motif-like (HEYL),	-1.26266767	0.23169254
NM_153486	lactate dehydrogenase D (LDHD), nuclear gene encoding mitochondrial protein, transcript variant 1,	-1.26265161	1.06559859
NM_152328	adenylosuccinate synthase like 1 (ADSSL1), transcript variant 2,	-1.26244473	0.01565136
NM_000407	glycoprotein Ib (platelet), beta polypeptide (GP1BB),	-1.26113282	0.03272318
NM_012101	tripartite motif-containing 29 (TRIM29), transcript variant 1,	-1.25985191	0.55243153

NM_00100591<br>2	inositol hexaphosphate kinase 2 (IHPK2), transcript variant 5,	-1.25874794	0.19694361
NM_032637	S-phase kinase-associated protein 2 (p45) (SKP2), transcript variant 2,	-1.25831473	0.29342578
NM_001532	solute carrier family 29 (nucleoside transporters), member 2 (SLC29A2),	-1.2575856	0.04942627
XR_011531	hypothetical protein LOC706118 (LOC706118),	-1.25672487	0.10222583
NM_182574	hypothetical protein FLJ36070 (FLJ36070),	-1.25662607	0.24521041
NM_001853	collagen, type IX, alpha 3 (COL9A3),	-1.25647387	1.0485263
NM_025228	TRAF3-interacting Jun N-terminal kinase (JNK)-activating modulator (T3JAM),	-1.25523391	0.73390072
NM_006329	fibulin 5 (FBLN5),	-1.25503573	0.19243677
NM_033128	scinderin (SCIN),	-1.25481951	0.08546467
CK232488	PSG4	-1.25416741	0.09630082
NM_004840	Rac/Cdc42 guanine nucleotide exchange factor (GEF) 6 (ARHGEF6),	-1.25369745	0.16133252
NM_033064	ataxia, cerebellar, Cayman type (caytaxin) (ATCAY),	-1.25346078	1.05390329
NM_003617	regulator of G-protein signalling 5 (RGS5),	-1.25310272	0.06946824
NM_023915	G protein-coupled receptor 87 (GPR87),	-1.25177607	0.4124271
NM_014216	inositol 1,3,4-triphosphate 5/6 kinase (ITPK1),	-1.25142638	0.0900177
NM_173469	hypothetical protein LOC92912 (LOC92912),	-1.2503988	0.03022843
DR766716	ANK2	-1.24888302	0.00938845
CB554423	MMSP0024_G06 MMSP cDNA, sequence	-1.24733461	0.99318142
NM_173653	solute carrier family 9 (sodium/hydrogen exchanger), isoform 9 (SLC9A9),	-1.24662092	0.27059962
NM_206918	degenerative spermatocyte homolog 2, lipid desaturase (Drosophila) (DEGS2),	-1.2464266	0.34454968
NM_030568	chromosome 6 open reading frame 148 (C6orf148),	-1.24533701	0.27870025
NM_00100191<br>7	olfactory receptor, family 56, subfamily A, member 1 (OR56A1),	-1.24528443	0.71629261
NM_178154	fucosyltransferase 8 (alpha (1,6) fucosyltransferase) (FUT8), transcript variant 2,	-1.24433914	0.25174514
NM_005559	laminin, alpha 1 (LAMA1),	-1.24401274	0.08644948
NM_005252	v-fos FBJ murine osteosarcoma viral oncogene homolog (FOS),	-1.24287035	0.32260269
NM_014696	KIAA0514 (KIAA0514),	-1.24219402	0.05923562
NM_000616	CD4 antigen (p55) (CD4),	-1.24139153	0.23668386
NM_199248	calcium channel, voltage-dependent, beta 1 subunit (CACNB1), transcript variant 3,	-1.23878238	0.01130786
NM_021223	myosin, light polypeptide 7, regulatory (MYL7),	-1.23823035	0.16463032
NM_020428	CTL2 protein (CTL2),	-1.23743739	0.06796562
NM_002576	p21/Cdc42/Rac1-activated kinase 1 (STE20 homolog, yeast) (PAK1),	-1.23700798	0.04800663
DR771261	RARRES2	-1.23528562	0.10094888
NM_003023	SH3-domain binding protein 2 (SH3BP2),	-1.23515761	0.02167505
NM_006848	hepatitis delta antigen-interacting protein A (DIPA),	-1.23454187	0.1116403
NM_138967	secretory carrier membrane protein 5 (SCAMP5),	-1.23407286	0.26304174
NM_174941	scavenger receptor cysteine-rich type 1 protein M160 (M160),	-1.23402554	0.29427714
NM_014396	vacuolar protein sorting 41 (yeast) (VPS41), transcript variant 1,	-1.23351032	0.39630369
CO725791	PXMP2	-1.23243191	0.00931489
NM_144699	ATPase, Na+/K+ transporting, alpha 4 polypeptide (ATP1A4),	-1.23219448	0.91767407
NM_030765	UDP-GlcNAc:betaGal beta-1,3-N-acetylglucosaminyltransferase 4 (B3GNT4),	-1.23161249	0.13741368
NM_033086	FYVE, RhoGEF and PH domain containing 3 (FGD3),	-1.23110571	0.07296307
XM_168060	chromosome 6 open reading frame 154 (C6orf154),	-1.22926022	0.05309858
NM_021201	membrane-spanning 4-domains, subfamily A, member 7 (MS4A7), transcript variant 1,	-1.22872178	0.39013732
NM_207112	hydroxyacylglutathione hydrolase-like (HAGHL), transcript variant 1,	-1.22851845	0.16905191

NM_00101297<br>3	placenta-specific 9 (PLAC9),	-1.22734213	0.02772345
NM_033421	chromosome 20 open reading frame 161 (C20orf161), transcript variant 1,	-1.22351902	0.34166036
NM_018430	translin-associated factor X interacting protein 1 (TSNAXIP1),	-1.22329851	0.92224651
NM_145728	desmuslin (DMN), transcript variant A,	-1.2225982	0.34698094
XR_014094	Notch homolog 3 (NOTCH3),	-1.22162735	0.33012754
XR_009904	Vitamin K-dependent protein S precursor (LOC694845),	-1.22067503	0.1655542
NM_014726	ProSAPiP2 protein (ProSAPiP2),	-1.21962045	0.11055488
CB311278	AGENCOURT_11616400 NICHD_Rh_Ov1 cDNA clone IMAGE:6915394 5', sequence	-1.21879587	0.13243132
NM_000426	laminin, alpha 2 (merosin, congenital muscular dystrophy) (LAMA2),	-1.21828464	0.24062296
NM_001884	hyaluronan and proteoglycan link protein 1 (HAPLN1),	-1.21825635	0.82577653
NM_173619	hypothetical protein MGC34761 (MGC34761),	-1.21712615	0.14638784
NM_175061	juxtaposed with another zinc finger gene 1 (JAZF1),	-1.21672486	0.40042533
NM_175738	RAB37, member RAS oncogene family (RAB37),	-1.21625916	0.76957191
DR771261	RARRES2	-1.21413152	0.19617104
NM_006844	ilvB (bacterial acetolactate synthase)-like (ILVBL), transcript variant 1,	-1.21399162	0.05934525
XR_014379	cell division cycle 25 homolog B (LOC717573),	-1.21356242	0.01358845
NM_016228	aminoadipate aminotransferase (AADAT), transcript variant 1,	-1.21315593	0.48610737
NM_007375	TAR DNA binding protein (TARDBP),	-1.21235537	0.47145661
NM_198232	ribonuclease, RNase A family, 1 (pancreatic) (RNASE1), transcript variant 3,	-1.21231139	0.51995581
NM_145798	oxysterol binding protein-like 7 (OSBPL7), transcript variant 1,	-1.21217344	0.08071696
NM_018418	spermatogenesis associated 7 (SPATA7),	-1.21205429	0.10700298
NM_145176	solute carrier family 2 (facilitated glucose transporter), member 12 (SLC2A12),	-1.21124752	0.19526906
NM_000964	retinoic acid receptor, alpha (RARA),	-1.20980912	0.1025397
NM_006982	cartilage paired-class homeoprotein 1 (CART1),	-1.20842157	0.24267046
NM_00103525<br>4	family with sequence similarity 102, member A (FAM102A), transcript variant 1,	-1.20774067	0.00700366
NM_022109	CDW92 antigen (CDW92),	-1.20719112	0.03866027
NM_002630	progastricsin (pepsinogen C) (PGC),	-1.20488571	1.18228658
NM_052844	WD repeat domain 34 (WDR34),	-1.20359245	0.07713696
NM_018557	low density lipoprotein-related protein 1B (deleted in tumors) (LRP1B),	-1.20178621	0.07010231
XR_011045	tubulin, beta, 4 (TUBB3),	-1.20147021	0.18019024
NM_005398	protein phosphatase 1, regulatory (inhibitor) subunit 3C (PPP1R3C),	-1.20114499	0.25672281
AB075502	neuroblastoma cDNA, clone:Nbla00237, full insert sequence	-1.20012988	0.1388077
XR_012167	leucine rich repeat neuronal 3 (LOC701932),	-1.19985478	0.28497712
NM_006697	cisplatin resistance associated (CRA),	-1.19974682	0.05218248
NM_013974	dimethylarginine dimethylaminohydrolase 2 (DDAH2),	-1.19963868	0.13121033
NM_173587	REST corepressor 2 (RCOR2),	-1.19796376	0.29804559
NM_000667	alcohol dehydrogenase 1A (class I), alpha polypeptide (ADH1A),	-1.19787564	0.00861416
NM_032256	hypothetical protein DKFZp434K2435 (DKFZp434K2435),	-1.19688329	0.19666098
NM_206808	citrate lyase beta like (CLYBL), transcript variant 2,	-1.19554087	0.35616495
NM_005046	kallikrein 7 (chymotryptic, stratum corneum) (KLK7), transcript variant 1,	-1.19442068	0.07747783
NM_000478	alkaline phosphatase, liver/bone/kidney (ALPL),	-1.1942463	0.03252076
NM_052880	HGFL gene (MGC17330),	-1.19392299	0.27002931
XR_012701	hypothetical protein LOC707218 (LOC707218),	-1.19377485	0.743409
NM_000214	jagged 1 (Alagille syndrome) (JAG1),	-1.19274123	0.25813416

NM_033183	chorionic gonadotropin, beta polypeptide 8 (CGB8),	-1.19097044	0.86803887
NM_004107	Fc fragment of IgG, receptor, transporter, alpha (FCGRT),	-1.19047131	0.00907452
NM_145234	chordin-like 1 (CHRDL1),	-1.19003979	0.09873874
CN644408	COL4A1	-1.18962475	0.01218802
NM_153614	DnaJ (Hsp40) related, subfamily B, member 13 (DNAJB13),	-1.1888025	0.91303006
NM_144691	calpain 12 (CAPN12),	-1.18869023	0.04093587
NM_033549	tripartite motif-containing 41 (TRIM41), transcript variant 1,	-1.18758412	0.97710581
NM_181711	GRP1 (general receptor for phosphoinositides 1)-associated scaffold protein (GRASP),	-1.1869817	0.1103977
XR_012522	spectrin repeat containing, nuclear envelope 2 isoform e (LOC706759),	-1.18677359	0.15868252
NM_152732	chromosome 6 open reading frame 206 (C6orf206),	-1.18663376	0.17392718
NM_032868	hypothetical protein FLJ14981 (FLJ14981),	-1.18494518	0.09831658
NM_031910	C1q and tumor necrosis factor related protein 6 (C1QTNF6), transcript variant 1,	-1.18466728	0.67289616
NM_000155	galactose-1-phosphate uridylyltransferase (GALT), transcript variant 1,	-1.18431588	0.18955214
NM_024722	acyl-Coenzyme A binding domain containing 4 (ACBD4),	-1.18399272	0.00397612
NM_181711	GRP1 (general receptor for phosphoinositides 1)-associated scaffold protein (GRASP),	-1.18389127	0.16100985
NM_004693	cytokeratin type II (K6HF),	-1.18314959	0.05801654
NM_198540	UDP-Gal:betaGal beta 1,3-galactosyltransferase polypeptide 7 (B3GALT7),	-1.18108734	0.35569031
NM_152619	doublecortin and CaM kinase-like 2 (DCAMKL2),	-1.18049357	0.48069007
NM_052844	WD repeat domain 34 (WDR34),	-1.1801455	0.12880122
NM_00101188<br>0	secretory protein LOC497190 (LOC497190),	-1.17951262	0.94685358
NM_015964	brain specific protein (CGI-38),	-1.17888199	0.21148203
NM_030756	transcription factor 7-like 2 (T-cell specific, HMG-box) (TCF7L2),	-1.17821766	0.09787572
NM_000405	GM2 ganglioside activator (GM2A),	-1.17793278	0.00071588
NM_001512	glutathione S-transferase A4 (GSTA4),	-1.17704916	0.17513588
NM_020201	5',3'-nucleotidase, mitochondrial (NT5M), nuclear gene encoding mitochondrial protein,	-1.17563227	0.11246544
NM_014010	astrotactin 2 (ASTN2), transcript variant 1,	-1.17538641	0.25885498
NM_198563	RIKEN cDNA 1810038N08 gene (MGC52022),	-1.17501975	0.20244735
A_01_P011695	Unknown	-1.17489662	0.10541893
NM_032192	protein phosphatase 1, regulatory (inhibitor) subunit 1B (dopamine and cAMP regulated phosphoprotein, DARPP-32) (PPP1R1B),	-1.17362842	0.35003654
NM_005865	protease, serine, 16 (thymus) (PRSS16),	-1.17219536	1.0355989
NM_003808	tumor necrosis factor (ligand) superfamily, member 13 (TNFSF13), transcript variant alpha,	-1.17170199	0.17124695
NM_018710	hypothetical protein DKFZp762O076 (DKFZp762O076),	-1.17137309	0.56849529
NM_017766	hypothetical protein FLJ20321 (FLJ20321),	-1.17134802	0.31055904
XR_014043	Ras association domain family 2 (RASSF2),	-1.17123773	0.05535246
NM_138290	Rap2-binding protein 9 (RPIB9),	-1.17108688	0.02038056
NM_006697	myotubularin related protein 11 (MTMR11),	-1.17097816	0.05226351
NM_001400	endothelial differentiation, sphingolipid G-protein-coupled receptor, 1 (EDG1),	-1.17051505	0.48316355
NM_182647	opiate receptor-like 1 (OPRL1), transcript variant 1,	-1.17044962	0.84546698
DQ148184	clone ss1_g14_t7_405 zinc finger protein 561 (ZNF561) partial cds	-1.16970632	0.37671911
NM_001776	ectonucleoside triphosphate diphosphohydrolase 1 (ENTPD1),	-1.16804264	0.16725479
NM_018376	nipsnap homolog 3B (C. elegans) (NIPSNAP3B),	-1.16767708	0.00995137
NM_153486	lactate dehydrogenase D (LDHD), nuclear gene encoding mitochondrial protein, transcript variant 1,	-1.16711472	0.6997389
NM_033102	prostate cancer associated protein 6 (PCANAP6),	-1.1664433	0.24047124

NM_144621	zinc finger and BTB domain containing 8 (ZBTB8),	-1.16545951	0.03118134
NM_024711	human immune associated nucleotide 2 (hIAN2),	-1.16544982	0.12742666
NM_003019	surfactant, pulmonary-associated protein D (SFTPD),	-1.16526312	0.73305552
NM_178827	hypothetical protein FLJ35834 (FLJ35834),	-1.16474549	0.27980485
NM_002576	p21/Cdc42/Rac1-activated kinase 1 (STE20 homolog, yeast) (PAK1),	-1.16372555	0.15532377
NM_014661	KIAA0140 (KIAA0140),	-1.16350636	0.02290833
CO725791	PXMP2	-1.16346379	0.07648212
NM_033046	rhotekin (RTKN), transcript variant 2,	-1.16313115	0.07726161
NM_012109	chromosome 19 open reading frame 4 (C19orf4),	-1.16230959	0.10012763
XR_012553	IQ motif containing GTPase activating protein 2 (IQGAP2),	-1.16187502	0.4795867
NM_014550	caspase recruitment domain family, member 10 (CARD10),	-1.16157886	0.26601688
NM_018317	hypothetical protein FLJ11082 (FLJ11082),	-1.16122541	0.1537559
XR_014235	Ras-related protein Rab-40B (SOCS box-containing protein RAR) (Rar protein) (LOC719629),	-1.16053529	0.25093807
NM_213608	IIDS6411 (UNQ6411),	-1.16006582	0.05949455
NM_173847	sperm acrosome associated 3 (SPACA3),	-1.15937115	0.80566145
XM_372391	olfactory receptor, family 8, subfamily H, member 3 (OR8H3),	-1.15912619	0.01737151
NM_152335	chromosome 15 open reading frame 27 (C15orf27),	-1.15788836	0.03458173
NM_015976	sorting nexin 7 (SNX7), transcript variant 1,	-1.15780734	0.24935508
NM_001290	LIM domain binding 2 (LDB2),	-1.1562615	0.10727881
NM_030756	transcription factor 7-like 2 (T-cell specific, HMG-box) (TCF7L2),	-1.15571491	0.12376566
NM_015886	protease inhibitor 15 (PI15),	-1.15538523	0.85566064
XR_010573	citron (LOC703199),	-1.15468563	0.11056121
XR_009904	Vitamin K-dependent protein S precursor (LOC694845),	-1.15417023	0.00213032
NM_003279	troponin C2, fast (TNNC2),	-1.15295878	0.70590316
NM_022819	phospholipase A2, group IIF (PLA2G2F),	-1.15260824	0.53418992
NM_206808	citrate lyase beta like (CLYBL), transcript variant 2,	-1.15037224	0.05120969
NM_139056	a disintegrin-like and metalloprotease (reprolysin type) with thrombospondin type 1 motif, 16 (ADAMTS16),	-1.15010552	0.52271574
NM_178232	hyaluronan and proteoglycan link protein 3 (HAPLN3),	-1.14919666	0.73287912
NM_199248	calcium channel, voltage-dependent, beta 1 subunit (CACNB1), transcript variant 3,	-1.14873069	0.1152332
NM_024579	hypothetical protein FLJ23221 (FLJ23221),	-1.14805281	0.15162535
NM_017766	hypothetical protein FLJ20321 (FLJ20321),	-1.14735575	0.03255668
NM_033128	scinderin (SCIN),	-1.1472431	0.41186059
NM_001847	collagen, type IV, alpha 6 (COL4A6), transcript variant A,	-1.14710699	0.31239674
NM_024532	PF20 (PF20),	-1.14686813	0.71009781
NM_000955	prostaglandin E receptor 1 (subtype EP1), 42kDa (PTGER1),	-1.14561608	0.69105819
NM_018937	protocadherin beta 3 (PCDHB3),	-1.14409153	0.09069389
NM_199336	hypothetical protein DKFZp434N062 (DKFZp434N062),	-1.14384932	0.01909131
NM_003256	tissue inhibitor of metalloproteinase 4 (TIMP4),	-1.14237316	0.17657715
CO580739	C1QG	-1.14140682	0.07998543
NM_017882	ceroid-lipofuscinosis, neuronal 6, late infantile, variant (CLN6),	-1.14042426	0.59233309
NM_006174	neuropeptide Y receptor Y5 (NPY5R),	-1.13879923	0.04611818
XR_013101	EVIN1 (LOC714977),	-1.13747909	0.02405403
NM_134266	solute carrier family 26, member 7 (SLC26A7), transcript variant 2,	-1.1373905	0.47111779
NM_052858	MARVEL domain containing 3 (MARVELD3),	-1.13703213	0.73999442
NM_024006	vitamin K epoxide reductase complex, subunit 1 (VKORC1), transcript variant 1,	-1.13678598	0.10124701
NM_030663	mitochondrial capsule selenoprotein (MCSP), nuclear gene encoding mitochondrial protein,	-1.13621307	0.69389778

NM_016373	WW domain containing oxidoreductase (WWOX), transcript variant 1,	-1.13550147	0.21968929
XR_011133	hypothetical protein LOC700625 (LOC700625),	-1.13516049	0.20128426
NM_002589	BH-protocadherin (brain-heart) (PCDH7), transcript variant a,	-1.13470568	0.51416132
NM_033036	galactose-3-O-sulfotransferase 3 (GAL3ST3),	-1.13403736	0.00147262
XR_009750	Cathepsin Z precursor (Cathepsin X) (Cathepsin P) (LOC694157),	-1.13239717	0.10008645
NM_138804	hypothetical protein BC014602 (LOC130951),	-1.13073179	0.09958748
NM_003505	frizzled homolog 1 (Drosophila) (FZD1),	-1.13031209	0.00011489
NM_032414	prokineticin 1 (PROK1),	-1.12996757	0.7345007
NM_005613	regulator of G-protein signalling 4 (RGS4),	-1.12986644	0.2790306
NM_002528	nth endonuclease III-like 1 (E. coli) (NTHL1),	-1.12967107	0.08057878
NM_00100380<br>1	SWI/SNF related, matrix associated, actin dependent regulator of chromatin, subfamily d, member 3 (SMARCD3), transcript variant 3,	-1.127172	0.04471661
NM_014942	ankyrin repeat domain 6 (ANKRD6),	-1.12705873	0.21336519
NM_145170	tetratricopeptide repeat domain 18 (TTC18),	-1.12678669	0.55132992
NM_007112	thrombospondin 3 (THBS3),	-1.12641004	0.09611015
DR766716	ANK2	-1.12628679	0.00718763
NM_007352	elastase 3B, pancreatic (ELA3B),	-1.1253934	0.60244693
NM_005572	lamin A/C (LMNA), transcript variant 2,	-1.12488338	0.14140511
XR_009750	Cathepsin Z precursor (Cathepsin X) (Cathepsin P) (LOC694157),	-1.12465743	0.03017249
NM_030582	collagen, type XVIII, alpha 1 (COL18A1), transcript variant 1,	-1.12309028	0.09045535
NM_015271	tripartite motif-containing 2 (TRIM2),	-1.12293572	0.88003363
CN646050	Hs.432799	-1.12269056	0.19696916
NM_012101	tripartite motif-containing 29 (TRIM29), transcript variant 1,	-1.12183063	0.21689477
NM_000851	glutathione S-transferase M5 (GSTM5),	-1.12171705	0.03318138
NM_002287	leukocyte-associated Ig-like receptor 1 (LAIR1), transcript variant a,	-1.12114516	0.27355588
NM_004952	ephrin-A3 (EFNA3),	-1.12111037	0.21662602
XM_496306	chromosome 19 open reading frame 14 (C19orf14),	-1.12070344	0.19655383
NM_015896	zinc finger, MYND-type containing 10 (ZMYND10),	-1.12044281	0.50203553
NM_018215	hypothetical protein FLJ10781 (FLJ10781),	-1.11986907	0.36143428
NM_000717	carbonic anhydrase IV (CA4),	-1.11976457	1.08724988
NM_003063	sarcolipin (SLN),	-1.11922305	0.75970874
NM_000193	sonic hedgehog homolog (Drosophila) (SHH),	-1.11796411	1.01554357
NM_207112	hydroxyacylglutathione hydrolase-like (HAGHL), transcript variant 1,	-1.11759733	0.03653452
NM_002962	S100 calcium binding protein A5 (S100A5),	-1.11748311	0.44077113
XR_010411	G-protein signalling modulator 2 (AGS3-like, C. elegans) (LOC696458),	-1.11740558	0.11395492
NM_173469	hypothetical protein LOC92912 (LOC92912),	-1.11725632	0.18148419
CN646702	Hs.42834	-1.11692125	0.12472093
NM_015493	ankyrin repeat domain 25 (ANKRD25),	-1.11541388	0.04142209
NM_012198	grancalcin, EF-hand calcium binding protein (GCA),	-1.11529241	0.15108575
NM_182571	hypothetical protein 284297 (FLJ35258),	-1.11519839	0.5942615
NM_004722	adaptor-related protein complex 4, mu 1 subunit (AP4M1),	-1.11445865	0.16623715
NM_000136	Fanconi anemia, complementation group C (FANCC),	-1.11396451	0.89411082
NM_001512	glutathione S-transferase A4 (GSTA4),	-1.11335983	0.09427995
NM_025092	hypothetical protein FLJ22635 (FLJ22635),	-1.11234744	0.07861349
NM_002313	actin binding LIM protein 1 (ABLIM1), transcript variant ABLIM-l,	-1.11222504	0.05471499
NM_173505	ankyrin repeat domain 29 (ANKRD29),	-1.11144885	0.17850767

NM_000853	glutathione S-transferase theta 1 (GSTT1),	-1.11133568	0.41815615
NM_014451	parathyroid hormone-responsive B1 gene (B1), transcript variant 1,	-1.11111237	0.02801246
NM_014010	astrotactin 2 (ASTN2), transcript variant 1,	-1.11082593	0.13926655
NM_018204	cytoskeleton associated protein 2 (CKAP2),	-1.11041293	1.07434937
NM_152306	ubiquitin-like, containing PHD and RING finger domains, 2 (UHRF2), transcript variant 1,	-1.11036945	0.04226268
NM_001255	CDC20 cell division cycle 20 homolog (S. cerevisiae) (CDC20),	-1.11020761	0.10532056
NM_016044	fumarylacetoacetate hydrolase domain containing 2A (FAHD2A),	-1.11017336	0.03194076
NM_003098	syntrophin, alpha 1 (dystrophin-associated protein A1, 59kDa, acidic component) (SNTA1),	-1.10690806	0.26594131
NM_032496	Rho GTPase activating protein 9 (ARHGAP9),	-1.10612654	0.04705438
NM_001776	ectonucleoside triphosphate diphosphohydrolase 1 (ENTPD1),	-1.10611826	0.23126473
CN644408	COL4A1	-1.1058607	0.23632233
NM_024876	aarF domain containing kinase 4 (ADCK4),	-1.10583215	0.00093138
NM_014836	Rho-related BTB domain containing 1 (RHOBTB1), transcript variant 1,	-1.10571445	0.02034718
NM_004952	ephrin-A3 (EFNA3),	-1.10542509	0.37077381
NM_002402	mesoderm specific transcript homolog (mouse) (MEST), transcript variant 1,	-1.10524247	0.0100172
NM_003006	selectin P ligand (SELPLG),	-1.10456426	0.01264618
XR_014523	Protein C9orf116 (Pierce 1) (LOC720855),	-1.10377968	0.25400611
NM_000662	N-acetyltransferase 1 (arylamine N-acetyltransferase) (NAT1),	-1.10353861	0.05412287
NM_020415	resistin (RETN),	-1.10285387	0.0352271
NM_000044	androgen receptor (dihydrotestosterone receptor; testicular feminization; spinal and bulbar muscular atrophy; Kennedy disease) (AR),	-1.10250353	0.11454978
NM_024575	hypothetical protein FLJ23467 (FLJ23467),	-1.10215618	0.0375509
NM_022783	DEP domain containing 6 (DEPDC6),	-1.10051805	0.20780001
NM_014370	serine/threonine kinase 23 (STK23),	-1.09929552	0.14084378
NM_198098	aquaporin 1 (channel-forming integral protein, 28kDa) (AQP1), transcript variant 1,	-1.09919078	0.28040014
NM_005252	v-fos FBJ murine osteosarcoma viral oncogene homolog (FOS),	-1.09892055	0.0951528
NM_014726	ProSAPiP2 protein (ProSAPiP2),	-1.09800912	0.32665065
NM_024579	hypothetical protein FLJ23221 (FLJ23221),	-1.09795159	0.00567547
NM_014836	Rho-related BTB domain containing 1 (RHOBTB1), transcript variant 1,	-1.09771395	0.12652735
NM_018937	protocadherin beta 3 (PCDHB3),	-1.09737672	0.15556884
NM_007112	thrombospondin 3 (THBS3),	-1.0960059	0.25114833
NM_016356	doublecortin domain containing 2 (DCDC2),	-1.09482279	0.15648504
NM_013314	B-cell linker (BLNK),	-1.09388758	0.09601144
NM_178423	histone deacetylase 9 (HDAC9), transcript variant 4,	-1.09367979	0.60194587
NM_020802	KIAA1377 protein (KIAA1377),	-1.09357476	0.00243998
NM_014725	START domain containing 8 (STARD8),	-1.09334275	0.06204246
NM_004485	guanine nucleotide binding protein (G protein), gamma 4 (GNG4),	-1.09333345	0.5746548
NM_006340	BAI1-associated protein 2 (BAIAP2), transcript variant 3,	-1.09320124	0.05548282
NM_007029	stathmin-like 2 (STMN2),	-1.09183184	0.12898672
NM_022912	chromosome 2 open reading frame 23 (C2orf23),	-1.09029266	0.06685787
CO648296	MGC50844	-1.09016837	0.02311718
NM_175856	chondroitin sulfate synthase 3 (CSS3),	-1.09003642	0.49444244
NM_152676	F-box protein 15 (FBXO15),	-1.08937306	0.15070486
NM_000381	midline 1 (Opitz/BBB syndrome) (MID1), transcript variant 1,	-1.08823509	0.21081537
CN644332	ARHB	-1.08788939	0.0369462
DQ155428	LILRAb complete cds	-1.08731918	0.0152736

NM_052875	hypothetical protein MGC10485 (MGC10485),	-1.08697985	0.18112139
NM_021198	CTD (carboxy-terminal domain, RNA polymerase II, polypeptide A) small phosphatase 1 (CTDSP1),	-1.08660094	0.15564838
NM_017450	BAI1-associated protein 2 (BAIAP2), transcript variant 1,	-1.085866	0.10256451
NM_014721	phosphatase and actin regulator 2 (PHACTR2),	-1.08562096	0.12549263
NM_017668	nudE nuclear distribution gene E homolog 1 (A. nidulans) (NDE1),	-1.08485064	0.23816488
NM_001885	crystallin, alpha B (CRYAB),	-1.08444404	0.02175119
NM_024806	hypothetical protein FLJ23554 (FLJ23554), transcript variant 1,	-1.08396901	0.14370959
NM_006225	phospholipase C, delta 1 (PLCD1),	-1.08291583	0.07202033
NM_004185	wingless-type MMTV integration site family, member 2B (WNT2B), transcript variant WNT-2B1,	-1.0810755	0.14035723
NM_153274	vitelliform macular dystrophy 2-like 2 (VMD2L2),	-1.07991677	0.08482085
NM_002528	nth endonuclease III-like 1 (E. coli) (NTHL1),	-1.07914653	0.06994596
NM_002357	MAX dimerization protein 1 (MAD),	-1.07892034	1.05884246
NM_002960	S100 calcium binding protein A3 (S100A3),	-1.0789092	0.13463239
NM_000363	troponin I, cardiac (TNNI3),	-1.07869797	0.21109583
NM_152282	acid phosphatase-like 2 (ACPL2),	-1.07783178	0.13480523
NM_016564	BM88 antigen (BM88),	-1.07685567	0.31102395
NM_016373	WW domain containing oxidoreductase (WWOX), transcript variant 1,	-1.07663676	0.09020298
NM_014451	parathyroid hormone-responsive B1 gene (B1), transcript variant 1,	-1.07622779	0.0060973
CO646399	CTSD	-1.07605376	0.05393935
NM_032257	zinc finger, MYND domain containing 12 (ZMYND12),	-1.07314844	0.33667329
NM_017512	enolase superfamily member 1 (ENOSF1), transcript variant 2,	-1.0719607	0.07117315
NM_021570	BarH-like homeobox 1 (BARX1),	-1.071543	0.62251248
NM_000224	keratin 18 (KRT18), transcript variant 1,	-1.07070557	0.16876406
NM_007152	zinc finger protein 195 (ZNF195),	-1.07035861	0.03515826
NM_014661	KIAA0140 (KIAA0140),	-1.0701387	0.13188241
NM_052831	chromosome 6 open reading frame 192 (C6orf192),	-1.07008086	0.15896238
NM_015916	family with sequence similarity 26, member B (FAM26B),	-1.06970434	0.12838941
NM_201591	glycoprotein M6A (GPM6A), transcript variant 2,	-1.06854013	0.07688901
NM_015278	SAM and SH3 domain containing 1 (SASH1),	-1.06851731	0.14656182
NM_005211	colony stimulating factor 1 receptor, formerly McDonough feline sarcoma viral (v-fms) oncogene homolog (CSF1R),	-1.06791983	0.29339057
NM_004751	glucosaminyl (N-acetyl) transferase 3, mucin type (GCNT3),	-1.0678298	0.58384857
NM_052831	chromosome 6 open reading frame 192 (C6orf192),	-1.06703003	0.05684743
NM_001715	B lymphoid tyrosine kinase (BLK),	-1.06651024	0.14180638
NM_177454	KIAA1946 (KIAA1946),	-1.06576624	0.67950053
NM_033113	zinc finger protein 628 (ZNF628),	-1.06426611	0.04648662
CB554851	MMSP0045_G08 MMSP cDNA, sequence	-1.06423331	0.30999183
NM_001100	actin, alpha 1, skeletal muscle (ACTA1),	-1.06400805	0.14688935
XM_168060	chromosome 6 open reading frame 154 (C6orf154),	-1.06326045	0.02214585
NM_001584	chromosome 11 open reading frame 8 (C11orf8),	-1.06311218	0.05425116
NM_144978	hypothetical protein FLJ32745 (FLJ32745),	-1.06305378	0.07448081
XR_014618	Mps one binder kinase activator-like 2A (Mob1 homolog 2A) (LOC721441),	-1.06144873	0.80551826
NM_182487	olfactomedin-like 2A (OLFML2A),	-1.06081918	0.04754022
NM_015278	SAM and SH3 domain containing 1 (SASH1),	-1.06069932	0.3623788
NM_032714	chromosome 14 open reading frame 151 (C14orf151),	-1.06036214	0.69805951
NM_001146	angiopoietin 1 (ANGPT1), transcript variant 1,	-1.06036105	0.01432302
NM_003873	neuropilin 1 (NRP1),	-1.05890473	0.02738124

NM_001666	Rho GTPase activating protein 4 (ARHGAP4),	-1.05848885	0.24411115
NM_003062	slit homolog 3 (Drosophila) (SLIT3),	-1.05822755	0.05354519
NM_018460	Rho GTPase activating protein 15 (ARHGAP15),	-1.05711893	0.0528473
NM_000572	interleukin 10 (IL10),	-1.05628593	0.95144597
NM_025179	plexin A2 (PLXNA2),	-1.0554794	0.14081976
NM_000459	TEK tyrosine kinase, endothelial (venous malformations, multiple cutaneous and mucosal) (TEK),	-1.05496385	0.70495998
NM_030899	zinc finger protein 323 (ZNF323),	-1.0544999	0.41066857
NM_130847	angiomotin like 1 (AMOTL1),	-1.05435692	1.02186406
NM_138330	zinc finger protein 675 (ZNF675),	-1.05424832	0.82221253
XM_291277	hypothetical protein DKFZp761P0423 (DKFZp761P0423),	-1.05360705	0.2389024
NM_032301	F-box and WD-40 domain protein 9 (FBXW9),	-1.05214331	0.0534597
NM_001856	collagen, type XVI, alpha 1 (COL16A1),	-1.05153009	0.16670823
NM_000426	laminin, alpha 2 (merosin, congenital muscular dystrophy) (LAMA2),	-1.05129208	0.02705097
NM_018376	nipsnap homolog 3B (C. elegans) (NIPSNAP3B),	-1.05053946	0.02413793
NM_020458	tetratricopeptide repeat domain 7A (TTC7A),	-1.05026849	0.17160998
NM_021030	zinc finger protein 14 (KOX 6) (ZNF14),	-1.04989276	0.07531458
NM_00100725<br>5	kelch/ankyrin repeat containing cyclin A1 interacting protein (KARCA1), transcript variant 2,	-1.04916252	0.01999314
XR_010491	centaurin, gamma-like family, member 1 (LOC698675),	-1.04878546	0.7116396
NM_004364	CCAAT/enhancer binding protein (C/EBP), alpha (CEBPA),	-1.04848758	0.56423538
NM_022460	HS1-binding protein 3 (HS1BP3),	-1.04791668	0.17141051
NM_031426	chromosome 9 open reading frame 58 (C9orf58),	-1.04643299	0.19467856
NM_005185	calmodulin-like 3 (CALML3),	-1.04619959	0.69146796
NM_013351	T-box 21 (TBX21),	-1.04609331	0.03872297
NM_178545	transmembrane protein 52 (TMEM52),	-1.04539597	0.07480055
NM_005879	TRAF interacting protein (TRIP),	-1.04521269	0.01333938
NM_015493	ankyrin repeat domain 25 (ANKRD25),	-1.04427846	0.06931371
NM_000673	alcohol dehydrogenase 7 (class IV), mu or sigma polypeptide (ADH7),	-1.04405794	0.00557332
XR_013101	EVIN1 (LOC714977),	-1.04394075	0.03399387
NM_177533	nudix (nucleoside diphosphate linked moiety X)-type motif 14 (NUDT14),	-1.04385397	0.05581458
NM_016240	scavenger receptor class A, member 3 (SCARA3), transcript variant 1,	-1.04372691	0.23129226
NM_031469	SH3 domain binding glutamic acid-rich protein like 2 (SH3BGRL2),	-1.04349739	0.44554187
NM_020998	macrophage stimulating 1 (hepatocyte growth factor-like) (MST1),	-1.04330237	0.39988259
NM_015927	transforming growth factor beta 1 induced transcript 1 (TGFB1I1),	-1.04310212	0.32919902
NM_182973	transmembrane serine protease 9 (TMPRSS9),	-1.04307225	0.09485113
NM_145234	chordin-like 1 (CHRDL1),	-1.0426118	0.17814196
NM_004385	chondroitin sulfate proteoglycan 2 (versican) (CSPG2),	-1.04220601	0.16591663
NM_182513	spindle pole body component 24 homolog (S. cerevisiae) (SPBC24),	-1.04193468	0.82254846
NM_000667	alcohol dehydrogenase 1A (class I), alpha polypeptide (ADH1A),	-1.04125439	0.14044039
XM_496394	KIAA1693 protein (LOC440673),	-1.03959036	0.10882017
NM_012276	leukocyte immunoglobulin-like receptor, subfamily A (without TM domain), member 4 (ILT7),	-1.03932579	0.04792296
NM_181471	replication factor C (activator 1) 2, 40kDa (RFC2), transcript variant 1,	-1.03904691	0.90393779
NM_001535	HMT1 hnRNP methyltransferase-like 1 (S. cerevisiae) (HRMT1L1), transcript variant 2,	-1.03600464	0.03882361
NM_198232	ribonuclease, RNase A family, 1 (pancreatic) (RNASE1), transcript variant 3,	-1.03474831	0.37706998
NM_013258	PYD and CARD domain containing (PYCARD), transcript variant 1,	-1.03471238	0.05291135
NM_004538	nucleosome assembly protein 1-like 3 (NAP1L3),	-1.03411352	0.11864411

XR_014749	calcium channel, voltage-dependent, alpha 1H subunit (CACNA1H),	-1.03390954	0.17733978
NM_153026	prickle-like 1 (Drosophila) (PRICKLE1),	-1.03368235	0.05553012
NM_020650	reticulocalbin 3, EF-hand calcium binding domain (RCN3),	-1.03162491	0.28102205
CN803406	IGFBP6	-1.03156855	0.09379924
NM_024726	hypothetical protein FLJ22527 (FLJ22527),	-1.03148344	0.14705538
NM_001132	AFG3 ATPase family gene 3-like 1 (yeast) (AFG3L1),	-1.0296818	0.21717449
NM_173507	chromosome 1 open reading frame 127 (C1orf127),	-1.02895506	0.16898297
NM_013227	aggrecan 1 (chondroitin sulfate proteoglycan 1, large aggregating proteoglycan, antigen identified by monoclonal antibody A0122) (AGC1), transcript variant 2,	-1.02869735	0.32500496
NM_022003	FXYD domain containing ion transport regulator 6 (FXYD6),	-1.02845496	0.09050673
NM_032038	spinster (SPIN1),	-1.02845357	0.04780905
NM_000851	glutathione S-transferase M5 (GSTM5),	-1.0282784	0.21198036
NM_000260	myosin VIIA (Usher syndrome 1B (autosomal recessive, severe)) (MYO7A),	-1.02764642	0.15281039
NM_012109	chromosome 19 open reading frame 4 (C19orf4),	-1.02677127	0.15911622
NM_000044	androgen receptor (dihydrotestosterone receptor; testicular feminization; spinal and bulbar muscular atrophy; Kennedy disease) (AR),	-1.0267082	0.16125334
NM_013974	dimethylarginine dimethylaminohydrolase 2 (DDAH2),	-1.02646869	0.09805008
NM_003975	SH2 domain protein 2A (SH2D2A),	-1.02639523	0.513568
NM_000478	alkaline phosphatase, liver/bone/kidney (ALPL),	-1.02560124	0.06329409
NM_201574	solute carrier family 4, anion exchanger, member 3 (SLC4A3),	-1.02534388	0.23737117
NM_001627	activated leukocyte cell adhesion molecule (ALCAM),	-1.02517873	0.02166105
NM_033438	SLAM family member 9 (SLAMF9),	-1.0250511	0.03709833
NM_080629	collagen, type XI, alpha 1 (COL11A1), transcript variant B,	-1.02502588	0.08503542
NM_018470	chromosome 10 open reading frame 110 (C10orf110),	-1.02407485	0.95886846
NM_173474	N-terminal asparagine amidase (NTAN1),	-1.02364945	0.03594598
NM_030634	zinc finger protein 436 (ZNF436),	-1.02280682	0.11945849
CB230657	AGENCOURT_11469158 NICHD_Rh_Ov1 cDNA clone IMAGE:6883614 5', sequence	-1.02250504	0.85807318
NM_013351	T-box 21 (TBX21),	-1.02142722	0.0550711
NM_018719	transcription factor RAM2 (RAM2),	-1.0210433	0.21470687
NM_021177	LSM2 homolog, U6 small nuclear RNA associated (S. cerevisiae) (LSM2),	-1.01978903	0.38841263
NM_015916	family with sequence similarity 26, member B (FAM26B),	-1.0194498	0.07399943
XR_011087	dedicator of cytokinesis 8 (LOC698081),	-1.01916627	0.28329398
NM_006293	TYRO3 protein tyrosine kinase (TYRO3),	-1.01902905	0.08936163
NM_004693	cytokeratin type II (K6HF),	-1.01837535	0.34838793
NM_002402	mesoderm specific transcript homolog (mouse) (MEST), transcript variant 1,	-1.0178317	0.14896353
NM_175859	CTP synthase II (CTPS2), transcript variant 2,	-1.01769732	0.48351052
NM_001465	FYN binding protein (FYB-120/130) (FYB),	-1.0172892	0.26143146
NM_000673	alcohol dehydrogenase 7 (class IV), mu or sigma polypeptide (ADH7),	-1.0168315	0.07119257
NM_147184	tumor protein p53 inducible protein 3 (TP53I3), transcript variant 2,	-1.01576462	0.00149822
NM_144969	zinc finger, DHHC domain containing 15 (ZDHHC15),	-1.01546536	0.1000572
NM_052913	KIAA1913 (KIAA1913),	-1.01516969	0.69922355
NM_198232	ribonuclease, RNase A family, 1 (pancreatic) (RNASE1), transcript variant 3,	-1.0148864	0.03399421
NM_014766	secernin 1 (SCRN1),	-1.01469689	0.18646988
XR_013121	hypothetical protein LOC712369 (LOC712369),	-1.01448704	0.6917038
XR_013017	exosome component 7 (EXOSC7),	-1.01349031	0.2102559
NM_005572	lamin A/C (LMNA), transcript variant 2,	-1.0123542	0.1914338

NM_024806	hypothetical protein FLJ23554 (FLJ23554), transcript variant 1,	-1.01220199	0.09983143
NM_004512	interleukin 11 receptor, alpha (IL11RA), transcript variant 1,	-1.01214785	0.01152072
NM_000955	prostaglandin E receptor 1 (subtype EP1), 42kDa (PTGER1),	-1.0120731	0.74909979
NM_182973	transmembrane serine protease 9 (TMPRSS9),	-1.01197981	0.34308555
NM_014475	dihydrodiol dehydrogenase (dimeric) (DHDH),	-1.01176784	0.45293386
XM_496394	KIAA1693 protein (LOC440673),	-1.01160808	0.10098998
NM_152676	F-box protein 15 (FBXO15),	-1.01135509	0.30111679
XM_046861	KRAB box containing C2H2 type zinc finger bA526D8.4 (BA526D8.4),	-1.01095074	0.1962182
NM_006662	Snf2-related CBP activator protein (SRCAP),	-1.01066706	0.16057369
XR_010573	citron (LOC703199),	-1.01007511	0.15236014
XR_013997	Ubiquinone biosynthesis protein COQ4 homolog (Coenzyme Q biosynthesis protein 4 homolog) (COQ4),	-1.00903222	0.00742835
NM_138392	SH3KBP1 binding protein 1 (SHKBP1),	-1.00844221	0.01238673
NM_018676	thrombospondin, type I, domain containing 1 (THSD1), transcript variant 1,	-1.00837741	0.01630104
NM_004448	v-erb-b2 erythroblastic leukemia viral oncogene homolog 2, neuro/glioblastoma derived oncogene homolog (avian) (ERBB2),	-1.00722382	0.00516496
NM_001492	growth differentiation factor 1 (GDF1),	-1.00683784	0.16553869
NM_080549	protein tyrosine phosphatase, non-receptor type 6 (PTPN6), transcript variant 3,	-1.0063544	0.07350181
NM_198472	chromosome 10 open reading frame 125 (C10orf125),	-1.00609787	0.02284322
NM_005654	nuclear receptor subfamily 2, group F, member 1 (NR2F1),	-1.00592261	0.13113515
NM_144650	alcohol dehydrogenase, iron containing, 1 (ADHFE1),	-1.00528605	0.02817069
NM_001885	crystallin, alpha B (CRYAB),	-1.0052609	0.14538804
NM_025125	chromosome 10 open reading frame 57 (C10orf57),	-1.00524968	0.21343782
A_01_P003214	Unknown	-1.00448264	0.25989524
XR_010179	short-chain dehydrogenase	-1.00427929	0.05304789
NM_152284	Snf7 homologue associated with Alix 3 (Shax3),	-1.00357302	0.01024219
NM_012415	RAD54 homolog B (S. cerevisiae) (RAD54B), transcript variant 1,	-1.00332364	0.00074343
NM_013258	PYD and CARD domain containing (PYCARD), transcript variant 1,	-1.00322587	0.02766053
NM_207112	hydroxyacylglutathione hydrolase-like (HAGHL), transcript variant 1,	-1.00311608	0.10570453
NM_014227	solute carrier family 5 (low affinity glucose cotransporter), member 4 (SLC5A4),	-1.0030859	0.93285673
NM_019013	family with sequence similarity 64, member A (FAM64A),	-1.0027378	0.10986821
NM_175887	hypothetical protein LOC222171 (LOC222171),	-1.00210924	0.08039069
NM_017530	hypothetical protein LOC55565 (LOC55565),	-1.00171291	0.11941912
NM_031426	chromosome 9 open reading frame 58 (C9orf58),	-1.0011693	0.01577471
NM_173608	chromosome 14 open reading frame 80 (C14orf80),	-1.00065958	0.09346741
