# Supplementary material for: Microglia Are Mediators of Borrelia burgdorferi–Induced Apoptosis in SH-SY5Y Neuronal Cells
Source: PLoS Pathog. 2009 Nov 13;5(11):e1000659. doi: 10.1371/journal.ppat.1000659 (PMC2771360; doi:10.1371/journal.ppat.1000659)
Supplement: Table S2 — Microglia + Borrelia burgdorferi vs. Microglia alone, animal 2 (0.23 MB PDF) [file ppat.1000659.s002.pdf]

Table S2: Microglia + Borrelia burgdorferi vs. Microglia alone, animal 2

GeneName	Description	Average Log2 Normalized Fold-change	Standard Deviation
NM_000596	insulin-like growth factor binding protein 1 (IGFBP1)	4.66132921	3.75903242
NM_005623	chemokine (C-C motif) ligand 8 (CCL8)	4.60616971	1.51535433
NM_002090	chemokine (C-X-C motif) ligand 3 (CXCL3)	4.48200844	0.14741772
NM_002090	chemokine (C-X-C motif) ligand 3 (CXCL3)	4.11771953	0.12556731
NM_001511	chemokine (C-X-C motif) ligand 1 (melanoma growth stimulating activity, alpha) (CXCL1)	4.02661041	0.11458224
NM_001511	chemokine (C-X-C motif) ligand 1 (melanoma growth stimulating activity, alpha) (CXCL1)	3.97815565	0.102891
NM_176072	purinergic receptor P2Y, G-protein coupled, 2 (P2RY2), transcript variant 1	3.60409887	2.39169894
NM_002425	matrix metalloproteinase 10 (stromelysin 2) (MMP10)	3.44216891	1.83608732
NM_004433	E74-like factor 3 (ets domain transcription factor, epithelial-specific) (ELF3)	3.21679362	3.00869735
NM_002993	chemokine (C-X-C motif) ligand 6 (granulocyte chemotactic protein 2) (CXCL6)	3.18678771	0.09848732
NM_024698	solute carrier family 25 (mitochondrial carrier: glutamate), member 22 (SLC25A22)	3.1834986	1.24357491
NM_002993	chemokine (C-X-C motif) ligand 6 (granulocyte chemotactic protein 2) (CXCL6)	3.17001306	0.10301143
NM_000600	interleukin 6 (interferon, beta 2) (IL6)	3.09735094	0.20816456
NM_002982	chemokine (C-C motif) ligand 2 (CCL2)	3.05358394	0.12750943
NM_002982	chemokine (C-C motif) ligand 2 (CCL2)	3.05159701	0.00385607
NM_006536	chloride channel, calcium activated, family member 2 (CLCA2)	2.99968472	1.69692433
NM_000600	interleukin 6 (interferon, beta 2) (IL6)	2.99604904	0.05735944
NM_002422	matrix metalloproteinase 3 (stromelysin 1, progelatinase) (MMP3)	2.98115312	0.12416962
BX647205	mRNA; cDNA DKFZp686D2162 (from clone DKFZp686D2162) [BX647205]	2.95756716	0.11506758
NM_002164	indoleamine-pyrrole 2,3 dioxygenase (INDO)	2.91842949	0.20293249
A_01_P004595	Unknown	2.85475031	2.61837823
NM_015597	G-protein signalling modulator 1 (AGS3-like, C. elegans) (GPSM1)	2.82796833	0.97347669
NM_002164	indoleamine-pyrrole 2,3 dioxygenase (INDO)	2.75597546	0.25280668
NM_000636	superoxide dismutase 2, mitochondrial (SOD2), nuclear gene encoding mitochondrial protein, transcript variant 1	2.6924408	0.53730535

NM_004994	matrix metalloproteinase 9 (gelatinase B, 92kDa gelatinase, 92kDa type IV collagenase) (MMP9)	2.6643109	0.03925984
NM_145293	hypothetical protein FLJ20897 (LOC196549)	2.65673447	2.41798389
NM_002985	chemokine (C-C motif) ligand 5 (CCL5)	2.65556207	0.17960614
NM_000576	interleukin 1, beta (IL1B)	2.62060216	0.93805008
NM_001165	baculoviral IAP repeat-containing 3 (BIRC3), transcript variant 1	2.56519607	1.74934155
NM_004000	chitinase 3-like 2 (CHI3L2)	2.52997914	0.46416292
NM_002985	chemokine (C-C motif) ligand 5 (CCL5)	2.503988	0.07462564
NM_000576	interleukin 1, beta (IL1B)	2.49549913	0.24893314
NM_005408	chemokine (C-C motif) ligand 13 (CCL13)	2.48856829	0.15992355
NM_005408	chemokine (C-C motif) ligand 13 (CCL13)	2.47127771	0.18740649
CK232222	ILLUMIGEN_MCQ_3441 Katze_MMPL2 cDNA 5' human TFPI2 (Hs.438231)	2.46747197	0.15741311
CK232222	ILLUMIGEN_MCQ_3441 Katze_MMPL2 cDNA 5' human TFPI2 (Hs.438231)	2.45395554	0.15613704
NM_000594	tumor necrosis factor (TNF superfamily, member 2) (TNF)	2.44811644	0.44689509
NM_004994	matrix metalloproteinase 9 (gelatinase B, 92kDa gelatinase, 92kDa type IV collagenase) (MMP9)	2.44367406	0.1946218
NM_006290	tumor necrosis factor, alpha-induced protein 3 (TNFAIP3)	2.42982066	0.24522956
CK232222	ILLUMIGEN_MCQ_3441 Katze_MMPL2 cDNA 5' human TFPI2 (Hs.438231)	2.42126503	0.02570038
NM_003219	telomerase reverse transcriptase (TERT), transcript variant 1	2.39068451	2.15340358
NM_004000	chitinase 3-like 2 (CHI3L2)	2.33084566	0.61803748
NM_000594	tumor necrosis factor (TNF superfamily, member 2) (TNF)	2.32482169	0.03249704
NM_004613	transglutaminase 2 (C polypeptide, protein-glutamine-gamma-glutamyltransferase) (TGM2), transcript variant 1	2.31987888	0.00696209
NM_020215	chromosome 14 open reading frame 132 (C14orf132)	2.29736004	1.69143462
NM_012252	transcription factor EC (TFEC), transcript variant 1	2.24910879	1.43653463
NM_181842	zinc finger and BTB domain containing 12 (ZBTB12)	2.23636335	1.33801116
NM_017938	hypothetical protein FLJ20716 (FLJ20716)	2.175498	0.67080287
NM_000636	superoxide dismutase 2, mitochondrial (SOD2), nuclear gene encoding mitochondrial protein, transcript variant 1	2.16862649	0.21555307
NM_002185	interleukin 7 receptor (IL7R)	2.15296511	0.28327969
CO647386	ILLUMIGEN_MCQ_40418 Katze_MMPB2 cDNA clone IBIUW:21432 5' Bases 185 to 778 highly human CXCL2 (Hs.75765)	2.13794342	0.02594915

NM_003856	interleukin 1 receptor-like 1 (IL1RL1), transcript variant 2	2.13555527	0.75575715
NM_003842	tumor necrosis factor receptor superfamily, member 10b (TNFRSF10B), transcript variant 1	2.11644484	1.81048787
NM_003557	phosphatidylinositol-4-phosphate 5-kinase, type I, alpha (PIP5K1A)	2.09846068	0.11088818
NM_001838	chemokine (C-C motif) receptor 7 (CCR7)	2.09203707	1.98759676
NM_002620	platelet factor 4 variant 1 (PF4V1)	2.04228948	0.04558736
NM_007115	tumor necrosis factor, alpha-induced protein 6 (TNFAIP6)	2.02923055	1.47568178
NM_004613	transglutaminase 2 (C polypeptide, protein-glutamine-gamma-glutamyltransferase) (TGM2), transcript variant 1	2.02707184	0.0022676
NM_004054	complement component 3a receptor 1 (C3AR1)	2.00961103	0.91307445
NM_002421	matrix metalloproteinase 1 (interstitial collagenase) (MMP1)	2.00122687	0.26441937
NM_000022	adenosine deaminase (ADA)	1.99016661	1.29878838
NM_002620	platelet factor 4 variant 1 (PF4V1)	1.9848723	0.08962713
NM_173213	keratin 23 (histone deacetylase inducible) (KRT23), transcript variant 2	1.97749633	0.88643224
NM_004195	tumor necrosis factor receptor superfamily, member 18 (TNFRSF18), transcript variant 1	1.97236991	0.90237195
CO647386	ILLUMIGEN_MCQ_40418 Katze_MMPB2 cDNA clone IBIUW:21432 5' Bases 185 to 778 highly human CXCL2 (Hs.75765)	1.96953798	0.29271487
A_01_P009641	Unknown	1.96772043	1.30604392
NM_006290	tumor necrosis factor, alpha-induced protein 3 (TNFAIP3)	1.96734292	0.92785364
NM_007036	endothelial cell-specific molecule 1 (ESM1)	1.96651616	0.04613327
NM_024630	zinc finger, DHHC-type containing 14 (ZDHHC14)	1.9423846	0.69376698
XM_290848	hypothetical protein LOC339344 (LOC339344)	1.92448793	0.0066737
NM_002425	matrix metalloproteinase 10 (stromelysin 2) (MMP10)	1.90038186	0.50151744
NM_007036	endothelial cell-specific molecule 1 (ESM1)	1.88281576	0.41654568
NM_001006600	erbb2 interacting protein (ERBB2IP), transcript variant 7	1.86809865	1.1731053
A_01_P017592	Unknown	1.8626639	1.40799943
NM_005623	chemokine (C-C motif) ligand 8 (CCL8)	1.82698281	0.77308874
NM_018155	solute carrier family 25, member 36 (SLC25A36)	1.82425029	0.73296021
NM_001001437	chemokine (C-C motif) ligand 3-like, centromeric (MGC12815)	1.81657455	0.10251825
CN643589	ILLUMIGEN_MCQ_8103 Katze_MMPL2 cDNA clone IBIUW:3398 5' Bases 370 to 1045 highly human Unigene Hs.99785	1.80545008	1.47971862

NM_016724	folate receptor 1 (adult) (FOLR1), transcript variant 7	1.80198126	0.22970153
NM_022154	solute carrier family 39 (zinc transporter), member 8 (SLC39A8)	1.78604578	0.09305628
NM_004054	complement component 3a receptor 1 (C3AR1)	1.78537675	0.03887034
NM_022154	solute carrier family 39 (zinc transporter), member 8 (SLC39A8)	1.76541254	0.06047697
NM_002185	interleukin 7 receptor (IL7R)	1.75309069	0.81167165
NM_001354	aldo-keto reductase family 1, member C2 (dihydrodiol dehydrogenase 2; bile acid binding protein; 3-alpha hydroxysteroid dehydrogenase, type III) (AKR1C2), transcript variant 1	1.72509815	0.01012871
XM_056254	heparan sulfate (glucosamine) 3-O-sulfotransferase 4 (HS3ST4)	1.7235559	1.13811977
NM_001001437	chemokine (C-C motif) ligand 3-like, centromeric (MGC12815)	1.72031085	0.08108556
NM_003855	interleukin 18 receptor 1 (IL18R1)	1.72027225	0.72125153
NM_002421	matrix metalloproteinase 1 (interstitial collagenase) (MMP1)	1.71956934	0.21372396
NM_031419	molecule possessing ankyrin repeats induced by lipopolysaccharide (MAIL), homolog of mouse (MAIL)	1.71691276	0.05058248
NM_033064	ataxia, cerebellar, Cayman type (caytaxin) (ATCAY)	1.68895224	1.27107199
NM_001710	B-factor, properdin (BF)	1.68335532	0.070814
NM_032119	monogenic, audiogenic seizure susceptibility 1 homolog (mouse) (MASS1)	1.67420584	1.11943398
NM_002638	protease inhibitor 3, skin-derived (SKALP) (PI3)	1.67275055	0.0154993
NM_005725	tetraspanin 2 (TSPAN2)	1.65400176	1.21728813
XR_009756	hypothetical protein LOC693940 (LOC693940)	1.64832876	1.23497814
NM_001835	clathrin, heavy polypeptide-like 1 (CLTCL1), transcript variant 1	1.61796833	1.35844897
XR_013663	Putative lymphocyte G0 [XR_013663]	1.60710991	0.19614354
NM_031419	molecule possessing ankyrin repeats induced by lipopolysaccharide (MAIL), homolog of mouse (MAIL)	1.58059255	0.08893115
NM_172240	WD repeat domain 51B (WDR51B)	1.56198058	0.94383682
NM_178025	gamma-glutamyltransferase-like 3 (GGTL3), transcript variant 2	1.5531342	1.09468769
NM_006203	phosphodiesterase 4D, cAMP-specific (phosphodiesterase E3 dunce homolog, Drosophila) (PDE4D)	1.55205348	0.47020778
NM_006273	chemokine (C-C motif) ligand 7 (CCL7)	1.54951696	0.01983634
NM_001078	vascular cell adhesion molecule 1 (VCAM1), transcript variant 1	1.54764775	0.18798973

CN801994	ILLUMIGEN_MCQ_37433 Katze_MMBR cDNA clone IBIUW:16004 5' Bases 1 to 410 highly human MT1X (Hs.374950)	1.53988	0.04399357
NM_001078	vascular cell adhesion molecule 1 (VCAM1), transcript variant 1	1.52719851	0.06520901
NM_001734	complement component 1, s subcomponent (C1S), transcript variant 1	1.50463255	0.30457495
NM_012337	coiled-coil domain containing 19 (CCDC19)	1.50331435	1.12424728
NM_174941	scavenger receptor cysteine-rich type 1 protein M160 (M160)	1.49258025	0.13034373
NM_018370	hypothetical protein FLJ11259 (FLJ11259)	1.48163789	0.14527386
NM_001353	aldo-keto reductase family 1, member C1 (dihydrodiol dehydrogenase 1; 20-alpha (3-alpha)-hydroxysteroid dehydrogenase) (AKR1C1)	1.47929214	0.24175588
NM_020125	SLAM family member 8 (SLAMF8)	1.47697891	0.07718282
AY635466	cytochrome P450 CYP3A66 (CYP3A66) mRNA, complete cds [AY635466]	1.47448438	0.36982056
NM_001267	chondroadherin (CHAD)	1.46829235	0.24193422
CK230409	ILLUMIGEN_MCQ_731 Katze_MMPL2 cDNA 5' human GLRX (Hs.28988)	1.4657802	0.02019016
NM_021641	a disintegrin and metalloproteinase domain 12 (meltrin alpha) (ADAM12), transcript variant 2	1.45863996	0.71569185
NM_006795	EH-domain containing 1 (EHD1)	1.44723998	1.03185334
A_01_P002921	Unknown	1.44486428	0.87394803
NM_018370	hypothetical protein FLJ11259 (FLJ11259)	1.43494954	0.02895763
NM_002600	phosphodiesterase 4B, cAMP-specific (phosphodiesterase E4 dunce homolog, Drosophila) (PDE4B)	1.43228012	0.85548801
NM_177551	G protein-coupled receptor 109A (GPR109A)	1.43165199	0.19379247
NM_002638	protease inhibitor 3, skin-derived (SKALP) (PI3)	1.43119013	0.32318589
NM_000577	interleukin 1 receptor antagonist (IL1RN), transcript variant 3	1.4304837	0.00993908
NM_003004	secreted and transmembrane 1 (SECTM1)	1.42799381	0.0422735
NM_002350	v-yes-1 Yamaguchi sarcoma viral related oncogene homolog (LYN)	1.42409906	0.32205338
CN801994	ILLUMIGEN_MCQ_37433 Katze_MMBR cDNA clone IBIUW:16004 5' Bases 1 to 410 highly human MT1X (Hs.374950)	1.41992754	0.22262405
NM_173828	chromosome 5 open reading frame 16 (C5orf16)	1.41576239	0.30412125
NM_005565	lymphocyte cytosolic protein 2 (SH2 domain containing leukocyte protein of 76kDa) (LCP2)	1.4157007	0.38857216
NM_001835	clathrin, heavy polypeptide-like 1 (CLTCL1), transcript variant 1	1.41031488	0.45811215
NM_001007072	zinc finger and SCAN domain containing 2 (ZSCAN2), transcript variant 3	1.40704694	1.0685369

NM_006273	chemokine (C-C motif) ligand 7 (CCL7)	1.38699806	0.18915312
NM_018425	phosphatidylinositol 4-kinase type II (PI4KII)	1.37564798	0.65125578
A_01_P000634	Unknown	1.37203457	0.8942453
NM_000022	adenosine deaminase (ADA)	1.3647191	0.08472036
NM_001353	aldo-keto reductase family 1, member C1 (dihydrodiol dehydrogenase 1; 20-alpha (3-alpha)-hydroxysteroid dehydrogenase) (AKR1C1)	1.36416682	0.28801531
NM_032381	Unknown	1.35447725	0.28108743
NM_033449	FCH and double SH3 domains 1 (FCHSD1)	1.35234192	0.48383188
NM_000064	complement component 3 (C3)	1.35085693	0.00867155
NM_001007531	chromosome 6 open reading frame 194 (C6orf194)	1.34797237	0.11257226
NM_006573	tumor necrosis factor (ligand) superfamily, member 13b (TNFSF13B)	1.34410893	0.03035545
XR_014707	Complement C1r subcomponent precursor (Complement component 1, r subcomponent) (LOC722131)	1.33171567	0.04136522
NM_000577	interleukin 1 receptor antagonist (IL1RN), transcript variant 3	1.32650724	0.02948292
XR_014707	Complement C1r subcomponent precursor (Complement component 1, r subcomponent) (LOC722131)	1.32608263	0.1293156
NM_198797	prostaglandin E synthase (PTGES), transcript variant 2	1.32247743	0.15094461
NM_001710	B-factor, properdin (BF)	1.31138493	0.61785651
NM_024689	chromosome X open reading frame 36 (CXorf36)	1.31112288	0.64045882
NM_002426	matrix metalloproteinase 12 (macrophage elastase) (MMP12)	1.31110028	0.76663654
NM_001570	interleukin-1 receptor-associated kinase 2 (IRAK2)	1.30461394	0.15034433
XR_012592	tau tubulin kinase 2 (LOC712249)	1.3037609	0.01957346
A_01_P007370	Unknown	1.29215689	0.73871323
XR_013663	Putative lymphocyte G0 [XR_013663]	1.27929623	0.47119611
NM_000689	aldehyde dehydrogenase 1 family, member A1 (ALDH1A1)	1.27812905	0.83477588
NM_153223	hypothetical protein FLJ36090 (FLJ36090)	1.27690285	0.76604223
NM_016184	C-type (calcium dependent, carbohydrate-recognition domain) lectin, superfamily member 6 (CLECSF6), transcript variant 1	1.27655016	0.13676513
NM_002243	potassium inwardly-rectifying channel, subfamily J, member 15 (KCNJ15), transcript variant 2	1.2683162	0.24000273
NM_000397	cytochrome b-245, beta polypeptide (chronic granulomatous disease) (CYBB)	1.26336342	0.0155334
NM_000689	aldehyde dehydrogenase 1 family, member A1 (ALDH1A1)	1.25479305	0.11605541
NM_000575	interleukin 1, alpha (IL1A)	1.25385087	0.4118695
NM_194294	hypothetical protein LOC169355 (LOC169355)	1.25257107	0.00148404

NM_033549	tripartite motif-containing 41 (TRIM41), transcript variant 1	1.25147804	0.49643936
NM_002068	guanine nucleotide binding protein (G protein), alpha 15 (Gq class) (GNA15)	1.24764635	0.15802012
NM_198797	prostaglandin E synthase (PTGES), transcript variant 2	1.24213192	0.18733988
NM_014808	FERM, RhoGEF and pleckstrin domain protein 2 (FARP2)	1.24141393	0.5162353
NM_020370	G protein-coupled receptor 84 (GPR84)	1.24079035	0.10009495
NM_020125	SLAM family member 8 (SLAMF8)	1.23816495	0.61595098
NM_080757	chromosome 20 open reading frame 127 (C20orf127)	1.2360878	0.06418335
NM_175617	metallothionein 1E (functional) (MT1E)	1.23556959	0.05427791
DR774422	ILLUMIGEN_MCQ_58593 Katze_MMLV cDNA clone IBIUW:34887 5' Bases 9 to 306 highly human Unigene Hs.529672	1.23418556	0.09879622
CO645773	ILLUMIGEN_MCQ_30118 Katze_MMPB cDNA clone IBIUW:22572 5' Bases 1 to 42 highly human RARRES3 (Hs.17466)	1.23344355	0.46364181
NM_002030	formyl peptide receptor-like 2 (FPRL2)	1.22604487	0.00986366
NM_004726	RALBP1 associated Eps domain containing 2 (REPS2)	1.21582776	0.46345127
NM_003608	G protein-coupled receptor 65 (GPR65)	1.21380922	0.06803306
NM_023003	transmembrane 6 superfamily member 1 (TM6SF1)	1.21125314	0.28048792
NM_021800	DnaJ (Hsp40) homolog, subfamily C, member 12 (DNAJC12), transcript variant 1	1.21059622	0.57433262
NM_005211	colony stimulating factor 1 receptor, formerly McDonough feline sarcoma viral (v-fms) oncogene homolog (CSF1R)	1.20986913	0.05166158
XM_056254	heparan sulfate (glucosamine) 3-O-sulfotransferase 4 (HS3ST4)	1.20867983	0.15171197
NM_177551	G protein-coupled receptor 109A (GPR109A)	1.2059952	0.18478358
NM_005949	metallothionein 1F (functional) (MT1F)	1.20539389	0.12967259
NM_001010986	ATPase, Class VI, type 11C (ATP11C), transcript variant 2	1.2006151	0.12742559
NM_032098	protocadherin gamma subfamily B, 4 (PCDHGB4), transcript variant 2	1.19789384	0.10683329
NM_145259	activin A receptor, type IC (ACVR1C)	1.19618663	0.75603426
XM_375224	cervical cancer suppressor-1 (LOC400410)	1.18923296	0.62539647
NM_016184	C-type (calcium dependent, carbohydrate-recognition domain) lectin, superfamily member 6 (CLECSF6), transcript variant 1	1.18218258	0.17697981
NM_175617	metallothionein 1E (functional) (MT1E)	1.17976881	0.06760833
NM_001734	complement component 1, s subcomponent (C1S), transcript variant 1	1.17807829	0.23337792

NM_145725	TNF receptor-associated factor 3 (TRAF3), transcript variant 1	1.17739934	0.43902888
NM_018309	hypothetical protein FLJ11046 (FLJ11046)	1.17735224	0.02090298
NM_000064	complement component 3 (C3)	1.17717208	0.07172058
NM_177551	G protein-coupled receptor 109A (GPR109A)	1.17446359	0.10090004
NM_001159	aldehyde oxidase 1 (AOX1)	1.1716749	0.19310534
CO644910	ILLUMIGEN_MCQ_43164 Katze_MMJJ cDNA clone IBIUW:23286 5' Bases 7 to 752 highly human NMES1 (Hs.112242)	1.17030404	0.23966745
NM_172374	interleukin 4 induced 1 (IL4I1), transcript variant 2	1.16944195	0.05025056
CK230409	ILLUMIGEN_MCQ_731 Katze_MMPL2 cDNA 5' human GLRX (Hs.28988)	1.16588333	0.58559434
NM_025245	pre-B-cell leukemia transcription factor 4 (PBX4)	1.16480393	0.62949183
NM_024746	hypothetical protein FLJ13840 (FLJ13840)	1.16187399	0.62576544
NM_152327	adenylate kinase 7 (AK7)	1.15762494	0.36587529
NM_002350	v-yes-1 Yamaguchi sarcoma viral related oncogene homolog (LYN)	1.15150538	0.12530758
NM_003956	cholesterol 25-hydroxylase (CH25H)	1.14987285	0.14085076
NM_006509	v-rel reticuloendotheliosis viral oncogene homolog B, nuclear factor of kappa light polypeptide gene enhancer in B-cells 3 (avian) (RELB)	1.14546961	0.00654834
NM_152309	phosphoinositide-3-kinase adaptor protein 1 (PIK3AP1)	1.14148265	0.20510416
NM_006946	spectrin, beta, non-erythrocytic 2 (SPTBN2)	1.1411499	0.37909844
NM_004318	aspartate beta-hydroxylase (ASPH), transcript variant 1	1.14108443	0.46597871
NM_004289	nuclear factor (erythroid-derived 2)-like 3 (NFE2L3)	1.14021139	0.37113869
NM_005949	metallothionein 1F (functional) (MT1F)	1.13837621	0.06818933
NM_002984	chemokine (C-C motif) ligand 4 (CCL4)	1.13817322	0.06712686
NM_174941	scavenger receptor cysteine-rich type 1 protein M160 (M160)	1.13386829	0.29036856
NM_015687	filamin A interacting protein 1 (FILIP1)	1.12767966	0.42268168
NM_018898	protocadherin alpha subfamily C, 1 (PCDHAC1), transcript variant 1	1.12474301	0.13800232
NM_153347	hypothetical protein FLJ90119 (FLJ90119)	1.12320591	0.6124046
NM_033102	prostate cancer associated protein 6 (PCANAP6)	1.12089027	0.2700534
NM_001801	cysteine dioxygenase, type I (CDO1)	1.11901905	0.23612113
NM_002040	GA binding protein transcription factor, alpha subunit 60kDa (GABPA)	1.11820422	0.23553928
CN645851	ILLUMIGEN_MCQ_24996 Katze_MMBR cDNA clone IBIUW:10667 5' Bases 1 to 902 highly human Unigene Hs.411391	1.11606633	0.42063985
NM_002053	guanylate binding protein 1, interferon-inducible, 67kDa (GBP1)	1.11207566	0.20003281

NM_173514	hypothetical protein FLJ90709 (FLJ90709)	1.10571023	0.34333313
NM_017745	BCL6 co-repressor (BCOR), transcript variant 1	1.10191192	0.27187543
NM_031415	melanoma-derived leucine zipper, extra-nuclear factor (MLZE)	1.10154704	0.1292536
NM_178817	melanocortin 2 receptor accessory protein (MRAP), transcript variant 1	1.0980262	0.00528652
NM_020370	G protein-coupled receptor 84 (GPR84)	1.09735576	0.05751044
A_01_P009752	Unknown	1.09509231	0.44552921
NM_001165	baculoviral IAP repeat-containing 3 (BIRC3), transcript variant 1	1.09040297	0.10767257
NM_133467	Cbp/p300-interacting transactivator, with Glu/Asp-rich carboxy-terminal domain, 4 (CITED4)	1.08937944	0.02040022
NM_018898	protocadherin alpha subfamily C, 1 (PCDHAC1), transcript variant 1	1.08688711	0.29812119
NM_005565	lymphocyte cytosolic protein 2 (SH2 domain containing leukocyte protein of 76kDa) (LCP2)	1.08380707	0.05175288
NM_032935	metallothionein IV (MT4)	1.08344627	0.00315627
NM_032604	abhydrolase domain containing 1 (ABHD1), transcript variant 1	1.08145589	0.16735126
CN644516	ILLUMIGEN_MCQ_10491 Katze_MMPL2 cDNA clone IBIUW:9332 5' Bases 1 to 959 highly human ARL7 (Hs.111554)	1.0804497	0.02505962
NM_152309	phosphoinositide-3-kinase adaptor protein 1 (PIK3AP1)	1.07899665	0.09016314
NM_015196	KIAA0922 protein (KIAA0922)	1.0753973	0.48399518
NM_000596	insulin-like growth factor binding protein 1 (IGFBP1)	1.06691657	0.25890273
NM_002669	pleiotropic regulator 1 (PRL1homolog, Arabidopsis) (PLRG1)	1.06602669	0.08972343
NM_005988	small proline-rich protein 2A (SPRR2A)	1.06412809	0.55489302
XR_010654	acyl-CoA synthetase long-chain family member 5 isoform a (LOC696404)	1.06399398	0.21082542
CO644910	ILLUMIGEN_MCQ_43164 Katze_MMJJ cDNA clone IBIUW:23286 5' Bases 7 to 752 highly human NMES1 (Hs.112242)	1.05983826	0.06718617
NM_000873	intercellular adhesion molecule 2 (ICAM2)	1.05665256	0.24226537
NM_001007189	bovine IgA regulatory protein (LOC492311)	1.05455264	0.3218679
NM_003004	secreted and transmembrane 1 (SECTM1)	1.0544153	0.04891211
NM_022367	sema domain, immunoglobulin domain (Ig), transmembrane domain (TM) and short cytoplasmic domain, (semaphorin) 4A (SEMA4A)	1.05328092	0.08591012
XR_014518	mitogen-activated protein kinase 13 (LOC719085)	1.05075955	0.42146831
NM_022136	SAM domain, SH3 domain and nuclear localisation signals, 1 (SAMSN1)	1.04754127	0.09009337
NM_020992	PDZ and LIM domain 1 (elfin) (PDLIM1)	1.04671727	0.09878113

NM_001002294	flavin containing monooxygenase 3 (FMO3), transcript variant 2	1.04547513	0.45599537
NM_006403	neural precursor cell expressed, developmentally down-regulated 9 (NEDD9)	1.04266762	0.02595243
NM_022136	SAM domain, SH3 domain and nuclear localisation signals, 1 (SAMSN1)	1.04041588	0.34205805
NM_003953	myelin protein zero-like 1 (MPZL1), transcript variant 1	1.03904299	0.37503009
NM_015424	chordin-like 2 (CHRDL2)	1.03875047	0.17725575
NM_000104	cytochrome P450, family 1, subfamily B, polypeptide 1 (CYP1B1)	1.03507992	0.03058814
CN647646	ILLUMIGEN_MCQ_28879 Katze_MMPB cDNA clone IBIUW:7398 5' Bases 1 to 917 highly human ARHGEF11 (Hs.371602)	1.03326653	0.35317624
NM_000094	collagen, type VII, alpha 1 (epidermolysis bullosa, dystrophic, dominant and recessive) (COL7A1)	1.03070573	0.19173469
NM_000591	CD14 antigen (CD14)	1.0297969	0.06451845
NM_000094	collagen, type VII, alpha 1 (epidermolysis bullosa, dystrophic, dominant and recessive) (COL7A1)	1.02926238	0.12875512
NM_001906	chymotrypsinogen B1 (CTRB1)	1.02862047	0.28728667
NM_000376	vitamin D (1,25- dihydroxyvitamin D3) receptor (VDR), transcript variant 1	1.02780771	0.38939418
NM_025225	adiponutrin (ADPN)	1.0270693	0.15595194
NM_144586	LY6/PLAUR domain containing 1 (LYPDC1)	1.02373897	0.0379763
NM_020240	CDC42 small effector 2 (CDC42SE2)	1.02297156	0.107695
NM_002030	formyl peptide receptor-like 2 (FPRL2)	1.02057826	0.13591301
NM_033086	FYVE, RhoGEF and PH domain containing 3 (FGD3)	1.01261811	0.49248976
NM_018593	solute carrier family 16 (monocarboxylic acid transporters), member 10 (SLC16A10)	1.01155694	0.17366101
NM_199168	chemokine (C-X-C motif) ligand 12 (stromal cell-derived factor 1) (CXCL12)	1.00977437	0.06856185
NM_018487	hepatocellular carcinoma-associated antigen 112 (HCA112)	1.00966339	0.386361
NM_032935	metallothionein IV (MT4)	1.00922306	0.16186096
DQ148132	clone ss1_j21_t7_328 aryl hydrocarbon receptor nuclear translocator 2 (ARNT2) mRNA, 3' UTR [DQ148132]	1.00846829	0.41795559
NM_001010986	ATPase, Class VI, type 11C (ATP11C), transcript variant 2	1.00739303	0.05488051
NM_020529	nuclear factor of kappa light polypeptide gene enhancer in B-cells inhibitor, alpha (NFKBIA)	1.00290611	0.15861519
NM_002193	inhibin, beta B (activin AB beta polypeptide) (INHBB)	1.00248357	0.27842584
NM_004750	cytokine receptor-like factor 1 (CRLF1)	1.00022739	0.00981598
NM_021972	sphingosine kinase 1 (SPHK1)	0.9989461	0.34159597

NM_014622	loss of heterozygosity, 11, chromosomal region 2, gene A (LOH11CR2A), transcript variant 1	0.99115071	0.01720048
NM_001295	chemokine (C-C motif) receptor 1 (CCR1)	0.99109033	0.17779983
NM_021965	phosphoglucomutase 5 (PGM5)	0.98672129	0.32025812
NM_019609	carboxypeptidase X (M14 family) (CPXM)	0.98631887	0.0253944
NM_016293	bridging integrator 2 (BIN2)	0.98625546	0.02485989
NM_022367	sema domain, immunoglobulin domain (Ig), transmembrane domain (TM) and short cytoplasmic domain, (semaphorin) 4A (SEMA4A)	0.98388708	0.21978256
NM_001906	chymotrypsinogen B1 (CTRB1)	0.98315373	0.05662752
NM_015110	SMC5 structural maintenance of chromosomes 5-like 1 (yeast) (SMC5L1)	0.9827499	0.35716303
NM_020992	PDZ and LIM domain 1 (elfin) (PDLIM1)	0.98129868	0.04861582
A_01_P010787	Unknown	0.9803921	0.38172097
NM_080283	ATP-binding cassette, sub-family A (ABC1), member 9 (ABCA9), transcript variant 1	0.97963657	0.14342425
NM_000397	cytochrome b-245, beta polypeptide (chronic granulomatous disease) (CYBB)	0.97366507	0.19223928
NM_000609	chemokine (C-X-C motif) ligand 12 (stromal cell-derived factor 1) (CXCL12)	0.97345559	0.07967997
NM_002984	chemokine (C-C motif) ligand 4 (CCL4)	0.97220866	0.0690664
NM_018487	hepatocellular carcinoma-associated antigen 112 (HCA112)	0.97108207	0.40537935
NM_138455	collagen triple helix repeat containing 1 (CTHRC1)	0.96823531	0.01953204
NM_178817	melanocortin 2 receptor accessory protein (MRAP), transcript variant 1	0.96384314	0.17739085
NM_177551	G protein-coupled receptor 109A (GPR109A)	0.96051731	0.25350823
NM_080283	ATP-binding cassette, sub-family A (ABC1), member 9 (ABCA9), transcript variant 1	0.95906047	0.23731617
NM_014622	loss of heterozygosity, 11, chromosomal region 2, gene A (LOH11CR2A), transcript variant 1	0.95787013	0.05229576
NM_003706	phospholipase A2, group IVC (cytosolic, calcium-independent) (PLA2G4C)	0.95600136	0.32990784
NM_002426	matrix metalloproteinase 12 (macrophage elastase) (MMP12)	0.95233298	0.03532773
NM_001677	ATPase, Na+/K+ transporting, beta 1 polypeptide (ATP1B1)	0.94864422	0.19531948
NM_005204	mitogen-activated protein kinase kinase kinase 8 (MAP3K8)	0.94589876	0.03168683
NM_001009584	armadillo repeat containing, X-linked 6 (ARMCX6), transcript variant 2	0.94539923	0.40290883
NM_000240	monoamine oxidase A (MAOA), nuclear gene encoding mitochondrial protein	0.94372782	0.07413394
XR_010654	acyl-CoA synthetase long-chain family member 5 isoform a (LOC696404)	0.93788098	0.09298242

NM_000201	intercellular adhesion molecule 1 (CD54), human rhinovirus receptor (ICAM1)	0.93155436	0.00715484
DQ266251	Macaca fascicularis 17-beta hydroxysteroid dehydrogenase 5 (HSD17B5) mRNA, complete cds [DQ266251]	0.92921564	0.07126365
A_01_P000414	Unknown	0.92761579	0.20820457
NM_005204	mitogen-activated protein kinase kinase kinase 8 (MAP3K8)	0.9268988	0.14507218
NM_012324	mitogen-activated protein kinase 8 interacting protein 2 (MAPK8IP2), transcript variant 1	0.92544695	0.28615533
NM_080757	chromosome 20 open reading frame 127 (C20orf127)	0.92507396	0.05793961
NM_000693	aldehyde dehydrogenase 1 family, member A3 (ALDH1A3)	0.92216845	0.23059159
NM_017515	solute carrier family 35, member F2 (SLC35F2)	0.92025634	0.10943643
XM_290546	KIAA0830 protein (KIAA0830)	0.91971209	0.33747686
NM_003937	kynureninase (L-kynurenine hydrolase) (KYNU)	0.91905644	0.1737923
NM_178443	UNC-112 related protein 2 (URP2), transcript variant URP2LF	0.91861202	0.10817814
NM_013368	SERTA domain containing 3 (SERTAD3), transcript variant 1	0.91764236	0.17170133
NM_000698	arachidonate 5-lipoxygenase (ALOX5)	0.91738576	0.11423725
NM_015310	pleckstrin and Sec7 domain containing 3 (PSD3), transcript variant 1	0.9166105	0.29458317
NM_033102	prostate cancer associated protein 6 (PCANAP6)	0.91451573	0.12273328
NM_178443	UNC-112 related protein 2 (URP2), transcript variant URP2LF	0.91443536	0.0453395
NM_015687	filamin A interacting protein 1 (FILIP1)	0.91402783	0.19204048
NM_175895	hypothetical protein FLJ25590 (FLJ25590)	0.91363391	0.19664174
NM_004079	cathepsin S (CTSS)	0.91355414	0.15373203
NM_001637	acyloxyacyl hydrolase (neutrophil) (AOAH)	0.91320832	0.03147705
NM_178148	solute carrier family 35, member B2 (SLC35B2)	0.91270174	0.30862974
NM_138397	Unknown	0.91132659	0.16745688
DR771828	ILLUMIGEN_MCQ_10784 Katze_MMPL2 cDNA clone IBIUW:37887 5' Bases 199 to 549 highly human SLC7A2 (Hs.448520)	0.90884449	0.02766927
NM_148170	cathepsin C (CTSC), transcript variant 2	0.9080295	0.30041062
NM_000693	aldehyde dehydrogenase 1 family, member A3 (ALDH1A3)	0.90782606	0.35555268
NM_148979	cathepsin H (CTSH), transcript variant 2	0.90411449	0.33908081
BC043401	hypothetical protein MGC2752	0.9022628	0.19102131
NM_025079	zinc finger CCCH-type containing 12A (ZC3H12A)	0.89537853	0.1308531
DQ266251	Macaca fascicularis 17-beta hydroxysteroid dehydrogenase 5 (HSD17B5) mRNA, complete cds [DQ266251]	0.89265058	0.14413165

NM_001647	apolipoprotein D (APOD)	0.89161105	0.27673915
XR_010082	neutrophil cytosolic factor 4 (40kD) isoform 1 (LOC695612)	0.89072188	0.19367102
NM_018836	transmembrane protein SHREW1 (SHREW1)	0.88115817	0.22845938
NM_005461	v-maf musculoaponeurotic fibrosarcoma oncogene homolog B (avian) (MAFB)	0.88112651	0.23969467
CO647394	ILLUMIGEN_MCQ_40429 Katze_MMPB2 cDNA clone IBIUW:24175 5' Bases 238 to 766 highly human Unigene Hs.517602	0.87917407	0.03482732
NM_001637	acyloxyacyl hydrolase (neutrophil) (AOAH)	0.87622616	0.08210984
CR603940	full-length cDNA clone CS0DI086YN12 of Placenta Cot 25-normalized of (human) [CR603940]	0.87479194	0.27425632
NM_000617	solute carrier family 11 (proton-coupled divalent metal ion transporters), member 2 (SLC11A2)	0.87331284	0.0614324
NM_032801	junctional adhesion molecule 3 (JAM3)	0.87078026	0.23790107
XM_496386	Fc gamma receptor type I (LOC440607)	0.87040965	0.18245429
NM_005239	v-ets erythroblastosis virus E26 oncogene homolog 2 (avian) (ETS2)	0.86992237	0.06226559
NM_004750	cytokine receptor-like factor 1 (CRLF1)	0.86669981	0.08617555
NM_006762	Lysosomal-associated multispanning membrane protein-5 (LAPTM5)	0.8666752	0.03392545
NM_176798	pyrimidinergic receptor P2Y, G-protein coupled, 6 (P2RY6), transcript variant 2	0.86658655	0.07686848
NM_002068	guanine nucleotide binding protein (G protein), alpha 15 (Gq class) (GNA15)	0.86642833	0.06197955
NM_001005498	rhomboid, veinlet-like 6 (Drosophila) (RHBDL6), transcript variant 2	0.86591023	0.156759
DR771828	ILLUMIGEN_MCQ_10784 Katze_MMPL2 cDNA clone IBIUW:37887 5' Bases 199 to 549 highly human SLC7A2 (Hs.448520)	0.86392207	0.00131863
CN644277	ILLUMIGEN_MCQ_10044 Katze_MMPL2 cDNA clone IBIUW:9084 5' Bases 7 to 588 highly human TFRC (Hs.185726)	0.8594252	0.01607103
NM_002040	GA binding protein transcription factor, alpha subunit 60kDa (GABPA)	0.85885564	0.13313721
NM_156039	colony stimulating factor 3 receptor (granulocyte) (CSF3R), transcript variant 3	0.85859042	0.09770033
NM_001920	decorin (DCN), transcript variant A1	0.85825505	0.14087979
NM_015900	phospholipase A1 member A (PLA1A)	0.85792768	0.22663011
NM_002197	aconitase 1, soluble (ACO1)	0.8568742	0.09223792
NM_006509	v-rel reticuloendotheliosis viral oncogene homolog B, nuclear factor of kappa light polypeptide gene enhancer in B-cells 3 (avian) (RELB)	0.85512967	0.04598139

CN641959	ILLUMIGEN_MCQ_5696 Katze_MMBR cDNA clone IBIUW:5372 5' Bases 23 to 1049 highly human PRKAR2B (Hs.77439)	0.85443474	0.15507588
NM_005195	CCAAT/enhancer binding protein (C/EBP), delta (CEBPD)	0.85059404	0.15792303
NM_021101	claudin 1 (CLDN1)	0.84877166	0.1723594
NM_005797	epithelial V-like antigen 1 (EVA1), transcript variant 1	0.8446329	0.1074125
NM_013370	pregnancy-induced growth inhibitor (OKL38)	0.84286188	0.20042075
NM_015488	myofibrillogenesis regulator 1 (MR-1)	0.84257395	0.18831791
CN642751	ILLUMIGEN_MCQ_6807 Katze_MMPL2 cDNA clone IBIUW:4441 5' Bases 11 to 651 highly human Unigene Hs.303284	0.84137058	0.10241279
NM_006151	lactoperoxidase (LPO)	0.83960497	0.10738693
NM_016293	bridging integrator 2 (BIN2)	0.8389408	0.05085104
NM_002988	chemokine (C-C motif) ligand 18 (pulmonary and activation-regulated) (CCL18)	0.83748117	0.18750154
NM_020974	signal peptide, CUB domain, EGF-like 2 (SCUBE2)	0.83645278	0.12245671
NM_004864	growth differentiation factor 15 (GDF15)	0.83111452	0.08497041
NM_003956	cholesterol 25-hydroxylase (CH25H)	0.82875137	0.04977702
NM_004556	nuclear factor of kappa light polypeptide gene enhancer in B-cells inhibitor, epsilon (NFKBIE)	0.8278669	0.01013288
NM_005479	frequently rearranged in advanced T-cell lymphomas (FRAT1), transcript variant 1	0.82781497	0.1679011
NM_017515	solute carrier family 35, member F2 (SLC35F2)	0.82498576	0.10460014
CO645321	ILLUMIGEN_MCQ_10653 Katze_MMPL2 cDNA clone IBIUW:21923 5' Bases 3 to 906 highly human SEC24A (Hs.211612)	0.82477087	0.05364644
NM_013372	gremlin 1 homolog, cysteine knot superfamily (Xenopus laevis) (GREM1)	0.82328282	0.03738378
CN644516	ILLUMIGEN_MCQ_10491 Katze_MMPL2 cDNA clone IBIUW:9332 5' Bases 1 to 959 highly human ARL7 (Hs.111554)	0.81980724	0.1784906
NM_176895	phosphatidic acid phosphatase type 2A (PPAP2A), transcript variant 2	0.8171761	0.17808642
CO648296	ILLUMIGEN_MCQ_41618 Katze_MMPB2 cDNA clone IBIUW:25660 5' Bases 4 to 498 highly human MGC50844 (Hs.27267)	0.81604475	0.12134598
NM_013372	gremlin 1 homolog, cysteine knot superfamily (Xenopus laevis) (GREM1)	0.81590568	0.03945095
NM_030957	a disintegrin-like and metalloprotease (reprolysin type) with thrombospondin type 1 motif, 10 (ADAMTS10)	0.8155715	0.06461382
NM_024644	chromosome 14 open reading frame 169 (C14orf169)	0.81528131	0.1486081

NM_006399	basic leucine zipper transcription factor, ATF-like (BATF)	0.81497267	0.0175158
BC090934	ribonuclease L (2',5'-oligoisoadenylate synthetase-dependent)	0.81307808	0.02211016
NM_004414	Down syndrome critical region gene 1 (DSCR1), transcript variant 1	0.81283088	0.08788444
NM_138771	alpha-1,3(6)-mannosylglycoprotein beta-1,6-N-acetyl-glucosaminyltransferase-like (LOC90693)	0.81167229	0.11258224
NM_003264	toll-like receptor 2 (TLR2)	0.81150708	0.11406014
CN644277	ILLUMIGEN_MCQ_10044 Katze_MMPL2 cDNA clone IBIUW:9084 5' Bases 7 to 588 highly human TFRC (Hs.185726)	0.81036953	0.1011622
NM_173050	signal peptide, CUB domain, EGF-like 1 (SCUBE1)	0.81028593	0.01886157
NM_001422	E74-like factor 5 (ets domain transcription factor) (ELF5), transcript variant 2	0.81005127	0.00304327
NM_006209	ectonucleotide pyrophosphatase/phosphodiesterase 2 (autotaxin) (ENPP2)	0.80905398	0.05105721
NM_004454	ets variant gene 5 (ets-related molecule) (ETV5)	0.8086315	0.03678348
NM_004318	aspartate beta-hydroxylase (ASPH), transcript variant 1	0.80859281	0.03838559
NM_152858	Wilms tumor 1 associated protein (WTAP), transcript variant 3	0.80591392	0.04987979
NM_006216	serine (or cysteine) proteinase inhibitor, clade E (nexin, plasminogen activator inhibitor type 1), member 2 (SERPINE2)	0.80569237	0.06102858
NM_001001691	FLJ44790 protein (FLJ44790)	0.80314082	0.095801
XM_033173	protocadherin 19 (PCDH19)	0.80050296	0.01843252
NM_013370	pregnancy-induced growth inhibitor (OKL38)	0.79908549	0.202122
NM_025176	KIAA0980 protein (KIAA0980)	0.79748149	0.14691044
NM_003177	spleen tyrosine kinase (SYK)	0.79164502	0.1415578
NM_144765	epithelial V-like antigen 1 (EVA1), transcript variant 2	0.7909308	0.1551211
NM_000484	amyloid beta (A4) precursor protein (protease nexin-II, Alzheimer disease) (APP), transcript variant 1	0.78945403	0.11029934
NM_014143	CD274 antigen (CD274)	0.78898794	0.08891918
CK231427	ILLUMIGEN_MCQ_2215 Katze_MMLG cDNA 5'	0.78607383	0.08076418
XR_012436	mannosidase, alpha, class 2A, member 1 (LOC705480)	0.78464545	0.02740787
NM_000803	folate receptor 2 (fetal) (FOLR2)	0.78165127	0.01335931
NM_198319	HMT1 hnRNP methyltransferase-like 2 (S. cerevisiae) (HRMT1L2), transcript variant 2	0.7789341	0.14894206

NM_023028	fibroblast growth factor receptor 2 (bacteria-expressed kinase, keratinocyte growth factor receptor, craniofacial dysostosis 1, Crouzon syndrome, Pfeiffer syndrome, Jackson-Weiss syndrome) (FGFR2), transcript variant 10	0.77699348	0.02372044
NM_148170	cathepsin C (CTSC), transcript variant 2	0.77650468	0.03235952
NM_014467	sushi-repeat-containing protein, X-linked 2 (SRPX2)	0.77600267	0.04415254
NM_022740	homeodomain interacting protein kinase 2 (HIPK2)	0.77586858	0.08666922
NM_021958	H2.0-like homeo box 1 (Drosophila) (HLX1)	0.77502063	0.00305296
NM_006667	progesterone receptor membrane component 1 (PGRMC1)	0.77448632	0.08969703
NM_016245	dehydrogenase/reductase (SDR family) member 8 (DHRS8)	0.77320975	0.12954052
NM_005428	vav 1 oncogene (VAV1)	0.77257738	0.07095571
NM_016354	solute carrier organic anion transporter family, member 4A1 (SLCO4A1)	0.77245863	0.05173379
NM_025250	tweety homolog 3 (Drosophila) (TTYH3)	0.76980326	0.13579889
NM_178443	UNC-112 related protein 2 (URP2), transcript variant URP2LF	0.7692745	0.07449234
NM_006207	platelet-derived growth factor receptor-like (PDGFRL)	0.7670032	0.07195607
NM_001175	Rho GDP dissociation inhibitor (GDI) beta (ARHGDIB)	0.76664147	0.08577633
NM_006717	spindlin (SPIN)	0.76645212	0.06199362
NM_000201	intercellular adhesion molecule 1 (CD54), human rhinovirus receptor (ICAM1)	0.76603805	0.0892765
NM_014376	cytoplasmic FMR1 interacting protein 2 (CYFIP2)	0.76582212	0.12345875
NM_005308	G protein-coupled receptor kinase 5 (GRK5)	0.76145018	0.05197038
NM_014552	transcription factor CP2-like 2 (TFCP2L2), transcript variant 1	0.76093255	0.01189043
NM_000104	cytochrome P450, family 1, subfamily B, polypeptide 1 (CYP1B1)	0.76067689	0.0755939
NM_138455	collagen triple helix repeat containing 1 (CTHRC1)	0.76055653	0.10096271
NM_002863	phosphorylase, glycogen; liver (Hers disease, glycogen storage disease type VI) (PYGL)	0.75941143	0.07395108
NM_001100	actin, alpha 1, skeletal muscle (ACTA1)	0.75901187	0.00916144
CK231427	ILLUMIGEN_MCQ_2215 Katze_MMLG cDNA 5'	0.75840919	0.10049637
NM_014020	LR8 protein (LR8)	0.75837038	0.09179017
NM_020529	nuclear factor of kappa light polypeptide gene enhancer in B-cells inhibitor, alpha (NFKBIA)	0.75788947	0.14745121
NM_138422	hypothetical protein BC011824 (LOC113179)	0.75685545	0.06966034
NM_006399	basic leucine zipper transcription factor, ATF-like (BATF)	0.75658966	0.10495705

NM_003706	phospholipase A2, group IVC (cytosolic, calcium-independent) (PLA2G4C)	0.75508731	0.10078128
NM_175892	hypothetical protein FLJ37266 (FLJ37266)	0.75350273	0.05722299
NM_023028	fibroblast growth factor receptor 2 (bacteria-expressed kinase, keratinocyte growth factor receptor, craniofacial dysostosis 1, Crouzon syndrome, Pfeiffer syndrome, Jackson-Weiss syndrome) (FGFR2), transcript variant 10	0.74979727	0.08691306
NM_001628	aldo-keto reductase family 1, member B1 (aldose reductase) (AKR1B1)	0.74880801	0.04696417
NM_021101	claudin 1 (CLDN1)	0.74750593	0.11441288
NM_152680	hypothetical protein FLJ32028 (FLJ32028)	0.74416889	0.00264921
NM_006526	zinc finger protein 217 (ZNF217)	0.74396122	0.12106577
NM_025079	zinc finger CCCH-type containing 12A (ZC3H12A)	0.74310484	0.02493327
NM_002009	fibroblast growth factor 7 (keratinocyte growth factor) (FGF7)	0.74164964	0.00326257
NM_017923	membrane-associated ring finger (C3HC4) 1 (MARCH1)	0.74038878	0.10520078
NM_006864	leukocyte immunoglobulin-like receptor, subfamily B (with TM and ITIM domains), member 3 (LILRB3)	0.73981591	0.03724629
NM_015238	KIBRA protein (KIBRA)	0.7387461	0.10300848
NM_023028	fibroblast growth factor receptor 2 (bacteria-expressed kinase, keratinocyte growth factor receptor, craniofacial dysostosis 1, Crouzon syndrome, Pfeiffer syndrome, Jackson-Weiss syndrome) (FGFR2), transcript variant 10	0.73454638	0.02340097
NM_213602	CD33 antigen-like 3 (CD33L3)	0.73353012	0.0840078
NM_000698	arachidonate 5-lipoxygenase (ALOX5)	0.73316647	0.04238856
NM_015339	activity-dependent neuroprotector (ADNP), transcript variant 1	0.73152247	0.04581683
NM_005194	CCAAT/enhancer binding protein (C/EBP), beta (CEBPB)	0.72935963	0.08602618
NM_006216	serine (or cysteine) proteinase inhibitor, clade E (nexin, plasminogen activator inhibitor type 1), member 2 (SERPINE2)	0.72721054	0.00337562
NM_017890	Cohen syndrome 1 (COH1), transcript variant 5	0.72379107	0.01641969
XR_012677	solute carrier family 13 member 3 (SLC13A3)	0.72197061	0.08214457
NM_016546	complement component 1, r subcomponent-like (C1RL)	0.72140082	0.08856142
NM_015900	phospholipase A1 member A (PLA1A)	0.71708881	0.02692805
NM_021149	coactosin-like 1 (Dictyostelium) (COTL1)	0.71575428	0.04150054
NM_001250	CD40 antigen (TNF receptor superfamily member 5) (CD40), transcript variant 1	0.71506883	0.00320543
NM_003264	toll-like receptor 2 (TLR2)	0.71460271	0.05533537

NM_194430	ribonuclease, RNase A family, 4 (RNASE4), transcript variant 1	0.71248888	0.04119776
NM_005756	G protein-coupled receptor 64 (GPR64)	0.70992779	0.05938918
NM_000115	endothelin receptor type B (EDNRB), transcript variant 1	0.70895284	0.0773297
NM_003177	spleen tyrosine kinase (SYK)	0.70754755	0.03296009
NM_003638	integrin, alpha 8 (ITGA8)	0.70529322	0.00838857
CN644277	ILLUMIGEN_MCQ_10044 Katze_MMPL2 cDNA clone IBIUW:9084 5' Bases 7 to 588 highly human TFRC (Hs.185726)	0.70528862	0.05063744
NM_001251	CD68 antigen (CD68)	0.7005715	0.00228958
CB548939	MMPL0007_F02 MMPL cDNA	0.6988964	0.00078496
NM_007256	solute carrier organic anion transporter family, member 2B1 (SLCO2B1)	0.6941995	0.0375761
NM_001005498	rhomboid, veinlet-like 6 (Drosophila) (RHBDL6), transcript variant 2	0.69324938	0.05844212
NM_198540	UDP-Gal:betaGal beta 1,3-galactosyltransferase polypeptide 7 (B3GALT7)	0.68442122	0.00406944
XM_290546	KIAA0830 protein (KIAA0830)	0.68241622	0.04578519
AK094654	cDNA FLJ37335 fis, clone BRAMY2020355 [AK094654]	0.68186608	0.02902299
NM_144503	F11 receptor (F11R), transcript variant 4	0.68088831	0.0049264
NM_016246	dehydrogenase/reductase (SDR family) member 10 (DHRS10)	0.67975778	0.02984864
NM_000295	serine (or cysteine) proteinase inhibitor, clade A (alpha-1 antiproteinase, antitrypsin), member 1 (SERPINA1), transcript variant 1	0.67714304	0.00399363
NM_016594	FK506 binding protein 11, 19 kDa (FKBP11)	0.66235804	0.0121382
NM_080873	ankyrin repeat and SOCS box-containing 11 (ASB11)	-3.83311315	2.2535863
NM_022085	thioredoxin domain containing 5 (TXNDC5), transcript variant 2	-3.67850506	3.75058665
NM_014479	ADAM-like, decysin 1 (ADAMDEC1)	-3.64796838	2.02804153
NM_000385	aquaporin 1 (channel-forming integral protein, 28kDa) (AQP1), transcript variant 2	-3.54141029	2.37786992
NM_024875	synaptopodin 2-like (SYNPO2L)	-3.41174611	1.56232254
NM_001659	ADP-ribosylation factor 3 (ARF3)	-3.24700217	0.44423361
NM_133510	RAD51-like 1 (S. cerevisiae) (RAD51L1), transcript variant 2	-3.15664702	1.33698091
AY954513	synaptotagmin XIV derived protein mRNA, complete cds [AY954513]	-3.03863883	1.35841137
CO579386	ILLUMIGEN_MCQ_50238 Katze_MMIL cDNA clone IBIUW:20499 5' Bases 5 to 300 highly human TFF1 (Hs.350470)	-2.91165505	2.08258829
NM_145208	methyl-CpG binding domain protein 3-like 1 (MBD3L1)	-2.87127086	1.28793901
XM_499343	paternally expressed 10 (PEG10)	-2.8675768	2.79246029

NM_014470	Rho family GTPase 1 (RND1)	-2.86025676	2.59260032
NM_004465	fibroblast growth factor 10 (FGF10)	-2.65677288	0.55234457
NM_181572	regulator of G-protein signalling like 1 (RGSL1)	-2.64641925	2.52174329
A_01_P005330	Unknown	-2.59998134	2.64970832
NM_033410	hypothetical protein MGC13138 (MGC13138)	-2.56898809	0.02030897
NM_006693	cleavage and polyadenylation specific factor 4, 30kDa (CPSF4)	-2.54929004	2.58557502
NM_001246	ectonucleoside triphosphate diphosphohydrolase 2 (ENTPD2), transcript variant 2	-2.46312396	0.40995479
NM_015533	DKFZP586B1621 protein (DKFZP586B1621)	-2.44420076	1.22704063
NM_199334	thyroid hormone receptor, alpha (erythroblastic leukemia viral (v-erb-a) oncogene homolog, avian) (THRA), transcript variant 1	-2.37457803	0.00112865
NM_005459	guanylate cyclase activator 1C (GUCA1C)	-2.37201155	1.84590048
NM_002776	kallikrein 10 (KLK10), transcript variant 1	-2.32628777	1.06239137
NM_030932	diaphanous homolog 3 (Drosophila) (DIAPH3)	-2.30726748	1.98242196
NM_181614	keratin associated protein 19-7 (KRTAP19-7)	-2.29139621	1.16379929
NM_002776	kallikrein 10 (KLK10), transcript variant 1	-2.27942183	1.03359788
NM_021948	brevican (BCAN), transcript variant 1	-2.24377404	1.86433468
NM_201400	hypothetical protein SB153 (SB153), transcript variant 1	-2.22636401	0.73520208
NM_013401	RAB3A interacting protein (rabin3)-like 1 (RAB3IL1)	-2.22232407	0.64343563
XR_013285	hemicentin 1 (LOC709816)	-2.22013076	1.19876009
NM_031912	synaptotagmin XV (SYT15), transcript variant a	-2.17841861	0.77372504
NM_031954	potassium channel tetramerisation domain containing 10 (KCTD10)	-2.17268504	1.58327774
NM_005419	signal transducer and activator of transcription 2, 113kDa (STAT2)	-2.13921708	1.96796193
NM_000506	coagulation factor II (thrombin) (F2)	-2.1277035	0.88431096
NM_016083	cannabinoid receptor 1 (brain) (CNR1), transcript variant 1	-2.1272205	1.63749599
XR_014412	hypothetical protein LOC717316 (LOC717316)	-2.12408476	0.95943912
NM_198098	aquaporin 1 (channel-forming integral protein, 28kDa) (AQP1), transcript variant 1	-2.09158161	0.91450131
NM_015599	phosphoglucomutase 3 (PGM3)	-2.0806819	0.47529416
NM_207339	P antigen family, member 2 (prostate associated) (PAGE2)	-2.05996397	1.95991632
CN641390	ILLUMIGEN_MCQ_4382 Katze_MMPL2 cDNA clone IBIUW:5627 5' Bases 5 to 631 highly human Unigene Hs.405253	-2.04485776	0.273944
NM_001005266	chromosome 15 open reading frame 21 (C15orf21), transcript variant 1	-2.00468974	0.94156386
NM_000095	cartilage oligomeric matrix protein (COMP)	-1.98511595	0.26468268
NM_019617	gastrokine 1 (GKN1)	-1.97518545	0.08837818
NM_000095	cartilage oligomeric matrix protein (COMP)	-1.97273592	0.24700175

NM_019013	family with sequence similarity 64, member A (FAM64A)	-1.92858571	0.32932783
NM_001786	cell division cycle 2, G1 to S and G2 to M (CDC2), transcript variant 1	-1.91863275	0.52679656
NM_001007532	saitohin (STH)	-1.91329617	0.0034501
NM_006424	solute carrier family 34 (sodium phosphate), member 2 (SLC34A2)	-1.89674251	1.29549516
NM_001004692	olfactory receptor, family 2, subfamily T, member 12 (OR2T12)	-1.86965459	0.53061445
NM_153225	RPE-spondin (RPESP)	-1.86924499	0.85993331
NM_025168	leucine rich repeat containing 1 (LRRC1)	-1.85800501	0.29665653
NM_198098	aquaporin 1 (channel-forming integral protein, 28kDa) (AQP1), transcript variant 1	-1.85039922	0.20423495
NM_080552	vesicular inhibitory amino acid transporter (VIAAT)	-1.8347005	1.49489524
XR_011953	Dihydrofolate reductase (LOC702446)	-1.83304532	1.11772549
NM_021619	PR domain containing 12 (PRDM12)	-1.83283905	0.08098559
NM_004094	eukaryotic translation initiation factor 2, subunit 1 alpha, 35kDa (EIF2S1)	-1.83083556	0.89703335
XR_012167	leucine rich repeat neuronal 3 (LOC701932)	-1.82446286	1.61414206
XM_370737	hypothetical protein FLJ10357 (FLJ10357)	-1.82256046	1.40702174
NM_003920	timeless homolog (Drosophila) (TIMELESS)	-1.81913695	1.5997422
NM_145654	RAD52 homolog B (S. cerevisiae) (RAD52B)	-1.8025612	0.13485532
NM_002036	Duffy blood group (FY)	-1.79667625	0.66401575
XR_010287	BUB1 budding uninhibited by benzimidazoles 1 homolog (LOC696598)	-1.78977221	0.22187642
NM_024102	MEP50 protein (MEP50)	-1.78009846	0.42536253
NM_174930	postmeiotic segregation increased 2-like 5 (PMS2L5)	-1.7792943	0.65174783
NM_014783	Rho GTPase activating protein 11A (ARHGAP11A)	-1.77608256	0.73689231
NM_033122	testis development protein NYD-SP26 (NYD-SP26)	-1.76543281	0.11122294
NM_022479	Williams-Beuren syndrome chromosome region 17 (WBSCR17)	-1.75243082	1.04531186
NM_019114	erythrocyte membrane protein band 4.1 like 4B (EPB41L4B)	-1.73975857	0.00248371
NM_015585	chromosome 20 open reading frame 26 (C20orf26)	-1.73658429	0.51063829
NM_152512	hypothetical protein FLJ25421 (FLJ25421)	-1.70660837	0.9435231
NM_033276	Ku70-binding protein 3 (KUB3)	-1.7062387	0.89342812
NM_018691	chromosome 5 open reading frame 3 (C5orf3)	-1.70516521	1.28221712
NM_024094	defective in sister chromatid cohesion homolog 1 (S. cerevisiae) (DCC1)	-1.69134099	0.11913397
NM_033084	Fanconi anemia, complementation group D2 (FANCD2)	-1.69005129	1.21303857
NM_007249	Kruppel-like factor 12 (KLF12), transcript variant 1	-1.68950112	0.21905069
NM_005604	POU domain, class 3, transcription factor 2 (POU3F2)	-1.68727869	1.25538383
NM_002023	fibromodulin (FMOD)	-1.68368302	0.1927644

NM_014571	hairy/enhancer-of-split related with YRPW motif-like (HEYL)	-1.68289418	0.08640917
NM_000174	glycoprotein IX (platelet) (GP9)	-1.67347604	0.75070618
NM_018492	PDZ binding kinase (PBK)	-1.65939874	0.56906264
NM_007314	v-abl Abelson murine leukemia viral oncogene homolog 2 (arg, Abelson-related gene) (ABL2), transcript variant b	-1.65633396	1.16267589
A_01_P018484	Unknown	-1.64865558	0.92552757
NM_000092	collagen, type IV, alpha 4 (COL4A4)	-1.64071415	0.07491768
NM_182798	hypothetical protein FLJ39155 (FLJ39155), transcript variant 2	-1.63924755	0.68370033
NM_014391	ankyrin repeat domain 1 (cardiac muscle) (ANKRD1)	-1.63280042	0.46004069
NM_001237	cyclin A2 (CCNA2)	-1.62974316	0.18785617
NM_130385	murine retrovirus integration site 1 homolog (MRVI1), transcript variant 2	-1.6227345	1.20043341
A_01_P011597	Unknown	-1.5983547	1.23491165
NM_032325	hypothetical protein MGC11102 (MGC11102)	-1.58793032	1.00343505
NM_005771	dehydrogenase/reductase (SDR family) member 9 (DHRS9), transcript variant 1	-1.58088243	0.56915999
NM_000908	natriuretic peptide receptor C/guanylate cyclase C (atrionatriuretic peptide receptor C) (NPR3)	-1.57012032	0.88185965
A_01_P008093	Unknown	-1.56423871	0.93119602
NM_031308	epiplakin 1 (EPPK1)	-1.56329142	0.4225333
NM_005214	cytotoxic T-lymphocyte-associated protein 4 (CTLA4)	-1.56045141	0.43167783
NM_138386	hypothetical protein BC008207 (LOC92345)	-1.5492763	1.1335224
NM_025045	hypothetical protein FLJ22582 (FLJ22582)	-1.54719723	0.23555366
NM_003530	histone 1, H3d (HIST1H3D)	-1.54151222	1.18451113
NM_001255	CDC20 cell division cycle 20 homolog (S. cerevisiae) (CDC20)	-1.53721906	0.14622517
NM_004350	runt-related transcription factor 3 (RUNX3)	-1.53081198	0.39027414
NM_003981	protein regulator of cytokinesis 1 (PRC1), transcript variant 1	-1.52636275	0.05988467
NM_181803	ubiquitin-conjugating enzyme E2C (UBE2C), transcript variant 6	-1.51859287	0.10263658
NM_181725	hypothetical protein FLJ12760 (FLJ12760)	-1.51582903	0.86520087
NM_001867	cytochrome c oxidase subunit VIIc (COX7C), nuclear gene encoding mitochondrial protein	-1.5101821	0.71013367
NM_001004698	olfactory receptor, family 2, subfamily W, member 5 (OR2W5)	-1.50260046	1.18041405
NM_001211	BUB1 budding uninhibited by benzimidazoles 1 homolog beta (yeast) (BUB1B)	-1.49650859	0.90965498
NM_020678	HT017 protein (HT017)	-1.49025073	1.09617824
NM_153225	RPE-spondin (RPESP)	-1.48781926	0.23152695

A_01_P007283	Unknown	-1.48484193	0.04874158
NM_005309	glutamic-pyruvate transaminase (alanine aminotransferase) (GPT)	-1.4789279	0.89216434
NM_024772	zinc finger, MYM domain containing 1 (ZMYM1)	-1.47722475	0.96756332
NM_005844	HLA complex group 9 (HCG9)	-1.47505779	1.12526011
NM_024572	UDP-N-acetyl-alpha-D-galactosamine:polypeptide N-acetylgalactosaminyltransferase 14 (GalNAc-T14) (GALNT14)	-1.47456322	0.69923477
NM_030919	chromosome 20 open reading frame 129 (C20orf129)	-1.47449015	0.12088657
A_01_P008301	Unknown	-1.47430836	0.84028818
A_01_P014909	Unknown	-1.47404908	0.23820348
NM_019013	family with sequence similarity 64, member A (FAM64A)	-1.47179779	0.00924779
XR_014627	rhophilin-like protein (LOC721526)	-1.4664081	0.44030702
NM_214710	protease, serine-like 1 (PRSSL1)	-1.46031784	0.10402454
NM_025045	hypothetical protein FLJ22582 (FLJ22582)	-1.45672855	0.05403307
NM_017776	zinc finger protein 673 (ZNF673)	-1.4517288	0.16873193
NM_032268	zinc and ring finger 1 (ZNRF1)	-1.44830051	0.0130204
NM_015964	brain specific protein (CGI-38)	-1.44049293	0.14147977
NM_003617	regulator of G-protein signalling 5 (RGS5)	-1.43915061	0.03827831
NM_205841	serine protease inhibitor, Kazal type 6 (SPINK6)	-1.43670181	0.15769303
NM_153229	hypothetical protein FLJ33318 (FLJ33318)	-1.43613906	1.06356596
XR_014664	protein C, cardiac (MYBPC3)	-1.4342038	0.43975988
NM_003617	regulator of G-protein signalling 5 (RGS5)	-1.42453254	0.19843392
CN646916	ILLUMIGEN_MCQ_27395 Katze_MMBR cDNA clone IBIUW:8115 5' Bases 1 to 600 highly human DSCR6 (Hs.254560)	-1.41439895	0.78214536
NM_001463	frizzled-related protein (FRZB)	-1.40342713	0.11481311
NM_030919	chromosome 20 open reading frame 129 (C20orf129)	-1.40276534	0.09392123
XR_012085	forkhead box M1 isoform 3 (LOC708805)	-1.40129503	0.80525856
NM_001255	CDC20 cell division cycle 20 homolog (S. cerevisiae) (CDC20)	-1.39068426	0.23292029
NM_001004753	olfactory receptor, family 51, subfamily F, member 2 (OR51F2)	-1.39023202	0.21421378
NM_057749	cyclin E2 (CCNE2), transcript variant 1	-1.39012308	0.17983298
NM_152515	hypothetical protein FLJ40629 (FLJ40629)	-1.38566575	0.16985928
NM_001809	centromere protein A, 17kDa (CENPA)	-1.38341563	0.25950585
NM_181334	PRR5-ARHGAP8 fusion (LOC553158)	-1.38107556	0.10475837
XR_012588	myosin, heavy polypeptide 7B, cardiac muscle, beta (LOC712230)	-1.37946654	0.6962113
NM_002023	fibromodulin (FMOD)	-1.37469866	0.23424588
NM_018424	erythrocyte membrane protein band 4.1 like 4B (EPB41L4B)	-1.36762474	0.52876041
XR_010652	discs large homolog 7 (LOC696772)	-1.36445504	0.49393582

NM_182507	hypothetical protein LOC144501 (LOC144501)	-1.35573212	0.00398907
NM_020859	Shroom-related protein (ShrmL)	-1.35105135	0.34455218
NM_006108	spondin 1, extracellular matrix protein (SPON1)	-1.34843147	0.10952343
NM_013962	neuregulin 1 (NRG1), transcript variant GGF2	-1.34504885	0.69183617
NM_014391	ankyrin repeat domain 1 (cardiac muscle) (ANKRD1)	-1.34379507	0.01179891
NM_006198	Purkinje cell protein 4 (PCP4)	-1.33335168	0.84824516
NM_020897	hyperpolarization activated cyclic nucleotide-gated potassium channel 3 (HCN3)	-1.33077411	0.16777512
NM_005733	kinesin family member 20A (KIF20A)	-1.3292297	0.62393854
NM_153269	chromosome 20 open reading frame 96 (C20orf96)	-1.32423148	0.78946373
NM_024745	SHC SH2-domain binding protein 1 (SHCBP1)	-1.3216433	0.64277916
AF303085	epididymal-specific lipocalin LCN6 mRNA, complete cds [AF303085]	-1.31821885	0.23112456
NM_006845	kinesin family member 2C (KIF2C)	-1.31063721	0.06023232
XR_014664	protein C, cardiac (MYBPC3)	-1.30900944	0.02284906
XM_211305	hypothetical protein LOC284021 (LOC284021)	-1.30724048	0.25786695
NM_005549	potassium voltage-gated channel, shaker-related subfamily, member 10 (KCNA10)	-1.30024728	0.09402566
NM_022046	kallikrein 14 (KLK14)	-1.29636946	0.19763708
NM_181789	collomin (COLM)	-1.29127952	0.3140138
NM_032711	hypothetical protein MGC13090 (MGC13090)	-1.2881847	0.80816799
NM_000856	guanylate cyclase 1, soluble, alpha 3 (GUCY1A3)	-1.28763474	0.21419531
NM_022046	kallikrein 14 (KLK14)	-1.28716872	0.26380813
XR_012825	early B-cell factor 3 (LOC713536)	-1.2835775	0.70186226
NM_001645	apolipoprotein C-I (APOC1)	-1.28237843	0.19692413
NM_080668	cell division cycle associated 5 (CDCA5)	-1.28150596	0.10961434
NM_001091	amiloride binding protein 1 (amine oxidase (copper-containing)) (ABP1)	-1.27965671	0.36230283
NM_203349	rai-like protein (RaLP)	-1.27610569	0.52832887
XM_372411	olfactory receptor, family 5, subfamily AN, member 1 (OR5AN1)	-1.2748799	0.58252364
NM_004485	guanine nucleotide binding protein (G protein), gamma 4 (GNG4)	-1.27286559	0.38190925
NM_000088	collagen, type I, alpha 1 (COL1A1)	-1.27138966	0.25948062
XR_014094	Notch homolog 3 (NOTCH3)	-1.26577803	0.29209625
XM_116936	RIKEN cDNA 4832428D23 gene (LOC196541)	-1.26071821	0.43123067
NM_001211	BUB1 budding uninhibited by benzimidazoles 1 homolog beta (yeast) (BUB1B)	-1.25951214	0.37742362
NM_018693	F-box protein 11 (FBXO11), transcript variant 2	-1.25287654	0.55889874
NM_006025	26 serine protease (P11)	-1.25065684	0.29062238
NM_017440	Mdm4, transformed 3T3 cell double minute 1, p53 binding protein (mouse) (MDM1)	-1.24582655	0.35260127
NM_152512	hypothetical protein FLJ25421 (FLJ25421)	-1.24265709	0.09343406
NM_020890	KIAA1524 protein (KIAA1524)	-1.24191385	0.54015523

NM_000023	sarcoglycan, alpha (50kDa dystrophin-associated glycoprotein) (SGCA)	-1.2348251	0.349626
NM_017413	apelin, AGTRL1 ligand (APLN)	-1.23176497	0.71475818
NM_031966	cyclin B1 (CCNB1)	-1.22022087	0.17094109
NM_016613	hypothetical protein DKFZp434L142 (DKFZp434L142)	-1.22017102	0.05909632
NM_014571	hairy/enhancer-of-split related with YRPW motif-like (HEYL)	-1.2190427	0.05598701
NM_153370	protease inhibitor 16 (PI16)	-1.21903411	0.03290963
NM_013359	zinc finger protein 221 (ZNF221)	-1.2163062	0.2993435
NM_198501	FLJ42461 protein (FLJ42461)	-1.21557787	0.20944248
NM_206833	cortexin 1 (CTXN1)	-1.21385706	0.28058999
NM_004345	cathelicidin antimicrobial peptide (CAMP)	-1.21225622	0.66234813
NM_144665	sestrin 3 (SESN3)	-1.20650565	0.25532646
NM_152462	transmembrane protein 21A (TMEM21A)	-1.20364776	0.52996369
NM_025218	UL16 binding protein 1 (ULBP1)	-1.19927719	0.32239588
NM_005450	noggin (NOG)	-1.19764061	0.6485667
NM_021170	bHLH factor Hes4 (Hes4)	-1.19571668	0.17215723
NM_013302	eukaryotic elongation factor-2 kinase (EEF2K)	-1.19336249	0.74624902
NM_032829	hypothetical protein FLJ14721 (FLJ14721)	-1.19030668	0.60299556
NM_031300	MAX dimerization protein 3 (MXD3)	-1.18931354	0.021545
NM_001646	apolipoprotein C-IV (APOC4)	-1.1886891	0.1321837
XM_372842	olfactory receptor OR1-33 (LOC391211)	-1.18813792	0.41763401
NM_144717	fibronectin type III domain containing 6 (FNDC6)	-1.18629153	0.56723283
NM_005192	cyclin-dependent kinase inhibitor 3 (CDK2-associated dual specificity phosphatase) (CDKN3)	-1.18599412	0.01833797
XR_011982	DNA topoisomerase II, alpha isozyme (TOP2A)	-1.18234272	0.14089209
A_01_P010359	Unknown	-1.18123897	0.2084443
NM_001253	CDC5 cell division cycle 5-like (S. pombe) (CDC5L)	-1.17929139	0.31696841
XR_010652	discs large homolog 7 (LOC696772)	-1.17871613	0.6036788
NM_022744	hypothetical protein FLJ13868 (FLJ13868)	-1.17682074	0.55502139
NM_001786	cell division cycle 2, G1 to S and G2 to M (CDC2), transcript variant 1	-1.17575549	0.06627084
NM_005192	cyclin-dependent kinase inhibitor 3 (CDK2-associated dual specificity phosphatase) (CDKN3)	-1.16642173	0.10159082
XR_011039	serine [XR_011039]	-1.16394151	0.51161403
NM_005807	proteoglycan 4 (PRG4)	-1.16348398	0.03574015
NM_031948	pancreasin (MPN)	-1.1571764	0.56642649
NM_000523	homeo box D13 (HOXD13)	-1.15646406	0.16444782
NM_138376	tetratricopeptide repeat domain 5 (TTC5)	-1.15466326	0.18168781
CN646100	ILLUMIGEN_MCQ_25621 Katze_MMBR cDNA clone IBIUW:10916 5' Bases 6 to 961 highly human Unigene Hs.529393	-1.15458393	0.25871811
NM_203394	E2F transcription factor 7 (E2F7)	-1.15323771	0.01579719
NM_015931	fls485 (LOC51066)	-1.15253947	0.23866033
NM_152522	ADP-ribosylation-like factor 6-interacting protein 6 (MGC33864)	-1.1490452	0.13760684

NM_058173	small breast epithelial mucin (LOC118430)	-1.14826447	0.01421653
NM_031299	cell division cycle associated 3 (CDCA3)	-1.14715322	0.04231178
NM_144665	sestrin 3 (SESN3)	-1.14418517	0.08575874
NM_001237	cyclin A2 (CCNA2)	-1.14400148	0.67808221
NM_001001710	4931415M17 protein (LOC401565)	-1.14345037	0.24507468
XR_010727	hypothetical protein LOC696654 (LOC696654)	-1.14223861	0.01014123
NM_018248	nei endonuclease VIII-like 3 (E. coli) (NEIL3)	-1.14159772	0.06530322
NM_000088	collagen, type I, alpha 1 (COL1A1)	-1.14118299	0.05911703
NM_006101	kinetochore associated 2 (KNTC2)	-1.14055866	0.32289023
NM_001671	asialoglycoprotein receptor 1 (ASGR1)	-1.14029009	0.39445397
NM_173497	HECT domain containing 2 (HECTD2), transcript variant 2	-1.13925025	0.68897441
NM_018101	cell division cycle associated 8 (CDCA8)	-1.13671267	0.00688058
NM_022772	EPS8-like 2 (EPS8L2)	-1.13227192	0.05293446
NM_178229	IQ motif containing GTPase activating protein 3 (IQGAP3)	-1.13130957	0.41514673
NM_032803	solute carrier family 7 (cationic amino acid transporter, y+ system), member 3 (SLC7A3)	-1.12722183	0.19447561
NM_052956	butyryl Coenzyme A synthetase 1 (BUCS1)	-1.12485574	0.65001539
NM_198501	FLJ42461 protein (FLJ42461)	-1.12008926	0.25498074
NM_013359	zinc finger protein 221 (ZNF221)	-1.11943717	0.17456085
NM_031308	epiplakin 1 (EPPK1)	-1.11835389	0.05106372
NM_004701	cyclin B2 (CCNB2)	-1.11370081	0.23013017
NM_006824	EBNA1 binding protein 2 (EBNA1BP2)	-1.1100831	0.02293243
NM_182854	selectin ligand interactor cytoplasmic-1 (SLIC1)	-1.10921062	0.19675162
NM_001613	actin, alpha 2, smooth muscle, aorta (ACTA2)	-1.10708448	0.05876219
NM_001645	apolipoprotein C-I (APOC1)	-1.10690228	0.16855164
NM_182543	NOL1/NOP2/Sun domain family, member 6 (NSUN6)	-1.10655244	0.45428671
NM_020851	KIAA1465 protein (KIAA1465)	-1.10510948	0.46081601
NM_004867	integral membrane protein 2A (ITM2A)	-1.10176737	0.55861949
NM_006059	laminin, gamma 3 (LAMC3)	-1.10047517	0.16386929
NM_001613	actin, alpha 2, smooth muscle, aorta (ACTA2)	-1.10041287	0.05759031
A_01_P017300	Unknown	-1.0976269	0.41251657
NM_182645	vestigial like 2 (Drosophila) (VGLL2), transcript variant 1	-1.09724799	0.60748622
NM_022068	family with sequence similarity 38, member B (FAM38B)	-1.09576801	0.60056449
NM_144612	lipoxygenase homology domains 1 (LOXHD1)	-1.09251152	0.29100544
NM_018131	chromosome 10 open reading frame 3 (C10orf3)	-1.09116102	0.58514168
NM_003085	synuclein, beta (SNCB)	-1.0900515	0.58510713
NM_001115	adenylate cyclase 8 (brain) (ADCY8)	-1.08945999	0.39280789
NM_033208	tigger transposable element derived 7 (TIGD7)	-1.08782387	0.34261904
NM_014715	Rho GTPase-activating protein (RICS)	-1.08722594	0.40657977
NM_033516	protein kinase NYD-SP25 (NYD-SP25), transcript variant 1	-1.08487907	0.55007128

NM_003981	protein regulator of cytokinesis 1 (PRC1), transcript variant 1	-1.08254045	0.01958084
XR_000216	cysteine-rich hydrophobic domain 1 (CHIC1)	-1.08210231	0.03275491
NM_004789	LIM homeobox 2 (LHX2)	-1.08128234	0.02197331
NM_171998	RAB39B, member RAS oncogene family (RAB39B)	-1.08072851	0.08353324
NM_001790	cell division cycle 25C (CDC25C), transcript variant 1	-1.0793375	0.52971416
NM_000180	guanylate cyclase 2D, membrane (retina-specific) (GUCY2D)	-1.07869822	0.20955658
NM_001809	centromere protein A, 17kDa (CENPA)	-1.07539649	0.0958558
NM_004568	serine (or cysteine) proteinase inhibitor, clade B (ovalbumin), member 6 (SERPINB6)	-1.0749954	0.49991081
NM_198689	keratin associated protein 10-7 (KRTAP10-7)	-1.07410897	0.03708854
NM_138555	kinesin family member 23 (KIF23), transcript variant 1	-1.07158578	0.02857402
NM_003258	thymidine kinase 1, soluble (TK1)	-1.07044257	0.00415609
NM_007281	scrapie responsive protein 1 (SCRG1)	-1.06994642	0.43082775
NM_018186	chromosome 1 open reading frame 112 (C1orf112)	-1.06793185	0.15668863
NM_002466	v-myb myeloblastosis viral oncogene homolog (avian)-like 2 (MYBL2)	-1.06409442	0.06803876
NM_031299	cell division cycle associated 3 (CDCA3)	-1.05928016	0.12963898
NM_004701	cyclin B2 (CCNB2)	-1.05899647	0.10697744
NM_021025	T-cell leukemia homeobox 3 (TLX3)	-1.05473817	0.42938098
NM_178012	tubulin, beta polypeptide paralog (MGC8685)	-1.05422147	0.07546818
NM_015429	ABI gene family, member 3 (NESH) binding protein (ABI3BP)	-1.05411211	0.33006622
NM_007174	citron (rho-interacting, serine/threonine kinase 21) (CIT)	-1.05142852	0.0858353
NM_015429	ABI gene family, member 3 (NESH) binding protein (ABI3BP)	-1.05121336	0.17914578
NM_015163	tripartite motif-containing 9 (TRIM9), transcript variant 1	-1.04828882	0.32411352
NM_138568	protein 7 transactivated by hepatitis B virus X antigen (HBxAg) (XTP7)	-1.04446377	0.0504682
NM_002466	v-myb myeloblastosis viral oncogene homolog (avian)-like 2 (MYBL2)	-1.03941672	0.12701851
NM_001001995	glycoprotein M6B (GPM6B), transcript variant 1	-1.03862005	0.01169129
XR_010574	protein kinase Myt1 isoform 1 (LOC699350)	-1.0383739	0.2008077
NM_181519	synaptotagmin XV (SYT15), transcript variant b	-1.03726483	0.10100632
NM_000079	cholinergic receptor, nicotinic, alpha polypeptide 1 (muscle) (CHRNA1)	-1.03601892	0.15639194
XM_031561	chromosome 15 open reading frame 23 (C15orf23)	-1.03565947	0.14819944
NM_001885	crystallin, alpha B (CRYAB)	-1.03524503	0.12791236
NM_205848	synaptotagmin VI (SYT6)	-1.03267857	0.17409073
NM_003862	fibroblast growth factor 18 (FGF18), transcript variant 1	-1.03243747	0.14252157
NM_000394	crystallin, alpha A (CRYAA)	-1.03186924	0.37183273

NM_012310	kinesin family member 4A (KIF4A)	-1.03090233	0.09877531
NM_030945	C1q and tumor necrosis factor related protein 3 (C1QTNF3), transcript variant 1	-1.02981272	0.35512808
NM_014791	maternal embryonic leucine zipper kinase (MELK)	-1.02946579	0.30649448
NM_198278	hypothetical protein LOC255743 (LOC255743)	-1.02806669	0.10100659
NM_024891	hypothetical protein FLJ11783 (FLJ11783)	-1.0269064	0.39968309
NM_001012507	chromosome 6 open reading frame 173 (C6orf173)	-1.02103358	0.3466432
NM_207337	hypothetical protein LOC196394 (LOC196394)	-1.02028676	0.36698091
NM_032815	nuclear factor of activated T-cells, cytoplasmic, calcineurin-dependent 2 interacting protein (NFATC2IP)	-1.01993336	0.01295056
XM_031561	chromosome 15 open reading frame 23 (C15orf23)	-1.01810073	0.02085828
NM_001885	crystallin, alpha B (CRYAB)	-1.01372271	0.24675853
NM_024529	hyperparathyroidism 2 (with jaw tumor) (HRPT2)	-1.01289568	0.27002777
NM_033554	major histocompatibility complex, class II, DP alpha 1 (HLA-DPA1)	-1.00992021	0.30560243
CK231501	ILLUMIGEN_MCQ_2353 Katze_MMLG cDNA 5' human Unigene Hs.417764	-1.00869274	8.23E-05
NM_016613	hypothetical protein DKFZp434L142 (DKFZp434L142)	-1.00458744	0.23762234
NM_002371	mal, T-cell differentiation protein (MAL), transcript variant a	-1.00369525	0.31619415
NM_016343	centromere protein F, 350/400ka (mitosin) (CENPF)	-1.0035635	0.27612307
NM_152562	cell division cycle associated 2 (CDCA2)	-1.00275286	0.13271973
NM_007174	citron (rho-interacting, serine/threonine kinase 21) (CIT)	-0.99978419	0.00498343
NM_003542	histone 1, H4c (HIST1H4C)	-0.99682919	0.43329657
NM_031942	cell division cycle associated 7 (CDCA7), transcript variant 1	-0.99607371	0.45370972
XR_011982	DNA topoisomerase II, alpha isozyme (TOP2A)	-0.9956382	0.30833031
NM_018131	chromosome 10 open reading frame 3 (C10orf3)	-0.99330152	0.0430125
NM_017975	Zwilch (FLJ10036)	-0.99259435	0.13019225
NM_001001995	glycoprotein M6B (GPM6B), transcript variant 1	-0.98850499	0.14657251
NM_024910	hypothetical protein FLJ12700 (FLJ12700)	-0.98735794	0.44133832
NM_020236	mitochondrial ribosomal protein L1 (MRPL1), nuclear gene encoding mitochondrial protein	-0.98666868	0.10316474
NM_001012507	chromosome 6 open reading frame 173 (C6orf173)	-0.98332798	0.00029836
NM_004867	integral membrane protein 2A (ITM2A)	-0.98230257	0.16702339
NM_004137	potassium large conductance calcium-activated channel, subfamily M, beta member 1 (KCNMB1)	-0.97988272	0.39605843
NM_012112	TPX2, microtubule-associated, homolog (Xenopus laevis) (TPX2)	-0.97788474	0.20144015
NM_012310	kinesin family member 4A (KIF4A)	-0.97529613	0.27893497
NM_001010844	interleukin-1 receptor-associated kinase 1 binding protein 1 (IRAK1BP1)	-0.97503714	0.18860868
NM_001006618	mitogen-activated protein kinase associated protein 1 (MAPKAP1), transcript variant 6	-0.97338607	0.04653724

NM_017414	ubiquitin specific protease 18 (USP18)	-0.97271515	0.05132152
NM_014322	opsin 3 (encephalopsin, panopsin) (OPN3)	-0.96673402	0.2060966
NM_004153	origin recognition complex, subunit 1-like (yeast) (ORC1L)	-0.96456774	0.1554536
NM_007281	scrapie responsive protein 1 (SCRG1)	-0.96282026	0.13688637
A_01_P004221	Unknown	-0.96128427	0.33433758
NM_152500	coiled-coil domain containing 17 (CCDC17)	-0.9601934	0.24866979
XR_012298	Rough Deal homolog, centromere [XR_012298]	-0.95977767	0.43503143
NM_006843	serine dehydratase (SDS)	-0.95953211	0.07938126
CK231501	ILLUMIGEN_MCQ_2353 Katze_MMLG cDNA 5' human Unigene Hs.417764	-0.95855494	0.07665871
NM_003287	tumor protein D52-like 1 (TPD52L1)	-0.95374678	0.13814529
U65410	Human Mad2 (hsMAD2) mRNA, complete cds [U65410]	-0.95347504	0.21948002
NM_005857	zinc metalloproteinase (STE24 homolog, yeast) (ZMPSTE24)	-0.94953537	0.22011425
NM_012276	leukocyte immunoglobulin-like receptor, subfamily A (without TM domain), member 4 (ILT7)	-0.94934035	0.36288515
NM_018685	anillin, actin binding protein (scraps homolog, Drosophila) (ANLN)	-0.94813406	0.25118073
NM_080668	cell division cycle associated 5 (CDCA5)	-0.94262885	0.08480301
NM_152515	hypothetical protein FLJ40629 (FLJ40629)	-0.94242717	0.36886447
NM_181803	ubiquitin-conjugating enzyme E2C (UBE2C), transcript variant 6	-0.94209346	0.08691932
NM_020859	Shroom-related protein (ShrmL)	-0.94181926	0.20158077
NM_005030	polo-like kinase 1 (Drosophila) (PLK1)	-0.93984034	0.25457436
XR_012167	leucine rich repeat neuronal 3 (LOC701932)	-0.93933234	0.01535856
NM_014224	pepsinogen 5, group I (pepsinogen A) (PGA5)	-0.93775784	0.08378665
NM_014791	maternal embryonic leucine zipper kinase (MELK)	-0.92412832	0.04571578
NM_016954	T-box 22 (TBX22)	-0.92323782	0.14957112
NM_152607	hypothetical protein FLJ40201 (FLJ40201)	-0.92063826	0.03223051
NM_001463	frizzled-related protein (FRZB)	-0.91894358	0.16926211
NM_019111	major histocompatibility complex, class II, DR alpha (HLA-DRA)	-0.91603248	0.18624763
NM_003533	histone 1, H3i (HIST1H3I)	-0.91525413	0.03990667
NM_005382	neurofilament 3 (150kDa medium) (NEF3)	-0.91260193	0.15544359
NM_005030	polo-like kinase 1 (Drosophila) (PLK1)	-0.91170061	0.22668796
CN645773	ILLUMIGEN_MCQ_24841 Katze_MMPL2 cDNA clone IBIUW:10589 5' Bases 1 to 773 highly human FLJ10970 (Hs.173233)	-0.91055946	0.21516601
NM_005262	growth factor, augmenter of liver regeneration (ERV1 homolog, S. cerevisiae) (GFER)	-0.90908059	0.04900034
NM_018965	triggering receptor expressed on myeloid cells 2 (TREM2)	-0.90717589	0.10214014
NM_153041	hypothetical protein FLJ32955 (FLJ32955)	-0.8981376	0.28154663

NM_133637	DEAQ box polypeptide 1 (RNA-dependent ATPase) (DQX1)	-0.89720604	0.31410588
NM_002129	high-mobility group box 2 (HMGB2)	-0.89666703	0.04888027
NM_005771	dehydrogenase/reductase (SDR family) member 9 (DHRS9), transcript variant 1	-0.89489476	0.14680776
NM_019111	major histocompatibility complex, class II, DR alpha (HLA-DRA)	-0.89480751	0.0281341
NM_000426	laminin, alpha 2 (merosin, congenital muscular dystrophy) (LAMA2)	-0.89364517	0.12882398
NM_198468	chromosome 6 open reading frame 167 (C6orf167)	-0.89344139	0.24674774
NM_004137	potassium large conductance calcium-activated channel, subfamily M, beta member 1 (KCNMB1)	-0.89101303	0.13511374
CR604926	full-length cDNA clone CS0DF038YH05 of Fetal brain of (human) [CR604926]	-0.88946278	0.09750519
NM_152322	BTB (POZ) domain containing 11 (BTBD11), transcript variant 1	-0.88558134	0.28706886
NM_138983	oligodendrocyte transcription factor 1 (OLIG1)	-0.8852947	0.02725478
NM_004219	pituitary tumor-transforming 1 (PTTG1)	-0.88402402	0.07791442
NM_006101	kinetochore associated 2 (KNTC2)	-0.88257738	0.21719113
A_01_P017300	Unknown	-0.88096903	0.11072066
NM_001964	early growth response 1 (EGR1)	-0.88051395	0.22645345
NM_016095	DNA replication complex GINS protein PSF2 (Pfs2)	-0.880362	0.28696385
NM_018349	multiple C2-domains with two transmembrane regions 2 (MCTP2)	-0.87559686	0.12158021
XR_012719	alpha 2 type V collagen (COL5A2)	-0.87515799	0.15032946
NM_006262	peripherin (PRPH)	-0.87499919	0.08385426
NM_020205	zinc finger, A20 domain containing 1 (ZA20D1)	-0.87240991	0.29087099
NM_013282	ubiquitin-like, containing PHD and RING finger domains, 1 (UHRF1)	-0.87155865	0.18457534
NM_006059	laminin, gamma 3 (LAMC3)	-0.8703154	0.20561134
NM_173507	chromosome 1 open reading frame 127 (C1orf127)	-0.86414339	0.02075032
NM_004736	xenotropic and polytropic retrovirus receptor (XPR1)	-0.86279893	0.26159286
NM_014583	LIM and cysteine-rich domains 1 (LMCD1)	-0.86161341	0.01280286
NM_018410	hypothetical protein DKFZp762E1312 (DKFZp762E1312)	-0.8585959	0.20332727
NM_007208	mitochondrial ribosomal protein L3 (MRPL3), nuclear gene encoding mitochondrial protein	-0.85776823	0.29158557
NM_000426	laminin, alpha 2 (merosin, congenital muscular dystrophy) (LAMA2)	-0.85748002	0.11066326
NM_003287	tumor protein D52-like 1 (TPD52L1)	-0.85217237	0.24216733
NM_001153	annexin A4 (ANXA4)	-0.85183678	0.0201311
NM_053025	myosin, light polypeptide kinase (MYLK), transcript variant 1	-0.85180006	0.20579899

NM_002851	protein tyrosine phosphatase, receptor-type, Z polypeptide 1 (PTPRZ1)	-0.8516712	0.24046883
NM_002247	potassium large conductance calcium-activated channel, subfamily M, alpha member 1 (KCNMA1)	-0.8507177	0.2537013
XM_498423	interferon-induced protein with tetratricopeptide repeats 1 (IFIT1)	-0.85066893	0.13812795
XR_013013	Zinc finger CW-type PWWP domain protein 1 homolog (LOC711911)	-0.84980635	0.050152
NM_001063	transferrin (TF)	-0.84848068	0.07430355
NM_022045	Mdm2, transformed 3T3 cell double minute 2, p53 binding protein (mouse) binding protein, 104kDa (MTBP)	-0.84553794	0.15383264
NM_005609	phosphorylase, glycogen; muscle (McArdle syndrome, glycogen storage disease type V) (PYGM)	-0.84314006	0.18339892
XR_010412	hypothetical protein LOC696459 (LOC696459)	-0.84308824	0.06730736
NM_006583	retinal pigment epithelium-derived rhodopsin homolog (RRH)	-0.8425169	0.17836338
NM_005480	trophinin associated protein (tastin) (TROAP)	-0.84218331	0.25826958
NM_024649	Bardet-Biedl syndrome 1 (BBS1)	-0.84100147	0.09303458
NM_015931	fls485 (LOC51066)	-0.84016745	0.16940635
NM_014830	zinc finger and BTB domain containing 39 (ZBTB39)	-0.83699843	0.13227252
NM_018152	chromosome 20 open reading frame 12 (C20orf12)	-0.83583816	0.08544496
XM_375633	solute carrier family 8 (sodium-calcium exchanger), member 2 (SLC8A2)	-0.83354266	0.25895497
NM_015419	matrix-remodelling associated 5 (MXRA5)	-0.83245693	0.21306554
NM_138967	secretory carrier membrane protein 5 (SCAMP5)	-0.83168194	0.02806025
NM_018410	hypothetical protein DKFZp762E1312 (DKFZp762E1312)	-0.82423346	0.1476757
NM_022145	leucine zipper protein FKSG14 (FKSG14)	-0.82308494	0.12660068
NM_198586	NHL repeat containing 1 (NHLRC1)	-0.82124416	0.02576227
NM_003256	tissue inhibitor of metalloproteinase 4 (TIMP4)	-0.82122025	0.06628686
NM_003545	histone 1, H4e (HIST1H4E)	-0.81946384	0.03670178
XR_010287	BUB1 budding uninhibited by benzimidazoles 1 homolog (LOC696598)	-0.8179936	0.13617778
NM_144610	hypothetical protein FLJ25006 (FLJ25006)	-0.81757161	0.19587536
NM_000202	iduronate 2-sulfatase (Hunter syndrome) (IDS), transcript variant 1	-0.81750697	0.0617243
NM_138555	kinesin family member 23 (KIF23), transcript variant 1	-0.81354712	0.01924803
NM_007011	abhydrolase domain containing 2 (ABHD2), transcript variant 1	-0.81193507	0.12997558
NM_004852	one cut domain, family member 2 (ONECUT2)	-0.81145861	0.11161346
NM_015881	dickkopf homolog 3 (Xenopus laevis) (DKK3)	-0.80883136	0.01298837
NM_005202	collagen, type VIII, alpha 2 (COL8A2)	-0.80827093	0.16132906

XR_013214	protein phosphatase 2, regulatory subunit B, beta isoform 1 (LOC709587)	-0.80738026	0.15209658
NM_014620	homeo box C4 (HOXC4), transcript variant 1	-0.80580024	0.07160859
NM_013281	fibronectin leucine rich transmembrane protein 3 (FLRT3), transcript variant 1	-0.80540231	0.14283477
NM_004010	dystrophin (muscular dystrophy, Duchenne and Becker types) (DMD), transcript variant Dp427p2	-0.8042054	0.04573652
NM_006596	polymerase (DNA directed), theta (POLQ), transcript variant 1	-0.8009873	0.00871201
NM_015964	brain specific protein (CGI-38)	-0.79987145	0.03898606
NM_006461	sperm associated antigen 5 (SPAG5)	-0.7971544	0.01374262
NM_152562	cell division cycle associated 2 (CDCA2)	-0.79670847	0.0240695
NM_170587	regulator of G-protein signalling 20 (RGS20)	-0.79585208	0.04810306
NM_005261	GTP binding protein overexpressed in skeletal muscle (GEM), transcript variant 1	-0.79269283	0.08857911
NM_012211	integrin, alpha 11 (ITGA11), transcript variant 2	-0.7908085	0.04795663
NM_198278	hypothetical protein LOC255743 (LOC255743)	-0.79080266	0.07734223
XR_012719	alpha 2 type V collagen (COL5A2)	-0.79078503	0.07619029
NM_014583	LIM and cysteine-rich domains 1 (LMCD1)	-0.79059235	0.00152488
NM_017926	chromosome 14 open reading frame 118 (C14orf118), transcript variant 1	-0.78769067	0.09530816
NM_018101	cell division cycle associated 8 (CDCA8)	-0.78639475	0.0284573
NM_005556	keratin 7 (KRT7)	-0.78580873	0.04281161
NM_018965	triggering receptor expressed on myeloid cells 2 (TREM2)	-0.78520974	0.0462476
NM_003258	thymidine kinase 1, soluble (TK1)	-0.78291633	0.04689675
NM_000366	tropomyosin 1 (alpha) (TPM1)	-0.78211342	0.02243698
NM_152463	essential meiotic endonuclease 1 homolog 1 (S. pombe) (EME1)	-0.78144924	0.14016579
NM_181519	synaptotagmin XV (SYT15), transcript variant b	-0.77913802	0.08120991
NM_182507	hypothetical protein LOC144501 (LOC144501)	-0.77817232	0.10768026
NM_032552	DAB2 interacting protein (DAB2IP)	-0.77736306	0.10251143
NM_004802	otoferlin (OTOF), transcript variant 2	-0.77507872	0.15584663
NM_021170	bHLH factor Hes4 (Hes4)	-0.77488019	0.11947438
XR_012636	Wee1-like protein kinase (WEE1hu) (WEE1)	-0.77424689	0.01302852
NM_145060	chromosome 18 open reading frame 24 (C18orf24)	-0.77388248	0.11966015
NM_032495	homeodomain-only protein (HOP), transcript variant 1	-0.77332539	0.05142699
NM_178229	IQ motif containing GTPase activating protein 3 (IQGAP3)	-0.77242266	0.0001946
NM_002561	purinergic receptor P2X, ligand-gated ion channel, 5 (P2RX5), transcript variant 1	-0.77234513	0.02244128
NM_031912	synaptotagmin XV (SYT15), transcript variant a	-0.7714552	0.01501162
NM_005382	neurofilament 3 (150kDa medium) (NEF3)	-0.76887825	0.09640729
NM_006730	deoxyribonuclease I-like 1 (DNASE1L1)	-0.76796822	0.08135095
NM_012211	integrin, alpha 11 (ITGA11), transcript variant 2	-0.767684	0.06975706

NM_212482	fibronectin 1 (FN1), transcript variant 1	-0.76730563	0.0156735
NM_005841	sprouty homolog 1, antagonist of FGF signaling (Drosophila) (SPRY1), transcript variant 1	-0.76699357	0.0791092
NM_017752	FLJ20298 protein (FLJ20298), transcript variant 1	-0.7644561	0.05946445
NM_005025	serine (or cysteine) proteinase inhibitor, clade I (neuroserpin), member 1 (SERPINI1)	-0.75845781	0.08848883
NM_024028	hypothetical protein MGC3265 (MGC3265)	-0.7578108	0.04741947
NM_001004439	integrin, alpha 11 (ITGA11), transcript variant 1	-0.75747941	0.03098587
NM_133493	CD109 antigen (Gov platelet alloantigens) (CD109)	-0.75635485	0.05022281
NM_024647	nucleoporin 43kDa (NUP43), transcript variant 2	-0.75188213	0.13014931
NM_014176	HSPC150 protein ubiquitin-conjugating enzyme (HSPC150)	-0.75175614	0.02753593
NM_020783	synaptotagmin IV (SYT4)	-0.75073322	0.02579815
NM_005876	aortic preferentially expressed protein 1 (APEG1)	-0.74988958	0.05307467
CN647523	ILLUMIGEN_MCQ_28660 Katze_MMBR cDNA clone IBIUW:7531 5' Bases 1 to 826 highly human GFAP (Hs.406397)	-0.74926962	0.03673128
NM_025165	elongation factor RNA polymerase II-like 3 (ELL3)	-0.74908179	0.11126919
NM_212482	fibronectin 1 (FN1), transcript variant 1	-0.74812674	0.02189816
NM_016343	centromere protein F, 350/400ka (mitosin) (CENPF)	-0.74419384	0.05287495
NM_199206	T-cell leukemia/lymphoma 1B (TCL1B), transcript variant 2	-0.74316882	0.07999047
NM_005140	cyclic nucleotide gated channel alpha 2 (CNGA2)	-0.74245336	0.08912376
NM_003881	WNT1 inducible signaling pathway protein 2 (WISP2)	-0.73613345	0.11249117
NM_005824	leucine rich repeat containing 17 (LRRC17)	-0.73466621	0.06411692
NM_014783	Rho GTPase activating protein 11A (ARHGAP11A)	-0.73384832	0.08873345
NM_003979	retinoic acid induced 3 (RAI3)	-0.7338153	0.10375638
NM_004949	desmocollin 2 (DSC2), transcript variant Dsc2b	-0.7315453	0.10189699
NM_001442	fatty acid binding protein 4, adipocyte (FABP4)	-0.72798547	0.06881928
NM_031433	membrane frizzled-related protein (MFRP)	-0.72770862	0.07266796
NM_022785	CAP-binding protein complex interacting protein 1 (FLJ23588), transcript variant 1	-0.72658386	0.05333701
NM_152527	solute carrier family 16 (monocarboxylic acid transporters), member 14 (SLC16A14)	-0.72641091	0.05627223
NM_201591	glycoprotein M6A (GPM6A), transcript variant 2	-0.72251433	0.07589184
XM_374578	enhancer of zeste homolog 2 (Drosophila) (EZH2)	-0.71960807	0.05621929
NM_199295	cortistatin (CORT), transcript variant 5	-0.71948168	0.01936234
NM_001033	ribonucleotide reductase M1 polypeptide (RRM1)	-0.7187821	0.00191734
NM_002388	MCM3 minichromosome maintenance deficient 3 (S. cerevisiae) (MCM3)	-0.71373341	0.00639782
NM_004217	aurora kinase B (AURKB)	-0.71027856	0.06979755
NM_001646	apolipoprotein C-IV (APOC4)	-0.7091827	0.0788806
NM_000465	BRCA1 associated RING domain 1 (BARD1)	-0.70321857	0.02686151
NM_001823	creatine kinase, brain (CKB)	-0.70234972	0.0589376
NM_006959	zinc finger protein 17 (HPF3, KOX 10) (ZNF17)	-0.7021733	0.00965566

XR_012085	forkhead box M1 isoform 3 (LOC708805)	-0.70162079	0.03638229
XR_012471	phosphoserine aminotransferase isoform 2 (LOC711679)	-0.69469382	0.03476864
NM_138373	myeloid-associated differentiation marker (MYADM), transcript variant 2	-0.68836024	8.90E-05
NM_199039	kelch-like 5 (Drosophila) (KLHL5), transcript variant b	-0.68089243	0.02696541
NM_138450	ADP-ribosylation factor-like 11 (ARL11)	-0.66629906	0.00552052
CK230655	ILLUMIGEN_MCQ_1009 Katze_MMPL2 cDNA 5' human Unigene Hs.500464	-0.6620124	0.01182495
