# Supplementary material for: Microglia Are Mediators of Borrelia burgdorferi–Induced Apoptosis in SH-SY5Y Neuronal Cells
Source: PLoS Pathog. 2009 Nov 13;5(11):e1000659. doi: 10.1371/journal.ppat.1000659 (PMC2771360; doi:10.1371/journal.ppat.1000659)
Supplement: Table S3 — SY3D + B. burgdorferi vs. SY3D alone (0.28 MB PDF) [file ppat.1000659.s003.pdf]

Table S3: SY3D + B. burgdorferi vs. SY3D alone.

| GeneName | Description | Average Normalized Log2 Fold Change | Standard Deviation |
|---|---|---|---|
| A_24_P247454 | Unknown | 3.38193351 | 0.97350516 |
| ENST00000283760 | Unknown | 2.608580248 | 2.34600026 |
| NM_005163 | v-akt murine thymoma viral oncogene homolog 1 (AKT1), transcript variant 1, | 2.549581523 | 2.48381712 |
| A_24_P350060 | Unknown | 2.423944509 | 0.45465644 |
| NM_019886 | carbohydrate (N-acetylglucosamine 6-O) sulfotransferase 7 (CHST7), | 2.342018893 | 1.76898057 |
| A_24_P230057 | Unknown | 2.229561985 | 1.7904142 |
| A_24_P561165 | Unknown | 2.195816333 | 1.48711598 |
| NM_015975 | TAF9B RNA polymerase II, TATA box binding protein (TBP)-associated factor, 31kDa (TAF9B), | 2.158463499 | 2.03534785 |
| NM_001033515 | similar to hypothetical protein MGC27019 (LOC389833), | 2.113326565 | 1.90222995 |
| NM_004227 | pleckstrin homology, Sec7 and coiled-coil domains 3 (PSCD3), | 1.925010195 | 1.45495052 |
| NM_000319 | peroxisomal biogenesis factor 5 (PEX5), | 1.913717879 | 0.8555003 |
| NM_000582 | secreted phosphoprotein 1 (osteopontin, bone sialoprotein I, early T-lymphocyte activation 1) (SPP1), transcript variant 2, | 1.912394614 | 0.64960789 |
| NM_173624 | hypothetical protein FLJ40504 (FLJ40504), | 1.818939376 | 0.43599815 |
| BC032462 | vacuolar protein sorting 29 (yeast), | 1.796271442 | 0.81912227 |
| A_24_P584463 | Unknown | 1.791602956 | 0.08867162 |
| NM_016603 | chromosome 5 open reading frame 5 (C5orf5), | 1.770665268 | 1.6417262 |
| A_24_P306704 | Unknown | 1.749588712 | 0.07506165 |
| NM_145296 | immunoglobulin superfamily, member 4C (IGSF4C), | 1.704215386 | 1.56711322 |
| NM_000224 | keratin 18 (KRT18), transcript variant 1, | 1.691387253 | 0.12373115 |
| NM_000582 | secreted phosphoprotein 1 (osteopontin, bone sialoprotein I, early T-lymphocyte activation 1) (SPP1), transcript variant 2, | 1.678774873 | 0.22013525 |
| A_24_P418687 | Unknown | 1.668878619 | 0.90238319 |
| A_24_P383660 | Unknown | 1.661399336 | 0.64171853 |
| NM_014738 | KIAA0195 (KIAA0195), | 1.649341187 | 1.34017985 |
| T25391 | T25391 PTHI034 HTCDL1 cDNA 5'/3' similar to HepG2 3'-directed MboI gene, | 1.641092066 | 1.33165908 |
| NM_000224 | keratin 18 (KRT18), transcript variant 1, | 1.640914934 | 0.0433973 |
| NM_000224 | keratin 18 (KRT18), transcript variant 1, | 1.626358706 | 0.09770366 |
| NM_147777 | sorting nexin 15 (SNX15), transcript variant B, | 1.604850758 | 1.10529276 |
| NM_001552 | insulin-like growth factor binding protein 4 (IGFBP4), | 1.604561427 | 1.05584535 |
| THC2285293 | Unknown | 1.597545187 | 0.37875941 |
| NM_000582 | secreted phosphoprotein 1 (osteopontin, bone sialoprotein I, early T-lymphocyte activation 1) (SPP1), transcript variant 2, | 1.593956777 | 0.18858353 |
| CN414940 | 328755629 GRN_EB cDNA 5', | 1.567367215 | 1.04845219 |
| A_24_P186746 | Unknown | 1.54878396 | 1.29227692 |
| NM_006125 | Rho GTPase activating protein 6 (ARHGAP6), transcript variant 3, | 1.512160133 | 1.38052387 |
| NM_000582 | secreted phosphoprotein 1 (osteopontin, bone sialoprotein I, early T-lymphocyte activation 1) (SPP1), transcript variant 2, | 1.512097349 | 0.1738227 |
| NM_176814 | hypothetical protein LOC168850 (LOC168850), | 1.509282879 | 0.5746407 |
| NM_006315 | polycomb group ring finger 3 (PCGF3), | 1.505782722 | 1.3777283 |
| AK074056 | | 1.503362404 | 0.25595901 |

| | | | |
|---|---|---|---|
| NM_080861 | splA/ryanodine receptor domain and SOCS box containing 3 (SPSB3), | 1.482051228 | 1.32610525 |
| NM_000582 | secreted phosphoprotein 1 (osteopontin, bone sialoprotein I, early T-lymphocyte activation 1) (SPP1), transcript variant 2, | 1.475297424 | 0.25842083 |
| NM_014787 | DnaJ (Hsp40) homolog, subfamily C, member 6 (DNAJC6), | 1.470124762 | 0.14830155 |
| AK056176 | cDNA FLJ31614 fis, clone NT2RI2002970. [AK056176] | 1.463826117 | 1.27294201 |
| A_24_P161733 | Unknown | 1.455232235 | 0.08285255 |
| NM_000091 | collagen, type IV, alpha 3 (Goodpasture antigen) (COL4A3), transcript variant 1, | 1.436405883 | 1.42774258 |
| BX115105 | BX115105 Soares_pregnant_uterus_NbHPU cDNA clone IMAGp998H081169, | 1.434950147 | 0.8848046 |
| A_24_P255954 | Unknown | 1.430337462 | 0.04992742 |
| NM_012155 | echinoderm microtubule associated protein like 2 (EML2), | 1.428161047 | 0.96434574 |
| NM_014203 | adaptor-related protein complex 2, alpha 1 subunit (AP2A1), transcript variant 1, | 1.428063208 | 1.02244101 |
| BE739632 | BE739632 601556329T1 NIH_MGC_58 cDNA clone IMAGE:3826255 3', | 1.425535195 | 0.95160844 |
| AL050061 | | 1.420218615 | 0.24366658 |
| NM_022138 | SPARC related modular calcium binding 2 (SMOC2), | 1.420204969 | 0.10204897 |
| NM_002402 | mesoderm specific transcript homolog (mouse) (MEST), transcript variant 1, | 1.414727214 | 0.99068977 |
| NM_000582 | secreted phosphoprotein 1 (osteopontin, bone sialoprotein I, early T-lymphocyte activation 1) (SPP1), transcript variant 2, | 1.412864875 | 0.42383746 |
| AB007937 | | 1.409664815 | 0.90674799 |
| BC019824 | Homo sapiens, clone IMAGE:4454331, | 1.406225938 | 1.02524173 |
| NM_000582 | secreted phosphoprotein 1 (osteopontin, bone sialoprotein I, early T-lymphocyte activation 1) (SPP1), transcript variant 2, | 1.402931839 | 0.11712954 |
| A_24_P195164 | Unknown | 1.401779091 | 0.17991143 |
| NM_000582 | secreted phosphoprotein 1 (osteopontin, bone sialoprotein I, early T-lymphocyte activation 1) (SPP1), transcript variant 2, | 1.381931791 | 0.04825162 |
| THC2293989 | Unknown | 1.378129013 | 1.29330232 |
| NM_000582 | secreted phosphoprotein 1 (osteopontin, bone sialoprotein I, early T-lymphocyte activation 1) (SPP1), transcript variant 2, | 1.364080945 | 0.33629705 |
| NM_000214 | jagged 1 (Alagille syndrome) (JAG1), | 1.350214563 | 0.66855202 |
| NM_213604 | ADAMTS-like 5 (ADAMTSL5), | 1.331276846 | 0.07788179 |
| A_24_P16230 | Unknown | 1.327597418 | 0.36833913 |
| A_23_P122650 | Unknown | 1.315397788 | 0.30915108 |
| NM_005967 | NGFI-A binding protein 2 (EGR1 binding protein 2) (NAB2), | 1.299019207 | 0.44375759 |
| BC022083 | cDNA clone IMAGE:4825558. [BC022083] | 1.291531684 | 0.94010662 |
| BE798911 | 601585434F1 NIH_MGC_7 cDNA clone IMAGE:3939776 5', | 1.264817883 | 1.06714226 |
| NM_207382 | FLJ43276 protein (FLJ43276), | 1.250807939 | 0.40246108 |
| NM_015085 | GTPase activating Rap/RanGAP domain-like 4 (GARNL4), | 1.245010886 | 0.20857239 |
| NM_003239 | transforming growth factor, beta 3 (TGFB3), | 1.239682714 | 0.62687181 |
| A_24_P230466 | Unknown | 1.232587235 | 0.10404567 |
| NM_057159 | endothelial differentiation, lysophosphatidic acid G-protein-coupled receptor, 2 (EDG2), transcript variant 2, | 1.224059981 | 0.70547155 |
| NM_177974 | cancer susceptibility candidate 4 (CASC4), transcript variant 2, | 1.208408876 | 0.45998054 |
| A_24_P264293 | Unknown | 1.202434592 | 1.03554565 |
| NM_021937 | eukaryotic elongation factor, selenocysteine-tRNA-specific (EEFSEC), | 1.201319408 | 0.66397265 |
| NM_002442 | musashi homolog 1 (Drosophila) (MSI1), | 1.187025284 | 1.07921911 |
| NM_022373 | HERPUD family member 2 (HERPUD2), | 1.179967825 | 0.96442248 |
| NM_032133 | MYCBP associated protein (MYCBPAP), | 1.175576741 | 1.07411934 |

| | | | |
|---|---|---|---|
| NM_025220 | ADAM metallopeptidase domain 33 (ADAM33), transcript variant 1, | 1.168011706 | 0.92138696 |
| A_24_P398370 | Unknown | 1.165193971 | 0.19890523 |
| AK093641 | cDNA FLJ36322 fis, clone THYMU2005507. [AK093641] | 1.164063575 | 1.08044726 |
| THC2380963 | Q7SG75 (Q7SG75) Predicted protein, partial (3%) [THC2380963] | 1.157158692 | 0.88821072 |
| NM_004444 | EPH receptor B4 (EPHB4), | 1.156457359 | 0.64633197 |
| A_24_P247233 | Unknown | 1.14555477 | 0.04943564 |
| NM_133462 | tetratricopeptide repeat domain 14 (TTC14), | 1.144673394 | 1.02075602 |
| NM_024871 | MAP6 domain containing 1 (MAP6D1), | 1.140918939 | 0.73674028 |
| NM_012465 | tolloid-like 2 (TLL2), | 1.130102847 | 0.32380969 |
| NM_000625 | nitric oxide synthase 2A (inducible, hepatocytes) (NOS2A), transcript variant 1, | 1.12880445 | 0.19868146 |
| NM_025263 | proline rich 3 (PRR3), | 1.119116303 | 0.45858591 |
| NM_005386 | neuronatin (NNAT), transcript variant 1, | 1.115564048 | 0.04444799 |
| NM_000089 | collagen, type I, alpha 2 (COL1A2), | 1.102087498 | 0.67493507 |
| A_24_P780319 | Unknown | 1.10053941 | 0.25256002 |
| NM_015675 | growth arrest and DNA-damage-inducible, beta (GADD45B), | 1.088780438 | 0.04039946 |
| NM_007124 | utrophin (homologous to dystrophin) (UTRN), | 1.083542485 | 0.89096039 |
| NM_213662 | signal transducer and activator of transcription 3 (acute-phase response factor) (STAT3), transcript variant 3, | 1.076469398 | 0.90690321 |
| NM_001001522 | transgelin (TAGLN), transcript variant 1, | 1.075926447 | 0.28959908 |
| NM_004447 | epidermal growth factor receptor pathway substrate 8 (EPS8), | 1.06943426 | 0.14631359 |
| NM_032907 | ubiquitin-like 7 (bone marrow stromal cell-derived) (UBL7), transcript variant 1, | 1.067177286 | 0.00928878 |
| NM_016459 | proapoptotic caspase adaptor protein (PACAP), | 1.066086115 | 0.56735476 |
| A_24_P109661 | Unknown | 1.051033985 | 0.01156634 |
| NM_016593 | cytochrome P450, family 39, subfamily A, polypeptide 1 (CYP39A1), | 1.047117079 | 0.17178133 |
| NM_138957 | mitogen-activated protein kinase 1 (MAPK1), transcript variant 2, | 1.039465064 | 0.73527232 |
| NM_002834 | protein tyrosine phosphatase, non-receptor type 11 (Noonan syndrome 1) (PTPN11), | 1.038577176 | 0.731757 |
| NM_052937 | protein-L-isoaspartate (D-aspartate) O-methyltransferase domain containing 1 (PCMTD1), | 1.034523813 | 0.48541025 |
| NM_145232 | ATP binding domain 3 (ATPBD3), | 1.030742983 | 0.08637633 |
| ENST0000029554 9 | hypothetical gene supported by BC013438, | 1.023239537 | 0.10438943 |
| NM_002651 | phosphatidylinositol 4-kinase, catalytic, beta polypeptide (PIK4CB), | 1.021409761 | 0.3005223 |
| NM_000222 | v-kit Hardy-Zuckerman 4 feline sarcoma viral oncogene homolog (KIT), | 1.019765512 | 0.53631551 |
| NM_001456 | filamin A, alpha (actin binding protein 280) (FLNA), | 1.01512754 | 0.32046025 |
| A_23_P412927 | Unknown | 1.009254907 | 0.44949126 |
| NM_000222 | v-kit Hardy-Zuckerman 4 feline sarcoma viral oncogene homolog (KIT), | 1.00895309 | 0.13877666 |
| NM_025182 | KIAA1539 (KIAA1539), | 1.007966475 | 0.1185627 |
| NM_001002260 | chromosome 9 open reading frame 58 (C9orf58), transcript variant 2, | 1.001372383 | 0.74192805 |
| NM_001508 | G protein-coupled receptor 39 (GPR39), | 0.997726079 | 0.73246061 |
| NM_002254 | kinesin family member 3C (KIF3C), | 0.995780053 | 0.3027477 |
| NM_000582 | secreted phosphoprotein 1 (osteopontin, bone sialoprotein I, early T-lymphocyte activation 1) (SPP1), transcript variant 2, | 0.992037624 | 0.25614806 |
| NM_002521 | natriuretic peptide precursor B (NPPB), | 0.98945193 | 0.45539196 |
| NM_000222 | v-kit Hardy-Zuckerman 4 feline sarcoma viral oncogene homolog (KIT), | 0.989161198 | 0.38145618 |
| NM_000089 | collagen, type I, alpha 2 (COL1A2), | 0.988903225 | 0.21082552 |

| | | | |
|---|---|---|---|
| NM_003564 | transgelin 2 (TAGLN2), | 0.987972823 | 0.42720262 |
| NM_000089 | collagen, type I, alpha 2 (COL1A2), | 0.987158186 | 0.04829829 |
| NM_003162 | striatin, calmodulin binding protein (STRN), | 0.985578401 | 0.62333866 |
| NM_015944 | amidohydrolase domain containing 2 (AMDHD2), | 0.985110178 | 0.15376253 |
| NM_032536 | netrin G2 (NTNG2), | 0.983564653 | 0.0903417 |
| A_24_P384369 | Unknown | 0.982622209 | 0.07899849 |
| NM_198040 | polyhomeotic-like 2 (Drosophila) (PHC2), transcript variant 1, | 0.973673102 | 0.0476099 |
| A_24_P773958 | Unknown | 0.967368892 | 0.62751828 |
| NM_000089 | collagen, type I, alpha 2 (COL1A2), | 0.962299232 | 0.61122059 |
| THC2294276 | ALU2_HUMAN (P39189) Alu subfamily SB sequence contamination warning entry, partial (17%) [THC2294276] | 0.961504416 | 0.51367901 |
| NM_016352 | carboxypeptidase A4 (CPA4), | 0.961384834 | 0.0024916 |
| NM_001127 | adaptor-related protein complex 1, beta 1 subunit (AP1B1), transcript variant 1, | 0.961194445 | 0.38894725 |
| NM_002746 | mitogen-activated protein kinase 3 (MAPK3), transcript variant 1, | 0.959773712 | 0.06686768 |
| NM_003618 | mitogen-activated protein kinase kinase kinase kinase 3 (MAP4K3), | 0.9562818 | 0.70934384 |
| NM_023110 | fibroblast growth factor receptor 1 (fms-related tyrosine kinase 2, Pfeiffer syndrome) (FGFR1), transcript variant 1, | 0.955634589 | 0.66777059 |
| A_32_P58912 | Unknown | 0.955465345 | 0.34090212 |
| A_24_P596251 | Unknown | 0.952689213 | 0.54288622 |
| NM_153247 | solute carrier family 29 (nucleoside transporters), member 4 (SLC29A4), transcript variant 2, | 0.95135034 | 0.47880458 |
| NM_007101 | sarcosine dehydrogenase (SARDH), | 0.949176055 | 0.53321116 |
| NM_000089 | collagen, type I, alpha 2 (COL1A2), | 0.9447016 | 0.32966351 |
| NM_030801 | melanoma antigen family D, 4 (MAGED4), transcript variant 1, | 0.943197528 | 0.0141167 |
| NM_138473 | Sp1 transcription factor (SP1), | 0.941890847 | 0.57894421 |
| NM_152792 | Skin ASpartic Protease (FLJ25084), | 0.938977161 | 0.63874068 |
| A_24_P281374 | Unknown | 0.937007705 | 0.09056829 |
| NM_019105 | tenascin XB (TNXB), transcript variant XB, | 0.932827709 | 0.68991029 |
| NM_013320 | host cell factor C2 (HCFC2), | 0.92975043 | 0.31772032 |
| BE379389 | BE379389 601237074F1 NIH_MGC_44 cDNA clone IMAGE:3609162 5', | 0.929043176 | 0.59276172 |
| NM_006459 | SPFH domain family, member 1 (SPFH1), | 0.92895797 | 0.43751986 |
| NM_145245 | ecotropic viral integration site 5-like (EVI5L), | 0.928634372 | 0.6040299 |
| NM_002969 | mitogen-activated protein kinase 12 (MAPK12), | 0.928426046 | 0.23592383 |
| NM_017656 | zinc finger protein 562 (ZNF562), | 0.928186946 | 0.18366131 |
| NM_018936 | protocadherin beta 2 (PCDHB2), | 0.927925557 | 0.71892258 |
| NM_019009 | toll interacting protein (TOLLIP), | 0.923567365 | 0.15983779 |
| A_24_P937931 | Unknown | 0.923511884 | 0.54300765 |
| NM_001885 | crystallin, alpha B (CRYAB), | 0.918718758 | 0.17245673 |
| ENST00000328043 | low density lipoprotein receptor-related protein 11, | 0.915783454 | 0.10406327 |
| NM_031435 | THAP domain containing, apoptosis associated protein 2 (THAP2), | 0.911714682 | 0.09320236 |
| NM_000051 | ataxia telangiectasia mutated (includes complementation groups A, C and D) (ATM), transcript variant 1, | 0.907515079 | 0.06057136 |
| NM_000089 | collagen, type I, alpha 2 (COL1A2), | 0.905889818 | 0.4125338 |
| BE535360 | 601058513F1 NIH_MGC_10 cDNA clone IMAGE:3445322 5', | 0.901551288 | 0.58692246 |
| NM_019103 | zinc finger, matrin type 5 (ZMAT5), transcript variant 1, | 0.898696241 | 0.44567413 |
| NM_000222 | v-kit Hardy-Zuckerman 4 feline sarcoma viral oncogene homolog (KIT), | 0.898289617 | 0.13868307 |
| NM_001455 | forkhead box O3A (FOXO3A), transcript variant 1, | 0.895794618 | 0.32851495 |

| | | | |
|---|---|---|---|
| NM_198976 | TH1-like (Drosophila) (TH1L), transcript variant 1, | 0.890663622 | 0.04695408 |
| NM_006767 | leucine-zipper-like transcription regulator 1 (LZTR1), | 0.888743024 | 0.07810278 |
| NM_001164 | amyloid beta (A4) precursor protein-binding, family B, member 1 (Fe65) (APBB1), transcript variant 1, | 0.888280626 | 0.47922392 |
| THC2274051 | BOVPOUBA polyubiquitin {Bos taurus;} , partial (52%) [THC2274051] | 0.888220478 | 0.65146969 |
| NM_152617 | ring finger protein 168 (RNF168), | 0.886833785 | 0.20634158 |
| BC020868 | signal transducer and activator of transcription 5B, | 0.886719097 | 0.35054748 |
| NM_031284 | ADP-dependent glucokinase (ADPGK), | 0.884830486 | 0.11349848 |
| NM_177444 | PTPRF interacting protein, binding protein 1 (liprin beta 1) (PPFIBP1), transcript variant 2, | 0.881802582 | 0.60031904 |
| A_24_P358406 | Unknown | 0.881030372 | 0.20745319 |
| NM_021976 | retinoid X receptor, beta (RXRB), | 0.879778392 | 0.3134205 |
| NM_007121 | nuclear receptor subfamily 1, group H, member 2 (NR1H2), | 0.878645735 | 0.31493404 |
| NM_000222 | v-kit Hardy-Zuckerman 4 feline sarcoma viral oncogene homolog (KIT), | 0.876805281 | 0.20499907 |
| NM_032832 | low density lipoprotein receptor-related protein 11 (LRP11), | 0.876315388 | 0.48606992 |
| AK057762 | cDNA FLJ25033 fis, clone CBL02720. [AK057762] | 0.876084341 | 0.57239203 |
| AK001565 | cDNA FLJ10703 fis, clone NT2RP3000836. [AK001565] | 0.874677403 | 0.66723872 |
| AF338232 | CTAGE-4 protein | 0.869284041 | 0.13810405 |
| NM_005608 | protein tyrosine phosphatase, receptor type, C-associated protein (PTPRCAP), | 0.866819225 | 0.64450309 |
| NM_078471 | myosin XVIIIA (MYO18A), transcript variant 1, | 0.866808687 | 0.3717622 |
| NM_138384 | mitochondrial GTPase 1 homolog (S. cerevisiae) (MTG1), | 0.866170601 | 0.11551656 |
| NM_014748 | sorting nexin 17 (SNX17), | 0.864580129 | 0.11763261 |
| NM_001888 | crystallin, mu (CRYM), transcript variant 1, | 0.864559534 | 0.14049516 |
| NM_001010892 | radial spokehead-like 3 (RSHL3), | 0.864217323 | 0.35969552 |
| NM_016246 | dehydrogenase/reductase (SDR family) member 10 (DHRS10), | 0.864003626 | 0.22054854 |
| AK026368 | cDNA: FLJ22715 fis, clone HSI13726. [AK026368] | 0.863338659 | 0.58551487 |
| NM_006206 | platelet-derived growth factor receptor, alpha polypeptide (PDGFRA), | 0.861832855 | 0.07097264 |
| NM_024040 | CUE domain containing 2 (CUEDC2), | 0.858642611 | 0.05654784 |
| NM_012193 | frizzled homolog 4 (Drosophila) (FZD4), | 0.855074369 | 0.00566664 |
| NM_153482 | interleukin 17 receptor E (IL17RE), transcript variant 4, | 0.848657311 | 0.52748389 |
| NM_033513 | chromosome 19 open reading frame 20 (C19orf20), | 0.84720071 | 0.27129789 |
| NM_032143 | zinc finger, RAN-binding domain containing 3 (ZRANB3), | 0.84706762 | 0.61663632 |
| NM_004317 | arsA arsenite transporter, ATP-binding, homolog 1 (bacterial) (ASNA1), | 0.847050044 | 0.09473346 |
| NM_032019 | histone deacetylase 10 (HDAC10), | 0.84619142 | 0.37187707 |
| AA599881 | AA599881 ag32e07.s1 Human bone marrow stromal cells cDNA clone IMAGE:1091268 3' similar to gb:M21574 ALPHA PLATELET-DERIVED GROWTH FACTOR RECEPTOR PRECURSOR (HUMAN);, | 0.844640851 | 0.0796762 |
| NM_025129 | fuzzy homolog (Drosophila) (FUZ), | 0.840411784 | 0.01543831 |
| XM_934128 | PREDICTED: plasminogen-like A1, transcript variant 6 (PLGLA1), | 0.839363077 | 0.47306491 |
| THC2379691 | Unknown | 0.835658857 | 0.23598939 |
| NM_033198 | phosphatidylinositol glycan anchor biosynthesis, class S (PIGS), | 0.835000571 | 0.49526642 |
| NM_001015053 | histone deacetylase 5 (HDAC5), transcript variant 3, | 0.83225606 | 0.03799845 |
| XM_939847 | PREDICTED: similar to Williams Beuren syndrome chromosome region 19 (LOC650766), | 0.831731312 | 0.49247649 |
| NM_058230 | zinc finger protein 354B (ZNF354B), | 0.826754436 | 0.33735132 |
| NM_004383 | c-src tyrosine kinase (CSK), | 0.819312479 | 0.14615991 |
| NM_000041 | apolipoprotein E (APOE), | 0.818128607 | 0.18038782 |
| NM_144635 | chromosome 3 open reading frame 40 (C3orf40), | 0.817923533 | 0.1701737 |

| | | | |
|---|---|---|---|
| NM_079836 | tubulin, alpha 2 (TUBA2), transcript variant 2, | 0.815212832 | 0.3020545 |
| NM_002861 | phosphate cytidylyltransferase 2, ethanolamine (PCYT2), | 0.812571527 | 0.11561472 |
| NM_006339 | high-mobility group 20B (HMG20B), | 0.810459645 | 0.28289439 |
| AK127485 | cDNA FLJ45577 fis, clone BRTHA3011265, highly similar to Spindlin. [AK127485] | 0.808270509 | 0.57378077 |
| NM_007126 | valosin-containing protein (VCP), | 0.807120392 | 0.00445311 |
| NM_001018000 | kazrin (KIAA1026), transcript variant B, | 0.806571137 | 0.40656312 |
| NM_025104 | DBF4 homolog B (S. cerevisiae) (DBF4B), transcript variant 2, | 0.805473858 | 0.56911754 |
| ENST0000029554 9 | hypothetical gene supported by BC013438, | 0.805258081 | 0.15913749 |
| NM_004656 | BRCA1 associated protein-1 (ubiquitin carboxy-terminal hydrolase) (BAP1), | 0.803672698 | 0.02912321 |
| NM_005452 | WD repeat domain 46 (WDR46), | 0.800552619 | 0.20988965 |
| NM_005007 | nuclear factor of kappa light polypeptide gene enhancer in B-cells inhibitor-like 1 (NFKBIL1), | 0.797568465 | 0.0273867 |
| BC032064 | hypothetical gene supported by BC032064; BC041612, | 0.796778348 | 0.54621893 |
| NM_017789 | sema domain, immunoglobulin domain (Ig), transmembrane domain (TM) and short cytoplasmic domain, (semaphorin) 4C (SEMA4C), | 0.7959978 | 0.36791093 |
| NM_017432 | prostate tumor overexpressed gene 1 (PTOV1), | 0.795557975 | 0.04862514 |
| AF085896 | full length insert cDNA clone YQ05H10. [AF085896] | 0.794984998 | 0.19137693 |
| BC027471 | LOC440173, | 0.79345184 | 0.45977303 |
| NM_032529 | KIAA1875 (KIAA1875), | 0.791886324 | 0.22024539 |
| BF895757 | BF895757 RC3-MT0162-221100-012-h03 MT0162 cDNA, | 0.789660825 | 0.15590407 |
| NM_152529 | G protein-coupled receptor 155 (GPR155), transcript variant 2, | 0.789444916 | 0.18274997 |
| NM_014447 | ADP-ribosylation factor interacting protein 1 (arfaptin 1) (ARFIP1), transcript variant 3, | 0.789183824 | 0.35325593 |
| NM_024800 | NIMA (never in mitosis gene a)- related kinase 11 (NEK11), transcript variant 1, | 0.789172096 | 0.42918783 |
| NM_015381 | family with sequence similarity 19 (chemokine (C-C motif)-like), member A5 (FAM19A5), | 0.787783016 | 0.34907286 |
| NM_032169 | acyl-Coenzyme A dehydrogenase family, member 11 (ACAD11), | 0.787313082 | 0.20563903 |
| A_24_P910169 | Unknown | 0.780356139 | 0.41272138 |
| NM_001010000 | Rho GTPase activating protein 28 (ARHGAP28), transcript variant 1, | 0.779881633 | 0.37025893 |
| NM_024769 | adipocyte-specific adhesion molecule (ASAM), | 0.778661064 | 0.2846778 |
| NM_000259 | myosin VA (heavy polypeptide 12, myoxin) (MYO5A), | 0.777244753 | 0.50590209 |
| NM_002213 | integrin, beta 5 (ITGB5), | 0.772369869 | 0.02427703 |
| NM_004263 | sema domain, immunoglobulin domain (Ig), transmembrane domain (TM) and short cytoplasmic domain, (semaphorin) 4F (SEMA4F), | 0.771295079 | 0.43080933 |
| NR_002936 | transducer of ERBB2, 2 pseudogene (LOC222699) on chromosome 6 [NR_002936] | 0.76777094 | 0.45965993 |
| NM_024419 | phosphatidylglycerophosphate synthase (PGS1), | 0.76769157 | 0.28325748 |
| CB047924 | CB047924 NISC_gj01g10.x1 NCI_CGAP_Pr28 cDNA clone IMAGE:3270714 3', | 0.766506573 | 0.26931133 |
| NM_004364 | CCAAT/enhancer binding protein (C/EBP), alpha (CEBPA), | 0.765651554 | 0.11163539 |
| NM_003492 | chromosome X open reading frame 12 (CXorf12), | 0.764877505 | 0.20454249 |
| NM_014674 | ER degradation enhancer, mannosidase alpha-like 1 (EDEM1), | 0.759919649 | 0.18916604 |
| CR740121 | CR740121 library (Ebert L) cDNA clone IMAGp971G1750 ; IMAGE:767753 5', | 0.75883035 | 0.02554048 |
| NM_022918 | transmembrane protein 135 (TMEM135), | 0.758137532 | 0.03305994 |
| NM_206966 | similar to AVLV472 (MGC23985), | 0.756893232 | 0.1883622 |
| NM_004394 | death-associated protein (DAP), | 0.754120255 | 0.40302265 |
| THC2407039 | Unknown | 0.753930554 | 0.10904934 |
| AK097411 | cDNA FLJ40092 fis, clone TESTI2003756. [AK097411] | 0.752558442 | 0.49516216 |

| | | | |
|---|---|---|---|
| NM_000041 | apolipoprotein E (APOE), | 0.752319963 | 0.12054403 |
| NM_004765 | B-cell CLL/lymphoma 7C (BCL7C), | 0.751100544 | 0.08739233 |
| NM_032604 | abhydrolase domain containing 1 (ABHD1), | 0.745098396 | 0.41448034 |
| NM_006110 | CD2 (cytoplasmic tail) binding protein 2 (CD2BP2), | 0.744476407 | 0.45614393 |
| NM_022460 | HS1-binding protein 3 (HS1BP3), | 0.743390306 | 0.16518782 |
| NM_021649 | toll-like receptor adaptor molecule 2 (TICAM2), | 0.742350593 | 0.40449296 |
| ENST0000036714 2 | cDNA FLJ38536 fis, clone HCHON2001200. [AK095855] | 0.74176767 | 0.36276825 |
| NM_203339 | clusterin (CLU), transcript variant 2, | 0.741451866 | 0.12450569 |
| NM_001009812 | ladybird homeobox homolog 2 (Drosophila) (LBX2), | 0.741226304 | 0.45760449 |
| NM_002862 | phosphorylase, glycogen; brain (PYGB), | 0.741164458 | 0.07144196 |
| NM_184231 | NCK interacting protein with SH3 domain (NCKIPSD), transcript variant 2, | 0.734441879 | 0.01922706 |
| NM_002088 | glutamate receptor, ionotropic, kainate 5 (GRIK5), | 0.732108541 | 0.39279681 |
| NM_032701 | suppressor of variegation 4-20 homolog 2 (Drosophila) (SUV420H2), | 0.730824965 | 0.23986123 |
| AF218008 | clone PP3501 unknown | 0.730132107 | 0.0765327 |
| NM_017704 | ankyrin repeat domain 49 (ANKRD49), | 0.729671455 | 0.06866231 |
| NM_022450 | rhomboid 5 homolog 1 (Drosophila) (RHBDF1), | 0.728370262 | 0.41136627 |
| NM_006453 | transducin (beta)-like 3 (TBL3), | 0.727415834 | 0.20490433 |
| NM_002743 | protein kinase C substrate 80K-H (PRKCSH), transcript variant 1, | 0.725845543 | 0.33540066 |
| NM_178150 | F-box protein, helicase, 18 (FBXO18), transcript variant 2, | 0.722967194 | 0.30355986 |
| NM_004755 | ribosomal protein S6 kinase, 90kDa, polypeptide 5 (RPS6KA5), transcript variant 1, | 0.722911941 | 0.0113879 |
| NM_003481 | ubiquitin specific peptidase 5 (isopeptidase T) (USP5), | 0.721565745 | 0.11810158 |
| NM_175852 | taxilin alpha (TXLNA), | 0.720597528 | 0.30726966 |
| NM_022170 | Williams-Beuren syndrome chromosome region 1 (WBSCR1), transcript variant 1, | 0.72059087 | 0.01667989 |
| NM_080629 | collagen, type XI, alpha 1 (COL11A1), transcript variant B, | 0.720397316 | 0.31645789 |
| NM_004656 | BRCA1 associated protein-1 (ubiquitin carboxy-terminal hydrolase) (BAP1), | 0.716801455 | 0.30978989 |
| NM_000222 | v-kit Hardy-Zuckerman 4 feline sarcoma viral oncogene homolog (KIT), | 0.716653073 | 0.05666119 |
| NM_018972 | ganglioside-induced differentiation-associated protein 1 (GDAP1), transcript variant 1, | 0.715832426 | 0.24515913 |
| BC009078 | MGC17624 protein, | 0.715716246 | 0.12780688 |
| NM_178126 | hypothetical protein LOC162427 (LOC162427), | 0.713565285 | 0.14492209 |
| NM_014502 | PRP19/PSO4 pre- | 0.712871472 | 0.23640927 |
| NM_005576 | lysyl oxidase-like 1 (LOXL1), | 0.710476848 | 0.09513172 |
| NM_198317 | kelch-like 17 (Drosophila) (KLHL17), | 0.710263529 | 0.14085029 |
| NM_025129 | fuzzy homolog (Drosophila) (FUZ), | 0.709624972 | 0.12557132 |
| A_24_P375962 | Unknown | 0.709375482 | 0.07931897 |
| AK074776 | cDNA FLJ90295 fis, clone NT2RP2000240. [AK074776] | 0.706948707 | 0.07593044 |
| NM_001613 | actin, alpha 2, smooth muscle, aorta (ACTA2), | 0.706196814 | 0.16371803 |
| NM_031454 | selenoprotein O (SELO), | 0.703707446 | 0.22405631 |
| A_24_P549927 | Unknown | 0.703252074 | 0.38308829 |
| NM_138578 | BCL2-like 1 (BCL2L1), nuclear gene encoding mitochondrial protein, transcript variant 1, | 0.703077919 | 0.33770127 |
| NM_001496 | GDNF family receptor alpha 3 (GFRA3), | 0.702797374 | 0.15064704 |
| NM_005134 | protein phosphatase 4, regulatory subunit 1 (PPP4R1), transcript variant 2, | 0.700395343 | 0.24303287 |
| NM_212482 | fibronectin 1 (FN1), transcript variant 1, | 0.700375445 | 0.02629258 |
| NM_004723 | rho/rac guanine nucleotide exchange factor (GEF) 2 (ARHGEF2), | 0.699664192 | 0.32781461 |
| NM_005250 | forkhead box L1 (FOXL1), | 0.699156088 | 0.1337846 |

| | | | |
|---|---|---|---|
| NM_153693 | homeobox C6 (HOXC6), transcript variant 2, | 0.697573284 | 0.00552431 |
| NM_007257 | paraneoplastic antigen MA2 (PNMA2), | 0.697004007 | 0.38749155 |
| NM_019015 | chondroitin sulfate glucuronyltransferase (CSGlcA-T), | 0.69691031 | 0.06775806 |
| NM_000041 | apolipoprotein E (APOE), | 0.696482928 | 0.1011506 |
| NM_024589 | rogdi homolog (Drosophila) (ROGDI), | 0.695314521 | 0.26123855 |
| NM_000196 | hydroxysteroid (11-beta) dehydrogenase 2 (HSD11B2), | 0.694130535 | 0.39684132 |
| A_24_P761457 | Unknown | 0.693011739 | 0.01679851 |
| NM_015411 | sulfatase modifying factor 2 (SUMF2), transcript variant 2, | 0.692469969 | 0.12983954 |
| NM_199444 | coatomer protein complex, subunit epsilon (COPE), transcript variant 3, | 0.691986006 | 0.09711425 |
| NM_002502 | nuclear factor of kappa light polypeptide gene enhancer in B-cells 2 (p49/p100) (NFKB2), | 0.689910069 | 0.27069237 |
| NM_001967 | eukaryotic translation initiation factor 4A, isoform 2 (EIF4A2), | 0.688255835 | 0.01267933 |
| NM_144601 | CKLF-like MARVEL transmembrane domain containing 3 (CMTM3), transcript variant 1, | 0.687825232 | 0.05383664 |
| ENST00000332534 | Unknown | 0.686301305 | 0.03903021 |
| NM_014817 | KIAA0644 gene product (KIAA0644), | 0.685774843 | 0.2808258 |
| NM_024106 | zinc finger protein 426 (ZNF426), | 0.685477619 | 0.34550068 |
| ENST00000332750 | MUC3A | 0.682998706 | 0.32685938 |
| NM_019035 | protocadherin 18 (PCDH18), | 0.682453169 | 0.24237633 |
| XM_934787 | PREDICTED: similar to Acyl-protein thioesterase 2 (Lysophospholipase II) (LPL-I), transcript variant 3 (LOC653639), | 0.68123625 | 0.15545792 |
| NM_198229 | regulator of G-protein signalling 12 (RGS12), transcript variant 1, | 0.680744649 | 0.04939635 |
| NM_001002880 | PKD2 interactor, golgi and endoplasmic reticulum associated 1 (PGEA1), transcript variant 2, | 0.679081367 | 0.06540021 |
| NM_013986 | Ewing sarcoma breakpoint region 1 (EWSR1), transcript variant EWS-b, | 0.677668884 | 0.28484717 |
| NM_024722 | acyl-Coenzyme A binding domain containing 4 (ACBD4), | 0.677223318 | 0.09026824 |
| NM_006354 | transcriptional adaptor 3 (NGG1 homolog, yeast)-like (TADA3L), transcript variant 1, | 0.676550563 | 0.03922029 |
| NM_019085 | F-box and leucine-rich repeat protein 19 (FBXL19), | 0.675735647 | 0.1803717 |
| NM_152314 | chromosome 11 open reading frame 69 (C11orf69), | 0.67506812 | 0.01004722 |
| NM_004716 | proprotein convertase subtilisin/kexin type 7 (PCSK7), | 0.671905351 | 0.04938517 |
| NM_152605 | hypothetical protein FLJ37549 (FLJ37549), | 0.671078948 | 0.20324063 |
| NM_152519 | hypothetical protein FLJ23861 (FLJ23861), | 0.671036234 | 0.05702576 |
| NM_003615 | solute carrier family 4, sodium bicarbonate cotransporter, member 7 (SLC4A7), | 0.670838782 | 0.08252839 |
| A_24_P812265 | Unknown | 0.67060234 | 0.14398326 |
| NM_018358 | ATP-binding cassette, sub-family F (GCN20), member 3 (ABCF3), | 0.669410231 | 0.09085757 |
| NM_031229 | RanBP-type and C3HC4-type zinc finger containing 1 (RBCK1), transcript variant 2, | 0.669145968 | 0.05503433 |
| NM_145230 | ATPase, H+ transporting V0 subunit E2-like (rat) (ATP6V0E2L), | 0.668321183 | 0.02466938 |
| ENST00000244221 | cDNA FLJ37016 fis, clone BRACE2010632. [AK094335] | 0.668207719 | 0.33010017 |
| NM_025161 | chromosome 17 open reading frame 70 (C17orf70), | 0.668167543 | 0.2155789 |
| CR596550 | full-length cDNA clone CS0DA010YB10 of Neuroblastoma of (human). [CR596550] | 0.667648753 | 0.00146973 |
| NM_015398 | DKFZP564J102 protein (DKFZP564J102), transcript variant 1, | 0.666735375 | 0.12242799 |
| NM_001235 | serpin peptidase inhibitor, clade H (heat shock protein 47), member 1, (collagen binding protein 1) (SERPINH1), | 0.666636468 | 0.07691448 |
| NM_032831 | transmembrane protein 142B (TMEM142B), | 0.666340763 | 0.07336186 |
| NM_032038 | spinster (SPIN1), | 0.66537463 | 0.15976792 |

| | | | |
|---|---|---|---|
| NM_001014809 | collapsin response mediator protein 1 (CRMP1), transcript variant 1, | 0.664864954 | 0.28999465 |
| NM_017951 | neutral sphingomyelinase 3 (NSMASE3), transcript variant 2, | 0.664233328 | 0.37172343 |
| NM_002821 | PTK7 protein tyrosine kinase 7 (PTK7), transcript variant PTK7-1, | 0.664064759 | 0.23096655 |
| NM_138401 | hypothetical protein BC011840 (LOC93343), | 0.66397827 | 0.13088491 |
| NM_001294 | cleft lip and palate associated transmembrane protein 1 (CLPTM1), | 0.662153805 | 0.10841141 |
| NM_003359 | UDP-glucose dehydrogenase (UGDH), | 0.661634928 | 0.32677364 |
| NM_001006607 | c114 SLIT-like testicular protein (LOC474170), | 0.660700055 | 0.03519942 |
| NM_006302 | glucosidase I (GCS1), | 0.660336561 | 0.24208858 |
| NM_057161 | kelch domain containing 3 (KLHDC3), | 0.660118967 | 0.14087325 |
| NM_003819 | poly(A) binding protein, cytoplasmic 4 (inducible form) (PABPC4), | 0.659680814 | 0.21007526 |
| NM_020137 | GRIP1 associated protein 1 (GRIPAP1), transcript variant 1, | 0.658450908 | 0.14973695 |
| NM_003846 | peroxisomal biogenesis factor 11B (PEX11B), | 0.657916872 | 0.1134918 |
| NM_021070 | latent transforming growth factor beta binding protein 3 (LTBP3), | 0.65718975 | 0.32341653 |
| NM_198939 | prostaglandin E synthase 2 (PTGES2), transcript variant 3, | 0.655525639 | 0.27923899 |
| ENST0000030496 3 | | 0.655050988 | 0.29048926 |
| NM_001013398 | insulin-like growth factor binding protein 3 (IGFBP3), transcript variant 1, | 0.654490863 | 0.056506 |
| NM_006191 | proliferation-associated 2G4, 38kDa (PA2G4), | 0.654411295 | 0.17298145 |
| XM_927338 | PREDICTED: hypothetical protein LOC644117 (LOC644117), | 0.653937194 | 0.28431438 |
| NM_022145 | centromere protein K (CENPK), | 0.653193225 | 0.06822223 |
| NM_052987 | cyclin-dependent kinase (CDC2-like) 10 (CDK10), transcript variant 2, | 0.652958373 | 0.16782665 |
| NM_014849 | synaptic vesicle glycoprotein 2A (SV2A), | 0.652716042 | 0.31276034 |
| NM_032752 | zinc finger protein 496 (ZNF496), | 0.650438769 | 0.28684258 |
| NM_003089 | small nuclear ribonucleoprotein 70kDa polypeptide (RNP antigen) (SNRP70), transcript variant 1, | 0.648869777 | 0.14502899 |
| NM_005157 | v-abl Abelson murine leukemia viral oncogene homolog 1 (ABL1), transcript variant a, | 0.64742936 | 0.1427234 |
| NM_006185 | nuclear mitotic apparatus protein 1 (NUMA1), | 0.646891006 | 0.21467698 |
| ENST0000037124 2 | Unknown | 0.645702346 | 0.18773134 |
| NM_006565 | CCCTC-binding factor (zinc finger protein) (CTCF), | 0.64503277 | 0.09830865 |
| NM_016016 | CGI-69 protein (CGI-69), | 0.644589329 | 0.04781749 |
| NM_001005360 | dynamin 2 (DNM2), transcript variant 1, | 0.643641368 | 0.21012608 |
| NM_017861 | phosphatidylinositol glycan anchor biosynthesis, class X (PIGX), | 0.642716153 | 0.18079615 |
| NM_015650 | TNF receptor-associated factor 3 interacting protein 1 (TRAF3IP1), | 0.641657366 | 0.31589477 |
| THC2265989 | BI333945 602997212F1 NIH_MGC_12 cDNA clone IMAGE:5139483 5', | 0.638864176 | 0.13044813 |
| NM_020944 | glucosidase, beta (bile acid) 2 (GBA2), | 0.636690993 | 0.14798616 |
| BX393727 | BX393727 NEUROBLASTOMA COT 25-NORMALIZED cDNA clone CS0DC001YP02 5-PRIME, | 0.63646661 | 0.12362957 |
| NM_133640 | surfeit 5 (SURF5), transcript variant b, | 0.633715586 | 0.28766386 |
| NM_000180 | guanylate cyclase 2D, membrane (retina-specific) (GUCY2D), | 0.633700169 | 0.16947347 |
| ENST0000031825 1 | Unknown | 0.632262453 | 0.28933346 |
| NM_002473 | myosin, heavy polypeptide 9, non-muscle (MYH9), | 0.631540155 | 0.11757818 |
| NM_145233 | zinc finger protein 625 (ZNF625), | 0.630835359 | 0.30320077 |

| | | | |
|---|---|---|---|
| NM_005881 | branched chain ketoacid dehydrogenase kinase (BCKDK), | 0.630688637 | 0.26243016 |
| NM_003040 | solute carrier family 4, anion exchanger, member 2 (erythrocyte membrane protein band 3-like 1) (SLC4A2), | 0.630240857 | 0.05351835 |
| NM_003491 | ARD1 homolog A, N-acetyltransferase (S. cerevisiae) (ARD1A), | 0.629525697 | 0.20436728 |
| NM_080821 | chromosome 20 open reading frame 108 (C20orf108), | 0.62889716 | 0.11294536 |
| NM_033425 | DIX domain containing 1 (DIXDC1), transcript variant 2, | 0.62879497 | 0.17032599 |
| NM_005126 | nuclear receptor subfamily 1, group D, member 2 (NR1D2), | 0.627863975 | 0.091835 |
| NM_014341 | mitochondrial carrier homolog 1 (C. elegans) (MTCH1), nuclear gene encoding mitochondrial protein, | 0.627813706 | 0.16007077 |
| NM_014830 | zinc finger and BTB domain containing 39 (ZBTB39), | 0.626757819 | 0.28660506 |
| NM_001538 | heat shock transcription factor 4 (HSF4), transcript variant 1, | 0.62495647 | 0.14373527 |
| Z74615 | H.sapiens | 0.624920222 | 0.03818752 |
| NM_003246 | thrombospondin 1 (THBS1), | 0.624113739 | 0.01886047 |
| BC020336 | ribosomal protein S2, | 0.623507234 | 0.27255363 |
| NM_005163 | v-akt murine thymoma viral oncogene homolog 1 (AKT1), transcript variant 1, | 0.622942114 | 0.28698455 |
| NM_003780 | UDP-Gal:betaGlcNAc beta 1,4- galactosyltransferase, polypeptide 2 (B4GALT2), transcript variant 2, | 0.622254458 | 0.20850093 |
| NM_002394 | solute carrier family 3 (activators of dibasic and neutral amino acid transport), member 2 (SLC3A2), transcript variant 3, | 0.621456943 | 0.09459058 |
| NM_180989 | G protein-coupled receptor 180 (GPR180), | 0.621343614 | 0.14940635 |
| NM_016643 | mesenchymal stem cell protein DSC43 (LOC51333), | 0.621082971 | 0.11719965 |
| AK055407 | cDNA FLJ30845 fis, clone FEBRA2002727. [AK055407] | 0.619080237 | 0.30268791 |
| NM_013291 | cleavage and polyadenylation specific factor 1, 160kDa (CPSF1), | 0.618888727 | 0.19783824 |
| NM_152754 | sema domain, immunoglobulin domain (Ig), short basic domain, secreted, (semaphorin) 3D (SEMA3D), | 0.614989558 | 0.18081265 |
| AK055808 | cDNA FLJ31246 fis, clone KIDNE2005266. [AK055808] | 0.614909669 | 0.02239589 |
| NM_003765 | syntaxin 10 (STX10), | 0.613094669 | 0.00275824 |
| NM_014063 | drebrin-like (DBNL), transcript variant 1, | 0.612625454 | 0.16374783 |
| NM_144573 | nexilin (F actin binding protein) (NEXN), | 0.612568932 | 0.22111863 |
| NM_001299 | calponin 1, basic, smooth muscle (CNN1), | 0.611034195 | 0.06111348 |
| Z74615 | H.sapiens | 0.610964997 | 0.26034728 |
| NM_001004351 | similar to Williams Beuren syndrome chromosome region 19 (MGC57359), | 0.610611208 | 0.00839922 |
| AK056260 | cDNA FLJ31698 fis, clone NT2RI2005966. [AK056260] | 0.61018285 | 0.12283587 |
| NM_012268 | phospholipase D family, member 3 (PLD3), transcript variant 2, | 0.610137134 | 0.18351752 |
| NM_016219 | mannosidase, alpha, class 1B, member 1 (MAN1B1), | 0.609371149 | 0.21210911 |
| NM_007369 | G protein-coupled receptor 161 (GPR161), transcript variant 1, | 0.609149313 | 0.16198081 |
| A_24_P554882 | Unknown | 0.607533711 | 0.09966186 |
| NM_212482 | fibronectin 1 (FN1), transcript variant 1, | 0.607393648 | 0.21375026 |
| NM_033337 | caveolin 3 (CAV3), transcript variant 1, | 0.604523705 | 0.17415636 |
| BC033223 | solute carrier family 45, member 4, | 0.603926314 | 0.24413333 |
| NM_013443 | ST6 (alpha-N-acetyl-neuraminyl-2,3-beta-galactosyl-1,3)-N-acetylgalactosaminide alpha-2,6-sialyltransferase 6 (ST6GALNAC6), | 0.603598083 | 0.00889723 |
| ENST0000032480 8 | cDNA FLJ23754 fis, clone HEP17288. [AK074334] | 0.603283268 | 0.26692259 |
| NM_212482 | fibronectin 1 (FN1), transcript variant 1, | 0.603281289 | 0.18925174 |
| NM_023112 | OTU domain, ubiquitin aldehyde binding 2 (OTUB2), | 0.603251032 | 0.25679458 |
| NM_006814 | proteasome (prosome, macropain) inhibitor subunit 1 (PI31) (PSMF1), transcript variant 1, | 0.602599118 | 0.11675894 |
| NM_000041 | apolipoprotein E (APOE), | 0.60253763 | 0.14506592 |

| | | | |
|---|---|---|---|
| NM_002529 | neurotrophic tyrosine kinase, receptor, type 1 (NTRK1), transcript variant 2, | 0.602353907 | 0.14594587 |
| NM_003121 | Spi-B transcription factor (Spi-1/PU.1 related) (SPIB), mRNA [NM_003121] | -4.157724902 | 2.55198766 |
| NM_006398 | ubiquitin D (UBD), mRNA [NM_006398] | -4.021440594 | 0.1300189 |
| NM_181755 | hydroxysteroid (11-beta) dehydrogenase 1 (HSD11B1), transcript variant 2, mRNA [NM_181755] | -3.96677759 | 1.78959944 |
| THC2279115 | ALU8_(P39195) Alu subfamily SX sequence contamination warning entry, partial (12%) [THC2279115] | -3.890067984 | 1.39195201 |
| NM_032588 | tripartite motif-containing 63 (TRIM63), mRNA [NM_032588] | -3.613095736 | 1.72481875 |
| NM_005755 | Epstein-Barr virus induced gene 3 (EBI3), mRNA [NM_005755] | -3.601154046 | 0.13730778 |
| NM_005195 | CCAAT/enhancer binding protein (C/EBP), delta (CEBPD), mRNA [NM_005195] | -3.496713681 | 0.46938858 |
| NM_144586 | LY6/PLAUR domain containing 1 (LYPD1), mRNA [NM_144586] | -3.495382356 | 1.87504511 |
| NM_000201 | intercellular adhesion molecule 1 (CD54), rhinovirus receptor (ICAM1), mRNA [NM_000201] | -3.469523959 | 0.08006659 |
| NM_000201 | intercellular adhesion molecule 1 (CD54), rhinovirus receptor (ICAM1), mRNA [NM_000201] | -3.338850867 | 1.20120694 |
| NM_003897 | immediate early response 3 (IER3), transcript variant short, mRNA [NM_003897] | -3.325655008 | 0.69930199 |
| NM_007021 | chromosome 10 open reading frame 10 (C10orf10), mRNA [NM_007021] | -3.321539143 | 1.08516732 |
| NM_006291 | tumor necrosis factor, alpha-induced protein 2 (TNFAIP2), mRNA [NM_006291] | -3.262756544 | 0.59954217 |
| NM_153006 | N-acetylglutamate synthase (NAGS), mRNA [NM_153006] | -3.146070728 | 0.57922605 |
| NM_020705 | TBC1 domain family, member 24 (TBC1D24), mRNA [NM_020705] | -3.104053872 | 2.96359143 |
| NM_017855 | odontogenic, ameloblast asssociated (ODAM), mRNA [NM_017855] | -3.097229431 | 2.67452732 |
| NM_198941 | serine incorporator 3 (SERINC3), transcript variant 2, mRNA [NM_198941] | -3.086054595 | 2.38006003 |
| NM_001236 | carbonyl reductase 3 (CBR3), mRNA [NM_001236] | -3.000084444 | 2.69809247 |
| NM_000710 | bradykinin receptor B1 (BDKRB1), mRNA [NM_000710] | -2.97930219 | 1.31489879 |
| ENST0000038019 5 | piccolo (presynaptic cytomatrix protein), mRNA (cDNA clone IMAGE:3452412), complete cds. [BC001304] | -2.875344095 | 2.6213522 |
| NM_001017402 | laminin, beta 3 (LAMB3), transcript variant 2, mRNA [NM_001017402] | -2.853646397 | 0.30394119 |
| NM_000930 | plasminogen activator, tissue (PLAT), transcript variant 1, mRNA [NM_000930] | -2.805655038 | 0.06493534 |
| NM_015714 | G0/G1switch 2 (G0S2), mRNA [NM_015714] | -2.786786781 | 0.72283199 |
| NM_004688 | N-myc (and STAT) interactor (NMI), mRNA [NM_004688] | -2.758025392 | 1.1855156 |
| THC2279466 | Unknown | -2.755171924 | 1.47710957 |
| NM_000930 | plasminogen activator, tissue (PLAT), transcript variant 1, mRNA [NM_000930] | -2.740510029 | 0.16165239 |
| NM_017635 | suppressor of variegation 4-20 homolog 1 (Drosophila) (SUV420H1), transcript variant 1, mRNA [NM_017635] | -2.705736291 | 0.71154673 |
| NM_007350 | pleckstrin homology-like domain, family A, member 1 (PHLDA1), mRNA [NM_007350] | -2.687775459 | 1.16225535 |
| NM_004145 | myosin IXB (MYO9B), mRNA [NM_004145] | -2.686980471 | 1.94464133 |
| NM_000710 | bradykinin receptor B1 (BDKRB1), mRNA [NM_000710] | -2.673317179 | 0.37307239 |
| NM_172374 | interleukin 4 induced 1 (IL4I1), transcript variant 2, mRNA [NM_172374] | -2.654368293 | 0.37522549 |
| NM_000176 | nuclear receptor subfamily 3, group C, member 1 (glucocorticoid receptor) (NR3C1), transcript variant 5, mRNA [NM_000176] | -2.646730425 | 2.63795849 |
| NM_000930 | plasminogen activator, tissue (PLAT), transcript variant 1, mRNA [NM_000930] | -2.611496655 | 0.01415438 |
| NM_024817 | thrombospondin, type I, domain containing 4 (THSD4), mRNA [NM_024817] | -2.591886284 | 1.85068435 |
| NM_001165 | baculoviral IAP repeat-containing 3 (BIRC3), transcript variant 1, mRNA [NM_001165] | -2.59048119 | 0.21997037 |
| NM_004165 | Ras-related associated with diabetes (RRAD), mRNA [NM_004165] | -2.454810066 | 0.35254516 |
| NM_000930 | plasminogen activator, tissue (PLAT), transcript variant 1, mRNA [NM_000930] | -2.429629881 | 0.32810945 |
| NM_030934 | chromosome 1 open reading frame 25 (C1orf25), mRNA [NM_030934] | -2.412389903 | 2.18290089 |
| NM_000930 | plasminogen activator, tissue (PLAT), transcript variant 1, mRNA [NM_000930] | -2.403075334 | 0.15353252 |
| NM_004972 | Janus kinase 2 (a protein tyrosine kinase) (JAK2), mRNA [NM_004972] | -2.394691786 | 2.08842459 |
| NM_000930 | plasminogen activator, tissue (PLAT), transcript variant 1, mRNA [NM_000930] | -2.386475452 | 0.23776419 |

| | | | |
|---|---|---|---|
| THC2349894 | Q5TVW8 (Q5TVW8) ENSANGP00000029604 (Fragment), partial (6%) [THC2349894] | -2.382001842 | 2.02716319 |
| NM_018370 | hypothetical protein FLJ11259 (FLJ11259), mRNA [NM_018370] | -2.339656733 | 0.52597512 |
| NM_000930 | plasminogen activator, tissue (PLAT), transcript variant 1, mRNA [NM_000930] | -2.336412337 | 0.18691805 |
| NM_000201 | intercellular adhesion molecule 1 (CD54), rhinovirus receptor (ICAM1), mRNA [NM_000201] | -2.317561571 | 0.70688641 |
| NM_003913 | PRP4 pre-mRNA processing factor 4 homolog B (yeast) (PRPF4B), mRNA [NM_003913] | -2.312754802 | 2.16693041 |
| NM_000930 | plasminogen activator, tissue (PLAT), transcript variant 1, mRNA [NM_000930] | -2.295420474 | 0.34074721 |
| NM_006198 | Purkinje cell protein 4 (PCP4), mRNA [NM_006198] | -2.286080392 | 1.19657529 |
| NM_005658 | TNF receptor-associated factor 1 (TRAF1), mRNA [NM_005658] | -2.285762901 | 0.12221158 |
| NM_025216 | wingless-type MMTV integration site family, member 10A (WNT10A), mRNA [NM_025216] | -2.271148593 | 0.13308512 |
| NM_000930 | plasminogen activator, tissue (PLAT), transcript variant 1, mRNA [NM_000930] | -2.230829907 | 0.08494563 |
| NM_002341 | lymphotoxin beta (TNF superfamily, member 3) (LTB), transcript variant 1, mRNA [NM_002341] | -2.209864359 | 0.02640704 |
| NM_000201 | intercellular adhesion molecule 1 (CD54), rhinovirus receptor (ICAM1), mRNA [NM_000201] | -2.205490408 | 0.08906378 |
| NM_003264 | toll-like receptor 2 (TLR2), mRNA [NM_003264] | -2.192284324 | 2.19232966 |
| NM_000201 | intercellular adhesion molecule 1 (CD54), rhinovirus receptor (ICAM1), mRNA [NM_000201] | -2.184008612 | 0.29264442 |
| NM_000201 | intercellular adhesion molecule 1 (CD54), rhinovirus receptor (ICAM1), mRNA [NM_000201] | -2.162505161 | 0.219948 |
| NM_007350 | pleckstrin homology-like domain, family A, member 1 (PHLDA1), mRNA [NM_007350] | -2.113415291 | 0.4752333 |
| NM_153756 | fibronectin type III domain containing 5 (FNDC5), mRNA [NM_153756] | -2.107325597 | 0.0207477 |
| BC033126 | zinc finger protein 697, mRNA (cDNA clone IMAGE:3050333), complete cds. [BC033126] | -2.072409008 | 0.02082746 |
| ENST00000381577 | Unknown | -2.057716696 | 0.5282747 |
| NM_002658 | plasminogen activator, urokinase (PLAU), mRNA [NM_002658] | -2.053584943 | 2.05671762 |
| NM_000930 | plasminogen activator, tissue (PLAT), transcript variant 1, mRNA [NM_000930] | -2.04940734 | 0.05037698 |
| NM_000623 | bradykinin receptor B2 (BDKRB2), mRNA [NM_000623] | -2.044530259 | 0.39052508 |
| BC033086 | transcription factor 19 (SC1), mRNA (cDNA clone MGC:45652 IMAGE:3160434), complete cds. [BC033086] | -2.041321324 | 0.93426738 |
| NM_005258 | GTP cyclohydrolase I feedback regulator (GCHFR), mRNA [NM_005258] | -2.030104672 | 0.55536496 |
| NM_033452 | tripartite motif-containing 47 (TRIM47), mRNA [NM_033452] | -2.01857261 | 0.20048023 |
| NM_002996 | chemokine (C-X3-C motif) ligand 1 (CX3CL1), mRNA [NM_002996] | -2.007696774 | 0.73545037 |
| NM_003101 | sterol O-acyltransferase (acyl-Coenzyme A: cholesterol acyltransferase) 1 (SOAT1), transcript variant 688113, mRNA [NM_003101] | -1.97615138 | 0.59458246 |
| AB007895 | KIAA0435 mRNA, partial cds. [AB007895] | -1.962305771 | 1.67510238 |
| AF116619 | PRO1051 mRNA, complete cds. [AF116619] | -1.945508833 | 0.71626396 |
| BM989272 | BM989272 UI-H-DP0-ats-f-05-0-UI.s1 NCI_CGAP_Fs1 cDNA clone IMAGE:5863684 3', mRNA sequence [BM989272] | -1.931279259 | 1.36767128 |
| NM_024295 | Der1-like domain family, member 1 (DERL1), mRNA [NM_024295] | -1.918755986 | 1.33536664 |
| NM_031283 | transcription factor 7-like 1 (T-cell specific, HMG-box) (TCF7L1), mRNA [NM_031283] | -1.90645478 | 0.90106647 |
| NM_014045 | low density lipoprotein receptor-related protein 10 (LRP10), mRNA [NM_014045] | -1.900726057 | 1.40020997 |
| D87467 | mRNA for KIAA0277 gene, partial cds. [D87467] | -1.892030036 | 0.08081294 |
| BC017766 | polymerase (RNA) I polypeptide E, 53kDa, mRNA (cDNA clone IMAGE:4695468). [BC017766] | -1.851496748 | 0.33091314 |
| NM_002985 | chemokine (C-C motif) ligand 5 (CCL5), mRNA [NM_002985] | -1.833672452 | 0.31228968 |
| AK129982 | cDNA FLJ26472 fis, clone KDN04506. [AK129982] | -1.828092708 | 0.76710851 |
| NM_002485 | nibrin (NBN), transcript variant 1, mRNA [NM_002485] | -1.815405493 | 1.28784319 |
| NM_198181 | hypothetical protein LOC440295 (LOC440295), mRNA [NM_198181] | -1.814686761 | 0.48028161 |
| THC2379706 | Q86XQ1 (Q86XQ1) XTP9, partial (3%) [THC2379706] | -1.795193239 | 1.04549415 |
| THC2317058 | Unknown | -1.794916646 | 1.76065701 |
| ENST00000349637 | high molecular weight salivary mucin MG1 (MUC5B) mRNA, partial cds. [U63836] | -1.790720845 | 1.43514048 |

| | | | |
|---|---|---|---|
| NM_018370 | hypothetical protein FLJ11259 (FLJ11259), mRNA [NM_018370] | -1.775703297 | 0.58806725 |
| A_32_P220897 | Unknown | -1.773566175 | 1.43511144 |
| NM_013341 | GTP-binding protein PTD004 (PTD004), transcript variant 1, mRNA [NM_013341] | -1.763364944 | 1.34779565 |
| NM_001012631 | interleukin 32 (IL32), transcript variant 1, mRNA [NM_001012631] | -1.745933746 | 0.68192399 |
| A_32_P132928 | Unknown | -1.743105548 | 1.46300807 |
| NM_001346 | diacylglycerol kinase, gamma 90kDa (DGKG), mRNA [NM_001346] | -1.732750244 | 0.46772558 |
| NM_001955 | endothelin 1 (EDN1), mRNA [NM_001955] | -1.730603038 | 0.43409221 |
| NM_005230 | ELK3, ETS-domain protein (SRF accessory protein 2) (ELK3), mRNA [NM_005230] | -1.720774284 | 1.70594247 |
| NM_000594 | tumor necrosis factor (TNF superfamily, member 2) (TNF), mRNA [NM_000594] | -1.709941503 | 0.0229228 |
| NM_017786 | hypothetical protein FLJ20366 (FLJ20366), mRNA [NM_017786] | -1.705300531 | 0.10956061 |
| THC2342265 | O21342 (O21342) NADH dehydrogenase subunit 1, partial (5%) [THC2342265] | -1.701711943 | 0.14349271 |
| NM_002581 | pregnancy-associated plasma protein A, pappalysin 1 (PAPPA), mRNA [NM_002581] | -1.699931612 | 0.61837064 |
| NM_014143 | CD274 molecule (CD274), mRNA [NM_014143] | -1.67017827 | 1.60819581 |
| NM_199168 | chemokine (C-X-C motif) ligand 12 (stromal cell-derived factor 1) (CXCL12), transcript variant 1, mRNA [NM_199168] | -1.665384245 | 0.61923967 |
| NM_003825 | synaptosomal-associated protein, 23kDa (SNAP23), transcript variant 1, mRNA [NM_003825] | -1.661786036 | 0.86228298 |
| NM_007115 | tumor necrosis factor, alpha-induced protein 6 (TNFAIP6), mRNA [NM_007115] | -1.658051875 | 0.66066712 |
| NM_199168 | chemokine (C-X-C motif) ligand 12 (stromal cell-derived factor 1) (CXCL12), transcript variant 1, mRNA [NM_199168] | -1.655640248 | 0.28786397 |
| NM_002317 | lysyl oxidase (LOX), mRNA [NM_002317] | -1.648904571 | 0.03591007 |
| NM_005755 | Epstein-Barr virus induced gene 3 (EBI3), mRNA [NM_005755] | -1.63008657 | 0.47839454 |
| NM_152709 | storkhead box 1 (STOX1), mRNA [NM_152709] | -1.614777748 | 0.4859964 |
| AB051467 | mRNA for KIAA1680 protein, partial cds. [AB051467] | -1.612841428 | 1.18418745 |
| NM_178562 | tetraspanin 33 (TSPAN33), mRNA [NM_178562] | -1.601642341 | 1.59421137 |
| A_32_P205522 | Unknown | -1.590705561 | 0.93812442 |
| AK057576 | cDNA FLJ33014 fis, clone THYMU1000382. [AK057576] | -1.583226885 | 1.54888687 |
| NM_030791 | sphingosine-1-phosphate phosphatase 1 (SGPP1), mRNA [NM_030791] | -1.578305296 | 1.03571205 |
| NM_058197 | cyclin-dependent kinase inhibitor 2A (melanoma, p16, inhibits CDK4) (CDKN2A), transcript variant 3, mRNA [NM_058197] | -1.554174544 | 1.17943575 |
| NM_006038 | spermatogenesis associated 2 (SPATA2), mRNA [NM_006038] | -1.542707424 | 0.21409372 |
| NM_016072 | golgi transport 1 homolog B (S. cerevisiae) (GOLT1B), mRNA [NM_016072] | -1.538940705 | 0.52699823 |
| NM_152710 | chromosome 10 open reading frame 27 (C10orf27), mRNA [NM_152710] | -1.536657944 | 0.71194166 |
| NM_004165 | Ras-related associated with diabetes (RRAD), mRNA [NM_004165] | -1.534214621 | 0.62875251 |
| NM_001078 | vascular cell adhesion molecule 1 (VCAM1), transcript variant 1, mRNA [NM_001078] | -1.53102536 | 0.2956242 |
| ENST0000037895 3 | cDNA FLJ46914 fis, clone SPLEN2027852. [AK128882] | -1.528857985 | 0.80901439 |
| NM_025079 | zinc finger CCCH-type containing 12A (ZC3H12A), mRNA [NM_025079] | -1.525976649 | 0.60449176 |
| NM_003619 | protease, serine, 12 (neurotrypsin, motopsin) (PRSS12), mRNA [NM_003619] | -1.518104274 | 0.66580186 |
| NM_004726 | RALBP1 associated Eps domain containing 2 (REPS2), mRNA [NM_004726] | -1.501194401 | 0.64477522 |
| NM_152271 | LON peptidase N-terminal domain and ring finger 1 (LONRF1), mRNA [NM_152271] | -1.500077965 | 0.82515134 |
| NM_000539 | rhodopsin (opsin 2, rod pigment) (retinitis pigmentosa 4, autosomal dominant) (RHO), mRNA [NM_000539] | -1.490692232 | 0.91726401 |
| ENST0000035730 3 | cDNA FLJ37034 fis, clone BRACE2011478. [AK094353] | -1.48865319 | 1.15314771 |
| NM_198947 | family with sequence similarity 111, member B (FAM111B), mRNA [NM_198947] | -1.48546827 | 0.75777224 |

| | | | |
|---|---|---|---|
| NM_003260 | transducin-like enhancer of split 2 (E(sp1) homolog, Drosophila) (TLE2), mRNA [NM_003260] | -1.481043804 | 0.33544605 |
| NM_001955 | endothelin 1 (EDN1), mRNA [NM_001955] | -1.451734212 | 0.1355856 |
| NM_020714 | zinc finger protein 490 (ZNF490), mRNA [NM_020714] | -1.44975632 | 1.11175188 |
| AK094769 | cDNA FLJ37450 fis, clone BRAWH2010354, highly similar to MONOAMINE-SULFATING PHENOL SULFOTRANSFERASE (EC 2.8.2.1). [AK094769] | -1.449302247 | 0.65180596 |
| NM_002133 | heme oxygenase (decycling) 1 (HMOX1), mRNA [NM_002133] | -1.43779771 | 0.49518666 |
| NM_199168 | chemokine (C-X-C motif) ligand 12 (stromal cell-derived factor 1) (CXCL12), transcript variant 1, mRNA [NM_199168] | -1.436496755 | 0.44222161 |
| AK091439 | cDNA FLJ34120 fis, clone FCBBF3009541. [AK091439] | -1.434898853 | 0.58110341 |
| NM_013272 | solute carrier organic anion transporter family, member 3A1 (SLCO3A1), mRNA [NM_013272] | -1.43114461 | 0.3111765 |
| NM_003264 | toll-like receptor 2 (TLR2), mRNA [NM_003264] | -1.424769749 | 0.76183604 |
| NM_182901 | chromosome 11 open reading frame 17 (C11orf17), transcript variant 1, mRNA [NM_182901] | -1.418435963 | 0.78060469 |
| AK024186 | cDNA FLJ14124 fis, clone MAMMA1002498. [AK024186] | -1.41493505 | 1.10771876 |
| ENST00000341824 | Unknown | -1.411113751 | 1.32216463 |
| BC012029 | Homo sapiens, clone IMAGE:4477067, mRNA, partial cds. [BC012029] | -1.407377858 | 0.5104048 |
| NM_002318 | lysyl oxidase-like 2 (LOXL2), mRNA [NM_002318] | -1.404021531 | 0.57392764 |
| NM_016562 | toll-like receptor 7 (TLR7), mRNA [NM_016562] | -1.40115412 | 0.17046992 |
| NM_006546 | insulin-like growth factor 2 mRNA binding protein 1 (IGF2BP1), mRNA [NM_006546] | -1.394666563 | 0.56475128 |
| NM_198554 | thyroid adenoma associated (THADA), transcript variant 2, mRNA [NM_198554] | -1.39226465 | 0.31514416 |
| NM_032556 | interleukin 1 family, member 10 (theta) (IL1F10), transcript variant 1, mRNA [NM_032556] | -1.382745717 | 1.33531562 |
| THC2339389 | Unknown | -1.379298665 | 0.25613846 |
| NM_000945 | protein phosphatase 3 (formerly 2B), regulatory subunit B, 19kDa, alpha isoform (calcineurin B, type I) (PPP3R1), mRNA [NM_000945] | -1.376358993 | 0.05172827 |
| AK026768 | cDNA: FLJ23115 fis, clone LNG07933. [AK026768] | -1.375599911 | 0.83558755 |
| AK022150 | cDNA FLJ12088 fis, clone HEMBB1002545. [AK022150] | -1.357045355 | 0.82047279 |
| AI090937 | AI090937 qa52c01.s1 Soares_NhHMPu_S1 cDNA clone IMAGE:1690368 3', mRNA sequence [AI090937] | -1.335278308 | 0.34662415 |
| A_32_P220567 | Unknown | -1.328778681 | 0.32833454 |
| NM_002114 | immunodeficiency virus type I enhancer binding protein 1 (HIVEP1), mRNA [NM_002114] | -1.321052 | 0.94454826 |
| ENST00000368491 | cDNA clone IMAGE:5270591. [BC045657] | -1.30406827 | 0.67590382 |
| A_24_P635355 | Unknown | -1.303868983 | 0.23732376 |
| NM_199168 | chemokine (C-X-C motif) ligand 12 (stromal cell-derived factor 1) (CXCL12), transcript variant 1, mRNA [NM_199168] | -1.299775658 | 0.03768699 |
| NM_013989 | deiodinase, iodothyronine, type II (DIO2), transcript variant 1, mRNA [NM_013989] | -1.295800261 | 0.02158836 |
| NM_152495 | cornichon homolog 3 (Drosophila) (CNIH3), mRNA [NM_152495] | -1.294572151 | 0.2625958 |
| NM_013264 | DEAD (Asp-Glu-Ala-Asp) box polypeptide 25 (DDX25), mRNA [NM_013264] | -1.293379676 | 0.59284601 |
| NM_021738 | supervillin (SVIL), transcript variant 2, mRNA [NM_021738] | -1.289664849 | 0.78893792 |
| NM_213662 | signal transducer and activator of transcription 3 (acute-phase response factor) (STAT3), transcript variant 3, mRNA [NM_213662] | -1.28876115 | 0.70034092 |
| NM_006598 | solute carrier family 12 (potassium/chloride transporters), member 7 (SLC12A7), mRNA [NM_006598] | -1.286663364 | 0.20610459 |
| NM_006399 | basic leucine zipper transcription factor, ATF-like (BATF), mRNA [NM_006399] | -1.278970822 | 0.22083227 |
| XM_496707 | PREDICTED: hypothetical LOC441027 (LOC441027), mRNA [XM_496707] | -1.277733516 | 0.09620469 |
| A_32_P10589 | Unknown | -1.273700755 | 0.08421032 |
| NM_005110 | glutamine-fructose-6-phosphate transaminase 2 (GFPT2), mRNA [NM_005110] | -1.27121079 | 0.43141941 |

| | | | |
|---|---|---|---|
| CB528527 | CB528527 UI-H-FT2-bjd-k-24-0-UI.s1 NCI_CGAP_FT2 cDNA clone UI-H-FT2-bjd-k-24-0-UI 3', mRNA sequence [CB528527] | -1.269362872 | 1.22574472 |
| AK092577 | cDNA FLJ35258 fis, clone PROST2004146. [AK092577] | -1.269103671 | 0.47598454 |
| T40959 | T40959 ya15a04.s1 Stratagene liver (#937224) cDNA clone IMAGE:61518 3', mRNA sequence [T40959] | -1.266947626 | 0.23874456 |
| NM_015235 | cleavage stimulation factor, 3' pre-RNA, subunit 2, 64kDa, tau variant (CSTF2T), mRNA [NM_015235] | -1.258960748 | 0.96570969 |
| NM_002160 | tenascin C (hexabrachion) (TNC), mRNA [NM_002160] | -1.257260183 | 0.07762431 |
| NM_002318 | lysyl oxidase-like 2 (LOXL2), mRNA [NM_002318] | -1.25398643 | 0.37995274 |
| THC2382784 | B40201 artifact-warning sequence (translated ALU class B) - {Homo sapiens;} , partial (8%) [THC2382784] | -1.250617569 | 0.38311888 |
| ENST0000024291 6 | PREDICTED: similar to transcription elongation factor B (SIII), polypeptide 1 (LOC644540), mRNA [XM_927664] | -1.24712996 | 1.00697875 |
| CR619250 | full-length cDNA clone CS0DF034YK16 of Fetal brain of (human). [CR619250] | -1.244419255 | 0.10379091 |
| NM_005178 | B-cell CLL/lymphoma 3 (BCL3), mRNA [NM_005178] | -1.24157962 | 0.42562285 |
| BF718373 | BF718373 EST146 microdissected normal epidermis cDNA clone S81230.NIH-71-R 3', mRNA sequence [BF718373] | -1.241273962 | 0.89866221 |
| A_24_P660797 | Unknown | -1.235262835 | 1.03507728 |
| NM_001025366 | vascular endothelial growth factor (VEGF), transcript variant 1, mRNA [NM_001025366] | -1.230474571 | 0.91674756 |
| NM_000094 | collagen, type VII, alpha 1 (epidermolysis bullosa, dystrophic, dominant and recessive) (COL7A1), mRNA [NM_000094] | -1.228533399 | 0.34856648 |
| NM_001015880 | 3'-phosphoadenosine 5'-phosphosulfate synthase 2 (PAPSS2), transcript variant 2, mRNA [NM_001015880] | -1.22574106 | 1.06991319 |
| NM_002658 | plasminogen activator, urokinase (PLAU), mRNA [NM_002658] | -1.225527662 | 0.01282392 |
| NM_003998 | nuclear factor of kappa light polypeptide gene enhancer in B-cells 1 (p105) (NFKB1), mRNA [NM_003998] | -1.222991692 | 0.16999789 |
| NM_000722 | calcium channel, voltage-dependent, alpha 2/delta subunit 1 (CACNA2D1), mRNA [NM_000722] | -1.221027083 | 0.11990497 |
| AF467442 | Smith-Magenis syndrome chromosome region candidate 5 protein (SMCR5) mRNA, complete cds. [AF467442] | -1.220194559 | 0.36543148 |
| NM_199168 | chemokine (C-X-C motif) ligand 12 (stromal cell-derived factor 1) (CXCL12), transcript variant 1, mRNA [NM_199168] | -1.216971181 | 0.81718209 |
| X97261 | H.sapiens mRNA for metallothionein isoform 1R. [X97261] | -1.209725025 | 0.01800903 |
| NM_003324 | tubby like protein 3 (TULP3), mRNA [NM_003324] | -1.208178246 | 0.58778679 |
| NM_002658 | plasminogen activator, urokinase (PLAU), mRNA [NM_002658] | -1.198201432 | 0.75353523 |
| NM_001119 | adducin 1 (alpha) (ADD1), transcript variant 1, mRNA [NM_001119] | -1.195740832 | 0.98214164 |
| NM_002306 | lectin, galactoside-binding, soluble, 3 (galectin 3) (LGALS3), mRNA [NM_002306] | -1.194237826 | 0.23846884 |
| NM_018375 | solute carrier family 39 (zinc transporter), member 9 (SLC39A9), mRNA [NM_018375] | -1.193995414 | 0.47499995 |
| NM_003392 | wingless-type MMTV integration site family, member 5A (WNT5A), mRNA [NM_003392] | -1.192699649 | 0.93734991 |
| NM_134427 | regulator of G-protein signalling 3 (RGS3), transcript variant 4, mRNA [NM_134427] | -1.191937255 | 0.0700174 |
| BX538303 | mRNA; cDNA DKFZp686L04109 (from clone DKFZp686L04109) [BX538303] | -1.191137301 | 0.25204663 |
| NM_003998 | nuclear factor of kappa light polypeptide gene enhancer in B-cells 1 (p105) (NFKB1), mRNA [NM_003998] | -1.19025613 | 0.05568518 |
| NM_004972 | Janus kinase 2 (a protein tyrosine kinase) (JAK2), mRNA [NM_004972] | -1.189095496 | 0.09799819 |
| NM_000405 | GM2 ganglioside activator (GM2A), mRNA [NM_000405] | -1.182423084 | 0.0204777 |
| AK022339 | cDNA FLJ12277 fis, clone MAMMA1001711. [AK022339] | -1.182115594 | 0.983294 |
| NM_003264 | toll-like receptor 2 (TLR2), mRNA [NM_003264] | -1.181768608 | 0.40759276 |
| THC2375673 | Unknown | -1.180855476 | 0.96517731 |
| NM_012449 | six transmembrane epithelial antigen of the prostate 1 (STEAP1), mRNA [NM_012449] | -1.176807887 | 0.55082851 |
| NM_013322 | sorting nexin 10 (SNX10), mRNA [NM_013322] | -1.175914052 | 0.11109336 |

| | | | |
|---|---|---|---|
| ENST00000374279 | BP398053 pancreatic islet cDNA clone htp-09-88 3', mRNA sequence [BP398053] | -1.175507214 | 0.26718792 |
| THC2429731 | BC019890 MPZL1 protein {Homo sapiens;} , complete [THC2429731] | -1.175253826 | 0.77077203 |
| AK021543 | cDNA FLJ11481 fis, clone HEMBA1001803. [AK021543] | -1.170600469 | 0.83812706 |
| NM_018470 | chromosome 10 open reading frame 110 (C10orf110), mRNA [NM_018470] | -1.169067085 | 0.84575192 |
| NM_003185 | TAF4 RNA polymerase II, TATA box binding protein (TBP)-associated factor, 135kDa (TAF4), mRNA [NM_003185] | -1.168314769 | 0.74809135 |
| DB362335 | DB362335 NT2RM4 cDNA clone NT2RM4000005 3', mRNA sequence [DB362335] | -1.165792338 | 0.39249558 |
| NM_020666 | CDC-like kinase 4 (CLK4), mRNA [NM_020666] | -1.161056055 | 0.75400697 |
| NM_031313 | alkaline phosphatase, placental-like 2 (ALPPL2), mRNA [NM_031313] | -1.160919843 | 0.86883818 |
| NM_173797 | PAP associated domain containing 4 (PAPD4), mRNA [NM_173797] | -1.160903685 | 1.02878385 |
| NM_004972 | Janus kinase 2 (a protein tyrosine kinase) (JAK2), mRNA [NM_004972] | -1.157702736 | 0.69780486 |
| THC2433066 | Unknown | -1.157056668 | 0.04637206 |
| THC2370450 | AA805504 oc12e06.s1 NCI_CGAP_GCB1 cDNA clone IMAGE:1340674 3' similar to contains Alu repetitive element;, mRNA sequence [AA805504] | -1.154102121 | 0.55197055 |
| NM_001955 | endothelin 1 (EDN1), mRNA [NM_001955] | -1.153532398 | 0.2501415 |
| NM_006834 | RAB32, member RAS oncogene family (RAB32), mRNA [NM_006834] | -1.153455013 | 0.41516028 |
| NM_001025300 | RAB12, member RAS oncogene family (RAB12), mRNA [NM_001025300] | -1.151845174 | 0.91392253 |
| NM_139164 | START domain containing 4, sterol regulated (STARD4), mRNA [NM_139164] | -1.145776215 | 1.01409449 |
| NM_024830 | acyltransferase like 2 (AYTL2), mRNA [NM_024830] | -1.143845311 | 0.95842937 |
| NM_003311 | pleckstrin homology-like domain, family A, member 2 (PHLDA2), mRNA [NM_003311] | -1.141428434 | 0.34407064 |
| NM_004688 | N-myc (and STAT) interactor (NMI), mRNA [NM_004688] | -1.138883816 | 0.87108804 |
| NM_000480 | adenosine monophosphate deaminase (isoform E) (AMPD3), transcript variant 1, mRNA [NM_000480] | -1.134536841 | 0.30703992 |
| AA563626 | AA563626 ng47f01.s1 NCI_CGAP_Co3 cDNA clone IMAGE:937945 3', mRNA sequence [AA563626] | -1.133360531 | 0.04890723 |
| BC039117 | ovostatin 2, mRNA (cDNA clone IMAGE:4827636). [BC039117] | -1.129985975 | 0.72889556 |
| NM_003782 | UDP-Gal:betaGlcNAc beta 1,3-galactosyltransferase, polypeptide 4 (B3GALT4), mRNA [NM_003782] | -1.129871921 | 0.59873492 |
| NM_020987 | ankyrin 3, node of Ranvier (ankyrin G) (ANK3), transcript variant 1, mRNA [NM_020987] | -1.124664375 | 0.06753529 |
| NM_015704 | DNA segment, Chr 15, Wayne State University 75, expressed (D15Wsu75e), mRNA [NM_015704] | -1.124232293 | 0.912724 |
| NM_003821 | receptor-interacting serine-threonine kinase 2 (RIPK2), mRNA [NM_003821] | -1.119458598 | 0.28560772 |
| NM_005261 | GTP binding protein overexpressed in skeletal muscle (GEM), transcript variant 1, mRNA [NM_005261] | -1.119148492 | 0.06192135 |
| NM_024809 | chromosome 12 open reading frame 38 (C12orf38), mRNA [NM_024809] | -1.117126783 | 0.79546496 |
| BC107568 | cDNA clone IMAGE:3683736. [BC107568] | -1.115546144 | 0.0355863 |
| NM_002658 | plasminogen activator, urokinase (PLAU), mRNA [NM_002658] | -1.112528846 | 0.10906753 |
| AK021694 | cDNA FLJ11632 fis, clone HEMBA1004272. [AK021694] | -1.111612497 | 0.22700048 |
| NM_030665 | retinoic acid induced 1 (RAI1), mRNA [NM_030665] | -1.106969596 | 0.57467804 |
| NM_018148 | lines homolog 1 (Drosophila) (LINS1), transcript variant 1, mRNA [NM_018148] | -1.105465143 | 0.80362778 |
| D86974 | mRNA for KIAA0220 gene, partial cds. [D86974] | -1.104645232 | 0.95085811 |
| NM_006778 | tripartite motif-containing 10 (TRIM10), transcript variant 1, mRNA [NM_006778] | -1.104037185 | 0.04326749 |
| NM_032626 | retinoblastoma binding protein 6 (RBBP6), transcript variant 3, mRNA [NM_032626] | -1.102834979 | 0.3583237 |
| THC2405366 | Unknown | -1.097136624 | 0.02528054 |
| NM_002133 | heme oxygenase (decycling) 1 (HMOX1), mRNA [NM_002133] | -1.0949097 | 0.33063691 |
| AK092728 | cDNA FLJ35409 fis, clone SKNSH2009435. [AK092728] | -1.093857149 | 0.95732848 |
| THC2404028 | Unknown | -1.092416251 | 0.69101856 |
| NM_003613 | cartilage intermediate layer protein, nucleotide pyrophosphohydrolase (CILP), mRNA [NM_003613] | -1.092237077 | 0.72646359 |
| NM_001955 | endothelin 1 (EDN1), mRNA [NM_001955] | -1.091167375 | 0.20769655 |

| | | | |
|---|---|---|---|
| NM_005270 | GLI-Kruppel family member GLI2 (GLI2), mRNA [NM_005270] | -1.090588692 | 0.42184351 |
| NM_018896 | calcium channel, voltage-dependent, alpha 1G subunit (CACNA1G), transcript variant 1, mRNA [NM_018896] | -1.089510652 | 0.13568459 |
| NM_201525 | G protein-coupled receptor 56 (GPR56), transcript variant 3, mRNA [NM_201525] | -1.088768512 | 0.3973075 |
| CR610211 | full-length cDNA clone CS0DD002YI15 of Neuroblastoma Cot 50-normalized of (human). [CR610211] | -1.088645344 | 0.51564878 |
| ENST00000222543 | cDNA FLJ26323 fis, clone HRT00813, highly similar to Tissue factor pathway inhibitor 2 precursor (TFPI-2). [AK129833] | -1.08711698 | 0.42349246 |
| NM_198920 | chromosome 6 open reading frame 157 (C6orf157), mRNA [NM_198920] | -1.082604937 | 0.54917802 |
| NM_052864 | TRAF-interacting protein with a forkhead-associated domain (TIFA), mRNA [NM_052864] | -1.080296746 | 0.0224202 |
| NM_002658 | plasminogen activator, urokinase (PLAU), mRNA [NM_002658] | -1.079788996 | 0.04886532 |
| NM_153006 | N-acetylglutamate synthase (NAGS), mRNA [NM_153006] | -1.079445039 | 0.71230592 |
| NM_000610 | CD44 molecule (Indian blood group) (CD44), transcript variant 1, mRNA [NM_000610] | -1.077803572 | 0.19881302 |
| NM_004994 | matrix metallopeptidase 9 (gelatinase B, 92kDa gelatinase, 92kDa type IV collagenase) (MMP9), mRNA [NM_004994] | -1.076115077 | 0.4751375 |
| NM_002318 | lysyl oxidase-like 2 (LOXL2), mRNA [NM_002318] | -1.075247155 | 0.03550921 |
| NM_005527 | heat shock 70kDa protein 1-like (HSPA1L), mRNA [NM_005527] | -1.073093829 | 0.81429782 |
| AK021798 | cDNA FLJ11736 fis, clone HEMBA1005468. [AK021798] | -1.072509433 | 0.8458006 |
| NM_022126 | phospholysine phosphohistidine inorganic pyrophosphate phosphatase (LHPP), mRNA [NM_022126] | -1.070759753 | 0.3355578 |
| NM_003998 | nuclear factor of kappa light polypeptide gene enhancer in B-cells 1 (p105) (NFKB1), mRNA [NM_003998] | -1.061545381 | 0.13503147 |
| NM_199168 | chemokine (C-X-C motif) ligand 12 (stromal cell-derived factor 1) (CXCL12), transcript variant 1, mRNA [NM_199168] | -1.057588891 | 0.6814089 |
| NM_000610 | CD44 molecule (Indian blood group) (CD44), transcript variant 1, mRNA [NM_000610] | -1.057355971 | 0.3757537 |
| NM_020951 | zinc finger protein 529 (ZNF529), mRNA [NM_020951] | -1.055658941 | 0.24338581 |
| NM_001406 | ephrin-B3 (EFNB3), mRNA [NM_001406] | -1.052261012 | 0.81892736 |
| A_32_P94521 | Unknown | -1.048634699 | 0.67573909 |
| A_32_P208076 | Unknown | -1.039707533 | 0.0858333 |
| NM_002658 | plasminogen activator, urokinase (PLAU), mRNA [NM_002658] | -1.038377483 | 0.5452626 |
| NM_014266 | hematopoietic cell signal transducer (HCST), transcript variant 1, mRNA [NM_014266] | -1.032965915 | 0.12529125 |
| NM_024891 | hypothetical protein FLJ11783 (FLJ11783), mRNA [NM_024891] | -1.029109065 | 0.61318511 |
| CR602075 | full-length cDNA clone CS0DD009YF23 of Neuroblastoma Cot 50-normalized of (human). [CR602075] | -1.028633595 | 0.29550018 |
| NM_004079 | cathepsin S (CTSS), mRNA [NM_004079] | -1.026814058 | 0.21452063 |
| NM_207014 | WD repeat domain 78 (WDR78), transcript variant 2, mRNA [NM_207014] | -1.0267786 | 0.58245874 |
| XM_934270 | PREDICTED: hypothetical protein LOC647065 (LOC647065), mRNA [XM_934270] | -1.025553222 | 0.14384212 |
| NM_005604 | POU domain, class 3, transcription factor 2 (POU3F2), mRNA [NM_005604] | -1.024310365 | 0.05146969 |
| NM_020830 | WD repeat and FYVE domain containing 1 (WDFY1), mRNA [NM_020830] | -1.023312807 | 0.58033292 |
| NM_003998 | nuclear factor of kappa light polypeptide gene enhancer in B-cells 1 (p105) (NFKB1), mRNA [NM_003998] | -1.022014294 | 0.04696104 |
| ENST00000356170 | Unknown | -1.020331637 | 0.06931376 |
| THC2371963 | AIP1_(Q86UL8) Atrophin-1 interacting protein 1 (Atrophin-1 interacting protein A) (Membrane associated guanylate kinase inverted-2) (MAGI-2), partial (3%) [THC2371963] | -1.01976115 | 0.42109133 |
| BC034222 | HRAS-like suppressor family, member 5, mRNA (cDNA clone MGC:39540 IMAGE:5268703), complete cds. [BC034222] | -1.018813482 | 0.15751439 |
| CR619653 | full-length cDNA clone CS0DC014YO10 of Neuroblastoma Cot 25-normalized of (human). [CR619653] | -1.0164395 | 0.19181915 |
| NM_139278 | leucine-rich repeat LGI family, member 3 (LGI3), mRNA [NM_139278] | -1.015444602 | 0.36478373 |
| NM_014279 | olfactomedin 1 (OLFM1), transcript variant 1, mRNA [NM_014279] | -1.010891331 | 0.64239952 |

| | | | |
|---|---|---|---|
| BE644757 | BE644757 7e39h04.x1 NCI_CGAP_Lu24 cDNA clone IMAGE:3284887 3' similar to gb:U04897 NUCLEAR RECEPTOR ROR-ALPHA-1 (HUMAN);, mRNA sequence [BE644757] | -1.010508725 | 0.62983097 |
| NM_004994 | matrix metallopeptidase 9 (gelatinase B, 92kDa gelatinase, 92kDa type IV collagenase) (MMP9), mRNA [NM_004994] | -1.010069628 | 0.25927244 |
| NM_002866 | RAB3A, member RAS oncogene family (RAB3A), mRNA [NM_002866] | -1.009696546 | 0.22722848 |
| NM_002970 | spermidine/spermine N1-acetyltransferase (SAT), mRNA [NM_002970] | -1.004900281 | 0.35994611 |
| AK130644 | cDNA FLJ27134 fis, clone SPL08315. [AK130644] | -1.004120181 | 0.74184772 |
| NM_016229 | cytochrome b5 reductase 2 (CYB5R2), mRNA [NM_016229] | -1.003727601 | 0.11363003 |
| NM_003998 | nuclear factor of kappa light polypeptide gene enhancer in B-cells 1 (p105) (NFKB1), mRNA [NM_003998] | -1.002240486 | 0.07766023 |
| NM_199168 | chemokine (C-X-C motif) ligand 12 (stromal cell-derived factor 1) (CXCL12), transcript variant 1, mRNA [NM_199168] | -1.000311653 | 0.1768913 |
| NM_003821 | receptor-interacting serine-threonine kinase 2 (RIPK2), mRNA [NM_003821] | -0.999806588 | 0.2820975 |
| ENST00000337682 | family with sequence similarity 60, member A, mRNA (cDNA clone MGC:88660 IMAGE:5924274), complete cds. [BC071966] | -0.999548387 | 0.80665878 |
| THC2284350 | Unknown | -0.998510515 | 0.80199767 |
| NM_000899 | KIT ligand (KITLG), transcript variant b, mRNA [NM_000899] | -0.996006233 | 0.23138579 |
| NM_002658 | plasminogen activator, urokinase (PLAU), mRNA [NM_002658] | -0.991271801 | 0.04556288 |
| NM_014689 | dedicator of cytokinesis 10 (DOCK10), mRNA [NM_014689] | -0.988866714 | 0.00582471 |
| NM_003998 | nuclear factor of kappa light polypeptide gene enhancer in B-cells 1 (p105) (NFKB1), mRNA [NM_003998] | -0.980698776 | 0.27526568 |
| THC2282618 | Unknown | -0.979080159 | 0.58142605 |
| THC2295157 | Unknown | -0.977751691 | 0.79304103 |
| BC036637 | cell division cycle 2-like 6 (CDK8-like), mRNA (cDNA clone IMAGE:5296862). [BC036637] | -0.977433037 | 0.08601979 |
| NM_002658 | plasminogen activator, urokinase (PLAU), mRNA [NM_002658] | -0.97678424 | 0.42358808 |
| A_24_P187298 | Unknown | -0.975068321 | 0.36024732 |
| NM_020932 | melanoma antigen family E, 1 (MAGEE1), mRNA [NM_020932] | -0.974151248 | 0.70617735 |
| NM_152595 | piggyBac transposable element derived 4 (PGBD4), mRNA [NM_152595] | -0.972654195 | 0.55357456 |
| NM_001030059 | phosphatidic acid phosphatase type 2 domain containing 1A (PPAPDC1A), mRNA [NM_001030059] | -0.971768872 | 0.75823659 |
| NM_012123 | mitochondrial translation optimization 1 homolog (S. cerevisiae) (MTO1), transcript variant 2, mRNA [NM_012123] | -0.971086786 | 0.40447522 |
| NM_003190 | TAP binding protein (tapasin) (TAPBP), transcript variant 1, mRNA [NM_003190] | -0.968013856 | 0.48334216 |
| NM_002133 | heme oxygenase (decycling) 1 (HMOX1), mRNA [NM_002133] | -0.966790236 | 0.33079685 |
| A_24_P843552 | Unknown | -0.965173499 | 0.64325828 |
| NM_020898 | calcium binding and coiled-coil domain 1 (CALCOCO1), mRNA [NM_020898] | -0.964102262 | 0.25817286 |
| NM_002658 | plasminogen activator, urokinase (PLAU), mRNA [NM_002658] | -0.961345852 | 0.16524621 |
| THC2379106 | Q93NK8 (Q93NK8) YsaW, partial (7%) [THC2379106] | -0.960157532 | 0.27035502 |
| NM_001955 | endothelin 1 (EDN1), mRNA [NM_001955] | -0.959679105 | 0.01702176 |
| NM_003998 | nuclear factor of kappa light polypeptide gene enhancer in B-cells 1 (p105) (NFKB1), mRNA [NM_003998] | -0.958892686 | 0.09292971 |
| NM_032880 | immunoglobin superfamily, member 21 (IGSF21), mRNA [NM_032880] | -0.956732107 | 0.44603443 |
| NM_003201 | transcription factor A, mitochondrial (TFAM), mRNA [NM_003201] | -0.956452074 | 0.6184859 |
| BF197320 | BF197320 hr78g02.x1 NCI_CGAP_Kid11 cDNA clone IMAGE:3134642 3', mRNA sequence [BF197320] | -0.955961067 | 0.31112336 |
| THC2376915 | AE017295 pyridoxal phosphate biosynthetic protein {Leptospira interrogans serovar Copenhageni str. Fiocruz L1-130;} , partial (9%) [THC2376915] | -0.954035751 | 0.685269 |
| NM_032569 | cytokine-like nuclear factor n-pac (N-PAC), mRNA [NM_032569] | -0.953400727 | 0.7623789 |
| NM_005223 | deoxyribonuclease I (DNASE1), mRNA [NM_005223] | -0.953242256 | 0.45005536 |
| THC2309960 | Q7ZX66 (Q7ZX66) RNPC7 protein (Fragment), partial (9%) [THC2309960] | -0.951303948 | 0.61609394 |

| | | | |
|---|---|---|---|
| NM_018984 | slingshot homolog 1 (Drosophila) (SSH1), mRNA [NM_018984] | -0.95121094 | 0.1197238 |
| THC2305108 | CATD_(P07339) Cathepsin D precursor , partial (19%) [THC2305108] | -0.950111264 | 0.31552814 |
| NM_213662 | signal transducer and activator of transcription 3 (acute-phase response factor) (STAT3), transcript variant 3, mRNA [NM_213662] | -0.947938575 | 0.44296089 |
| NM_001734 | complement component 1, s subcomponent (C1S), transcript variant 1, mRNA [NM_001734] | -0.947294094 | 0.44183269 |
| BC089454 | cDNA clone MGC:105145 IMAGE:30563285, complete cds. [BC089454] | -0.945961001 | 0.23152824 |
| NM_003998 | nuclear factor of kappa light polypeptide gene enhancer in B-cells 1 (p105) (NFKB1), mRNA [NM_003998] | -0.94495289 | 0.14643465 |
| NM_033204 | zinc finger protein 101 (ZNF101), mRNA [NM_033204] | -0.943527332 | 0.75854406 |
| BX538051 | mRNA; cDNA DKFZp686F09156 (from clone DKFZp686F09156). [BX538051] | -0.939107756 | 0.58691262 |
| ENST00000372045 | full-length cDNA clone CS0DI016YJ18 of Placenta Cot 25-normalized of (human). [CR623913] | -0.937785101 | 0.21744043 |
| NM_001779 | CD58 molecule (CD58), mRNA [NM_001779] | -0.937662259 | 0.29464041 |
| NM_006738 | A kinase (PRKA) anchor protein 13 (AKAP13), transcript variant 1, mRNA [NM_006738] | -0.932530224 | 0.5846553 |
| AK025431 | cDNA: FLJ21778 fis, clone HEP00201. [AK025431] | -0.932438926 | 0.16430038 |
| NM_004285 | hexose-6-phosphate dehydrogenase (glucose 1-dehydrogenase) (H6PD), mRNA [NM_004285] | -0.932327197 | 0.56959868 |
| NM_001029863 | chromosome 6 open reading frame 120 (C6orf120), mRNA [NM_001029863] | -0.932127105 | 0.01302029 |
| NM_016530 | RAB8B, member RAS oncogene family (RAB8B), mRNA [NM_016530] | -0.931140553 | 0.38854163 |
| NM_199168 | chemokine (C-X-C motif) ligand 12 (stromal cell-derived factor 1) (CXCL12), transcript variant 1, mRNA [NM_199168] | -0.9306067 | 0.55391178 |
| NM_004877 | glia maturation factor, gamma (GMFG), mRNA [NM_004877] | -0.928001057 | 0.55579788 |
| AK021933 | cDNA FLJ11871 fis, clone HEMBA1007052. [AK021933] | -0.927110934 | 0.01947909 |
| THC2317093 | Unknown | -0.9262258 | 0.41019346 |
| THC2249196 | CICL_(P51801) Chloride channel protein ClC-Kb (Chloride channel Kb) (ClC-K2), complete [THC2249196] | -0.923573973 | 0.24943597 |
| NM_002336 | low density lipoprotein receptor-related protein 6 (LRP6), mRNA [NM_002336] | -0.92188428 | 0.37031562 |
| NM_080626 | BRI3 binding protein (BRI3BP), mRNA [NM_080626] | -0.921453142 | 0.47182099 |
| NM_006122 | mannosidase, alpha, class 2A, member 2 (MAN2A2), mRNA [NM_006122] | -0.921373235 | 0.20895311 |
| NM_003177 | spleen tyrosine kinase (SYK), mRNA [NM_003177] | -0.920785286 | 0.59615034 |
| THC2453189 | -50 | -0.919918928 | 0.71199843 |
| NM_001039650 | zinc finger, MYM-type 5 (ZMYM5), transcript variant 1, mRNA [NM_001039650] | -0.917182469 | 0.57245523 |
| NM_002317 | lysyl oxidase (LOX), mRNA [NM_002317] | -0.91605407 | 0.2289468 |
| BC007366 | chromosome 9 open reading frame 70, mRNA (cDNA clone MGC:16153 IMAGE:3632580), complete cds. [BC007366] | -0.914492821 | 0.35843291 |
| NM_133631 | roundabout, axon guidance receptor, homolog 1 (Drosophila) (ROBO1), transcript variant 2, mRNA [NM_133631] | -0.912214446 | 0.52566927 |
| NM_145906 | RIO kinase 3 (yeast) (RIOK3), transcript variant 2, mRNA [NM_145906] | -0.911600173 | 0.69200919 |
| NM_004333 | v-raf murine sarcoma viral oncogene homolog B1 (BRAF), mRNA [NM_004333] | -0.910988509 | 0.59561094 |
| NM_015001 | spen homolog, transcriptional regulator (Drosophila) (SPEN), mRNA [NM_015001] | -0.910765187 | 0.56983383 |
| NM_018490 | leucine-rich repeat-containing G protein-coupled receptor 4 (LGR4), mRNA [NM_018490] | -0.908394828 | 0.07756525 |
| NM_021199 | sulfide quinone reductase-like (yeast) (SQRDL), mRNA [NM_021199] | -0.908021024 | 0.2342464 |
| NM_019000 | hypothetical protein FLJ20152 (FLJ20152), transcript variant 2, mRNA [NM_019000] | -0.906182178 | 0.69678248 |
| BC100972 | similar to Williams Beuren syndrome chromosome region 19, mRNA (cDNA clone IMAGE:40005221), complete cds. [BC100972] | -0.903245604 | 0.46900004 |
| AK126405 | cDNA FLJ44441 fis, clone UTERU2020242. [AK126405] | -0.90255237 | 0.49741911 |
| NM_182977 | nicotinamide nucleotide transhydrogenase (NNT), mRNA [NM_182977] | -0.901502461 | 0.22664826 |

| | | | |
|---|---|---|---|
| NM_005952 | metallothionein 1X (MT1X), mRNA [NM_005952] | -0.900647239 | 0.0140362 |
| NM_003998 | nuclear factor of kappa light polypeptide gene enhancer in B-cells 1 (p105) (NFKB1), mRNA [NM_003998] | -0.898956518 | 0.11117073 |
| NM_207387 | chromosome 17 open reading frame 76 (C17orf76), mRNA [NM_207387] | -0.897237299 | 0.14726267 |
| NM_024551 | adiponectin receptor 2 (ADIPOR2), mRNA [NM_024551] | -0.897224117 | 0.2233249 |
| NM_000610 | CD44 molecule (Indian blood group) (CD44), transcript variant 1, mRNA [NM_000610] | -0.895544765 | 0.55520724 |
| NM_002200 | interferon regulatory factor 5 (IRF5), transcript variant 1, mRNA [NM_002200] | -0.890692373 | 0.47590431 |
| AL713792 | mRNA; cDNA DKFZp667J1615 (from clone DKFZp667J1615). [AL713792] | -0.890033314 | 0.60974972 |
| NM_022093 | tenascin N (TNN), mRNA [NM_022093] | -0.889781708 | 0.27603051 |
| NM_014985 | centrosomal protein 152kDa (CEP152), mRNA [NM_014985] | -0.888613778 | 0.47540107 |
| NM_016955 | soluble liver antigen/liver pancreas antigen (SLA/LP), transcript variant 1, mRNA [NM_016955] | -0.888265304 | 0.58748126 |
| NM_172174 | interleukin 15 (IL15), transcript variant 1, mRNA [NM_172174] | -0.887115778 | 0.30268332 |
| NM_005951 | metallothionein 1H (MT1H), mRNA [NM_005951] | -0.881244709 | 0.14347903 |
| NM_022074 | family with sequence similarity 111, member A (FAM111A), transcript variant 1, mRNA [NM_022074] | -0.879832754 | 0.19144425 |
| NM_002223 | inositol 1,4,5-triphosphate receptor, type 2 (ITPR2), mRNA [NM_002223] | -0.878864475 | 0.33567711 |
| NM_002133 | heme oxygenase (decycling) 1 (HMOX1), mRNA [NM_002133] | -0.878072638 | 0.63275075 |
| NM_032932 | RAB11 family interacting protein 4 (class II) (RAB11FIP4), mRNA [NM_032932] | -0.875833179 | 0.46266839 |
| AA665072 | AA665072 nu76b01.s1 NCI_CGAP_Alv1 cDNA clone IMAGE:1216585, mRNA sequence [AA665072] | -0.874347999 | 0.47297254 |
| NM_000610 | CD44 molecule (Indian blood group) (CD44), transcript variant 1, mRNA [NM_000610] | -0.872529821 | 0.31177118 |
| NM_002317 | lysyl oxidase (LOX), mRNA [NM_002317] | -0.870745466 | 0.16116219 |
| A_24_P401051 | Unknown | -0.869762438 | 0.35666817 |
| AF011794 | cell cycle progression restoration 8 protein (CPR8) mRNA, complete cds. [AF011794] | -0.869272595 | 0.36611838 |
| NM_001050 | somatostatin receptor 2 (SSTR2), mRNA [NM_001050] | -0.867982769 | 0.132659 |
| NM_001955 | endothelin 1 (EDN1), mRNA [NM_001955] | -0.865249122 | 0.05121336 |
| NM_005076 | contactin 2 (axonal) (CNTN2), mRNA [NM_005076] | -0.861023311 | 0.50101259 |
| NM_003631 | poly (ADP-ribose) glycohydrolase (PARG), mRNA [NM_003631] | -0.859304053 | 0.43260391 |
| BC089454 | cDNA clone MGC:105145 IMAGE:30563285, complete cds. [BC089454] | -0.859047276 | 0.02580533 |
| A_24_P711050 | Unknown | -0.857434204 | 0.53928967 |
| NM_005103 | fasciculation and elongation protein zeta 1 (zygin I) (FEZ1), transcript variant 1, mRNA [NM_005103] | -0.855740008 | 0.06514126 |
| A_24_P392900 | Unknown | -0.852840885 | 0.3382392 |
| NM_032352 | breast cancer metastasis-suppressor 1-like (BRMS1L), mRNA [NM_032352] | -0.850629375 | 0.28031288 |
| BC060758 | PPAR binding protein, mRNA (cDNA clone IMAGE:4822636), complete cds. [BC060758] | -0.848479733 | 0.19529878 |
| NM_000610 | CD44 molecule (Indian blood group) (CD44), transcript variant 1, mRNA [NM_000610] | -0.847861276 | 0.01700588 |
| BC089454 | cDNA clone MGC:105145 IMAGE:30563285, complete cds. [BC089454] | -0.845344873 | 0.1366538 |
| NM_023037 | furry homolog (Drosophila) (FRY), mRNA [NM_023037] | -0.845120402 | 0.19168239 |
| NM_001955 | endothelin 1 (EDN1), mRNA [NM_001955] | -0.844127947 | 0.20388359 |
| NM_032023 | Ras association (RalGDS/AF-6) domain family 4 (RASSF4), transcript variant 1, mRNA [NM_032023] | -0.843739176 | 0.31040014 |
| NM_198833 | serpin peptidase inhibitor, clade B (ovalbumin), member 8 (SERPINB8), transcript variant 2, mRNA [NM_198833] | -0.841412231 | 0.54888398 |
| NM_006267 | RAN binding protein 2 (RANBP2), mRNA [NM_006267] | -0.841072297 | 0.08098888 |
| AL109695 | mRNA full length insert cDNA clone EUROIMAGE 39820. [AL109695] | -0.840906923 | 0.51701315 |
| NM_006178 | N-ethylmaleimide-sensitive factor (NSF), mRNA [NM_006178] | -0.838488428 | 0.27687338 |
| NM_000362 | TIMP metallopeptidase inhibitor 3 (Sorsby fundus dystrophy, pseudoinflammatory) (TIMP3), mRNA [NM_000362] | -0.837382051 | 0.43306601 |
| NM_006828 | activating signal cointegrator 1 complex subunit 3 (ASCC3), transcript variant 1, mRNA [NM_006828] | -0.834518071 | 0.07159602 |
| NM_031438 | nudix (nucleoside diphosphate linked moiety X)-type motif 12 (NUDT12), mRNA [NM_031438] | -0.833775991 | 0.15254993 |
| NM_002133 | heme oxygenase (decycling) 1 (HMOX1), mRNA [NM_002133] | -0.831916492 | 0.39812222 |

| | | | |
|---|---|---|---|
| NM_030794 | tudor domain containing 3 (TDRD3), mRNA [NM_030794] | -0.831402078 | 0.04163861 |
| NM_005947 | metallothionein 1B (functional) (MT1B), mRNA [NM_005947] | -0.827362402 | 0.2031679 |
| NM_199168 | chemokine (C-X-C motif) ligand 12 (stromal cell-derived factor 1) (CXCL12), transcript variant 1, mRNA [NM_199168] | -0.82526038 | 0.11396091 |
| A_24_P615462 | Unknown | -0.825256077 | 0.44809266 |
| NM_001138 | agouti related protein homolog (mouse) (AGRP), transcript variant 1, mRNA [NM_001138] | -0.824223824 | 0.46880276 |
| NM_004054 | complement component 3a receptor 1 (C3AR1), mRNA [NM_004054] | -0.823164053 | 0.0532866 |
| NM_015058 | KIAA0564 protein (KIAA0564), transcript variant 1, mRNA [NM_015058] | -0.822991507 | 0.11183234 |
| AB015282 | mRNA for MNB/DYRK protein kinase, partial cds, alternatively spliced transcript MNB31. [AB015282] | -0.822136381 | 0.56777145 |
| NM_145238 | zinc finger protein 31 (ZNF31), mRNA [NM_145238] | -0.81837877 | 0.5255804 |
| NM_016166 | protein inhibitor of activated STAT, 1 (PIAS1), mRNA [NM_016166] | -0.817494661 | 0.21667799 |
| NM_173462 | papilin, proteoglycan-like sulfated glycoprotein (PAPLN), mRNA [NM_173462] | -0.814742773 | 0.00022031 |
| NM_177559 | casein kinase 2, alpha 1 polypeptide (CSNK2A1), transcript variant 1, mRNA [NM_177559] | -0.811014891 | 0.35290832 |
| NM_014737 | Ras association (RalGDS/AF-6) domain family 2 (RASSF2), transcript variant 1, mRNA [NM_014737] | -0.809904847 | 0.02121832 |
| NM_000610 | CD44 molecule (Indian blood group) (CD44), transcript variant 1, mRNA [NM_000610] | -0.809654251 | 0.17521412 |
| NM_004380 | CREB binding protein (Rubinstein-Taybi syndrome) (CREBBP), mRNA [NM_004380] | -0.809545087 | 0.4368466 |
| THC2304761 | ALU5_(P39192) Alu subfamily SC sequence contamination warning entry, partial (7%) [THC2304761] | -0.808757884 | 0.20875381 |
| NM_153183 | nudix (nucleoside diphosphate linked moiety X)-type motif 10 (NUDT10), mRNA [NM_153183] | -0.80805968 | 0.01055151 |
| NM_005746 | pre-B-cell colony enhancing factor 1 (PBEF1), transcript variant 1, mRNA [NM_005746] | -0.801861393 | 0.29010625 |
| NM_002133 | heme oxygenase (decycling) 1 (HMOX1), mRNA [NM_002133] | -0.799871138 | 0.2470149 |
| CR617352 | full-length cDNA clone CS0DD007YO17 of Neuroblastoma Cot 50-normalized of (human). [CR617352] | -0.798341189 | 0.21823643 |
| THC2429167 | Unknown | -0.797417394 | 0.35019799 |
| NM_000915 | oxytocin, prepro- (neurophysin I) (OXT), mRNA [NM_000915] | -0.797399774 | 0.2486026 |
| CD743044 | CD743044 UI-H-FT1-bjx-e-03-0-UI.s1 NCI_CGAP_FT1 cDNA clone UI-H-FT1-bjx-e-03-0-UI 3', mRNA sequence [CD743044] | -0.796576155 | 0.02548059 |
| NM_002977 | sodium channel, voltage-gated, type IX, alpha (SCN9A), mRNA [NM_002977] | -0.794428869 | 0.33034421 |
| NM_024749 | vasohibin 2 (VASH2), mRNA [NM_024749] | -0.794305763 | 0.30520604 |
| A_24_P196019 | Unknown | -0.793107779 | 0.43228283 |
| NM_024963 | F-box and leucine-rich repeat protein 18 (FBXL18), mRNA [NM_024963] | -0.792105214 | 0.42927284 |
| NM_031215 | Cdk5 and Abl enzyme substrate 2 (CABLES2), mRNA [NM_031215] | -0.791000431 | 0.36747314 |
| NM_004973 | Jumonji, AT rich interactive domain 2 (JARID2), mRNA [NM_004973] | -0.788755959 | 0.2244007 |
| A_32_P153604 | Unknown | -0.788031269 | 0.42077481 |
| A_32_P167212 | Unknown | -0.787655495 | 0.06254897 |
| NM_018664 | Jun dimerization protein p21SNFT (SNFT), mRNA [NM_018664] | -0.78717484 | 0.02516222 |
| NM_004994 | matrix metallopeptidase 9 (gelatinase B, 92kDa gelatinase, 92kDa type IV collagenase) (MMP9), mRNA [NM_004994] | -0.787086501 | 0.16535059 |
| NM_002133 | heme oxygenase (decycling) 1 (HMOX1), mRNA [NM_002133] | -0.785516894 | 0.45601733 |
| NM_002928 | regulator of G-protein signalling 16 (RGS16), mRNA [NM_002928] | -0.784605872 | 0.07766158 |
| BC063666 | cAMP responsive element binding protein 3-like 2, mRNA (cDNA clone IMAGE:4185677), complete cds. [BC063666] | -0.783406825 | 0.02935973 |
| NM_175884 | hypothetical protein FLJ36031 (FLJ36031), mRNA [NM_175884] | -0.783289901 | 0.23212922 |
| NM_002006 | fibroblast growth factor 2 (basic) (FGF2), mRNA [NM_002006] | -0.782873978 | 0.14288792 |
| NM_005124 | nucleoporin 153kDa (NUP153), mRNA [NM_005124] | -0.78262751 | 0.23877736 |
| AF086329 | full length insert cDNA clone ZD54C08. [AF086329] | -0.782335331 | 0.24307803 |
| NM_022154 | solute carrier family 39 (zinc transporter), member 8 (SLC39A8), mRNA [NM_022154] | -0.780743211 | 0.08372034 |

| | | | |
|---|---|---|---|
| BC004343 | chromosome 21 open reading frame 122, mRNA (cDNA clone MGC:10960 IMAGE:3633193), complete cds. [BC004343] | -0.780530121 | 0.39274165 |
| A_24_P499215 | Unknown | -0.779984493 | 0.24561714 |
| NM_004636 | sema domain, immunoglobulin domain (Ig), short basic domain, secreted, (semaphorin) 3B (SEMA3B), transcript variant 1, mRNA [NM_004636] | -0.779660097 | 0.11927181 |
| ENST00000344441 | mRNA for KIAA0268 gene, partial cds. [D87742] | -0.777396166 | 0.14073231 |
| NM_001632 | alkaline phosphatase, placental (Regan isozyme) (ALPP), mRNA [NM_001632] | -0.777284466 | 0.32471989 |
| NM_005180 | B lymphoma Mo-MLV insertion region (mouse) (BMI1), mRNA [NM_005180] | -0.776546542 | 0.30295165 |
| NM_002658 | plasminogen activator, urokinase (PLAU), mRNA [NM_002658] | -0.774376662 | 0.37150619 |
| NM_001031702 | sema domain, seven thrombospondin repeats (type 1 and type 1-like), transmembrane domain (TM) and short cytoplasmic domain, (semaphorin) 5B (SEMA5B), transcript variant 1, mRNA [NM_001031702] | -0.77359302 | 0.08721084 |
| BC005913 | surfactant, pulmonary-associated protein C, mRNA (cDNA clone MGC:14509 IMAGE:4043169), complete cds. [BC005913] | -0.771640622 | 0.52515144 |
| NM_018948 | ERBB receptor feedback inhibitor 1 (ERRFI1), mRNA [NM_018948] | -0.771516405 | 0.23942614 |
| ENST00000344556 | Unknown | -0.769658919 | 0.13520742 |
| NM_015033 | formin binding protein 1 (FNBP1), mRNA [NM_015033] | -0.769254486 | 0.18876046 |
| NM_032441 | zinc finger, matrin type 1 (ZMAT1), transcript variant 3, mRNA [NM_032441] | -0.768705369 | 0.36246681 |
| NM_172229 | kringle containing transmembrane protein 2 (KREMEN2), transcript variant 4, mRNA [NM_172229] | -0.768412263 | 0.04208064 |
| NM_003719 | phosphodiesterase 8B (PDE8B), transcript variant 1, mRNA [NM_003719] | -0.766941189 | 0.30285851 |
| NM_000311 | prion protein (p27-30) (Creutzfeldt-Jakob disease, Gerstmann-Strausler-Scheinker syndrome, fatal familial insomnia) (PRNP), transcript variant 1, mRNA [NM_000311] | -0.766689421 | 0.18713841 |
| THC2379232 | Unknown | -0.764664298 | 0.40854691 |
| NM_000382 | aldehyde dehydrogenase 3 family, member A2 (ALDH3A2), transcript variant 2, mRNA [NM_000382] | -0.764533993 | 0.15889719 |
| NM_144705 | tektin 4 (TEKT4), mRNA [NM_144705] | -0.763797195 | 0.25317968 |
| NM_015990 | kelch-like 5 (Drosophila) (KLHL5), transcript variant 1, mRNA [NM_015990] | -0.763113994 | 0.01461568 |
| NM_002497 | NIMA (never in mitosis gene a)-related kinase 2 (NEK2), mRNA [NM_002497] | -0.762651405 | 0.43262325 |
| BF326020 | BF326020 QV1-AN0059-040800-295-e05 AN0059 cDNA, mRNA sequence [BF326020] | -0.762365406 | 0.48485611 |
| NM_006404 | protein C receptor, endothelial (EPCR) (PROCR), mRNA [NM_006404] | -0.761480151 | 0.39288596 |
| BC017382 | ubiquitin specific peptidase 53, mRNA (cDNA clone IMAGE:4082351), complete cds. [BC017382] | -0.760996082 | 0.41492534 |
| NM_014278 | heat shock 70kDa protein 4-like (HSPA4L), mRNA [NM_014278] | -0.760227991 | 0.26594335 |
| NM_144643 | hypothetical protein FLJ30655 (FLJ30655), mRNA [NM_144643] | -0.759185819 | 0.04331948 |
| BF378046 | BF378046 RC1-TN0151-270900-013-b06 TN0151 cDNA, mRNA sequence [BF378046] | -0.758007776 | 0.25415551 |
| BE671816 | BE671816 7a47d01.x1 NCI_CGAP_GC6 cDNA clone IMAGE:3221857 3' similar to gb:X12433 PROTEIN PHPS1-2 (HUMAN);, mRNA sequence [BE671816] | -0.756409323 | 0.3690263 |
| NM_144978 | hypothetical protein FLJ32745 (FLJ32745), mRNA [NM_144978] | -0.756016801 | 0.23066229 |
| NM_003861 | WD repeat domain 22 (WDR22), mRNA [NM_003861] | -0.755421865 | 0.34073527 |
| AK074256 | cDNA FLJ23676 fis, clone HEP08548, highly similar to mRNA for MOP-3. [AK074256] | -0.755393426 | 0.00740465 |
| NM_005950 | metallothionein 1G (MT1G), mRNA [NM_005950] | -0.754572791 | 0.24623507 |
| NM_005509 | Dmx-like 1 (DMXL1), mRNA [NM_005509] | -0.754001851 | 0.04099892 |
| NM_003382 | vasoactive intestinal peptide receptor 2 (VIPR2), mRNA [NM_003382] | -0.752094972 | 0.02721172 |
| NM_004560 | receptor tyrosine kinase-like orphan receptor 2 (ROR2), mRNA [NM_004560] | -0.747974767 | 0.02159638 |
| AF238487 | olfactory-like receptor PJCG2 (PJCG2) mRNA, partial cds. [AF238487] | -0.746342829 | 0.00821747 |
| AF271775 | DC49 mRNA, complete cds. [AF271775] | -0.746169958 | 0.30931526 |
| AK055439 | cDNA FLJ30877 fis, clone FEBRA2004443. [AK055439] | -0.746046053 | 0.25126922 |

| | | | |
|---|---|---|---|
| A_32_P118556 | Unknown | -0.744240149 | 0.3869196 |
| NM_031894 | ferritin, heavy polypeptide-like 17 (FTHL17), mRNA [NM_031894] | -0.743647358 | 0.37469849 |
| NM_004510 | SP110 nuclear body protein (SP110), transcript variant b, mRNA [NM_004510] | -0.743448621 | 0.32733364 |
| NM_024920 | DnaJ (Hsp40) homolog, subfamily B, member 14 (DNAJB14), transcript variant 2, mRNA [NM_024920] | -0.742391477 | 0.15556388 |
| AF147723 | lipopolysaccharide specific response-68 protein (LSR68) mRNA, complete cds. [AF147723] | -0.742315785 | 0.17208465 |
| NM_024570 | deleted in lymphocytic leukemia 8 (DLEU8), mRNA [NM_024570] | -0.741773186 | 0.13862662 |
| NM_001645 | apolipoprotein C-I (APOC1), mRNA [NM_001645] | -0.740334163 | 0.10417567 |
| NM_148894 | family with sequence similarity 44, member A (FAM44A), mRNA [NM_148894] | -0.739325994 | 0.46566527 |
| THC2439773 | Unknown | -0.738450437 | 0.11370278 |
| NM_152455 | zinc finger protein 690 (ZNF690), mRNA [NM_152455] | -0.737141346 | 0.28925965 |
| NM_000311 | prion protein (p27-30) (Creutzfeldt-Jakob disease, Gerstmann-Strausler-Scheinker syndrome, fatal familial insomnia) (PRNP), transcript variant 1, mRNA [NM_000311] | -0.736440191 | 0.34455351 |
| THC2337980 | Unknown | -0.735647913 | 0.41635265 |
| NM_001845 | collagen, type IV, alpha 1 (COL4A1), mRNA [NM_001845] | -0.734535374 | 0.21629213 |
| NM_175861 | transmembrane and tetratricopeptide repeat containing 1 (TMTC1), mRNA [NM_175861] | -0.734069338 | 0.05126128 |
| NM_007257 | paraneoplastic antigen MA2 (PNMA2), mRNA [NM_007257] | -0.733786966 | 0.14699262 |
| NM_000618 | insulin-like growth factor 1 (somatomedin C) (IGF1), mRNA [NM_000618] | -0.733376785 | 0.32320935 |
| AW268902 | AW268902 xv48h10.x1 Soares_NFL_T_GBC_S1 cDNA clone IMAGE:2816419 3', mRNA sequence [AW268902] | -0.733258112 | 0.16433191 |
| THC2416008 | U74612 forkhead box M1A {Homo sapiens;} , partial (3%) [THC2416008] | -0.732131821 | 0.1605639 |
| NM_031419 | nuclear factor of kappa light polypeptide gene enhancer in B-cells inhibitor, zeta (NFKBIZ), transcript variant 1, mRNA [NM_031419] | -0.732005374 | 0.13808776 |
| CR590573 | full-length cDNA clone CS0DI042YD07 of Placenta Cot 25-normalized of (human). [CR590573] | -0.7318293 | 0.40904749 |
| NM_016235 | G protein-coupled receptor, family C, group 5, member B (GPRC5B), mRNA [NM_016235] | -0.731165484 | 0.12067885 |
| THC2313065 | MNN4_YEAST (P36044) MNN4 protein, partial (3%) [THC2313065] | -0.730222429 | 0.39455141 |
| THC2440027 | P70872 (P70872) FliY protein (Fragment), partial (5%) [THC2440027] | -0.730145799 | 0.46128111 |
| NM_153689 | hypothetical protein FLJ38973 (FLJ38973), mRNA [NM_153689] | -0.729506454 | 0.03756404 |
| NM_030911 | cytidine and dCMP deaminase domain containing 1 (CDADC1), mRNA [NM_030911] | -0.729389271 | 0.39533817 |
| NM_173554 | chromosome 10 open reading frame 107 (C10orf107), mRNA [NM_173554] | -0.72877515 | 0.14663804 |
| NM_152906 | chromosome 22 open reading frame 25 (C22orf25), mRNA [NM_152906] | -0.72851033 | 0.36447199 |
| NM_002133 | heme oxygenase (decycling) 1 (HMOX1), mRNA [NM_002133] | -0.727568276 | 0.20590489 |
| NM_020943 | KIAA1604 protein (KIAA1604), mRNA [NM_020943] | -0.727241666 | 0.12472419 |
| AB016898 | HGC6.4 mRNA, complete cds. [AB016898] | -0.724104609 | 0.42879149 |
| ENST0000037137 2 | mRNA for calcium channel, voltage-dependent, L type, alpha 1B subunit variant protein. [AB209467] | -0.722311313 | 0.00412185 |
| NM_006323 | SEC24 related gene family, member B (S. cerevisiae) (SEC24B), mRNA [NM_006323] | -0.722029344 | 0.20288304 |
| A_32_P172215 | Unknown | -0.72188802 | 0.21536524 |
| NM_015534 | zinc finger, ZZ-type containing 3 (ZZZ3), mRNA [NM_015534] | -0.721812066 | 0.31277337 |
| NM_002874 | RAD23 homolog B (S. cerevisiae) (RAD23B), mRNA [NM_002874] | -0.721472415 | 0.23065022 |
| NM_002318 | lysyl oxidase-like 2 (LOXL2), mRNA [NM_002318] | -0.718410552 | 0.01582885 |
| NM_015446 | AT hook containing transcription factor 1 (AHCTF1), mRNA [NM_015446] | -0.717942529 | 0.25343256 |
| NM_181659 | nuclear receptor coactivator 3 (NCOA3), transcript variant 1, mRNA [NM_181659] | -0.717234407 | 0.08407441 |
| THC2270231 | PBEF_(P43490) Pre-B cell enhancing factor precursor, partial (20%) [THC2270231] | -0.715767999 | 0.09709362 |
| AK025816 | cDNA: FLJ22163 fis, clone HRC00430. [AK025816] | -0.715703393 | 0.11868267 |
| NM_012449 | six transmembrane epithelial antigen of the prostate 1 (STEAP1), mRNA [NM_012449] | -0.715179184 | 0.2514157 |
| BC024020 | transmembrane protein 49, mRNA (cDNA clone IMAGE:4295896), with apparent retained intron. [BC024020] | -0.714776331 | 0.09390399 |
| THC2458496 | ALU1_(P39188) Alu subfamily J sequence contamination warning entry, partial (17%) [THC2458496] | -0.714746888 | 0.02872497 |

| | | | |
|---|---|---|---|
| NM_170587 | regulator of G-protein signalling 20 (RGS20), transcript variant 1, mRNA [NM_170587] | -0.714213429 | 0.15809564 |
| NM_002318 | lysyl oxidase-like 2 (LOXL2), mRNA [NM_002318] | -0.713747989 | 0.12622382 |
| NM_023016 | ankyrin repeat domain 57 (ANKRD57), mRNA [NM_023016] | -0.711116547 | 0.10157348 |
| NM_000944 | protein phosphatase 3 (formerly 2B), catalytic subunit, alpha isoform (calcineurin A alpha) (PPP3CA), mRNA [NM_000944] | -0.710691261 | 0.07132828 |
| CA314936 | UI-CF-FN0-afi-d-17-0-UI.s1 UI-CF-FN0 cDNA clone UI-CF-FN0-afi-d-17-0-UI 3', mRNA sequence [CA314936] | -0.7095343 | 0.38371681 |
| NM_017748 | coiled-coil domain containing 49 (CCDC49), mRNA [NM_017748] | -0.707337594 | 0.07525629 |
| A_32_P85360 | Unknown | -0.706836383 | 0.22553675 |
| BC041926 | cDNA clone IMAGE:5300349. [BC041926] | -0.706707629 | 0.29092453 |
| NM_004337 | chromosome 8 open reading frame 1 (C8orf1), mRNA [NM_004337] | -0.706106329 | 0.21492555 |
| NM_003998 | nuclear factor of kappa light polypeptide gene enhancer in B-cells 1 (p105) (NFKB1), mRNA [NM_003998] | -0.705594926 | 0.18966472 |
| NM_025246 | transmembrane protein 22 (TMEM22), mRNA [NM_025246] | -0.704748822 | 0.2770499 |
| NM_018361 | 1-acylglycerol-3-phosphate O-acyltransferase 5 (lysophosphatidic acid acyltransferase, epsilon) (AGPAT5), mRNA [NM_018361] | -0.704206975 | 0.0115596 |
| A_24_P409361 | Unknown | -0.701923547 | 0.09311218 |
| NM_014021 | synovial sarcoma, X breakpoint 2 interacting protein (SSX2IP), mRNA [NM_014021] | -0.701684874 | 0.17227124 |
| NM_012449 | six transmembrane epithelial antigen of the prostate 1 (STEAP1), mRNA [NM_012449] | -0.701348751 | 0.02281787 |
| NM_025132 | WD repeat domain 19 (WDR19), mRNA [NM_025132] | -0.701275963 | 0.19639544 |
| N47124 | N47124 yy53b06.r1 Soares_multiple_sclerosis_2NbHMSP cDNA clone IMAGE:277235 5', mRNA sequence [N47124] | -0.70102858 | 0.24438358 |
| NM_015176 | F-box protein 28 (FBXO28), mRNA [NM_015176] | -0.699208125 | 0.24875587 |
| A_24_P233560 | Unknown | -0.698011104 | 0.39152632 |
| THC2364621 | AF014891 NADH dehydrogenase subunit 2 {Homo sapiens;} , partial (10%) [THC2364621] | -0.697042989 | 0.15090219 |
| NM_014701 | KIAA0256 gene product (KIAA0256), mRNA [NM_014701] | -0.696859705 | 0.21312615 |
| ENST0000037153 4 | cDNA: FLJ21403 fis, clone COL03735. [AK025056] | -0.695465359 | 0.30092582 |
| NM_024003 | L1 cell adhesion molecule (L1CAM), transcript variant 2, mRNA [NM_024003] | -0.694950507 | 0.29290966 |
| NM_004994 | matrix metallopeptidase 9 (gelatinase B, 92kDa gelatinase, 92kDa type IV collagenase) (MMP9), mRNA [NM_004994] | -0.694256256 | 0.01598552 |
| THC2434747 | 1P3K_A Chain A, Crystallographic Studies Of Nucleosome Core Particles Containing Histone 'sin' Mutants. {Xenopus laevis;} , partial (30%) [THC2434747] | -0.693747196 | 0.27659596 |
| BC035518 | Homo sapiens, clone IMAGE:4214654, mRNA. [BC035518] | -0.69308737 | 0.28531023 |
| A_24_P306994 | Unknown | -0.692791083 | 0.21249982 |
| NM_019556 | motile sperm domain containing 1 (MOSPD1), mRNA [NM_019556] | -0.69276133 | 0.2509917 |
| AK127132 | cDNA FLJ45189 fis, clone BRAWH3049068. [AK127132] | -0.692754665 | 0.02175708 |
| NM_001955 | endothelin 1 (EDN1), mRNA [NM_001955] | -0.69092097 | 0.18176927 |
| NM_080860 | testis specific A2 homolog (mouse) (TSGA2), mRNA [NM_080860] | -0.690249169 | 0.40614615 |
| NM_024900 | PHD finger protein 17 (PHF17), transcript variant S, mRNA [NM_024900] | -0.689834154 | 0.11226164 |
| NM_198793 | CD47 molecule (CD47), transcript variant 2, mRNA [NM_198793] | -0.689536588 | 0.14969421 |
| NM_017590 | zinc finger CCCH-type containing 7B (ZC3H7B), mRNA [NM_017590] | -0.687773733 | 0.03219775 |
| NM_016264 | zinc finger protein 44 (ZNF44), mRNA [NM_016264] | -0.687017368 | 0.22785666 |
| NM_021629 | guanine nucleotide binding protein (G protein), beta polypeptide 4 (GNB4), mRNA [NM_021629] | -0.686425613 | 0.20652933 |
| NM_199346 | profilin family, member 4 (PFN4), mRNA [NM_199346] | -0.685485134 | 0.17919629 |
| NM_006007 | zinc finger, AN1-type domain 5 (ZFAND5), mRNA [NM_006007] | -0.685295922 | 0.16167069 |
| A_24_P408981 | Unknown | -0.684541777 | 0.18676451 |

| | | | |
|---|---|---|---|
| AK128731 | cDNA FLJ46899 fis, clone UTERU3022588, highly similar to Cyclic-AMP-dependent transcription factor ATF-2. [AK128731] | -0.682253683 | 0.01026247 |
| NM_016125 | PTD016 protein (LOC51136), mRNA [NM_016125] | -0.681548363 | 0.0297125 |
| NM_012316 | karyopherin alpha 6 (importin alpha 7) (KPNA6), mRNA [NM_012316] | -0.681287236 | 0.33123484 |
| A_24_P521544 | Unknown | -0.680081931 | 0.00463443 |
| A_24_P204135 | Unknown | -0.679993498 | 0.17409999 |
| NM_052952 | disrupted in renal carcinoma 1 (DIRC1), mRNA [NM_052952] | -0.677301174 | 0.03890516 |
| NM_173822 | hypothetical protein MGC39518 (MGC39518), mRNA [NM_173822] | -0.676723749 | 0.3682643 |
| NM_002231 | CD82 molecule (CD82), transcript variant 1, mRNA [NM_002231] | -0.676407897 | 0.23355248 |
| NM_004275 | Trf (TATA binding protein-related factor)-proximal homolog (Drosophila) (TRFP), mRNA [NM_004275] | -0.675318766 | 0.25890505 |
| NM_172097 | cation channel, sperm associated 2 (CATSPER2), transcript variant 4, mRNA [NM_172097] | -0.674574094 | 0.28057354 |
| NM_018084 | KIAA1212 (KIAA1212), mRNA [NM_018084] | -0.672653936 | 0.18468523 |
| NM_002198 | interferon regulatory factor 1 (IRF1), mRNA [NM_002198] | -0.672438407 | 0.00248049 |
| NM_001012302 | transmembrane protein 16J (TMEM16J), mRNA [NM_001012302] | -0.671446666 | 0.38103114 |
| NM_014646 | lipin 2 (LPIN2), mRNA [NM_014646] | -0.669975232 | 0.01402359 |
| NM_005339 | huntingtin interacting protein 2 (HIP2), mRNA [NM_005339] | -0.66964941 | 0.25026693 |
| ENST00000341154 | cDNA FLJ10617 fis, clone NT2RP2005464. [AK001479] | -0.668912126 | 0.19684975 |
| NM_014795 | zinc finger homeobox 1b (ZFHX1B), mRNA [NM_014795] | -0.668583956 | 0.24190105 |
| THC2442442 | ALU5_(P39192) Alu subfamily SC sequence contamination warning entry, partial (10%) [THC2442442] | -0.668024948 | 0.27217621 |
| AK021751 | cDNA FLJ11689 fis, clone HEMBA1004977. [AK021751] | -0.667994032 | 0.00920731 |
| NM_175623 | RAB3A interacting protein (rabin3) (RAB3IP), transcript variant alpha 2, mRNA [NM_175623] | -0.667297249 | 0.14301983 |
| NM_032119 | G protein-coupled receptor 98 (GPR98), mRNA [NM_032119] | -0.666733148 | 0.05566915 |
| AK025142 | cDNA: FLJ21489 fis, clone COL05450. [AK025142] | -0.666614198 | 0.00741723 |
| ENST00000382921 | cDNA FLJ10955 fis, clone PLACE1000406, moderately similar to PTB-ASSOCIATED SPLICING FACTOR. [AK001817] | -0.66611467 | 0.22661861 |
| BC089454 | cDNA clone MGC:105145 IMAGE:30563285, complete cds. [BC089454] | -0.665354004 | 0.28707139 |
| NM_002318 | lysyl oxidase-like 2 (LOXL2), mRNA [NM_002318] | -0.664746269 | 0.08365602 |
| NM_021219 | junctional adhesion molecule 2 (JAM2), mRNA [NM_021219] | -0.661614905 | 0.15423731 |
| NM_004688 | N-myc (and STAT) interactor (NMI), mRNA [NM_004688] | -0.659276468 | 0.26882232 |
| NM_033305 | vacuolar protein sorting 13 homolog A (S. cerevisiae) (VPS13A), transcript variant A, mRNA [NM_033305] | -0.657288418 | 0.1483972 |
| NM_017768 | leucine rich repeat containing 40 (LRRC40), mRNA [NM_017768] | -0.657156691 | 0.18031577 |
| NM_181501 | integrin, alpha 1 (ITGA1), mRNA [NM_181501] | -0.65654258 | 0.06187781 |
| AK021777 | cDNA FLJ11715 fis, clone HEMBA1005223. [AK021777] | -0.655249449 | 0.34990454 |
| NM_018229 | chromosome 14 open reading frame 108 (C14orf108), mRNA [NM_018229] | -0.653834305 | 0.29589662 |
| NM_000923 | phosphodiesterase 4C, cAMP-specific (phosphodiesterase E1 dunce homolog, Drosophila) (PDE4C), mRNA [NM_000923] | -0.653388909 | 0.29813717 |
| NM_021205 | ras homolog gene family, member U (RHOU), mRNA [NM_021205] | -0.651457125 | 0.34159915 |
| NM_003947 | kalirin, RhoGEF kinase (KALRN), transcript variant 2, mRNA [NM_003947] | -0.64998703 | 0.04925796 |
| NM_013448 | bromodomain adjacent to zinc finger domain, 1A (BAZ1A), transcript variant 1, mRNA [NM_013448] | -0.64951419 | 0.23080427 |
| NM_000633 | B-cell CLL/lymphoma 2 (BCL2), nuclear gene encoding mitochondrial protein, transcript variant alpha, mRNA [NM_000633] | -0.648993686 | 0.03763315 |
| NM_005919 | MADS box transcription enhancer factor 2, polypeptide B (myocyte enhancer factor 2B) (MEF2B), mRNA [NM_005919] | -0.648916634 | 0.14294444 |
| NM_004289 | nuclear factor (erythroid-derived 2)-like 3 (NFE2L3), mRNA [NM_004289] | -0.648447199 | 0.10866814 |

| | | | |
|---|---|---|---|
| NM_007063 | TBC1 domain family, member 8 (with GRAM domain) (TBC1D8), mRNA [NM_007063] | -0.648400054 | 0.06312613 |
| THC2374512 | Unknown | -0.648266957 | 0.32317248 |
| NM_002692 | polymerase (DNA directed), epsilon 2 (p59 subunit) (POLE2), mRNA [NM_002692] | -0.647843364 | 0.2217286 |
| NM_177403 | RAB7B, member RAS oncogene family (RAB7B), mRNA [NM_177403] | -0.64649507 | 0.13821149 |
| NM_020231 | chromosome 3 open reading frame 9 (C3orf9), mRNA [NM_020231] | -0.646405607 | 0.0972439 |
| AK023629 | cDNA FLJ13567 fis, clone PLACE1008331. [AK023629] | -0.646322458 | 0.13011392 |
| NM_020319 | ankyrin repeat and MYND domain containing 2 (ANKMY2), mRNA [NM_020319] | -0.645801413 | 0.2754382 |
| NM_003376 | vascular endothelial growth factor (VEGF), transcript variant 2, mRNA [NM_003376] | -0.645463326 | 0.24875895 |
| BC107586 | methyltransferase like 2B, mRNA (cDNA clone MGC:104351 IMAGE:6494734), complete cds. [BC107586] | -0.645377188 | 0.19370549 |
| ENST0000038002<br>1 | mRNA for KIAA0372 gene, partial cds. [AB002370] | -0.645265596 | 0.20329745 |
| NM_033306 | caspase 4, apoptosis-related cysteine peptidase (CASP4), transcript variant gamma, mRNA [NM_033306] | -0.645204854 | 0.20095336 |
| NM_018362 | lin-7 homolog C (C. elegans) (LIN7C), mRNA [NM_018362] | -0.644031048 | 0.07667648 |
| THC2315330 | Unknown | -0.643173782 | 0.09860103 |
| NM_001719 | bone morphogenetic protein 7 (osteogenic protein 1) (BMP7), mRNA [NM_001719] | -0.642955588 | 0.21665392 |
| NM_017884 | PIN2-interacting protein 1 (PINX1), mRNA [NM_017884] | -0.642015548 | 0.28138412 |
| ENST0000037791<br>8 | cDNA FLJ10272 fis, clone HEMBB1001117. [AK001134] | -0.641068006 | 0.15348751 |
| NM_018555 | zinc finger protein 331 (ZNF331), mRNA [NM_018555] | -0.640146572 | 0.26747667 |
| NM_139266 | signal transducer and activator of transcription 1, 91kDa (STAT1), transcript variant beta, mRNA [NM_139266] | -0.639589162 | 0.05443967 |
| NM_018662 | disrupted in schizophrenia 1 (DISC1), transcript variant L, mRNA [NM_018662] | -0.637868598 | 0.23797484 |
| NM_002318 | lysyl oxidase-like 2 (LOXL2), mRNA [NM_002318] | -0.636983747 | 0.06234165 |
| NM_001039580 | microtubule-associated protein 9 (MAP9), mRNA [NM_001039580] | -0.636684742 | 0.32881902 |
| NM_003423 | zinc finger protein 43 (ZNF43), mRNA [NM_003423] | -0.636515867 | 0.12754797 |
| NM_006113 | vav 3 oncogene (VAV3), mRNA [NM_006113] | -0.635458886 | 0.32520603 |
| AK057596 | cDNA FLJ33034 fis, clone THYMU2000236. [AK057596] | -0.634874597 | 0.04813745 |
| AB051533 | mRNA for KIAA1746 protein, partial cds. [AB051533] | -0.634059185 | 0.31623588 |
| NM_006708 | glyoxalase I (GLO1), mRNA [NM_006708] | -0.632955873 | 0.105971 |
| AK093903 | cDNA FLJ36584 fis, clone TRACH2013450. [AK093903] | -0.632909301 | 0.04048123 |
| THC2312785 | Unknown | -0.631842558 | 0.00984308 |
| NM_015368 | pannexin 1 (PANX1), mRNA [NM_015368] | -0.629728015 | 0.02738586 |
| NM_004994 | matrix metallopeptidase 9 (gelatinase B, 92kDa gelatinase, 92kDa type IV collagenase) (MMP9), mRNA [NM_004994] | -0.629087688 | 0.1913672 |
| NM_015262 | family with sequence similarity 21, member C (FAM21C), mRNA [NM_015262] | -0.628499546 | 0.19827374 |
| NM_005746 | pre-B-cell colony enhancing factor 1 (PBEF1), transcript variant 1, mRNA [NM_005746] | -0.627404418 | 0.3182689 |
| NM_016084 | RAS, dexamethasone-induced 1 (RASD1), mRNA [NM_016084] | -0.626106707 | 0.30273459 |
| NM_033411 | RWD domain containing 2 (RWDD2), mRNA [NM_033411] | -0.624105286 | 0.23600788 |
| NM_006624 | zinc finger, MYND domain containing 11 (ZMYND11), transcript variant 1, mRNA [NM_006624] | -0.622649165 | 0.24090148 |
| NM_024089 | KDEL (Lys-Asp-Glu-Leu) containing 1 (KDELC1), mRNA [NM_024089] | -0.622019144 | 0.00435583 |
| AK091744 | cDNA FLJ34425 fis, clone HHDPC2008297. [AK091744] | -0.619655148 | 0.06064991 |
| NM_000641 | interleukin 11 (IL11), mRNA [NM_000641] | -0.618747356 | 0.2472179 |
| A_24_P289573 | Unknown | -0.616841379 | 0.18403478 |
| AF168717 | x 009 protein mRNA, complete cds. [AF168717] | -0.615892164 | 0.23948479 |
| THC2315024 | Unknown | -0.615852597 | 0.03534441 |

| | | | |
|---|---|---|---|
| NM_002502 | nuclear factor of kappa light polypeptide gene enhancer in B-cells 2 (p49/p100) (NFKB2), mRNA [NM_002502] | -0.614696358 | 0.1810312 |
| BG108194 | BG108194 602280063F1 NIH_MGC_86 cDNA clone IMAGE:4367799 5', mRNA sequence [BG108194] | -0.613226063 | 0.17772332 |
| NM_004994 | matrix metallopeptidase 9 (gelatinase B, 92kDa gelatinase, 92kDa type IV collagenase) (MMP9), mRNA [NM_004994] | -0.612349277 | 0.10642148 |
| NM_001733 | complement component 1, r subcomponent (C1R), mRNA [NM_001733] | -0.612231005 | 0.19921138 |
| NM_017785 | coiled-coil domain containing 99 (CCDC99), mRNA [NM_017785] | -0.611864498 | 0.26014625 |
| NM_015294 | tripartite motif-containing 37 (TRIM37), transcript variant 1, mRNA [NM_015294] | -0.611489177 | 0.16251684 |
| NM_001014765 | F-box protein 44 (FBXO44), transcript variant 4, mRNA [NM_001014765] | -0.611294781 | 0.16743399 |
| NM_003412 | Zic family member 1 (odd-paired homolog, Drosophila) (ZIC1), mRNA [NM_003412] | -0.610709913 | 0.09875065 |
| NM_032783 | carbonyl reductase 4 (CBR4), mRNA [NM_032783] | -0.610172132 | 0.13392262 |
| A_24_P204015 | Unknown | -0.610109282 | 0.09727146 |
| NM_002977 | sodium channel, voltage-gated, type IX, alpha (SCN9A), mRNA [NM_002977] | -0.608350052 | 0.14610651 |
| AA114874 | zl03b10.s1 Soares_pregnant_uterus_NbHPU cDNA clone IMAGE:491227 3' similar to SW:RL21_P46778 60S RIBOSOMAL PROTEIN L21. ;, mRNA sequence [AA114874] | -0.608027856 | 0.05271165 |
| THC2376725 | Unknown | -0.607960651 | 0.25696077 |
| THC2395899 | Unknown | -0.607644886 | 0.215318 |
| NM_145735 | Rho guanine nucleotide exchange factor (GEF) 7 (ARHGEF7), transcript variant 2, mRNA [NM_145735] | -0.607643737 | 0.0083286 |
| AK026687 | cDNA: FLJ23034 fis, clone LNG02018. [AK026687] | -0.606541188 | 0.26308399 |
| A_23_P135063 | Unknown | -0.604999285 | 0.00244101 |
| NM_177528 | sulfotransferase family, cytosolic, 1A, phenol-preferring, member 2 (SULT1A2), transcript variant 2, mRNA [NM_177528] | -0.604714182 | 0.27166556 |
| NM_017686 | ganglioside induced differentiation associated protein 2 (GDAP2), mRNA [NM_017686] | -0.604441266 | 0.26604252 |
| A_32_P12282 | Unknown | -0.603578561 | 0.20414591 |
| NM_005951 | metallothionein 1H (MT1H), mRNA [NM_005951] | -0.603513297 | 0.12792045 |
| NM_015681 | B9 protein (EPPB9), mRNA [NM_015681] | -0.602931647 | 0.26429275 |
| NM_015039 | nicotinamide nucleotide adenylyltransferase 2 (NMNAT2), transcript variant 1, mRNA [NM_015039] | -0.600383553 | 0.25840552 |
| NM_031483 | itchy homolog E3 ubiquitin protein ligase (mouse) (ITCH), mRNA [NM_031483] | -0.600158436 | 0.13644234 |
