# Supplementary material for: Microglia Are Mediators of Borrelia burgdorferi–Induced Apoptosis in SH-SY5Y Neuronal Cells
Source: PLoS Pathog. 2009 Nov 13;5(11):e1000659. doi: 10.1371/journal.ppat.1000659 (PMC2771360; doi:10.1371/journal.ppat.1000659)
Supplement: Table S4 — SY3D + B. burgdorferi + Microglia vs. SY3D alone (0.83 MB PDF) [file ppat.1000659.s004.pdf]

Table S4: SY3D + B. burgdorferi + Microglia vs. SY3D alone.

GeneName	Description	Average Normalized Log2 Fold Change	Standard Deviation
NM_080607	chromosome 20 open reading frame 102 (C20orf102), mRNA [NM_080607]	3.53328987	2.61665524
AW576335	AW576335 UI-HF-BN0-akl-g-11-0-UI.s1 NIH_MGC_50 cDNA clone IMAGE:3077588 3', mRNA sequence [AW576335]	3.45187108	2.01350923
NM_031449	hypothetical protein DKFZp761I2123 (DKFZp761I2123), transcript variant 1, mRNA [NM_031449]	2.69175323	0.29732616
NM_173480	zinc finger protein 57 (ZNF57), mRNA [NM_173480]	2.50943143	2.00143114
NM_005636	synovial sarcoma, X breakpoint 4 (SSX4), transcript variant 1, mRNA [NM_005636]	2.48223152	0.51401599
NM_017585	solute carrier family 2 (facilitated glucose transporter), member 6 (SLC2A6), mRNA [NM_017585]	2.46645278	0.07068799
NM_171998	RAB39B, member RAS oncogene family (RAB39B), mRNA [NM_171998]	2.43867305	1.09090197
AA599881	AA599881 ag32e07.s1 Human bone marrow stromal cells cDNA clone IMAGE:1091268 3' similar to gb:M21574 ALPHA PLATELET-DERIVED GROWTH FACTOR RECEPTOR PRECURSOR (HUMAN);, mRNA sequence [AA599881]	2.43637035	0.19363992
NM_002850	protein tyrosine phosphatase, receptor type, S (PTPRS), transcript variant 1, mRNA [NM_002850]	2.36660807	1.52452128
NM_007353	guanine nucleotide binding protein (G protein) alpha 12 (GNA12), mRNA [NM_007353]	2.25213529	2.00813475
BC035091	cDNA clone IMAGE:5261717. [BC035091]	2.24310462	1.80670541
NM_030981	RAB1B, member RAS oncogene family (RAB1B), mRNA [NM_030981]	2.18682337	0.03461176
NM_023004	reticulon 4 receptor (RTN4R), mRNA [NM_023004]	2.1594471	1.96919198
AL138396	AL138396 DKFZp762A194_s1 762 (synonym: hmel2) cDNA clone DKFZp762A194 3', mRNA sequence [AL138396]	2.12583283	1.89274901
NM_005163	v-akt murine thymoma viral oncogene homolog 1 (AKT1), transcript variant 1, mRNA [NM_005163]	2.12165928	1.69645312
NM_002133	heme oxygenase (decycling) 1 (HMOX1), mRNA [NM_002133]	2.10835739	0.35688172
NM_002133	heme oxygenase (decycling) 1 (HMOX1), mRNA [NM_002133]	2.10701157	1.1326083
NM_133462	tetratricopeptide repeat domain 14 (TTC14), mRNA [NM_133462]	2.07835379	1.85741556
NM_003074	SWI/SNF related, matrix associated, actin dependent regulator of chromatin, subfamily c, member 1 (SMARCC1), mRNA [NM_003074]	2.07305066	1.42854612
AF531436	CGI-301 protein mRNA, complete cds. [AF531436]	2.07108591	0.05726338
NM_006206	platelet-derived growth factor receptor, alpha polypeptide (PDGFRA), mRNA [NM_006206]	2.05702536	0.75322495
NM_001719	bone morphogenetic protein 7 (osteogenic protein 1) (BMP7), mRNA [NM_001719]	1.87469076	0.43812615
NM_000813	gamma-aminobutyric acid (GABA) A receptor, beta 2 (GABRB2), transcript variant 2, mRNA [NM_000813]	1.84266969	1.81666404
THC2272102	Q9H496 (Q9H496) IFRG15 protein, complete [THC2272102]	1.81518545	1.8125691
NM_002413	microsomal glutathione S-transferase 2 (MGST2), mRNA [NM_002413]	1.7791959	1.03803299
A_24_P853004	Unknown	1.77445502	0.25370081
NM_004192	acetylserotonin O-methyltransferase-like (ASMTL), mRNA [NM_004192]	1.75772213	0.37049207
BC003178	mutY homolog (E. coli), mRNA (cDNA clone MGC:4416 IMAGE:2958002), complete cds. [BC003178]	1.74442653	1.62043036
NM_002133	heme oxygenase (decycling) 1 (HMOX1), mRNA [NM_002133]	1.74401908	0.56371033
AF277624	KRAB zinc finger protein (KR19) mRNA, complete cds. [AF277624]	1.71271763	1.19030152
NM_00100663(	cholinergic receptor, muscarinic 2 (CHRM2), transcript variant 1, mRNA [NM_001006630]	1.70723352	0.84191833
ENST00000337(	family with sequence similarity 60, member A, mRNA (cDNA clone MGC:88660 IMAGE:5924274), complete cds. [BC071966]	1.70641251	0.80385059
NM_002133	heme oxygenase (decycling) 1 (HMOX1), mRNA [NM_002133]	1.69353293	0.0377169

NM_003202	transcription factor 7 (T-cell specific, HMG-box) (TCF7), transcript variant 1, mRNA [NM_003202]	1.66212346	0.43732731
NM_025252	Ras association (RalGDS/AF-6) and pleckstrin homology domains 1 (RAPH1), transcript variant 2, mRNA [NM_025252]	1.63041821	1.59826734
NM_002998	syndecan 2 (heparan sulfate proteoglycan 1, cell surface-associated, fibroglycan) (SDC2), mRNA [NM_002998]	1.62323984	0.1112644
NM_000740	cholinergic receptor, muscarinic 3 (CHRM3), mRNA [NM_000740]	1.61557249	0.13618242
NM_199180	kin of IRRE like 2 (Drosophila) (KIRREL2), transcript variant 3, mRNA [NM_199180]	1.60618422	0.07550883
NM_000240	monoamine oxidase A (MAOA), nuclear gene encoding mitochondrial protein, mRNA [NM_000240]	1.5902789	0.17789496
NM_00103988	similar to zinc finger protein 91 (FLJ44894), mRNA [NM_001039884]	1.58549643	0.81579295
NM_018229	chromosome 14 open reading frame 108 (C14orf108), mRNA [NM_018229]	1.55877708	1.54254585
NM_002133	heme oxygenase (decycling) 1 (HMOX1), mRNA [NM_002133]	1.54032478	0.34344272
NM_016263	fizzy/cell division cycle 20 related 1 (Drosophila) (FZR1), mRNA [NM_016263]	1.53794942	1.38304108
NM_022467	carbohydrate (N-acetylgalactosamine 4-0) sulfotransferase 8 (CHST8), mRNA [NM_022467]	1.53447275	1.06607523
NM_017853	thioredoxin-like 4B (TXNL4B), mRNA [NM_017853]	1.51571091	1.31695123
CA313037	CA313037 UI-CF-FN0-aex-g-14-0-UI.s1 UI-CF-FN0 cDNA clone UI-CF-FN0-aex-g-14-0-UI 3', mRNA sequence [CA313037]	1.50846806	0.04260756
NM_001444	fatty acid binding protein 5 (psoriasis-associated) (FABP5), mRNA [NM_001444]	1.49515754	0.1265616
NM_004929	calbindin 1, 28kDa (CALB1), mRNA [NM_004929]	1.47839083	0.38354833
NM_183422	TSC22 domain family, member 1 (TSC22D1), transcript variant 1, mRNA [NM_183422]	1.46991605	0.60941348
NM_005163	v-akt murine thymoma viral oncogene homolog 1 (AKT1), transcript variant 1, mRNA [NM_005163]	1.46841067	0.46926538
ENST0000366	cDNA FLJ38396 fis, clone FEBRA2007957. [AK095715]	1.46494645	0.26555633
AL137342	mRNA; cDNA DKFZp761G1111 (from clone DKFZp761G1111). [AL137342]	1.45996164	1.30753708
THC2382287	ALU5_HUMAN (P39192) Alu subfamily SC sequence contamination warning entry, partial (5%) [THC2382287]	1.4474357	1.33645708
NM_002606	phosphodiesterase 9A (PDE9A), transcript variant 1, mRNA [NM_002606]	1.44476333	1.31700558
NM_003298	nuclear receptor subfamily 2, group C, member 2 (NR2C2), mRNA [NM_003298]	1.44403592	0.79187202
NM_003948	cyclin-dependent kinase-like 2 (CDC2-related kinase) (CDKL2), mRNA [NM_003948]	1.44264641	1.38722191
NM_152490	beta-1,3-N-acetylgalactosaminyltransferase 2 (B3GALNT2), mRNA [NM_152490]	1.43220635	0.31340599
NM_015277	neural precursor cell expressed, developmentally down-regulated 4-like (NEDD4L), mRNA [NM_015277]	1.41119991	0.22968741
NM_006143	G protein-coupled receptor 19 (GPR19), mRNA [NM_006143]	1.40830808	0.84863316
NM_174976	zinc finger, DHHC-type containing 22 (ZDHHC22), mRNA [NM_174976]	1.40075805	0.3648917
NM_006928	silver homolog (mouse) (SILV), mRNA [NM_006928]	1.39546206	0.36229128
NM_020737	leucine rich repeat and fibronectin type III domain containing 2 (LRFN2), mRNA [NM_020737]	1.39360292	0.03388561
ENST0000366	Q7PKG0 (Q7PKG0) ENSANGP00000024462 (Fragment), partial (36%) [THC2375456]	1.3846592	0.09741823
NM_019058	DNA-damage-inducible transcript 4 (DDIT4), mRNA [NM_019058]	1.38377284	0.21135117
NM_00104028	PAP associated domain containing 5 (PAPD5), transcript variant 2, mRNA [NM_001040285]	1.37833432	1.22636124
NM_003953	myelin protein zero-like 1 (MPZL1), transcript variant 1, mRNA [NM_003953]	1.37710586	0.7517106
NM_006128	bone morphogenetic protein 1 (BMP1), transcript variant BMP1-2, mRNA [NM_006128]	1.3751551	1.09536863
NM_000363	troponin I type 3 (cardiac) (TNNI3), mRNA [NM_000363]	1.34652215	0.47415255
NM_015014	RNA binding motif protein 34 (RBM34), mRNA [NM_015014]	1.34248755	0.75262418
AB075828	mRNA for KIAA1948 protein. [AB075828]	1.3278018	1.25776858

NM_177925	H2A histone family, member J (H2AFJ), transcript variant 2, mRNA [NM_177925]	1.32430848	0.35251187
NM_002133	heme oxygenase (decycling) 1 (HMOX1), mRNA [NM_002133]	1.32000239	0.09533826
NM_003061	slit homolog 1 (Drosophila) (SLIT1), mRNA [NM_003061]	1.31908326	0.02560972
NM_015354	nucleoporin 188kDa (NUP188), mRNA [NM_015354]	1.31806797	0.73755362
AK094724	cDNA FLJ37405 fis, clone BRAMY2028269. [AK094724]	1.31571022	0.72012279
AI369133	AI369133 qy74e10.x1 NCI_CGAP_Brn25 cDNA clone IMAGE:2017770 3', mRNA sequence [AI369133]	1.31263947	1.09239609
NM_001928	complement factor D (adipsin) (CFD), mRNA [NM_001928]	1.29846892	1.01408252
NM_030643	apolipoprotein L, 4 (APOL4), transcript variant a, mRNA [NM_030643]	1.29550749	1.25991245
NM_014717	zinc finger protein 536 (ZNF536), mRNA [NM_014717]	1.28723303	0.60176155
NM_006158	neurofilament, light polypeptide 68kDa (NEFL), mRNA [NM_006158]	1.28621492	0.10045914
NM_000116	tafazzin (cardiomyopathy, dilated 3A (X-linked); endocardial fibroelastosis 2; Barth syndrome) (TAZ), transcript variant 1, mRNA [NM_000116]	1.28349715	1.1369506
XM_929747	PREDICTED: hypothetical protein LOC148203 (LOC148203), mRNA [XM_929747]	1.28196231	0.05033073
NM_033504	transmembrane protein 54 (TMEM54), mRNA [NM_033504]	1.28103367	0.3839253
NM_018896	calcium channel, voltage-dependent, alpha 1G subunit (CACNA1G), transcript variant 1, mRNA [NM_018896]	1.27213806	0.12804081
NM_003713	phosphatidic acid phosphatase type 2B (PPAP2B), transcript variant 1, mRNA [NM_003713]	1.27090792	0.03635068
A_24_P910169	Unknown	1.2706529	1.12094413
NM_001444	fatty acid binding protein 5 (psoriasis-associated) (FABP5), mRNA [NM_001444]	1.26270889	0.35716909
NM_014550	caspase recruitment domain family, member 10 (CARD10), mRNA [NM_014550]	1.26053605	0.66495165
NM_001155	annexin A6 (ANXA6), transcript variant 1, mRNA [NM_001155]	1.25990318	0.0175458
NM_032298	synaptotagmin III (SYT3), mRNA [NM_032298]	1.25695697	0.62598134
NR_002802	trophoblast-derived noncoding RNA (TncRNA) on chromosome 11 [NR_002802]	1.25405087	0.51564191
NM_014634	protein phosphatase 1F (PP2C domain containing) (PPM1F), mRNA [NM_014634]	1.24931387	0.78867504
BX090412	BX090412 Soares_testis_NHT cDNA clone IMAGp998J221862 ; IMAGE:757365, mRNA sequence [BX090412]	1.24778553	0.95238373
AK091547	cDNA FLJ34228 fis, clone FCBBF3025417. [AK091547]	1.24582563	1.05172135
NM_002227	Janus kinase 1 (a protein tyrosine kinase) (JAK1), mRNA [NM_002227]	1.23911258	0.89968921
NM_000430	platelet-activating factor acetylhydrolase, isoform Ib, alpha subunit 45kDa (PAFAH1B1), mRNA [NM_000430]	1.23470382	1.12476029
NM_015267	cut-like 2 (Drosophila) (CUTL2), mRNA [NM_015267]	1.22980308	0.42385109
NM_006000	tubulin, alpha 1 (testis specific) (TUBA1), mRNA [NM_006000]	1.22965839	0.06308184
NM_178812	metadherin (MTDH), mRNA [NM_178812]	1.22852028	1.07998485
NM_024572	UDP-N-acetyl-alpha-D-galactosamine:polypeptide N-acetylgalactosaminyltransferase 14 (GalNAc-T14) (GALNT14), mRNA [NM_024572]	1.2171964	0.46617767
NM_173092	potassium voltage-gated channel, subfamily H (eag-related), member 6 (KCNH6), transcript variant 2, mRNA [NM_173092]	1.21566338	0.48618032
NM_002192	inhibin, beta A (activin A, activin AB alpha polypeptide) (INHBA), mRNA [NM_002192]	1.21385905	0.40263842
NM_004162	RAB5A, member RAS oncogene family (RAB5A), mRNA [NM_004162]	1.20915029	1.05541737
NM_002133	heme oxygenase (decycling) 1 (HMOX1), mRNA [NM_002133]	1.20889836	0.43794808
NM_003507	frizzled homolog 7 (Drosophila) (FZD7), mRNA [NM_003507]	1.20818313	0.06605736
BC107798	troponin T type 1 (skeletal, slow), mRNA (cDNA clone MGC:104241 IMAGE:4247379), complete cds. [BC107798]	1.20491697	0.02763165
CX788817	HESC3_102_H07.g1_A036 Human embryonic stem cells cDNA clone IMAGE:7485544 5', mRNA sequence [CX788817]	1.20386747	0.03149161

NM_020451	selenoprotein N, 1 (SEPN1), transcript variant 1, mRNA [NM_020451]	1.20331417	0.97077178
NM_014235	ubiquitin-like 4A (UBL4A), mRNA [NM_014235]	1.2026812	0.41352844
NM_000865	5-hydroxytryptamine (serotonin) receptor 1E (HTR1E), mRNA [NM_000865]	1.19915068	0.33399287
NM_016307	paired related homeobox 2 (PRRX2), mRNA [NM_016307]	1.19539411	0.38462908
NM_005076	contactin 2 (axonal) (CNTN2), mRNA [NM_005076]	1.1949041	0.45971648
NM_000587	complement component 7 (C7), mRNA [NM_000587]	1.19286624	0.11525476
NM_021949	ATPase, Ca++ transporting, plasma membrane 3 (ATP2B3), transcript variant 1, mRNA [NM_021949]	1.19259891	0.12310867
NM_018013	hypothetical protein FLJ10159 (FLJ10159), mRNA [NM_018013]	1.19254997	0.15352521
NM_003073	SWI/SNF related, matrix associated, actin dependent regulator of chromatin, subfamily b, member 1 (SMARCB1), transcript variant 1, mRNA [NM_003073]	1.19071926	0.82695427
NM_004104	fatty acid synthase (FASN), mRNA [NM_004104]	1.18935686	0.44115572
NM_001206	Kruppel-like factor 9 (KLF9), mRNA [NM_001206]	1.18702954	0.01973224
NM_177995	protein tyrosine phosphatase domain containing 1 (PTPDC1), transcript variant 2, mRNA [NM_177995]	1.18645958	0.77720957
NM_183002	solute carrier family 8 (sodium-calcium exchanger), member 3 (SLC8A3), transcript variant c, mRNA [NM_183002]	1.18401678	0.61518902
ENST0000037l:	cDNA FLJ37465 fis, clone BRAWH2011823, highly similar to BONE MORPHOGENETIC PROTEIN 7 PRECURSOR. [AK094784]	1.18030992	0.09890096
NM_004207	solute carrier family 16 (monocarboxylic acid transporters), member 3 (SLC16A3), transcript variant 2, mRNA [NM_004207]	1.18016064	0.67837102
NM_000362	TIMP metallopeptidase inhibitor 3 (Sorsby fundus dystrophy, pseudoinflammatory) (TIMP3), mRNA [NM_000362]	1.17365726	0.81938041
NM_00102536(	vascular endothelial growth factor (VEGF), transcript variant 1, mRNA [NM_001025366]	1.16830415	0.78190321
NM_006157	NEL-like 1 (chicken) (NELL1), mRNA [NM_006157]	1.16566415	0.43264999
NM_005194	CCAAT/enhancer binding protein (C/EBP), beta (CEBPB), mRNA [NM_005194]	1.14681489	0.40471091
NM_031890	cat eye syndrome chromosome region, candidate 6 (CECR6), mRNA [NM_031890]	1.14440055	0.20216511
ENST0000034l!	hypothetical metallo-hydrolase/oxidoreductase structure-containing protein (C7orf18) mRNA, complete cds. [AY357337]	1.14113525	0.97413826
NM_003468	frizzled homolog 5 (Drosophila) (FZD5), mRNA [NM_003468]	1.13744726	0.21677005
A_23_P369758	Unknown	1.13371932	0.76615762
NM_005737	ADP-ribosylation factor-like 4C (ARL4C), mRNA [NM_005737]	1.13216196	0.15393667
NM_020692	UDP-N-acetyl-alpha-D-galactosamine:polypeptide N-acetylgalactosaminyltransferase-like 1 (GALNTL1), mRNA [NM_020692]	1.131393	0.26973793
NM_012147	double homeobox, 2 (DUX2), mRNA [NM_012147]	1.12909691	0.06878762
NM_032523	oxysterol binding protein-like 6 (OSBPL6), transcript variant 1, mRNA [NM_032523]	1.1263576	0.97893126
AA449494	AA449494 zx08h11.s1 Soares_total_fetus_Nb2HF8_9w cDNA clone IMAGE:785925 3' similar to gb:U15981 ADRENAL SPECIFIC 30 KD PROTEIN (HUMAN);, mRNA sequence [AA449494]	1.12031207	0.07389017
BC028842	cDNA clone IMAGE:4793458. [BC028842]	1.11981724	0.92489839
NM_018490	leucine-rich repeat-containing G protein-coupled receptor 4 (LGR4), mRNA [NM_018490]	1.11141262	0.29066181
NM_007308	synuclein, alpha (non A4 component of amyloid precursor) (SNCA), transcript variant NACP112, mRNA [NM_007308]	1.10669193	0.09200677
THC2442550	Q5VV53 (Q5VV53) Erythroblast membrane-associated protein, partial (12%) [THC2442550]	1.10638865	0.43733246
NM_004598	sparc/osteonectin, cwcv and kazal-like domains proteoglycan (testican) 1 (SPOCK1), mRNA [NM_004598]	1.10179603	0.22466425

NM_000318	peroxisomal membrane protein 3, 35kDa (Zellweger syndrome) (PXMP3), mRNA [NM_000318]	1.10168756	0.62090229
NM_00100578	SMT3 suppressor of mif two 3 homolog 1 (S. cerevisiae) (SUMO1), transcript variant 2, mRNA [NM_001005781]	1.09501855	0.07904206
NM_207309	UDP-N-acteylglucosamine pyrophosphorylase 1-like 1 (UAP1L1), mRNA [NM_207309]	1.09188522	0.22132656
NM_024786	zinc finger, DHHC-type containing 11 (ZDHHC11), mRNA [NM_024786]	1.09014273	0.11859868
NM_001410	multiple EGF-like-domains 8 (MEGF8), mRNA [NM_001410]	1.08622366	0.32616418
NM_001853	collagen, type IX, alpha 3 (COL9A3), mRNA [NM_001853]	1.07910472	0.29689764
NR_002146	olfactory receptor, family 7, subfamily E, member 24 (OR7E24) on chromosome 19 [NR_002146]	1.07655253	0.81146721
NM_000176	nuclear receptor subfamily 3, group C, member 1 (glucocorticoid receptor) (NR3C1), transcript variant 5, mRNA [NM_000176]	1.07564517	0.736033
NM_021115	seizure related 6 homolog (mouse)-like (SEZ6L), mRNA [NM_021115]	1.07196235	0.94058604
NM_000389	cyclin-dependent kinase inhibitor 1A (p21, Cip1) (CDKN1A), transcript variant 1, mRNA [NM_000389]	1.07166306	0.36927761
ENST0000036	cDNA FLJ33320 fis, clone BNGH42007798, highly similar to Rattus norvegicus ankyrin binding cell adhesion molecule neurofascin mRNA. [AK090639]	1.0704081	0.39439177
NM_003459	solute carrier family 30 (zinc transporter), member 3 (SLC30A3), mRNA [NM_003459]	1.06900064	0.13197856
NM_000723	calcium channel, voltage-dependent, beta 1 subunit (CACNB1), transcript variant 1, mRNA [NM_000723]	1.06558954	0.87344456
NM_007308	synuclein, alpha (non A4 component of amyloid precursor) (SNCA), transcript variant NACP112, mRNA [NM_007308]	1.06458742	0.2435571
NM_001753	caveolin 1, caveolae protein, 22kDa (CAV1), mRNA [NM_001753]	1.06308839	0.86127387
BM666433	BM666433 UI-E-CQ1-aev-g-02-0-UI.s1 UI-E-CQ1 cDNA clone UI-E-CQ1-aev-g-02-0-UI 3', mRNA sequence [BM666433]	1.05948213	0.91426261
NM_000775	cytochrome P450, family 2, subfamily J, polypeptide 2 (CYP2J2), mRNA [NM_000775]	1.05926139	0.38638852
NM_001856	collagen, type XVI, alpha 1 (COL16A1), mRNA [NM_001856]	1.05807886	0.70390619
ENST0000031	cDNA: FLJ22044 fis, clone HEP09141. [AK025697]	1.0557617	0.73550175
NM_006379	sema domain, immunoglobulin domain (Ig), short basic domain, secreted, (semaphorin) 3C (SEMA3C), mRNA [NM_006379]	1.05575168	0.40537163
BC020336	ribosomal protein S2, mRNA (cDNA clone IMAGE:3857902). [BC020336]	1.05381478	0.1771961
NM_032569	cytokine-like nuclear factor n-pac (N-PAC), mRNA [NM_032569]	1.04292665	0.06717337
NM_133180	EPS8-like 1 (EPS8L1), transcript variant 1, mRNA [NM_133180]	1.04171401	0.11404944
NM_172215	calcium/calmodulin-dependent protein kinase kinase 2, beta (CAMKK2), transcript variant 6, mRNA [NM_172215]	1.04132622	0.80641315
THC2382871	Unknown	1.04019043	0.73689915
AK125077	cDNA FLJ43087 fis, clone BRTHA3019105. [AK125077]	1.03991794	0.304718
NM_030877	catenin, beta like 1 (CTNNBL1), mRNA [NM_030877]	1.03180396	0.25423957
NM_000314	phosphatase and tensin homolog (mutated in multiple advanced cancers 1) (PTEN), mRNA [NM_000314]	1.02703823	0.57655137
NM_018660	zinc finger protein 395 (ZNF395), mRNA [NM_018660]	1.02686339	0.3773494
NM_025246	transmembrane protein 22 (TMEM22), mRNA [NM_025246]	1.02501001	0.61669554
THC2375512	Unknown	1.02117475	0.43218036
NM_201446	EGF-like-domain, multiple 7 (EGFL7), transcript variant 2, mRNA [NM_201446]	1.0177862	0.17956829
NM_014719	KIAA0738 gene product (KIAA0738), mRNA [NM_014719]	1.01639035	0.85750078
NM_053036	neuropeptide FF receptor 2 (NPFFR2), transcript variant 2, mRNA [NM_053036]	1.01279164	0.79854131

ENST0000037013	Unknown	1.01009985	0.76692589
NM_004576	protein phosphatase 2 (formerly 2A), regulatory subunit B (PR 52), beta isoform (PPP2R2B), transcript variant 1, mRNA [NM_004576]	1.0090276	0.4922087
NM_004650	patatin-like phospholipase domain containing 4 (PNPLA4), mRNA [NM_004650]	1.00727418	0.27866327
NM_057091	artemin (ARTN), transcript variant 2, mRNA [NM_057091]	1.00572476	0.74942842
NM_144718	coiled-coil domain containing 52 (CCDC52), mRNA [NM_144718]	1.0008086	0.44441137
NM_001496	GDNF family receptor alpha 3 (GFRA3), mRNA [NM_001496]	0.99974055	0.07953872
NM_002861	phosphate cytidylyltransferase 2, ethanolamine (PCYT2), mRNA [NM_002861]	0.99515172	0.13662692
NM_032239	La ribonucleoprotein domain family, member 2 (LARP2), transcript variant 3, mRNA [NM_032239]	0.99365608	0.82493801
NM_007308	synuclein, alpha (non A4 component of amyloid precursor) (SNCA), transcript variant NACP112, mRNA [NM_007308]	0.98997869	0.06174132
NM_000389	cyclin-dependent kinase inhibitor 1A (p21, Cip1) (CDKN1A), transcript variant 1, mRNA [NM_000389]	0.98679045	0.34364531
NM_000713	biliverdin reductase B (flavin reductase (NADPH)) (BLVRB), mRNA [NM_000713]	0.98676115	0.2971813
NM_006109	protein arginine methyltransferase 5 (PRMT5), transcript variant 1, mRNA [NM_006109]	0.98566872	0.63678227
ENST0000034119	cDNA clone IMAGE:4476024. [BC071630]	0.9855651	0.31194988
NM_015493	ankyrin repeat domain 25 (ANKRD25), mRNA [NM_015493]	0.97583234	0.27391711
NM_015009	PDZ domain containing RING finger 3 (PDZRN3), mRNA [NM_015009]	0.97475145	0.085725
AF085877	full length insert cDNA clone YP11C01. [AF085877]	0.97466653	0.48506747
NM_005530	isocitrate dehydrogenase 3 (NAD+) alpha (IDH3A), nuclear gene encoding mitochondrial protein, mRNA [NM_005530]	0.97061812	0.68231821
NM_015460	myosin VIIA and Rab interacting protein (MYRIP), mRNA [NM_015460]	0.96435496	0.08995913
BC043527	Homo sapiens, clone IMAGE:5167766, mRNA. [BC043527]	0.96385184	0.02988488
NM_020926	BCL6 co-repressor (BCOR), transcript variant 2, mRNA [NM_020926]	0.96348207	0.74335132
NM_00103372З	zinc finger protein 704 (ZNF704), mRNA [NM_001033723]	0.95794409	0.18721881
NM_022151	modulator of apoptosis 1 (MOAP1), mRNA [NM_022151]	0.95557403	0.67977181
NM_024296	coiled-coil domain containing 28B (CCDC28B), mRNA [NM_024296]	0.95537269	0.13146319
A_32_P200025	Unknown	0.95111597	0.41012544
NM_003028	Src homology 2 domain containing adaptor protein B (SHB), mRNA [NM_003028]	0.94888769	0.02906064
NM_014351	sulfotransferase family 4A, member 1 (SULT4A1), mRNA [NM_014351]	0.94487311	0.63469599
AI446524	tj05a07.x1 NCI_CGAP_Gas4 cDNA clone IMAGE:2140596 3', mRNA sequence [AI446524]	0.94059586	0.71236374
NM_007175	SPFH domain family, member 2 (SPFH2), transcript variant 1, mRNA [NM_007175]	0.93754434	0.12791453
NM_00102445	retrotransposon gag domain containing 4 (RGAG4), mRNA [NM_001024455]	0.93508466	0.16965358
NM_005103	fasciculation and elongation protein zeta 1 (zygin I) (FEZ1), transcript variant 1, mRNA [NM_005103]	0.93324254	0.01389271
NM_000465	BRCA1 associated RING domain 1 (BARD1), mRNA [NM_000465]	0.93233162	0.01849502
XM_930260	PREDICTED: similar to Achaete-scute homolog 3 (bHLH transcriptional regulator Sgn-1) (Mash-3) (LOC647219), mRNA [XM_930260]	0.92859079	0.10587707
NM_006389	hypoxia up-regulated 1 (HYOU1), mRNA [NM_006389]	0.92852572	0.35790043
NM_003812	ADAM metallopeptidase domain 23 (ADAM23), mRNA [NM_003812]	0.92338889	0.51917275
BG001037	BG001037 RC5-GN0132-131100-012-E05 GN0132 cDNA, mRNA sequence [BG001037]	0.92296226	0.23474018
NM_006648	WNK lysine deficient protein kinase 2 (WNK2), mRNA [NM_006648]	0.92284081	0.07143092
CR608347	full-length cDNA clone CS0DG002YJ10 of B cells (Ramos cell line) of (human). [CR608347]	0.92245392	0.03584903
ENST0000024422	cDNA FLJ37016 fis, clone BRACE2010632. [AK094335]	0.92174697	0.3580862
AK026826	cDNA: FLJ23173 fis, clone LNG10019. [AK026826]	0.91962894	0.68264488

NM_002133	heme oxygenase (decycling) 1 (HMOX1), mRNA [NM_002133]	0.91629883	0.14558466
AL133642	mRNA; cDNA DKFZp586G1721 (from clone DKFZp586G1721). [AL133642]	0.91354533	0.53965208
NM_006254	protein kinase C, delta (PRKCD), transcript variant 1, mRNA [NM_006254]	0.90909617	0.31011465
NM_024668	ankyrin repeat and KH domain containing 1 (ANKHD1), transcript variant 3, mRNA [NM_024668]	0.90852592	0.05851978
NM_000728	calcitonin-related polypeptide, beta (CALCB), mRNA [NM_000728]	0.90824617	0.64866603
NM_002270	transportin 1 (TNPO1), transcript variant 1, mRNA [NM_002270]	0.90756787	0.14230788
ENST00000367[illegible]	cDNA FLJ13613 fis, clone PLACE1010856. [AK023675]	0.90518298	0.0085733
NM_014969	WD repeat domain 47 (WDR47), mRNA [NM_014969]	0.90202432	0.45604995
NM_000532	propionyl Coenzyme A carboxylase, beta polypeptide (PCCB), mRNA [NM_000532]	0.90072161	0.07168552
NM_020990	creatine kinase, mitochondrial 1B (CKMT1B), nuclear gene encoding mitochondrial protein, mRNA [NM_020990]	0.89949103	0.0128541
NM_138328	rhomboid, veinlet-like 3 (Drosophila) (RHBDL3), mRNA [NM_138328]	0.89847515	0.10643467
CR589996	full-length cDNA clone CS0DF019YO17 of Fetal brain of (human). [CR589996]	0.89802533	0.2952107
NM_198963	DEAH (Asp-Glu-Ala-Asp/His) box polypeptide 57 (DHX57), mRNA [NM_198963]	0.89668414	0.17524095
AB051500	mRNA for KIAA1713 protein, partial cds. [AB051500]	0.89358192	0.61285177
NM_015931	chromosome 3 open reading frame 32 (C3orf32), mRNA [NM_015931]	0.89234325	0.27224977
NM_00100578[illegible]	SMT3 suppressor of mif two 3 homolog 1 (S. cerevisiae) (SUMO1), transcript variant 2, mRNA [NM_001005781]	0.89208898	0.50515391
NM_181671	phosphatidylinositol transfer protein, cytoplasmic 1 (PITPNC1), transcript variant 2, mRNA [NM_181671]	0.89168968	0.57556772
NM_032354	transmembrane protein 107 (TMEM107), transcript variant 1, mRNA [NM_032354]	0.89122461	0.34994335
NM_000389	cyclin-dependent kinase inhibitor 1A (p21, Cip1) (CDKN1A), transcript variant 1, mRNA [NM_000389]	0.88902995	0.32543313
NM_175085	phosphoribosylglycinamide formyltransferase, phosphoribosylglycinamide synthetase, phosphoribosylaminoimidazole synthetase (GART), transcript variant 2, mRNA [NM_175085]	0.8855682	0.00021307
NM_199346	profilin family, member 4 (PFN4), mRNA [NM_199346]	0.88552213	0.54247725
NM_000389	cyclin-dependent kinase inhibitor 1A (p21, Cip1) (CDKN1A), transcript variant 1, mRNA [NM_000389]	0.88010723	0.18124884
NM_006214	phytanoyl-CoA 2-hydroxylase (PHYH), transcript variant 1, mRNA [NM_006214]	0.87984897	0.38794896
BC039397	cDNA clone IMAGE:5299888. [BC039397]	0.87974673	0.0981098
NM_006158	neurofilament, light polypeptide 68kDa (NEFL), mRNA [NM_006158]	0.87768951	0.50669632
AF161342	HSPC079 mRNA, partial cds. [AF161342]	0.87764765	0.08972555
AB040944	mRNA for KIAA1511 protein, partial cds. [AB040944]	0.87629033	0.26144344
NM_014476	PDZ and LIM domain 3 (PDLIM3), mRNA [NM_014476]	0.87574881	0.02046563
NM_00101297[illegible]	NGFRAP1-like 1 (NGFRAP1L1), mRNA [NM_001012978]	0.87466638	0.47215425
BC021035	cDNA clone IMAGE:3834434, **** WARNING: chimeric clone ****. [BC021035]	0.87130669	0.32093679
NM_181690	v-akt murine thymoma viral oncogene homolog 3 (protein kinase B, gamma) (AKT3), transcript variant 2, mRNA [NM_181690]	0.86838038	0.63437548
NM_032793	major facilitator superfamily domain containing 2 (MFSD2), mRNA [NM_032793]	0.86836105	0.24841375
NM_058182	chromosome 21 open reading frame 51 (C21orf51), transcript variant 1, mRNA [NM_058182]	0.86792422	0.46759912
NM_178868	CKLF-like MARVEL transmembrane domain containing 8 (CMTM8), mRNA [NM_178868]	0.86645951	0.49083475
NM_014333	immunoglobulin superfamily, member 4 (IGSF4), mRNA [NM_014333]	0.86446555	0.03597725
NM_017754	chromosome 6 open reading frame 107 (C6orf107), mRNA [NM_017754]	0.86347235	0.18028155

NM_004716	proprotein convertase subtilisin/kexin type 7 (PCSK7), mRNA [NM_004716]	0.86119177	0.65176674
NM_133170	protein tyrosine phosphatase, receptor type, T (PTPRT), transcript variant 1, mRNA [NM_133170]	0.8592714	0.48762387
NM_015845	methyl-CpG binding domain protein 1 (MBD1), transcript variant 2, mRNA [NM_015845]	0.85739223	0.28816939
NM_017596	kinesin family member 21B (KIF21B), mRNA [NM_017596]	0.85574544	0.15754057
NM_018973	dolichyl-phosphate mannosyltransferase polypeptide 3 (DPM3), transcript variant 1, mRNA [NM_018973]	0.85520609	0.2083336
NM_016436	PHD finger protein 20 (PHF20), mRNA [NM_016436]	0.85209533	0.12922274
NM_003115	UDP-N-acteylglucosamine pyrophosphorylase 1 (UAP1), mRNA [NM_003115]	0.84904603	0.44779328
NM_198516	UDP-N-acetyl-alpha-D-galactosamine:polypeptide N-acetylgalactosaminyltransferase-like 4 (GALNTL4), mRNA [NM_198516]	0.84583463	0.19707475
NM_006334	olfactomedin 1 (OLFM1), transcript variant 2, mRNA [NM_006334]	0.84537894	0.05890899
NM_014458	kelch-like 20 (Drosophila) (KLHL20), mRNA [NM_014458]	0.84180546	0.2737076
AL137349	mRNA; cDNA DKFZp434A0225 (from clone DKFZp434A0225). [AL137349]	0.84147485	0.5737061
AK090969	cDNA FLJ33650 fis, clone BRAMY2024514, highly similar to Rattus norvegicus potassium channel (erg2) mRNA. [AK090969]	0.83906161	0.24813418
AF113013	PRO0806 mRNA, complete cds. [AF113013]	0.83894999	0.32993677
NM_032970	SEC22 vesicle trafficking protein homolog C (S. cerevisiae) (SEC22C), transcript variant 1, mRNA [NM_032970]	0.83678329	0.52970419
NM_173562	potassium channel tetramerisation domain containing 20 (KCTD20), mRNA [NM_173562]	0.83658647	0.47579878
NM_000687	S-adenosylhomocysteine hydrolase (AHCY), mRNA [NM_000687]	0.83497439	0.40574777
NM_001333	cathepsin L2 (CTSL2), mRNA [NM_001333]	0.83273251	0.05032297
NM_000389	cyclin-dependent kinase inhibitor 1A (p21, Cip1) (CDKN1A), transcript variant 1, mRNA [NM_000389]	0.83081125	0.12364397
NM_002395	malic enzyme 1, NADP(+)-dependent, cytosolic (ME1), mRNA [NM_002395]	0.82978936	0.1964679
NM_013313	yippee-like 1 (Drosophila) (YPEL1), mRNA [NM_013313]	0.82879909	0.07409438
NM_003633	ectodermal-neural cortex (with BTB-like domain) (ENC1), mRNA [NM_003633]	0.82798844	0.03150372
NR_002734	pituitary tumor-transforming 3 (PTTG3) on chromosome 8 [NR_002734]	0.82713903	0.34752139
NM_138765	BCL2-associated X protein (BAX), transcript variant sigma, mRNA [NM_138765]	0.82639443	0.05348637
BE893137	601437034F1 NIH_MGC_72 cDNA clone IMAGE:3922112 5', mRNA sequence [BE893137]	0.8262702	0.21860367
NM_032281	ELAV (embryonic lethal, abnormal vision, Drosophila)-like 3 (Hu antigen C) (ELAVL3), transcript variant 2, mRNA [NM_032281]	0.82577379	0.08822328
AA714039	AA714039 nx81g06.s1 NCI_CGAP_GCB1 cDNA clone IMAGE:1268698 3', mRNA sequence [AA714039]	0.82425227	0.46836954
NM_012189	calcium binding tyrosine-(Y)-phosphorylation regulated (fibrousheathin 2) (CABYR), transcript variant 1, mRNA [NM_012189]	0.82287232	0.39686314
NM_004192	acetylserotonin O-methyltransferase-like (ASMTL), mRNA [NM_004192]	0.82135149	0.50254109
ENST0000037	cDNA: FLJ22260 fis, clone HRC02953. [AK025913]	0.81881829	0.02906504
NM_024010	5-methyltetrahydrofolate-homocysteine methyltransferase reductase (MTRR), transcript variant 2, mRNA [NM_024010]	0.81854586	0.50989687
NM_005723	tetraspanin 5 (TSPAN5), mRNA [NM_005723]	0.81819323	0.11858519
NM_012295	calcineurin binding protein 1 (CABIN1), mRNA [NM_012295]	0.81772107	0.00596813
NM_024959	solute carrier family 24 (sodium/potassium/calcium exchanger), member 6 (SLC24A6), mRNA [NM_024959]	0.81601136	0.502014
NM_007219	ring finger protein 24 (RNF24), mRNA [NM_007219]	0.81562309	0.27125113
NM_021968	histone 1, H4j (HIST1H4J), mRNA [NM_021968]	0.81546817	0.17697337

A_24_P901836	Unknown	0.81544059	0.46870324
NM_001005781	SMT3 suppressor of mif two 3 homolog 1 (S. cerevisiae) (SUMO1), transcript variant 2, mRNA [NM_001005781]	0.81311623	0.05373114
NM_024624	structural maintenance of chromosomes 6 (SMC6), mRNA [NM_024624]	0.81215906	0.46154566
THC2371517	IRO548013 HMG20A {Homo sapiens;} , partial (35%) [THC2371517]	0.81214571	0.56545321
NM_005828	WD repeat domain 68 (WDR68), mRNA [NM_005828]	0.81192299	0.17680446
NM_003908	eukaryotic translation initiation factor 2, subunit 2 beta, 38kDa (EIF2S2), mRNA [NM_003908]	0.81013944	0.46757598
NM_198270	Nance-Horan syndrome (congenital cataracts and dental anomalies) (NHS), mRNA [NM_198270]	0.8093556	0.12281611
NM_005184	calmodulin 3 (phosphorylase kinase, delta) (CALM3), mRNA [NM_005184]	0.80658497	0.17224659
NM_000746	cholinergic receptor, nicotinic, alpha 7 (CHRNA7), mRNA [NM_000746]	0.80655098	0.01286884
THC2276324	Unknown	0.80550882	0.15063875
NM_017712	pyroglutamyl-peptidase I (PGPEP1), mRNA [NM_017712]	0.8052816	0.49732813
A_24_P229766	Unknown	0.80446734	0.02665701
BX360933	BX360933 BX360933 PLACENTA COT 25-NORMALIZED cDNA clone CS0DI077YB17 3-PRIME, mRNA sequence [BX360933]	0.80208676	0.04632542
NM_001237	cyclin A2 (CCNA2), mRNA [NM_001237]	0.80108111	0.14266586
NM_001123	adenosine kinase (ADK), transcript variant ADK-short, mRNA [NM_001123]	0.79861673	0.49738043
NM_145719	tigger transposable element derived 3 (TIGD3), mRNA [NM_145719]	0.79269731	0.46269428
NM_004457	acyl-CoA synthetase long-chain family member 3 (ACSL3), transcript variant 1, mRNA [NM_004457]	0.7925616	0.35309943
NM_153832	G protein-coupled receptor 161 (GPR161), transcript variant 2, mRNA [NM_153832]	0.78960812	0.22131381
NM_000389	cyclin-dependent kinase inhibitor 1A (p21, Cip1) (CDKN1A), transcript variant 1, mRNA [NM_000389]	0.78748271	0.01385898
A_23_P250072	Unknown	0.7864592	0.19219089
NM_033375	myosin IC (MYO1C), mRNA [NM_033375]	0.78570509	0.36493408
AB188494	mRNA, clone: TH049G03. [AB188494]	0.78551734	0.145151
NM_024786	zinc finger, DHHC-type containing 11 (ZDHHC11), mRNA [NM_024786]	0.78522763	0.27537763
NM_002697	POU domain, class 2, transcription factor 1 (POU2F1), mRNA [NM_002697]	0.78507673	0.12358729
NM_153269	chromosome 20 open reading frame 96 (C20orf96), mRNA [NM_153269]	0.78347749	0.1039264
NM_000389	cyclin-dependent kinase inhibitor 1A (p21, Cip1) (CDKN1A), transcript variant 1, mRNA [NM_000389]	0.78276049	0.14626592
NM_025246	transmembrane protein 22 (TMEM22), mRNA [NM_025246]	0.78123979	0.09062171
NM_024753	tetratricopeptide repeat domain 21B (TTC21B), mRNA [NM_024753]	0.77795578	0.51575761
AB046770	mRNA for KIAA1550 protein, partial cds. [AB046770]	0.7750668	0.32181933
NM_007308	synuclein, alpha (non A4 component of amyloid precursor) (SNCA), transcript variant NACP112, mRNA [NM_007308]	0.77423749	0.25249443
NM_006959	zinc finger protein 17 (ZNF17), mRNA [NM_006959]	0.7724264	0.31148413
NM_020920	chromodomain helicase DNA binding protein 8 (CHD8), mRNA [NM_020920]	0.77242409	0.02882522
AB040888	mRNA for KIAA1455 protein, partial cds. [AB040888]	0.77180278	0.25312185
NM_018124	ring finger and WD repeat domain 3 (RFWD3), mRNA [NM_018124]	0.77032107	0.13915552
NM_005348	heat shock protein 90kDa alpha (cytosolic), class A member 1 (HSP90AA1), transcript variant 2, mRNA [NM_005348]	0.76963253	0.40700356
NM_000791	dihydrofolate reductase (DHFR), mRNA [NM_000791]	0.76782741	0.26013021

NM_00101480९	collapsin response mediator protein 1 (CRMP1), transcript variant 1, mRNA [NM_001014809]	0.7618326	0.18761502
NM_173515	CNKSR family member 3 (CNKSR3), mRNA [NM_173515]	0.76153209	0.35677088
NM_014783	Rho GTPase activating protein 11A (ARHGAP11A), transcript variant 1, mRNA [NM_014783]	0.75979126	0.14743862
NM_006260	DnaJ (Hsp40) homolog, subfamily C, member 3 (DNAJC3), mRNA [NM_006260]	0.75971811	0.47670101
NM_006332	interferon, gamma-inducible protein 30 (IFI30), mRNA [NM_006332]	0.75936834	0.42464583
NM_000876	insulin-like growth factor 2 receptor (IGF2R), mRNA [NM_000876]	0.75905339	0.13966054
NM_182620	family with sequence similarity 33, member A (FAM33A), mRNA [NM_182620]	0.7589423	0.30363588
NM_006254	protein kinase C, delta (PRKCD), transcript variant 1, mRNA [NM_006254]	0.75869641	0.03512487
NM_00100756	insulin-like growth factor binding protein-like 1 (IGFBPL1), mRNA [NM_001007563]	0.75806103	0.36909533
NM_207042	endosulfine alpha (ENSA), transcript variant 1, mRNA [NM_207042]	0.75567556	0.39727863
THC2309737	Unknown	0.75147493	0.430683
NM_021127	phorbol-12-myristate-13-acetate-induced protein 1 (PMAIP1), mRNA [NM_021127]	0.75013298	0.30757852
BC019824	Homo sapiens, clone IMAGE:4454331, mRNA. [BC019824]	0.74981651	0.3273394
NM_000430	platelet-activating factor acetylhydrolase, isoform Ib, alpha subunit 45kDa (PAFAH1B1), mRNA [NM_000430]	0.74968267	0.14411546
AK091784	cDNA FLJ34465 fis, clone HLUNG2003061. [AK091784]	0.74946685	0.35713647
NM_007308	synuclein, alpha (non A4 component of amyloid precursor) (SNCA), transcript variant NACP112, mRNA [NM_007308]	0.74864961	0.11407393
NM_176812	chromatin modifying protein 4B (CHMP4B), mRNA [NM_176812]	0.74822109	0.04257524
NM_015355	suppressor of zeste 12 homolog (Drosophila) (SUZ12), mRNA [NM_015355]	0.74672407	0.06609279
NM_021971	GDP-mannose pyrophosphorylase B (GMPPB), transcript variant 2, mRNA [NM_021971]	0.74645977	0.00447798
NM_014638	phospholipase C, eta 2 (PLCH2), mRNA [NM_014638]	0.74457416	0.16257725
NM_001812	centromere protein C 1 (CENPC1), mRNA [NM_001812]	0.74169709	0.09770817
NM_022170	Williams-Beuren syndrome chromosome region 1 (WBSCR1), transcript variant 1, mRNA [NM_022170]	0.73859485	0.08639136
NM_001164	amyloid beta (A4) precursor protein-binding, family B, member 1 (Fe65) (APBB1), transcript variant 1, mRNA [NM_001164]	0.73744694	0.28794773
NM_017846	tRNA selenocysteine associated protein 1 (TRSPAP1), mRNA [NM_017846]	0.73645569	0.13103732
THC2398077	BG978199 RC4-CI0194-060201-015-g03 CI0194 cDNA, mRNA sequence [BG978199]	0.73483152	0.05432177
NM_005194	CCAAT/enhancer binding protein (C/EBP), beta (CEBPB), mRNA [NM_005194]	0.73479391	0.14717822
NM_021831	hypothetical protein FLJ21839 (FLJ21839), transcript variant 2, mRNA [NM_021831]	0.73440996	0.14070029
ENST0000028९	full-length cDNA clone CS0DC011YO02 of Neuroblastoma Cot 25-normalized of (human). [CR611582]	0.73312797	0.21646802
NM_145206	vesicle transport through interaction with t-SNAREs homolog 1A (yeast) (VTI1A), mRNA [NM_145206]	0.73263462	0.26422571
NM_013443	ST6 (alpha-N-acetyl-neuraminyl-2,3-beta-galactosyl-1,3)-N-acetylgalactosaminide alpha-2,6-sialyltransferase 6 (ST6GALNAC6), mRNA [NM_013443]	0.73219994	0.22996807
NM_002660	phospholipase C, gamma 1 (PLCG1), transcript variant 1, mRNA [NM_002660]	0.73219631	0.11929561
NM_173515	CNKSR family member 3 (CNKSR3), mRNA [NM_173515]	0.73186966	0.08477701
ENST0000037	Q99KF9 (Q99KF9) Lrpprc protein, partial (39%) [THC2336834]	0.7316436	0.24796509
NM_021083	X-linked Kx blood group (McLeod syndrome) (XK), mRNA [NM_021083]	0.72938779	0.29347217
NM_014737	Ras association (RalGDS/AF-6) domain family 2 (RASSF2), transcript variant 1, mRNA [NM_014737]	0.72778378	0.3118434
NM_015440	methylenetetrahydrofolate dehydrogenase (NADP+ dependent) 1-like (MTHFD1L), mRNA [NM_015440]	0.72726198	0.18227024

NM_178819	1-acylglycerol-3-phosphate O-acyltransferase 6 (lysophosphatidic acid acyltransferase, zeta) (AGPAT6), mRNA [NM_178819]	0.72704035	0.12805924
NM_015541	leucine-rich repeats and immunoglobulin-like domains 1 (LRIG1), mRNA [NM_015541]	0.72702789	0.1563284
NM_006821	acyl-CoA thioesterase 2 (ACOT2), mRNA [NM_006821]	0.72654092	0.46155412
NM_033020	tripartite motif-containing 33 (TRIM33), transcript variant b, mRNA [NM_033020]	0.72625809	0.42644557
AF533250	zinc finger protein (ZNF397) mRNA, complete cds. [AF533250]	0.72596541	0.14677893
NM_012286	mortality factor 4 like 2 (MORF4L2), mRNA [NM_012286]	0.72440884	0.24413386
NM_024109	chromosome 16 open reading frame 68 (C16orf68), mRNA [NM_024109]	0.72270281	0.38986023
NM_018294	CWF19-like 1, cell cycle control (S. pombe) (CWF19L1), mRNA [NM_018294]	0.72115221	0.21125828
NM_005613	regulator of G-protein signalling 4 (RGS4), mRNA [NM_005613]	0.71976726	0.1644908
NM_005489	SH2 domain containing 3C (SH2D3C), transcript variant 1, mRNA [NM_005489]	0.71958275	0.10357498
NM_152515	cytoskeleton associated protein 2-like (CKAP2L), mRNA [NM_152515]	0.71888854	0.1945097
THC2338229	Unknown	0.71842966	0.43792652
NM_030752	t-complex 1 (TCP1), transcript variant 1, mRNA [NM_030752]	0.71747	0.06537104
NM_017905	transmembrane and coiled-coil domains 3 (TMCO3), mRNA [NM_017905]	0.71704041	0.09761811
NM_007086	WD repeat and HMG-box DNA binding protein 1 (WDHD1), transcript variant 1, mRNA [NM_007086]	0.71683041	0.10257281
NM_002093	glycogen synthase kinase 3 beta (GSK3B), mRNA [NM_002093]	0.71681345	0.3221761
ENST000003679	cDNA FLJ43132 fis, clone CTONG3005813. [AK125122]	0.71670802	0.08158649
NM_031407	HECT, UBA and WWE domain containing 1 (HUWE1), mRNA [NM_031407]	0.71648715	0.05943547
NM_032348	matrix-remodelling associated 8 (MXRA8), mRNA [NM_032348]	0.71640324	0.12417457
NM_024111	ChaC, cation transport regulator-like 1 (E. coli) (CHAC1), mRNA [NM_024111]	0.7129535	0.30633799
NM_023939	hypothetical protein MGC2752 (MGC2752), mRNA [NM_023939]	0.71138726	0.05879243
NM_025248	SNAP25-interacting protein (SNIP), mRNA [NM_025248]	0.71122784	0.21028083
NM_005721	ARP3 actin-related protein 3 homolog (yeast) (ACTR3), mRNA [NM_005721]	0.71084021	0.23101244
NM_012133	coatomer protein complex, subunit gamma 2 (COPG2), mRNA [NM_012133]	0.7097109	0.13797946
NM_006662	Snf2-related CBP activator protein (SRCAP), mRNA [NM_006662]	0.7092642	0.22986242
NM_022371	torsin family 3, member A (TOR3A), mRNA [NM_022371]	0.7086128	0.02022356
NM_001291	CDC-like kinase 2 (CLK2), transcript variant 2, mRNA [NM_001291]	0.70780788	0.31249389
NM_00101339{	insulin-like growth factor binding protein 3 (IGFBP3), transcript variant 1, mRNA [NM_001013398]	0.7070595	0.23828693
AB028977	mRNA for KIAA1054 protein, partial cds. [AB028977]	0.7066978	0.02513
NM_002375	microtubule-associated protein 4 (MAP4), transcript variant 1, mRNA [NM_002375]	0.70647117	0.42328183
A_24_P533142	Unknown	0.70566094	0.21660331
THC2305638	Unknown	0.70495005	0.1260395
NM_00100529	sterol regulatory element binding transcription factor 1 (SREBF1), transcript variant 1, mRNA [NM_001005291]	0.70411762	0.28339362
NM_005765	ATPase, H+ transporting, lysosomal accessory protein 2 (ATP6AP2), mRNA [NM_005765]	0.70312135	0.18488542
NM_031232	amyloid beta (A4) precursor protein-binding, family A, member 2 binding protein (APBA2BP), transcript variant 2, mRNA [NM_031232]	0.70196515	0.16006657
NM_000389	cyclin-dependent kinase inhibitor 1A (p21, Cip1) (CDKN1A), transcript variant 1, mRNA [NM_000389]	0.70171442	0.030937
NM_006979	solute carrier family 39 (zinc transporter), member 7 (SLC39A7), mRNA [NM_006979]	0.7016027	0.04854022
NM_138346	KIAA2013 (KIAA2013), mRNA [NM_138346]	0.70153278	0.23616654
ENST00000359	cDNA: FLJ20958 fis, clone ADSE02059. [AK024611]	0.69958469	0.30580083

NM_000484	amyloid beta (A4) precursor protein (peptidase nexin-II, Alzheimer disease) (APP), transcript variant 1, mRNA [NM_000484]	0.69946549	0.26217397
AF336795	NM-4 mRNA, complete cds. [AF336795]	0.69919608	0.16747225
NM_004737	like-glycosyltransferase (LARGE), transcript variant 1, mRNA [NM_004737]	0.6977334	0.20543469
NM_015039	nicotinamide nucleotide adenylyltransferase 2 (NMNAT2), transcript variant 1, mRNA [NM_015039]	0.69755776	0.38332546
NM_016120	ring finger protein 12 (RNF12), transcript variant 1, mRNA [NM_016120]	0.69748134	0.39394541
NM_052916	ring finger protein 157 (RNF157), mRNA [NM_052916]	0.69456342	0.10960735
ENST000003307	full length insert cDNA YO73E04. [AF075060]	0.69393661	0.25411253
NM_003104	sorbitol dehydrogenase (SORD), mRNA [NM_003104]	0.69385573	0.10048674
NM_018085	importin 9 (IPO9), mRNA [NM_018085]	0.691142	0.16708845
NM_020912	FLYWCH-type zinc finger 1 (FLYWCH1), transcript variant 2, mRNA [NM_020912]	0.68825966	0.06034718
NM_016570	ERGIC and golgi 2 (ERGIC2), mRNA [NM_016570]	0.68670618	0.36379843
NM_133369	unc-5 homolog A (C. elegans) (UNC5A), mRNA [NM_133369]	0.68621473	0.3668066
NM_012324	mitogen-activated protein kinase 8 interacting protein 2 (MAPK8IP2), transcript variant 1, mRNA [NM_012324]	0.68616928	0.12441891
NM_003202	transcription factor 7 (T-cell specific, HMG-box) (TCF7), transcript variant 1, mRNA [NM_003202]	0.68571499	0.17201346
NM_198079	golgi autoantigen, golgin subfamily a-like (FLJ40113), mRNA [NM_198079]	0.68302747	0.31741166
NM_018019	mediator of RNA polymerase II transcription, subunit 9 homolog (S. cerevisiae) (MED9), mRNA [NM_018019]	0.68229815	0.06675365
A_24_P478940	Unknown	0.68223247	0.25771828
NM_00101340	family with sequence similarity 27, member E2 (FAM27E2), mRNA [NM_001013404]	0.68030679	0.11615795
NM_015995	Kruppel-like factor 13 (KLF13), mRNA [NM_015995]	0.67732519	0.05824933
NM_020478	ankyrin 1, erythrocytic (ANK1), transcript variant 5, mRNA [NM_020478]	0.67617054	0.15099125
THC2319743	Q5TAY6 (Q5TAY6) OTTHUMP00000046033, partial (14%) [THC2319743]	0.67557546	0.35762641
NM_013339	asparagine-linked glycosylation 6 homolog (S. cerevisiae, alpha-1,3-glucosyltransferase) (ALG6), mRNA [NM_013339]	0.67349172	0.29572502
NM_203351	mitogen-activated protein kinase kinase kinase 3 (MAP3K3), transcript variant 1, mRNA [NM_203351]	0.67076591	0.01982998
NM_012333	c-myc binding protein (MYCBP), mRNA [NM_012333]	0.67022855	0.03741948
NM_014181	HSPC159 protein (HSPC159), mRNA [NM_014181]	0.66885338	0.14685118
NM_014694	ADAMTS-like 2 (ADAMTSL2), mRNA [NM_014694]	0.66708092	0.36095322
NM_004681	eukaryotic translation initiation factor 1A, Y-linked (EIF1AY), mRNA [NM_004681]	0.66582459	0.10033552
NM_000904	NAD(P)H dehydrogenase, quinone 2 (NQO2), mRNA [NM_000904]	0.66562275	0.05620444
NM_003818	CDP-diacylglycerol synthase (phosphatidate cytidylyltransferase) 2 (CDS2), mRNA [NM_003818]	0.66463027	0.0389651
NM_012175	F-box protein 3 (FBXO3), transcript variant 1, mRNA [NM_012175]	0.66319725	0.17580583
NM_006401	acidic (leucine-rich) nuclear phosphoprotein 32 family, member B (ANP32B), mRNA [NM_006401]	0.66161983	0.13479859
NM_003749	insulin receptor substrate 2 (IRS2), mRNA [NM_003749]	0.66070292	0.13523395
NM_022834	von Willebrand factor A domain containing 1 (VWA1), transcript variant 1, mRNA [NM_022834]	0.66041818	0.22484625
NM_016075	vacuolar protein sorting 36 (yeast) (VPS36), mRNA [NM_016075]	0.65961316	0.02902722
NM_032637	S-phase kinase-associated protein 2 (p45) (SKP2), transcript variant 2, mRNA [NM_032637]	0.6589045	0.07827031

NM_019009	toll interacting protein (TOLLIP), mRNA [NM_019009]	0.65822215	0.12661609
NM_020182	transmembrane, prostate androgen induced RNA (TMEPAI), transcript variant 1, mRNA [NM_020182]	0.65809999	0.15413814
NM_017582	ubiquitin-conjugating enzyme E2Q (putative) 1 (UBE2Q1), mRNA [NM_017582]	0.65741142	0.10921463
NM_000389	cyclin-dependent kinase inhibitor 1A (p21, Cip1) (CDKN1A), transcript variant 1, mRNA [NM_000389]	0.65709166	0.14914025
NM_017488	adducin 2 (beta) (ADD2), transcript variant beta-4, mRNA [NM_017488]	0.65681382	0.24457405
NM_006278	ST3 beta-galactoside alpha-2,3-sialyltransferase 4 (ST3GAL4), mRNA [NM_006278]	0.65645837	0.10793936
NM_022807	small nuclear ribonucleoprotein polypeptide N (SNRPN), transcript variant 4, mRNA [NM_022807]	0.65494529	0.03337131
NM_018950	major histocompatibility complex, class I, F (HLA-F), mRNA [NM_018950]	0.65400644	0.11349589
NM_001995	acyl-CoA synthetase long-chain family member 1 (ACSL1), mRNA [NM_001995]	0.65398269	0.1514223
NM_016203	protein kinase, AMP-activated, gamma 2 non-catalytic subunit (PRKAG2), transcript variant a, mRNA [NM_016203]	0.653732	0.15947832
NM_003098	syntrophin, alpha 1 (dystrophin-associated protein A1, 59kDa, acidic component) (SNTA1), mRNA [NM_003098]	0.6534717	0.00217925
NM_002207	integrin, alpha 9 (ITGA9), mRNA [NM_002207]	0.65259551	0.28468166
NM_00101795(	amplified in osteosarcoma (OS9), transcript variant 2, mRNA [NM_001017956]	0.6519941	0.2926467
NM_000314	phosphatase and tensin homolog (mutated in multiple advanced cancers 1) (PTEN), mRNA [NM_000314]	0.65187249	0.34033995
NM_002912	REV3-like, catalytic subunit of DNA polymerase zeta (yeast) (REV3L), mRNA [NM_002912]	0.65010421	0.0863409
NM_145213	mitochondrial ribosomal protein L30 (MRPL30), nuclear gene encoding mitochondrial protein, transcript variant 3, mRNA [NM_145213]	0.64953724	0.06806445
NM_194455	KRIT1, ankyrin repeat containing (KRIT1), transcript variant 4, mRNA [NM_194455]	0.64776791	0.12323545
NM_000401	exostoses (multiple) 2 (EXT2), transcript variant 1, mRNA [NM_000401]	0.64744351	0.1927281
NM_012310	kinesin family member 4A (KIF4A), mRNA [NM_012310]	0.64631714	0.15102137
NM_005076	contactin 2 (axonal) (CNTN2), mRNA [NM_005076]	0.64606583	0.259774
NM_152429	chromosome 10 open reading frame 13 (C10orf13), mRNA [NM_152429]	0.64576142	0.22803035
NM_005202	collagen, type VIII, alpha 2 (COL8A2), mRNA [NM_005202]	0.64553048	0.20127345
CR605947	full-length cDNA clone CS0DD004YC02 of Neuroblastoma Cot 50-normalized of (human). [CR605947]	0.64396185	0.11934193
NM_015208	ankyrin repeat domain 12 (ANKRD12), mRNA [NM_015208]	0.64286967	0.1416827
NM_004337	chromosome 8 open reading frame 1 (C8orf1), mRNA [NM_004337]	0.64117794	0.25859653
A_24_P307424	Unknown	0.64075196	0.08658784
CR616748	full-length cDNA clone CS0DK007YM08 of HeLa cells Cot 25-normalized of (human). [CR616748]	0.6402179	0.21734854
NM_022778	coiled-coil domain containing 21 (CCDC21), mRNA [NM_022778]	0.63997047	0.22935448
BC033829	cDNA clone IMAGE:3856003, partial cds. [BC033829]	0.63983284	0.03297861
NM_174923	coiled-coil domain containing 107 (CCDC107), mRNA [NM_174923]	0.639727	0.25660045
A_24_P609932	Unknown	0.63971449	0.30323362
CR593500	full-length cDNA clone CS0DF014YD20 of Fetal brain of (human). [CR593500]	0.63947467	0.27313044
NM_005347	heat shock 70kDa protein 5 (glucose-regulated protein, 78kDa) (HSPA5), mRNA [NM_005347]	0.63880047	0.2352226
NM_016381	three prime repair exonuclease 1 (TREX1), transcript variant 1, mRNA [NM_016381]	0.63735799	0.05831316
NM_003177	spleen tyrosine kinase (SYK), mRNA [NM_003177]	0.63699457	0.08305197
AF289562	clone pp6337 unknown mRNA. [AF289562]	0.63630725	0.13481546

NM_018455	centromere protein N (CENPN), mRNA [NM_018455]	0.63580492	0.13713545
NM_002928	regulator of G-protein signalling 16 (RGS16), mRNA [NM_002928]	0.63578973	0.00264773
NM_004073	polo-like kinase 3 (Drosophila) (PLK3), mRNA [NM_004073]	0.63535676	0.13314228
NM_00101239	fused toes homolog (mouse) (FTS), transcript variant 1, mRNA [NM_001012398]	0.63530937	0.33224437
AK092888	cDNA FLJ35569 fis, clone SPLEN2005783. [AK092888]	0.63487796	0.27123037
NM_003748	aldehyde dehydrogenase 4 family, member A1 (ALDH4A1), nuclear gene encoding mitochondrial protein, transcript variant P5CDhL, mRNA [NM_003748]	0.63412629	0.15832173
NM_052847	guanine nucleotide binding protein (G protein), gamma 7 (GNG7), mRNA [NM_052847]	0.63342971	0.19802918
NM_003272	G protein-coupled receptor 137B (GPR137B), mRNA [NM_003272]	0.63215986	0.29162375
NM_003676	degenerative spermatocyte homolog 1, lipid desaturase (Drosophila) (DEGS1), transcript variant 1, mRNA [NM_003676]	0.6321005	0.30149458
NM_006876	UDP-GlcNAc:betaGal beta-1,3-N-acetylglucosaminyltransferase 1 (B3GNT1), mRNA [NM_006876]	0.6315012	0.07274607
NM_015264	chromosome 22 open reading frame 9 (C22orf9), transcript variant 1, mRNA [NM_015264]	0.63066614	0.14624753
NM_194247	heterogeneous nuclear ribonucleoprotein A3 (HNRPA3), mRNA [NM_194247]	0.63047543	0.13283511
NM_080926	hypothetical protein similar to KIAA0187 gene product (LOC96610), mRNA [NM_080926]	0.63025238	0.07426751
NM_022443	myeloid leukemia factor 1 (MLF1), mRNA [NM_022443]	0.63024073	0.06879171
NM_000943	peptidylprolyl isomerase C (cyclophilin C) (PPIC), mRNA [NM_000943]	0.6301685	0.10518127
NM_001211	BUB1 budding uninhibited by benzimidazoles 1 homolog beta (yeast) (BUB1B), mRNA [NM_001211]	0.6295528	0.00047366
NM_005032	plastin 3 (T isoform) (PLS3), mRNA [NM_005032]	0.62704876	0.27284636
NM_00102536	vascular endothelial growth factor (VEGF), transcript variant 1, mRNA [NM_001025366]	0.62556176	0.20379453
NM_001237	cyclin A2 (CCNA2), mRNA [NM_001237]	0.62541759	0.0608129
NM_006761	tyrosine 3-monooxygenase/tryptophan 5-monooxygenase activation protein, epsilon polypeptide (YWHAE), mRNA [NM_006761]	0.62377221	0.02273461
BM999256	UI-H-DI0-avo-g-06-0-UI.s1 NCI_CGAP_DI0 cDNA clone IMAGE:5882141 3', mRNA sequence [BM999256]	0.62256522	0.19219858
NM_00101443	drebrin-like (DBNL), transcript variant 2, mRNA [NM_001014436]	0.62250403	0.04045705
NM_025241	UBX domain containing 1 (UBXD1), mRNA [NM_025241]	0.62192147	0.05666951
NM_001034	ribonucleotide reductase M2 polypeptide (RRM2), mRNA [NM_001034]	0.62172262	0.09028659
NM_005779	lipoma HMGIC fusion partner-like 2 (LHFPL2), mRNA [NM_005779]	0.62111102	0.25282405
THC2406815	Q98EK5 (Q98EK5) Sugar ABC transporter, permease, partial (8%) [THC2406815]	0.62023593	0.27156919
NM_004497	forkhead box A3 (FOXA3), mRNA [NM_004497]	0.61753022	0.11983104
NM_006254	protein kinase C, delta (PRKCD), transcript variant 1, mRNA [NM_006254]	0.61713386	0.0513141
NM_003691	serine/threonine kinase 16 (STK16), transcript variant 1, mRNA [NM_003691]	0.61686002	0.06097668
NM_015120	Alstrom syndrome 1 (ALMS1), mRNA [NM_015120]	0.61638668	0.26538911
BX110856	BX110856 Soares adult brain N2b4HB55Y cDNA clone IMAGp998M09331, mRNA sequence [BX110856]	0.61618603	0.02146942
NM_018132	centromere protein Q (CENPQ), mRNA [NM_018132]	0.61347168	0.17684199
NM_005190	cyclin C (CCNC), transcript variant 1, mRNA [NM_005190]	0.61290726	0.19758698
NM_006499	lectin, galactoside-binding, soluble, 8 (galectin 8) (LGALS8), transcript variant 1, mRNA [NM_006499]	0.61279046	0.25171774
NM_146421	glutathione S-transferase M1 (GSTM1), transcript variant 2, mRNA [NM_146421]	0.61246734	0.10426414
NM_00101799	SH3 and PX domains 2B (SH3PXD2B), mRNA [NM_001017995]	0.61209969	0.1454346
NM_207191	ADAM metallopeptidase domain 15 (metargidin) (ADAM15), transcript variant 1, mRNA [NM_207191]	0.61131901	0.1868493

NM_005773	zinc finger protein 256 (ZNF256), mRNA [NM_005773]	0.61005391	0.16456987
AA359500	AA359500 EST68526 Fetal lung II cDNA 5' end, mRNA sequence [AA359500]	0.60993499	0.28346163
A_32_P229447	Unknown	0.60952798	0.13431997
NM_145019	hypothetical protein FLJ30707 (FLJ30707), mRNA [NM_145019]	0.6087711	0.16538055
NM_020379	mannosidase, alpha, class 1C, member 1 (MAN1C1), mRNA [NM_020379]	0.60748932	0.29127496
NM_153713	Lix1 homolog (mouse) like (LIX1L), mRNA [NM_153713]	0.60748519	0.08781371
NM_024050	DDA1 (DDA1), mRNA [NM_024050]	0.60652815	0.10086511
AI818152	wk27g08.x1 NCI_CGAP_Brn25 cDNA clone IMAGE:2413598 3' similar to gb:X74795 CDC46 HOMOLOG (HUMAN);, mRNA sequence [AI818152]	0.60600057	0.02358606
A_23_P392897	Unknown	0.60596499	0.00723768
AK125299	cDNA FLJ43309 fis, clone NT2RI2004618, highly similar to Cytosolic acyl coenzyme A thioester hydrolase (EC 3.1.2.2). [AK125299]	0.60557968	0.15842787
NM_000527	low density lipoprotein receptor (familial hypercholesterolemia) (LDLR), mRNA [NM_000527]	0.6053223	0.13888326
ENST00000332!	Unknown	0.60504431	0.01056856
NM_015944	amidohydrolase domain containing 2 (AMDHD2), mRNA [NM_015944]	0.60429399	0.19936086
THC2398598	CO1A_HUMAN (P31146) Coronin-1A (Coronin-like protein p57) (Coronin-like protein A) (CLIPINA) (Tryptophan aspartate-containing coat protein) (TACO), partial (91%) [THC2398598]	0.60368669	0.04320554
NM_153824	pyrroline-5-carboxylate reductase 1 (PYCR1), transcript variant 2, mRNA [NM_153824]	0.60345319	0.02774555
NM_001670	armadillo repeat gene deletes in velocardiofacial syndrome (ARVCF), mRNA [NM_001670]	0.60258335	0.26604133
NM_024623	2-oxoglutarate and iron-dependent oxygenase domain containing 2 (OGFOD2), mRNA [NM_024623]	0.60094491	0.20320264
NM_018385	large subunit GTPase 1 homolog (S. cerevisiae) (LSG1), mRNA [NM_018385]	0.60086657	0.03805958
BU731317	BU731317 UI-E-CI1-afr-o-04-0-UI.s1 UI-E-CI1 cDNA clone UI-E-CI1-afr-o-04-0-UI 3', mRNA sequence [BU731317]	0.60043007	0.25558525
NM_002982	chemokine (C-C motif) ligand 2 (CCL2), mRNA [NM_002982]	-5.82146801	3.41070512
NM_002982	chemokine (C-C motif) ligand 2 (CCL2), mRNA [NM_002982]	-4.83111351	1.22624382
NM_002982	chemokine (C-C motif) ligand 2 (CCL2), mRNA [NM_002982]	-3.81527607	0.01065326
NM_002982	chemokine (C-C motif) ligand 2 (CCL2), mRNA [NM_002982]	-3.81211522	0.13752295
ENST00000314(	full-length cDNA clone CS0DK009YH13 of HeLa cells Cot 25-normalized of (human). [CR624886]	-3.7811513	2.27859532
NM_000891	potassium inwardly-rectifying channel, subfamily J, member 2 (KCNJ2), mRNA [NM_000891]	-3.76846114	3.19348307
NM_002982	chemokine (C-C motif) ligand 2 (CCL2), mRNA [NM_002982]	-3.66940267	0.01401875
NM_002982	chemokine (C-C motif) ligand 2 (CCL2), mRNA [NM_002982]	-3.65601041	0.03841028
NM_003106	SRY (sex determining region Y)-box 2 (SOX2), mRNA [NM_003106]	-3.15824342	2.12796962
NM_174940	transmembrane protein 80 (TMEM80), transcript variant 1, mRNA [NM_174940]	-3.04902878	1.2324708
NM_004994	matrix metallopeptidase 9 (gelatinase B, 92kDa gelatinase, 92kDa type IV collagenase) (MMP9), mRNA [NM_004994]	-3.04321801	0.42627189
NM_000582	secreted phosphoprotein 1 (osteopontin, bone sialoprotein I, early T-lymphocyte activation 1) (SPP1), transcript variant 2, mRNA [NM_000582]	-3.03858462	2.82271393
AK056119	cDNA FLJ31557 fis, clone NT2RI2001307. [AK056119]	-3.01833736	2.93748326
NM_006785	mucosa associated lymphoid tissue lymphoma translocation gene 1 (MALT1), transcript variant 1, mRNA [NM_006785]	-2.96079435	2.93201761

NM_173847	sperm acrosome associated 3 (SPACA3), mRNA [NM_173847]	-2.94886354	1.01076084
NM_003381	vasoactive intestinal peptide (VIP), transcript variant 1, mRNA [NM_003381]	-2.90995061	0.04240835
BI262095	BI262095 602953519T1 NIH_MGC_99 cDNA clone IMAGE:5087621 3', mRNA sequence [BI262095]	-2.63715615	0.53926215
NM_018199	exonuclease 3'-5' domain-like 2 (EXDL2), mRNA [NM_018199]	-2.57878604	0.93385143
NM_207326	hypothetical protein LOC149134 (LOC149134), mRNA [NM_207326]	-2.55461159	0.31540501
NM_000582	secreted phosphoprotein 1 (osteopontin, bone sialoprotein I, early T-lymphocyte activation 1) (SPP1), transcript variant 2, mRNA [NM_000582]	-2.54110274	2.28765733
NM_003955	suppressor of cytokine signaling 3 (SOCS3), mRNA [NM_003955]	-2.53701261	0.43773491
NM_133638	ADAM metallopeptidase with thrombospondin type 1 motif, 19 (ADAMTS19), mRNA [NM_133638]	-2.53674624	1.26749635
A_24_P914075	Unknown	-2.52920017	0.63687165
NM_058197	cyclin-dependent kinase inhibitor 2A (melanoma, p16, inhibits CDK4) (CDKN2A), transcript variant 3, mRNA [NM_058197]	-2.39651427	0.15627473
NM_00103241[illegible]	FKSG73 protein (LOC440888), mRNA [NM_001032412]	-2.3686657	0.09066714
NM_006983	matrix metallopeptidase 23B (MMP23B), mRNA [NM_006983]	-2.32103064	2.26201565
NM_006788	ralA binding protein 1 (RALBP1), mRNA [NM_006788]	-2.29162363	0.52774246
AK022213	cDNA FLJ12151 fis, clone MAMMA1000431. [AK022213]	-2.28952698	2.22101544
NM_025114	centrosomal protein 290kDa (CEP290), mRNA [NM_025114]	-2.28874525	1.8969957
NM_00100694[illegible]	syndecan 1 (SDC1), transcript variant 1, mRNA [NM_001006946]	-2.26207336	1.41553731
NM_003106	SRY (sex determining region Y)-box 2 (SOX2), mRNA [NM_003106]	-2.23699267	1.93806053
THC2364621	AF014891 NADH dehydrogenase subunit 2 {Homo sapiens;} , partial (10%) [THC2364621]	-2.18836835	0.46437651
NM_018130	SHQ1 homolog (S. cerevisiae) (SHQ1), mRNA [NM_018130]	-2.17279651	0.03559183
NM_001798	cyclin-dependent kinase 2 (CDK2), transcript variant 1, mRNA [NM_001798]	-2.09107494	0.96862859
NM_002602	phosphodiesterase 6G, cGMP-specific, rod, gamma (PDE6G), mRNA [NM_002602]	-2.01182707	1.78096905
NM_144974	hypothetical protein FLJ31846 (FLJ31846), mRNA [NM_144974]	-2.00221491	1.28264108
NM_015368	pannexin 1 (PANX1), mRNA [NM_015368]	-1.98944236	1.69906496
NM_020423	SCY1-like 3 (S. cerevisiae) (SCYL3), transcript variant 1, mRNA [NM_020423]	-1.98100175	0.84337945
NM_003688	calcium/calmodulin-dependent serine protein kinase (MAGUK family) (CASK), mRNA [NM_003688]	-1.97711322	1.57755719
NM_015534	zinc finger, ZZ-type containing 3 (ZZZ3), mRNA [NM_015534]	-1.94556618	1.18624432
ENST0000038[illegible]	piccolo (presynaptic cytomatrix protein), mRNA (cDNA clone IMAGE:3452412), complete cds. [BC001304]	-1.91427614	1.40837811
NM_00100535[illegible]	proline rich 13 (PRR13), transcript variant 3, mRNA [NM_001005354]	-1.91221256	1.86273013
NM_018115	SDA1 domain containing 1 (SDAD1), mRNA [NM_018115]	-1.90060719	0.94280966
AA495894	AA495894 zw04g12.r1 Soares_NhHMPu_S1 cDNA clone IMAGE:768358 5' similar to contains Alu repetitive element;, mRNA sequence [AA495894]	-1.89916874	0.64017824
NM_173851	solute carrier family 30 (zinc transporter), member 8 (SLC30A8), mRNA [NM_173851]	-1.89152122	1.49507574
NM_138444	potassium channel tetramerisation domain containing 12 (KCTD12), mRNA [NM_138444]	-1.87897964	0.01415258
A_24_P934718	Unknown	-1.85968817	1.26018307
NM_032752	zinc finger protein 496 (ZNF496), mRNA [NM_032752]	-1.84251965	0.00500869
THC2369735	1ABW_A Chain A, Deoxy Rhb1.1 (Recombinant Hemoglobin). {synthetic construct;} , partial (16%) [THC2369735]	-1.84052289	0.09676899
NM_152636	methyltransferase 5 domain containing 1 (METT5D1), mRNA [NM_152636]	-1.82569386	1.63697945
NM_006058	TNFAIP3 interacting protein 1 (TNIP1), mRNA [NM_006058]	-1.82548074	0.71414941

NM_000582	secreted phosphoprotein 1 (osteopontin, bone sialoprotein I, early T-lymphocyte activation 1) (SPP1), transcript variant 2, mRNA [NM_000582]	-1.81649744	1.22892492
A_24_P599225	Unknown	-1.80232731	0.15356468
NM_002977	sodium channel, voltage-gated, type IX, alpha (SCN9A), mRNA [NM_002977]	-1.79995544	1.65445737
BC072415	cDNA clone IMAGE:6168734. [BC072415]	-1.7969678	0.20269368
NM_032876	jub, ajuba homolog (Xenopus laevis) (JUB), transcript variant 1, mRNA [NM_032876]	-1.79109465	0.01050819
NM_015092	PI-3-kinase-related kinase SMG-1 (SMG1), mRNA [NM_015092]	-1.79015784	0.91927939
THC2438994	Unknown	-1.76869255	0.44455156
THC2301362	Unknown	-1.75931748	1.17977335
AF424541	unknown mRNA. [AF424541]	-1.75604164	0.57024234
NM_004389	catenin (cadherin-associated protein), alpha 2 (CTNNA2), mRNA [NM_004389]	-1.74787314	0.78537496
THC2364893	HSTMPO6 thymopoietin beta {Homo sapiens;} , partial (89%) [THC2364893]	-1.74289666	1.01535794
NM_058197	cyclin-dependent kinase inhibitor 2A (melanoma, p16, inhibits CDK4) (CDKN2A), transcript variant 3, mRNA [NM_058197]	-1.73944552	1.03542397
NM_032484	homolog of mouse LGP1 (LGP1), mRNA [NM_032484]	-1.73097436	1.41038362
AK091839	cDNA FLJ34520 fis, clone HLUNG2006935, weakly similar to CALCYPHOSINE. [AK091839]	-1.7195892	1.53079832
NM_000055	butyrylcholinesterase (BCHE), mRNA [NM_000055]	-1.71017269	0.67636494
NM_006499	lectin, galactoside-binding, soluble, 8 (galectin 8) (LGALS8), transcript variant 1, mRNA [NM_006499]	-1.70920809	1.01575296
NM_015347	RIMS binding protein 2 (RIMBP2), mRNA [NM_015347]	-1.68825869	1.58620708
NM_173672	peptidylprolyl isomerase (cyclophilin)-like 6 (PPIL6), mRNA [NM_173672]	-1.68775533	1.43791353
NM_002617	peroxisome biogenesis factor 10 (PEX10), transcript variant 2, mRNA [NM_002617]	-1.67713171	0.40704443
NM_014422	phosphatidylinositol (4,5) bisphosphate 5-phosphatase, A (PIB5PA), transcript variant 1, mRNA [NM_014422]	-1.67613145	0.43972255
NM_014839	plasticity related gene 1 (LPPR4), mRNA [NM_014839]	-1.66286733	0.27058782
NM_002529	neurotrophic tyrosine kinase, receptor, type 1 (NTRK1), transcript variant 2, mRNA [NM_002529]	-1.65005007	0.75067458
CR606629	full-length cDNA clone CS0DJ007YF12 of T cells (Jurkat cell line) Cot 10-normalized of (human). [CR606629]	-1.63956992	1.5091442
AK094175	cDNA FLJ36856 fis, clone ASTRO2014863. [AK094175]	-1.63553252	0.64599466
THC2283842	Unknown	-1.61672232	1.09211631
NM_152605	hypothetical protein FLJ37549 (FLJ37549), mRNA [NM_152605]	-1.61205476	1.20791834
NM_005821	neighbor of BRCA1 gene 2 (NBR2), mRNA [NM_005821]	-1.60501889	0.92226185
NM_005358	LIM domain 7 (LMO7), mRNA [NM_005358]	-1.5961006	1.44140917
A_24_P15906	Unknown	-1.58777811	1.2354905
NM_152410	PARK2 co-regulated (PACRG), mRNA [NM_152410]	-1.57806897	0.19608416
NM_005195	CCAAT/enhancer binding protein (C/EBP), delta (CEBPD), mRNA [NM_005195]	-1.55701202	1.50559943
NM_005482	phosphatidylinositol glycan anchor biosynthesis, class K (PIGK), mRNA [NM_005482]	-1.55073199	0.07485737
AJ291676	mRNA for chromosome 8 open reading frame 9 (c8ORF9). [AJ291676]	-1.54659557	0.4665695
NM_015534	zinc finger, ZZ-type containing 3 (ZZZ3), mRNA [NM_015534]	-1.53606206	0.40350258
NM_000164	gastric inhibitory polypeptide receptor (GIPR), mRNA [NM_000164]	-1.51532982	1.31106631
BC089454	cDNA clone MGC:105145 IMAGE:30563285, complete cds. [BC089454]	-1.48625754	0.73704571
NM_203393	hypothetical gene supported by BC031661 (LOC389458), mRNA [NM_203393]	-1.46581354	1.36205258
NM_014892	RNA binding motif protein 16 (RBM16), mRNA [NM_014892]	-1.46345618	0.91329095
NM_004994	matrix metallopeptidase 9 (gelatinase B, 92kDa gelatinase, 92kDa type IV collagenase) (MMP9), mRNA [NM_004994]	-1.46297825	0.29167139

NM_004994	matrix metallopeptidase 9 (gelatinase B, 92kDa gelatinase, 92kDa type IV collagenase) (MMP9), mRNA [NM_004994]	-1.46072964	1.02524204
BC073157	hypothetical protein LOC92482, mRNA (cDNA clone IMAGE:6063114), partial cds. [BC073157]	-1.45609816	0.8074737
NM_005160	adrenergic, beta, receptor kinase 2 (ADRBK2), mRNA [NM_005160]	-1.45251826	0.65235072
NM_138361	leucine rich repeat and sterile alpha motif containing 1 (LRSAM1), transcript variant 1, mRNA [NM_138361]	-1.44959739	1.24418338
NM_030957	ADAM metallopeptidase with thrombospondin type 1 motif, 10 (ADAMTS10), mRNA [NM_030957]	-1.44877578	0.445422
THC2429167	Unknown	-1.4435917	0.12086629
NM_015534	zinc finger, ZZ-type containing 3 (ZZZ3), mRNA [NM_015534]	-1.44175801	1.31869149
BC030122	cDNA clone IMAGE:4814828. [BC030122]	-1.43694522	0.77377077
NM_052849	coiled-coil domain containing 32 (CCDC32), mRNA [NM_052849]	-1.43674691	0.05375452
NM_015428	zinc finger protein 473 (ZNF473), transcript variant 1, mRNA [NM_015428]	-1.43476271	0.20593536
BQ184357	UI-E-EJ1-ajs-n-23-0-UI.s1 UI-E-EJ1 cDNA clone UI-E-EJ1-ajs-n-23-0-UI 3', mRNA sequence [BQ184357]	-1.43147173	0.03214917
AK095214	cDNA FLJ37895 fis, clone BRTHA2004642. [AK095214]	-1.42792682	0.09450212
NM_144670	alpha-2-macroglobulin-like 1 (A2ML1), mRNA [NM_144670]	-1.42069624	1.37640716
A_24_P50281	Unknown	-1.40758577	0.65897287
AK002066	cDNA FLJ11204 fis, clone PLACE1007810. [AK002066]	-1.40493923	1.16969631
BU679017	BU679017 UI-CF-DU1-aat-a-16-0-UI.s1 UI-CF-DU1 cDNA clone UI-CF-DU1-aat-a-16-0-UI 3', mRNA sequence [BU679017]	-1.38993929	0.36790273
NM_025106	splA/ryanodine receptor domain and SOCS box containing 1 (SPSB1), mRNA [NM_025106]	-1.3897285	0.21607001
NM_015901	nudix (nucleoside diphosphate linked moiety X)-type motif 13 (NUDT13), mRNA [NM_015901]	-1.38119455	1.12650074
NM_012407	protein interacting with PRKCA 1 (PICK1), transcript variant 1, mRNA [NM_012407]	-1.38040627	0.29809791
NM_000507	fructose-1,6-bisphosphatase 1 (FBP1), mRNA [NM_000507]	-1.37683075	0.66517149
NM_152486	sterile alpha motif domain containing 11 (SAMD11), mRNA [NM_152486]	-1.36988479	0.31625348
BC047110	cDNA clone IMAGE:5312754. [BC047110]	-1.36777656	0.21679133
NM_004423	dishevelled, dsh homolog 3 (Drosophila) (DVL3), mRNA [NM_004423]	-1.36689332	0.47537089
NM_000582	secreted phosphoprotein 1 (osteopontin, bone sialoprotein I, early T-lymphocyte activation 1) (SPP1), transcript variant 2, mRNA [NM_000582]	-1.36459732	1.1257565
NM_003185	TAF4 RNA polymerase II, TATA box binding protein (TBP)-associated factor, 135kDa (TAF4), mRNA [NM_003185]	-1.36323399	0.56996358
BC050733	ATP-binding cassette, sub-family C (CFTR/MRP), member 6, mRNA (cDNA clone IMAGE:6141205), complete cds. [BC050733]	-1.36257348	0.51280205
NM_138381	oxidoreductase NAD-binding domain containing 1 (OXNAD1), mRNA [NM_138381]	-1.36147277	1.03101271
NM_152829	testis derived transcript (3 LIM domains) (TES), transcript variant 2, mRNA [NM_152829]	-1.35260064	0.1999454
NM_006482	dual-specificity tyrosine-(Y)-phosphorylation regulated kinase 2 (DYRK2), transcript variant 2, mRNA [NM_006482]	-1.34918448	0.1282036
BC007307	Homo sapiens, Similar to zinc finger protein 268, clone IMAGE:3352268, mRNA, partial cds. [BC007307]	-1.34884233	0.7916423
THC2311618	AI452448 tj62b01.x1 Soares_NSF_F8_9W_OT_PA_P_S1 cDNA clone IMAGE:2146057 3', mRNA sequence [AI452448]	-1.34497804	0.29969097
AK096580	cDNA FLJ39261 fis, clone OCBBF2009391. [AK096580]	-1.33319524	0.08761705
AF087999	full length insert cDNA clone YX44E03. [AF087999]	-1.32826656	0.04074647

NM_030634	zinc finger protein 436 (ZNF436), mRNA [NM_030634]	-1.31760128	0.17483869
NM_014182	ORM1-like 2 (S. cerevisiae) (ORMDL2), mRNA [NM_014182]	-1.31652462	0.71001073
THC2437154	Unknown	-1.30654826	1.16846648
NM_152290	chromosome 1 open reading frame 158 (C1orf158), mRNA [NM_152290]	-1.30198341	0.51968145
NM_152736	zinc finger protein 187 (ZNF187), transcript variant 3, mRNA [NM_152736]	-1.29843365	0.68301637
NM_018717	mastermind-like 3 (Drosophila) (MAML3), mRNA [NM_018717]	-1.29471712	0.37143275
THC2439773	Unknown	-1.28818951	0.04704824
NM_004647	D4, zinc and double PHD fingers family 1 (DPF1), mRNA [NM_004647]	-1.28548406	0.63278554
NM_007115	tumor necrosis factor, alpha-induced protein 6 (TNFAIP6), mRNA [NM_007115]	-1.2821167	1.08355542
NM_024617	zinc finger, CCHC domain containing 6 (ZCCHC6), mRNA [NM_024617]	-1.27855738	1.17539245
NM_021945	chromosome 6 open reading frame 85 (C6orf85), mRNA [NM_021945]	-1.27547315	0.31479724
A_23_P122650	Unknown	-1.27246253	0.98994581
NM_002009	fibroblast growth factor 7 (keratinocyte growth factor) (FGF7), mRNA [NM_002009]	-1.26864332	0.28334546
NM_138578	BCL2-like 1 (BCL2L1), nuclear gene encoding mitochondrial protein, transcript variant 1, mRNA [NM_138578]	-1.26690863	0.52084267
NM_012232	polymerase I and transcript release factor (PTRF), mRNA [NM_012232]	-1.26645641	0.3936694
AK091357	cDNA FLJ34038 fis, clone FCBBF2005645. [AK091357]	-1.26544216	0.78714341
THC2292718	Unknown	-1.2613068	1.10278903
NM_005508	chemokine (C-C motif) receptor 4 (CCR4), mRNA [NM_005508]	-1.26119803	0.88430704
NM_007195	polymerase (DNA directed) iota (POLI), mRNA [NM_007195]	-1.24376877	0.47043405
NM_001797	cadherin 11, type 2, OB-cadherin (osteoblast) (CDH11), mRNA [NM_001797]	-1.24174455	0.16340844
AK090515	cDNA FLJ33196 fis, clone ADRGL2006034. [AK090515]	-1.24167491	0.50371064
NM_000898	monoamine oxidase B (MAOB), nuclear gene encoding mitochondrial protein, mRNA [NM_000898]	-1.23890751	0.50207868
NM_018178	golgi phosphoprotein 3-like (GOLPH3L), mRNA [NM_018178]	-1.22987316	0.01446263
NM_002775	HtrA serine peptidase 1 (HTRA1), mRNA [NM_002775]	-1.22219859	1.13887811
ENST0000037631	mRNA; cDNA DKFZp686N2052 (from clone DKFZp686N2052). [BX647989]	-1.21345783	0.66636853
AK095167	cDNA FLJ37848 fis, clone BRSSN2013544. [AK095167]	-1.21017488	0.56655453
NM_145252	similar to common salivary protein 1 (LOC124220), mRNA [NM_145252]	-1.20871515	0.40849231
NM_002064	glutaredoxin (thioltransferase) (GLRX), mRNA [NM_002064]	-1.20529049	0.3306635
NM_080740	suppressor of hairy wing homolog 1 (Drosophila) (SUHW1), mRNA [NM_080740]	-1.20323177	0.71236833
BC047417	cDNA clone IMAGE:5288894. [BC047417]	-1.19929084	0.25119559
NM_021645	UTP14, U3 small nucleolar ribonucleoprotein, homolog C (yeast) (UTP14C), mRNA [NM_021645]	-1.1969705	0.08521033
NM_002210	integrin, alpha V (vitronectin receptor, alpha polypeptide, antigen CD51) (ITGAV), mRNA [NM_002210]	-1.19682742	0.33487569
A_24_P195454	Unknown	-1.19640142	1.06882487
NM_182908	dehydrogenase/reductase (SDR family) member 2 (DHRS2), transcript variant 1, mRNA [NM_182908]	-1.19373468	0.68842475
XM_931434	PREDICTED: hypothetical gene supported by BC047417, transcript variant 2 (LOC400027), mRNA [XM_931434]	-1.18912779	0.23338341
NM_001560	interleukin 13 receptor, alpha 1 (IL13RA1), mRNA [NM_001560]	-1.18353268	0.38969559
NM_212482	fibronectin 1 (FN1), transcript variant 1, mRNA [NM_212482]	-1.18144416	0.57907859
THC2379232	Unknown	-1.17566797	0.71954646
NM_014959	caspase recruitment domain family, member 8 (CARD8), mRNA [NM_014959]	-1.17379606	0.03586617
NM_007218	ring finger protein 139 (RNF139), mRNA [NM_007218]	-1.17034234	0.441871

NM_002497	NIMA (never in mitosis gene a)-related kinase 2 (NEK2), mRNA [NM_002497]	-1.16894545	0.76489357
NM_000313	protein S (alpha) (PROS1), mRNA [NM_000313]	-1.16667764	0.15981521
THC2439426	Unknown	-1.1656315	0.17624996
NM_000090	collagen, type III, alpha 1 (Ehlers-Danlos syndrome type IV, autosomal dominant) (COL3A1), mRNA [NM_000090]	-1.16547589	0.02703809
NM_00103308(	chromosome 20 open reading frame 133 (C20orf133), transcript variant 1, mRNA [NM_001033086]	-1.15496247	0.08728335
NM_00103990(	hypothetical protein LOC644975 (FLJ30064), mRNA [NM_001039906]	-1.14987823	1.03932666
THC2338292	Unknown	-1.14972723	0.62102167
AL136548	mRNA; cDNA DKFZp761G18121 (from clone DKFZp761G18121). [AL136548]	-1.14701509	0.24011319
BC012452	transmembrane protein 76, mRNA (cDNA clone IMAGE:3880903), complete cds. [BC012452]	-1.145495	0.19981765
NM_023011	UPF3 regulator of nonsense transcripts homolog A (yeast) (UPF3A), transcript variant 1, mRNA [NM_023011]	-1.14501208	0.95750762
NM_018933	protocadherin beta 13 (PCDHB13), mRNA [NM_018933]	-1.14392669	0.15027396
THC2316748	Unknown	-1.14269033	0.82225605
NM_000610	CD44 molecule (Indian blood group) (CD44), transcript variant 1, mRNA [NM_000610]	-1.14082302	0.33087043
AI541080	pec1.2-1.H02 ecnorm cDNA 3', mRNA sequence [AI541080]	-1.14073809	0.09103912
THC2382264	Unknown	-1.13579091	0.86144001
AK057652	cDNA FLJ33090 fis, clone TRACH2000559. [AK057652]	-1.13265034	0.83219294
NM_002227	Janus kinase 1 (a protein tyrosine kinase) (JAK1), mRNA [NM_002227]	-1.13163691	0.04665093
CR936771	mRNA; cDNA DKFZp686A0668 (from clone DKFZp686A0668). [CR936771]	-1.13048688	0.28759538
NM_004994	matrix metallopeptidase 9 (gelatinase B, 92kDa gelatinase, 92kDa type IV collagenase) (MMP9), mRNA [NM_004994]	-1.12450647	0.93598317
NM_033305	vacuolar protein sorting 13 homolog A (S. cerevisiae) (VPS13A), transcript variant A, mRNA [NM_033305]	-1.11105438	0.88550263
NM_000582	secreted phosphoprotein 1 (osteopontin, bone sialoprotein I, early T-lymphocyte activation 1) (SPP1), transcript variant 2, mRNA [NM_000582]	-1.10510905	0.3593335
NM_024584	hypothetical protein FLJ13646 (FLJ13646), mRNA [NM_024584]	-1.10120245	0.26114223
AK126622	cDNA FLJ44660 fis, clone BRACE3002344, weakly similar to Vegetatible incompatibility protein HET-E-1. [AK126622]	-1.10090197	0.94387022
NM_015288	PHD finger protein 15 (PHF15), mRNA [NM_015288]	-1.10039082	0.36870374
NM_058197	cyclin-dependent kinase inhibitor 2A (melanoma, p16, inhibits CDK4) (CDKN2A), transcript variant 3, mRNA [NM_058197]	-1.09816244	0.27998375
NM_006509	v-rel reticuloendotheliosis viral oncogene homolog B, nuclear factor of kappa light polypeptide gene enhancer in B-cells 3 (avian) (RELB), mRNA [NM_006509]	-1.0938287	0.20579064
NM_152523	hypothetical protein FLJ40432 (FLJ40432), mRNA [NM_152523]	-1.09323209	0.03549074
NM_133456	apical protein 2 (APXL2), mRNA [NM_133456]	-1.09192378	0.36896216
AA594808	AA594808 nn86f10.s1 NCI_CGAP_Br2 cDNA clone IMAGE:1098091 3', mRNA sequence [AA594808]	-1.09023542	0.46284612
THC2287450	Unknown	-1.08904965	0.26802652
NM_000201	intercellular adhesion molecule 1 (CD54), human rhinovirus receptor (ICAM1), mRNA [NM_000201]	-1.08205334	0.87525266
AK001846	cDNA FLJ10984 fis, clone PLACE1001810. [AK001846]	-1.08166564	0.16193262
NM_020831	megakaryoblastic leukemia (translocation) 1 (MKL1), mRNA [NM_020831]	-1.07634283	0.43589932
NM_004275	Trf (TATA binding protein-related factor)-proximal homolog (Drosophila) (TRFP), mRNA [NM_004275]	-1.07424278	0.89163679

NM_015216	Histidine acid phosphatase domain containing 1 (HISPPD1), mRNA [NM_015216]	-1.07224186	0.06855901
ENST0000381:	IL6B_HUMAN (P40189) Interleukin-6 receptor beta chain precursor (IL-6R-beta) (Interleukin 6 signal transducer) (Membrane glycoprotein 130) (gp130) (Oncostatin M receptor) (CDw130) (CD130 antigen), partial (27%) [THC2315176]	-1.06724847	0.47970153
NM_002012	fragile histidine triad gene (FHIT), mRNA [NM_002012]	-1.06591956	0.91059417
BG031574	602299712F1 NIH_MGC_87 cDNA clone IMAGE:4394138 5', mRNA sequence [BG031574]	-1.06249277	0.05974845
NM_003407	zinc finger protein 36, C3H type, homolog (mouse) (ZFP36), mRNA [NM_003407]	-1.06193044	0.3288537
NM_00100368	ubiquinol-cytochrome c reductase complex (7.2 kD) (UCRC), transcript variant 2, mRNA [NM_001003684]	-1.06171327	0.20322965
NM_178566	zinc finger, DHHC-type containing 21 (ZDHHC21), mRNA [NM_178566]	-1.05956145	0.42427942
NM_018061	PRP38 pre-mRNA processing factor 38 (yeast) domain containing B (PRPF38B), mRNA [NM_018061]	-1.05693957	0.77439388
NM_002213	integrin, beta 5 (ITGB5), mRNA [NM_002213]	-1.05002645	0.1284667
NM_002737	protein kinase C, alpha (PRKCA), mRNA [NM_002737]	-1.0494756	0.21022502
NM_007281	scrapie responsive protein 1 (SCRG1), mRNA [NM_007281]	-1.04655623	0.61300709
NM_019119	protocadherin beta 9 (PCDHB9), mRNA [NM_019119]	-1.04396809	0.51623985
NM_031301	anterior pharynx defective 1 homolog B (C. elegans) (APH1B), mRNA [NM_031301]	-1.04385687	0.62812008
NM_007106	ubiquitin-like 3 (UBL3), mRNA [NM_007106]	-1.04298705	0.52649833
NM_018341	chromosome 6 open reading frame 70 (C6orf70), mRNA [NM_018341]	-1.04136775	0.11025452
NM_022787	nicotinamide nucleotide adenylyltransferase 1 (NMNAT1), mRNA [NM_022787]	-1.03996918	0.86413969
THC2264965	Unknown	-1.03920304	0.5265559
NM_002737	protein kinase C, alpha (PRKCA), mRNA [NM_002737]	-1.03872038	0.84876202
NM_014746	ring finger protein 144 (RNF144), mRNA [NM_014746]	-1.0385802	0.87211051
NM_004994	matrix metallopeptidase 9 (gelatinase B, 92kDa gelatinase, 92kDa type IV collagenase) (MMP9), mRNA [NM_004994]	-1.03808748	0.64986838
NM_005979	S100 calcium binding protein A13 (S100A13), transcript variant 2, mRNA [NM_005979]	-1.03685127	0.23332216
NM_152858	Wilms tumor 1 associated protein (WTAP), transcript variant 3, mRNA [NM_152858]	-1.03184786	0.53582341
NM_030626	leucine rich repeat containing 27 (LRRC27), mRNA [NM_030626]	-1.02846985	0.76683422
AI613259	AI613259 ty35c04.x1 NCI_CGAP_Ut2 cDNA clone IMAGE:2281062 3' similar to gb:X69391 60S RIBOSOMAL PROTEIN L6 (HUMAN);, mRNA sequence [AI613259]	-1.02845482	0.86235863
NM_016644	mesenchymal stem cell protein DSC54 (LOC51334), mRNA [NM_016644]	-1.02570031	0.23123009
NM_018152	chromosome 20 open reading frame 12 (C20orf12), mRNA [NM_018152]	-1.02269889	0.66975925
AK098124	cDNA FLJ40805 fis, clone TRACH2009060. [AK098124]	-1.0162032	0.34021808
NM_00101800	tropomyosin 1 (alpha) (TPM1), transcript variant 3, mRNA [NM_001018004]	-1.01163078	0.36314522
NM_014902	discs, large (Drosophila) homolog-associated protein 4 (DLGAP4), transcript variant 1, mRNA [NM_014902]	-1.00698201	0.25677623
NM_173849	goosecoid (GSC), mRNA [NM_173849]	-1.00292735	0.48778374
NM_007023	Rap guanine nucleotide exchange factor (GEF) 4 (RAPGEF4), mRNA [NM_007023]	-1.00072147	0.67852578
NM_207352	cytochrome P450, family 4, subfamily V, polypeptide 2 (CYP4V2), mRNA [NM_207352]	-1.00023337	0.48353078
NM_006988	ADAM metallopeptidase with thrombospondin type 1 motif, 1 (ADAMTS1), mRNA [NM_006988]	-0.99739774	0.63544306
NM_025249	KIAA1683 (KIAA1683), mRNA [NM_025249]	-0.99637424	0.01537613
NM_00100200	guanosine monophosphate reductase 2 (GMPR2), transcript variant 2, mRNA [NM_001002000]	-0.99460288	0.39180031
NM_005567	lectin, galactoside-binding, soluble, 3 binding protein (LGALS3BP), mRNA [NM_005567]	-0.99329423	0.40796211
BX101252	BX101252 NCI_CGAP_Lu24 cDNA clone IMAGp998I115625, mRNA sequence [BX101252]	-0.99300642	0.34252131

NM_001900	cystatin D (CST5), mRNA [NM_001900]	-0.99163284	0.17297881
NM_199357	Rho GTPase activating protein 11A (ARHGAP11A), transcript variant 2, mRNA [NM_199357]	-0.99086706	0.68807672
NM_017414	ubiquitin specific peptidase 18 (USP18), mRNA [NM_017414]	-0.98558866	0.32803771
NM_022755	inositol 1,3,4,5,6-pentakisphosphate 2-kinase (IPPK), mRNA [NM_022755]	-0.98497494	0.679972
AF011794	cell cycle progression restoration 8 protein (CPR8) mRNA, complete cds. [AF011794]	-0.98413423	0.21576958
THC2434479	DD19_HUMAN (Q9UMR2) ATP-dependent RNA helicase DDX19 (DEAD-box protein 19) (DEAD-box RNA helicase DEAD5), partial (90%) [THC2434479]	-0.98167141	0.47040156
NM_018046	angiogenic factor with G patch and FHA domains 1 (AGGF1), mRNA [NM_018046]	-0.97479344	0.2116853
NM_001556	inhibitor of kappa light polypeptide gene enhancer in B-cells, kinase beta (IKBKB), mRNA [NM_001556]	-0.97471689	0.38094701
NM_033204	zinc finger protein 101 (ZNF101), mRNA [NM_033204]	-0.97403624	0.29604272
NM_004994	matrix metallopeptidase 9 (gelatinase B, 92kDa gelatinase, 92kDa type IV collagenase) (MMP9), mRNA [NM_004994]	-0.97337076	0.19748181
NM_022074	family with sequence similarity 111, member A (FAM111A), transcript variant 1, mRNA [NM_022074]	-0.97301445	0.2962427
NM_003149	SH3 and cysteine rich domain (STAC), mRNA [NM_003149]	-0.9710385	0.62844492
NR_002770	deiodinase, iodothyronine, type III opposite strand (DIO3OS) on chromosome 14 [NR_002770]	-0.97049179	0.3962586
THC2409569	Unknown	-0.96870028	0.43604365
THC2315330	Unknown	-0.96841854	0.66709012
NM_024713	chromosome 15 open reading frame 29 (C15orf29), mRNA [NM_024713]	-0.96571715	0.63542593
AV707592	AV707592 ADB cDNA clone ADBAJA03 5', mRNA sequence [AV707592]	-0.96525323	0.67898137
NM_005466	mediator of RNA polymerase II transcription, subunit 6 homolog (S. cerevisiae) (MED6), mRNA [NM_005466]	-0.96345379	0.35919119
NM_015216	Histidine acid phosphatase domain containing 1 (HISPPD1), mRNA [NM_015216]	-0.95495772	0.3769853
THC2276504	Q9UID7 (Q9UID7) CYR61 protein, partial (87%) [THC2276504]	-0.95215532	0.23896948
THC2312637	moxR3 {Mycobacterium smegmatis str. MC2 155;} , partial (3%) [THC2312637]	-0.9496045	0.59566853
BE535679	601060331F1 NIH_MGC_10 cDNA clone IMAGE:3446983 5', mRNA sequence [BE535679]	-0.94882659	0.50447859
NM_145055	chromosome 18 open reading frame 25 (C18orf25), transcript variant 1, mRNA [NM_145055]	-0.94843119	0.35123361
NM_173502	protease, serine, 36 (PRSS36), mRNA [NM_173502]	-0.94805879	0.00556734
NM_021238	family with sequence similarity 60, member A (FAM60A), mRNA [NM_021238]	-0.94531008	0.0581943
ENST00000377	mRNA for KIAA1165 protein, partial cds. [AB032991]	-0.93793299	0.52430625
NM_004290	ring finger protein 14 (RNF14), transcript variant 1, mRNA [NM_004290]	-0.93296584	0.39438959
NM_005065	sel-1 suppressor of lin-12-like (C. elegans) (SEL1L), mRNA [NM_005065]	-0.93151997	0.46662236
AL136588	mRNA; cDNA DKFZp761D112 (from clone DKFZp761D112). [AL136588]	-0.92805435	0.01118499
CR590183	full-length cDNA clone CS0DF036YC04 of Fetal brain of (human). [CR590183]	-0.9276261	0.4974431
NM_198046	zinc finger, DHHC-type containing 16 (ZDHHC16), transcript variant 5, mRNA [NM_198046]	-0.92645194	0.2975294
ENST00000310	PREDICTED: similar to ribosomal protein L21 isoform 1 (LOC388532), mRNA [XM_371160]	-0.924062	0.30737756
AL136922	mRNA; cDNA DKFZp586J151 (from clone DKFZp586J151). [AL136922]	-0.92322687	0.08220712
NM_015291	DnaJ (Hsp40) homolog, subfamily C, member 16 (DNAJC16), mRNA [NM_015291]	-0.9220481	0.14003022
NM_006007	zinc finger, AN1-type domain 5 (ZFAND5), mRNA [NM_006007]	-0.91966034	0.13034356
NM_152463	essential meiotic endonuclease 1 homolog 1 (S. pombe) (EME1), mRNA [NM_152463]	-0.91943784	0.47370865
A_24_P118382	Unknown	-0.91917527	0.08122243
NM_016357	LIM domain and actin binding 1 (LIMA1), mRNA [NM_016357]	-0.91895249	0.24354605

AK126887	cDNA FLJ44939 fis, clone BRAMY3018754, weakly similar to Mus musculus junction-mediating and regulatory protein (Jmy-pending). [AK126887]	-0.91182999	0.26317663
NM_003528	histone 2, H2be (HIST2H2BE), mRNA [NM_003528]	-0.91002698	0.25759062
THC2320516	Unknown	-0.90902453	0.53142113
NM_006364	Sec23 homolog A (S. cerevisiae) (SEC23A), mRNA [NM_006364]	-0.90889315	0.43216781
CA429641	UI-H-FH1-bfo-d-01-0-UI.s1 NCI_CGAP_FH1 cDNA clone UI-H-FH1-bfo-d-01-0-UI 3', mRNA sequence [CA429641]	-0.90885179	0.17008294
NM_018334	leucine rich repeat neuronal 3 (LRRN3), mRNA [NM_018334]	-0.90574066	0.65130337
NM_018448	cullin-associated and neddylation-dissociated 1 (CAND1), mRNA [NM_018448]	-0.90553987	0.15075729
NM_020937	Fanconi anemia, complementation group M (FANCM), mRNA [NM_020937]	-0.90040567	0.04176206
NM_004994	matrix metallopeptidase 9 (gelatinase B, 92kDa gelatinase, 92kDa type IV collagenase) (MMP9), mRNA [NM_004994]	-0.90001415	0.02079956
W45382	W45382 zc80e10.s1 Pancreatic Islet cDNA clone IMAGE:328650 3' similar to gb:D13748 EUKARYOTIC INITIATION FACTOR 4A-I (HUMAN);, mRNA sequence [W45382]	-0.89942535	0.31808033
NM_018372	chromosome 1 open reading frame 103 (C1orf103), transcript variant 1, mRNA [NM_018372]	-0.89917944	0.526174
NM_005245	FAT tumor suppressor homolog 1 (Drosophila) (FAT), mRNA [NM_005245]	-0.89788571	0.49712848
AL136578	mRNA; cDNA DKFZp761C0824 (from clone DKFZp761C0824). [AL136578]	-0.89784262	0.09387078
NM_012124	cysteine and histidine-rich domain (CHORD)-containing 1 (CHORDC1), mRNA [NM_012124]	-0.89608861	0.32939622
NM_000956	prostaglandin E receptor 2 (subtype EP2), 53kDa (PTGER2), mRNA [NM_000956]	-0.89416261	0.23578937
ENST00000371:	Unknown	-0.88885785	0.43120842
A_32_P156549	Unknown	-0.88772104	0.24696651
NM_032437	KIAA1799 protein (KIAA1799), mRNA [NM_032437]	-0.88771479	0.54483394
NM_152330	FERM domain containing 6 (FRMD6), transcript variant 2, mRNA [NM_152330]	-0.88638508	0.10956092
BX107836	BX107836 Soares_fetal_heart_NbHH19W cDNA clone IMAGp998I17743, mRNA sequence [BX107836]	-0.88564062	0.19318971
NM_015534	zinc finger, ZZ-type containing 3 (ZZZ3), mRNA [NM_015534]	-0.88489714	0.02951726
NM_00100394!	aminolevulinate, delta-, dehydratase (ALAD), transcript variant 1, mRNA [NM_001003945]	-0.88479892	0.32480307
NM_032373	polycomb group ring finger 5 (PCGF5), mRNA [NM_032373]	-0.88270086	0.22719988
NM_080422	protein tyrosine phosphatase, non-receptor type 2 (PTPN2), transcript variant 2, mRNA [NM_080422]	-0.88191989	0.15454804
THC2295157	Unknown	-0.8812616	0.41979913
THC2455353	AA242987 zr66a03.s1 Soares_NhHMPu_S1 cDNA clone IMAGE:668332 3', mRNA sequence [AA242987]	-0.87827554	0.47857776
CR620977	full-length cDNA clone CS0CAP004YK15 of Thymus of (human). [CR620977]	-0.8772396	0.06136155
A_32_P145159	Unknown	-0.87517301	0.0883494
NM_006100	ST3 beta-galactoside alpha-2,3-sialyltransferase 6 (ST3GAL6), mRNA [NM_006100]	-0.87514508	0.26056044
ENST00000324(	slingshot homolog 2 (Drosophila), mRNA (cDNA clone IMAGE:4101583), complete cds. [BC011636]	-0.87478929	0.42348244
NM_032632	poly(A) polymerase alpha (PAPOLA), mRNA [NM_032632]	-0.87341115	0.41258984
THC2378401	Unknown	-0.87010674	0.15190126
NM_174887	intraflagellar transport 20 homolog (Chlamydomonas) (IFT20), mRNA [NM_174887]	-0.87003205	0.57506134
NM_030753	wingless-type MMTV integration site family, member 3 (WNT3), mRNA [NM_030753]	-0.86774454	0.49964454
NM_058197	cyclin-dependent kinase inhibitor 2A (melanoma, p16, inhibits CDK4) (CDKN2A), transcript variant 3, mRNA [NM_058197]	-0.8673249	0.15321571
THC2381535	Unknown	-0.86693341	0.50088586

AK056698	cDNA FLJ32136 fis, clone PEBLM2000395, moderately similar to ZINC FINGER PROTEIN 165. [AK056698]	-0.86577053	0.49942552
THC2275676	Unknown	-0.86575399	0.01556393
NM_017847	chromosome 1 open reading frame 27 (C1orf27), mRNA [NM_017847]	-0.86501466	0.17245459
AF086427	full length insert cDNA clone ZD79D11. [AF086427]	-0.86191216	0.25860782
ENST00000355(	mRNA for KIAA1473 protein, partial cds. [AB040906]	-0.86034999	0.47958947
NM_020951	zinc finger protein 529 (ZNF529), mRNA [NM_020951]	-0.85896871	0.19499912
THC2315164	Unknown	-0.85772907	0.61355571
NM_006859	lipoic acid synthetase (LIAS), nuclear gene encoding mitochondrial protein, transcript variant 1, mRNA [NM_006859]	-0.85639695	0.39749212
NM_173694	ATPase, Class VI, type 11C (ATP11C), transcript variant 1, mRNA [NM_173694]	-0.85600513	0.23942884
NM_152896	ubiquitin-like, containing PHD and RING finger domains, 2 (UHRF2), transcript variant 2, mRNA [NM_152896]	-0.85460721	0.27626394
NM_002210	integrin, alpha V (vitronectin receptor, alpha polypeptide, antigen CD51) (ITGAV), mRNA [NM_002210]	-0.85305822	0.28433147
A_24_P358390	Unknown	-0.85098246	0.32679409
NM_153340	ataxin 7-like 2 (ATXN7L2), mRNA [NM_153340]	-0.84728341	0.36025452
NM_134442	cAMP responsive element binding protein 1 (CREB1), transcript variant B, mRNA [NM_134442]	-0.84593626	0.3650917
NM_025125	chromosome 10 open reading frame 57 (C10orf57), mRNA [NM_025125]	-0.84474407	0.49986843
NM_031292	pseudouridylate synthase 7 homolog (S. cerevisiae)-like (PUS7L), mRNA [NM_031292]	-0.84339168	0.44901222
NM_003847	peroxisomal biogenesis factor 11A (PEX11A), mRNA [NM_003847]	-0.84316217	0.59157916
BC030084	cDNA clone IMAGE:4791887. [BC030084]	-0.84174418	0.12211381
NM_013448	bromodomain adjacent to zinc finger domain, 1A (BAZ1A), transcript variant 1, mRNA [NM_013448]	-0.84087505	0.37243672
NM_007159	sarcolemma associated protein (SLMAP), mRNA [NM_007159]	-0.84063514	0.00347425
NM_015690	serine/threonine kinase 36 (fused homolog, Drosophila) (STK36), mRNA [NM_015690]	-0.83816255	0.11083224
BC040982	cDNA clone IMAGE:4798675. [BC040982]	-0.83755715	0.16905245
NM_000195	Hermansky-Pudlak syndrome 1 (HPS1), transcript variant 1, mRNA [NM_000195]	-0.83458922	0.48103271
AF168717	x 009 protein mRNA, complete cds. [AF168717]	-0.83435331	0.37252526
NM_013448	bromodomain adjacent to zinc finger domain, 1A (BAZ1A), transcript variant 1, mRNA [NM_013448]	-0.83346116	0.33214142
AK074776	cDNA FLJ90295 fis, clone NT2RP2000240. [AK074776]	-0.83254513	0.27106503
NM_058197	cyclin-dependent kinase inhibitor 2A (melanoma, p16, inhibits CDK4) (CDKN2A), transcript variant 3, mRNA [NM_058197]	-0.82755722	0.17056111
AK024670	cDNA: FLJ21017 fis, clone CAE05907. [AK024670]	-0.82731506	0.16347329
AB111887	mRNA for KIAA2035 protein. [AB111887]	-0.82702209	0.00207633
NM_002737	protein kinase C, alpha (PRKCA), mRNA [NM_002737]	-0.82687581	0.33045808
NM_145048	chromosome 4 open reading frame 28 (C4orf28), mRNA [NM_145048]	-0.82590171	0.59412041
NM_054023	secretoglobin, family 3A, member 2 (SCGB3A2), mRNA [NM_054023]	-0.82471704	0.27836187
NM_015216	Histidine acid phosphatase domain containing 1 (HISPPD1), mRNA [NM_015216]	-0.82272622	0.01835591
NM_000555	doublecortex; lissencephaly, X-linked (doublecortin) (DCX), transcript variant 1, mRNA [NM_000555]	-0.82265171	0.06227006
BX647761	mRNA; cDNA DKFZp686K2231 (from clone DKFZp686K2231). [BX647761]	-0.82248763	0.55358758
NM_006504	protein tyrosine phosphatase, receptor type, E (PTPRE), transcript variant 1, mRNA [NM_006504]	-0.82219001	0.02846498

NM_178834	layilin (LAYN), mRNA [NM_178834]	-0.82159516	0.34560882
U12206	Human clone pL713 hypothetical protein mRNA, partial cds. [U12206]	-0.82149816	0.15995951
XM_070233	PREDICTED: similar to ribosomal protein L10a (LOC137107), mRNA [XM_070233]	-0.8207984	0.50908425
NM_022725	Fanconi anemia, complementation group F (FANCF), mRNA [NM_022725]	-0.81993719	0.30421262
ENST00000297	PNAS-12 mRNA, partial sequence. [AF274937]	-0.81667469	0.03265039
NM_013448	bromodomain adjacent to zinc finger domain, 1A (BAZ1A), transcript variant 1, mRNA [NM_013448]	-0.8166006	0.22126092
BC051368	cDNA clone IMAGE:3956684, **** WARNING: chimeric clone ****. [BC051368]	-0.81107359	0.02272668
AB040974	mRNA for KIAA1541 protein, partial cds. [AB040974]	-0.81054455	0.0921702
NM_004398	DEAD (Asp-Glu-Ala-Asp) box polypeptide 10 (DDX10), mRNA [NM_004398]	-0.81022981	0.43261707
NM_145294	WD repeat domain 90 (WDR90), mRNA [NM_145294]	-0.80988328	0.35016875
AK055302	cDNA FLJ30740 fis, clone FEBRA2000319. [AK055302]	-0.80983337	0.00758582
NM_015939	CGI-09 protein (CGI-09), mRNA [NM_015939]	-0.808595	0.11092398
NM_152495	cornichon homolog 3 (Drosophila) (CNIH3), mRNA [NM_152495]	-0.80826346	0.11099586
NM_024772	zinc finger, MYM-type 1 (ZMYM1), mRNA [NM_024772]	-0.80812556	0.2126117
NM_144567	angel homolog 2 (Drosophila) (ANGEL2), mRNA [NM_144567]	-0.80380322	0.14273176
THC2406285	Q9FH26 (Q9FH26) Arabidopsis thaliana genomic DNA, chromosome 5, TAC clone:K20J1, partial (3%) [THC2406285]	-0.80372493	0.28144099
NM_032237	hypothetical protein FLJ23356 (FLJ23356), mRNA [NM_032237]	-0.80311212	0.31339643
NM_013448	bromodomain adjacent to zinc finger domain, 1A (BAZ1A), transcript variant 1, mRNA [NM_013448]	-0.80272208	0.26229141
ENST00000327	cDNA FLJ43953 fis, clone TESTI4015477. [AK125941]	-0.80113296	0.55787436
ENST00000354	cDNA FLJ42934 fis, clone BRSSN2014112. [AK124924]	-0.80049996	0.28194033
AL136719	mRNA; cDNA DKFZp566G0346 (from clone DKFZp566G0346). [AL136719]	-0.7989317	0.09098955
NM_018341	chromosome 6 open reading frame 70 (C6orf70), mRNA [NM_018341]	-0.7974941	0.54190508
NM_015879	ST8 alpha-N-acetyl-neuraminide alpha-2,8-sialyltransferase 3 (ST8SIA3), mRNA [NM_015879]	-0.79592264	0.00638448
NM_006898	homeobox D3 (HOXD3), mRNA [NM_006898]	-0.79537639	0.00714301
NM_174916	ubiquitin protein ligase E3 component n-recognin 1 (UBR1), mRNA [NM_174916]	-0.79518412	0.36054489
CR601260	full-length cDNA clone CS0DM001YA20 of Fetal liver of (human). [CR601260]	-0.79499411	0.51639854
NM_000675	adenosine A2a receptor (ADORA2A), mRNA [NM_000675]	-0.79289747	0.32087133
AB013462	mRNA for Fzr1, complete cds. [AB013462]	-0.79120289	0.15617177
NM_006241	protein phosphatase 1, regulatory (inhibitor) subunit 2 (PPP1R2), mRNA [NM_006241]	-0.79036824	0.30127304
NM_001315	mitogen-activated protein kinase 14 (MAPK14), transcript variant 1, mRNA [NM_001315]	-0.78845833	0.1445481
NM_004380	CREB binding protein (Rubinstein-Taybi syndrome) (CREBBP), mRNA [NM_004380]	-0.78830822	0.4905431
AB058761	mRNA for KIAA1858 protein, partial cds. [AB058761]	-0.78725361	0.22665932
NM_005524	hairy and enhancer of split 1, (Drosophila) (HES1), mRNA [NM_005524]	-0.78609267	0.17885344
CF528315	CF528315 UI-1-BC0-aen-f-04-0-UI.s1 NCI_CGAP_Pl1 cDNA clone UI-1-BC0-aen-f-04-0-UI 3', mRNA sequence [CF528315]	-0.78420716	0.36302952
NM_147147	blood vessel epicardial substance (BVES), transcript variant B, mRNA [NM_147147]	-0.78297254	0.29805859
ENST00000316	small nuclear RNA activating complex, polypeptide 5, 19kDa, mRNA (cDNA clone MGC:22700 IMAGE:3997264), complete cds. [BC014315]	-0.78156775	0.34174169
NM_020347	leucine zipper transcription factor-like 1 (LZTFL1), mRNA [NM_020347]	-0.78149722	0.21886121
NM_181787	dpy-19-like 4 (C. elegans) (DPY19L4), mRNA [NM_181787]	-0.78118728	0.25149271
NM_002048	growth arrest-specific 1 (GAS1), mRNA [NM_002048]	-0.7808061	0.27653707
A_32_P171232	Unknown	-0.77935848	0.29715716

AF168717	x 009 protein mRNA, complete cds. [AF168717]	-0.77916896	0.37776736
BC000986	cDNA clone IMAGE:3446313, complete cds. [BC000986]	-0.77898714	0.47148523
NM_003566	early endosome antigen 1, 162kD (EEA1), mRNA [NM_003566]	-0.77622488	0.06414559
NM_002210	integrin, alpha V (vitronectin receptor, alpha polypeptide, antigen CD51) (ITGAV), mRNA [NM_002210]	-0.77395085	0.08896371
NM_002223	inositol 1,4,5-triphosphate receptor, type 2 (ITPR2), mRNA [NM_002223]	-0.77376521	0.24444231
NM_004360	cadherin 1, type 1, E-cadherin (epithelial) (CDH1), mRNA [NM_004360]	-0.77302692	0.52075667
NM_012301	membrane associated guanylate kinase, WW and PDZ domain containing 2 (MAGI2), mRNA [NM_012301]	-0.77210521	0.25846655
NM_144498	oxysterol binding protein-like 2 (OSBPL2), transcript variant 2, mRNA [NM_144498]	-0.77207721	0.01491119
NM_006470	tripartite motif-containing 16 (TRIM16), mRNA [NM_006470]	-0.77131723	0.15427142
NM_016122	coiled-coil domain containing 41 (CCDC41), transcript variant 1, mRNA [NM_016122]	-0.77107446	0.23096042
NM_002341	lymphotoxin beta (TNF superfamily, member 3) (LTB), transcript variant 1, mRNA [NM_002341]	-0.76938614	0.05479131
NM_002706	protein phosphatase 1B (formerly 2C), magnesium-dependent, beta isoform (PPM1B), transcript variant 1, mRNA [NM_002706]	-0.7676014	0.14989452
NM_003893	LIM domain binding 1 (LDB1), mRNA [NM_003893]	-0.7671608	0.49085337
BC000853	chromosome 2 open reading frame 3, mRNA (cDNA clone IMAGE:3459069), complete cds. [BC000853]	-0.7651381	0.20017888
Z74615	H.sapiens mRNA for prepro-alpha1(I) collagen. [Z74615]	-0.76324521	0.27784987
CB240827	UI-CF-FN0-afw-h-15-0-UI.s1 UI-CF-FN0 cDNA clone UI-CF-FN0-afw-h-15-0-UI 3', mRNA sequence [CB240827]	-0.7613321	0.2712402
NM_00100285[illegible]	annexin A2 (ANXA2), transcript variant 2, mRNA [NM_001002857]	-0.76092044	0.4912345
NM_052818	hypothetical gene CG018 (CG018), mRNA [NM_052818]	-0.75736273	0.32241176
NM_015216	Histidine acid phosphatase domain containing 1 (HISPPD1), mRNA [NM_015216]	-0.7538414	0.27949006
NM_000725	calcium channel, voltage-dependent, beta 3 subunit (CACNB3), mRNA [NM_000725]	-0.75383243	0.10943391
ENST00000321[illegible]	cDNA: FLJ23531 fis, clone LNG06065. [AK027184]	-0.75092666	0.36549415
AF168717	x 009 protein mRNA, complete cds. [AF168717]	-0.75066098	0.3365415
NM_014674	ER degradation enhancer, mannosidase alpha-like 1 (EDEM1), mRNA [NM_014674]	-0.75055586	0.0878831
NM_058197	cyclin-dependent kinase inhibitor 2A (melanoma, p16, inhibits CDK4) (CDKN2A), transcript variant 3, mRNA [NM_058197]	-0.74928103	0.45445414
ENST0000339[illegible]	TBC1 domain family, member 3 pseudogene 2, mRNA (cDNA clone MGC:64921 IMAGE:5744726), complete cds. [BC058890]	-0.74859947	0.17488179
BC070091	caspase recruitment domain family, member 9, mRNA (cDNA clone MGC:87491 IMAGE:30343821), complete cds. [BC070091]	-0.74848418	0.12886985
NM_177528	sulfotransferase family, cytosolic, 1A, phenol-preferring, member 2 (SULT1A2), transcript variant 2, mRNA [NM_177528]	-0.74747177	0.20746029
NM_003913	PRP4 pre-mRNA processing factor 4 homolog B (yeast) (PRPF4B), mRNA [NM_003913]	-0.74604111	0.34190827
NM_153329	aldehyde dehydrogenase 16 family, member A1 (ALDH16A1), mRNA [NM_153329]	-0.74557715	0.29271417
CR590573	full-length cDNA clone CS0DI042YD07 of Placenta Cot 25-normalized of (human). [CR590573]	-0.7446311	0.40320642
NM_032141	coiled-coil domain containing 55 (CCDC55), transcript variant 1, mRNA [NM_032141]	-0.7444121	0.43865598
CD518214	AGENCOURT_14375911 NIH_MGC_181 cDNA clone IMAGE:30407414 5', mRNA sequence [CD518214]	-0.74437336	0.39425704
NM_023934	FUN14 domain containing 2 (FUNDC2), mRNA [NM_023934]	-0.7434071	0.15095033
A_24_P289573	Unknown	-0.74260443	0.30851373

AB082533	mRNA for KIAA2002 protein. [AB082533]	-0.74247973	0.36994811
NM_014602	phosphoinositide-3-kinase, regulatory subunit 4, p150 (PIK3R4), mRNA [NM_014602]	-0.73934987	0.40549699
NM_173827	COX18 cytochrome c oxidase assembly homolog (S. cerevisiae) (COX18), mRNA [NM_173827]	-0.73840829	0.08964552
NM_182501	MTERF domain containing 2 (MTERFD2), mRNA [NM_182501]	-0.73806889	0.25645414
NM_006336	zyg-11 homolog B (C. elegans)-like (ZYG11BL), mRNA [NM_006336]	-0.73758492	0.12320792
NM_003082	small nuclear RNA activating complex, polypeptide 1, 43kDa (SNAPC1), mRNA [NM_003082]	-0.73705222	0.00351812
NM_003412	Zic family member 1 (odd-paired homolog, Drosophila) (ZIC1), mRNA [NM_003412]	-0.73594893	0.2001743
NM_025194	inositol 1,4,5-trisphosphate 3-kinase C (ITPKC), mRNA [NM_025194]	-0.73582555	0.0511429
NM_018200	high-mobility group 20A (HMG20A), mRNA [NM_018200]	-0.73492905	0.03715212
NM_017590	zinc finger CCCH-type containing 7B (ZC3H7B), mRNA [NM_017590]	-0.73481179	0.38642875
NM_006358	solute carrier family 25 (mitochondrial carrier; peroxisomal membrane protein, 34kDa), member 17 (SLC25A17), nuclear gene encoding mitochondrial protein, mRNA [NM_006358]	-0.73420744	0.12535626
AF495725	FP15737 mRNA, complete cds. [AF495725]	-0.73408243	0.41598746
NM_004994	matrix metallopeptidase 9 (gelatinase B, 92kDa gelatinase, 92kDa type IV collagenase) (MMP9), mRNA [NM_004994]	-0.73384684	0.46975353
NM_002686	phenylethanolamine N-methyltransferase (PNMT), mRNA [NM_002686]	-0.73375331	0.11879377
NM_003469	secretogranin II (chromogranin C) (SCG2), mRNA [NM_003469]	-0.73323014	0.05210123
NM_013448	bromodomain adjacent to zinc finger domain, 1A (BAZ1A), transcript variant 1, mRNA [NM_013448]	-0.73318311	0.16604321
NM_004787	slit homolog 2 (Drosophila) (SLIT2), mRNA [NM_004787]	-0.73251932	0.00510199
NM_020317	chromosome 1 open reading frame 63 (C1orf63), transcript variant 2, mRNA [NM_020317]	-0.73157327	0.090221
NM_194248	otoferlin (OTOF), transcript variant 1, mRNA [NM_194248]	-0.73135807	0.33699795
NM_014434	NADPH dependent diflavin oxidoreductase 1 (NDOR1), mRNA [NM_014434]	-0.73092277	0.04463689
NM_00100412	tumor suppressor candidate 1 (TUSC1), mRNA [NM_001004125]	-0.73073126	0.20679033
NM_004064	cyclin-dependent kinase inhibitor 1B (p27, Kip1) (CDKN1B), mRNA [NM_004064]	-0.72961004	0.27298359
NM_003427	zinc finger protein 76 (expressed in testis) (ZNF76), mRNA [NM_003427]	-0.7288565	0.0545347
NM_005902	SMAD, mothers against DPP homolog 3 (Drosophila) (SMAD3), mRNA [NM_005902]	-0.72810332	0.06649141
NR_002211	Meis1 homolog 3 (mouse) pseudogene 1 (MEIS3P1) on chromosome 17 [NR_002211]	-0.72804374	0.09444822
AK090474	mRNA for FLJ00396 protein. [AK090474]	-0.72804298	0.12480523
BQ010172	BQ010172 UI-H-ED0-axz-j-15-0-UI.s1 NCI_CGAP_ED0 cDNA clone IMAGE:5835758 3', mRNA sequence [BQ010172]	-0.72754168	0.10546949
NM_020825	Crm, cramped-like (Drosophila) (CRAMP1L), mRNA [NM_020825]	-0.72692083	0.42398046
NM_004064	cyclin-dependent kinase inhibitor 1B (p27, Kip1) (CDKN1B), mRNA [NM_004064]	-0.72498926	0.05070018
BC053858	zinc finger protein 550, mRNA (cDNA clone MGC:61593 IMAGE:6044705), complete cds. [BC053858]	-0.724391	0.07903916
ENST0000026	mRNA for KIAA0562 protein, partial cds. [AB011134]	-0.72426654	0.12312838
NM_152402	translocation associated membrane protein 1-like 1 (TRAM1L1), mRNA [NM_152402]	-0.72317776	0.20994491
NM_004849	ATG5 autophagy related 5 homolog (S. cerevisiae) (ATG5), mRNA [NM_004849]	-0.72200051	0.38726812
AL035301	H.sapiens gene from PAC 106H8. [AL035301]	-0.72151911	0.24154823
AY358619	clone DNA107443 AGLW2560 (UNQ2560) mRNA, complete cds. [AY358619]	-0.72146356	0.31663263
NM_015534	zinc finger, ZZ-type containing 3 (ZZZ3), mRNA [NM_015534]	-0.7194587	0.27824215
NM_015073	signal-induced proliferation-associated 1 like 3 (SIPA1L3), mRNA [NM_015073]	-0.71851972	0.11224696
NM_024092	transmembrane protein 109 (TMEM109), mRNA [NM_024092]	-0.71664252	0.31422753
NM_153261	chromosome 16 open reading frame 69 (C16orf69), mRNA [NM_153261]	-0.71655829	0.16047971

NM_002470	myosin, heavy polypeptide 3, skeletal muscle, embryonic (MYH3), mRNA [NM_002470]	-0.71521794	0.27841213
NM_004330	BCL2/adenovirus E1B 19kDa interacting protein 2 (BNIP2), mRNA [NM_004330]	-0.71516669	0.10217202
NM_003567	breast cancer anti-estrogen resistance 3 (BCAR3), mRNA [NM_003567]	-0.71444354	0.23673354
BC030112	cDNA clone IMAGE:4799578. [BC030112]	-0.71354562	0.32279378
NM_033121	ankyrin repeat domain 13A (ANKRD13A), mRNA [NM_033121]	-0.71334047	0.09893912
ENST00000357	cDNA FLJ13094 fis, clone NT2RP3002163. [AK023156]	-0.71231287	0.06051705
NM_018651	zinc finger protein 167 (ZNF167), transcript variant 1, mRNA [NM_018651]	-0.71026993	0.10504401
NM_173079	RUN domain containing 1 (RUNDC1), mRNA [NM_173079]	-0.71019925	0.02388125
NM_015017	ubiquitin specific peptidase 33 (USP33), transcript variant 1, mRNA [NM_015017]	-0.70982412	0.01280961
BC024007	chitobiase, di-N-acetyl-, mRNA (cDNA clone IMAGE:4823479), complete cds. [BC024007]	-0.70771853	0.15779017
ENST00000340	mRNA for FLJ00339 protein. [AK131103]	-0.70528341	0.05719772
NM_001419	ELAV (embryonic lethal, abnormal vision, Drosophila)-like 1 (Hu antigen R) (ELAVL1), mRNA [NM_001419]	-0.70507378	0.08088583
NM_018211	ribonucleoprotein, PTB-binding 2 (RAVER2), mRNA [NM_018211]	-0.70477447	0.10275739
NM_022574	PERQ amino acid rich, with GYF domain 1 (PERQ1), mRNA [NM_022574]	-0.70428049	0.2262126
NM_144778	muscleblind-like 2 (Drosophila) (MBNL2), transcript variant 1, mRNA [NM_144778]	-0.70336091	0.27125929
NM_00101242	YY1 associated factor 2 (YAF2), transcript variant 2, mRNA [NM_001012424]	-0.70164001	0.33243251
NM_020147	THAP domain containing 10 (THAP10), mRNA [NM_020147]	-0.70138756	0.01764481
NM_015216	Histidine acid phosphatase domain containing 1 (HISPPD1), mRNA [NM_015216]	-0.69977629	0.1539316
NM_058197	cyclin-dependent kinase inhibitor 2A (melanoma, p16, inhibits CDK4) (CDKN2A), transcript variant 3, mRNA [NM_058197]	-0.69923124	0.40453713
NM_178520	transmembrane protein 105 (TMEM105), mRNA [NM_178520]	-0.69875654	0.10763011
NM_052813	caspase recruitment domain family, member 9 (CARD9), mRNA [NM_052813]	-0.69783621	0.06458689
NM_021213	phosphatidylcholine transfer protein (PCTP), mRNA [NM_021213]	-0.69750235	0.01602846
AL136717	mRNA; cDNA DKFZp566D1346 (from clone DKFZp566D1346). [AL136717]	-0.69704268	0.14364955
NM_033450	ATP-binding cassette, sub-family C (CFTR/MRP), member 10 (ABCC10), mRNA [NM_033450]	-0.69641877	0.1289828
NM_004064	cyclin-dependent kinase inhibitor 1B (p27, Kip1) (CDKN1B), mRNA [NM_004064]	-0.69588394	0.17454577
ENST00000304	mRNA; cDNA DKFZp547E107 (from clone DKFZp547E107). [AL390132]	-0.69526087	0.16499385
NM_001109	ADAM metallopeptidase domain 8 (ADAM8), mRNA [NM_001109]	-0.69510686	0.22993725
NM_025103	intraflagellar transport 74 homolog (Chlamydomonas) (IFT74), mRNA [NM_025103]	-0.69372482	0.03628384
NM_133646	sterile alpha motif and leucine zipper containing kinase AZK (ZAK), transcript variant 2, mRNA [NM_133646]	-0.69346919	0.24369984
NM_021999	integral membrane protein 2B (ITM2B), mRNA [NM_021999]	-0.69337343	0.01353876
THC2340670	Unknown	-0.69276371	0.05773493
ENST00000372	similar to RIKEN cDNA 2310039H08, mRNA (cDNA clone MGC:75222 IMAGE:4364495), complete cds. [BC060325]	-0.6926465	0.10213971
NM_206910	glucuronidase, beta-like 2 (GUSBL2), transcript variant 3, mRNA [NM_206910]	-0.69194006	0.02307107
AK025431	cDNA: FLJ21778 fis, clone HEP00201. [AK025431]	-0.69164066	0.10518991
AL562818	AL562818 NEUROBLASTOMA COT 25-NORMALIZED cDNA clone CS0DC017YH14 3-PRIME, mRNA sequence [AL562818]	-0.69056367	0.12637247
NM_004380	CREB binding protein (Rubinstein-Taybi syndrome) (CREBBP), mRNA [NM_004380]	-0.6892564	0.33058003
A_24_P471099	Unknown	-0.68909443	0.39499475
NM_020750	exportin 5 (XPO5), mRNA [NM_020750]	-0.68781575	0.23619263
NM_014662	DEP domain containing 5 (DEPDC5), transcript variant 1, mRNA [NM_014662]	-0.68778501	0.10040466
NM_013448	bromodomain adjacent to zinc finger domain, 1A (BAZ1A), transcript variant 1, mRNA [NM_013448]	-0.68702756	0.0348401

NM_002890	RAS p21 protein activator (GTPase activating protein) 1 (RASA1), transcript variant 1, mRNA [NM_002890]	-0.6862901	0.23550855
NM_022036	G protein-coupled receptor, family C, group 5, member C (GPRC5C), transcript variant 1, mRNA [NM_022036]	-0.6861458	0.29324902
NM_002210	integrin, alpha V (vitronectin receptor, alpha polypeptide, antigen CD51) (ITGAV), mRNA [NM_002210]	-0.68612903	0.16156866
NM_005999	translin-associated factor X (TSNAX), mRNA [NM_005999]	-0.68577097	0.29459456
NM_000582	secreted phosphoprotein 1 (osteopontin, bone sialoprotein I, early T-lymphocyte activation 1) (SPP1), transcript variant 2, mRNA [NM_000582]	-0.68393832	0.40113357
CR592968	full-length cDNA clone CL0BB005ZA03 of Neuroblastoma of (human). [CR592968]	-0.6835162	0.33341612
AK023447	cDNA FLJ13385 fis, clone PLACE1001088. [AK023447]	-0.68293811	0.07074499
NM_003353	urocortin (UCN), mRNA [NM_003353]	-0.68025362	0.2084642
AK021668	cDNA FLJ11606 fis, clone HEMBA1003942. [AK021668]	-0.67981073	0.24882911
NM_199045	hypothetical LOC440248 (LOC440248), mRNA [NM_199045]	-0.67772893	0.01068581
NM_139076	coiled-coil domain containing 98 (CCDC98), mRNA [NM_139076]	-0.67771019	0.16948226
ENST0000369	clone DKFZp564A057 AG02 mRNA, partial cds. [AF419616]	-0.67649361	0.28339308
ENST0000373(	Unknown	-0.67584511	0.18800753
NM_006159	NEL-like 2 (chicken) (NELL2), mRNA [NM_006159]	-0.67491058	0.19976598
BC036529	enhancer of polycomb homolog 1 (Drosophila), mRNA (cDNA clone MGC:33656 IMAGE:4828057), complete cds. [BC036529]	-0.67440642	0.2586349
AB210041	mRNA for MPDZ variant protein, partial cds, clone: pf00482. [AB210041]	-0.67438453	0.10809353
NM_018674	amiloride-sensitive cation channel 4, pituitary (ACCN4), transcript variant 1, mRNA [NM_018674]	-0.67371332	0.25064136
NM_020784	KIAA1344 (KIAA1344), mRNA [NM_020784]	-0.67362526	0.00193766
NM_018257	protein-L-isoaspartate (D-aspartate) O-methyltransferase domain containing 2 (PCMTD2), mRNA [NM_018257]	-0.67347514	0.32638823
NM_033306	caspase 4, apoptosis-related cysteine peptidase (CASP4), transcript variant gamma, mRNA [NM_033306]	-0.67210735	0.09786179
NM_015609	chromosome 1 open reading frame 144 (C1orf144), mRNA [NM_015609]	-0.67178584	0.13657968
NM_014323	zinc finger protein 278 (ZNF278), transcript variant 1, mRNA [NM_014323]	-0.6711797	0.06400178
NM_152282	acid phosphatase-like 2 (ACPL2), transcript variant 1, mRNA [NM_152282]	-0.67019008	0.23800876
NM_170606	myeloid/lymphoid or mixed-lineage leukemia 3 (MLL3), transcript variant 2, mRNA [NM_170606]	-0.66854101	0.33666207
BI520212	603071460F1 NIH_MGC_119 cDNA clone IMAGE:5163326 5', mRNA sequence [BI520212]	-0.66835305	0.17394721
AF289596	clone pp7882 unknown mRNA. [AF289596]	-0.6677022	0.25941702
NM_005359	SMAD, mothers against DPP homolog 4 (Drosophila) (SMAD4), mRNA [NM_005359]	-0.66718973	0.11514247
NM_152686	DnaJ (Hsp40) homolog, subfamily C, member 18 (DNAJC18), mRNA [NM_152686]	-0.66417527	0.11578117
AL050376	mRNA; cDNA DKFZp586J101 (from clone DKFZp586J101). [AL050376]	-0.6639517	0.29265984
AK027341	cDNA FLJ14435 fis, clone HEMBA1007085. [AK027341]	-0.66346734	0.10439586
NM_004064	cyclin-dependent kinase inhibitor 1B (p27, Kip1) (CDKN1B), mRNA [NM_004064]	-0.66271031	0.24691066
NM_017423	UDP-N-acetyl-alpha-D-galactosamine:polypeptide N-acetylgalactosaminyltransferase 7 (GalNAc-T7) (GALNT7), mRNA [NM_017423]	-0.66244438	0.31421101
NM_014683	unc-51-like kinase 2 (C. elegans) (ULK2), mRNA [NM_014683]	-0.66119586	0.25106195
NM_015535	DNA polymerase-transactivated protein 6 (DNAPTP6), mRNA [NM_015535]	-0.66106608	0.04271391
NM_014773	KIAA0141 (KIAA0141), mRNA [NM_014773]	-0.65896432	0.16963147
NM_020863	zinc finger protein 406 (ZNF406), transcript variant ZFAT-1, mRNA [NM_020863]	-0.65867585	0.3100987

NM_024611	NMDA receptor regulated 2 (NARG2), transcript variant 1, mRNA [NM_024611]	-0.65720478	0.28079875
NM_018165	polybromo 1 (PB1), transcript variant 1, mRNA [NM_018165]	-0.65593465	0.21856002
BC041893	tetratricopeptide repeat domain 17, mRNA (cDNA clone IMAGE:5298645), complete cds. [BC041893]	-0.65571918	0.16287285
NM_152996	ST6 (alpha-N-acetyl-neuraminyl-2,3-beta-galactosyl-1,3)-N-acetylgalactosaminide alpha-2,6-sialyltransferase 3 (ST6GALNAC3), mRNA [NM_152996]	-0.6550114	0.18927898
BC038997	chromosome 6 open reading frame 189, mRNA (cDNA clone IMAGE:6059932), partial cds. [BC038997]	-0.65474387	0.20378144
NM_000283	phosphodiesterase 6B, cGMP-specific, rod, beta (congenital stationary night blindness 3, autosomal dominant) (PDE6B), mRNA [NM_000283]	-0.6546972	0.29527825
NM_020774	mindbomb homolog 1 (Drosophila) (MIB1), mRNA [NM_020774]	-0.65447775	0.15738073
NM_003190	TAP binding protein (tapasin) (TAPBP), transcript variant 1, mRNA [NM_003190]	-0.65363361	0.00397129
NM_00102986	chromosome 6 open reading frame 120 (C6orf120), mRNA [NM_001029863]	-0.65325182	0.35446832
NM_018177	Nedd4 binding protein 2 (N4BP2), mRNA [NM_018177]	-0.65304262	0.29129273
NM_022344	chromosome 17 open reading frame 75 (C17orf75), mRNA [NM_022344]	-0.65230148	0.04533866
BC014578	hypothetical gene supported by AK075484; BC014578, mRNA (cDNA clone IMAGE:3950925). [BC014578]	-0.65155052	0.27921319
THC2339926	Unknown	-0.65146244	0.10588669
NM_020117	leucyl-tRNA synthetase (LARS), mRNA [NM_020117]	-0.65117712	0.14194744
ENST0000033	cDNA FLJ30398 fis, clone BRACE2008402, highly similar to steroid receptor RNA activator isoform 3 mRNA. [AK054960]	-0.65086101	0.2214778
ENST0000037	AGENCOURT_10294432 NIH_MGC_126 cDNA clone IMAGE:6567406 5', mRNA sequence [BU537444]	-0.65085397	0.26298268
NM_000801	FK506 binding protein 1A, 12kDa (FKBP1A), transcript variant 12B, mRNA [NM_000801]	-0.65080251	0.0570562
NM_006166	nuclear transcription factor Y, beta (NFYB), mRNA [NM_006166]	-0.64993854	0.01028357
NM_178509	syntaxin binding protein 4 (STXBP4), mRNA [NM_178509]	-0.64842882	0.20328
NM_004870	mannose-P-dolichol utilization defect 1 (MPDU1), mRNA [NM_004870]	-0.64817133	0.08229617
NM_015387	preimplantation protein 3 (PREI3), transcript variant 1, mRNA [NM_015387]	-0.647835	0.25911935
NM_014393	staufen, RNA binding protein, homolog 2 (Drosophila) (STAU2), mRNA [NM_014393]	-0.64780086	0.27732719
AK096229	cDNA FLJ38910 fis, clone NT2NE2006813, weakly similar to CELL SURFACE GLYCOPROTEIN 1 PRECURSOR. [AK096229]	-0.64683924	0.3482862
NM_031454	selenoprotein O (SELO), mRNA [NM_031454]	-0.64646023	0.2403839
ENST0000037	cDNA clone IMAGE:4798227. [BC040655]	-0.64441834	0.18147232
THC2282652	Q9RW53 (Q9RW53) Mg(2+) transport ATPase-related protein, partial (8%) [THC2282652]	-0.644051	0.09789828
NM_014254	transmembrane protein 5 (TMEM5), mRNA [NM_014254]	-0.63928051	0.01890937
NM_003673	titin-cap (telethonin) (TCAP), mRNA [NM_003673]	-0.63911624	0.27773901
BC054050	THO complex 2, mRNA (cDNA clone IMAGE:5556338), partial cds. [BC054050]	-0.63903036	0.24439786
AK092942	cDNA FLJ35623 fis, clone SPLEN2010986. [AK092942]	-0.63882315	0.13047088
BF436529	BF436529 7p14f01.x1 NCI_CGAP_Br22 cDNA clone IMAGE:3645840 3' similar to contains MER22.t1 MER22 repetitive element ;, mRNA sequence [BF436529]	-0.63849957	0.06194417
A_32_P205913	Unknown	-0.63792607	0.24658051
AK023696	cDNA FLJ13634 fis, clone PLACE1011133. [AK023696]	-0.63477323	0.00869936
NM_019104	protein F25965 (F25965), mRNA [NM_019104]	-0.63423649	0.17333039
AB033058	mRNA for KIAA1232 protein, partial cds. [AB033058]	-0.63406462	0.22020975
AB096249	LOH11CR1J gene, loss of heterozygosity, 11, chromosomal region 1 gene J product. [AB096249]	-0.63244432	0.25682748

NM_013437	low density lipoprotein-related protein 12 (LRP12), mRNA [NM_013437]	-0.63184997	0.02667978
NM_018261	exocyst complex component 1 (EXOC1), transcript variant 1, mRNA [NM_018261]	-0.63107237	0.28661026
THC2381210	Q5T2R2 (Q5T2R2) OTTHUMP00000046079, partial (28%) [THC2381210]	-0.62995425	0.25250818
ENST0000031б:	Unknown	-0.62971115	0.17349252
BC013799	Homo sapiens, clone IMAGE:4386363, mRNA. [BC013799]	-0.62908174	0.12051449
NM_012393	phosphoribosylformylglycinamidine synthase (FGAR amidotransferase) (PFAS), mRNA [NM_012393]	-0.62874036	0.07339104
NM_144654	chromosome 9 open reading frame 116 (C9orf116), mRNA [NM_144654]	-0.62761597	0.26190367
NM_016544	Ras-associated protein Rap1 (RBJ), mRNA [NM_016544]	-0.62651051	0.01972119
BQ333643	BQ333643 CM0-MT0028-030600-414-h06 MT0028 cDNA, mRNA sequence [BQ333643]	-0.6257686	0.27841771
NM_138553	B-cell CLL/lymphoma 11A (zinc finger protein) (BCL11A), transcript variant 5, mRNA [NM_138553]	-0.62508111	0.24528563
NM_006630	zinc finger protein 234 (ZNF234), mRNA [NM_006630]	-0.62474881	0.0402271
THC2345392	Unknown	-0.62424964	0.12913026
NM_006948	stress 70 protein chaperone, microsome-associated, 60kDa (STCH), mRNA [NM_006948]	-0.62389645	0.03429873
NM_006716	DBF4 homolog (S. cerevisiae) (DBF4), mRNA [NM_006716]	-0.62387325	0.05953597
BC004565	hypothetical protein MGC12935, mRNA (cDNA clone IMAGE:4309284), partial cds. [BC004565]	-0.62260786	0.24129419
NM_206962	protein arginine methyltransferase 2 (PRMT2), transcript variant 1, mRNA [NM_206962]	-0.62186388	0.20598885
NM_017780	chromodomain helicase DNA binding protein 7 (CHD7), mRNA [NM_017780]	-0.62033933	0.14184996
NM_002210	integrin, alpha V (vitronectin receptor, alpha polypeptide, antigen CD51) (ITGAV), mRNA [NM_002210]	-0.61779809	0.04681648
NM_001331	catenin (cadherin-associated protein), delta 1 (CTNND1), mRNA [NM_001331]	-0.61660815	0.23900529
AL161991	mRNA; cDNA DKFZp761C169 (from clone DKFZp761C169). [AL161991]	-0.61514877	0.19123631
NM_001362	deiodinase, iodothyronine, type III (DIO3), mRNA [NM_001362]	-0.61391129	0.26832872
AK097322	cDNA FLJ40003 fis, clone STOMA2003716. [AK097322]	-0.61353929	0.21464146
NM_014388	chromosome 1 open reading frame 107 (C1orf107), mRNA [NM_014388]	-0.6119753	0.03977274
NM_015216	Histidine acid phosphatase domain containing 1 (HISPPD1), mRNA [NM_015216]	-0.61138619	0.14415381
NM_005900	SMAD, mothers against DPP homolog 1 (Drosophila) (SMAD1), transcript variant 1, mRNA [NM_005900]	-0.61069614	0.11685896
NM_002210	integrin, alpha V (vitronectin receptor, alpha polypeptide, antigen CD51) (ITGAV), mRNA [NM_002210]	-0.61046151	0.24473718
NM_004557	Notch homolog 4 (Drosophila) (NOTCH4), mRNA [NM_004557]	-0.60861037	0.09668976
NM_015534	zinc finger, ZZ-type containing 3 (ZZZ3), mRNA [NM_015534]	-0.60804523	0.01494462
AF292100	RP42 protein mRNA, complete cds. [AF292100]	-0.60767261	0.2667064
NM_00100225	C1q domain containing 1 (C1QDC1), transcript variant 1, mRNA [NM_001002259]	-0.60711457	0.16380957
AK124515	cDNA FLJ42524 fis, clone BRACE3001384. [AK124515]	-0.6064936	0.0603736
NM_031217	kinesin family member 18A (KIF18A), mRNA [NM_031217]	-0.60547327	0.17690896
BC022417	cDNA clone IMAGE:4243782, partial cds. [BC022417]	-0.60138069	0.12093723
